# Parallel and High Throughput Reaction Monitoring with Computer Vision

**DOI:** 10.1002/anie.202413395

**Published:** 2024-10-31

**Authors:** H. Barrington, T. J. D. McCabe, K. Donnachie, Calum Fyfe, A. McFall, M. Gladkikh, J. McGuire, C. Yan, M. Reid

**Affiliations:** ^1^ Department of Pure & Applied Chemistry University of Strathclyde Glasgow UK

**Keywords:** Computer Vision, High throughput, Reaction monitoring, Kinetics

## Abstract

We report the development and applications of a computer vision based reaction monitoring method for parallel and high throughput experimentation (HTE). Whereas previous efforts reported methods to extract bulk kinetics of one reaction from one video, this new approach enables one video to capture bulk kinetics of multiple reactions running in parallel. Case studies, in and beyond well‐plate high throughput settings, are described. Analysis of parallel dye‐quenching hydroxylations, DMAP‐catalysed esterification, solid‐liquid sedimentation dynamics, metal catalyst degradation, and biologically‐relevant sugar‐mediated nitro reduction reactions have each provided insight into the scope and limitations of camera‐enabled high throughput kinetics as a means of widening known analytical bottlenecks in HTE for reaction discovery, mechanistic understanding, and optimisation. It is envisaged that the nature of the multi‐reaction time‐resolved datasets made available by this analytical approach will later serve a broad range of downstream efforts in machine learning approaches to exploring chemical space.

## Graphical abstract

A novel computer vision‐based method for monitoring parallel and high throughput experimentation (HTE) is presented. Unlike previous methods, this approach captures the kinetics of multiple reactions in a single video. Case studies demonstrate its applicability in diverse chemical processes, offering insights into reaction discovery, mechanistic understanding, and optimization, with potential benefits for machine learning‐driven chemical exploration.

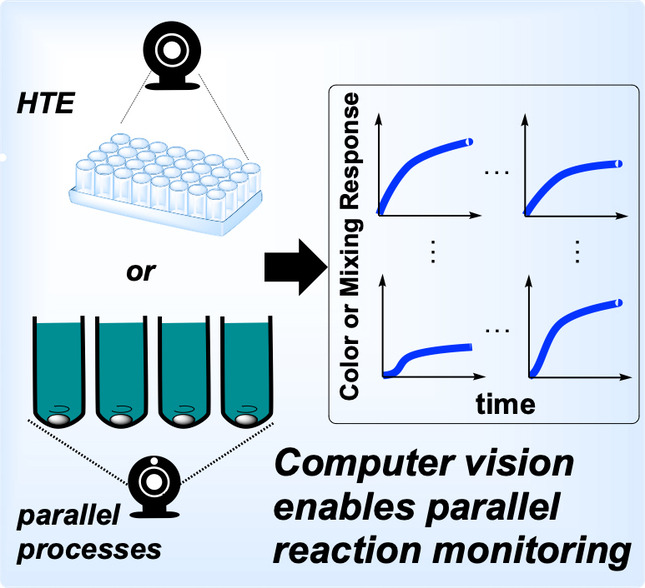

## 1 Introduction

**Parallel reaction monitoring and high throughput experimentation**. Parallel reaction analysis—either on a small scale or more formal high throughput experimentation (HTE)—has become an essential part of automated chemical discovery. Beyond the screening and optimisation of reactions towards new active pharmaceutical ingredients (APIs),[^1^, ^2^] HTE applications include (but are not limited to) materials discovery,[^3^, ^4^] phase separations,[^5^, ^6^, ^7^] solubility screening,[^8^, ^9^] and catalysis.[^10^, ^11^] HTE is used to perform many small‐scale experiments in parallel, typically using a carousel (ca. 8–24 experiments) or multi‐well (24, 96, 384) plate. It has been expanded to air sensitive chemistries, performed in 1536 well plates, and integrated into automated workflows.^[12]^ Efforts to conduct high throughput kinetics in flow have also recently been pioneered.^[13]^ Indeed, the increasing need for adoption of parallel reaction and HTE methods, for both yield prediction and more detailed kinetic analysis, extends to machine‐learning research where large, multidimensional experimental datasets are valuable from the perspective of accurate model development.[^14^, ^15^, ^16^]

While the ability to run experiments in parallel or high throughput is certainly important, the need to analyse each experiment appropriately deserves similar emphasis. In the above HTE examples, analytics span the use of NMR, HPLC, mass spectrometry, protein crystallography, and single image processing. It is the last of these analytical methods that represents the core theme of the parallel reaction monitoring developments reported in this paper.

**Computer vision in analytical chemistry (CVAC)**. CVAC is a developing field, wherein cameras are used to quantify the visual characteristics of chemical samples.[^17^, ^18^, ^19^] Such analyses typically make use of commercially available cameras, such as those installed in smartphones,[^20^, ^21^, ^22^] webcams,^[19]^ or standalone digital cameras.^[23]^ Computer vision applications in chemistry typically use colour analysis to indicate chemical concentration. The colour of blood, for example, is determined by the presence, speciation, and concentration of haemoglobin.^[24]^ Under controlled conditions, such as in a food manufacturing context, the colour of a solid's surface can indicate the presence of specific chemicals.^[23]^ However, CVAC has, until relatively recently, rarely seen use beyond identification tests and quality control via single image analysis. One reason for this limitation is perhaps rooted in the fact that the software needed to extract information from single images is more commonplace than that needed to analyse more computationally‐demanding video data. While the majority of applications of CVAC have utilised single images, we and others have focused on exploiting video recording to monitor reaction colour over time.[^5^, ^25^, ^26^, ^27^, ^28^]

**Computer vision in parallel reaction analysis**. Computer vision has been appreciably exploited in chemical applications,[^21^, ^22^, ^29^, ^30^, ^31^, ^32^] and especially in biological settings,[^33^, ^34^, ^35^, ^36^, ^37^, ^38^, ^39^, ^40^, ^41^] where HTE (in the formal sense, running reactions on well plates) has matured at a more rapid pace owing to the advantageous constraint of working in aqueous media under near‐ambient conditions. Several teams have made use of specialised hardware, creating micro‐lens arrays,[^29^, ^31^] to better view each well on the plate, or using mounted cameras which move to photograph each well independently.^[38]^

**Kineticolor**. Our team's ongoing contributions to CVAC are centred on the development of *Kineticolor*, a software principally designed to turn video input into kinetic information. We previously investigated the applicability of this reaction monitoring approach in the identification of controlled substances,^[42]^ the analysis of palladium catalyst degradation,^[43]^ mixing effects,[^44^, ^45^] and solid phase peptide synthesis.^[28]^

**Figure 1:**
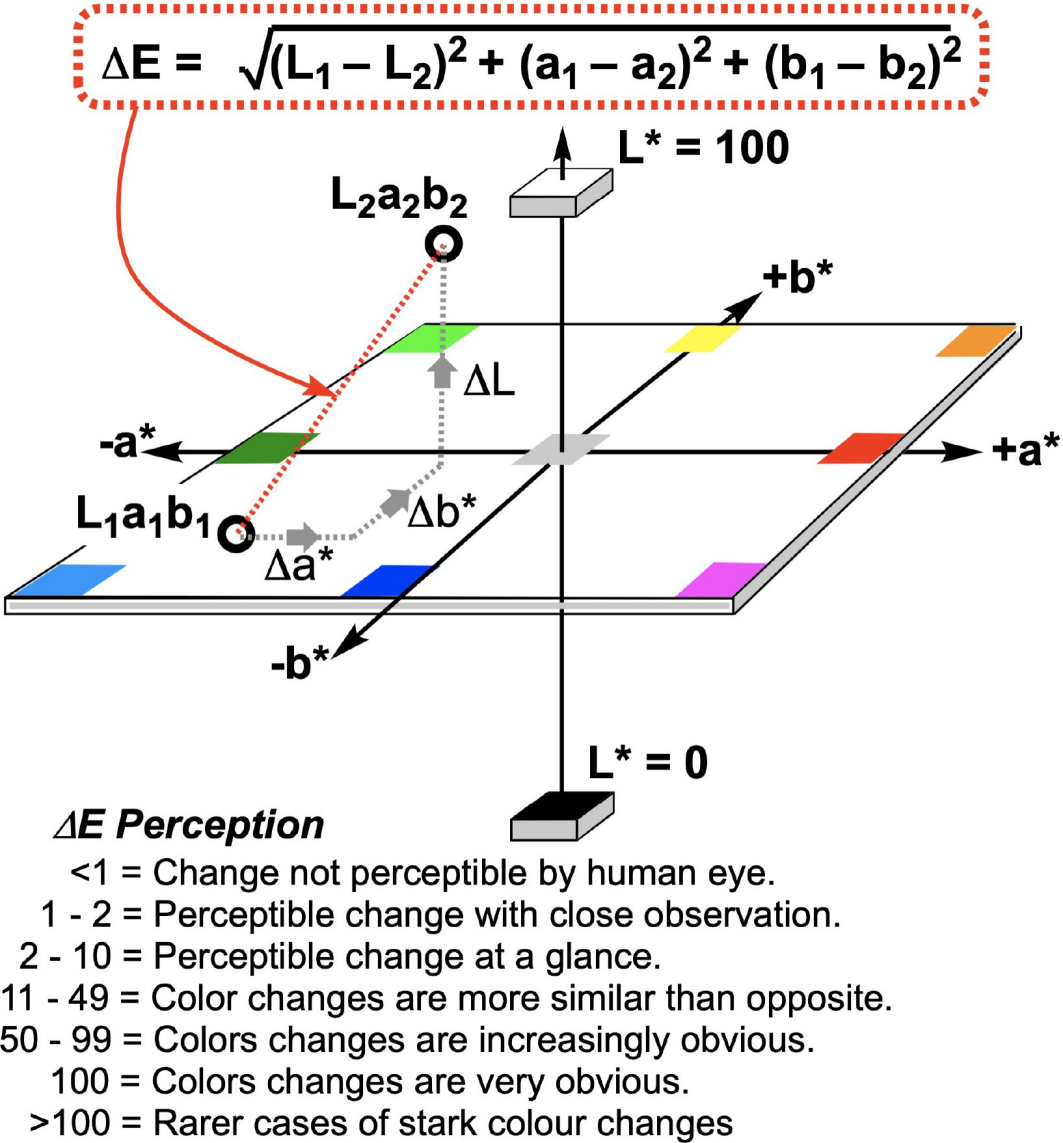
The CIE–L*a*b* colour space is a three‐dimensional map of colour, designed to be perceptually uniform. Measuring the absolute distance between two points in this space provides a reliable measure of difference between colours, ΔE .

*Kineticolor* records the average colour of a user‐selected region of interest from within the video of the chemical reaction. The process begins by recording the colour using a triplet of values from the RGB (red, green, blue) colour model. The combination of the R, G, and B values together represent one colour. This numerical representation of colour can then be converted to other colour spaces,^[17]^ such as HSV (Hue, Saturation, and Value) or CIE‐L*a*b* (covering lightness, a green‐red axis, and a yellow‐blue axis), enabling alternative perspectives on the same data. The CIE‐L*a*b* colour space is especially useful, as it is designed to be perceptually uniform;^[46]^ a colour change of 10 units along one axis is perceptually equivalent to a change of 10 units along any other axis. In other words, what one sees by‐eye maps more intuitively to the L*a*b* space than, say, the RGB space. This gives rise to one of the most versatile metrics presented by *Kineticolor*, ΔE , a colour‐agnostic measure of colour change (Figure 1). By comparing each frame of a video to the first frame, the change over time can be captured using ΔE .

**Figure 2:**
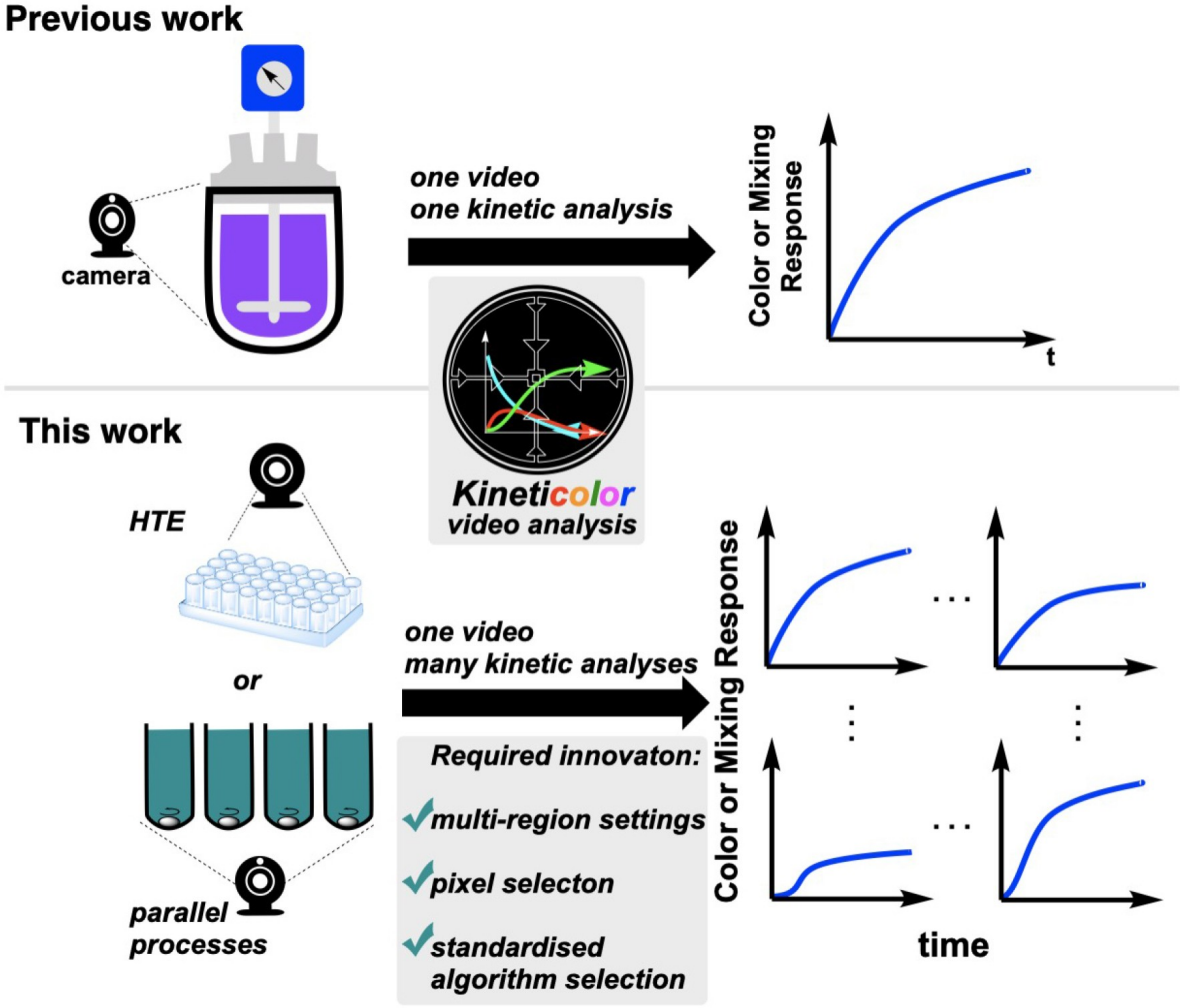
Aim of the current investigation versus prior art. Evolving our computer vision approach from a one‐reaction‐at‐a‐time analytical tool to one capable of parallel reaction analysis necessitated innovations in enabling flexible arrangements of multiple wells, new algorithms for pixel selection, and standardisation of computer vision analysis algorithm selection.

**Aims**. Further to these software developments, the current study was triggered by an unmet need to analyse more than one reaction captured in a single video. With the potential value of such multi‐reaction computer vision methods for HTE practitioners, automation experts, and digital chemists, expanding *Kineticolor* with functionality applicable to HTE became the main focus of this investigation (Figure 2). In the form of a question, we asked:

“*What additional insights might we gain from being able to select multiple regions of the same video to extract the kinetics of each reaction in each region, compared to our established single‐region, one‐video‐one‐reaction method, and how might this impact the efficiency of data generation?*”

## 2 Results and Discussion

### 2.1 Method Development with Dye Hydroxylation

**Figure 3:**
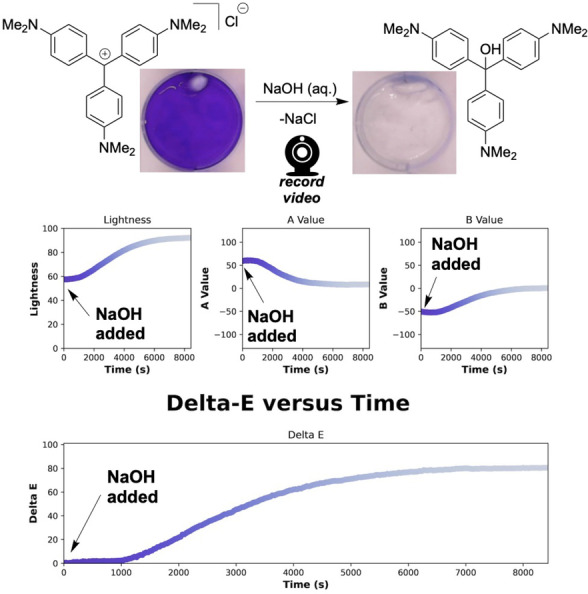
Top: Exemplar trityl cation hydroxylation and its representative colour change. Middle: Example kinetic outputs from quantifying the pixels in terms of the CIE–L*a*b* colour space. Bottom: The contrast parameter ΔE versus time, using the first video frame as the reference from which all other frames were measured.

Inspired by the educational computer vision work of Penn et al.,^[47]^ crystal violet hydroxylation was chosen as a simple reaction through which to evolve our software, from a one‐camera‐one‐reaction method to one in which a single video could be segmented to capture kinetics from many reactions, running in parallel. Hydroxylation of the crystal violet trityl cation produces a stark colour change, from purple‐blue to colourless (Figure 3). The manner in which this change is recorded by a camera is dependent on the starting concentration of crystal violet, lighting, and on the background colour, since the solution becomes translucent as the reaction progresses. While human perception can recognise a colourless, translucent solution, a digital camera simply records the colour of the background behind the increasingly translucent solution. In relation, the observable apparent induction period in reaction is likely to be a combination of physical (lighting), analytical (limits of detection for very dark solutions), and chemical (mixing and diffusion) effects. The nature of such kinetic profiles thus varies with experimental and photographic set‐up, for a given set of chemical conditions. Consequently, the most useful metric to extract from such time‐resolved computer vision data is often (but not exclusively) end point time.

*Kineticolor* analysis of the hydroxylation video recording captures its path through the L*a*b* colour space, as the colour becomes lighter (more positive on the L* axis), greener (more negative on the a* axis), and yellower (more positive on the b* axis) relative to the starting point. On completion, the reaction settles on the colourless centre of both the a* and b* axes (Figure 3).

### 2.2 Investigating Camera and Lighting Positions

**Figure 4:**
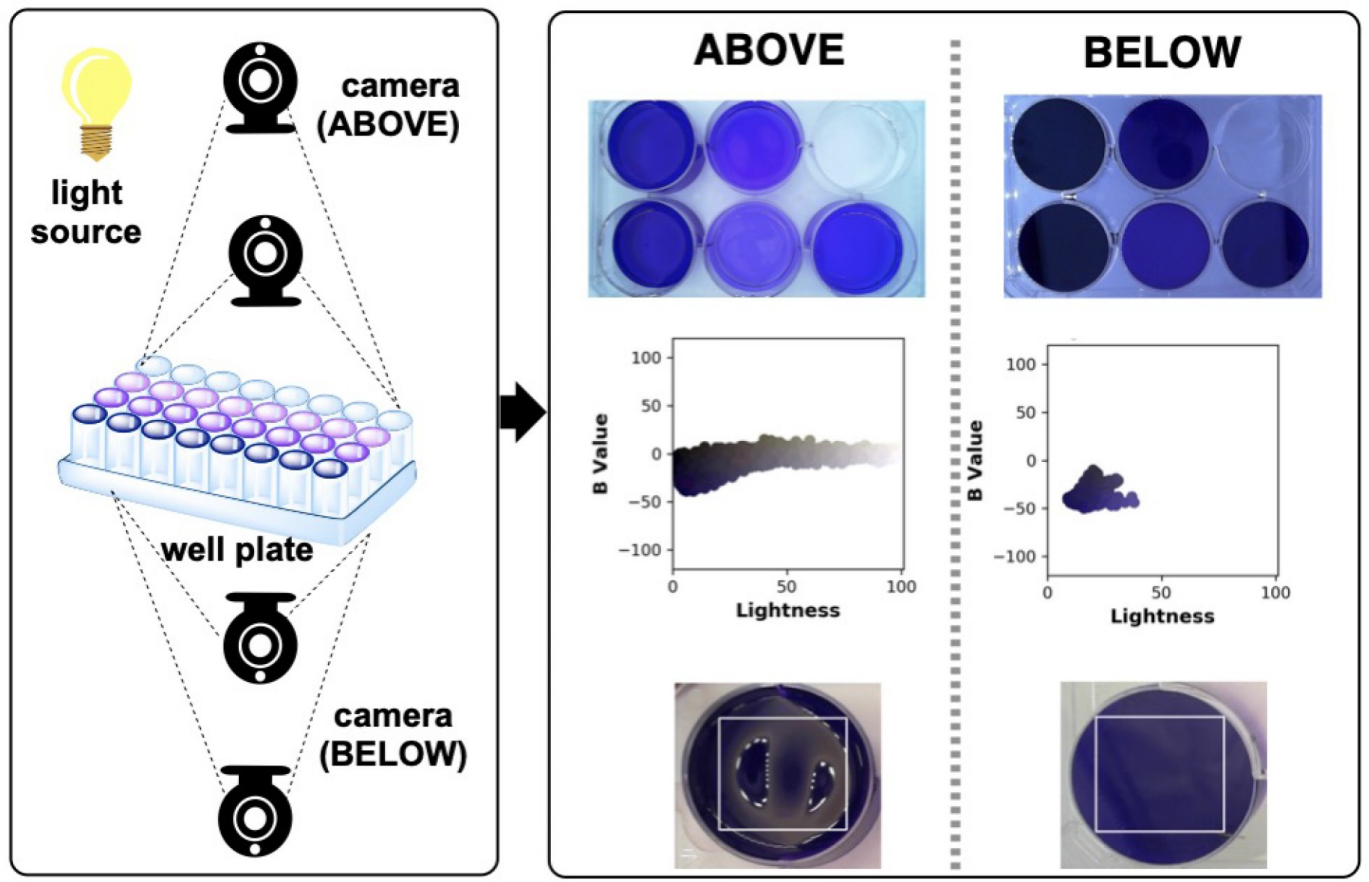
Left: Camera positions tested relative to well plate and light source. Right: Exemplar images of a 6‐well plate photographed from above the plate (same side as the light source) and below the plate (opposite side o the light source). Plots of b* versus Lightness help quantify the increased glare when filming from the same side rather than opposite that light source.

We explored applicable video recording set‐ups to enable suitable software developments of high throughput kinetic imaging. First, we compared positioning of a camera either opposite or on the same side as the light source, below or above a clear plastic well‐plate, respectively. We used *Kineticolor* to analyse single images of wells containing crystal violet and thus assess the distribution of colour across each well. To do this, the pixel data from within the user‐selected region of interest was presented as a 2D plot of L* (Lightness) versus b* (blue‐yellow axis from CIE–L*a*b*). The tighter the distribution of data points on this 2D colour plane, the less susceptible to glare was that particular video recording environment for the chemistry under study. This approach, shown in Figure 4, enabled us to quantify surface glare and thus choose the video recording environment that minimised its influence.

We ran reactions in 6, 24 and 96 well plates with varying combinations of video recording conditions. From these data, we determined that filming from below (opposite the light source) and further away from the plate (e.g. 43 vs 11 cm) gave the best results in terms of capturing more uniform colours, free from glare (Figure 4). Through this example, it is important to note that such investigations are lighting‐, chemistry‐, and camera‐specific. We recommend considering all elements of a computer vision reaction monitoring set‐up on a case‐by‐case basis, depending on the application.

**Figure 5:**
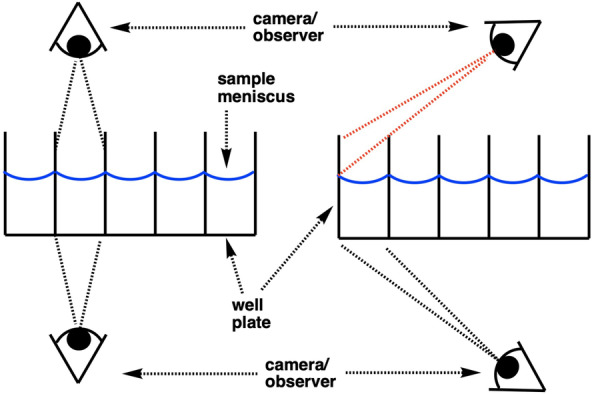
Video recording a well plate from below more reliably records a colour representative of the samples in each well.

Beyond glare, a parallax error can occur when the angle of the camera relative to the object moves further and further from the point perpendicular to the object being viewed. It can obscure a camera's cross‐sectional view of solutions from above a well plate. This error can be adequately managed by filming from below (Figure 5), where the camera is opposite the light source. It can also be managed by video recording the well plate from a greater distance. While beyond the scope of this study, others have managed the parallax error by using bi‐convex lenses placed between the camera and well plate, for single image applications.^[20]^

**Figure 6:**
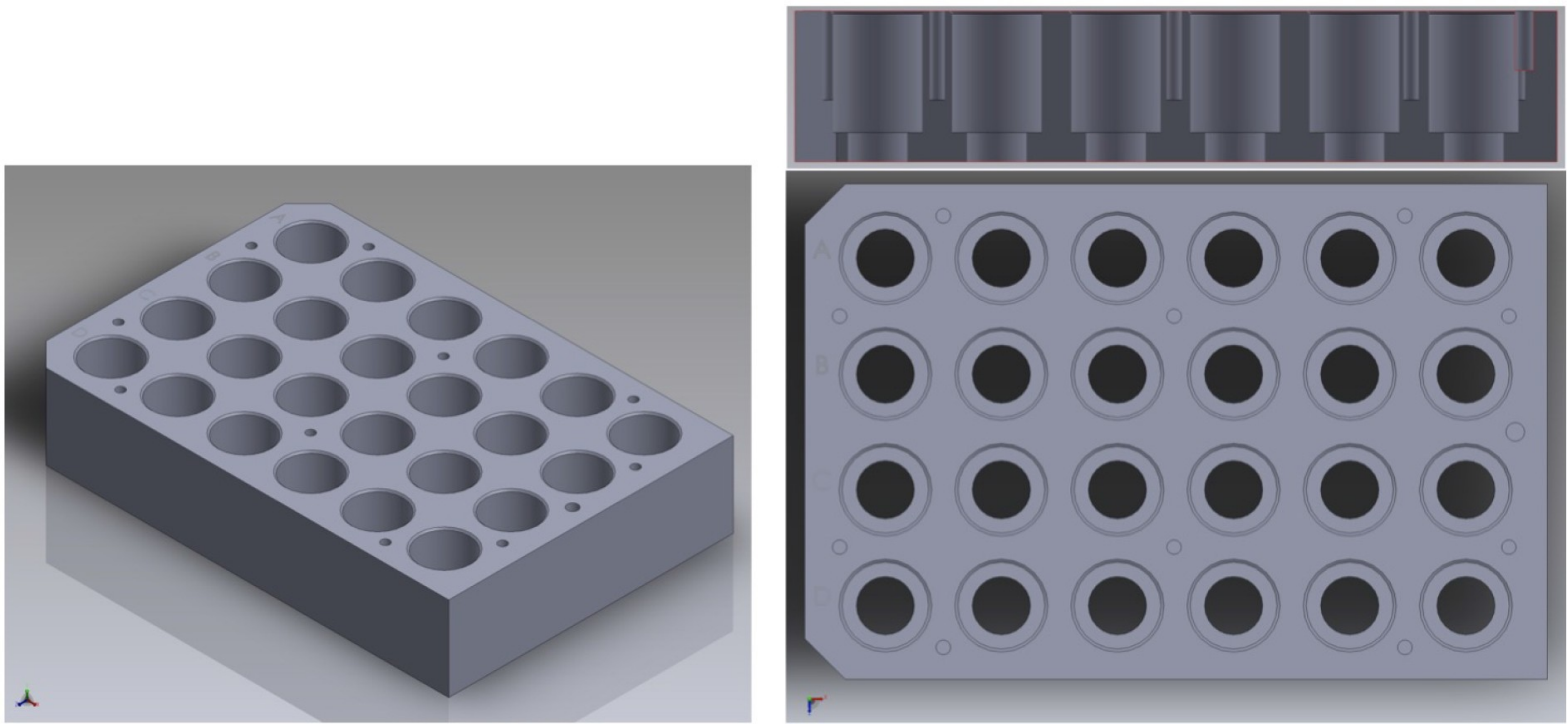
CAD renderings of bespoke 24‐well metal plate engineered to enable viewing of vial contents from below the plate.

When observing a plastic, transparent well plate (those typically used in biological applications), light from the sides can brighten the appearance of the outermost wells. However, since using glass vials in a metal well plate is necessary for comparatively more elaborate chemistry applications,^[12]^ deeper consideration of sufficient lighting is required. For this reason, and to explore our parallel reaction monitoring approach in metal well plates, we engineered a bespoke 24‐well stainless steel plate, with drilled holes at the bottom/centre of each well. Through these holes, we could set‐up the camera to observe colour changes occurring in the vials (Figure 6).

**Figure 7:**
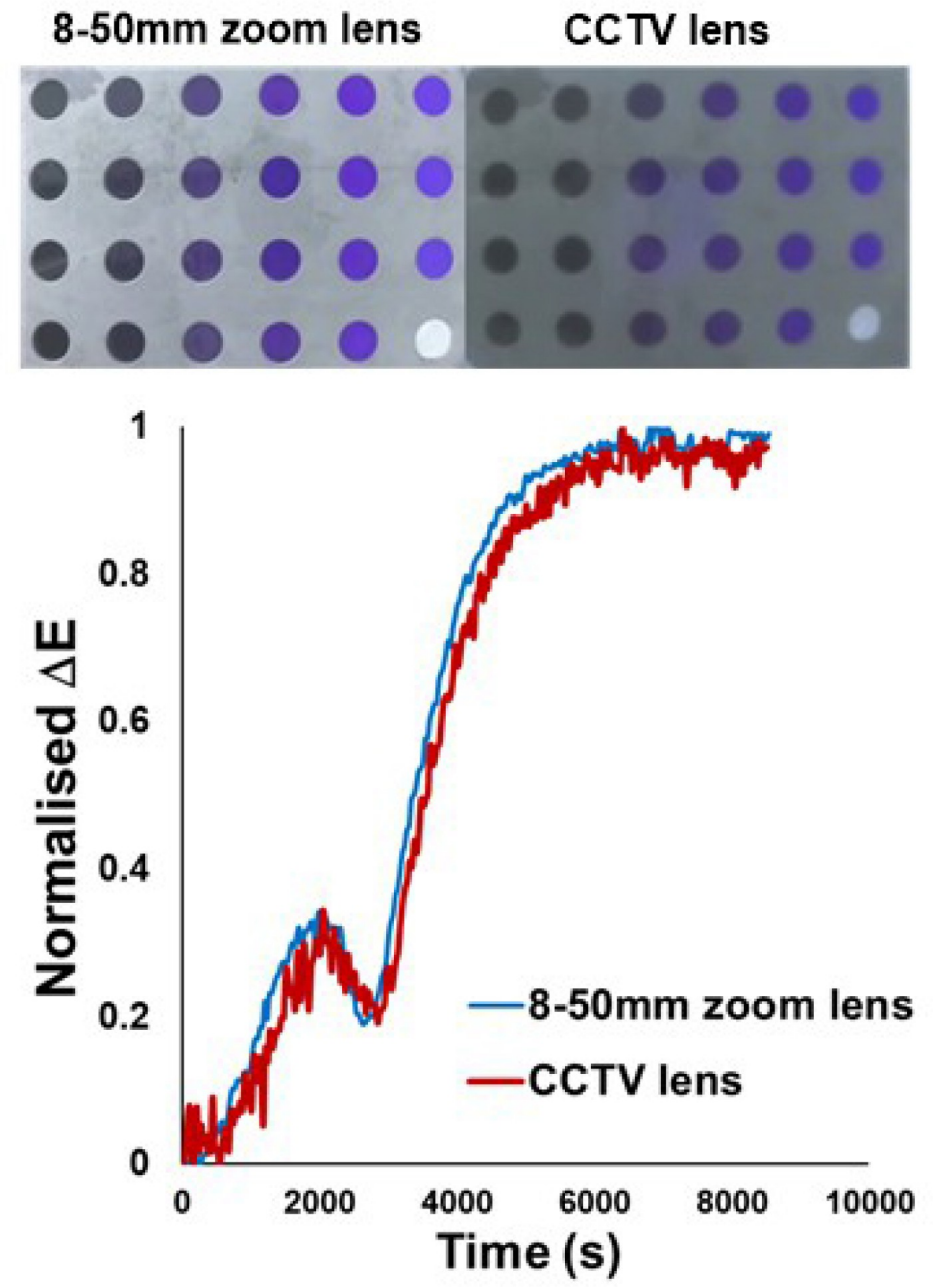
The clearer 8‐50mm zoom lens (top‐left) produces a smoother ΔE profile when compared to the CCTV lens (top‐right), with which the camera was unable to properly focus on the well plate. The CCTV lens produced noisier images, which directly impacted the quality of the ΔE profile. ΔE profile comparisons for each well can be found in the Supporting Information.

Closer camera positioning relative to the well plate can also lead to distortions in the field of view that can exacerbate aforementioned parallax issues. Out of focus images are blurrier and noisier. Such diminished image quality can be caused, for example, by using a lens whose depth‐of‐field is too shallow, resulting in the position of focus in front of the camera lens being mismatched relative to the position of the object of interest. Ultimately, such photographic errors produce noisier time series data, as displayed in Figure 7 for hydroxylation reactions run in the engineered well plate. The supporting information shares additional related studies, demonstrating how the kinetic outputs can be tuned with the use of band‐pass filters to block certain wavelength ranges from reaching the camera sensor.

### 2.3 High Throughput Reaction Monitoring with *Kineticolor*

**Figure 8:**
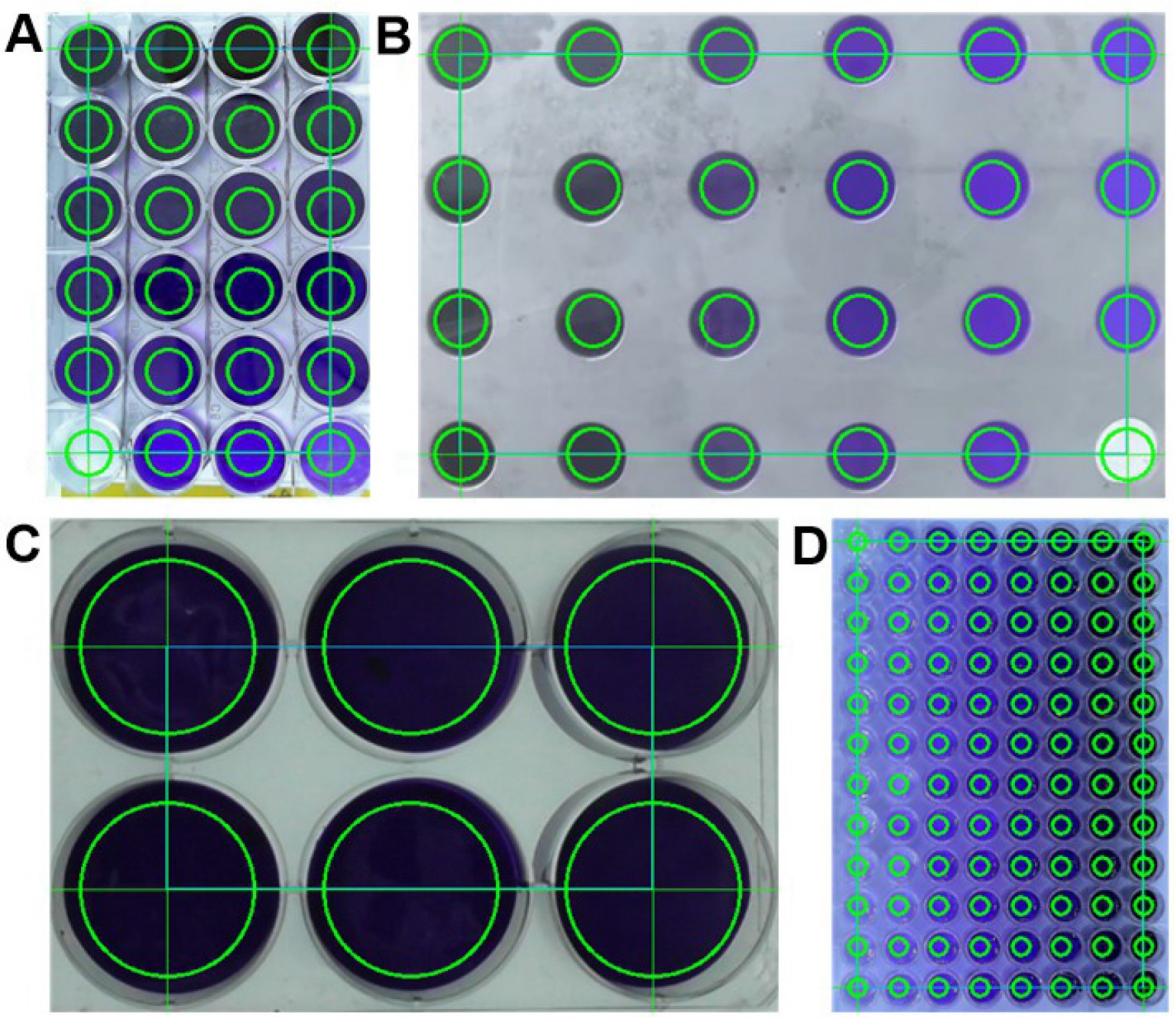
Examples of user‐guided multi‐ROI selection. A: a 4x6 selection for a plastic 24‐well plate. B: A 6x4 selection for a metal 24‐well plate. C: A 2x3 selection for a plastic 6‐well plate. D: A 12x8 selection for a plastic 96‐well plate.

**Beyond the single region of interest**. For all previous iterations of our computer vision work with *Kineticolor*, it has been sufficient to select a single, rectangular region of interest (ROI), to define the area of pixels to be included in any subsequent analysis.[^28^, ^42^, ^43^, ^44^] For the current HTE and parallel reaction monitoring work, it was necessary not only to encode multiple ROIs in a single analysis, but also enable circular (as opposed to square) ROIs, to account for typical well shapes. While we,^[44]^ and others,[^22^, ^27^, ^48^] have explored automated object recognition approaches to region of interest selection, this current work was best served by enabling full, user‐selected control of the number, diameter, and row‐by‐column dimensions of ROIs selected (Figure 8).

**Experimental testing of multi‐ROI analysis**. To test *Kineticolor*’s new multi‐region analysis functionality, a selection of crystal violet hydroxylation experiments were performed in the various well plate formats, using a range of starting concentrations for both crystal violet and sodium hydroxide. The outputs from a 24‐well plate example are summarised in Table 1. Using the multi‐ROI approach, all kinetics were analysed simultaneously for each well, from a single input video file. To approximate reaction end time, computational plateau analysis was performed,^[28]^ finding the time at which the gradient of the ΔE profile fell below a set threshold, in this case 0.05 ΔE .s^−1^. We previously applied related plateau analyses for monitoring amidation^[28]^ and mixing processes.^[45]^

**Table 1:** Initial concentrations of crystal violet and NaOH in each well. Left: The initial crystal violet experiment shown at the start (left), and after 90 minutes (right). Right: A summary of reaction end point time, derived from imaging data, versus ln ([NaOH]_0_/[CV]_0_).

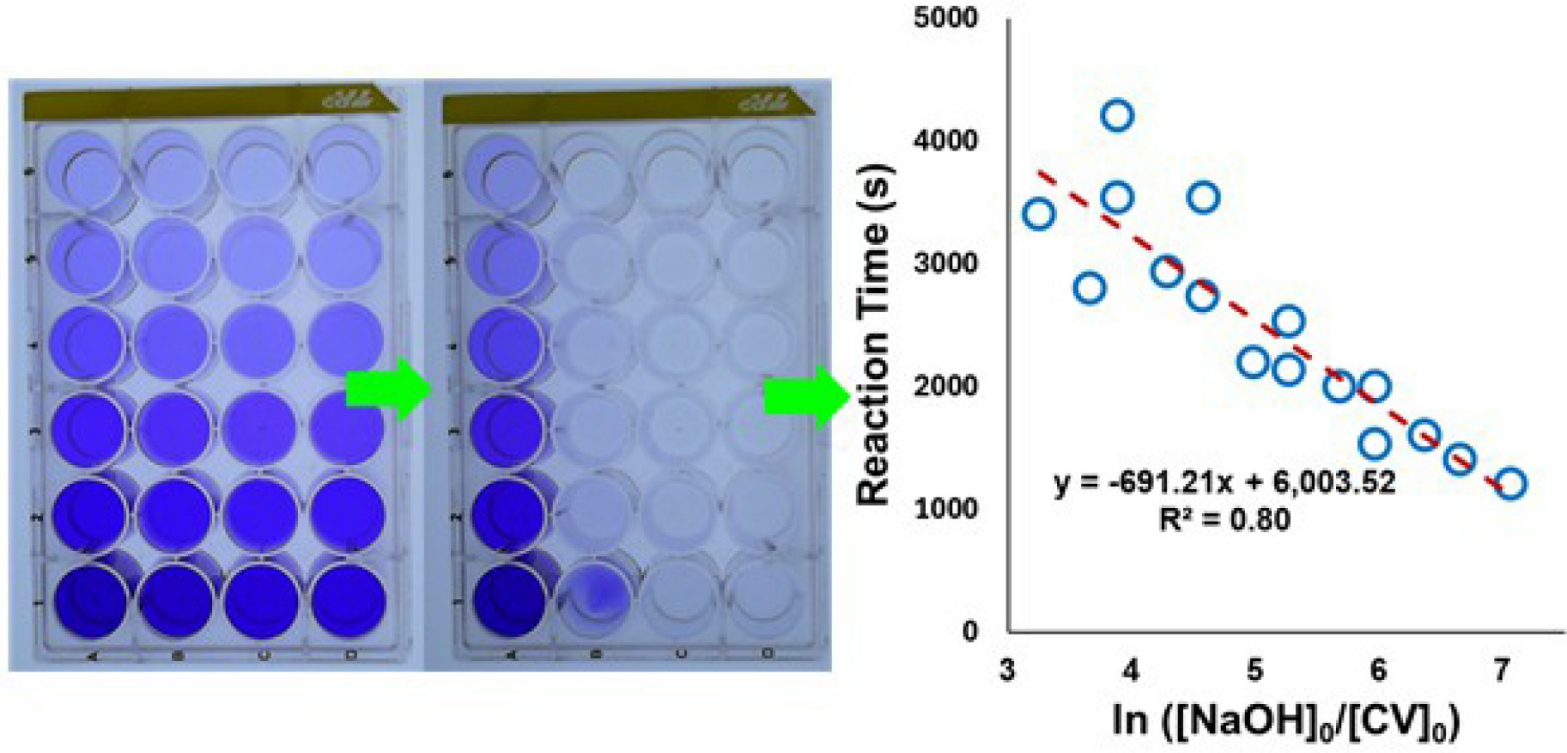			
Well	CV(mM)	NaOH(mM)	End Point(s)
0	0.000	0.00	N/A 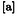
1	0.017	6.67	1534
2	0.017	13.3	1401
3	0.017	20.0	1201
4	0.034	0.00	N/A 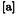
5	0.034	6.67	2134
6	0.034	13.3	2001
7	0.034	20.0	1601
8	0.068	0.00	N/A 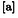
9	0.068	6.67	3535
10	0.068	13.3	2534
11	0.068	20.0	2001
12	0.136	0.00	N/A 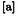
13	0.136	6.67	4202
14	0.136	13.3	2734
15	0.136	20.0	2201
16	0.272	0.00	N/A 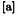
17	0.272	6.67	N/A 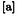
18	0.272	13.3	3535
19	0.272	20.0	2934
20	0.510	0.00	N/A 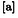
21	0.510	6.67	N/A 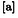
22	0.510	13.3	3401
23	0.510	20.0	2801

[a] No plateau detected/reaction incomplete within 90 minutes.

### 2.4 Assessing Mixing Parity Across a Well Plate

For a range of chemistries applied in HTE environments, it is known that mixing effects are an important consideration for placing results in a fair, unbiased context.[^12^, ^49^] On a well plate, the nature of the choice of mixing (or, indeed, shaking) method may not have uniform influence over each and every well on the plate. Based on our previous investigations of mixing with computer vision,^[44]^ we envisaged being able to use colorimetric bulk reaction rates to capture any macroscopic mixing differences across a well plate charged with the same stirring and chemical conditions.

**Figure 9:**
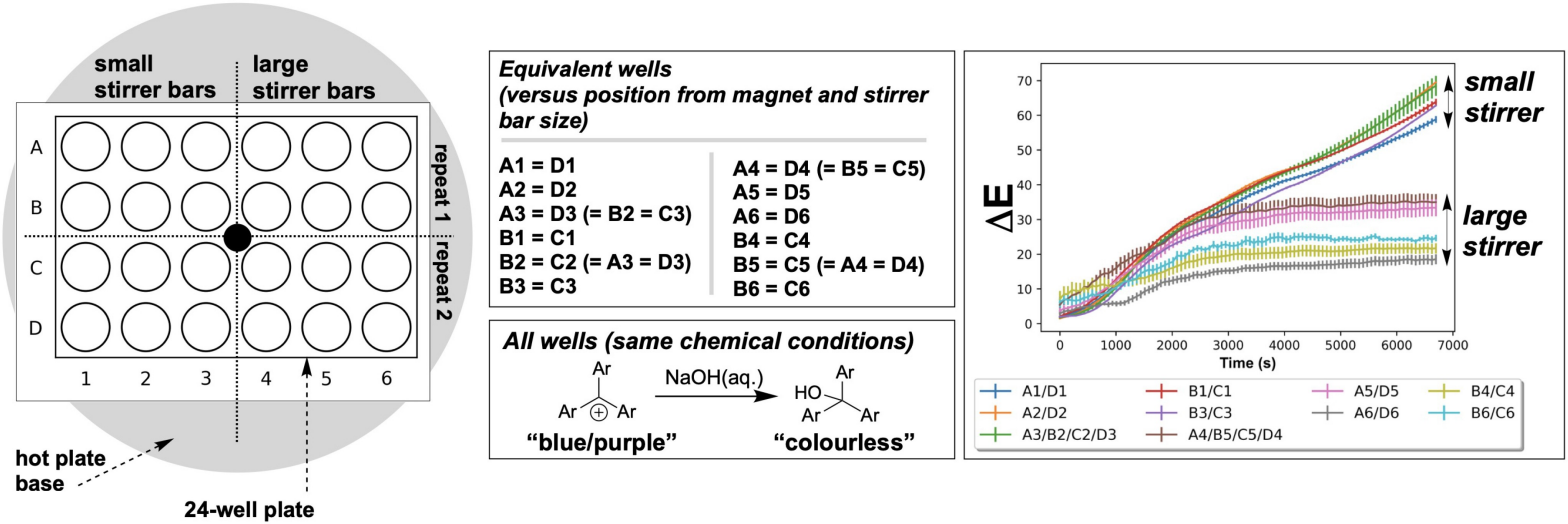
Left: A birds eye schematic view of a 24‐well plate, showing the position of the hotplate magnet (black dot). Also visible is the vertical segmentation of the wells into those containing small and large stirrer bars and horizontal segmentation into repetitions of a given experimental condition. Centre: A list of wells deemed equivalent in terms of their likely mixing profile, relative to the stirrer bar and position relative to the central magnet. All wells were charged with identical chemical conditions, running the crystal violet hydroxylation. Right: Δ*E* profiles from *Kineticolor* depicting mixing differences from across the well plate. The double‐headed arrows are a guide to the eye, showing that there is variation in the kinetics between wells on the plate, despite all wells containing equivalent chemical conditions.

As an extension of the proof‐of‐concept for multi‐region analysis shown in Table 1, we charged a 24‐well plate with stoichiometrically identical conditions of the crystal violet hydroxylation reaction. The only set‐up differences from well to well were: (i) the size of stirrer bar employed, and (ii) the distance of the well from the centre of the central stirring magnet inside the hotplate stirrer. The ΔE profiles in Figure 9 exemplify how this same multi‐region computer analysis tool records mixing differences across a single well plate. Here, if all wells were mixed equally, all ΔE profiles should overlap when, in fact, they do not. While beyond the scope of this current investigation (an our available resources), the same analytical approach could feasibly be used to assess other stirring systems (e.g. tumble stirrers).

### 2.5 Applications of High Throughput Imaging

**Figure 10:**
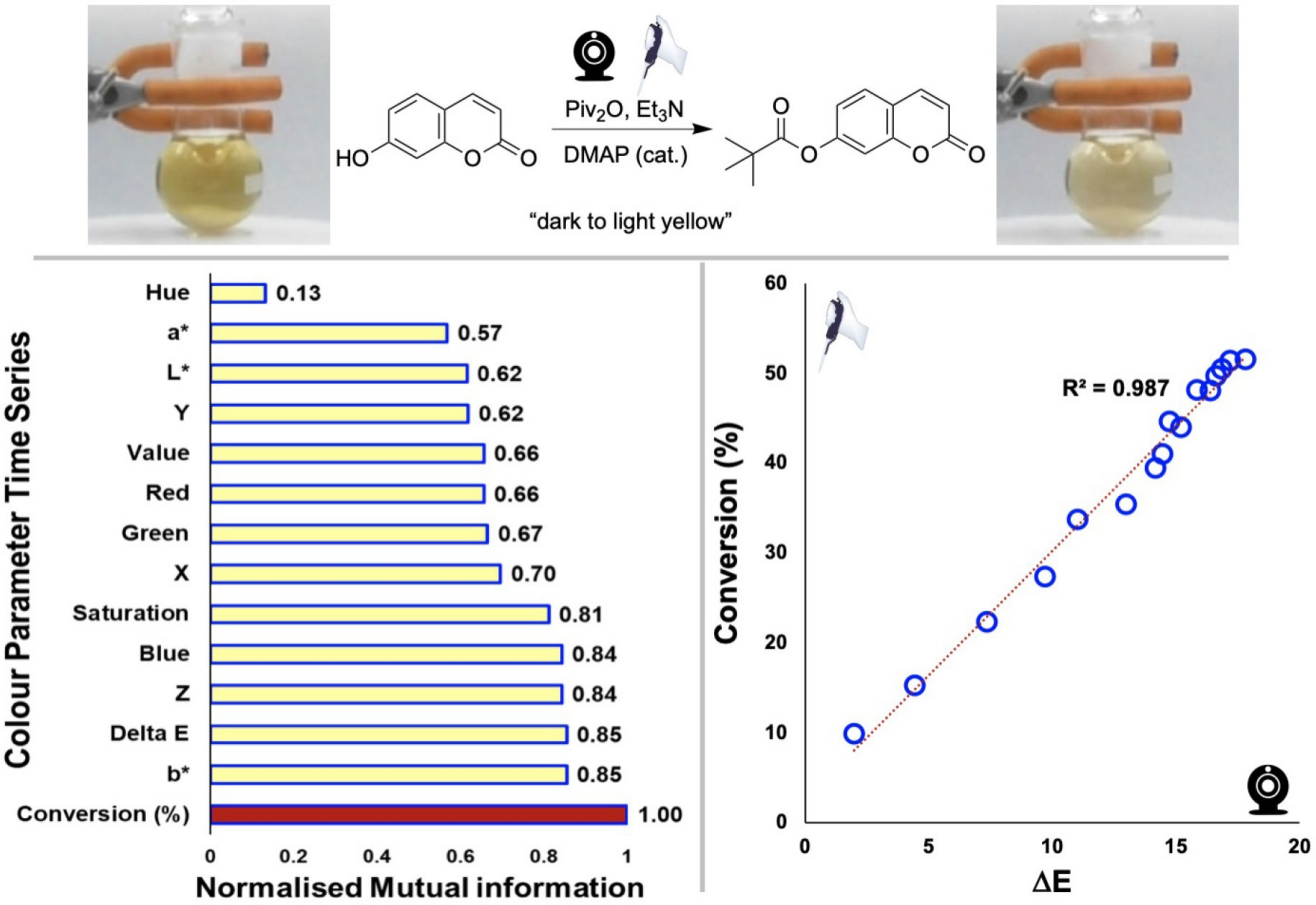
Top: Umbelliferone esterification and images of the bulk reaction colour at 0 and 4000 seconds. Bottom left: ranked and normalised mutual information scores for colorimetric time series based on their likelihood of holding information about product conversion as measured by HPLC. Bottom right: exemplar linear correlation plot between ΔE and HPLC conversion.

**Combining HTE computer vision with HTE robotics**. Inspired by use of the reaction in developing dielectric spectroscopy,^[50]^ we explored a DMAP‐catalysed esterification of umbelliferone (Figure 10 and Table 2). First studying a single reaction in batch, we co‐monitored the esterification with sampling for offline HPLC and with our camera‐based method. Then, using a statistical workflow first established in our earlier research,[^28^, ^43^, ^44^] we used mutual information analysis to rank‐order the colour parameter time series, calculated in *Kineticolor*, most likely to correlate with HPLC (Figure 10, bottom). This approach is useful because it enables exploration of correlations between parameters without assuming any linearity. Further details are provided the supporting information. In this case, the commonly‐used contrast metric ΔE was high in mutual information with respect to HPLC‐measured product conversion, and also gave a linear correlation (Figure 10, bottom).

**Table 2:** Graphic: Photos of the 24‐well plate before and after esterification, with the colour bar illustrating the subtle change in colour over time.

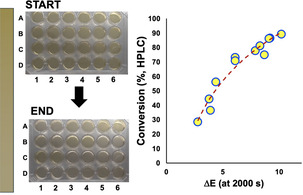			
Well	Piv_2_O: DMAP 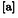	ΔE 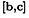	Conv.(%) 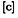
A1+A2	0.05: 0.5	2.8	28.5
A3+A4	0.05: 1.0	4.4	56.1
A5+A6	0.05: 1.5	6.1	73.1
B1+B2	0.05: 2.0	7.9	77.7
B3+B4	0.10: 0.5	3.9	36.5
B5+B6	0.10: 1.0	6.1	70.8
C1+C2	0.10: 1.5	8.3	81.2
C3+C4	0.10: 2.0	9.2	86.5
C5+C6	0.15: 0.5	3.8	44.4
D1+D2	0.15: 1.0	8.7	74.9
D3+D4	0.15: 1.5	9.1	86.1
D5+D6	0.15: 2.0	10.2	89.1

[a] Relative to 1 equiv. of umbelliferone. [b] At 2000 s, guided by computer vision kinetic analysis. [c] Average of two runs.

With sufficient statistical evidence that the rate of colour change in umbelliferone esterification was predictive of reaction conversion, we transferred the reaction to a well plate environment. Here, we used the OpenTrons OT‐2 robot to charge the wells on the plate in an automated fashion, before the plate was transferred to a light box for computer vision kinetic analysis. In this way, we were able to demonstrate the value in high throughput colour kinetics in mapping out the impact of increasing pivalic anhydride and DMAP catalyst concentrations on the overall rate of conversion to product (Table 2). This case study was also valuable to demonstrate the ability to use the camera‐enabled approach to capture and quantify what are, by‐eye, very subtle colour changes evident in the formation of the ester. Future efforts may focus on combining the charging and camera monitoring of the well plates within the robot itself, without transfer.

**Figure 11:**
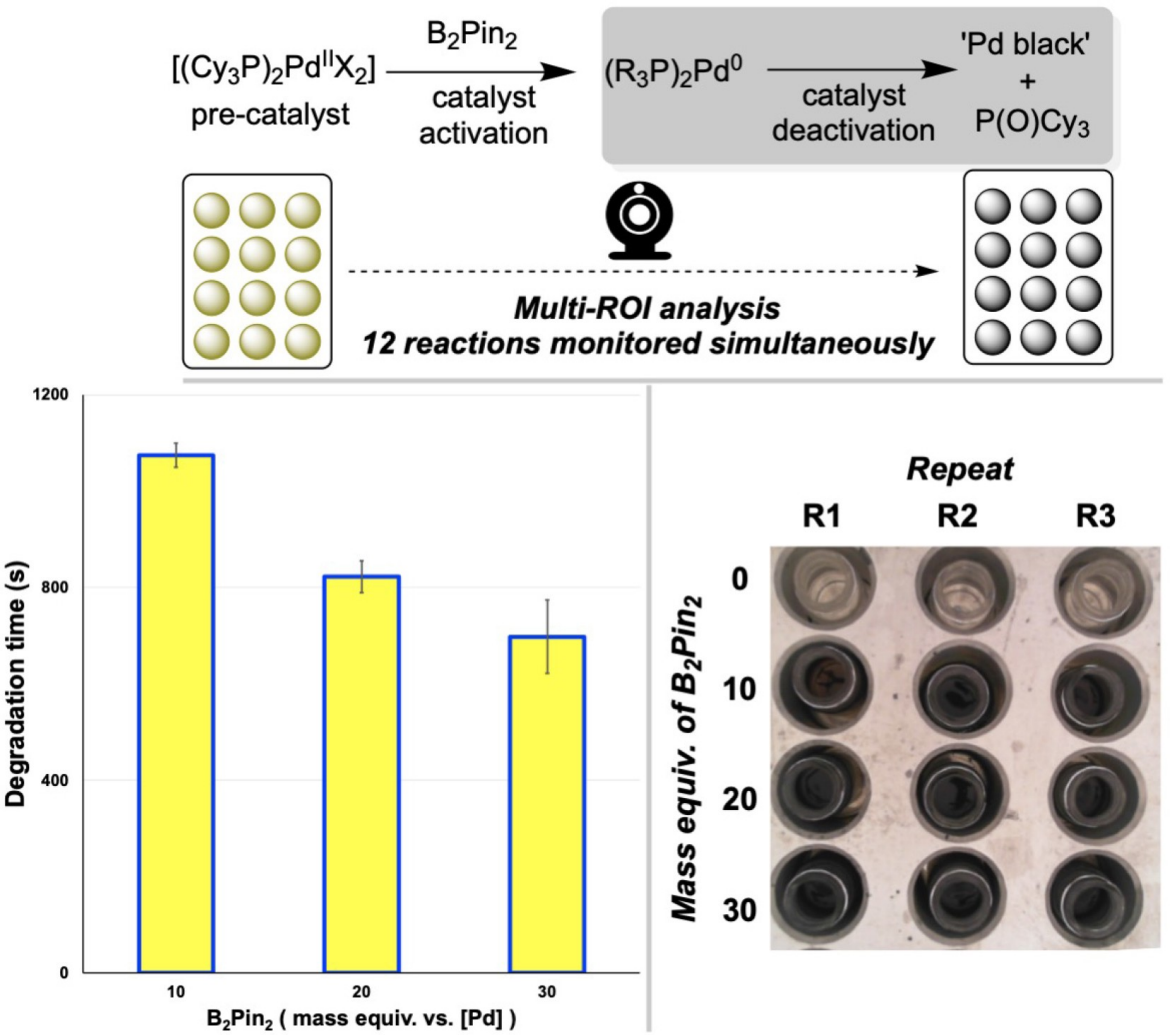
Parallel kinetic analysis of Pd(II) precatalyst degradation to form Pd black. Bottom left: Degradation time decreases with increasing [B_2_Pin_2_].

**Mapping Pd(II) precatalyst degradation**. In earlier work, we reported a computer vision approach to monitoring Pd‐catalysed Miyaura borylation and related catalyst degradation processes.^[43]^ There, we employed a one‐reaction‐at‐a‐time approach to mapping out the influences of boron and catalyst concentration, solvent, and temperature on the rate of Pd‐black formation. For the current study, we modified our previous method, recording a video of the Pd‐black formation, carried out using a multi‐stirrer system, rather than the single reaction vial used before. This change in set‐up, combined with the new multi‐region selection tools in *Kineticolor*, enabled us to record the twelve required reactions simultaneously. The resulting order of reactivity—with increasing concentration of B_2_Pin_2_ leading to more rapid Pd‐black formation—was consistent with the results of our one‐reaction‐at‐a‐time computer vision approach reported previously (Figure 11). Having collected all 12 data points in one 3,500 s recording rather than twelve recordings totalling 42,000 s, the ability to study the Pd‐black kinetics in high throughput resulted in a 92 % time saving.

**Monitoring sedimentation in metal commodities—parallel reactions without the well plates**. By developing an approach within *Kineticolor* that enables analysis of multiple regions of the same video, we were keen to explore applications of the approach beyond formal HTE and well‐plate environments. We envisaged that the approach could be equally valuable to those aiming to capture kinetics from any number of parallel reactions (not necessarily 24, 96, or more) in a wide variety of reaction vessels.

In collaboration with a local metal commodities manufacturer in Scotland, we learned of challenges in synthesising high quality copper (II) acetate on scale, without ingress of impurities (including polymeric filter aids) that would compromise downstream product price point. Traditionally, the presence of solid impurities in filtrate samples from the manufacturing plant are graded according to a by‐eye test of how long it takes for a visibly turbid sample to clear, as solid settles to the bottom of the transparent sample vessel. These tests are performed in series, one at a time. During a single test, the longer the apparent time taken for the turbid sample to settle, the lower the quality grading awarded to the product batch. Such sedimentation phenomena and measurement of settling times is of broader interest in fields as diverse as biochemical manufacturing,^[51]^ food processing,^[52]^ wastewater treatment,[^53^, ^54^] CO_2_ capture,^[55]^ and mixing analysis of solids suspended in batch reactors.^[44]^

**Figure 12:**
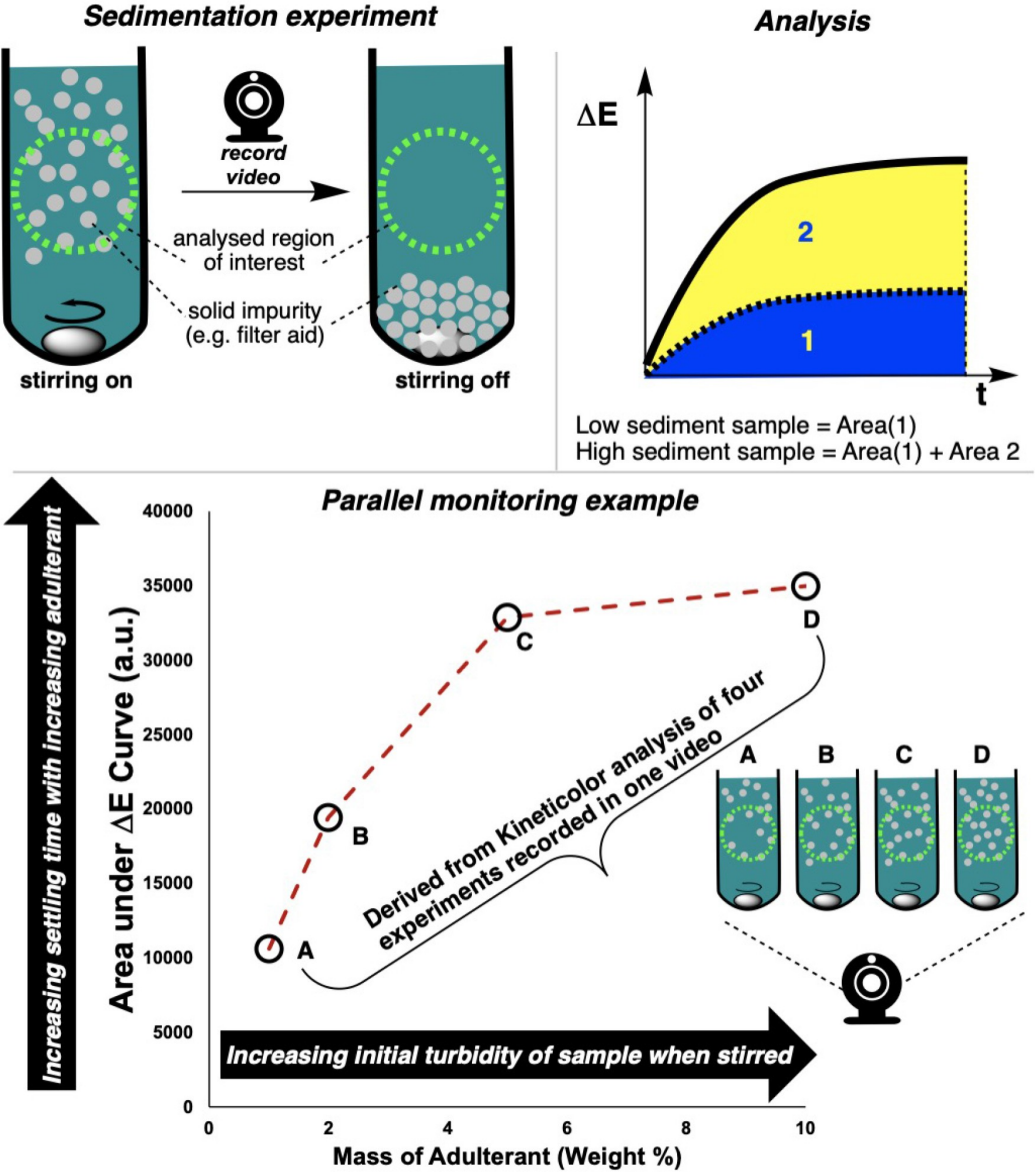
Use of multi‐region Kineticolor analysis to provide sedimentation analysis of four samples running in parallel. Top left: Schematic of the computer vision experiment. Top right: Intuition for the meaning of area under curve in the current context. Bottom: Analysis of four adulterated copper acetate samples, the data for which were collected in a single video file of all four processes being run simultaneously.

As part of our effort to explore the applications of multi‐region video analysis, we modified the industrial sedimentation test, and video recorded several samples being stirred and then left to stand, side by side, in parallel (Figure 12). Here, it was possible to show that the area under the ΔE –time curve increased with increasing mass of adulterant Figure 12, bottom).

**Figure 13:**
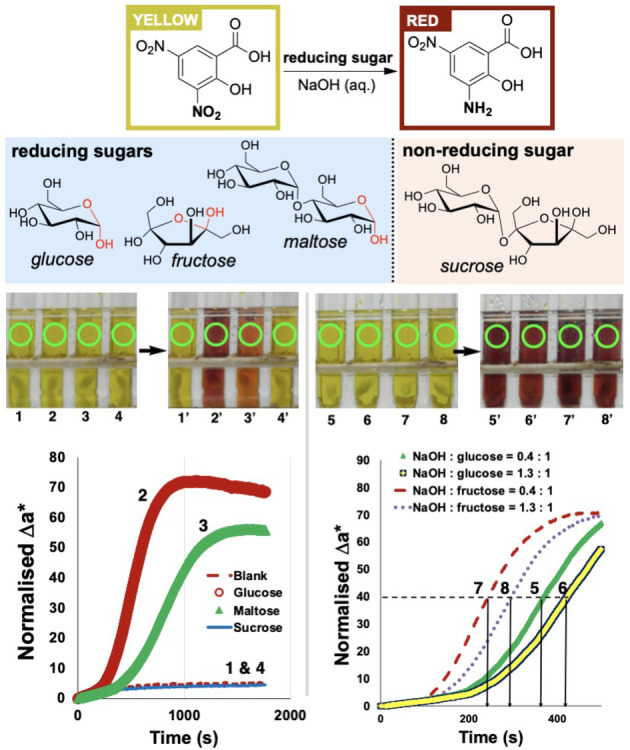
A: A sugar‐mediated regioselective reduction of 3,5‐dinitrosalilcylic acid (DNSA) under basic conditions. B: Exemplar reducing and non‐reducing sugars employed in this study. C: Before and after stills from videos of four parallel reactions to explore DNSA reduction kinetics with different sugars. Green circles represent multiple regions of interest used to capture parallel kinetic analysis from one video file each for processes **1**–**4** and **5**–**8**. D (left): sugar screening. D (right): concentration profiling for selected sugars.

**Sugar‐mediated aromatic nitro reduction**. To further explore the use of our multi‐region computer vision‐enabled parallel reaction kinetics method beyond the well plate environment, we explored the colorimetry of reducing sugars (Figure 13).[^56^, ^57^, ^58^] Upon exposure to an aqueous alkaline solution of a reducing sugar (e.g. glucose, fructose, or maltose) at high temperature, yellow 3,5‐nitro‐salicylic acid (DNSA) turns red as a result of chemoselective reduction of the 3‐nitro group. While this colorimetric reaction is normally employed for a single‐point determination of sugar concentration via UV‐vis spectroscopy following an enzymatic process,^[58]^ we demonstrated that DNSA reduction kinetics could be tracked in parallel, across four test tubes set‐up with different sugars and sugar concentrations (Figure 13). With reference to Figure 1, and the characteristic red colour of the amine produced from DNSA reduction, it was possible to use the isolated a* dimension from the CIE–L*a*b* time series recorded. The more positive the value of a*, the more red is the sample being record on video.

It was not our aim to fully investigate the mechanistic consequences of these observations in sugar‐mediated reduction chemistry. However, given the long‐suspected intricacy of the mechanism(s) involved,[^59^, ^60^, ^61^, ^62^] our experiments nonetheless revealed intriguing trends at a high level of temporal resolution (25 frames per second; 1 data point every 0.04 seconds). First, this parallel reaction monitoring approach captured data consistent with glucose (a monosaccharide) reducing DNSA faster and with a more intense overall ‚reddening’ of the reaction bulk than the maltose (a disaccharide). These comparative and parallel kinetic analyses were performed alongside further experiments to record a control experiment (no sugar present) and true negative test employing the non‐reducing sucrose (a disaccharide, absent of a free hemiacetal functional group that can convert into an open‐chain oxidisable aldehyde).

Similar parallel experiments have also evidenced concentration effects for both glucose and fructose on the effective rate of DNSA reduction. For both these reducing monosaccharides, excess hydroxide rather than excess reducing sugar led to faster reactions (Figure 13 bottom right, **5**>**6**, and **7**>**8**). Perhaps more intriguingly, fructose as the source of the reducing agent produced faster reactions than glucose (Figure 13 bottom right, **7** and **8** both faster than **5** and **6**). Compared to fructose, reactions with glucose appeared to have a longer induction period (>363 s for glucose versus >243 s for fructose) before reaching a maximum rate of colour change, according to the a* metric (the green‐to‐red colours in the CIE‐L*a*b* colour space). These data are consistent with the measured and calculated lower aqueous pK_*a*_ values of fructose relative to glucose, from which we might hypothesise that hydroxide‐mediated ring‐opening to form the active reducing species is likely faster for fructose than glucose.^[63]^ Beyond the biochemical use of colorimetric reactions with DNSA, these data could have implications for the choice of reducing sugar employed in efforts to design sustainable strategies for scalable reduction of nitrocompounds^[59]^ and use of sugars in metal‐catalysed processes.^[64]^ To the best of our knowledge, no other study—using imaging or other analytical method—has provided the same level of data‐rich non‐contact kinetic information on the chemistry of reducing sugars as that evidenced herien.[^59^, ^60^, ^61^, ^62^]

## 3 Conclusions

Through the further development of *Kineticolor*, we investigated how video analysis presents the opportunity to expand the types and volume of data collected through HTE and smaller scale parallel reaction monitoring efforts. We demonstrated how the move from a one‐video‐one‐reaction to a one‐video‐many‐reactions approach through multi‐region segmentation of the video footage provides the ability to generate kinetic insights of both chemical and non‐chemical dynamics staged in parallel. We have also shown that the manner in which reactions are video recorded is an important piece of this parallel kinetic analysis puzzle; this may be especially important for cases of dilute reactions that are at the perceptible limit of detectable colour change. Analytically, the application of plateau analysis, mutual information analysis, and regression, based on the parallel and high throughput imaging data acquired, serve to show the potential applicability of video analysis in providing a valuable data source for machine learning initiatives in HTE.^[65]^

A crucial point to take away from the current findings is the focus here on enabling a fully flexible, post‐processing approach to selecting the number, arrangement, and size of multiple regions of interest to be analysed in any given video. Akin the comparison of ‚tame’ and ‚wicked’ problems, the developments reported here focus on the latter, enabling one to consider the design of a wide range of experimental set‐ups that facilitate non‐contact kinetic analysis with computer vision, even if those set‐ups fall outside the scope of the comfortable regularity of a well‐plate environment. In balance, it is also envisaged that future work in this area could extend the choice of multi‐region algorithms to include automated as well as user‐controlled methods.

Altogether, these findings appreciably expand computer vision for analytical chemistry (CVAC), to bring video analysis up to the same level of adoption and maturity as single image variants. Ongoing work in our laboratories is now focused on expanding the range of chemistries amenable to parallel reaction monitoring enabled by our computer vision‐driven methodology.

## Conflict of Interests

M.R. is leading the commercialisation of *Kineticolor* software.

## Supporting information

As a service to our authors and readers, this journal provides supporting information supplied by the authors. Such materials are peer reviewed and may be re‐organized for online delivery, but are not copy‐edited or typeset. Technical support issues arising from supporting information (other than missing files) should be addressed to the authors.

Supporting Information

Supporting Information

Supporting Information
