# Supplementary material for: Parallel and High Throughput Reaction Monitoring with Computer Vision
Source: Angew Chem Int Ed Engl. 2024 Oct 31;64(1):e202413395. doi: 10.1002/anie.202413395 (PMC11701362; doi:10.1002/anie.202413395)
Supplement: Supplementary file 1 — Supporting Information [file ANIE-64-e202413395-s001.pdf]

Angewandte Chemie
Eine Zeitschrift der Gesellschaft Deutscher Chemiker

Supporting Information

Parallel and High Throughput Reaction Monitoring with Computer Vision

*H. Barrington, T. J. D. McCabe, K. Donnachie, C. Fyfe, A. McFall, M. Gladkikh, J. McGuire, C. Yan, M. Reid**

Supporting Information: Parallel Reaction and High Throughput Monitoring with Computer Vision

Henry Barrington,[a] Timothy J.D. McCabe,[a] Kristin Donnachie,[a] Calum Fyfe,[a] Aaron McFall,[a] Marina Gladkikh,[a] Jake McGuire,[a] Chunhui Yan,[a] Marc Reid*[a]

[a] Department of Pure & Applied Chemistry, University of Strathclyde, Glasgow, UK.
E-mail: marc.reid.100@strath.ac.uk

***NOTE –** in addition to the higher-level details shared in this document, a zipped folder of machine-readable data, ordered according to the Figure and Table numbers in the main text, is uploaded and available as part of the supporting information for this publication. This folder can be downloaded from the figshare repository:*

https://doi.org/10.6084/m9.figshare.26797735.v1

For information on licensing Kineticolor software, please contact the corresponding author and the University of Strathclyde technology transfer office:

marc.reid.100@strath.ac.uk; iprmanager@strath.ac.uk

Table of Contents

1. General Considerations 3
2. Equipment 3
3. Crystal violet hydroxylation 6
   3.1 General reaction procedure 6
   3.2 Exploration of camera position and glare 7
   3.3 Lighting and camera lenses when using metal well plates 11
   3.4 Quantifying mixing inequalities across a well plate 16
4. DMAP-catalysed umbelliferone esterification 19
   4.2 Mutual information analysis 22
   4.2 High throughput esterification set-up 27
   4.3 HPLC data 29
   4.4 NMR data 31
5. Monitoring palladium black formation 33
   5.1 Parallel reaction set-up 33
   5.2 Computer vision analysis of Pd degradation 34
6. Copper acetate sediment analysis 36
   6.1 Reaction set-up 36
   6.2 Computer vision analysis of sedimentation 37
   6.3 Additional experiments using graded samples from manufacturing effluent 39
7. DNSA reduction with reducing sugars 41
   7.1 Parallel reaction set-up 41
   7.2 UV-vis data 43
8. Kineticolor Analysis 47

1. General Considerations

All reagents were obtained from commercial suppliers and used without further purification, unless otherwise stated.

All HPLC analysis was carried out on an Agilent 1220 Infinity II HPLC instrument (max. pressure 600 bar). All methods and output chromatograms are supplied as part of the machine-readable supporting information zipped folder.

^{1}H spectra were recorded on a Bruker AVIII-400 spectrometer at 400 MHz. Chemical shifts are reported in ppm. Coupling constants are reported in Hz. Spectral printouts are supplied as part of the machine-readable supporting information zipped folder.

UV-vis spectra were collected in plastic 1 cm cuvettes in a Cary 60 spectrophotometer.

2. Equipment

All items listed below were required for the apparatus set-up used for video recording all spot tests reported in this study:

Lightbox (GODOX LST40 LED Mini Photography Studio Tent 40 cm^3)
https://amzn.eu/d/1ijxPCI (accessed 19 August 2024)

- Double LED Light Boards Studio Box for Photography
- 40W Power
- 10000~11000 Lumen
- 5800K±200K Color Temperature

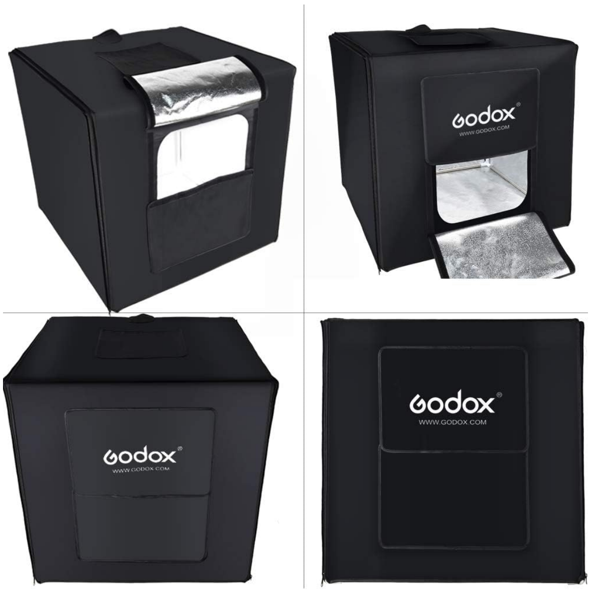

Webcam (Microsoft Q2F-00015 LifeCam Studio Webcam)
https://amzn.eu/d/b2ADwO0 (accessed 19 August 2024)

- Photo sensor technology: CMOS
- Video capture resolution: 1080p

- Maximum focal length: 10 Millimetres
- Maximum aperture: 0.04 Millimetres
- Flash memory type: SDHC, SDXC
- Video capture format: Flash
- Screen size: 2.2 Inches
- Connectivity technology: USB
- Colour: Silver/Black

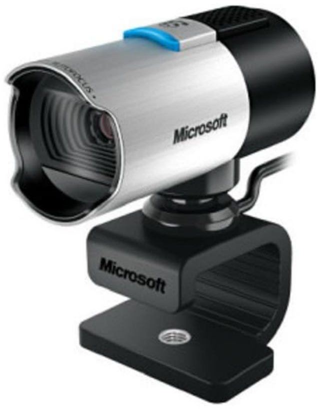

Light Meter (V·Resourcing Handheld Illuminance Meter)
https://amzn.eu/d/gpRZY8S (accessed 19 August 2024)

- **Display:** 4 digital color LCD display
- **Measuring range:** 0~200,000 lux, 0 ~ 20,000 Fc
- **Resolution:** <1000: 0.1; >1000: 1
- **Spectral response:** CIE photopic (CIE human eye response curve)
- **Over range indication:** LCD displays "-OL-" or "-LO-"
- **Spectral accuracy:** CIE V (λ) function (f1’ ≤ 6%, f2’ ≤ 2%)
- **Accuracy:** ±3% rdg ± 8 dgts (<10,000 Lux); ±4% rdg ±10 dgts (>10,000 Lux)
- **Sampling rate:** twice/sec
- **Photo detector:** Silicon photo-diode with spectral response filter
- **Auto power off:** 15mins when not in use

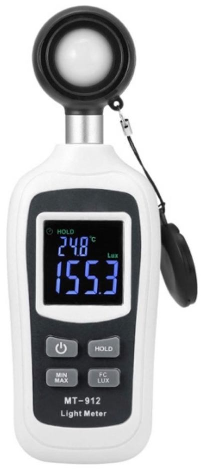

Hotplate Stirrer (Scilogex Circular Top Ceramic Hotplate Stirrer)
https://www.scilogex.com/scilogex-sci280-pro-circular-top-led-digital-hotplate-stirrers.html

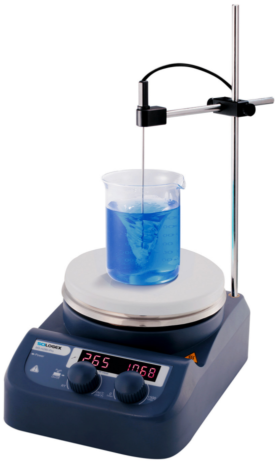

Mass Balance (VWR Analytical balance LA124i)
https://uk.vwr.com/store/product/8016826/vwr-la-classic-analytical-balances-lcd
(accessed 19 August 2024)

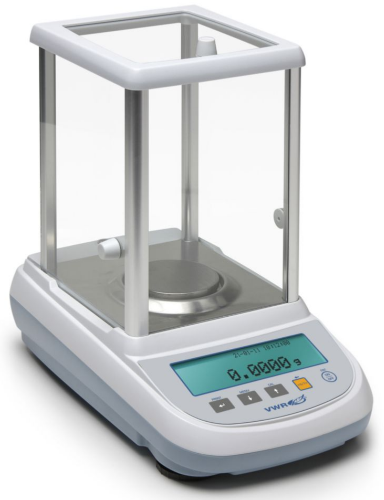

3. Crystal violet hydroxylation

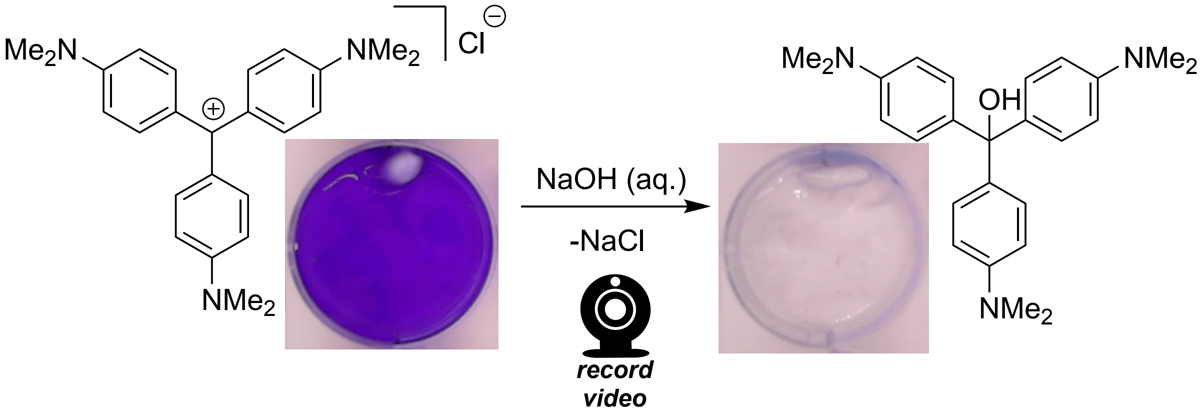

Figure 3 (reproduced). Exemplar trityl cation hydroxylation and its representative colour change.

3.1 General reaction procedure

For all variations of the experiment, crystal violet and sodium hydroxide were prepared to the desired stock concentration in separate 100 mL volumetric flasks, made up to the graduation mark with distilled water.

In all cases, crystal violet was charged into the reaction vessel first, and then the reaction triggered by addition of the target volume of NaOH stock solution. All subsequent kinetic analyses assumed the NaOH addition to represent time zero.

The exact specification of reactant concentrations, volumes, charging method, and reaction vessel are specific on a case-by-case basis below.

3.2 Exploration of camera position and glare

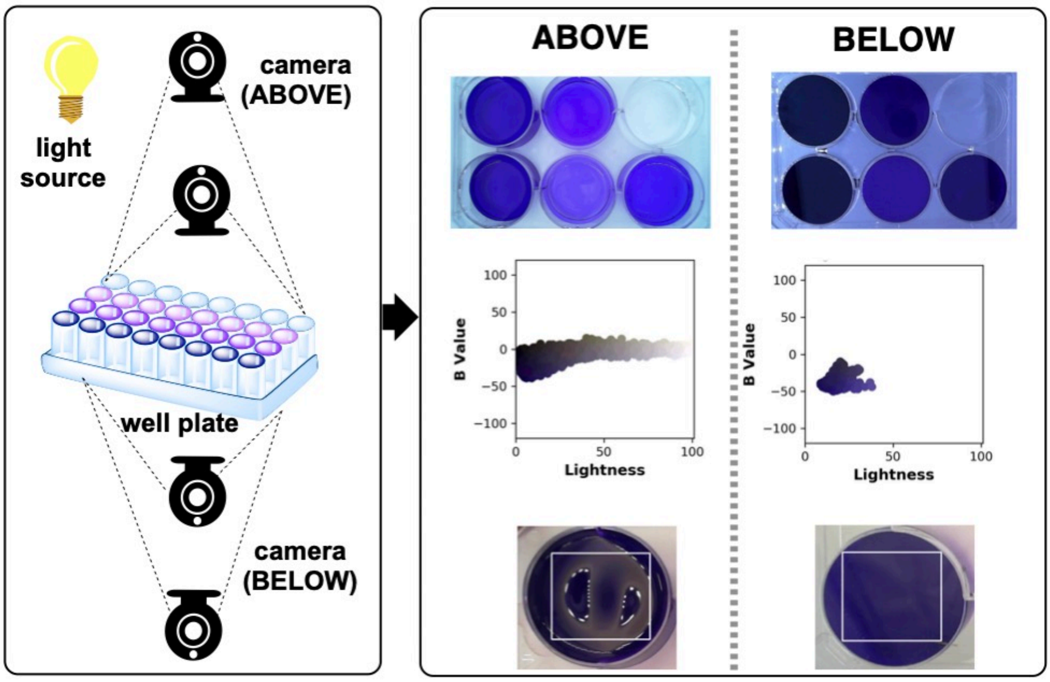

Figure 4 (reproduced). Left: Depiction of camera positions tested relative to fixed well plate and light source positions. Right: Exemplar images of a 6-well plate photographed from above and below. Plots of b* versus Lightness quantify the increased glare when filming from the same side rather than opposite that light source.

For a series of plastic 6- and 24-well plates, wells were charged with various ratios of crystal violet and NaOH stock solutions, as documented in Table S1 below. Web cam video recordings were used as recorded for high throughput kinetic analysis in Kineticolor (described in Section 8).

From the recordings, still images were extracted from the first frame (i.e. beginning of the video). These images were each analysed using Kineticolor's single image analysis suite. As exemplified in Figure 4 (above, from the manuscript), various 2D plots of different combinations of colour channels, from within a single colour space, were plotted. In doing so, these data revealed the spread or concentration of pixel values from within a single well at a single point in time.

Without recourse to more detailed analyses of variance, these plots were used, by visual inspection, to help determine opportunities to minimise glare distorting analysis of colour captured by the webcam employed.

Table S1. Summary of configurations of camera position and distances tested for monitoring crystal violet hydroxylations in high throughput.

<table>
<tr><th>Entry</th><th>Number of wells (plate dimensions, wells by row then column)</th><th>Filming Height from Plate (cm)</th><th>Above or below plate?</th></tr>
<tr><td>1</td><td>6 (2 x 3)</td><td>11</td><td>Above</td></tr>
<tr><td colspan="4">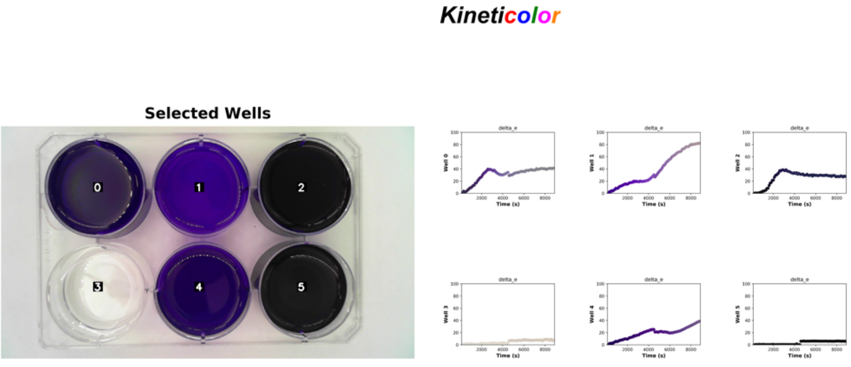
</td></tr>
<tr><td>2</td><td>6 (2 x 3)</td><td>11</td><td>Below</td></tr>
<tr><td colspan="4">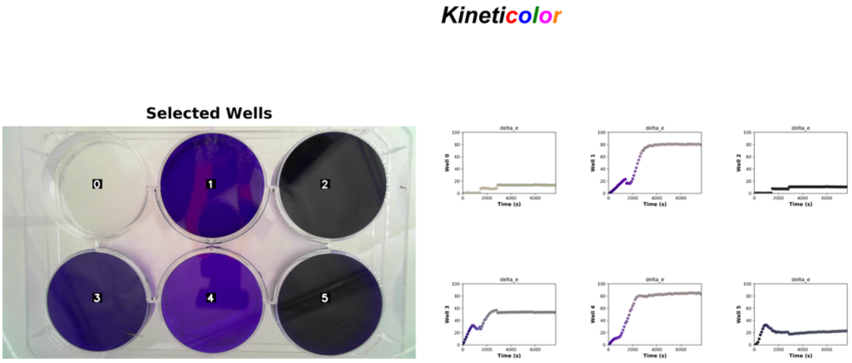
</td></tr>
<tr><td>3</td><td>6 (2 x 3)</td><td>43</td><td>Above</td></tr>
<tr><td colspan="4">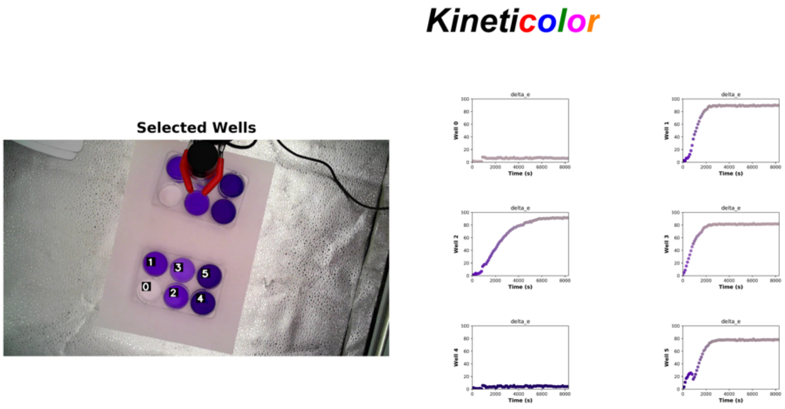
</td></tr>
<tr><td>4</td><td>6 (2 x 3)</td><td>43</td><td>Below</td></tr>
</table>

<table>
<tr><td colspan="4">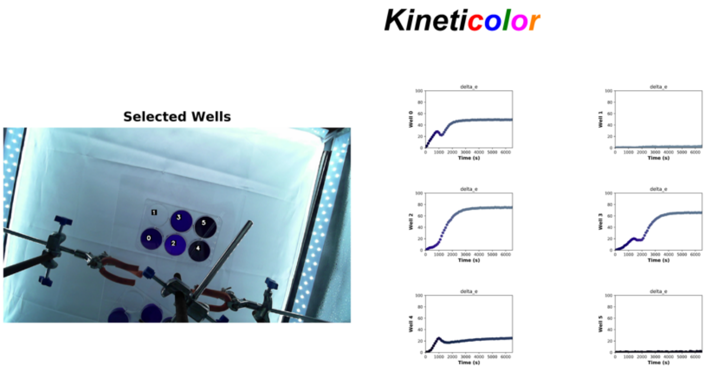
</td></tr>
<tr><td>5</td><td>24 (4 x 6)</td><td>11</td><td>Above</td></tr>
<tr><td colspan="4">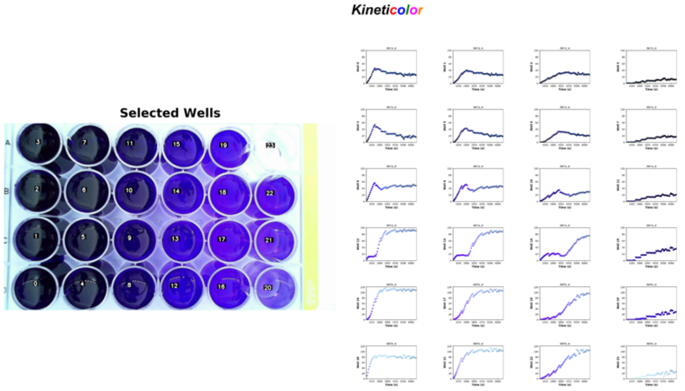
</td></tr>
<tr><td>6</td><td>24 (4 x 6)</td><td>11</td><td>Below</td></tr>
<tr><td colspan="4">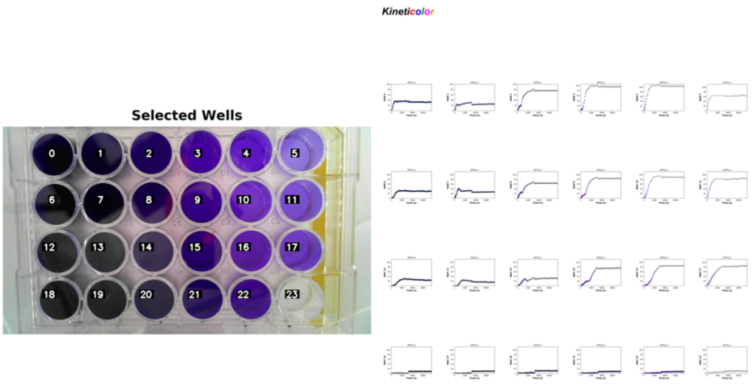
</td></tr>
<tr><td>7</td><td>24 (4 x 6)</td><td>43</td><td>Above</td></tr>
</table>

<table>
<tr><td colspan="4">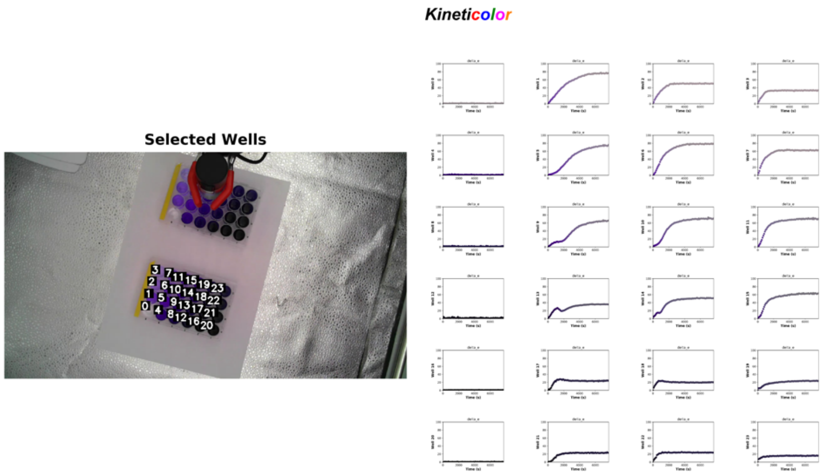
</td></tr>
<tr><td>8</td><td>24 (4 x 6)</td><td>43</td><td>Below</td></tr>
<tr><td colspan="4">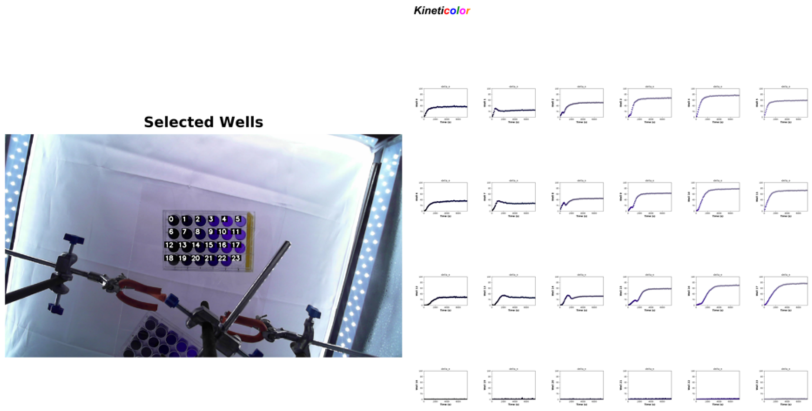
</td></tr>
</table>

Additional graph and machine-readable spreadsheet data are available in the zipped folder made available as part of the downloadable supporting information.

3.3 Lighting and camera lenses when using metal well plates

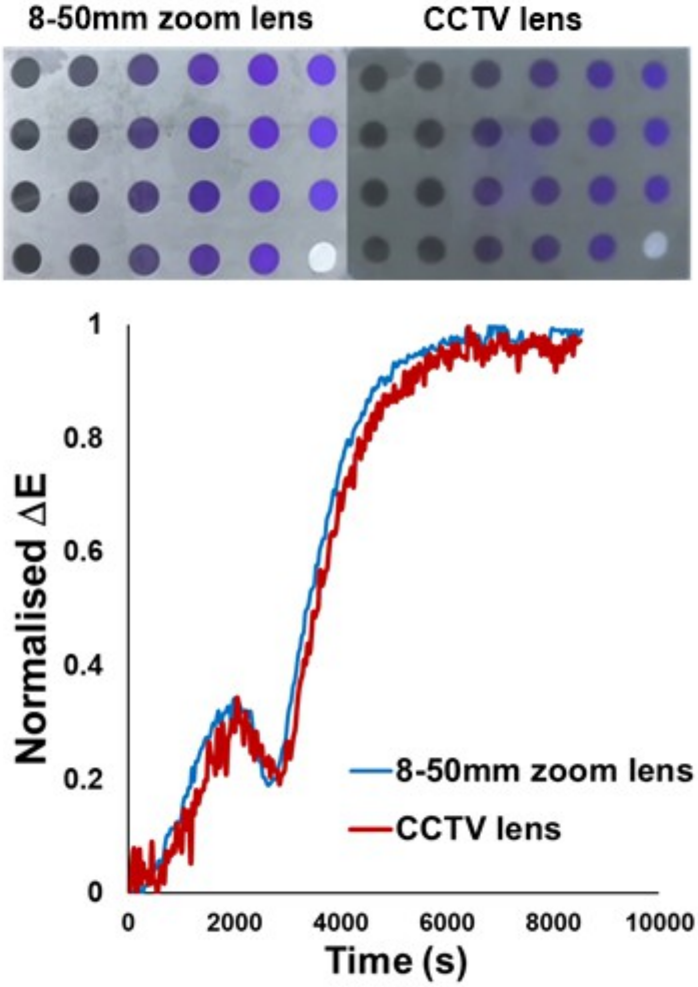

Figure 7 (reproduced). The clearer 8-50mm zoom lens (top-left) produces a smoother ΔE profile when compared to the CCTV lens (top-right), with which the camera was unable to properly focus on the well plate. The CCTV lens produced noisier images, which directly impacted the quality of the ΔE profile.

A 0.039M solution of NaOH was prepared from 155.1 mg and adding to 100 mL volumetric flask and making up to the mark with deionized water. A stock solution of crystal violet using 43.2 mg of crystal violet and deionized water in a 100 mL volumetric flask giving a concentration of 1.06 mM (0.106 mmol). The volumes below where pipetted into glass vials and placed in the order of well positions shown in Table S2.

The lighting used in the Godox lightbox was both LEDs 1 and 2 were turned on full. This can be seen in the set-up photo.

This study involved use of a Raspberry Pi 4, connected to the Raspberry Pi HQ camera, enabling use of detachable lenses for various purposes. The lenses used in this part of our study included:

8-50 mm zoom lens: https://thepihut.com/products/c-mount-8-50mm-zoom-lense-for-raspberry-pi-hq-camera (accessed 19 August 2024)

Tech Specs

- F/No - 1/2.3"
- Aperture - F1.4
- Connector - C-mount
- FoV - 45°-5.35°
- BFL - 17.53mm
- M.O.D - 0.20m
- Dimensions - 40.00×68.30mm
- Aperture Adjustment - Manual
- Weight - 148g

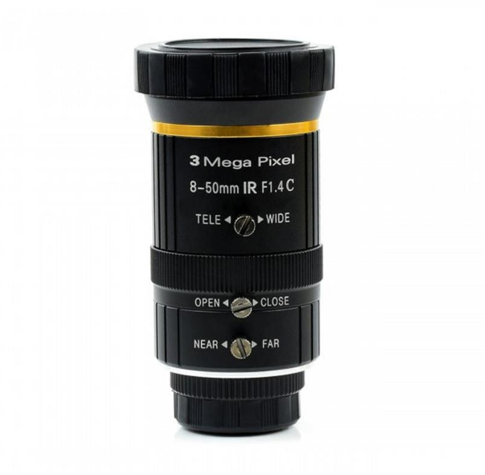

CCTV lens: https://thepihut.com/products/cs-mount-lens-for-raspberry-pi-hq-camera-12mm-focal-length (accessed 19 August 2024)

Tech Specs

- Optical Format: 1/2.3"
- Focal Length: 12mm
- Aperture: F1.6
- Field of View (FOV): 30°(H) *(on Raspberry Pi High Quality Camera)*
- Focus Type: manual
- Mount: CS mount
- Back Focal Length: 7.53mm
- MOD: 0.2m
- Dimension: Φ30×29mm
- Weight: 53g

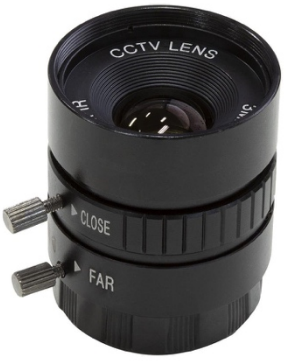

To operate the HQ camera on the Raspberry Pi, the following command was used on the RPi Linux terminal:

```
raspivid --brightness 0.2, --sharpness 5, --awb indoor, --t 9000000, my_file.h264
```

Where “`my_file`” was replaced with file names befitting the context.

Table S2. Summarised volumes in mL of chemicals charged into the plastic 24-well plate. The volumes relate to quantities taken from the CV (0.0016 M) and NaOH (0.039 M) stock solutions.

	A	B	C	D
1	1.5 CV<br>1.5 Water	1.5 CV<br>0.5 NaOH<br>1.0 Water	1.5 CV<br>1.0 NaOH<br>0.5 Water	1.5 CV<br>1.5 NaOH
2	0.8 CV<br>1.8 Water	0.8 CV<br>0.5 NaOH<br>1.7 Water	0.8 CV<br>1.0 NaOH<br>1.2 Water	0.8 CV<br>1.5 NaOH<br>0.7 Water
3	0.4 CV<br>2.1 Water	0.4 CV<br>0.5 NaOH<br>2.1 Water	0.4 CV<br>1.0 NaOH<br>1.6 Water	0.4 CV<br>1.5 NaOH<br>1.1 Water
4	0.2 CV<br>2.4 Water	0.2 CV<br>0.5 NaOH<br>2.3 Water	0.2 CV<br>1.0 NaOH<br>1.8 Water	0.2 CV<br>1.5 NaOH<br>1.3 Water
5	0.1 CV<br>2.7 Water	0.1 CV<br>0.5 NaOH<br>2.4 Water	0.1 CV<br>1.0 NaOH<br>1.9 Water	0.1 CV<br>1.5 NaOH<br>1.4 Water
6	2.5 Water	0.05 CV<br>0.5 NaOH<br>2.45 Water	0.05 CV<br>1.0 NaOH<br>1.95 Water	0.05 CV<br>1.50 NaOH<br>1.45 Water

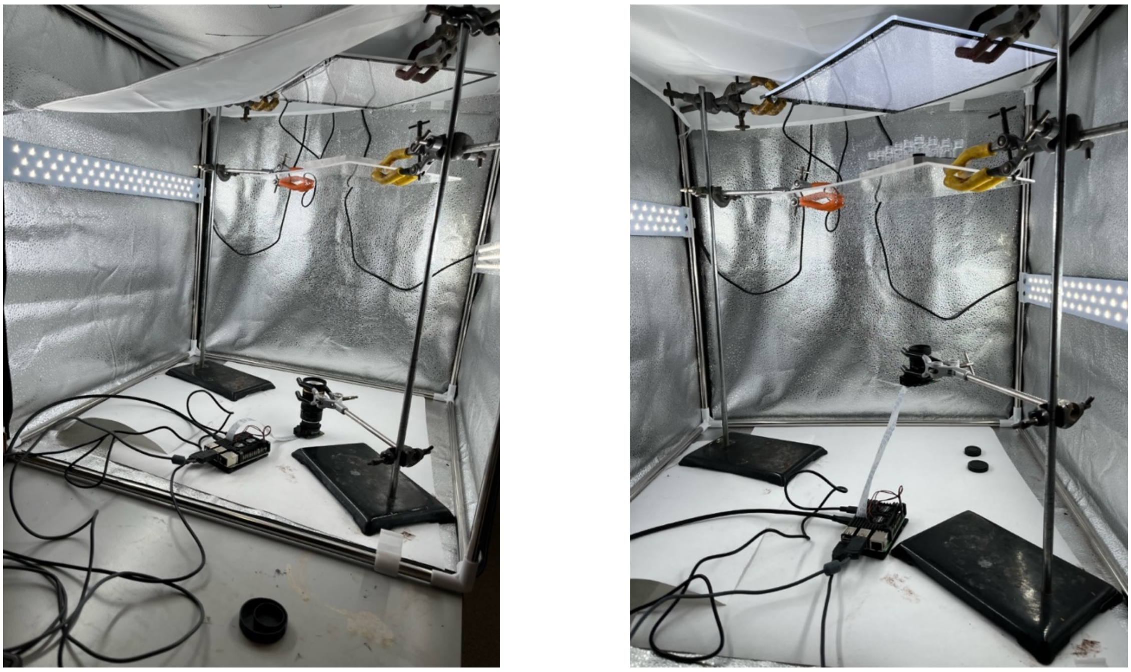

Figure S1. Exemplar photos showing the set-up of the Raspberry Pi, HQ camera module and lens attachments, lighting, and clamp-suspended well plates.

8-50 mm zoom lens

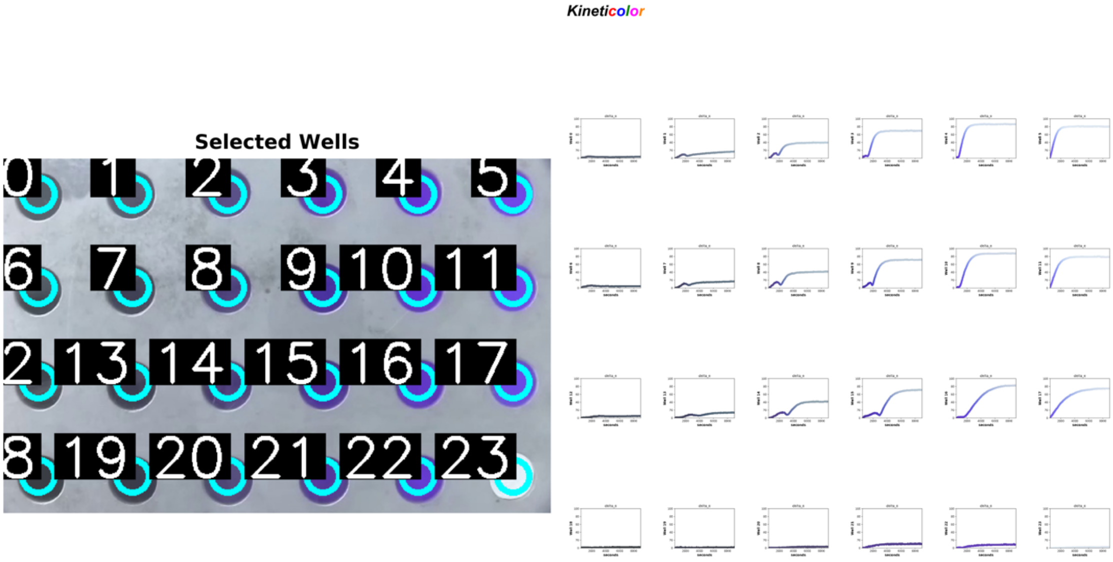

Figure S2. Exemplar Kineticolor high throughput ΔE versus time output for all 24 wells recorded using the zoom lens. All machine-readable data are available in the zipped folder provided.

CCTV lens

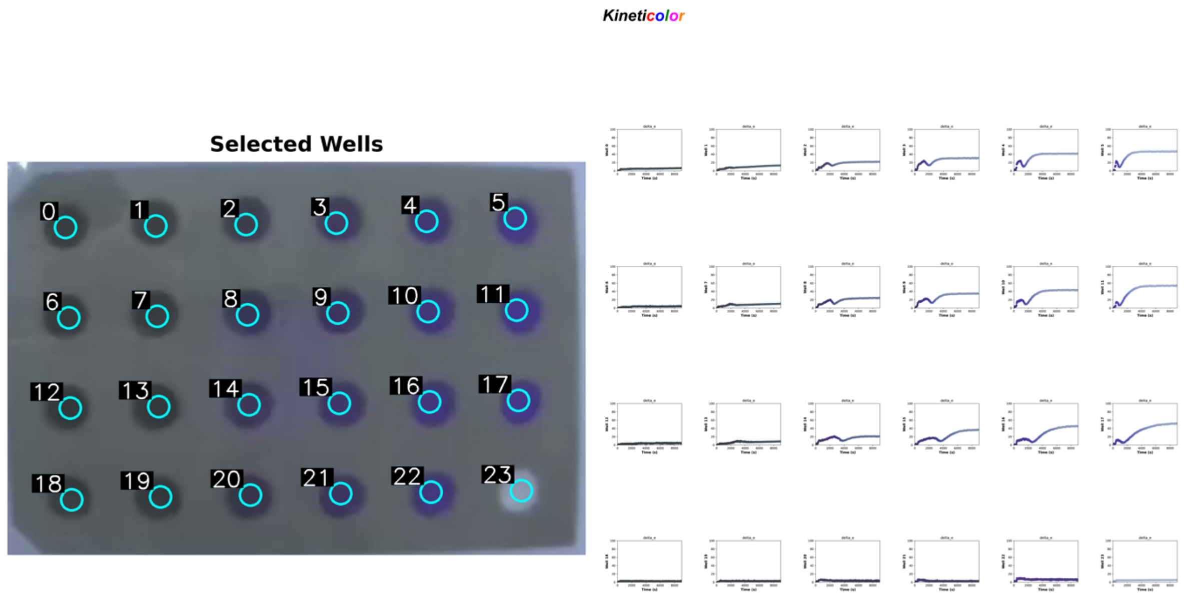

Figure S3. Exemplar Kineticolor high throughput ΔE versus time output for all 24 wells recorded using the CCTV lens. All machine-readable data are available in the zipped folder provided.

3.4 Quantifying mixing inequalities across a well plate

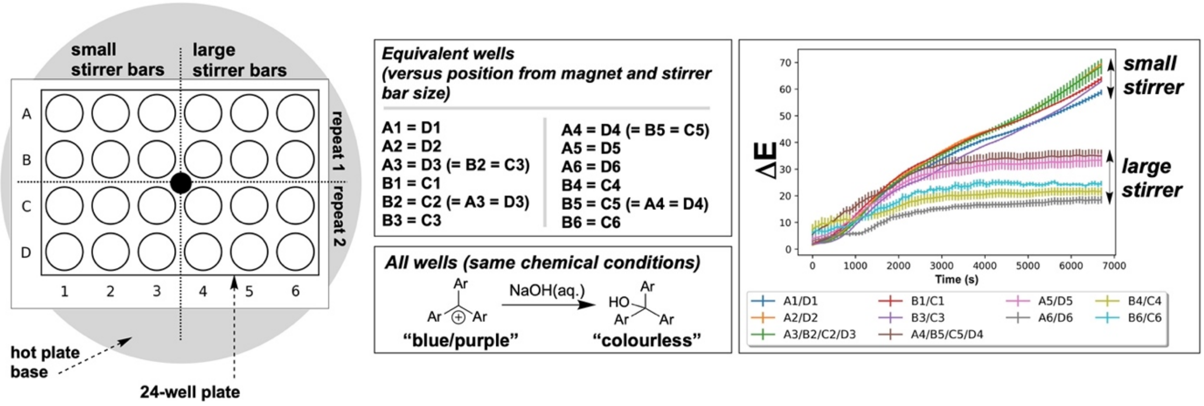

Figure 9 (reproduced). Left: A birds eye schematic view of a 24-well plate, showing the position of the hotplate magnet (black dot). Also visible is the vertical segmentation of the wells into those containing small and large stirrer bars and horizontal segmentation into repetitions of a given experimental condition. Centre: A list of wells deemed equivalent in terms of their likely mixing profile, relative to the stirrer bar and position relative to the central magnet. All wells were charged with identical chemical conditions, running the crystal violet hydroxylation. Right: ΔE profiles from Kineticolor depicting mixing differences from across the well plate. The double-headed arrows are a guide to the eye, showing that there is variation in the kinetics between wells on the plate, despite all wells containing equivalent chemical conditions.

To investigate the application of HTE computer vision kinetics in quantifying mixing and therefore reproducibility across a well plate, the crystal violet hydroxylation, with each well of a plastic 24-well plate charged with the same stoichiometry of reactants.

On the half of the well plate labelled "small stirrer bars", 1.5 x 5 mm shaped stirrer bars were added to each well. For the other half of the plate, labelled "large stirrer bars", 5 x 12 mm sized stirrer bars were added instead.

The quantities of reactants employed is shown in Table S3.

Table S3. Stock solutions of NaOH and crystal violet used for mixing study.

Metric	NaOH	Crystal violet
Stock solution (mL)	100	100
Mass of reagent for stock solution (g)	0.08	0.023
Stock concentration (M)	0.02	0.00057
Target volume of stock per well (mL)	0.25	1.75
Ratio of NaOH : crystal violet (with respect to concentration of each, per well)	5	1

Reservoirs of each stock solution were made by pouring approximately half of the original stock solution into a beaker. Each beaker was then placed inside a bespoke 3D-printed module whose footprint was set to fit in the module positions of the OpenTrons OT-2 liquid handling robot. The wells of the plate were charged using the OT-2.

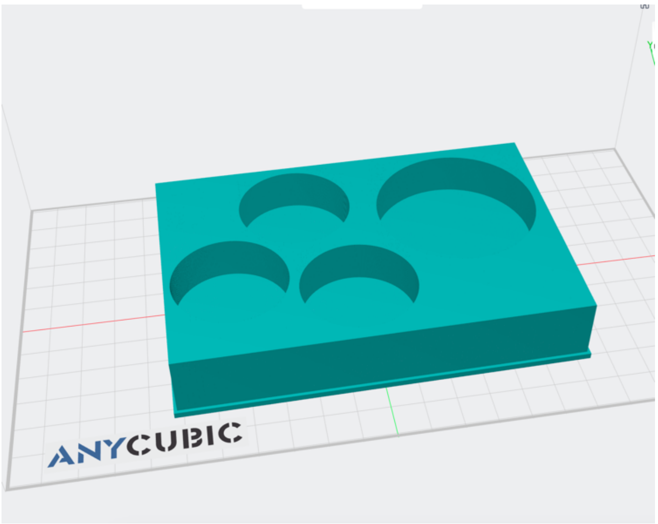

Figure S4. CAD rendering of beaker-holding module, made to fit the OpenTrons OT-2 robot module positions. All CAD and 3D printable files are provided in the machine-readable supporting information zipped folder.

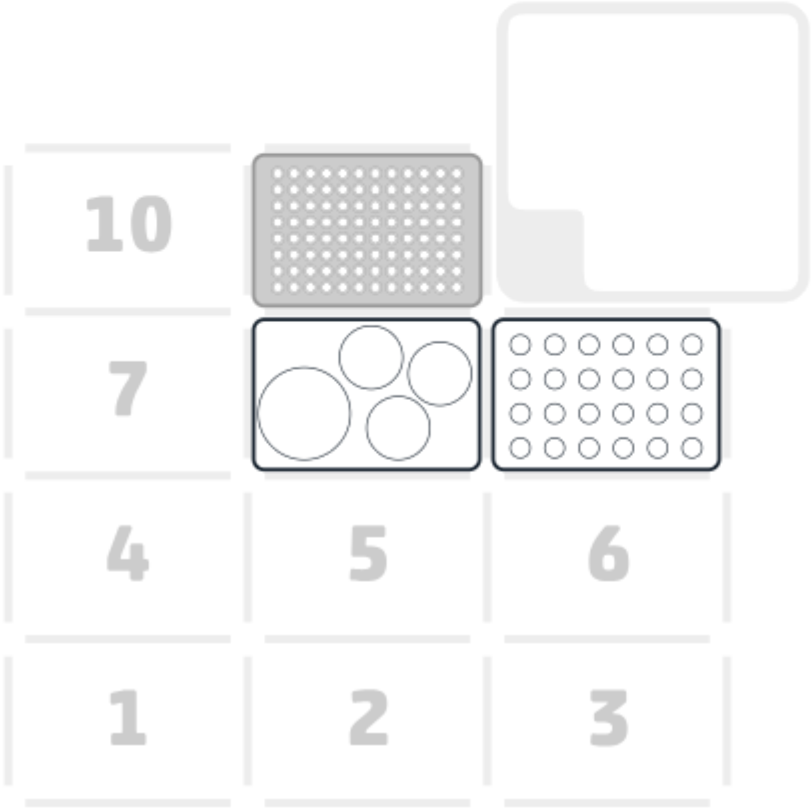

Figure S5. Birds eye view schematic of the OpenTrons OT-2 robot module floor layout. Protocols employed in this paper hosted the pipette tip rack in Module 9, the beaker holder in Module 8, and reaction well plate in Module 7. OT-2 code protocols are available in the zipped folder.

Once all wells were charged, the well plate was transferred to a magnetic stirrer inside a lightbox and filmed using a Panasonic HC-W580 camcorder with the settings shown in Table S4.

Table S4. Camera settings employed for the mixing study.

**Camera model**	Panasonic HC-W580
**Camera control (auto vs manual)**	Manual
**Camera ISO**	default
**Camera zoom**	1x
**Camera aperture**	F6.8
**Camera mount**	Clamp stand, facing down onto the hotplate stirrer; birds eye view of the well plate.
**Camera distance from monitored object(s)**	32 cm approx.
**Lighting hardware**	Godox 45 cm^3 lightbox
**Additional lighting info (e.g. light type)**	See photo for light box LED intensity settings
**White balance**	default
**Shutter speed**	"1/50"

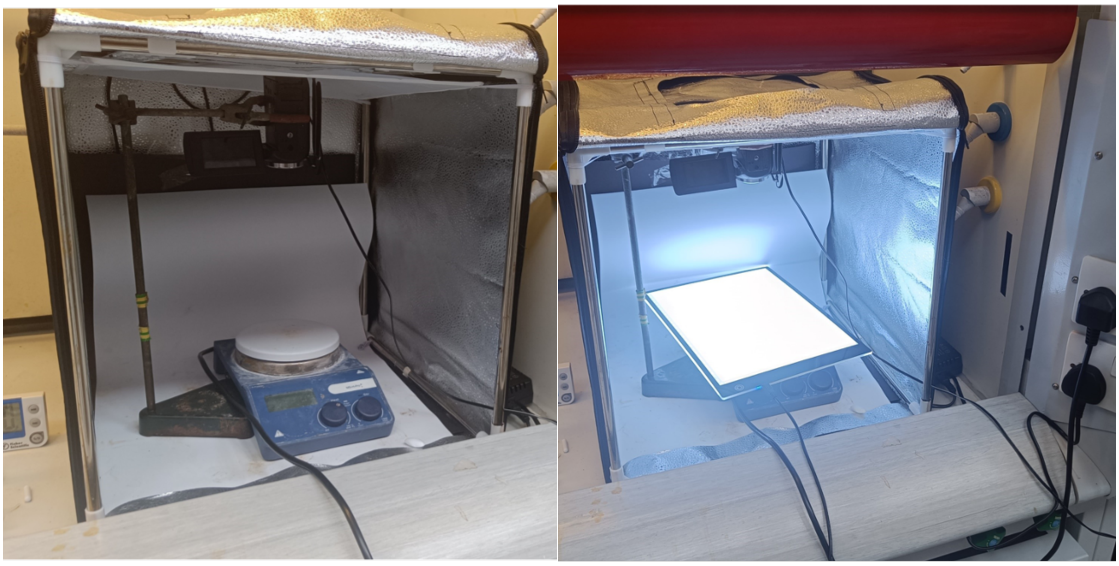

Figure S6. Images of the set-up used to monitor the plastic 24-well plate used for the mixing study.

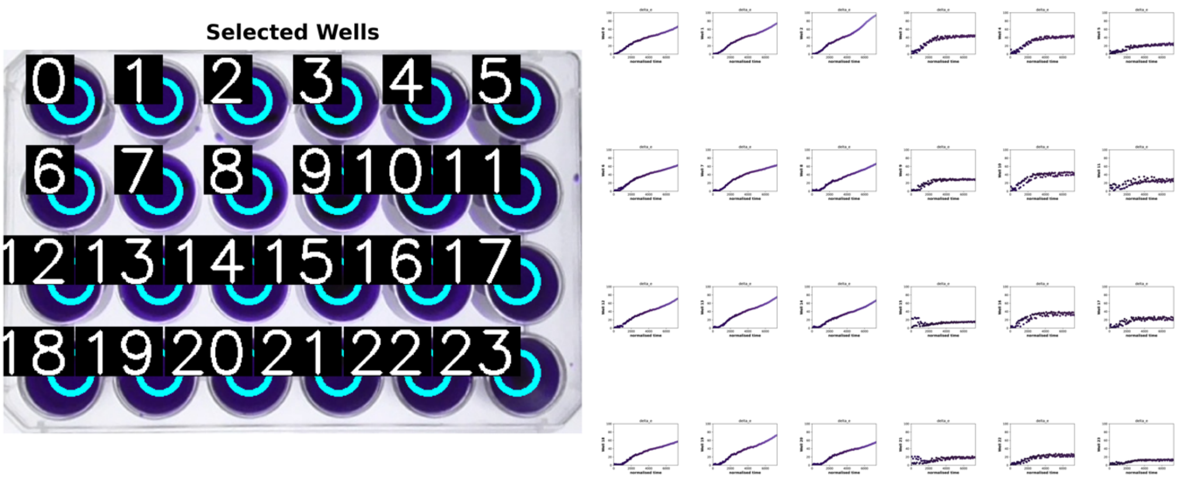

Figure S7. Exemplar Kineticolor high throughput ΔE versus time output for all 24 wells recorded for the mixing study. All machine-readable data are available in the zipped folder provided, including code used to co-plot equivalent wells and error bars shown in each time series in Figure 10 inset.

4. DMAP-catalysed umbelliferone esterification

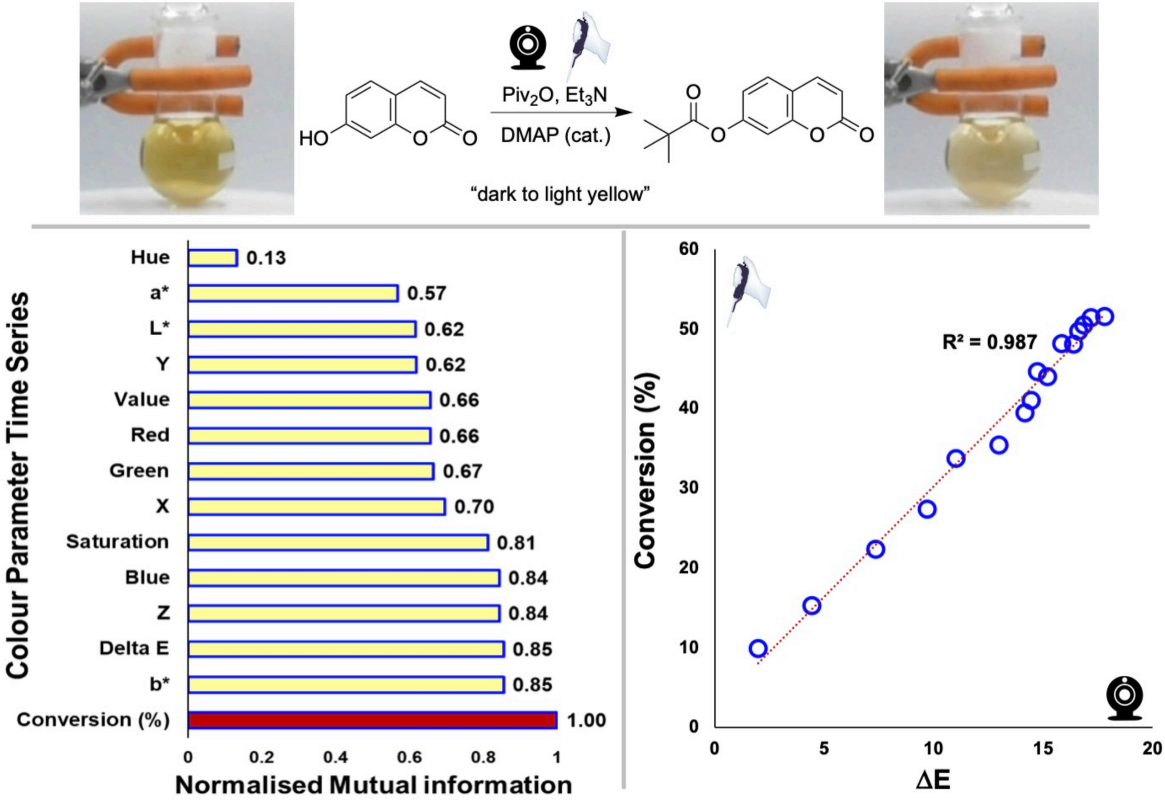

Figure 10 (reproduced). Top: Umbelliferone esterification and images of the bulk reaction colour at 0 and 4000 seconds. Bottom left: ranked and normalised mutual information scores for colorimetric time series based on their likelihood of holding information about product

conversion as measured by HPLC. Bottom right: exemplar linear correlation plot between ΔE and HPLC conversion.

4.1 Batch conditions for esterification to collect data for mutual information analysis

**1**	As a 'blank' HPLC sample, a HPLC vial of 0.75 ml water and 0.75 ml acetonitrile was prepared.
**2**	A 250 mL stock solution of umbelliferone and DMAP in acetonitrile was prepared.
**3**	For the HPLC internal standard, a 50 ml stock solution of acetophenone (0.2 g) in acetonitrile was prepared.
**4**	16 HPLC vials containing 0.75 ml of 1M HCl and 0.25 ml of acetophenone stock solution.
**5**	Vials were then labelled with multiples of 3 up to 30, and then multiples of 5 between 35 and 60 for the remaining vials. These labels corresponded to the minute at which each sample was to be extracted from the esterification reaction mixture.
**6**	A 25 ml round bottom flask (RBF) was positioned above a stirrer hot plate, using clamp stand.
**7**	A stirrer bar (2 cm oval) was added to the RBF.
**8**	From the umbelliferone/DMAP stock solution, 25 mL was pipetted into the RBF. Note the 'full' RBF volume coverage in the above photo.
**9**	The stirring rate on the hot plate stirrer was set to 500 RPM.
**10**	DIPEA base (131 µl, 0.75 mmol) was added to the RBF via pipette.
**11**	Pivalic anhydride (152 µl, 0.75 mmol) was added to the RBF, and a timer was triggered to mark the start of the reaction.
**12**	Every 3 minutes for the first 30 minutes, then every 5 minutes until 60 minutes:
**13**	A 0.5 ml sample from the reaction was transferred via pipette from the reaction mixture to the appropriately time-labelled HPLC vial.
**14**	To investigate reproducibility, steps 4-13 were repeated using a fresh RBF, and the new 16 HPLC vials.
**15**	For video recording, steps 8-12 were then repeated using a fresh RBF.
**16**	A video camera was set-up to observe the RBF. Camera and lighting set-up are described in Table S5 below.
**17**	Video recording was started before the reaction was initiated.
**18**	The target volume of pivalic anhydride (152 µl, 0.75 mmol) was added to the RBF.
**19**	While video recording was in progress, the set-up was left alone to avoid interference in the lighting of the video footage.
**20**	Recording was stopped after 60 minutes of reaction time.

Table S5. Camera settings employed for batch kinetic study on umbelliferone esterification.

**Camera model**	Panasonic HC-W580
**Camera control (auto vs manual)**	Manual
**Camera zoom**	See video for approx. zoom relative to camera position
**Camera aperture**	F6.8
**Camera mount**	tripod

**Camera distance from monitored object(s)**	68 cm approx.
**Lighting hardware**	Godox 45 cm^3 lightbox
**Lighting intensity (lux; by independent brightness measurement)**	760.6
**White balance**	auto wb
**Shutter speed**	"1/50"

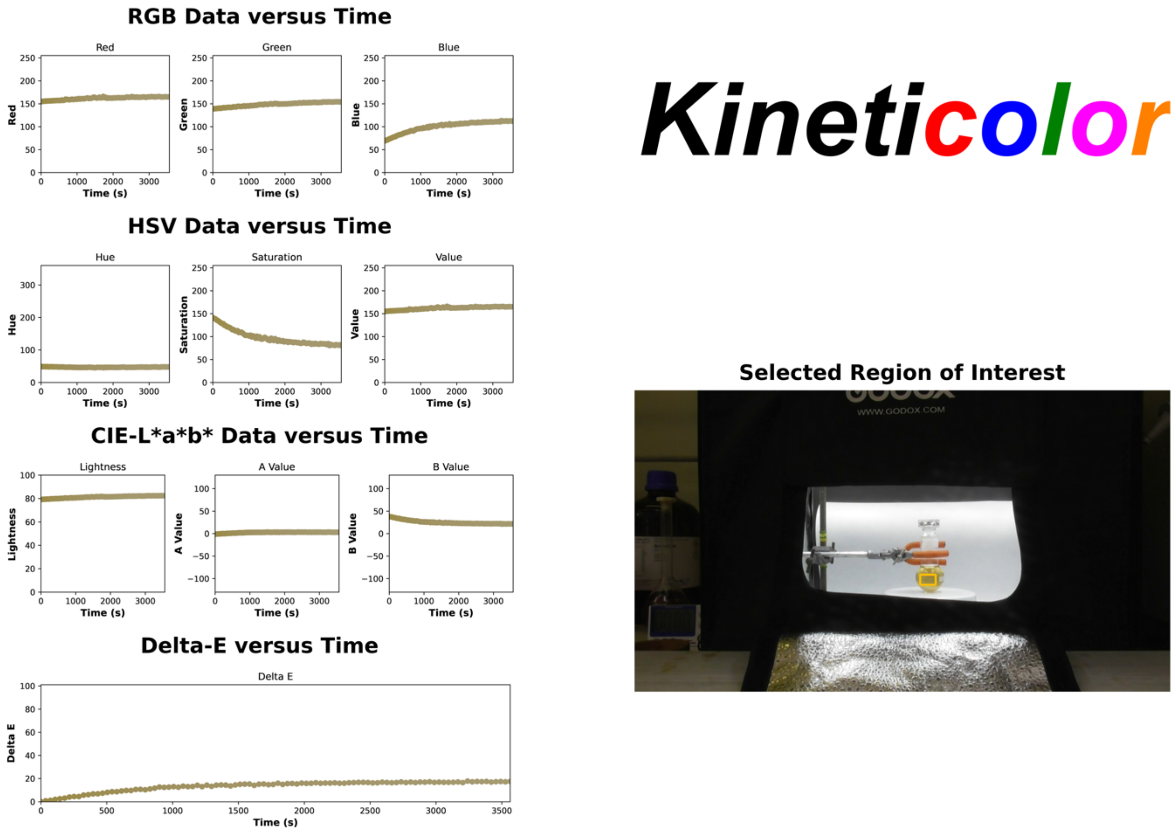

Figure S8. Exemplar Kineticolor high throughput ΔE versus time output for the kinetic analysis

Co-plotted conversion to ester (HPLC) and reaction colour change (ΔE, via Kineticolor) are exemplified in Figure S8 below.

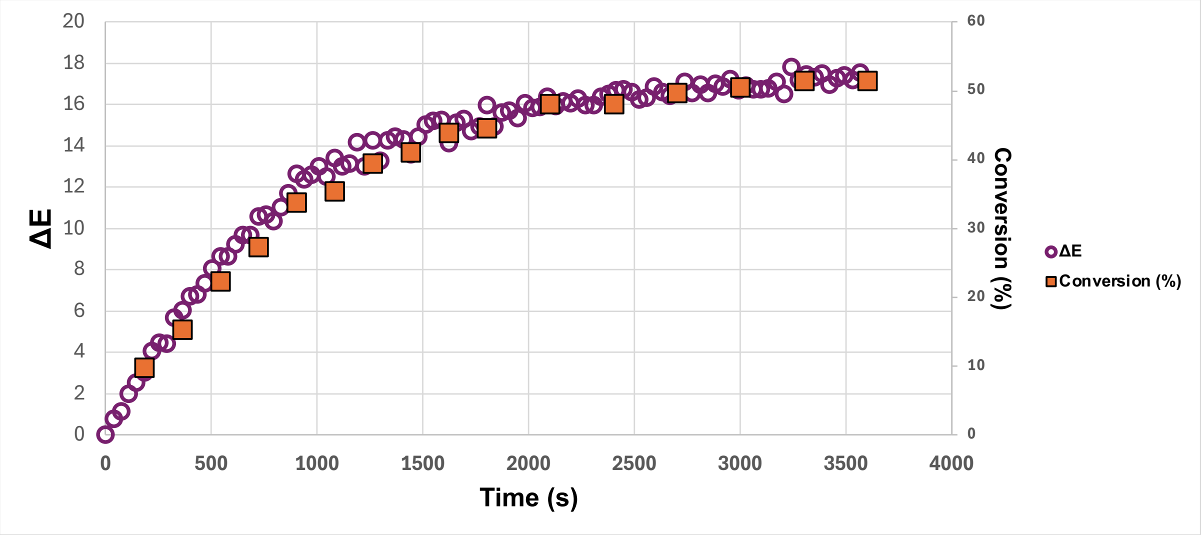

Figure S9. Co-plotted ΔE and HPLC conversion time series for the kinetic study of umbelliferone esterification.

All data are visible in the additional machine-readable supporting information zipped folder available to download on the publication landing page.

4.2 Mutual information analysis

Mutual information analysis was performed as per several previous publications from our team:

- DOI: https://pubs.acs.org/doi/full/10.1021/acs.oprd.2c00216
- DOI: https://doi.org/10.1039/D2SC05702F
- DOI: https://doi.org/10.1039/D3SC01383A

Entropy: A measure of the uncertainty associated with a random variable. The more uncertain we are, the more we lack information.
Information: the capacity to reduce uncertainty.

Quantification of information pioneered by Claude Shannon in *A Mathematical Theory of Communication Bell System Technical Journal, 1948).*
DOI: https://doi.org/10.1002/j.1538-7305.1948.tb01338.x.

For **A = (a_1..a_n)**, this could be (as a relevant example) time series, carrying an array of values occurring at specific times.

Probabilities = (p_1…p_n)
Self-information, I(a_i) = log (1/p_i)

If a data-source has a low-probability value, the event carries more 'surprisal' than when the source data has high probability.

Shannon Entropy (H) defines the amount of uncertainty in a data source as:

$$H = -\sum_{i=1}^{n} p_i . \log{(p_i)}$$

When working with one variable array, x, one must first consider how to calculate or source the value of the **marginal probability**, P(x):

$$H(X) = -\sum_{i=1}^{n} P(x_i) . \log\ \mathrm{P}(x_i)$$

One way to estimate marginal probability is the **histogram approach**:

1. Find **min**, **max**, and **range** of values.
2. Select number and size of **bins**.
3. Segment the **range** using the **number of bins**, each bin being of equivalent size.

The **marginal probabilities** to be summed are then the **frequency counts** falling within each histogram bin divided by the **total number of samples** (i.e. elements in the time series array).

$$P(x_i) = \frac{no. elements\ in\ bin}{total\ no. elements\ in\ series}$$

The **entropy** is then calculated based on the previous equation. If there are 10 histogram bins, the 'i' ranges from 1 – 10.

The process repeats for the number of supposedly independent features to be compared.

After calculating the **entropy** for each feature, one can then look to understand the **information** available in one feature when another feature is given. This is where **joint entropy** comes in.

Joint entropy of discrete random variables X and Y is the entropy of their pairing, **H(X,Y)**. By the same chord, we now need to consider **joint probability, P(x_i,y_i)** instead of **marginal probability**.

$$H(X,Y) = -\sum_{x \in X} \sum_{y \in Y} P(x,y) . log_2[P(x,y)]$$

Useful intuition builder: (X,Y) could represent the position of a **chess piece**. X stores row position, Y stores column position. The **entropy of the row of the piece** and the **entropy of the column of the piece** come together to represent the **entropy of the position of the piece**.

To estimate the **joint probabilities**, we can use 2D instead of 1D histograms. Each 1D histogram composing the 2D histogram can come from two 'independent' features.

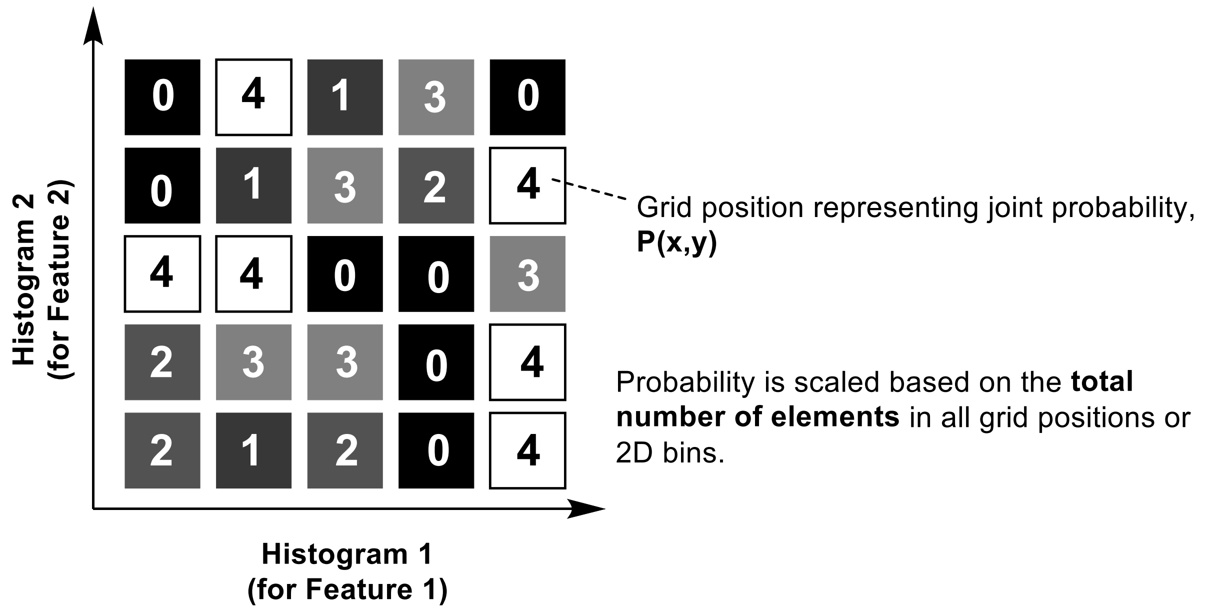

Figure S10. Depiction of joint probabilities used in mutual information analysis.

$$P(x, y) = \frac{no.\, elements\ in\ 2D\ grid\ position}{total\ no.\, elements\ in\ the\ 2D\ grid}$$

Taking all of this towards an understanding of the **mutual information** held between two features or variables demands a **Venn diagram** visualization of the entropies thus far formulated.

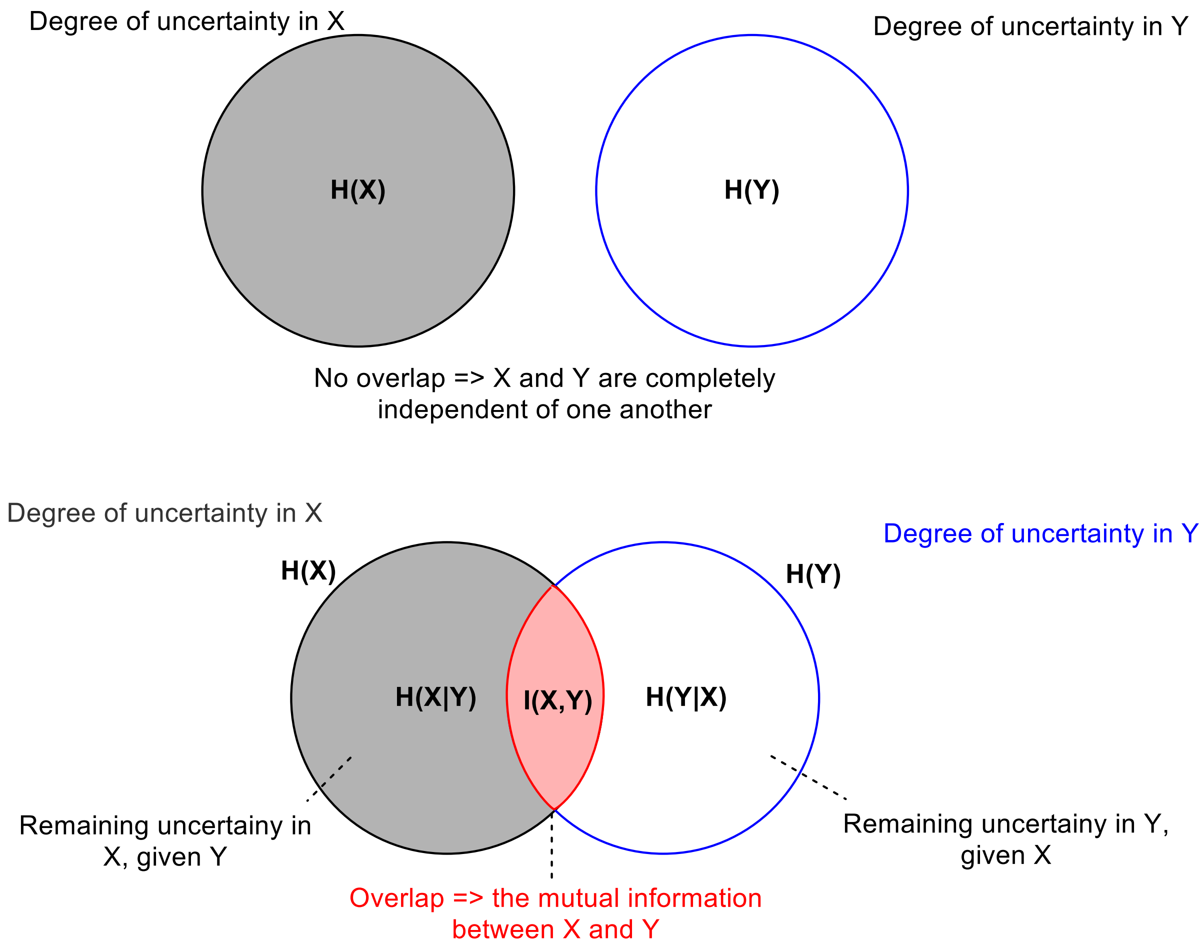

Figure S11. Schematic representation of the mathematical formalism of mutual information.

$$I(X;Y) = H(X) - H(X|Y)$$
$$I(X;Y) = H(Y) - H(Y|X)$$
$$I(X;Y) = H(X) + H(Y) - H(X,Y)$$
$$I(X;Y) = H(X,Y) - H(X|Y) - H(Y|X)$$

where, for example:

$$H(Y|X) = -\sum_{x\in X}\sum_{y\in Y} P(x,y).\,log_2\left[\frac{P(x,y)}{P(x)}\right] = P(x,y).\{log_2[P(x,y)] - log_2[P(x)]\}$$

Some notes on reading these diagrams:

- As the overlap increases, the amount of shared information between X and Y increases. It does **NOT** mean there is any sort of additional transfer of information between X and Y:

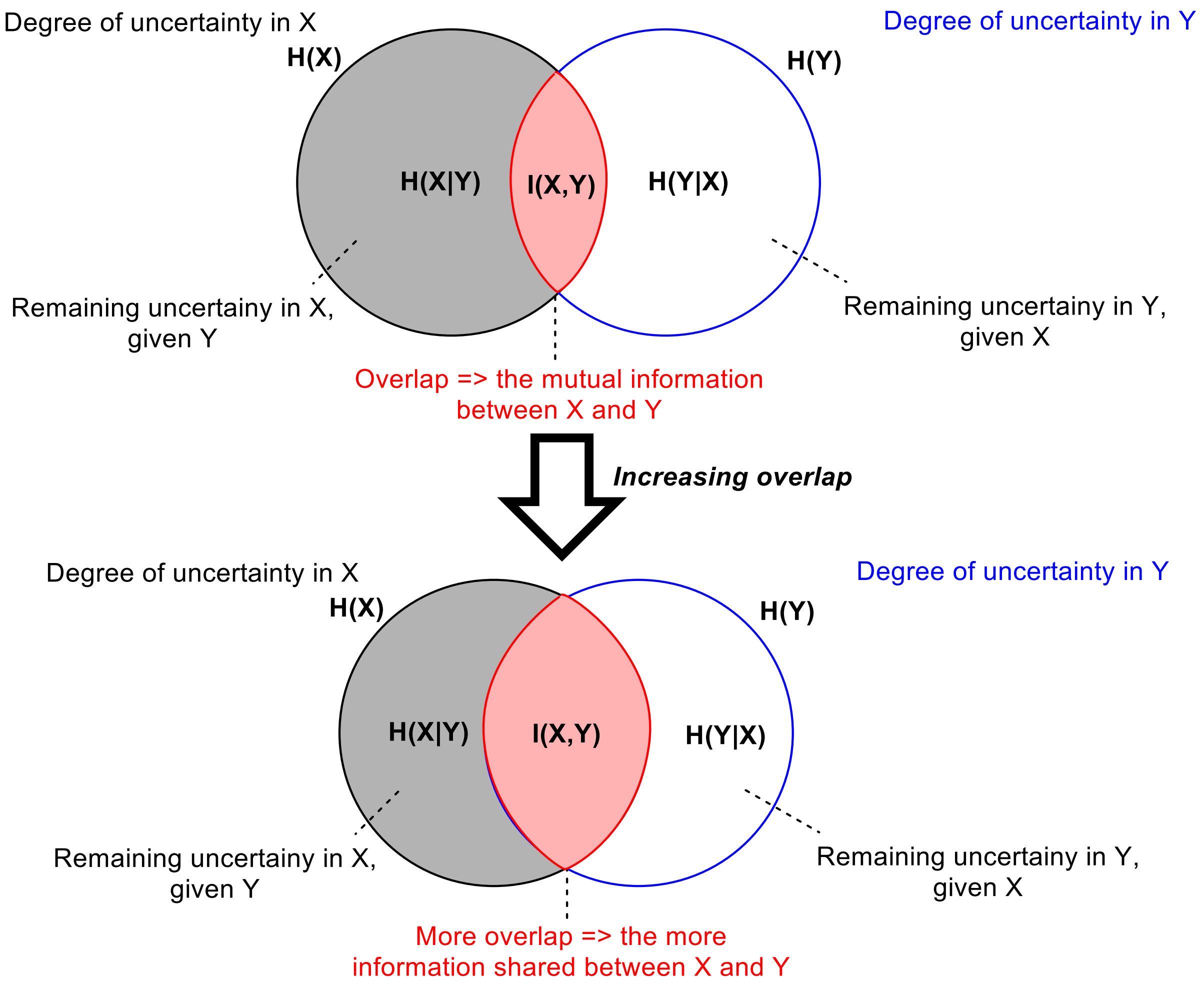

Figure S12. Schematic representation of increased mutual information between two datasets.

- When the **mutual information** is expressed using a *comma* between X and Y, it assumes the size of X and Y is the same**.** It also assumes we are only considering two features, X and Y. For example, X could be an array of HPLC samples over time. Y could be the subset of colour data at the same time points as HPLC samples, making arrays X and Y the same size.
- The use of a semi-colon is more general (e.g. for **conditional mutual information** involving >2 features). The semi-colon denotes everything that is to be measured (left) given everything known (right).

Collected spreadsheet inputs, scripts, and outputs for the analysis are provided in a zipped folder as part of the supporting information collection.

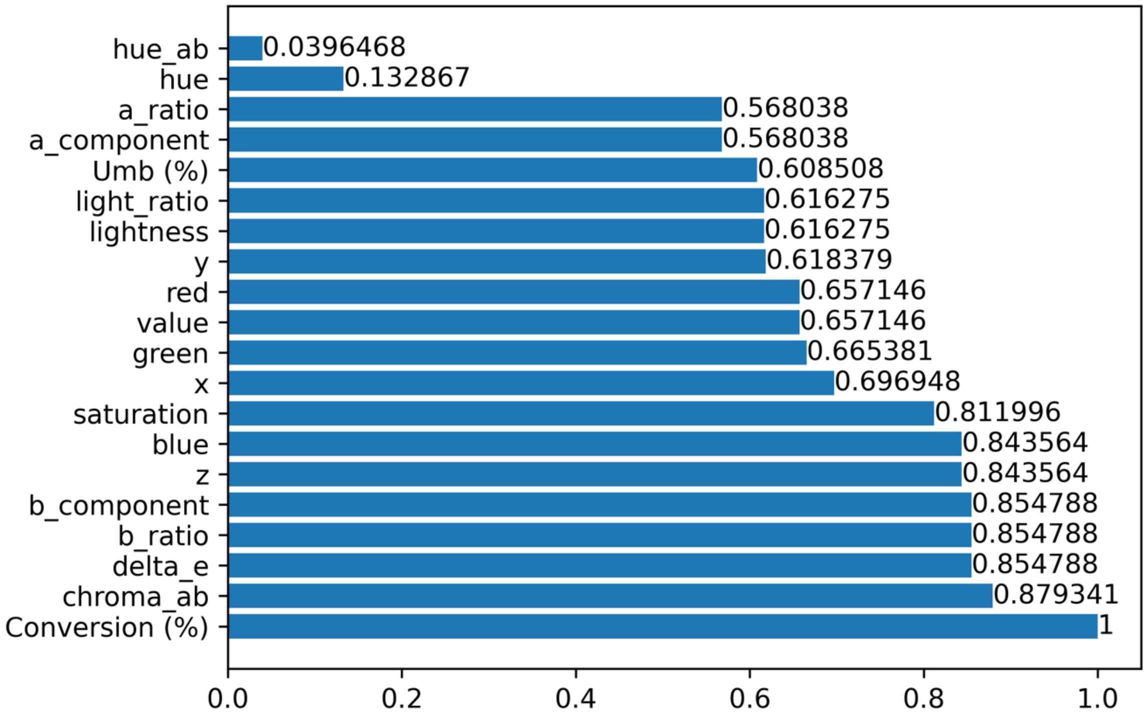

Figure S13. Normalised mutual information scores ranking the colour parameter time series from Kineticolor against the independent HPLC product conversion time series. The value of 1 against Conversion (%) is set to signify the maximum possible score for any of the colour metrics relative that Conversion (%). The higher the score, the more information about conversion we can say is contained in the colour time series.

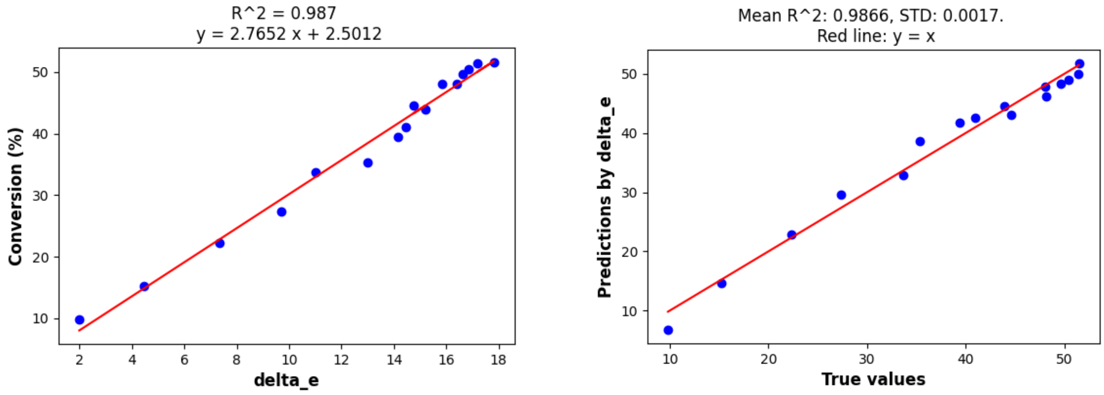

Figure S14. Exemplar regression (left) and leave-one-out cross-validated prediction outputs (right) from analysis of HPLC conversion vs colour parameters for those colour parameters (including ΔE, shown above) that produced high mutual information scores.

4.2 High throughput esterification set-up

Stock solutions of DMAP, pivalic anhydride, and umbelliferone+DIPEA, using acetonitrile as solvent, were prepared in the quantities expressed in Table S6 below.

Table S6. Stock solution compositions for esterification HTE study.

Reagent	Umbelliferone (U)	DIPEA	Pivalic Anhydride (PA)	DMAP
	Combined in one stock solution			
Molecular Weight (g/mol)	162.14	129.24	186.25	122.17
Target Stock Concentration (mol/L)	0.015	0.018	0.4	0.04
Target Stock Volume (ml)	100	100	25	25
Stock no. moles (mol)	0.0015	0.0018	0.01	0.001
Reagent Mass for stock (g)	0.243	0.233	1.863	0.122
Reagent Volume for stock (ml)	N/A	0.314	2.029	N/A

About 50% of each stock solution was charged into a separate beaker. Each beaker was then placed inside the bespoke 3D printout made for placement in the OT-2 module position, as described above in Section 3.4.

Across a plastic 24-well plate, two repeats of the following twelve conditions were programmed for delivery via the OT-2 pipette, as described in Table S7.

Table S7. The 12 reaction conditions used across the 24-well plate for high throughput reaction monitoring in the esterification study.

	Equivalents			Volumes (mL)			
**Conditions**	**U + DIPEA**	**DMAP**	**PA**	**U + DIPEA**	**DMAP**	**PA**	**Solvent**
**1**	1	0.05	0.5	2.475	0.046	0.046	0.233
**2**	1	0.05	1	2.475	0.046	0.093	0.186
**3**	1	0.05	1.5	2.475	0.046	0.139	0.140
**4**	1	0.05	2	2.475	0.046	0.186	0.093
**5**	1	0.1	0.5	2.475	0.093	0.046	0.186
**6**	1	0.1	1	2.475	0.093	0.093	0.139
**7**	1	0.1	1.5	2.475	0.093	0.139	0.093
**8**	1	0.1	2	2.475	0.093	0.186	0.046
**9**	1	0.15	0.5	2.475	0.139	0.046	0.140
**10**	1	0.15	1	2.475	0.139	0.093	0.093
**11**	1	0.15	1.5	2.475	0.139	0.139	0.047
**12**	1	0.15	2	2.475	0.139	0.186	0.000

The arrangement of modules on the OT-2 robot was the same as that depicted as Figure S5.

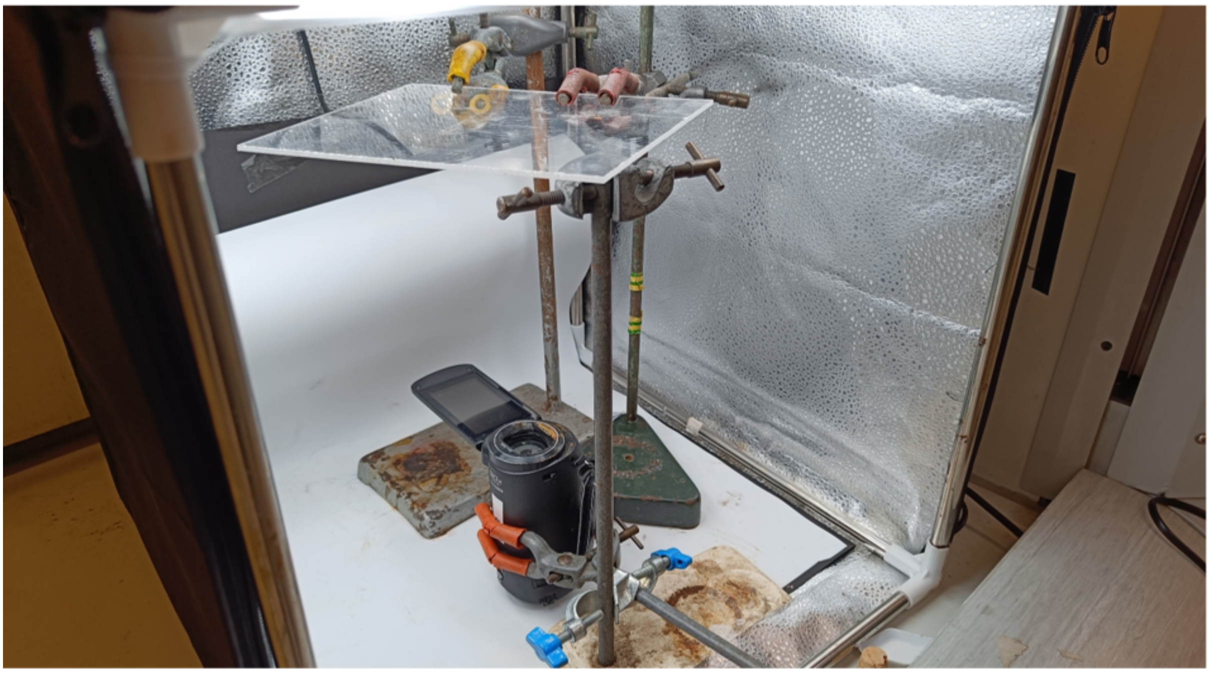

Figure S14. A photo of the set-up used for high throughput reaction monitoring and kinetics in the esterification case study. The camcorder is pointing up at the Perspex sheet on which the 24-well plate sits, and all inside the light box.

Table S8. Camera settings employed for high throughput kinetic study on umbelliferone esterification.

**Camera model**	Panasonic HC-V380
**Camera control (auto vs manual)**	Manual
**Camera zoom**	1x
**Camera aperture**	F6.8
**Camera mount**	Clamp stand
**Camera distance from monitored object(s)**	30 cm approx.
**Lighting hardware**	Godox 45 cm3 lightbox
**White balance**	default

4.3 HPLC data

Beyond summaries in Figures S15 and S16, HPLC methods and instrument chromatogram outputs are available in the supporting information zipped folder.

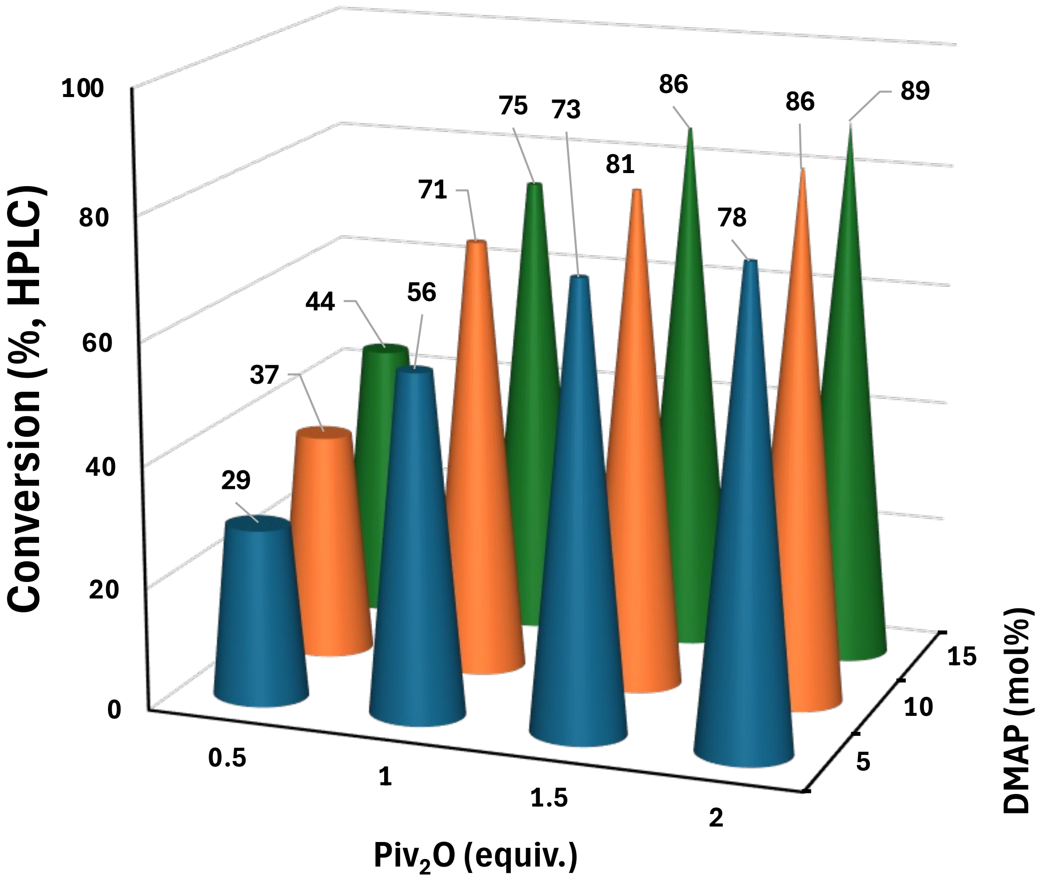

Figure S15. Summarised results of umbelliferone esterification via HPLC. Each result is the average of two runs (i.e. two positions on the well plate charged with the same reactant stoichiometry).

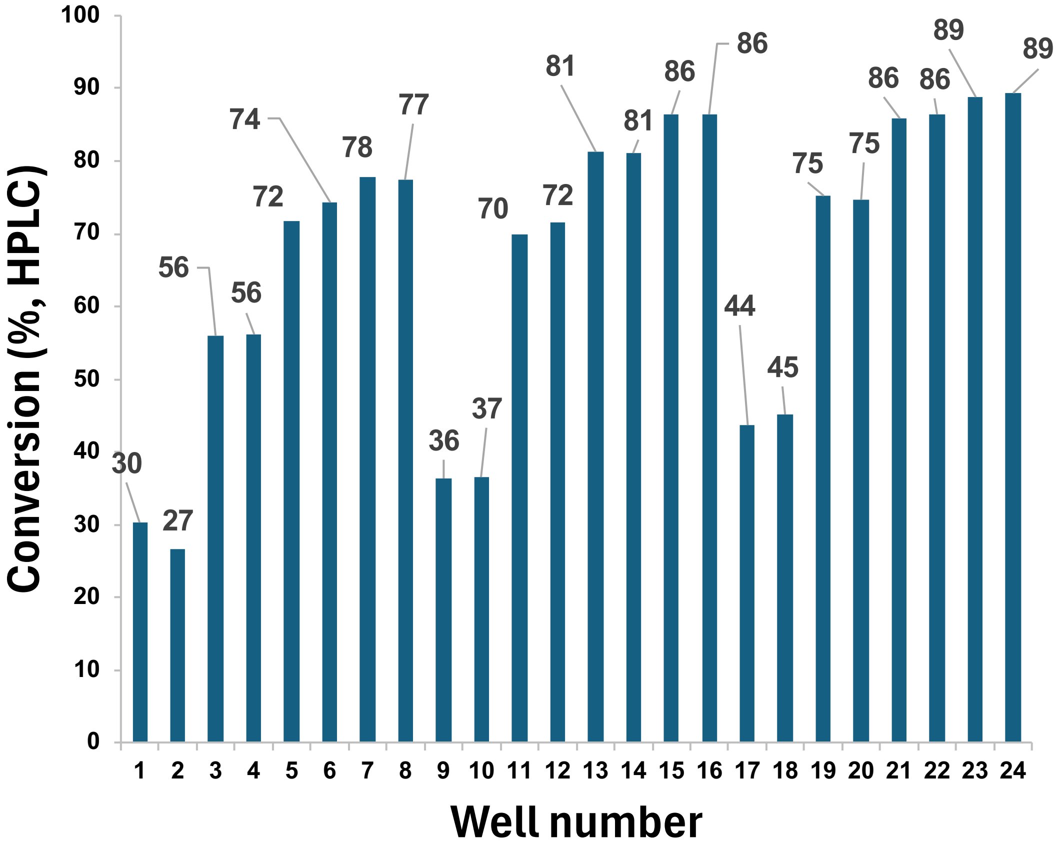

Figure S16. Summarised results of umbelliferone esterification via HPLC, without averaging. Wells 1+2 relate to Condition 1 in Table S7, wells 3+4 relate to Condition 2, and so on.

4.4 NMR data

For additional verification of the evidenced relationships between reaction colour and independent conversion measurements, the reaction product was further characterised by ^{1}H {^{13}C} NMR in $CDCl_3$. These data match reported values for the same ester reported in the literature (ref. 45 in the manuscript).

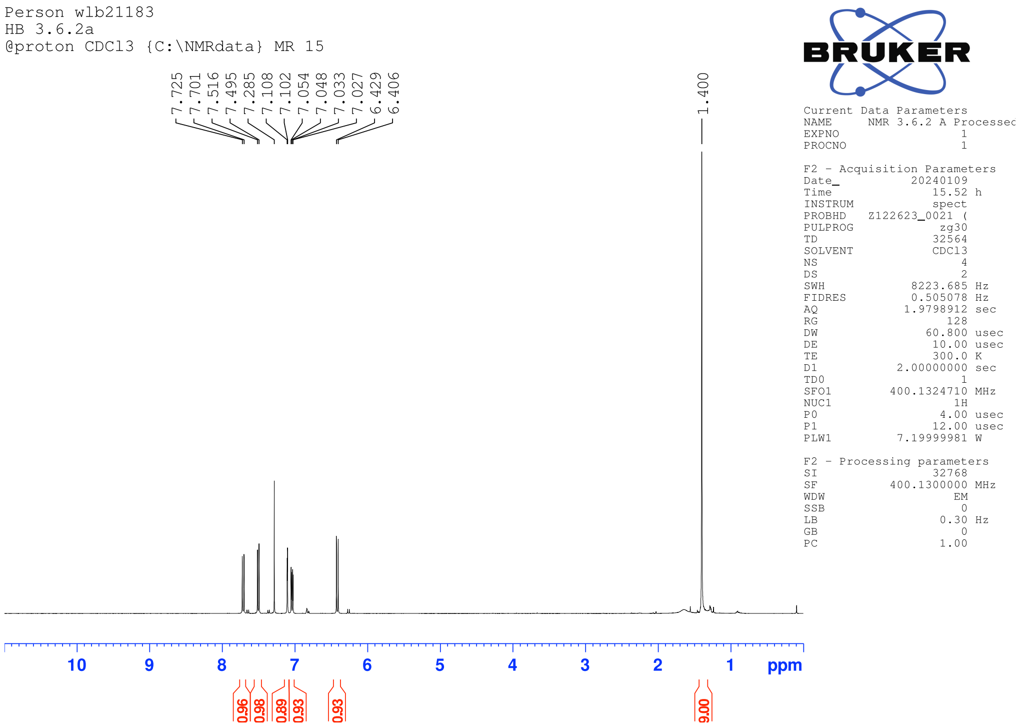

Figure S17. ^{1}H NMR of umbelliferone pivalate ester product.

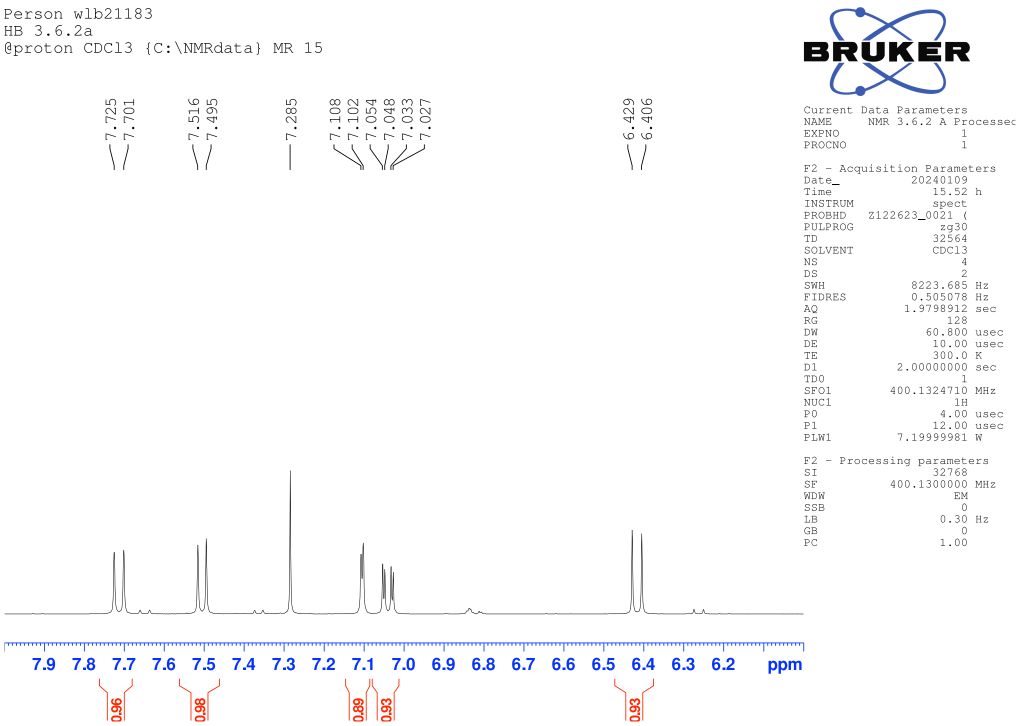

Figure S18. ^{1}H NMR of umbelliferone pivalate ester product, focused on the aromatic region.

5. Monitoring palladium black formation

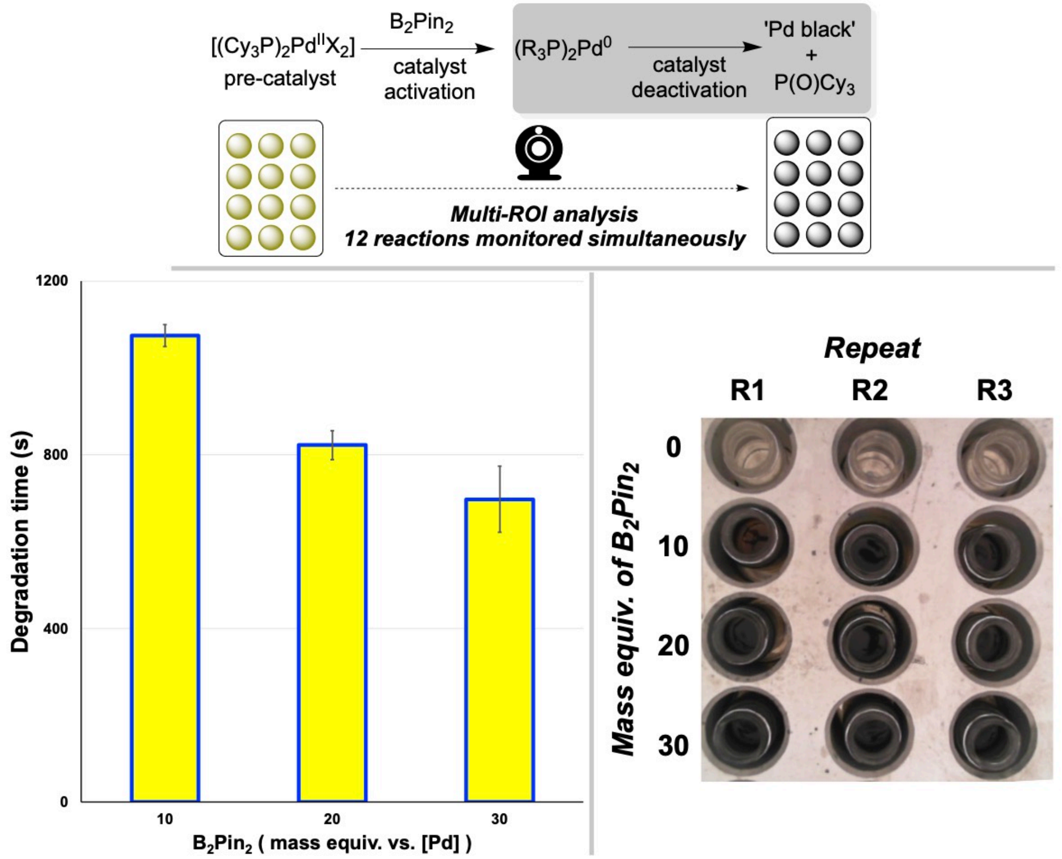

Figure 11 (reproduced). Parallel kinetic analysis of Pd(II) precatalyst degradation to form Pd black. Bottom left: Degradation time decreases with increasing [B_2Pin_2].

5.1 Parallel reaction set-up

Twelve 4-dram vials were charged with a stirrer bar (2x5 mm), dry toluene (4 mL), [$(Cy_3P)_2Pd(OAc)_2$] (3.7 mg, 0.005 mmol) and B_2Pin_2 (added in the proportions. The latter two were added in the mass ratios shared in Table S9 below.

The vials were set inside individual wells of a STEM Corporation RS5000A heater/shaker high throughput reaction station, inside a Godox 45 cm^3 light box. Stirring on the station was set to 400 RPM and the temperature to 70 ºC. A Microsoft LifeCam Studio Q2F-00016 HD Webcam was mounted approximately 30 cm above the reaction station to give a birds eye view of the open vials.

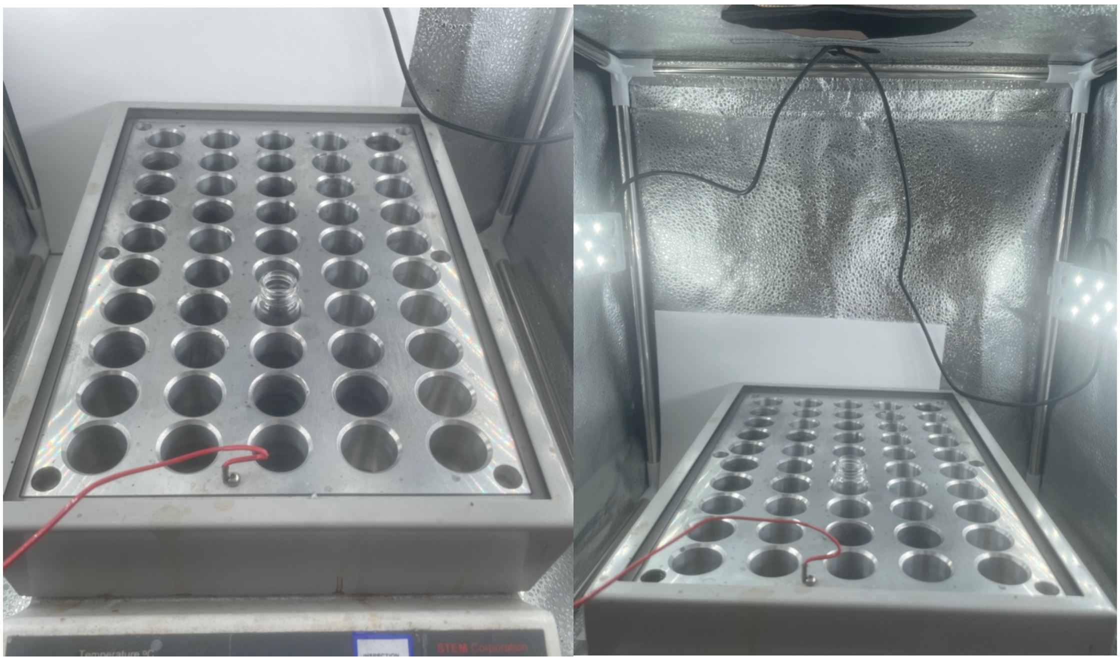

Figure S19. Key components of the reaction set-up for high throughput reaction monitoring of Pd complex degradation and Pd-black formation.

Table S9. Summarised quantities of B_2Pin_2 (mg) charged to each 4-dram vial on the high throughput station.

	**A (Repeat 1)**	**B (Repeat 2)**	**C (Repeat 3)**
**1**	Blank (toluene only)	Blank (toluene only)	Blank (toluene only)
**2**	39	39	39
**3**	78	78	78
**4**	117	117	117

5.2 Computer vision analysis of Pd degradation

From Kineticolor's high throughput multi-region video analysis output, plots of ΔE vs time generated profiles with sharp inflection points in all cases were Pd black formation was observed. Comparative degradation times were taken as the point of inflection on each plot.

These data were qualitatively consistent with reactivity reported in our previous publication:

- DOI: https://doi.org/10.1039/D2SC05702F

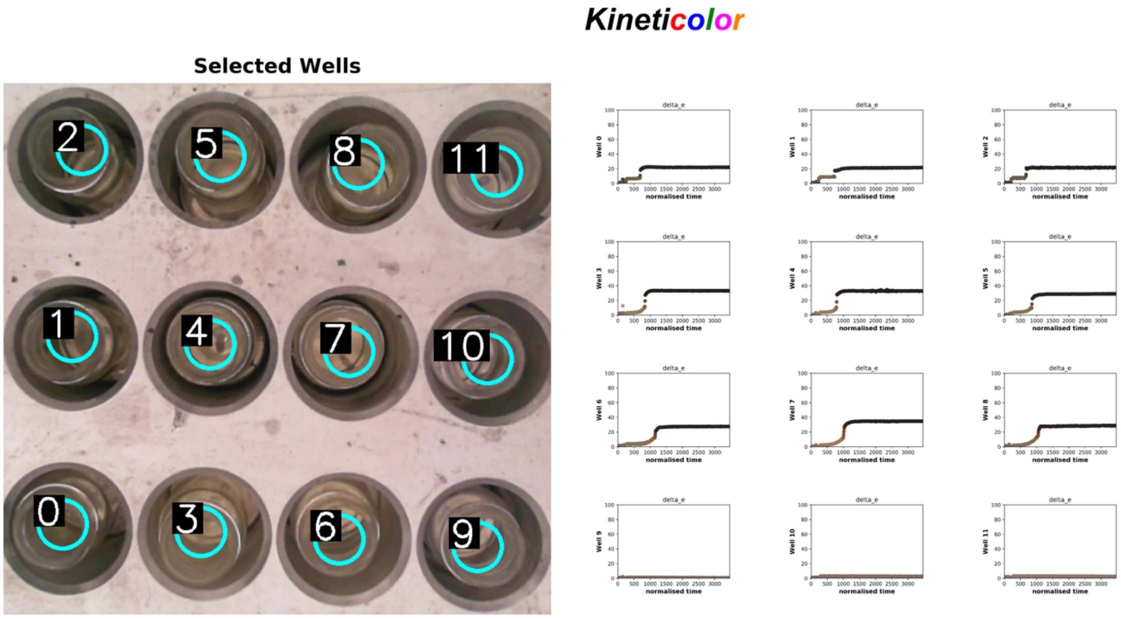

Figure S20. Exemplar Kineticolor high throughput ΔE versus time output for all 12 wells recorded for the Pd-black study. All machine-readable outputs of this analysis are included in the zipped folder.

6. Copper acetate sediment analysis

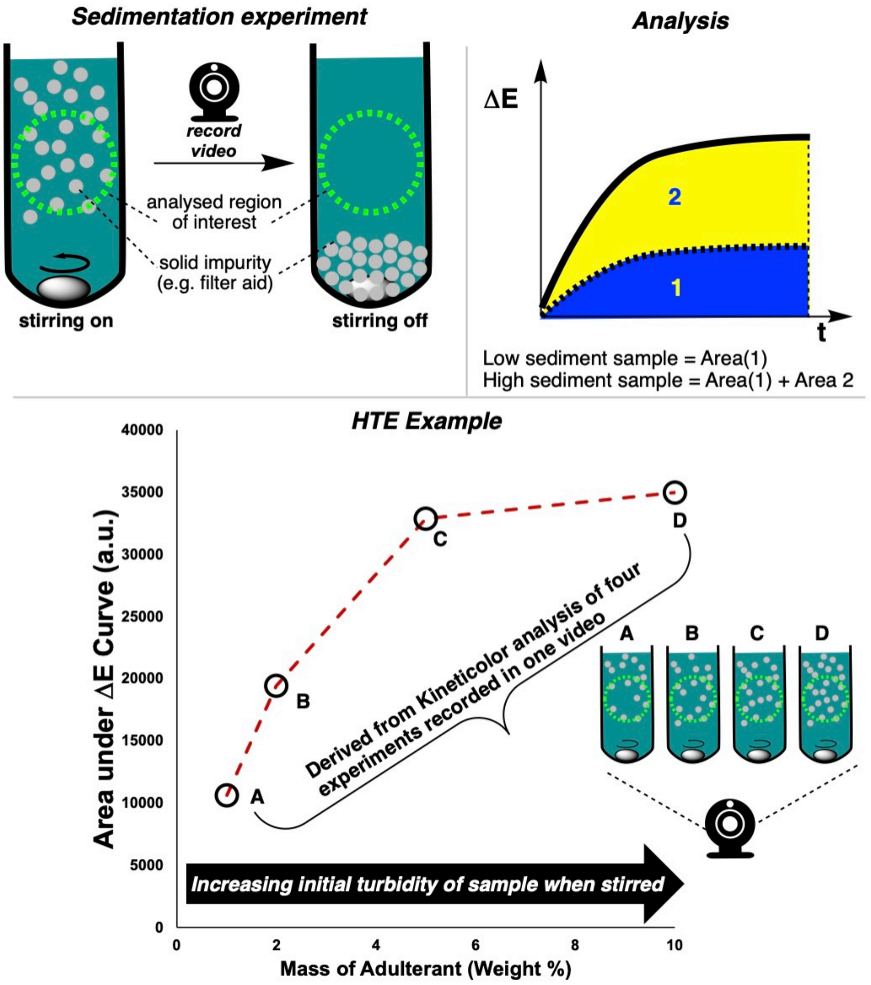

Figure 12 (reproduced). Use of multi-region Kineticolor analysis to provide sedimentation analysis in high throughput. Top left: Schematic of the computer vision experiment. Top right: Intuition for the meaning of area under curve in the current context. Bottom: Analysis of four adulterated copper acetate samples, the data for which were collected in a single video file of all four processes being run simultaneously.

6.1 Reaction set-up

1 4 test tubes were set in a rack.

2 To each was added a stirrer bar (12 x 4 mm with central pivot bar).

3 5 mL of liquid was used in each test tube. From left to rights 5 mL of a saturated copper acetate solution in 80% acetic acid into each test tubes. To these solutions, in each test tube, was added the following quantities of perlite:

Test tube	Mass of perlite (g)	Weight %
**1**	0.05	1
**2**	0.10	2
**3**	0.25	5
**4**	0.5	10

4. The test tube rack was place on the centre of the stirring plate and the stirrer set to 400 rpm.
5. The full set up was placed inside a 45 cm^3 Godox light box with two light panels switched on to maximum.
6. The solutions were stirred in parallel for 2 minutes.
7. The camera recording was started, and the mixing stopped.
8. The settling of perlite in the test tubes was then recorded for ~ 3 hour.

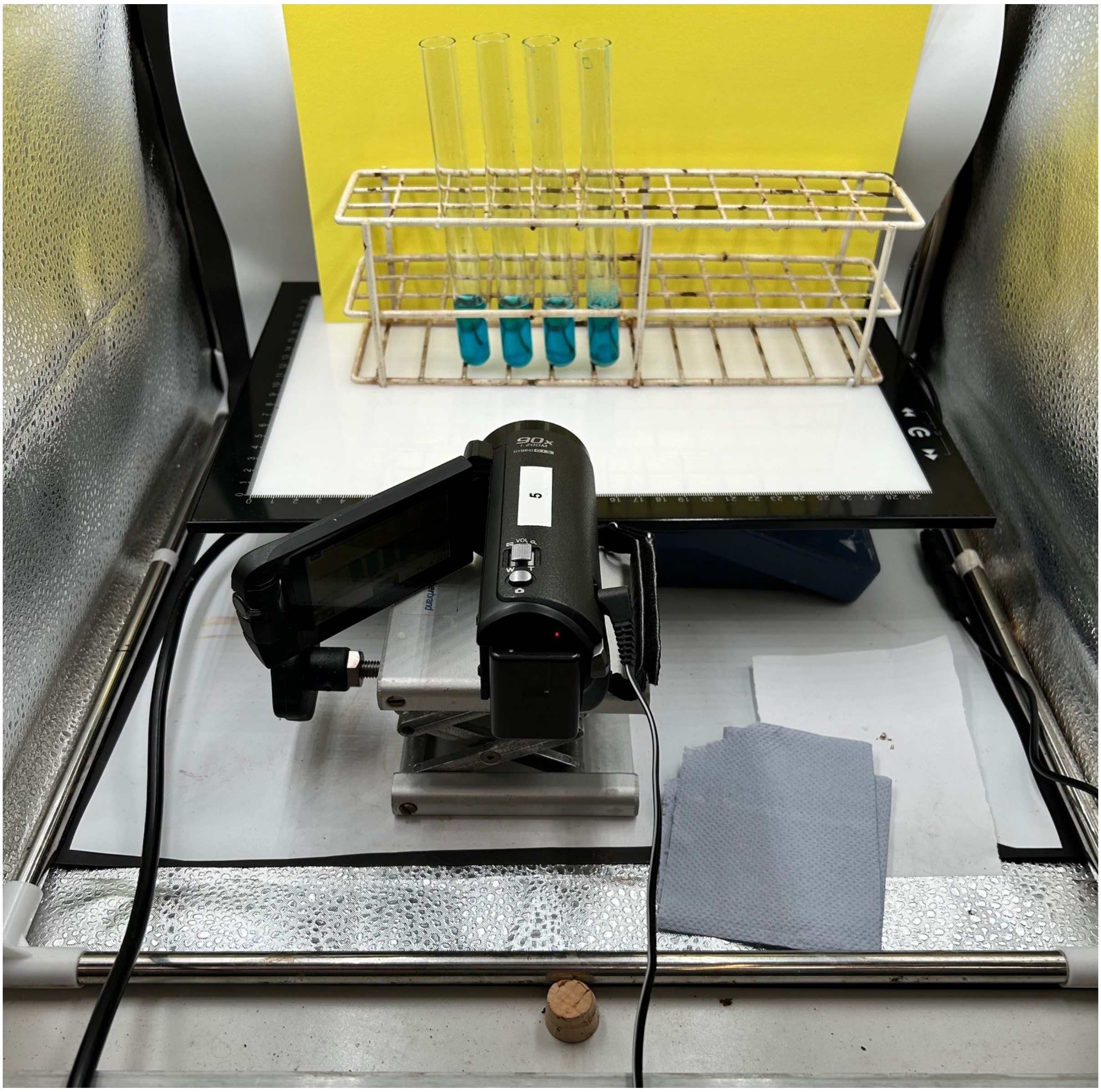

Figure S21. Key components of the reaction set-up for the sedimentation study.

6.2 Computer vision analysis of sedimentation

The multi-region tool in Kineticolor was used to set up four parallel circular regions of interest, each of maximum possible diameter across the width of the test tube. These regions were also placed so that the top of the circles lay just under the meniscus of the mixture.

The ΔE vs time profiles extracted from each region of interest in the video were plotted and their area-under-curve calculated using Simpson's rule as coded in the Python script named Sed_area.py, available in the machine-readable supporting information zipped folder.

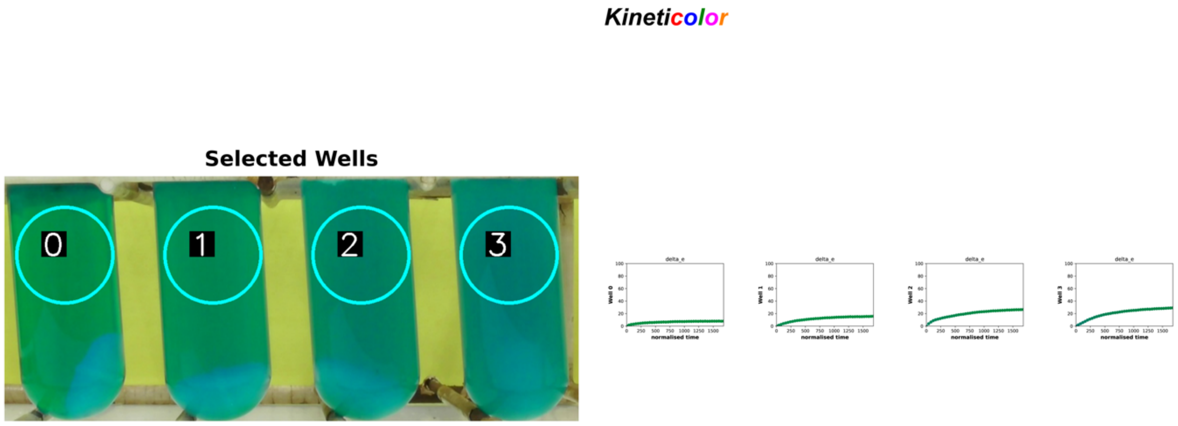

Figure S22. Exemplar Kineticolor output for the core sedimentation study. Left to right: vials represent increasing quantities of perlite suspended in the saturated copper acetate solution. All machine-readable outputs of this analysis are included in the zipped folder.

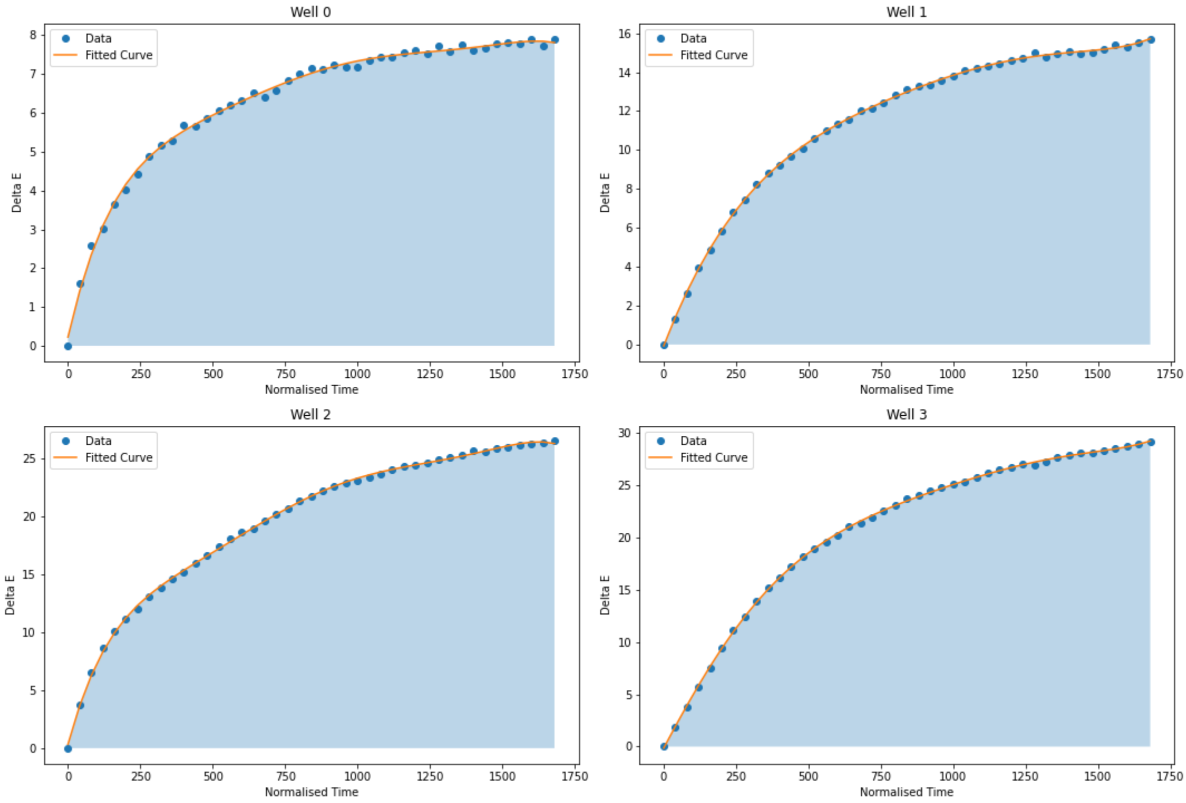

Figure S23. Outputs from area-under-curve calculations, based on the ΔE vs time profiles created in Kineticolor.

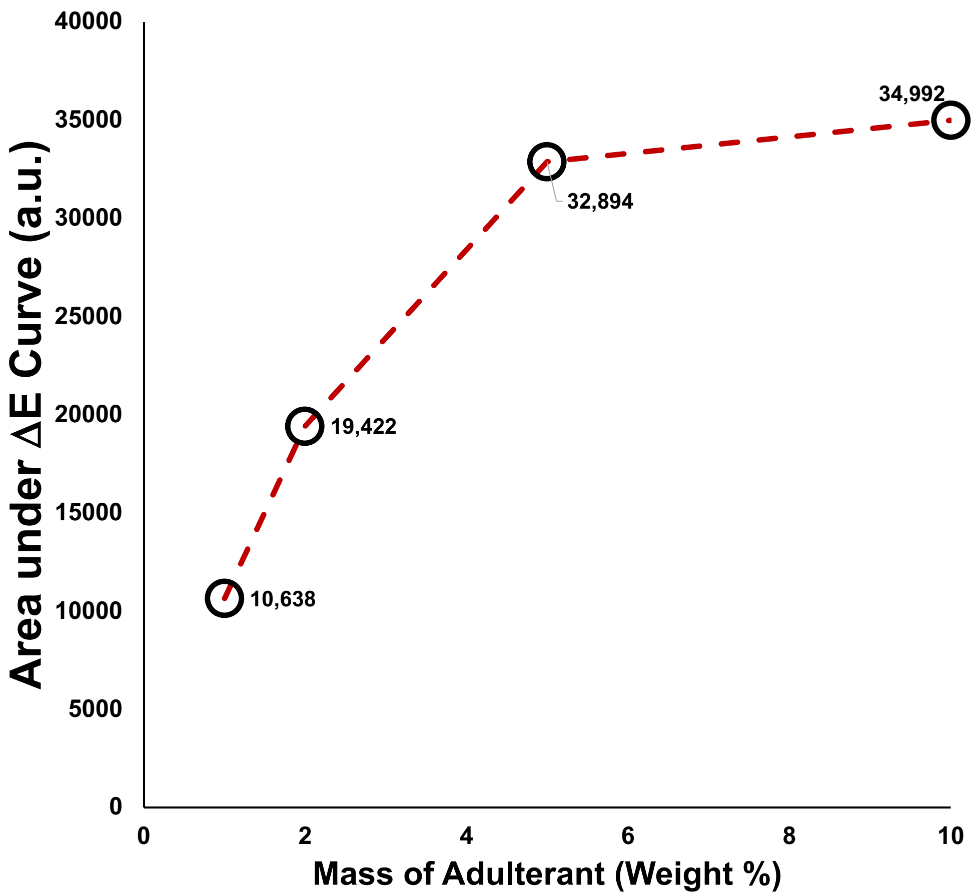

Figure S24. Increasing area-under-curve values, related to increasing turbidity and sediment settling time across the series of test tubes analysed in parallel.

6.3 Additional experiments using graded samples from manufacturing effluent

In addition to the key experiments described in the manuscript, we used the same procedure to assess two samples from copper acetate manufacture supplied from Bardyke Chemicals Ltd. One of the two samples was categorised as Grade 1 of 3, the other Grade 3 of 3. These values are derived from by-eye tests of turbidity and sedimentation rate by company technicians. Grade 1 represents the highest quality material, with little or no visible sedimentary impurity. Grade 3 is the worst possible grading, denoting appreciable visible sediment.

In this case, parallel monitoring and Kineticolor analysis was performed using two regions of interest per test tube. This was done firstly to demonstrate that the high throughput functionality need not mean one region per reaction. Second, the two regions analysed per test tube could be used to assess any differences in the amount of sediment suspended along different vertical positions along the height of the test tube.

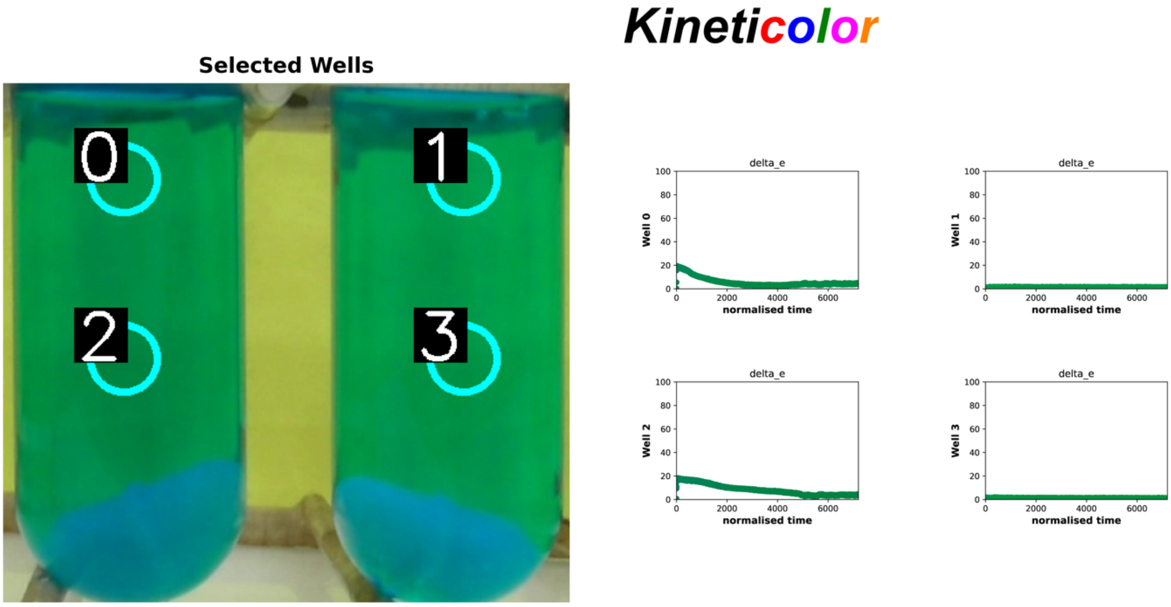

Figure S25. Exemplar Kineticolor output for additional sedimentation study. The left test tube relates to a poorer (Grade 3) sample; the right tube relates to the higher quality Grade 1 sample. All machine-readable outputs of this analysis are included in the zipped folder.

Consistent with these qualitative gradings of each sample, the calculated area under the ΔE vs time curve was much greater (approximately six-fold) for the poorer Grade 3 sample, containing most sediment, versus the higher quality Grade 1 sample, containing little visible sediment. In the final quantitative comparison of the test tubes, the two areas calculated for a single test tube were combined to give the final area used to quantify sample quality, shown in Figure S26.

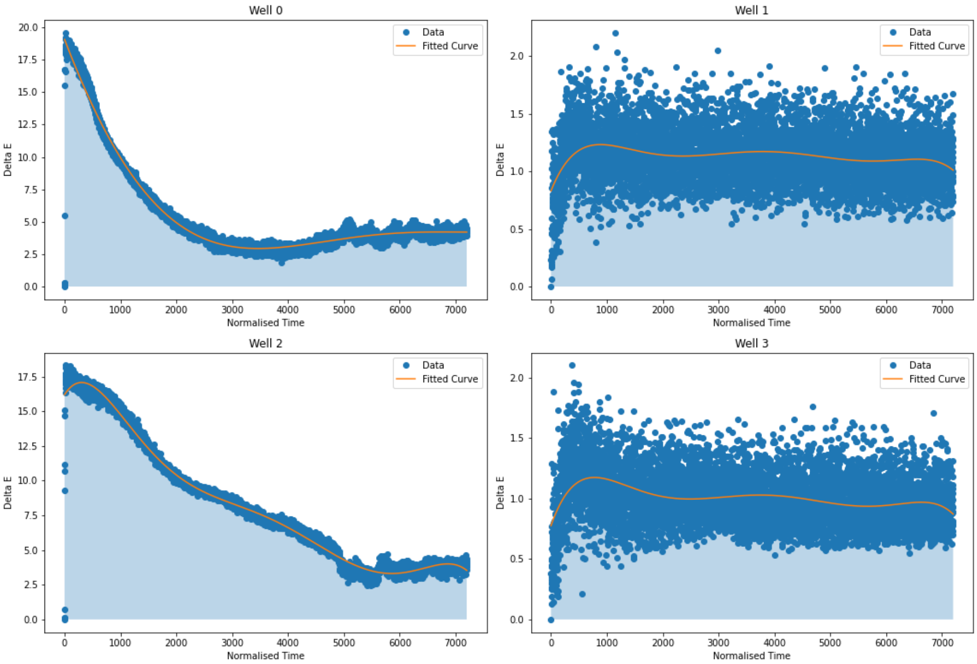

Figure S26. Outputs from area-under-curve calculations for additional graded copper acetate samples, based on the ΔE vs time profiles created in Kineticolor.

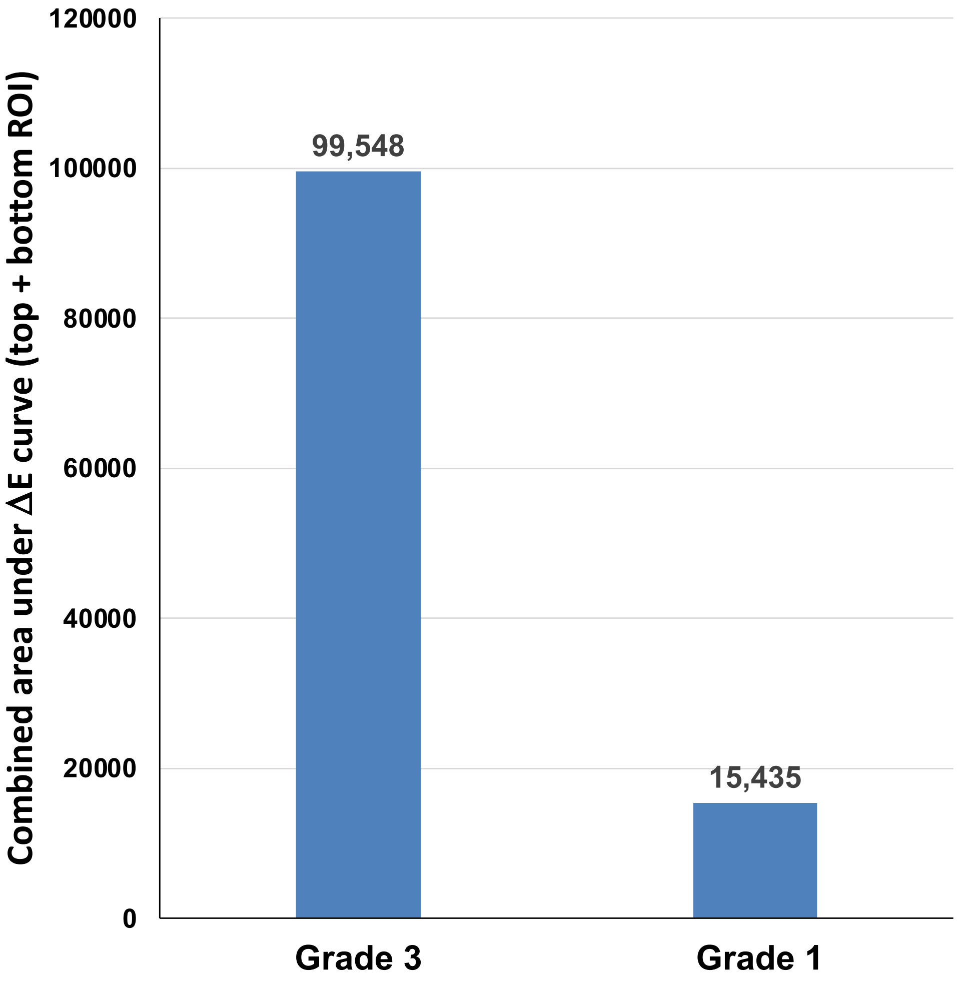

Figure S27. Summed areas under curves for the two ROIs calculated for each test tube. The higher area value for the poorer Grade 3 sample vs Grade 1 is consistent with the by-eye grading system.

7. DNSA reduction with reducing sugars

7.1 Parallel reaction set-up

1. An oil bath was pre-heated on a hot plate stirrer to the target temperature of 70 ºC. In this case, a stable temperature of 70-73 ºC. was achieved by setting the hotplate temperature thermocouple gauge to 85 ºC.
2. A 100 mL stock solution of NaOH (8 g, 0.2 mol, 2M) in deionised water was prepared.
3. A DNSA stock solution was prepared, using the stock solution of NaOH used in step 2. To do this, DNSA (0.5 g, 0.002 mol) was weighed into a 100 mL volumetric flask. To this, 20 mL of the NaOH stock solution added, resulting in the formation of a vibrant yellow solution. The DNSA stock was made up to 100 mL with deionised water.

4. The target mass of each sugar was weighed out, taking care to use the correct target mass based on whether the sugar is a mono- or di-saccharide. The masses used for each reaction were as follows:

Entry	Sugar	Mass in 100 mL stock solution (g)
**Sugar screen study**		
**1**	N/A (blank)	0
**2**	Glucose	0.101
**3**	Maltose	0.201
**4**	Sucrose	0.191
**Glucose vs Fructose sugar concentration study**		
**5**	Glucose	0.054
**6**	Glucose	0.162
**7**	Fructose	0.054
**8**	Fructose	0.162

5 The weighed sugar from the weighing boat to each reaction vessel (in this case, a 20 mL test tube).

6 A stirrer bar (2 cm oval) was added to each reaction vessel.

7 To the reaction vessel containing the sugar was added deionised water (5 mL).

8 DNSA stock solution (1 mL) was then added to each test tube at room temperature.

9 The reaction vessel was then mounted in a test tube rack that would later be submerged in the heated oil bath.

10 Steps 2-9 were repeated for all the sugars under study. For the blank solution (control reaction), without sugar, the reaction mixture contained everything EXCEPT a sugar.

11 Before heating the reaction vessels to start the reaction, the camcorder was set up according to the details provided below.

12 Video recording began while making final preparations to submerge the reaction vessels in the pre-heated oil bath.

13 The stirring rate on the hot plate was set to 750 RPM.

14 The reaction vessels were then submerged in the oil bath, ensuring the clamps were secure and stirring was even (not erratic).

15 The filming set-up and fume cupboard were not disturbed while video recording was in process.

16 The recording was stopped after approximately 30 mins, based on a by-eye judgement of the appearance of red colouration in the vessels where DNSA was expected to be reduced by the reducing sugars.

17 The reaction vessels were raised out of the oil bath and allowed to cool to room temperature.

18 Each reaction mixture to a 2-dram vial, sealed, and stored in a cool dry place. These samples were then used for independent UV-vis measurements.

19 UV-vis samples dilute the stock reaction mixture by two thirds with distilled water before collecting spectra in a 1 cm plastic cuvette on the UV-vis instrument.

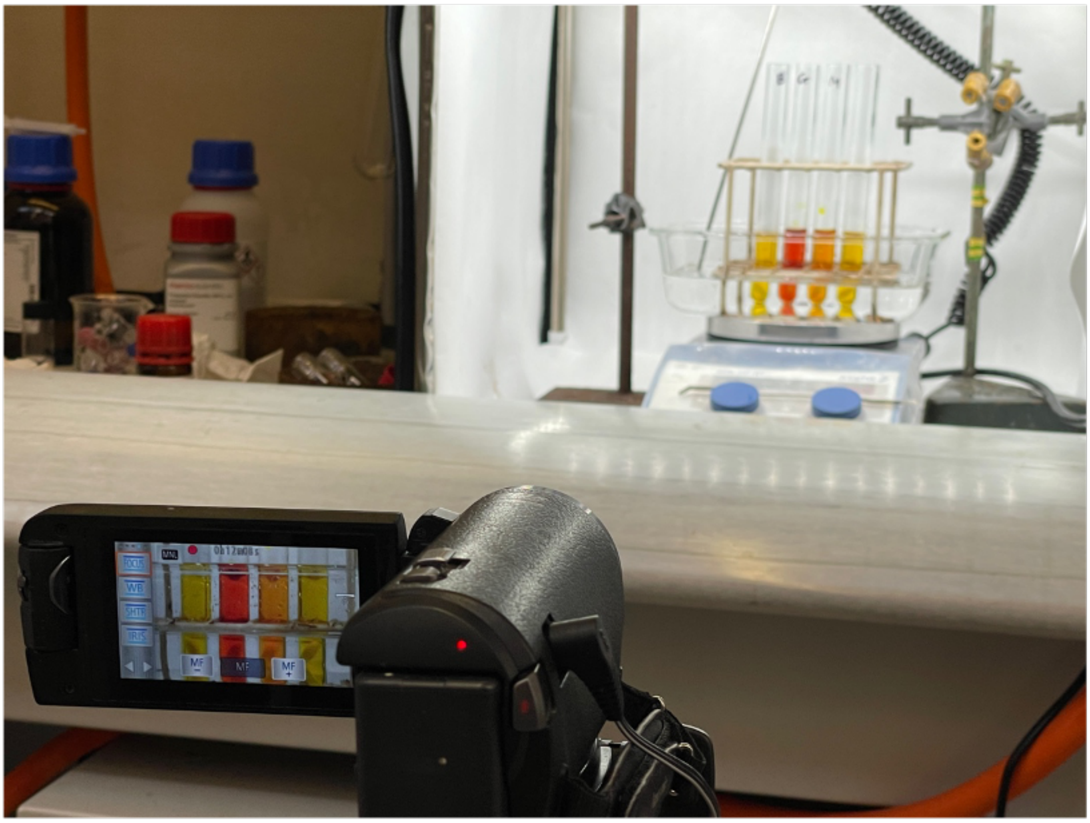

Figure S28. Key components of the reaction set-up used for the monitoring of DNSA reduction by reducing sugars under alkaline conditions.

Table S10. Camera settings employed for the reducing sugars study.

**Camera model**	Panasonic HC-W580
**Camera control (auto vs manual)**	Manual
**Camera ISO**	
**Camera zoom**	See video for approx. zoom relative to camera position
**Camera aperture**	F6.8
**Camera mount**	tripod
**Camera distance from monitored object(s)**	68 cm approx.
**Lighting hardware**	Godox 45 cm^3 lightbox
**Lighting intensity (lux; by independent brightness measurement)**	760.6
**White balance**	auto wb
**Shutter speed**	"1/50"

7.2 UV-vis data

Using the method above, UV-vis spectra were collected to all reaction mixtures, including the control, after exposure to the reaction conditions. In addition to the UV-vis spectra below, photographs of the reaction sample vials, resting atop an LED flat light, were analysed using Kineticolor's high throughput image analysis mode, providing averaged and pixel-resolved colour measurements for each sample.

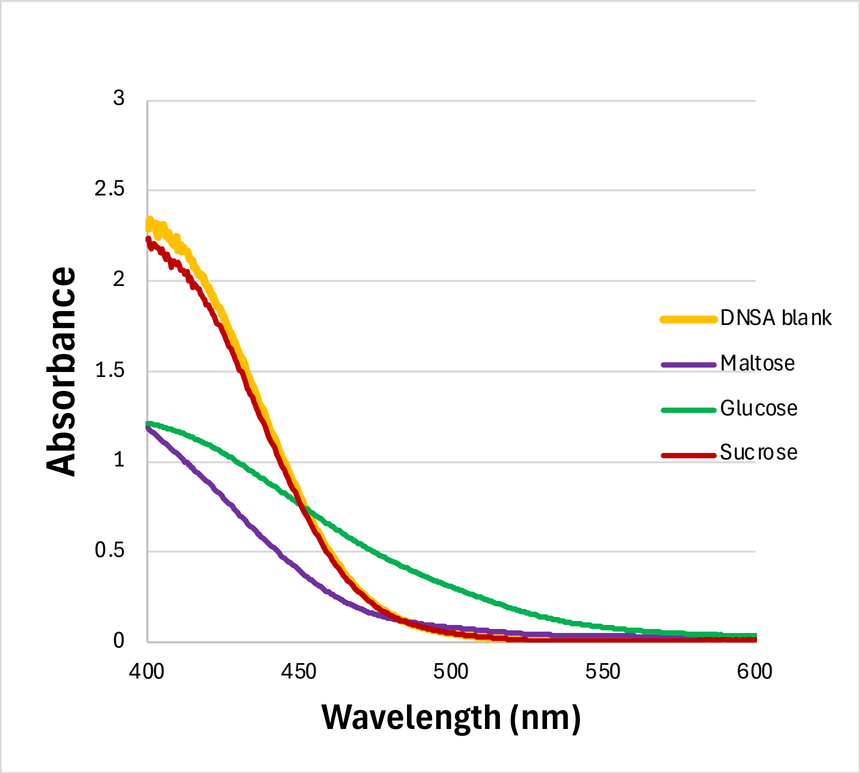

Figure S29. Co-plotted UV-vis spectra for reaction mixtures following the sugar screen.

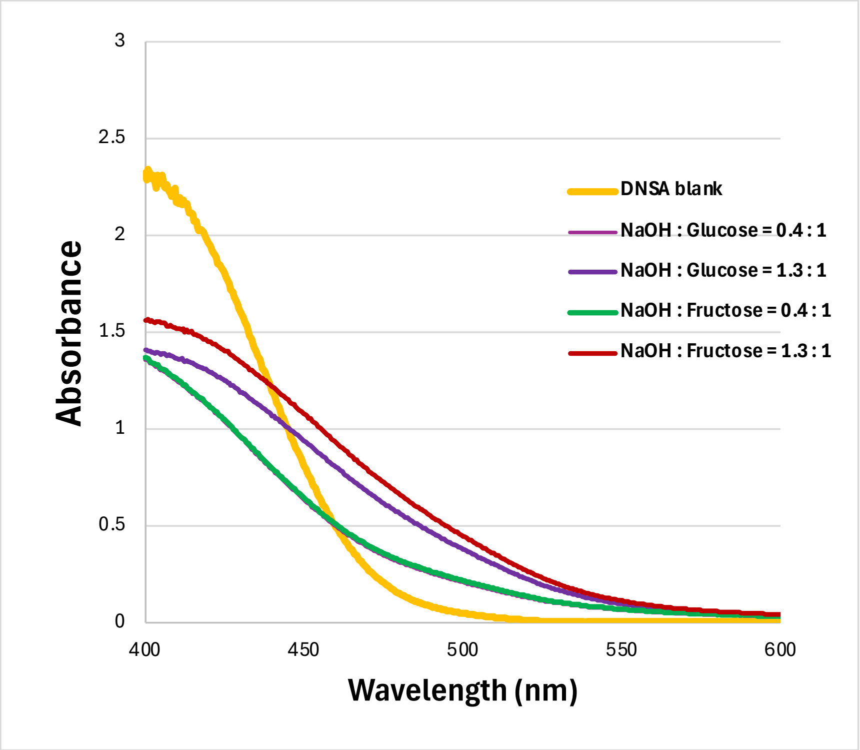

Figure S30. Co-plotted UV-vis spectra for reaction mixtures following the concentration study of glucose versus fructose.

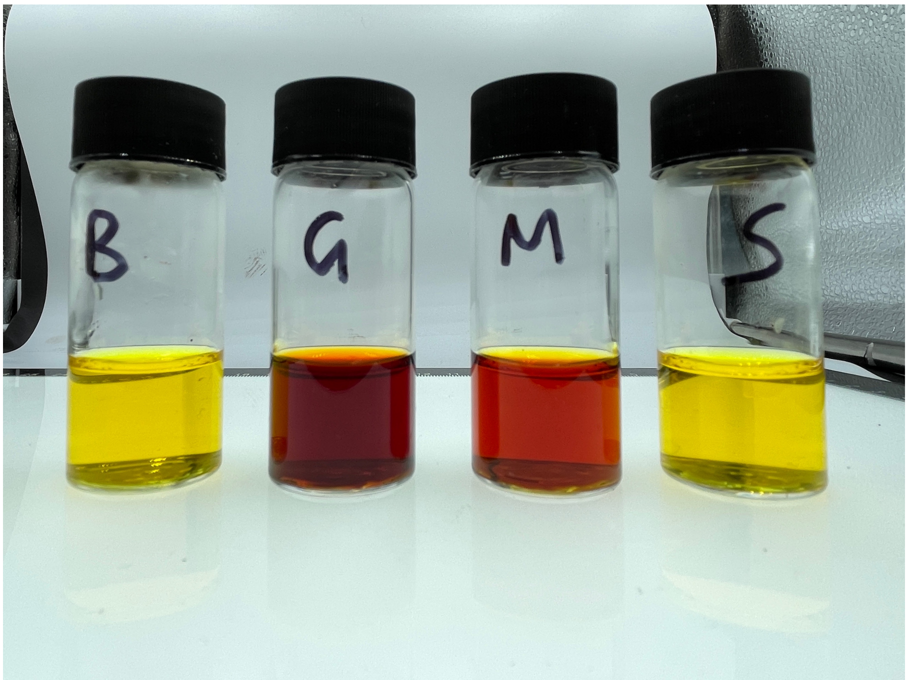

Figure S31. A photograph of all four reaction mixtures from the sugar screen. B = blank, G = glucose, M = maltose, S = sucrose. Redder solutions signify higher conversion of DNSA nitro reduction reaction.

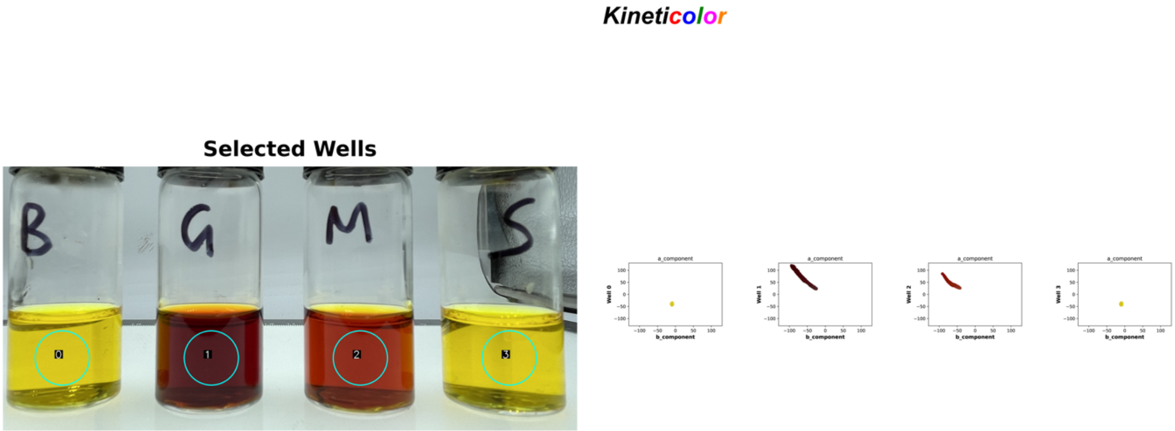

Figure S32. Exemplar Kineticolor high throughput image analysis output for all 4 wells recorded for the sugar screen part of the reducing sugars study, showing 2D plots of a* versus b* from the CIE–L*a*b* colour space. B = blank, G = glucose, M = maltose, S = sucrose. All machine-readable outputs of this analysis are included in the zipped folder.

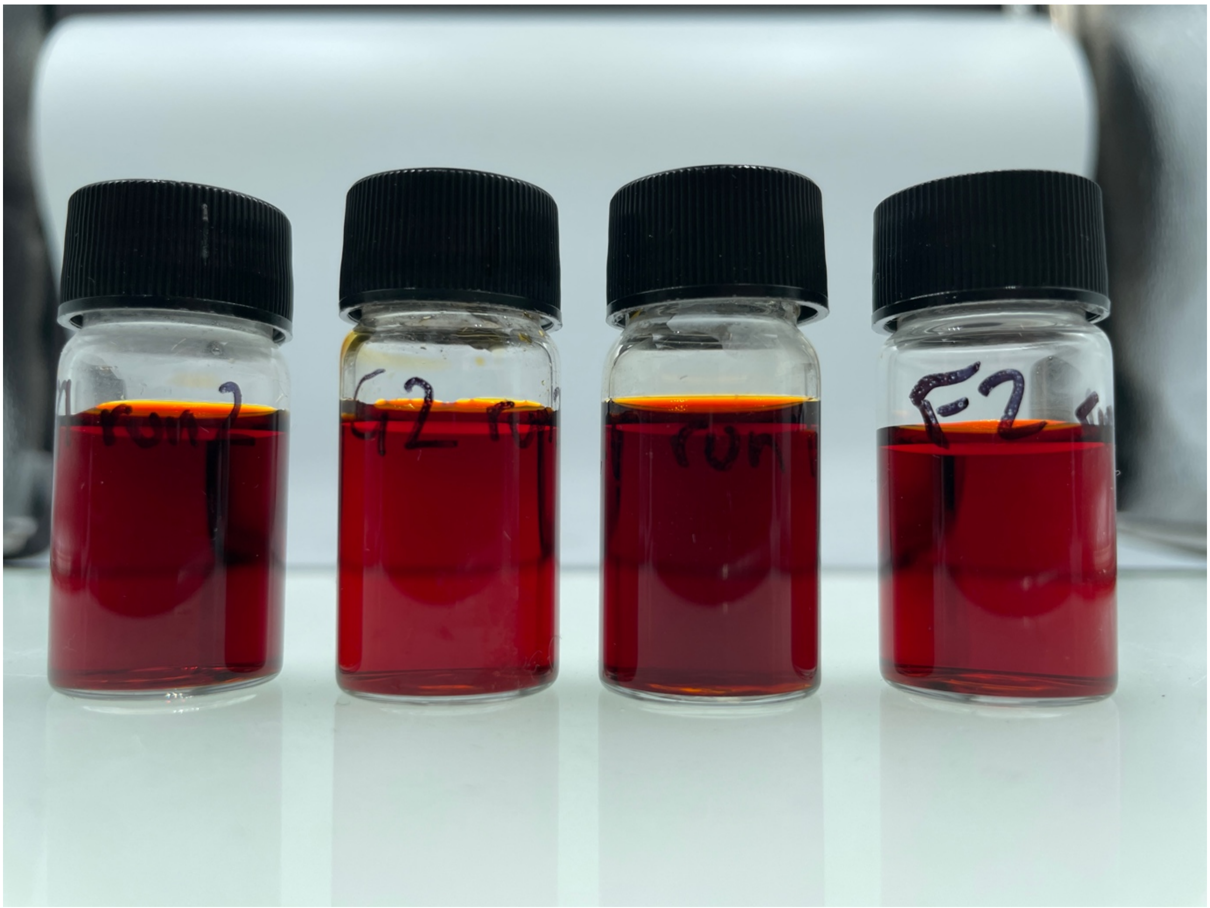

Figure S33. A photograph of all four reaction mixtures from the concentration study for glucose versus fructose. Left to right: (1.3 NaOH : 1 glucose), (0.4 NaOH : 1 glucose), (1.3 NaOH : 1 fructose), (0.4 NaOH : 1 fructose). Redder solutions signify higher conversion of DNSA nitro reduction reaction.

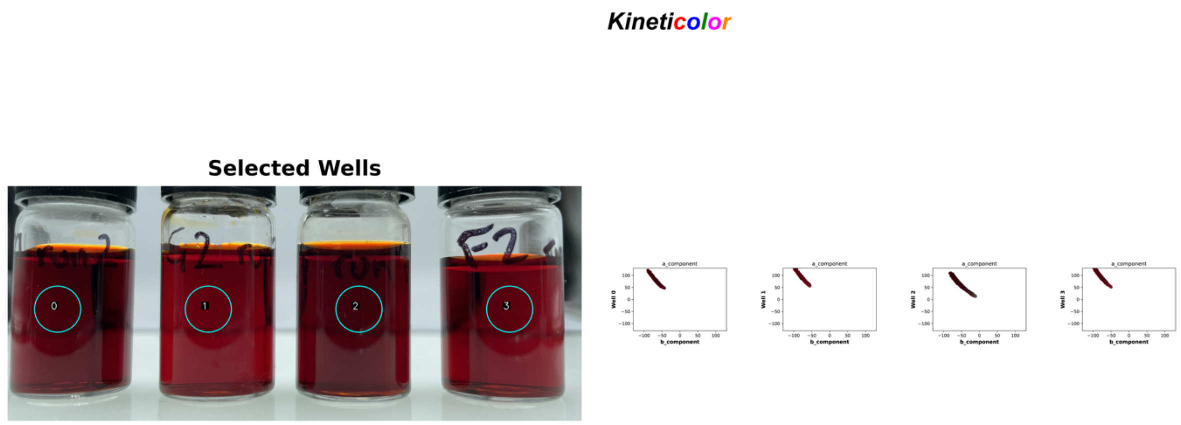

Figure S34. Exemplar Kineticolor high throughput image analysis output for all 4 wells recorded for the concentration comparison for glucose vs fructose in the reducing sugars study, showing 2D plots of a* versus b* from the CIE–L*a*b* colour space. Left to right: (1.3 NaOH : 1 glucose), (0.4 NaOH : 1 glucose), (1.3 NaOH : 1 fructose), (0.4 NaOH : 1 fructose). All machine-readable outputs of this analysis are included in the zipped folder.

8. Kineticolor Analysis

Video analysis was performed using the developmental video analysis software, *Kineticolor*. Earlier applications of this software have been reported in the literature, with examples including:

- DOI: https://pubs.acs.org/doi/full/10.1021/acs.oprd.2c00216
- DOI: https://doi.org/10.1039/D2SC05702F
- DOI: https://doi.org/10.1039/D3SC01383A

All video data in this report were analysed using Kineticolor version 0.3.2.

Videos were analysed by breaking videos into their constituent frames, and each frame being analysed, at the pixel level, in turn. A user-selected region of interest was analysed, averaging all pixel values in the selected range. All background data outside the selected region was ignored in the analysis. Data were analysed according to a user-selected number of frames to be skipped. Extracted colour data were provided from across a common subset of colour models, namely: RGB, HSV, CIE-L*a*b*, and CIE-XYZ. For the purposes of this report, analysis primarily focused on plots of ΔE versus time. ΔE is the colour-independent measure of contrast, measured as the Euclidean distance between two colours in the CIE_L*a*b* colour space.

Multi-ROI analysis is achieved by extracting multiple sets of pixels from each video frame being analysed. The ROIs are selected by the user first selecting a global rectangular outline ROI. Therein, the user then selects the desired number of rows and columns to produce a grid layout of equally spaced circular ROIs. The radius of the circular ROIs can be modified.

As part of the machine-readable data in the zipped folder provided, all Kineticolor outputs are provided alongside a config.txt file that documents the aforementioned user settings applied in each analysis.

A licensed version of the software is available on request from the University of Strathclyde, via the corresponding author and the Innovation and Industry Engagement team (quoting technology reference Tech 2141).

marc.reid.100@strath.ac.uk; ipr-manager@strath.ac.uk
