# Supplementary material for: Parallel and High Throughput Reaction Monitoring with Computer Vision
Source: Angew Chem Int Ed Engl. 2024 Oct 31;64(1):e202413395. doi: 10.1002/anie.202413395 (PMC11701362; doi:10.1002/anie.202413395)
Supplement: Supplementary file 2 — Supporting Information [file ANIE-64-e202413395-s002.zip › Supporting Info - Machine readable data part 1/Figure 7 - investigating camera lenses for well plate filming/Supplement - band-pass filters/AMcf_CV20/AMcf_CV20.docx]

**RISK ASSESSMENT (inc. COSHH) Lab Ref no.:** AMcf_CV20

**Short Reaction Description:** HTE Crystal Violet Kinetics

| **Assessor’s Name: Aaron Mcfall**  **Signature: *Aaron Mcfall***  **Supervisor’s**  **Signature: *[Marc’s signature]***  **Date: DD/MM/YYYY** | **Quantity** | Carcinogen, teratogen, mutagen | Toxic/very toxic | Harmful/irritant | Explosive | Pyrophoric | Flammable/highly flammable | Oxidising | Corrosive | Lachrymator | Other (please specify) |
| --- | --- | --- | --- | --- | --- | --- | --- | --- | --- | --- | --- |
| **Substance** |  |  |  |  |  |  |  |  |  |  |  |
| Crystal Violet  Sodium Hydroxide | ~ 45 mg  ~ 100 mL | X | X | X  X |  |  |  |  | X |  |  |
| Lab supervision needed? | **Yes** | | **No** | | | **On first attempt** | | | | | |

| Heating | Cryogens | Pressure | Vacuum | Other Equipment: |
| --- | --- | --- | --- | --- |
|  |  | Fume hood | Safety screen | Gloves (type): nitrile |
| Face mask | Dust mask | Overnight permit | Other Controls: | |
| Emergency procedures: | In case of fire, close fume hood sash and evacuate the lab.  For emergency assistance, dial ext. 2222 | | | |
| Disposal procedures: | NaOH neutralised, before disposal.  All reaction contents disposed *via* **non-halogenated waste** bottle. | | | |
| First aid: | In case of skin contact, wash with warm soapy water. Eye wash located at lab entrance. | | | |

| **Experiment Number** | | **Date** | **Summary** | | **References** | |
| --- | --- | --- | --- | --- | --- | --- |
| AMcf_CV20 | | 06/02/23 | Addition of OH anion to Crystal violet cation. Video of the reaction recorded of a well plate with filters | | Knutson T. et al.  Journal of Chemical Education, 2015, 1692-5, 92(10) | |
| **Chemicals** | | | | | | |
| **Name** | **Mol. Wt.** | **No. Moles** | **Mass** | **Actual mass used** | | **Volume** |
| **Crystal Violet** | 407.99 g/mol | 0.106 mmol | 43.8 mg | 43.2 mg | |  |
| **NaOH** | 40 g/mol | 0.004 mol | 160 mg | 161.8 mg | | 50 mL |
| Solvent(s) |  |  |  |  | |  |
| **Procedure** | | | | | | |
|   A 0.04M of NaOH was prepared from 161.8 mg and adding to 100 ml of deionized water stock solution in 100 mL volumetric flask and making up to the mark with deionized water. A stock solution of Crystal violet using 43.8 mg of crystal violet and deionized water in a 100 mL volumetric flask giving a concentration of 1.1 mM (0.11 mmol). The volumes below where pipetted a plastic well plate. The lighting used in the Godox lightbox LEDs 1 and 2 were turned on full as well as the LED sheet light. This can be seen in the set-up photo.  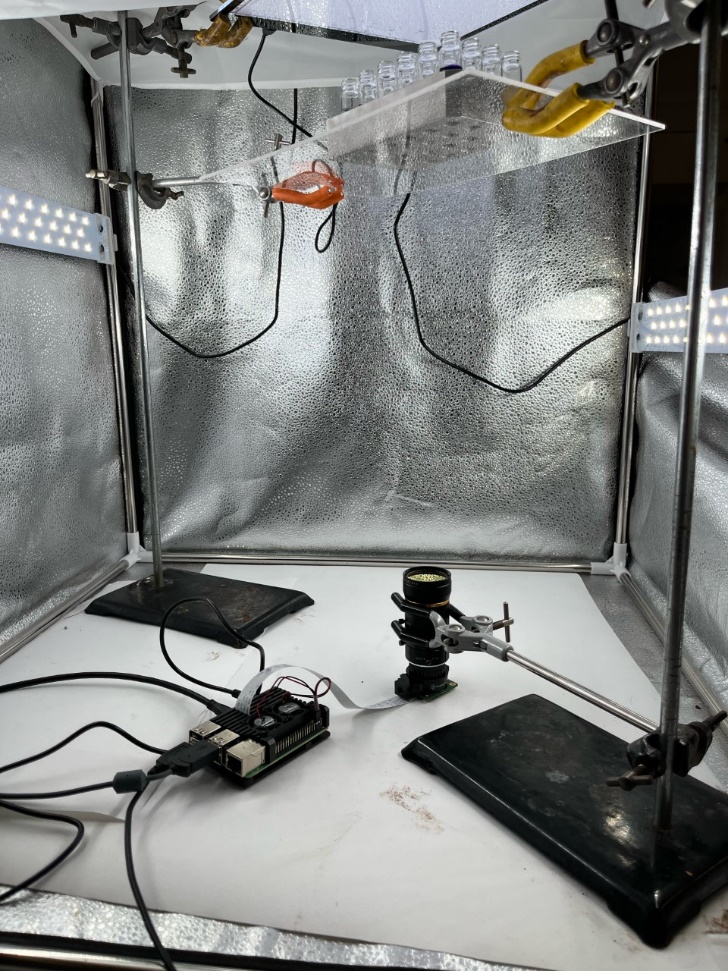   \|  \| A \| B \| C \| D \| \| --- \| --- \| --- \| --- \| --- \| \| 1 \| 1.5 CV, 1.5 Water \| 1.5 CV 0.5 NaOH 1.0 Water \| 1.5 CV 1.0 NaOH 0.5 Water \| 1.5 CV 1.5 NaOH \| \| 2 \| 0.8 CV 1.8 Water \| 0.8 CV 0.5 NaOH 1.7 Water \| 0.8 CV 1.0 NaOH 1.2 Water \| 0.8 CV 1.5 NaOH 0.7 Water \| \| 3 \| 0.4 CV 2.1 Water \| 0.4 CV 0.5 NaOH 2.1 Water \| 0.4 CV 1.0 NaOH 1.6 Water \| 0.4 CV 1.5 NaOH 1.1 Water \| \| 4 \| 0.2 CV 2.4 Water \| 0.2 CV 0.5 NaOH 2.3 Water \| 0.2 CV 1.0 NaOH 1.8 Water \| 0.2 CV 1.5 NaOH 1.3 Water \| \| 5 \| 0.1 CV 2.7 Water \| 0.1 CV 0.5 NaOH 2.4 Water \| 0.1 CV 1.0 NaOH 1.9 Water \| 0.1 CV 1.5 NaOH 1.4 Water \| \| 6 \| Three waters \| 0.05 CV 0.5 NaOH 2.45 Water \| 0.05 CV 1.0 NaOH 1.95 Water \| - 1. V 1.5 NaOH 1.45 Water \|   Volumes of NaOH were then added to the glass vials and placed into well plate as shown in the table above.  The lens used was the lens with a Green bypass filter on the camera lens. The camera settings that where input to the Raspi where: brightness 0.2, sharpness 5, awb indoor, t 9,000,000 | | | | | | |
| **Results** | | | | | | |
| - Video of reaction, color change. See data   Video of reaction (.mp4 and .h264 with Raspi)   - Color change expected (violet to colorless) - Kineticolour output for video | | | | | | |
| **Data**  Kineticolour data in Marc Reid group I-drive at: I:\Aaron McFall\e-lab books\Crystal Violet\AMcf_CV20\AMcf_CV20  Raw video in Marc Reid group I-drive at: I:\Aaron McFall\Experiment videos | | | | | | |
