# Supplementary figures and images for: Parallel and High Throughput Reaction Monitoring with Computer Vision (part 2 of 3)
Source: Angew Chem Int Ed Engl. 2024 Oct 31;64(1):e202413395. doi: 10.1002/anie.202413395 (PMC11701362; doi:10.1002/anie.202413395)

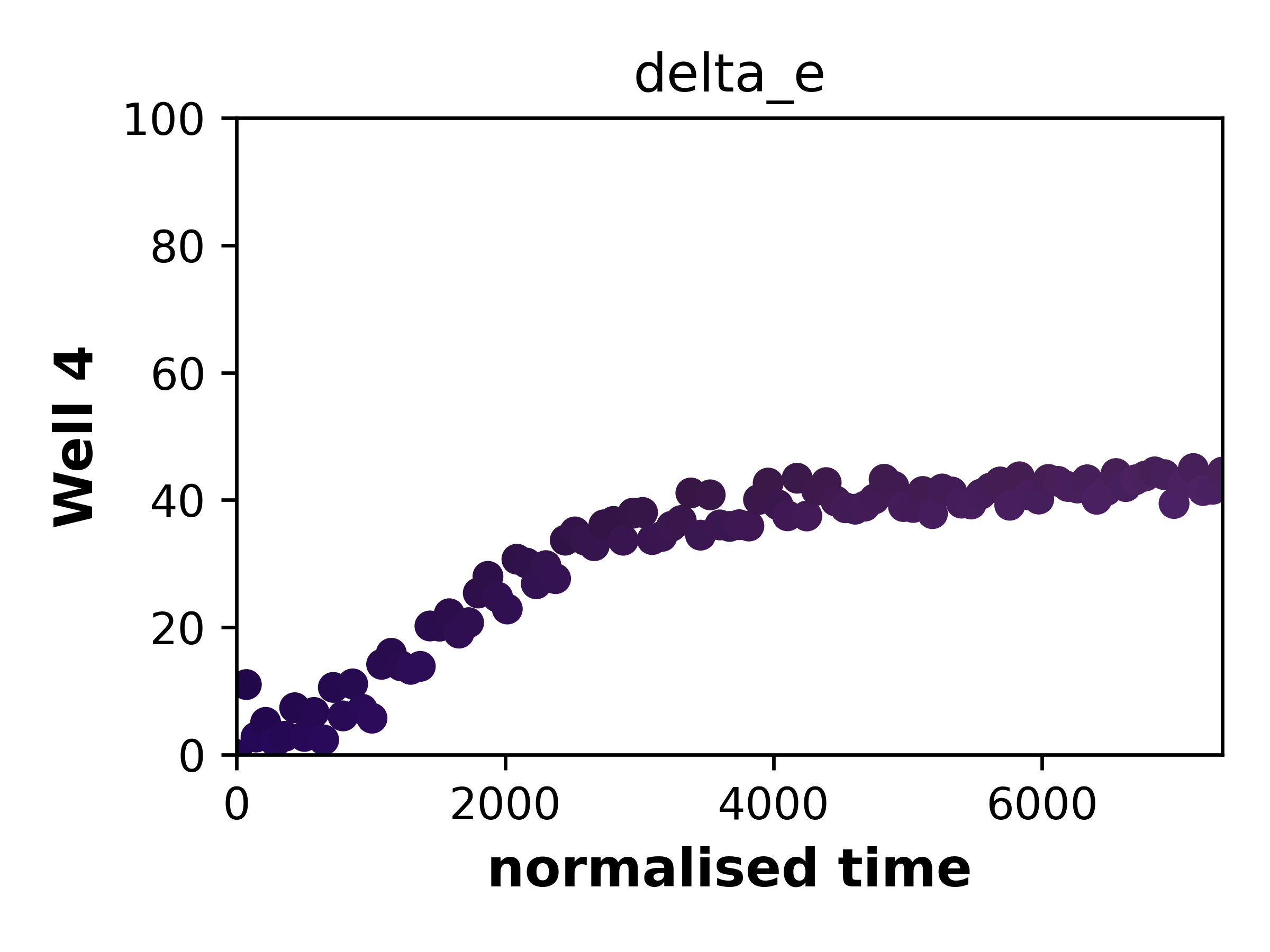

Supplement: Supplementary file 2 — Supporting Information [file ANIE-64-e202413395-s002.zip › Supporting Info - Machine readable data part 1/Figure 9 - crystal violet mixing analysis/Kineticolor outputs/temp/delta_e over normalised time - Well 4.png]

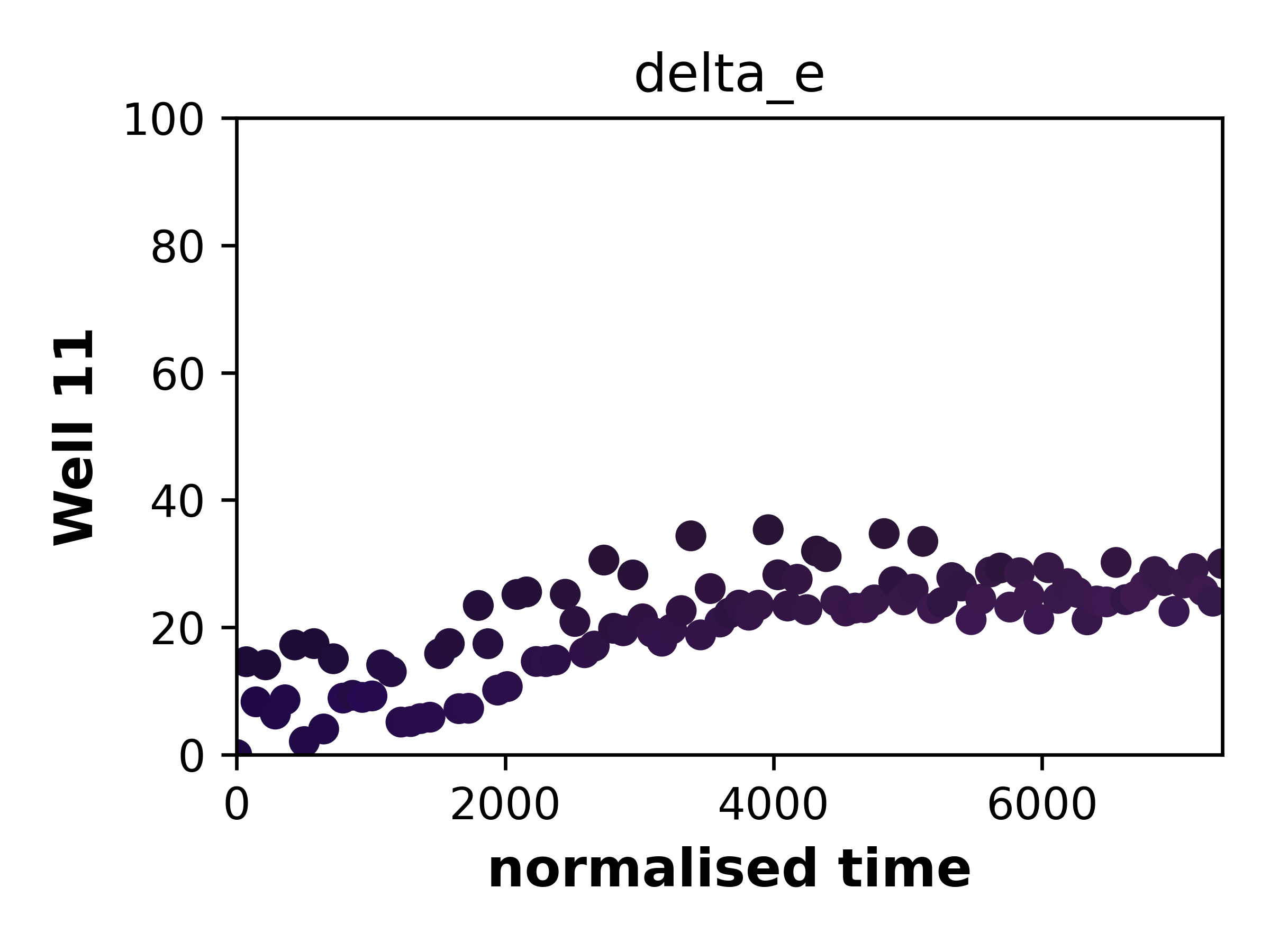

Supplement: Supplementary file 2 — Supporting Information [file ANIE-64-e202413395-s002.zip › Supporting Info - Machine readable data part 1/Figure 9 - crystal violet mixing analysis/Kineticolor outputs/temp/delta_e over normalised time - Well 11.png]

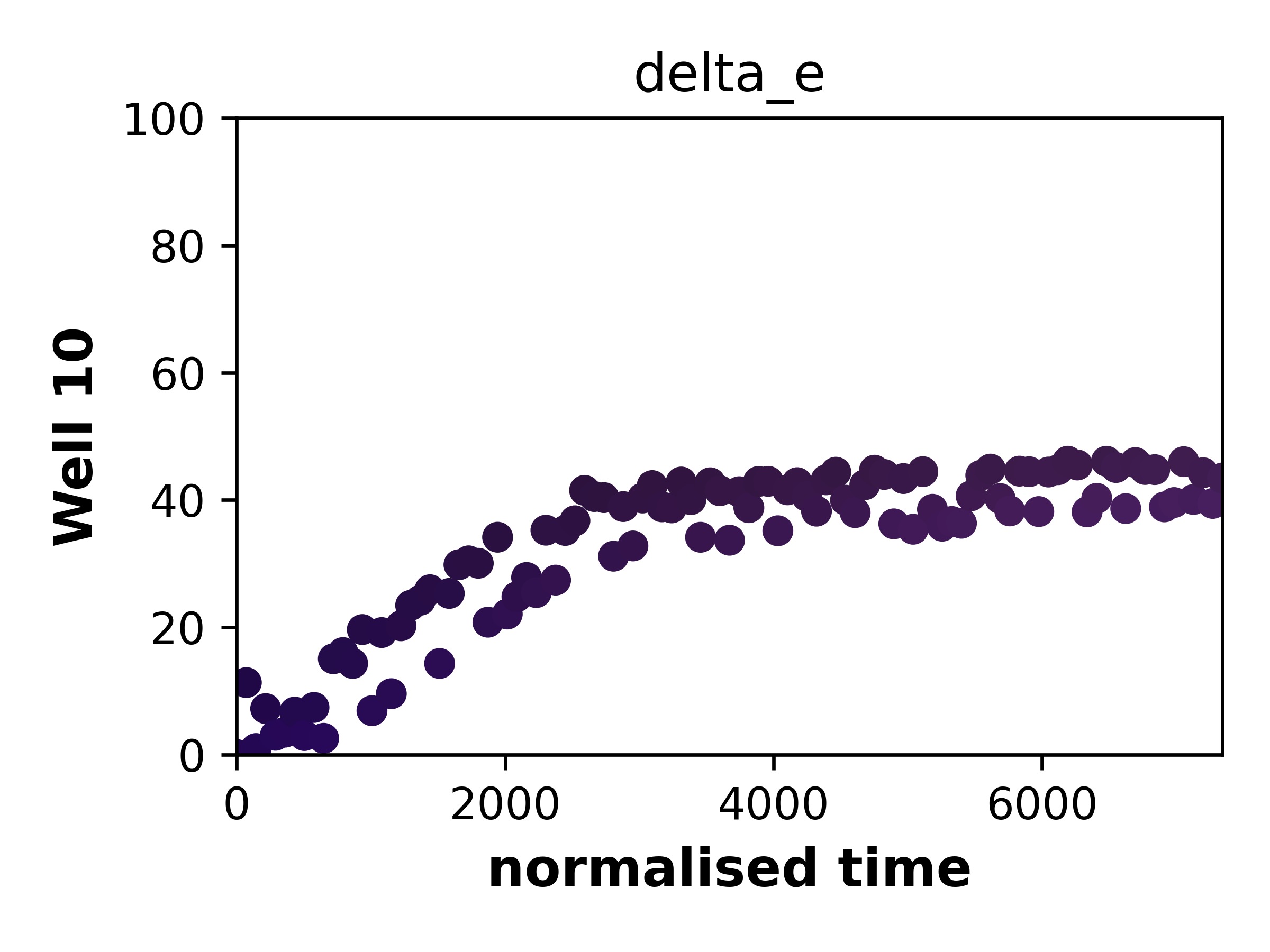

Supplement: Supplementary file 2 — Supporting Information [file ANIE-64-e202413395-s002.zip › Supporting Info - Machine readable data part 1/Figure 9 - crystal violet mixing analysis/Kineticolor outputs/temp/delta_e over normalised time - Well 10.png]

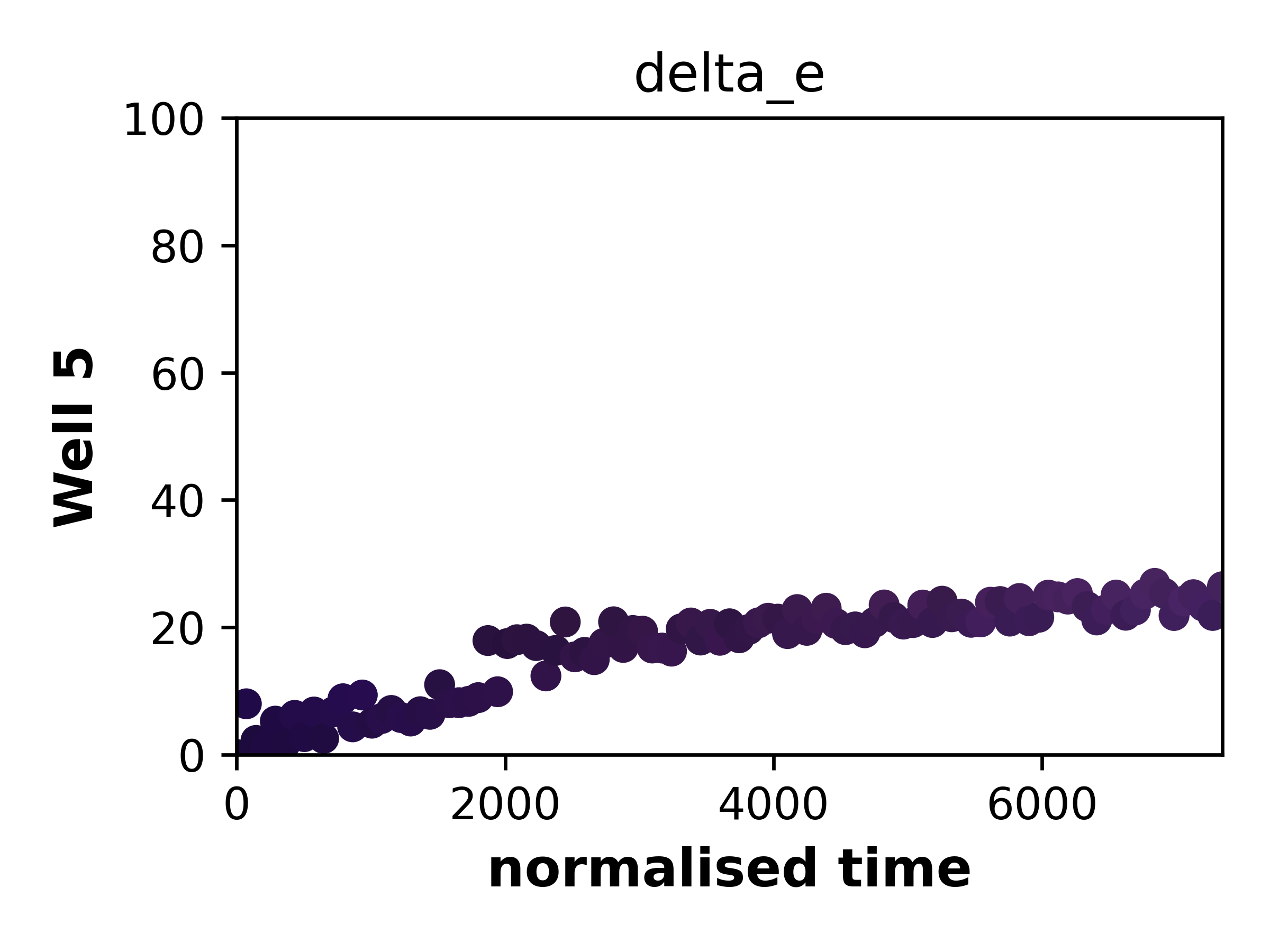

Supplement: Supplementary file 2 — Supporting Information [file ANIE-64-e202413395-s002.zip › Supporting Info - Machine readable data part 1/Figure 9 - crystal violet mixing analysis/Kineticolor outputs/temp/delta_e over normalised time - Well 5.png]

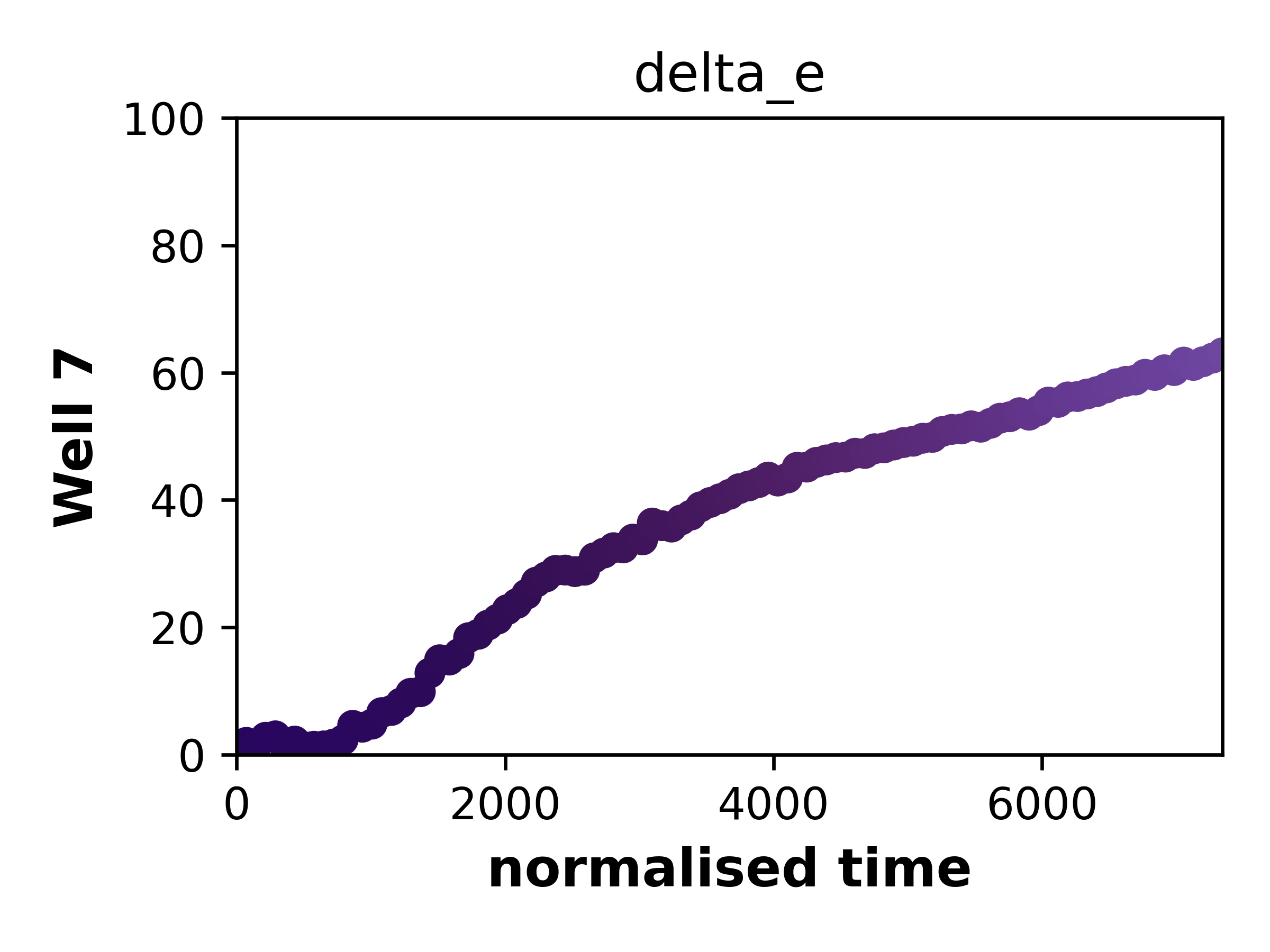

Supplement: Supplementary file 2 — Supporting Information [file ANIE-64-e202413395-s002.zip › Supporting Info - Machine readable data part 1/Figure 9 - crystal violet mixing analysis/Kineticolor outputs/temp/delta_e over normalised time - Well 7.png]

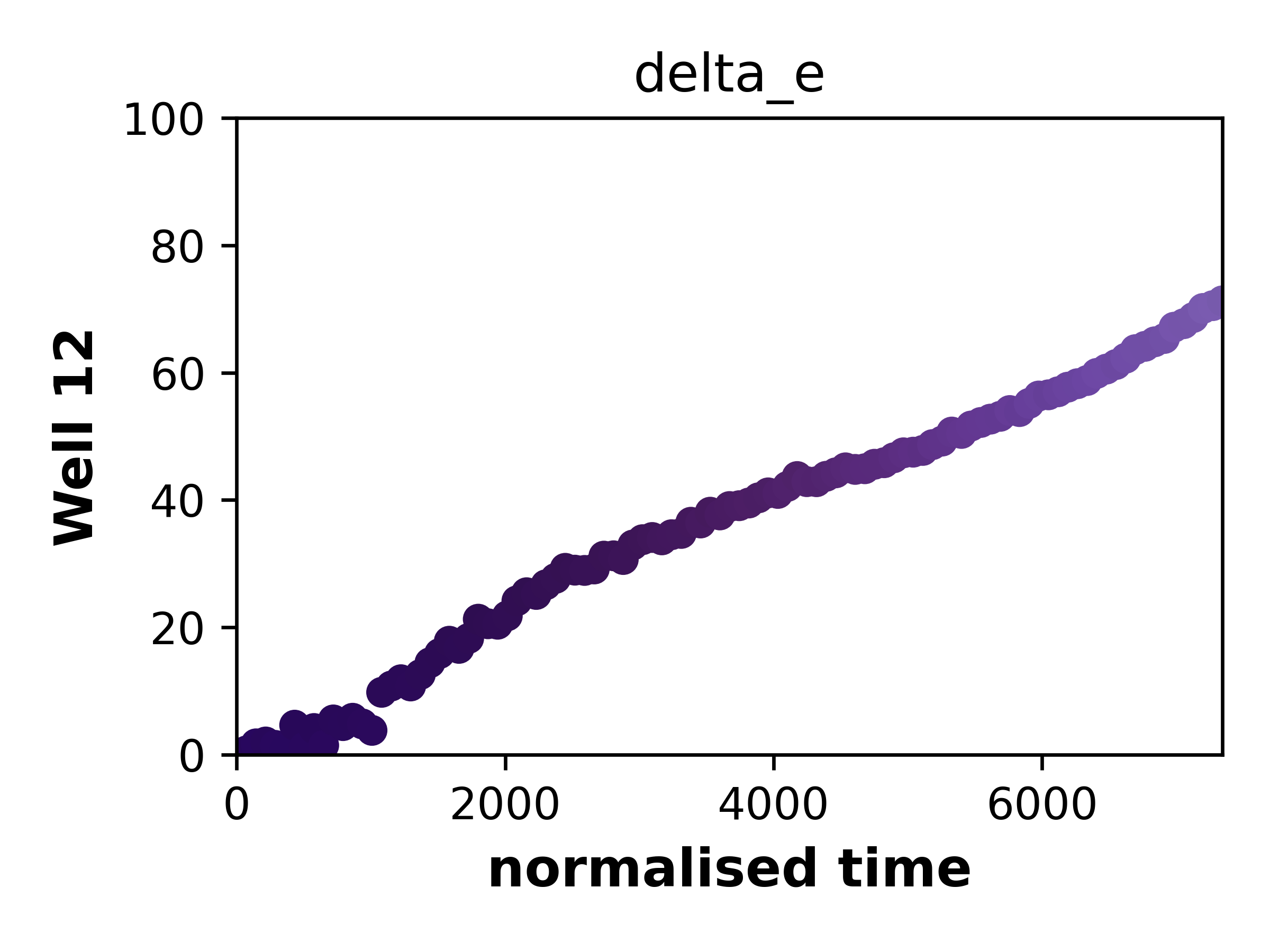

Supplement: Supplementary file 2 — Supporting Information [file ANIE-64-e202413395-s002.zip › Supporting Info - Machine readable data part 1/Figure 9 - crystal violet mixing analysis/Kineticolor outputs/temp/delta_e over normalised time - Well 12.png]

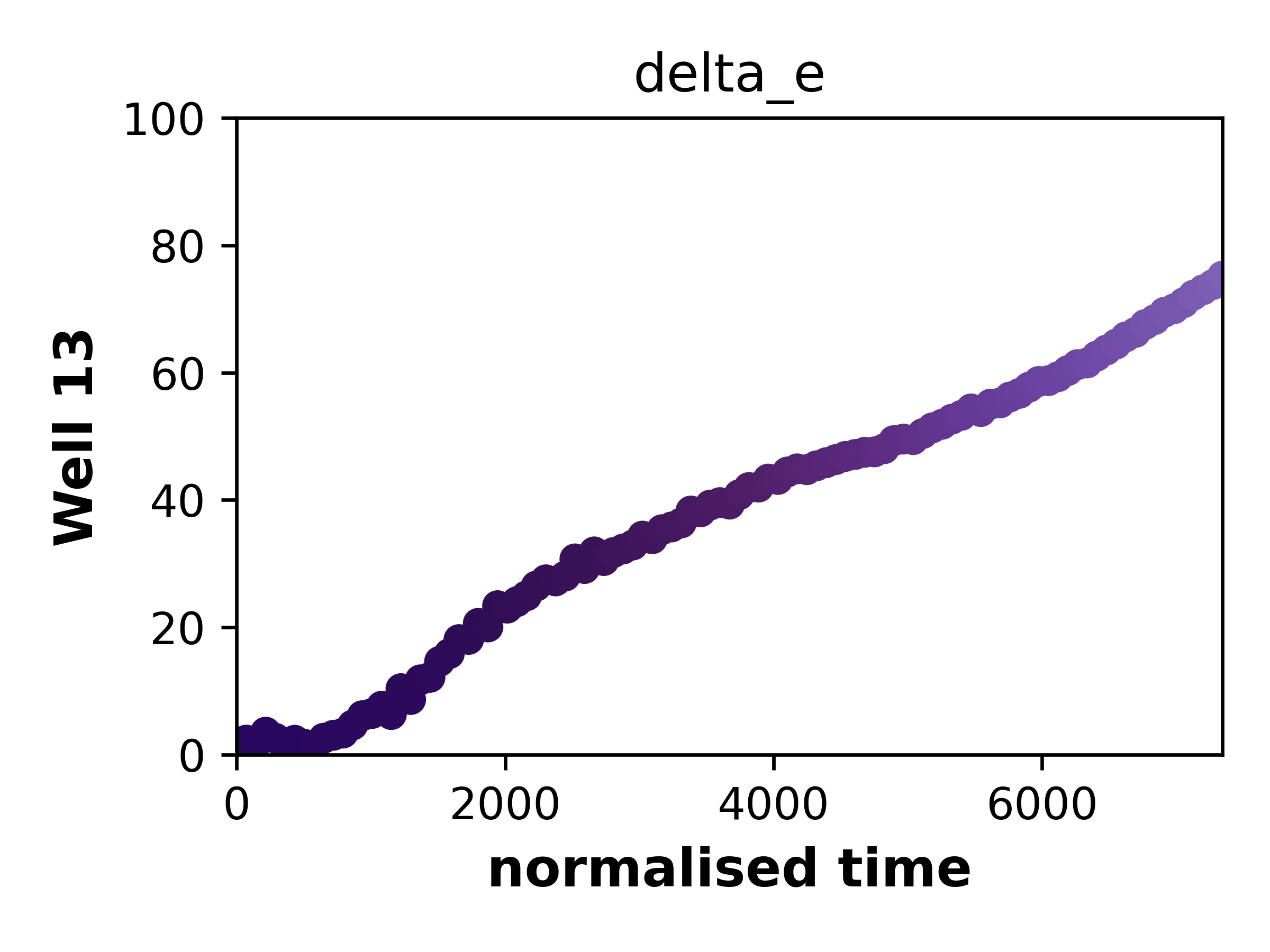

Supplement: Supplementary file 2 — Supporting Information [file ANIE-64-e202413395-s002.zip › Supporting Info - Machine readable data part 1/Figure 9 - crystal violet mixing analysis/Kineticolor outputs/temp/delta_e over normalised time - Well 13.png]

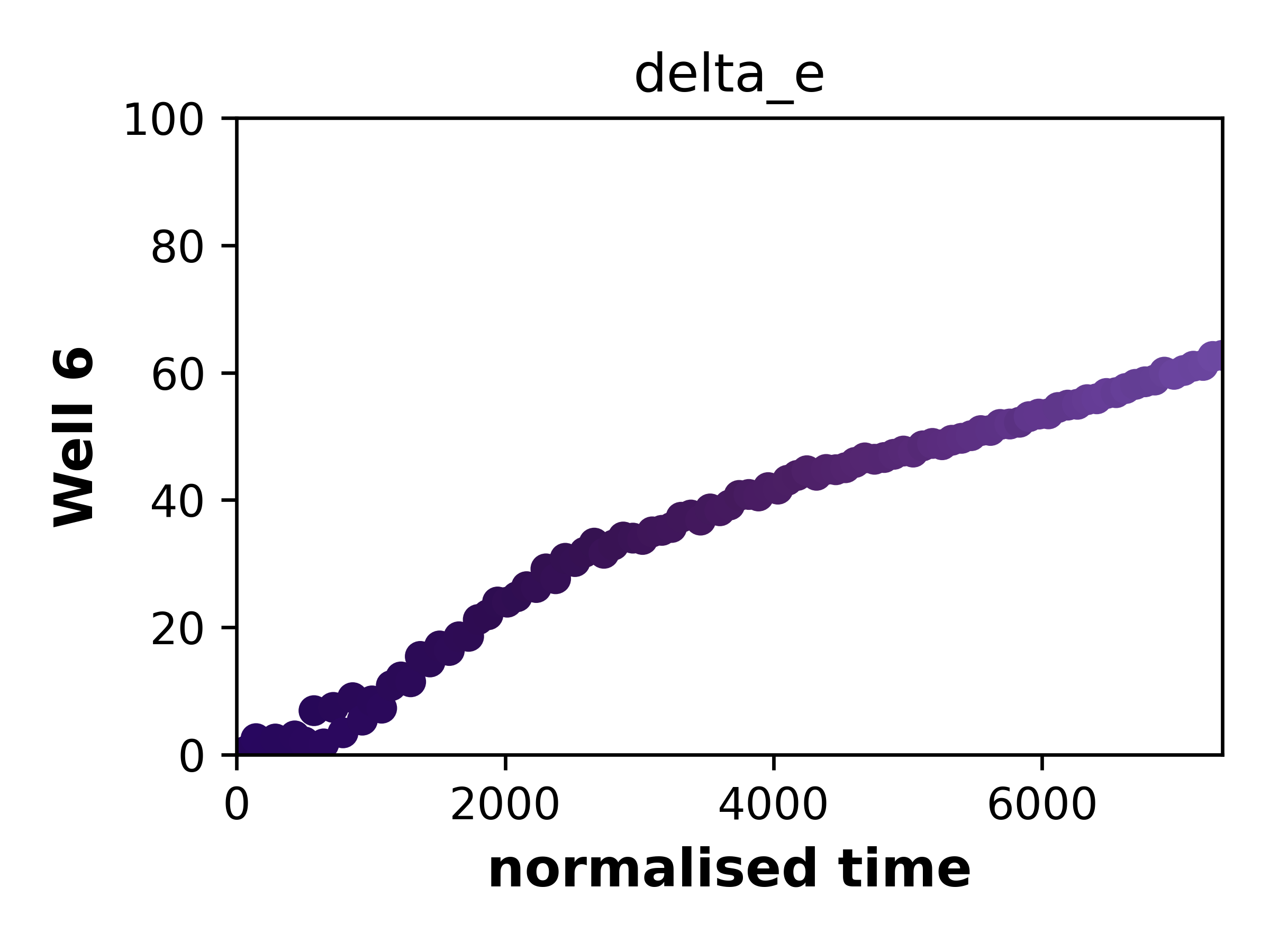

Supplement: Supplementary file 2 — Supporting Information [file ANIE-64-e202413395-s002.zip › Supporting Info - Machine readable data part 1/Figure 9 - crystal violet mixing analysis/Kineticolor outputs/temp/delta_e over normalised time - Well 6.png]

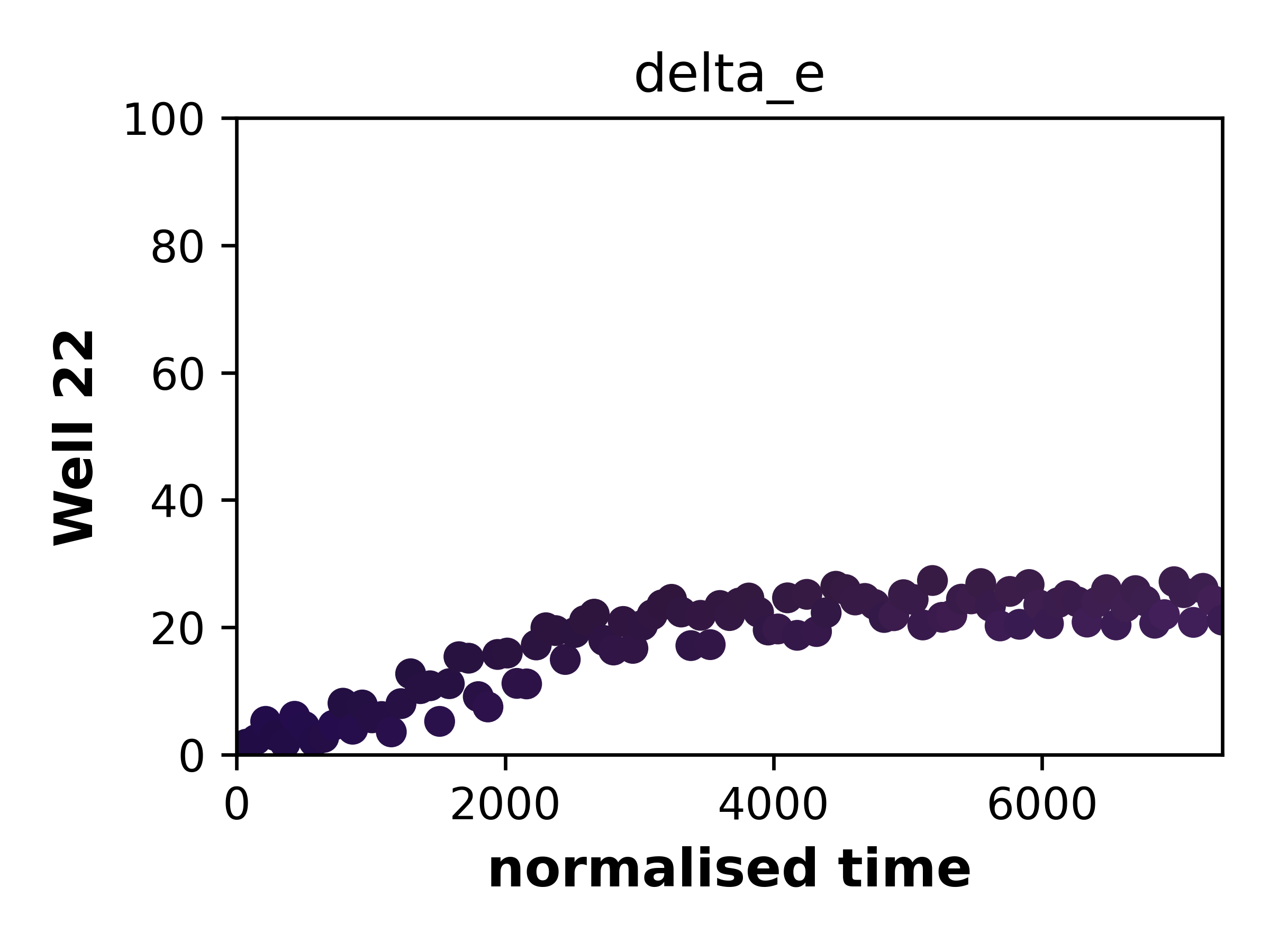

Supplement: Supplementary file 2 — Supporting Information [file ANIE-64-e202413395-s002.zip › Supporting Info - Machine readable data part 1/Figure 9 - crystal violet mixing analysis/Kineticolor outputs/temp/delta_e over normalised time - Well 22.png]

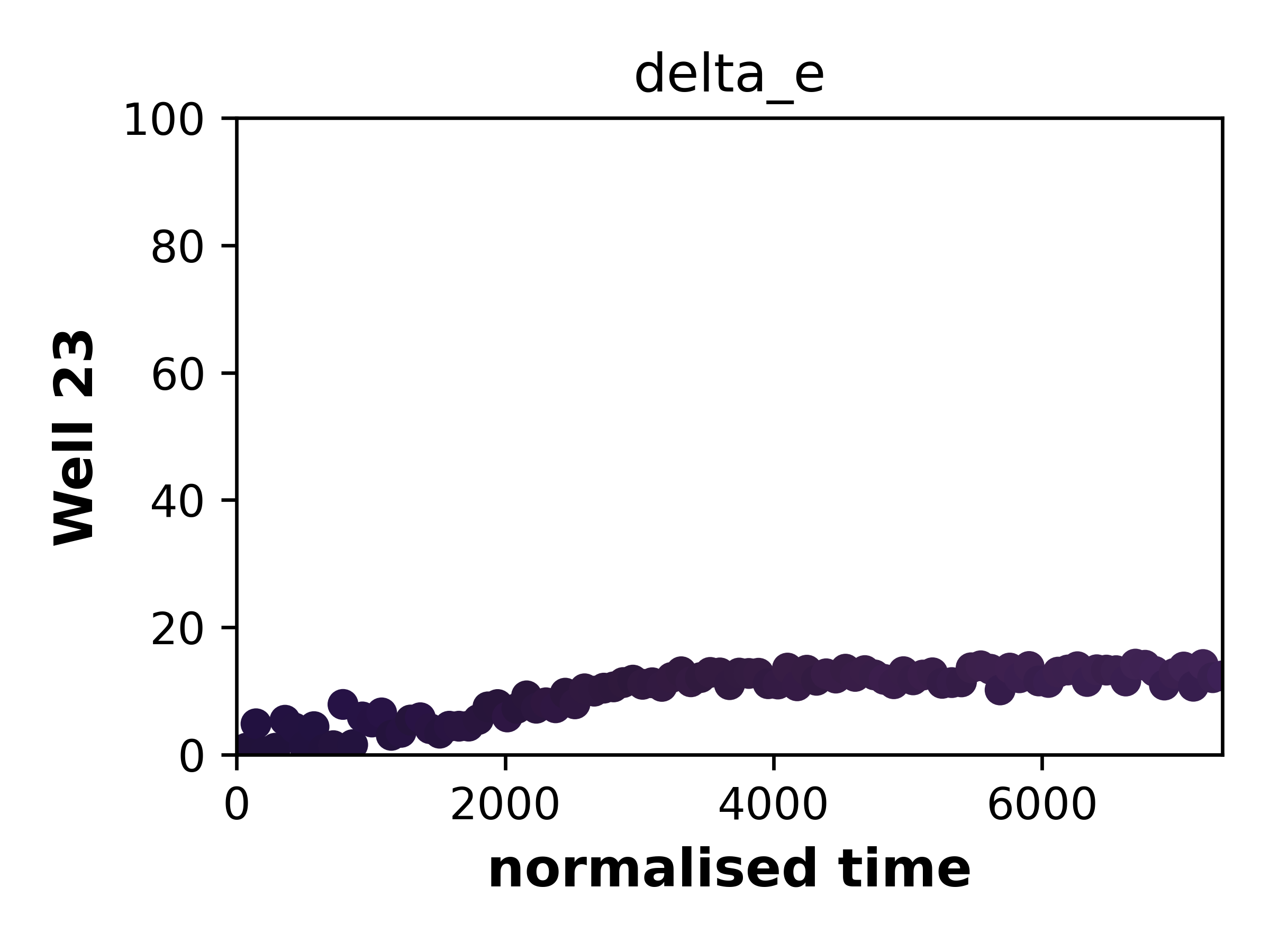

Supplement: Supplementary file 2 — Supporting Information [file ANIE-64-e202413395-s002.zip › Supporting Info - Machine readable data part 1/Figure 9 - crystal violet mixing analysis/Kineticolor outputs/temp/delta_e over normalised time - Well 23.png]

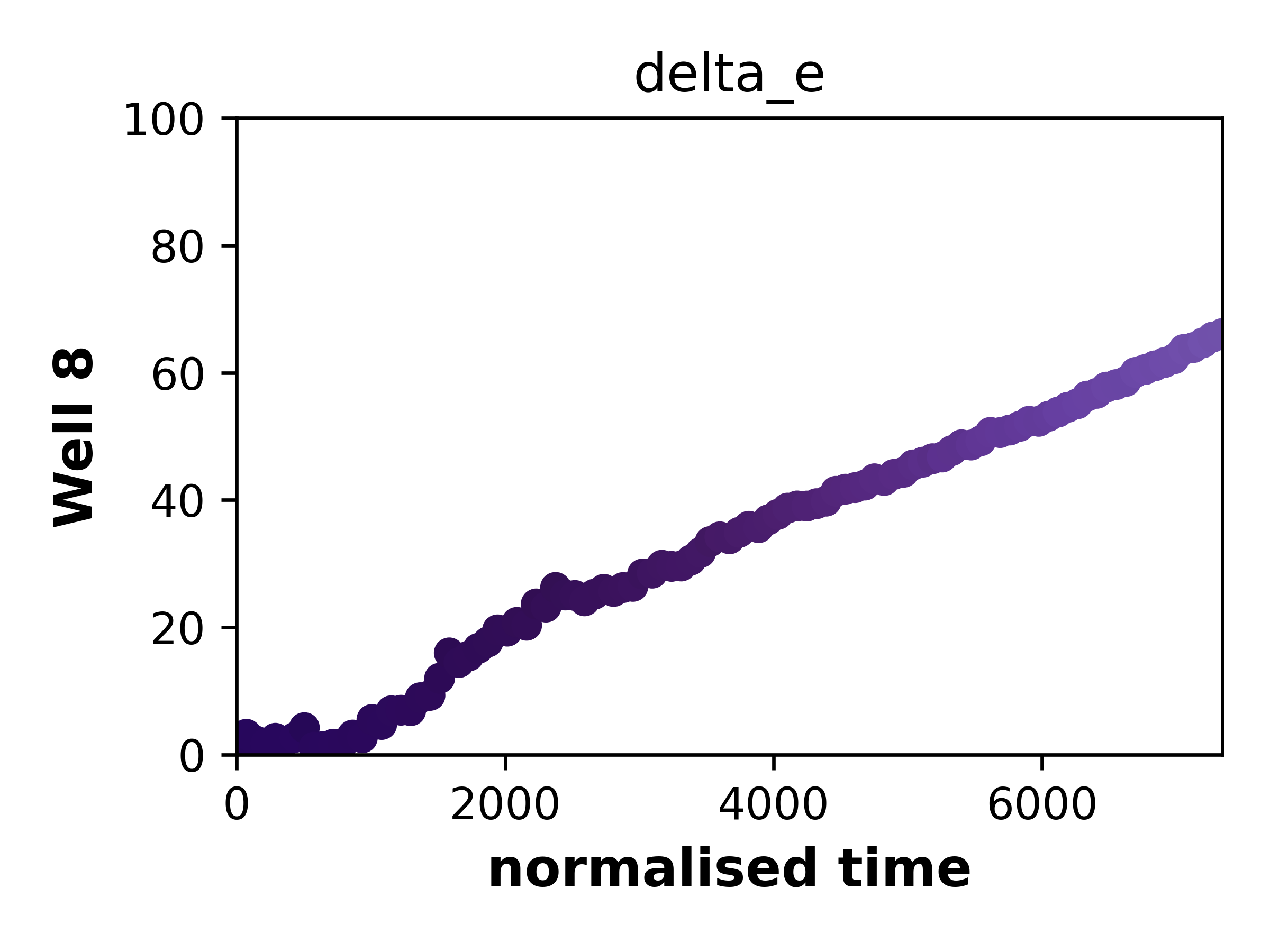

Supplement: Supplementary file 2 — Supporting Information [file ANIE-64-e202413395-s002.zip › Supporting Info - Machine readable data part 1/Figure 9 - crystal violet mixing analysis/Kineticolor outputs/temp/delta_e over normalised time - Well 8.png]

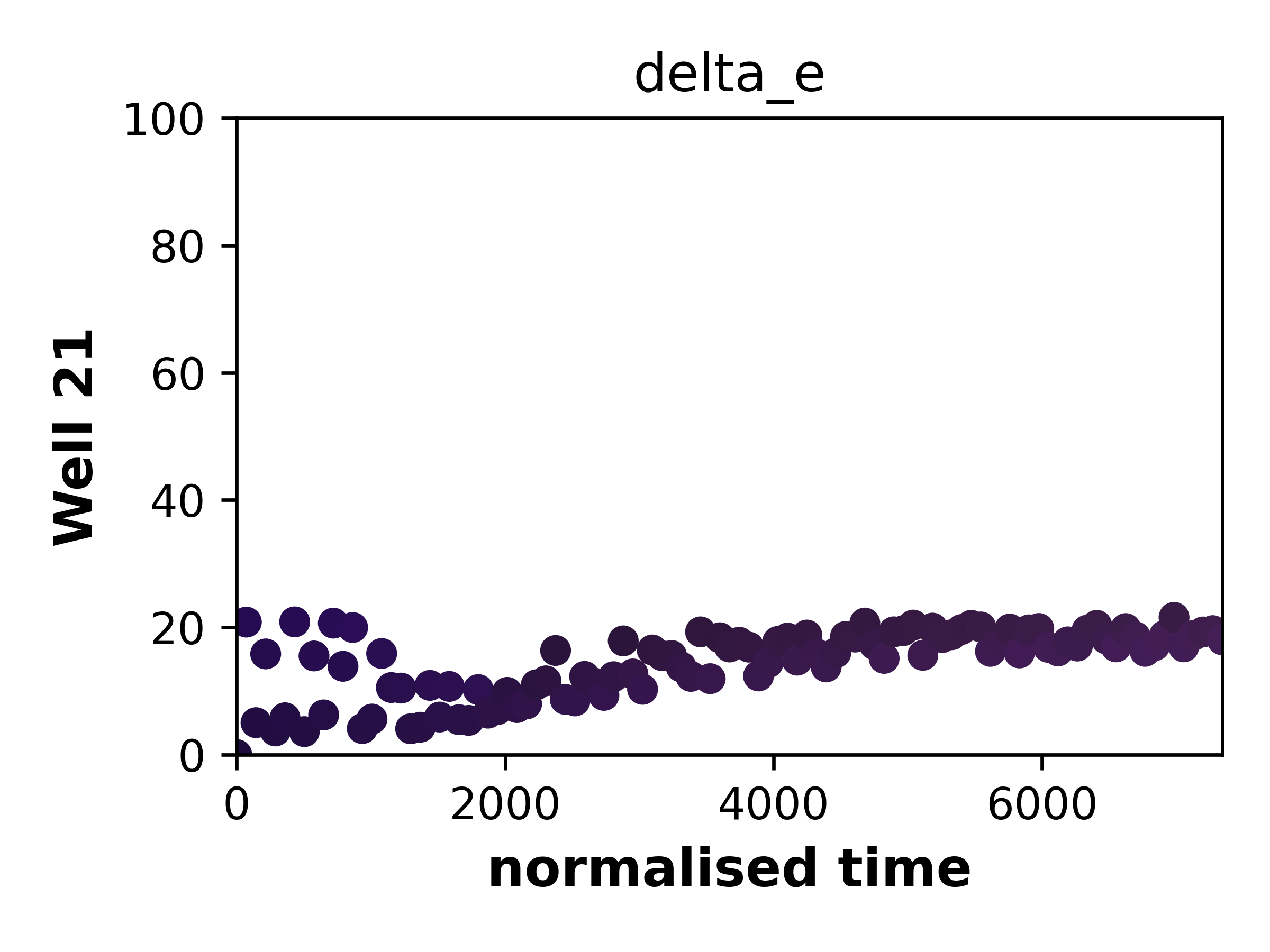

Supplement: Supplementary file 2 — Supporting Information [file ANIE-64-e202413395-s002.zip › Supporting Info - Machine readable data part 1/Figure 9 - crystal violet mixing analysis/Kineticolor outputs/temp/delta_e over normalised time - Well 21.png]

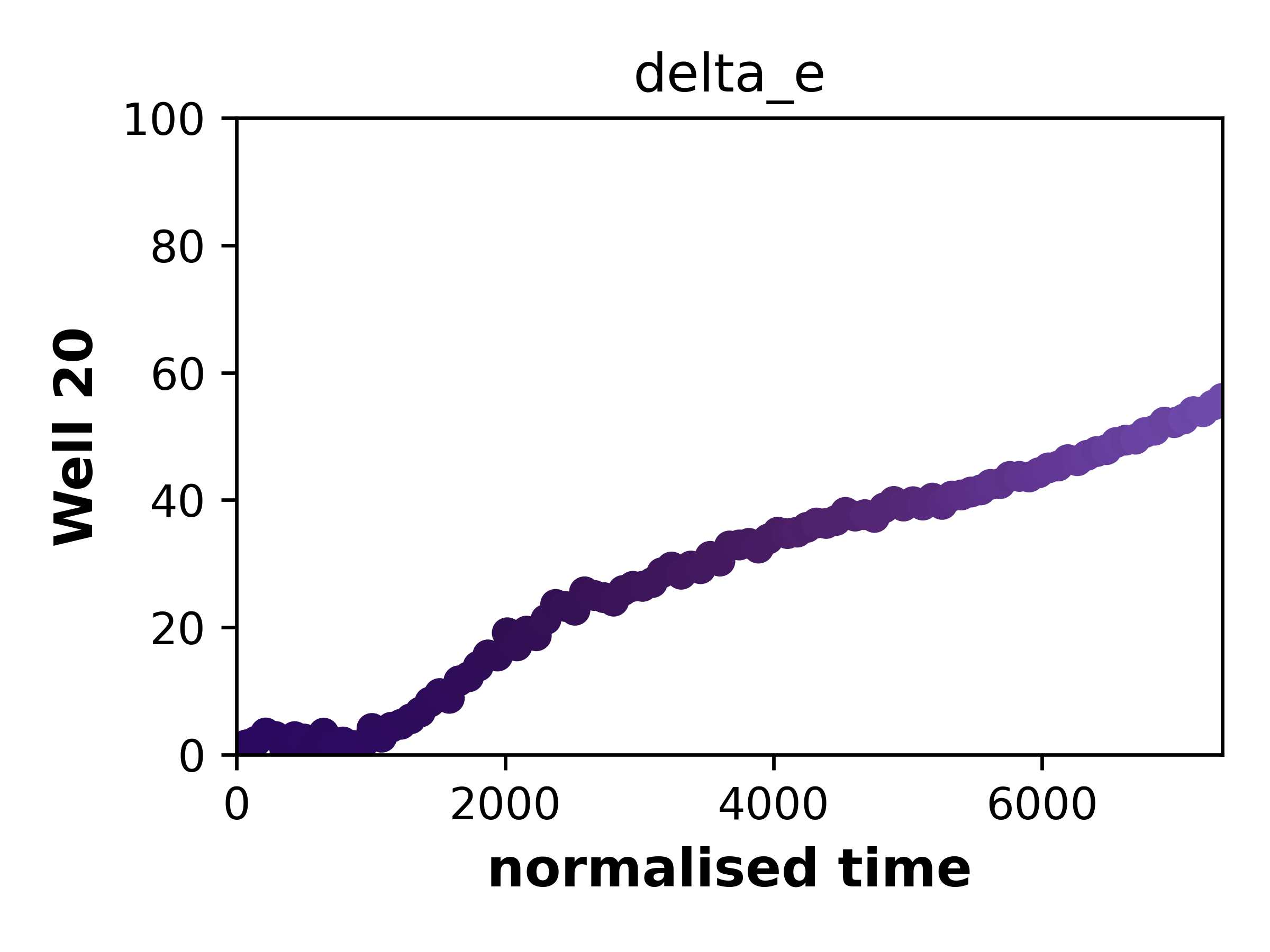

Supplement: Supplementary file 2 — Supporting Information [file ANIE-64-e202413395-s002.zip › Supporting Info - Machine readable data part 1/Figure 9 - crystal violet mixing analysis/Kineticolor outputs/temp/delta_e over normalised time - Well 20.png]

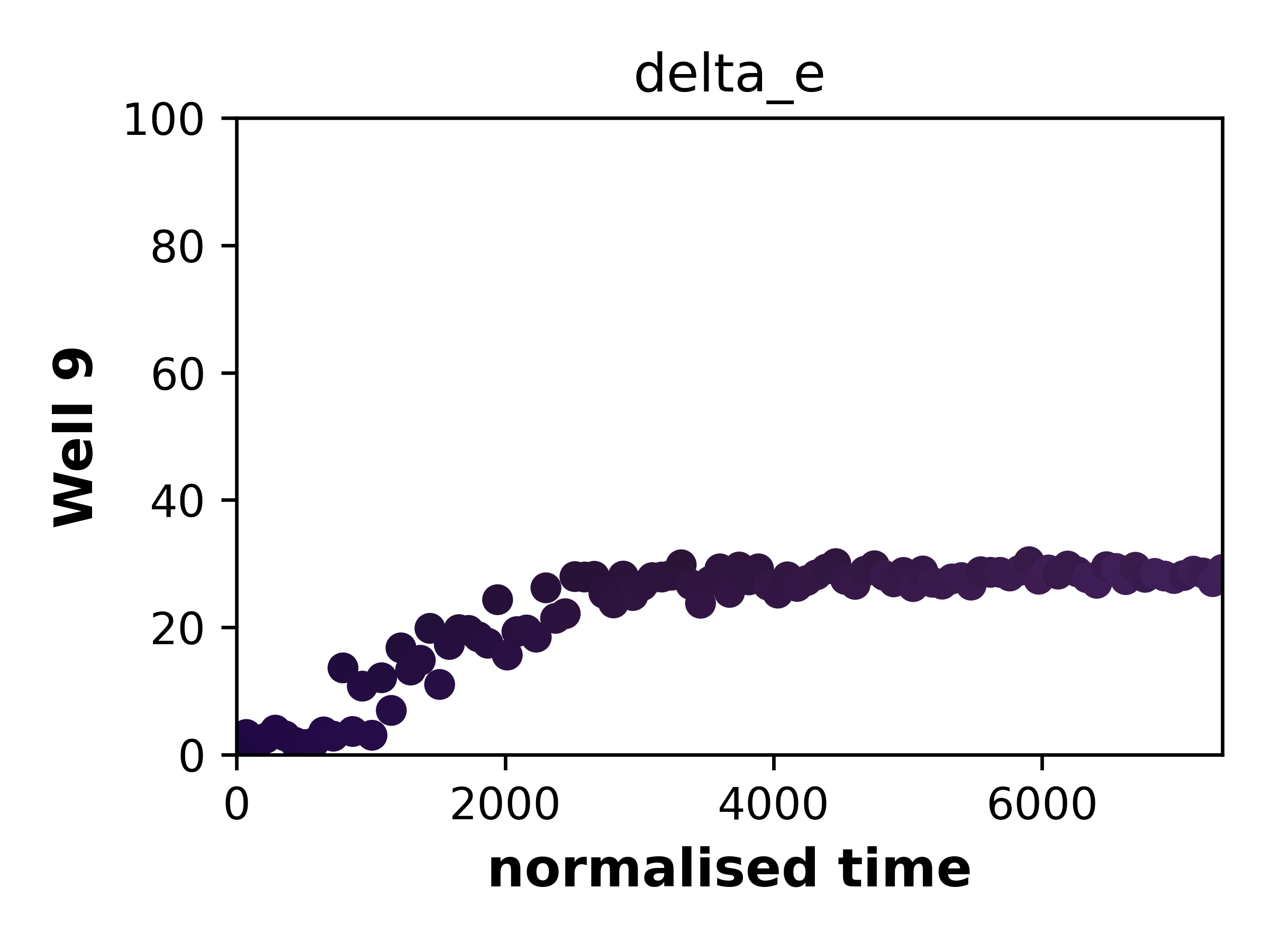

Supplement: Supplementary file 2 — Supporting Information [file ANIE-64-e202413395-s002.zip › Supporting Info - Machine readable data part 1/Figure 9 - crystal violet mixing analysis/Kineticolor outputs/temp/delta_e over normalised time - Well 9.png]

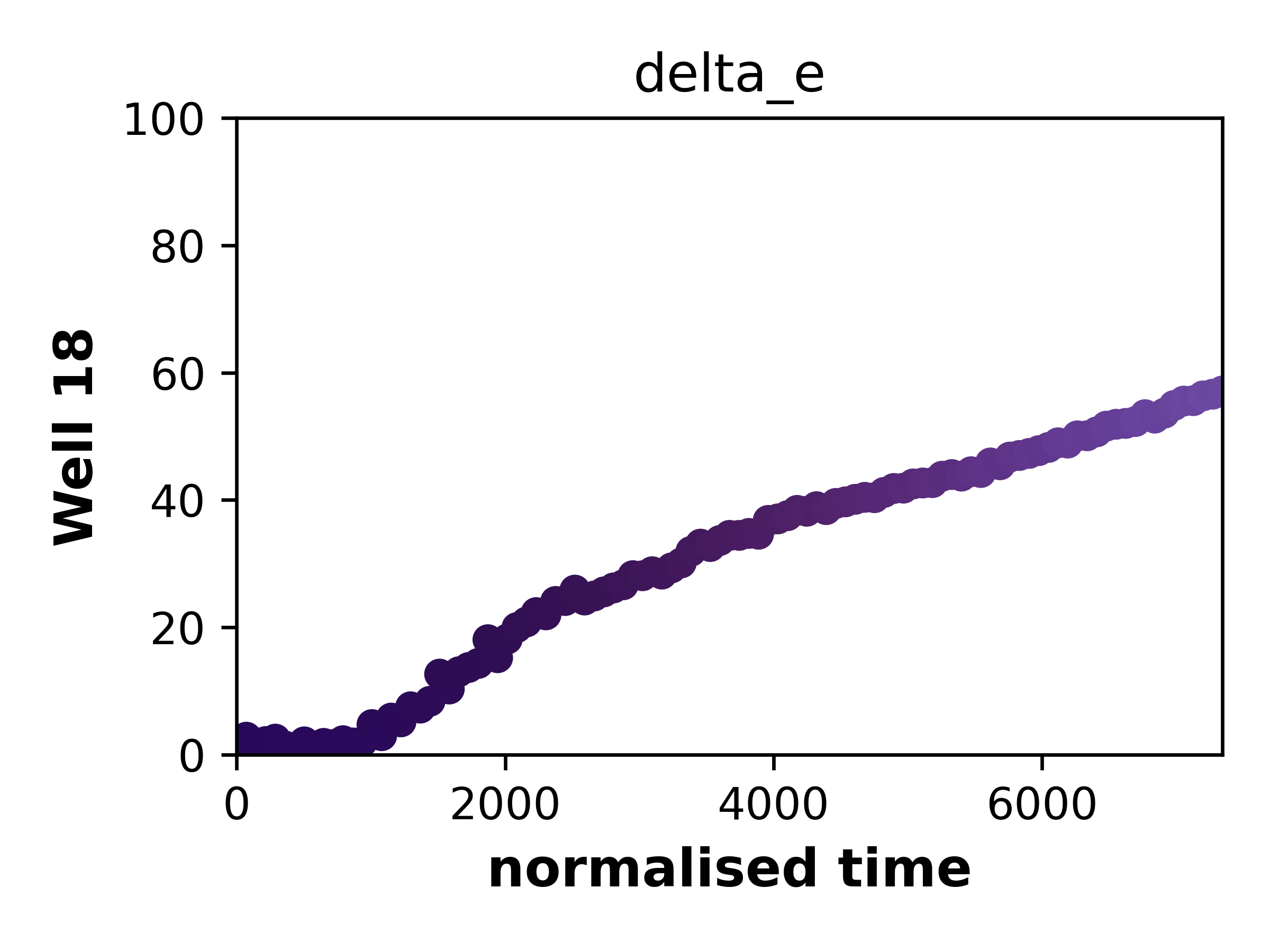

Supplement: Supplementary file 2 — Supporting Information [file ANIE-64-e202413395-s002.zip › Supporting Info - Machine readable data part 1/Figure 9 - crystal violet mixing analysis/Kineticolor outputs/temp/delta_e over normalised time - Well 18.png]

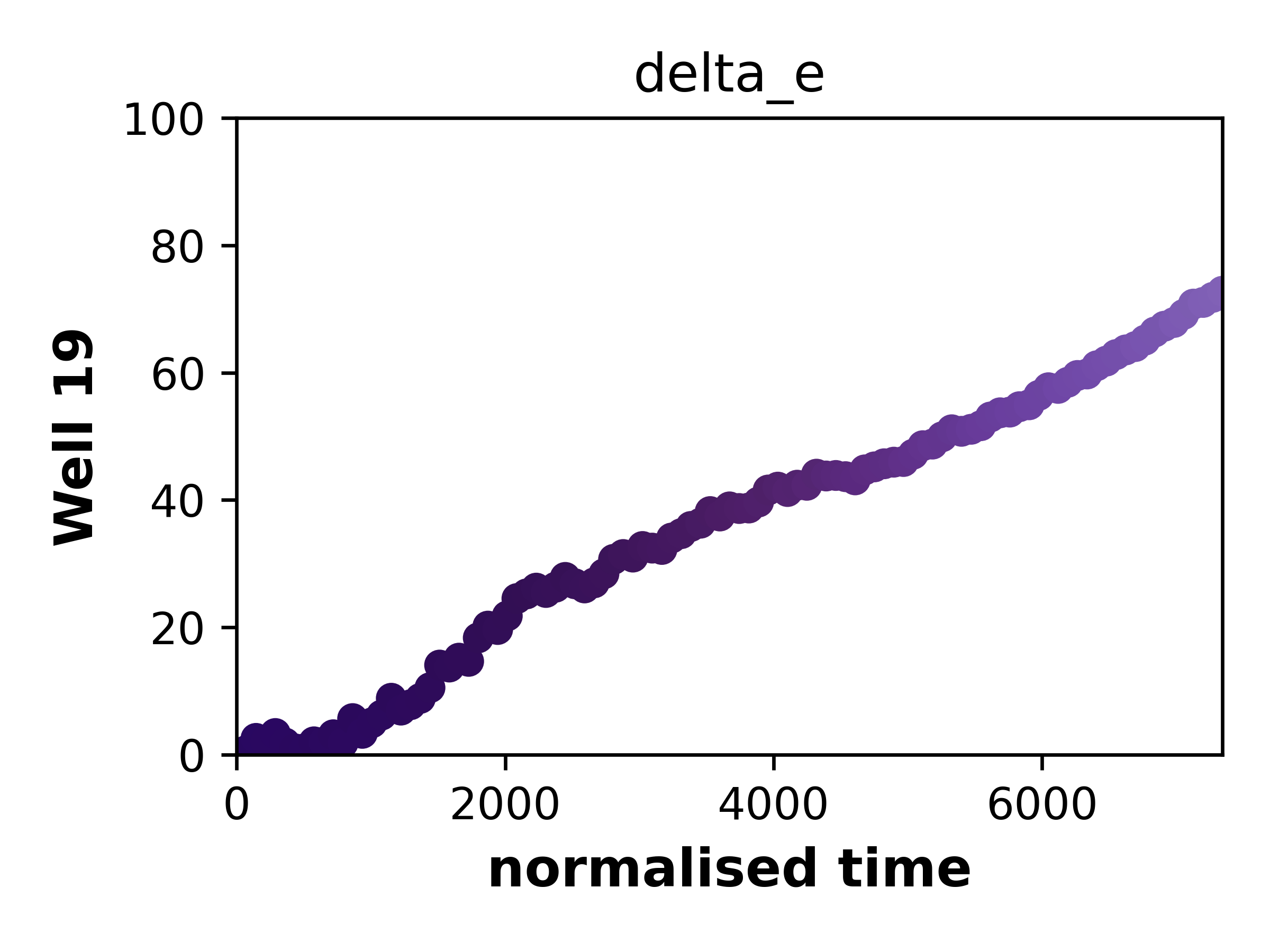

Supplement: Supplementary file 2 — Supporting Information [file ANIE-64-e202413395-s002.zip › Supporting Info - Machine readable data part 1/Figure 9 - crystal violet mixing analysis/Kineticolor outputs/temp/delta_e over normalised time - Well 19.png]

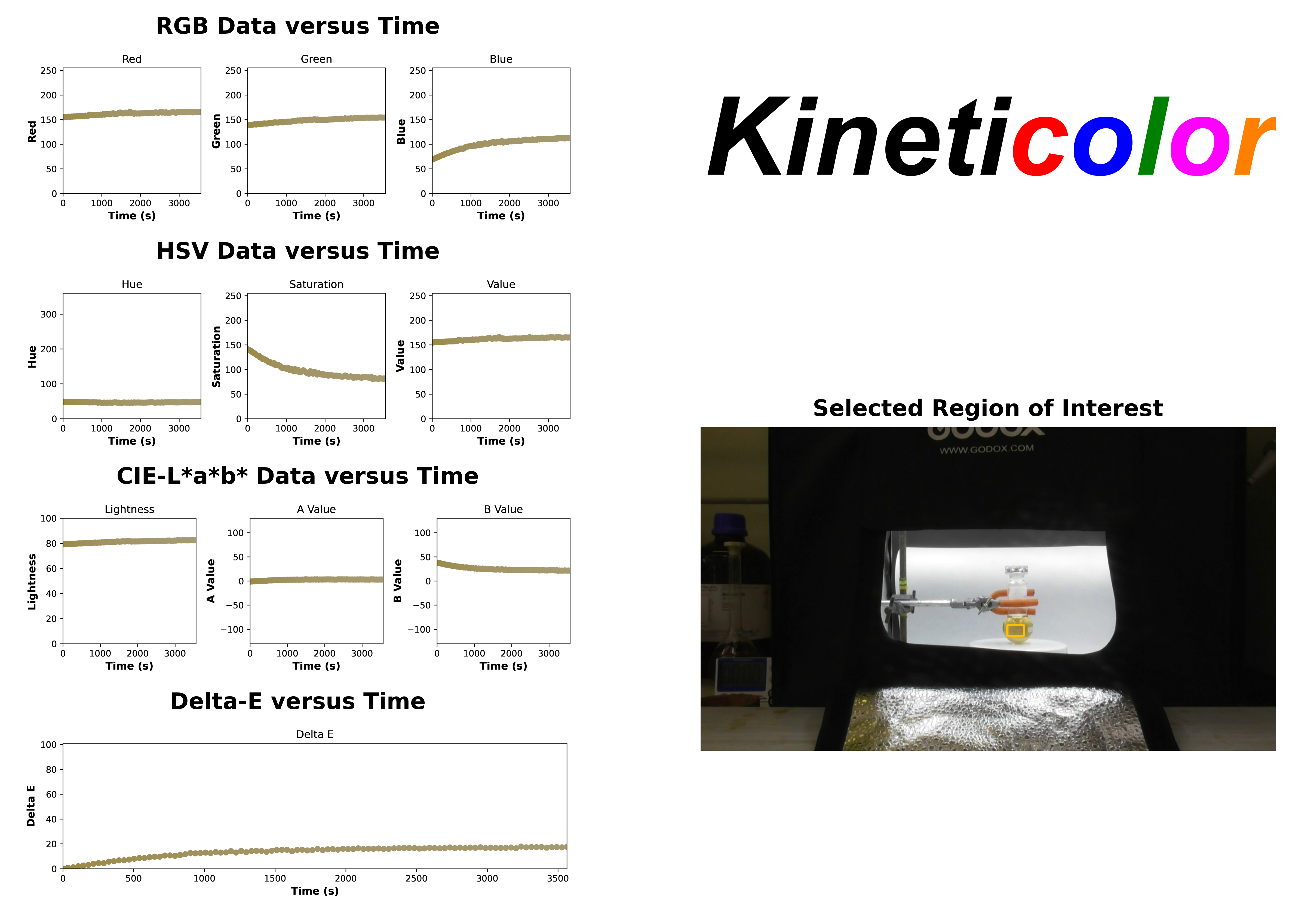

Supplement: Supplementary file 3 — Supporting Information [file ANIE-64-e202413395-s003.zip › Supporting Info - Machine readable data part 2/Figure 10 - esterification and mutual information/Kineticolor Kinetic study data/Summary 549XDPHH.PNG]

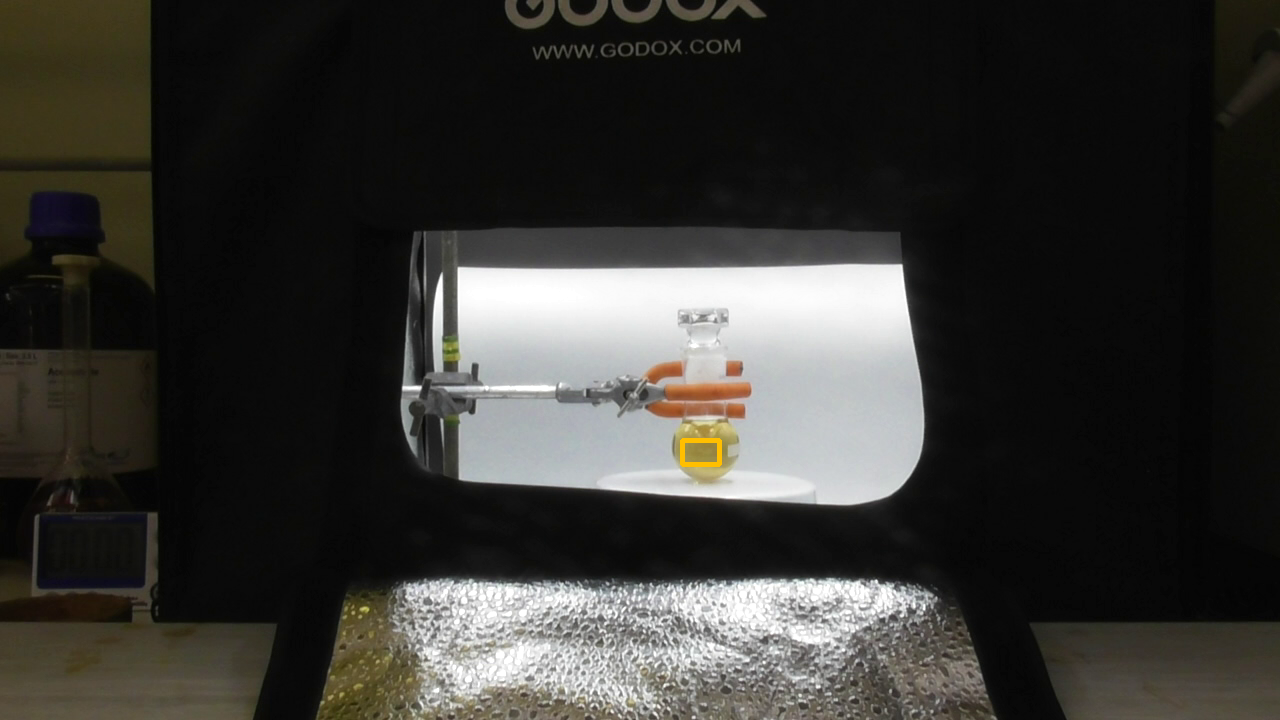

Supplement: Supplementary file 3 — Supporting Information [file ANIE-64-e202413395-s003.zip › Supporting Info - Machine readable data part 2/Figure 10 - esterification and mutual information/Kineticolor Kinetic study data/ROI.png]

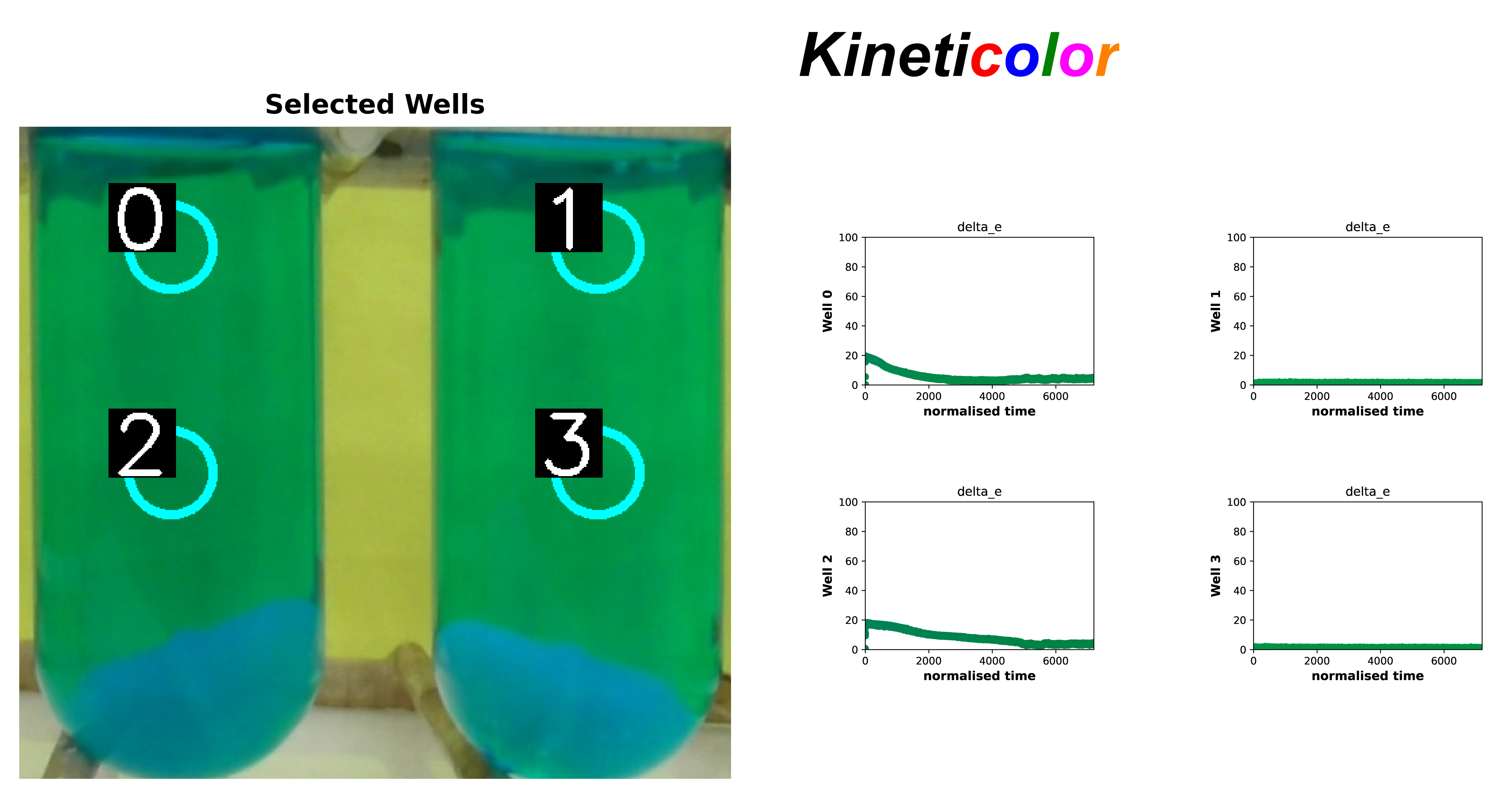

Supplement: Supplementary file 3 — Supporting Information [file ANIE-64-e202413395-s003.zip › Supporting Info - Machine readable data part 2/Figure 12 - Sedimentation in HTE/Kineticolor - Grade 1 vs Grade 3_/delta_e over normalised time Summary TM 14 - production sample 3 and supernatent comparison.PNG]

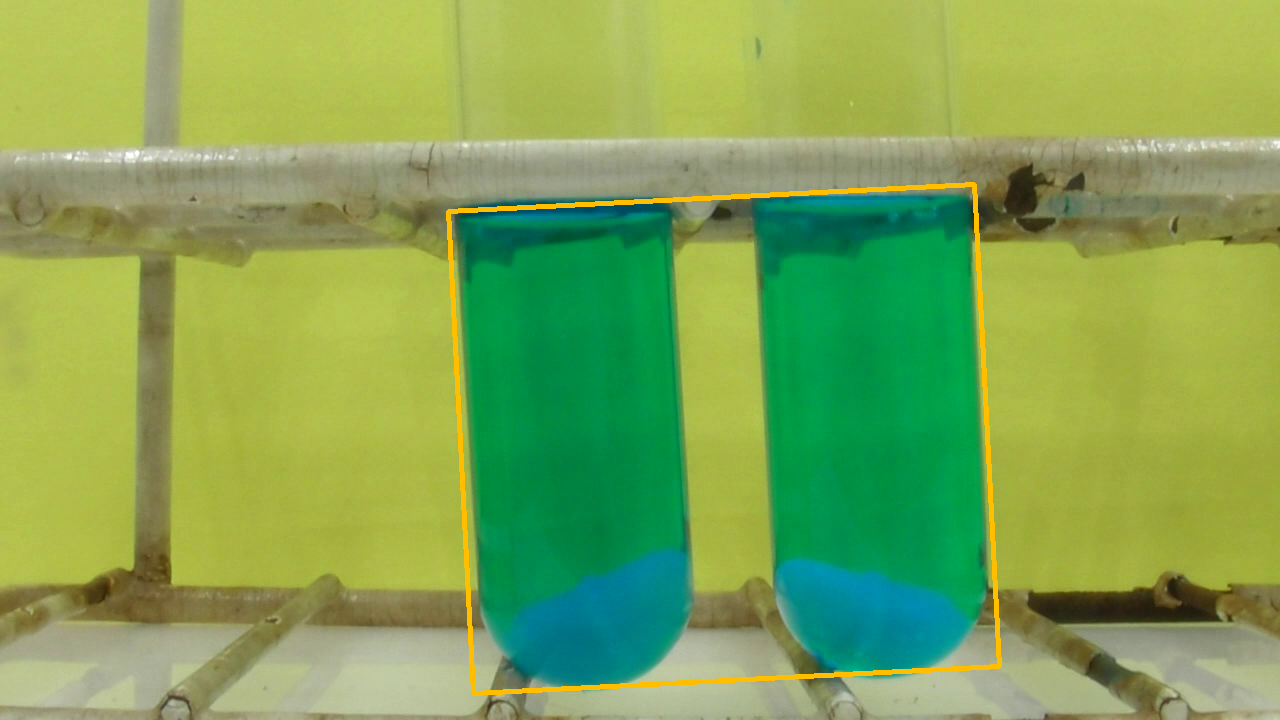

Supplement: Supplementary file 3 — Supporting Information [file ANIE-64-e202413395-s003.zip › Supporting Info - Machine readable data part 2/Figure 12 - Sedimentation in HTE/Kineticolor - Grade 1 vs Grade 3_/ROI.png]

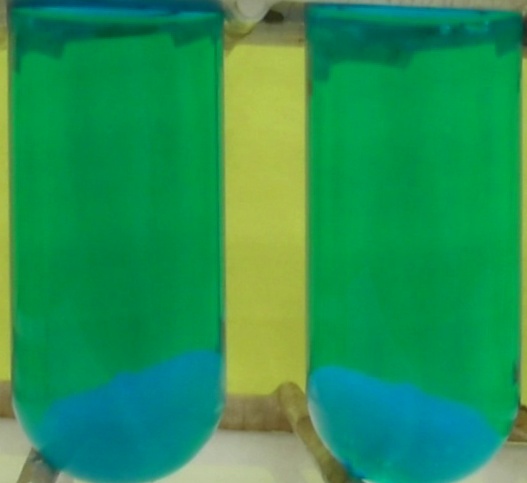

Supplement: Supplementary file 3 — Supporting Information [file ANIE-64-e202413395-s003.zip › Supporting Info - Machine readable data part 2/Figure 12 - Sedimentation in HTE/Kineticolor - Grade 1 vs Grade 3_/first_frame.jpg]

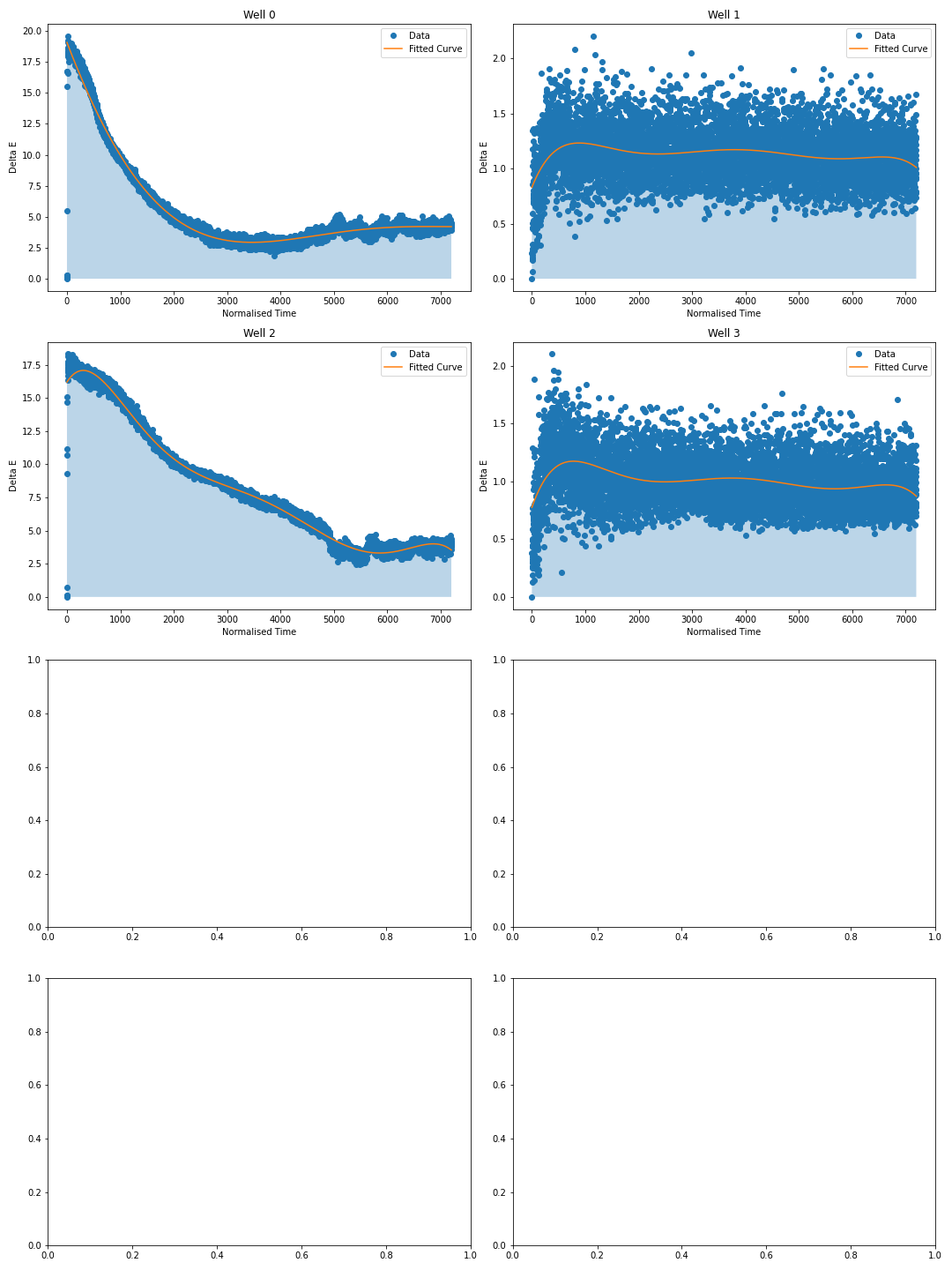

Supplement: Supplementary file 3 — Supporting Information [file ANIE-64-e202413395-s003.zip › Supporting Info - Machine readable data part 2/Figure 12 - Sedimentation in HTE/Kineticolor - Grade 1 vs Grade 3_/time_series_plots.png]

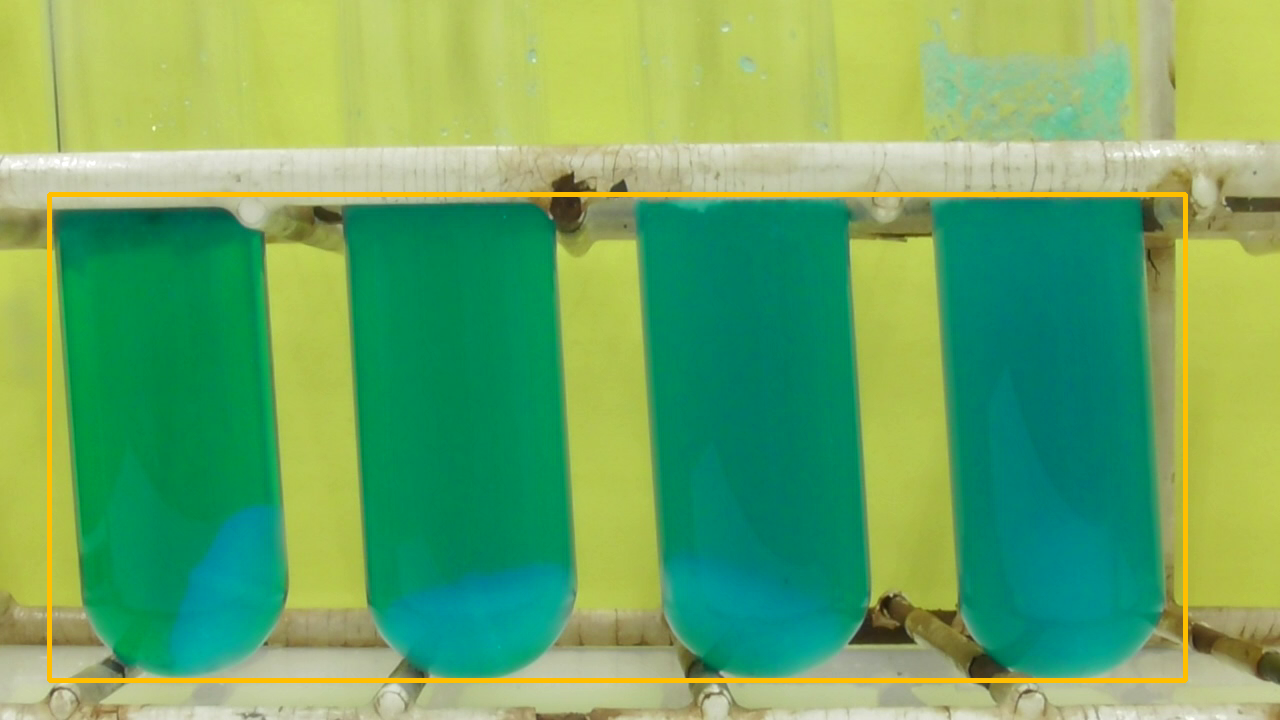

Supplement: Supplementary file 3 — Supporting Information [file ANIE-64-e202413395-s003.zip › Supporting Info - Machine readable data part 2/Figure 12 - Sedimentation in HTE/Kineticolor - perlite adulterated samples/ROI.png]

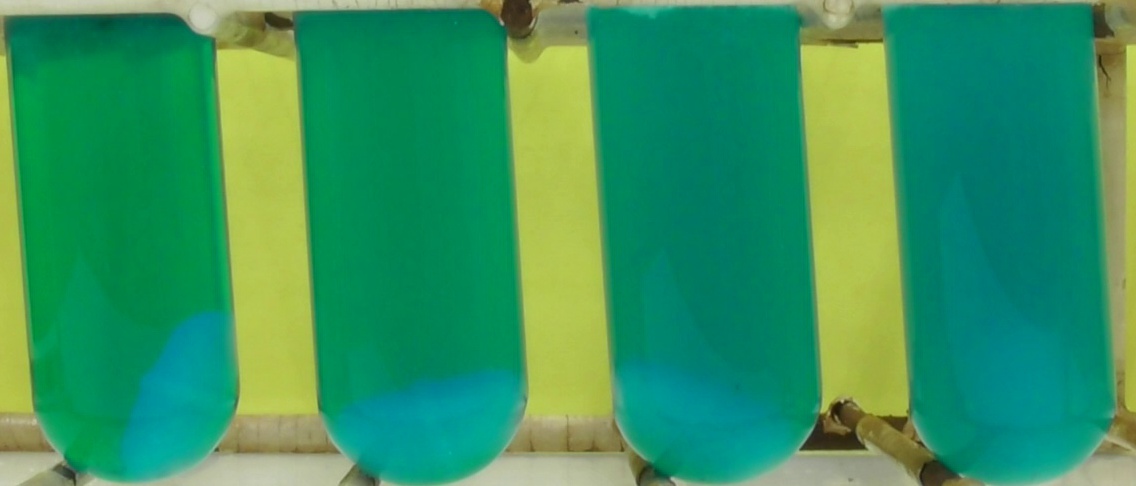

Supplement: Supplementary file 3 — Supporting Information [file ANIE-64-e202413395-s003.zip › Supporting Info - Machine readable data part 2/Figure 12 - Sedimentation in HTE/Kineticolor - perlite adulterated samples/first_frame.jpg]

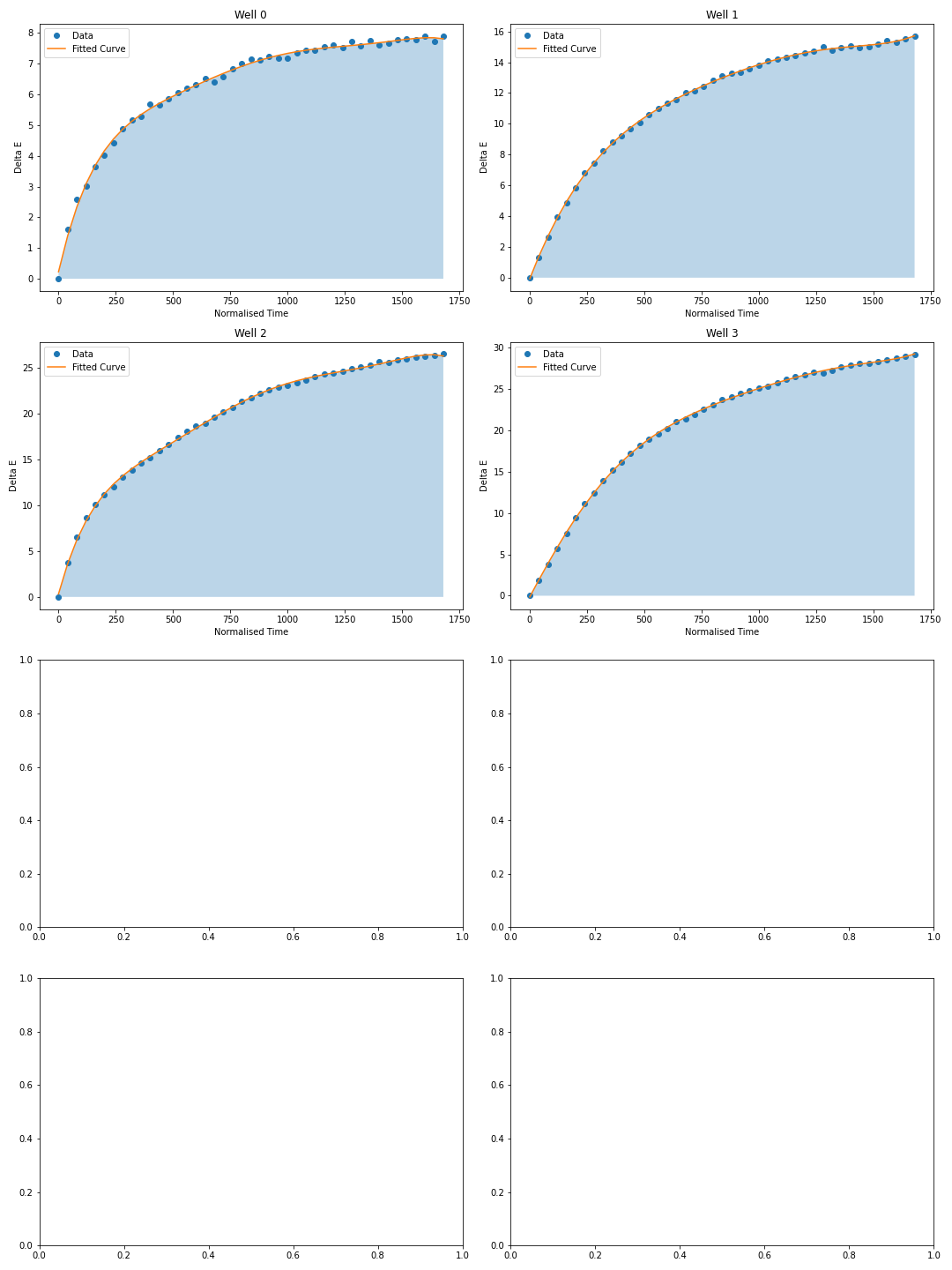

Supplement: Supplementary file 3 — Supporting Information [file ANIE-64-e202413395-s003.zip › Supporting Info - Machine readable data part 2/Figure 12 - Sedimentation in HTE/Kineticolor - perlite adulterated samples/time_series_plots.png]

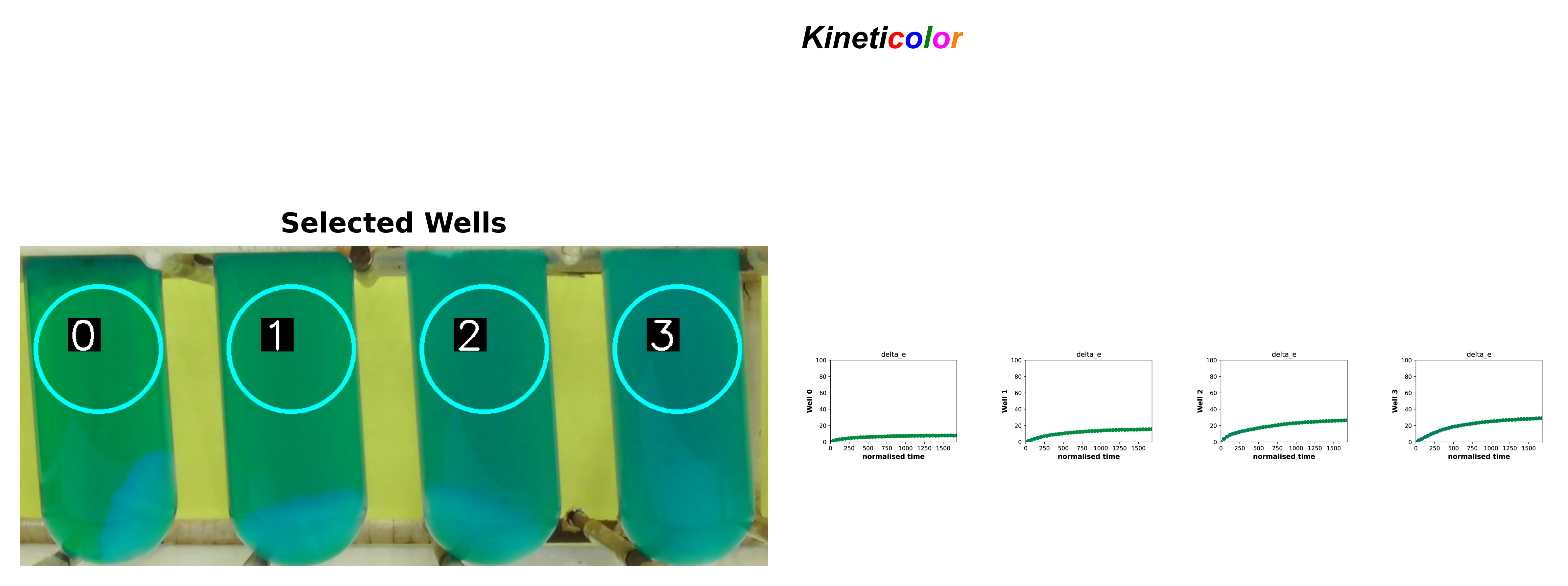

Supplement: Supplementary file 3 — Supporting Information [file ANIE-64-e202413395-s003.zip › Supporting Info - Machine readable data part 2/Figure 12 - Sedimentation in HTE/Kineticolor - perlite adulterated samples/delta_e over normalised time Summary S5600002.PNG]

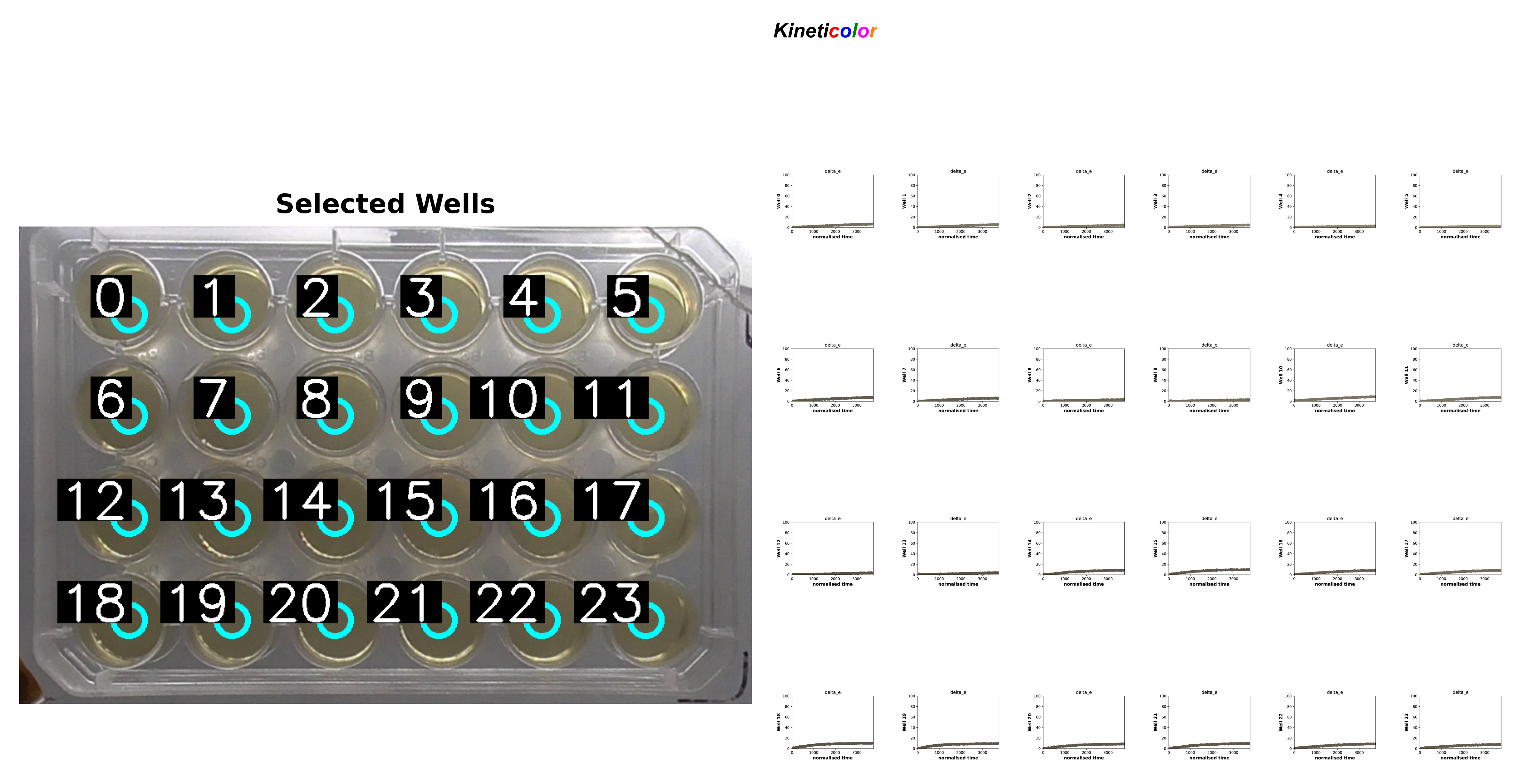

Supplement: Supplementary file 3 — Supporting Information [file ANIE-64-e202413395-s003.zip › Supporting Info - Machine readable data part 2/Table 2 - esterification in HTE/Kineticolor ouputs/delta_e over normalised time Summary Vari-PA+DMAP run 2.PNG]

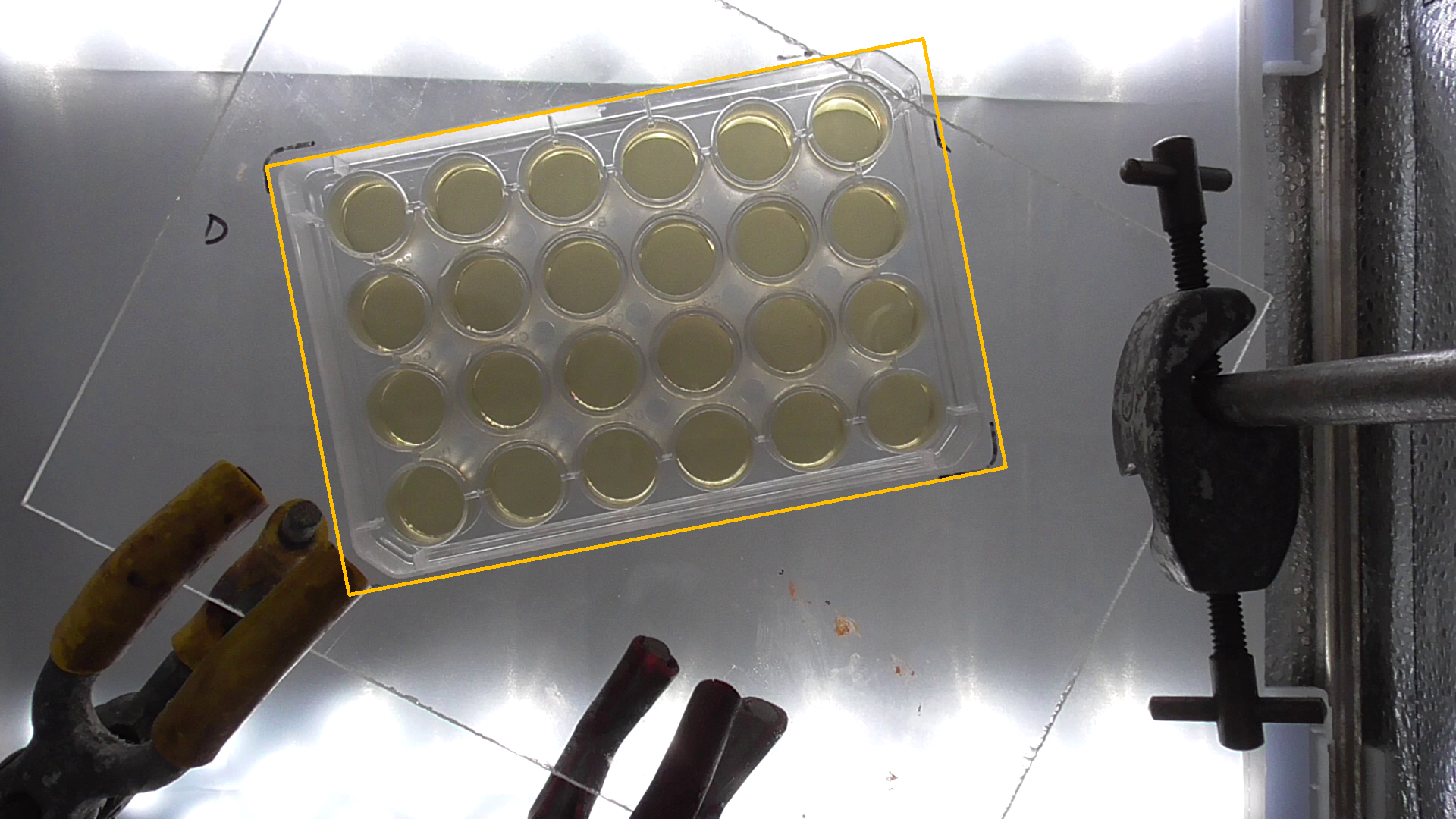

Supplement: Supplementary file 3 — Supporting Information [file ANIE-64-e202413395-s003.zip › Supporting Info - Machine readable data part 2/Table 2 - esterification in HTE/Kineticolor ouputs/ROI.png]

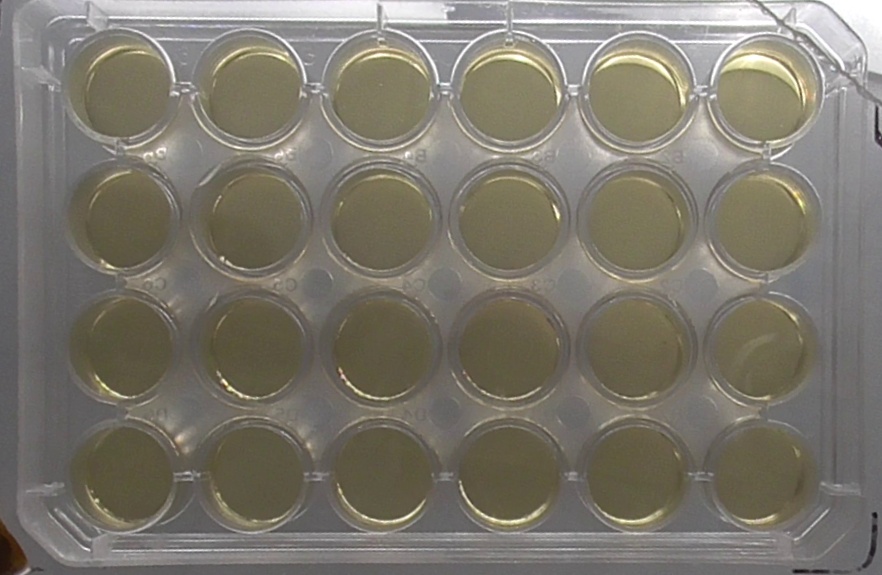

Supplement: Supplementary file 3 — Supporting Information [file ANIE-64-e202413395-s003.zip › Supporting Info - Machine readable data part 2/Table 2 - esterification in HTE/Kineticolor ouputs/first_frame.jpg]

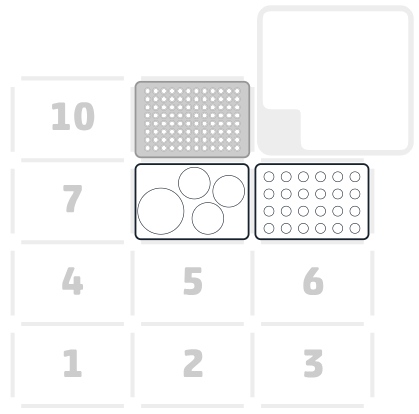

Supplement: Supplementary file 3 — Supporting Information [file ANIE-64-e202413395-s003.zip › Supporting Info - Machine readable data part 2/Table 2 - esterification in HTE/OT-2 Liquid Handling Robot Files/Esterification protocol layout.png]

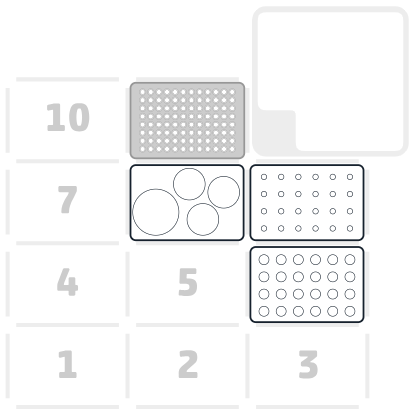

Supplement: Supplementary file 3 — Supporting Information [file ANIE-64-e202413395-s003.zip › Supporting Info - Machine readable data part 2/Table 2 - esterification in HTE/OT-2 Liquid Handling Robot Files/HPLC prep protocol layout.png]

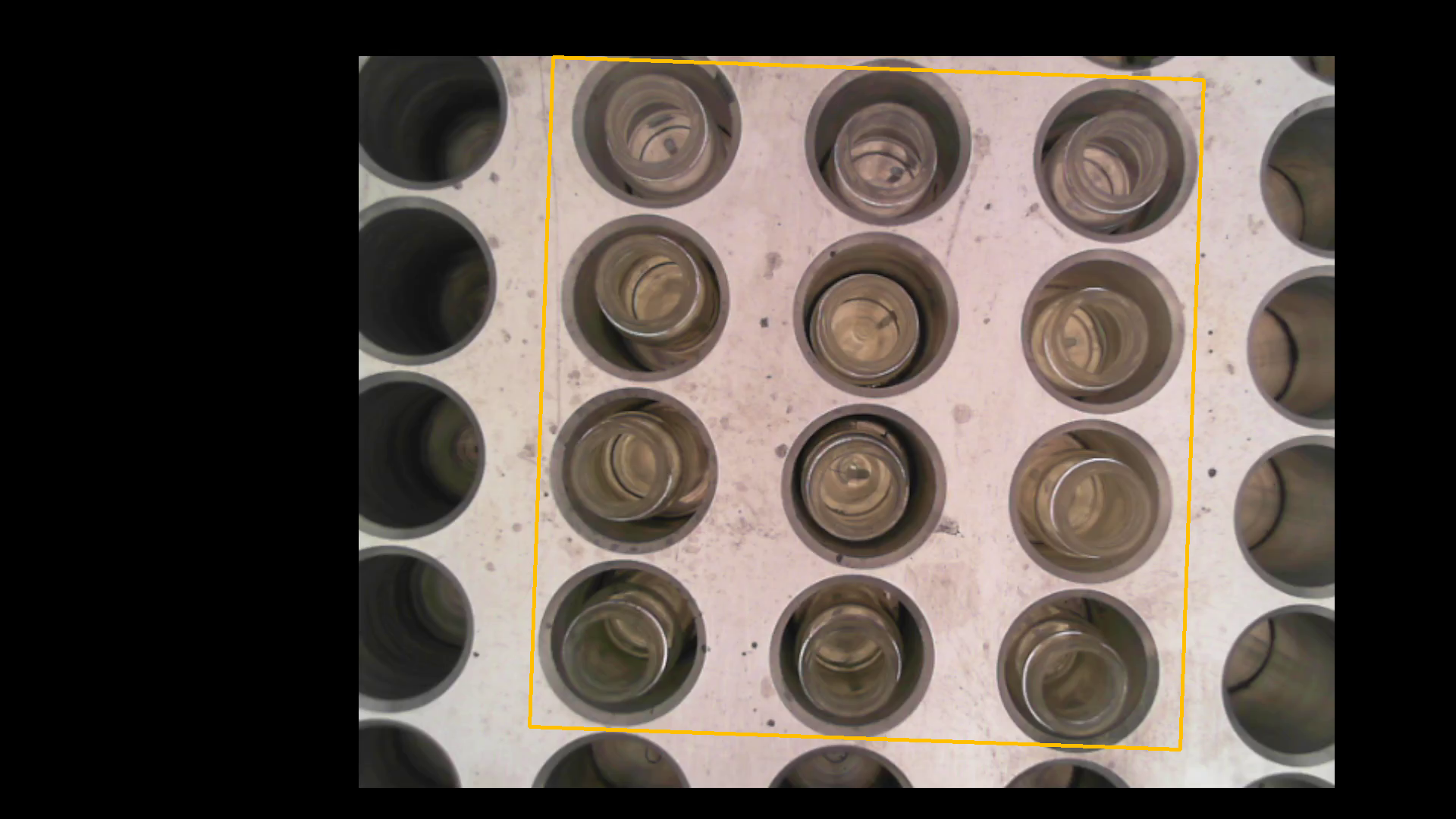

Supplement: Supplementary file 3 — Supporting Information [file ANIE-64-e202413395-s003.zip › Supporting Info - Machine readable data part 2/Figure 11 - Pd catalyst degradation in HTE/Kineticolor data/ROI.png]

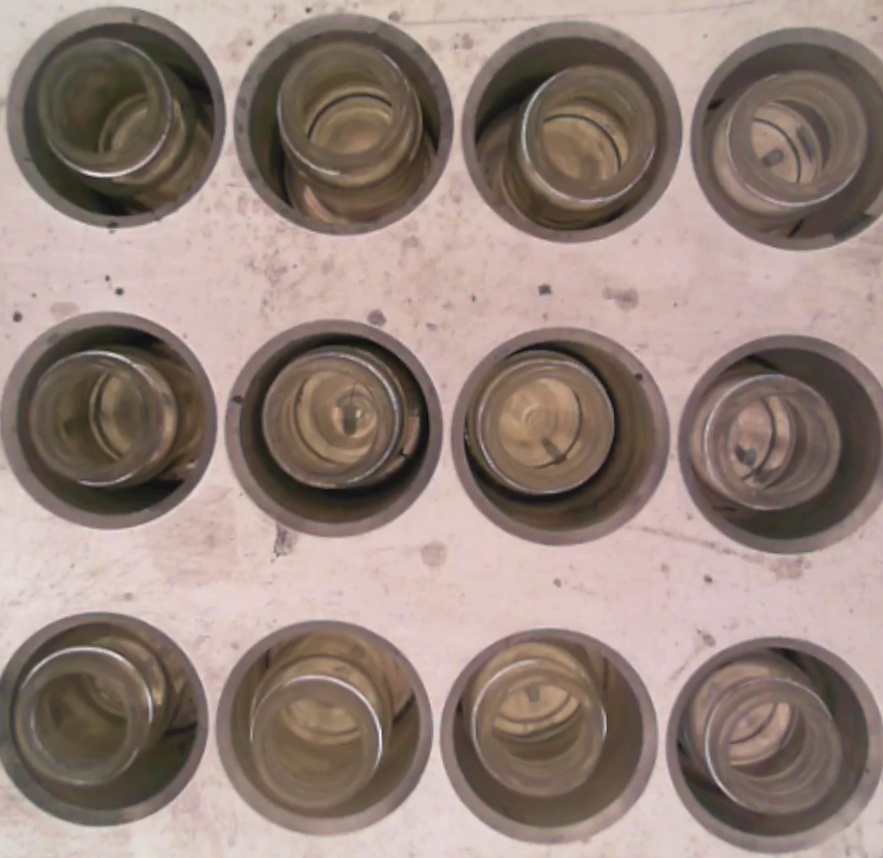

Supplement: Supplementary file 3 — Supporting Information [file ANIE-64-e202413395-s003.zip › Supporting Info - Machine readable data part 2/Figure 11 - Pd catalyst degradation in HTE/Kineticolor data/first_frame.jpg]

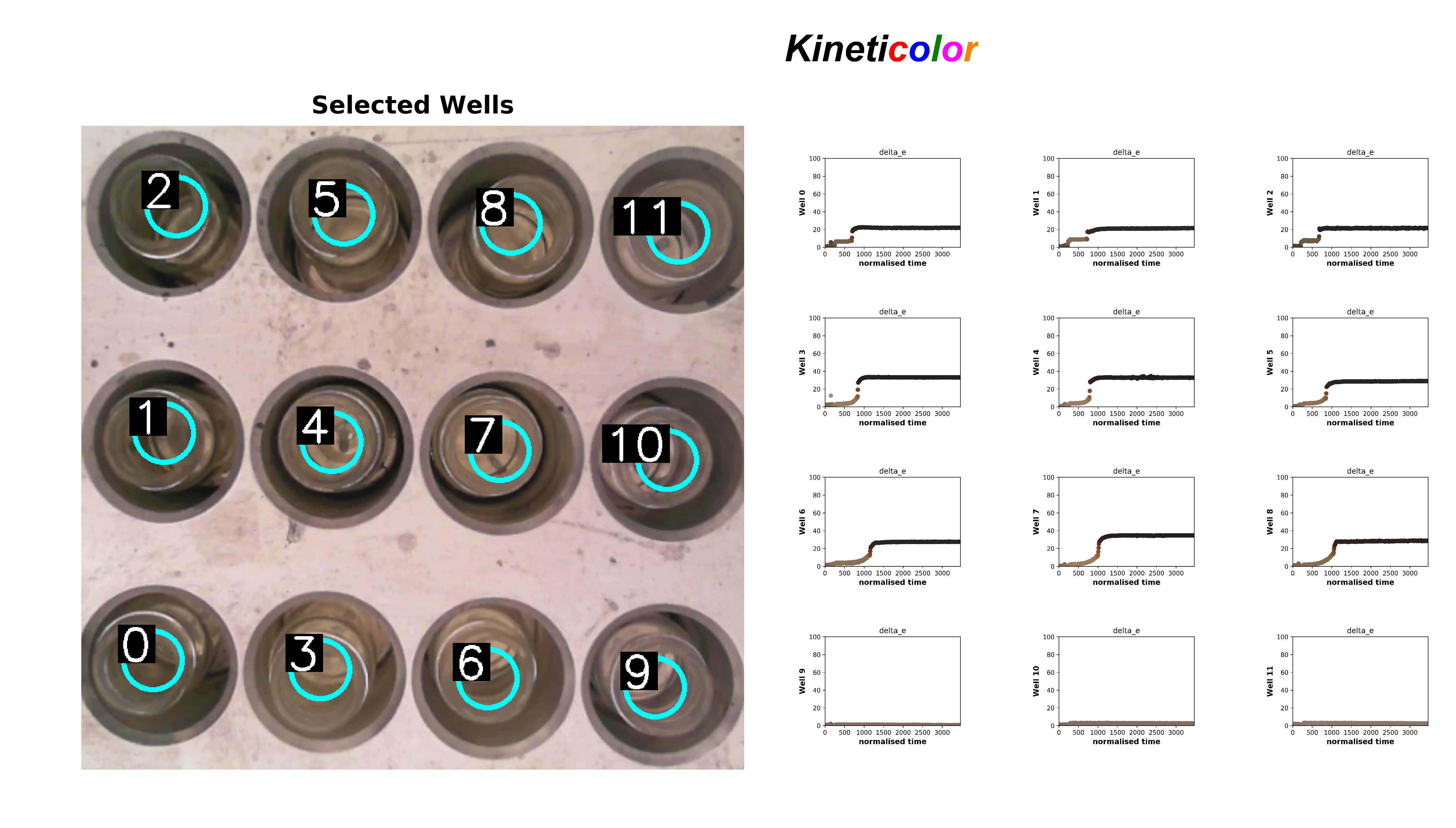

Supplement: Supplementary file 3 — Supporting Information [file ANIE-64-e202413395-s003.zip › Supporting Info - Machine readable data part 2/Figure 11 - Pd catalyst degradation in HTE/Kineticolor data/delta_e over normalised time Summary 2021-08-23 13-47-42 multiwell stirrer palladium bistricyclohexylphosphine diacetate boropinocol reaction in toluene.PNG]

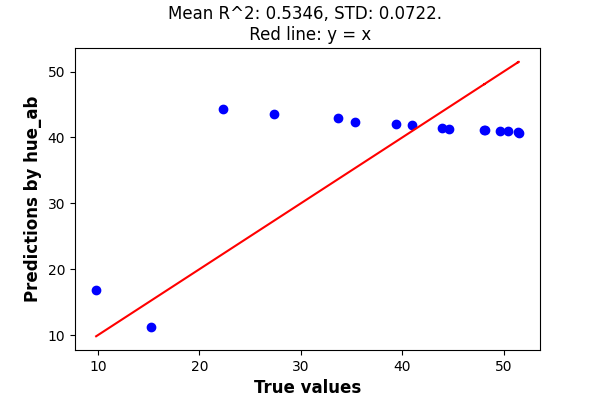

Supplement: Supplementary file 3 — Supporting Information [file ANIE-64-e202413395-s003.zip › Supporting Info - Machine readable data part 2/Figure 10 - esterification and mutual information/Mutual Information and Regression outputs/Cross validation charts/CV_LOO_hue_ab.png]

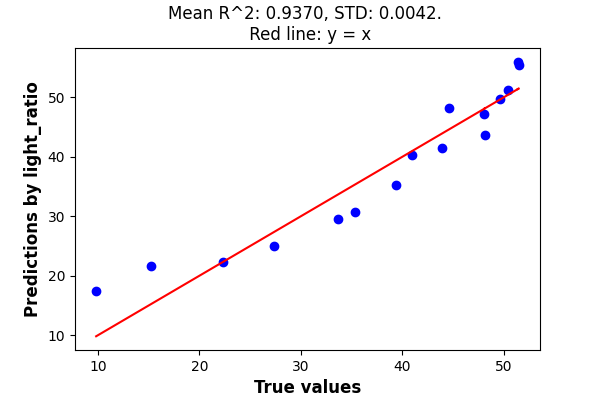

Supplement: Supplementary file 3 — Supporting Information [file ANIE-64-e202413395-s003.zip › Supporting Info - Machine readable data part 2/Figure 10 - esterification and mutual information/Mutual Information and Regression outputs/Cross validation charts/CV_LOO_light_ratio.png]

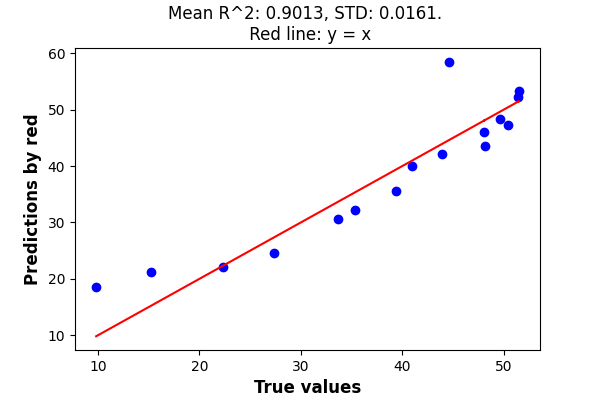

Supplement: Supplementary file 3 — Supporting Information [file ANIE-64-e202413395-s003.zip › Supporting Info - Machine readable data part 2/Figure 10 - esterification and mutual information/Mutual Information and Regression outputs/Cross validation charts/CV_LOO_red.png]

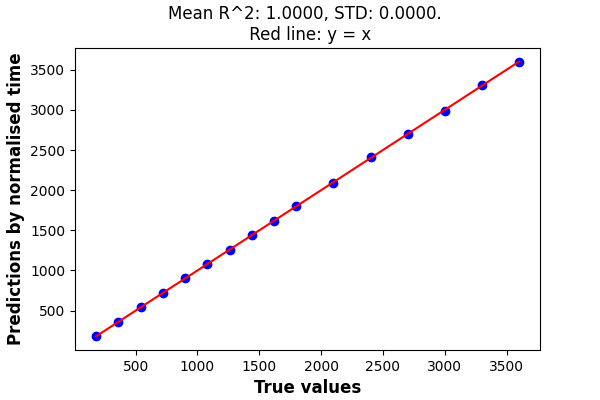

Supplement: Supplementary file 3 — Supporting Information [file ANIE-64-e202413395-s003.zip › Supporting Info - Machine readable data part 2/Figure 10 - esterification and mutual information/Mutual Information and Regression outputs/Cross validation charts/CV_LOO_normalised time.png]

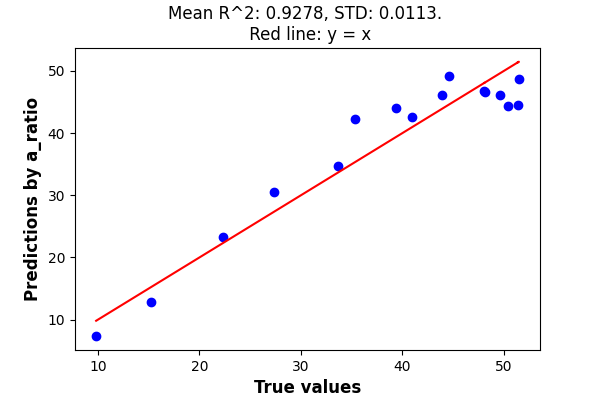

Supplement: Supplementary file 3 — Supporting Information [file ANIE-64-e202413395-s003.zip › Supporting Info - Machine readable data part 2/Figure 10 - esterification and mutual information/Mutual Information and Regression outputs/Cross validation charts/CV_LOO_a_ratio.png]

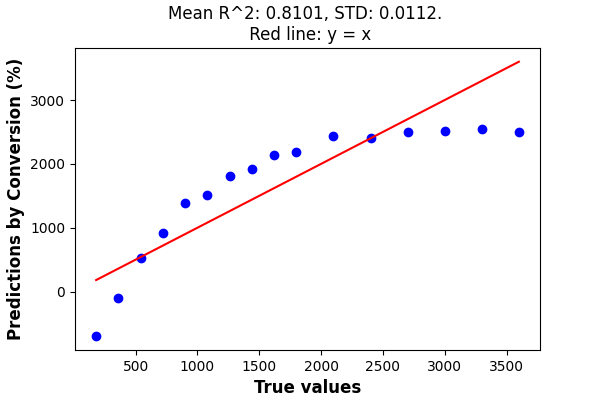

Supplement: Supplementary file 3 — Supporting Information [file ANIE-64-e202413395-s003.zip › Supporting Info - Machine readable data part 2/Figure 10 - esterification and mutual information/Mutual Information and Regression outputs/Cross validation charts/CV_LOO_Conversion (%).png]

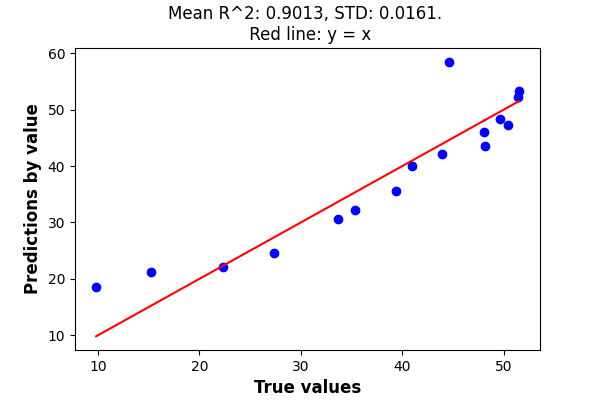

Supplement: Supplementary file 3 — Supporting Information [file ANIE-64-e202413395-s003.zip › Supporting Info - Machine readable data part 2/Figure 10 - esterification and mutual information/Mutual Information and Regression outputs/Cross validation charts/CV_LOO_value.png]

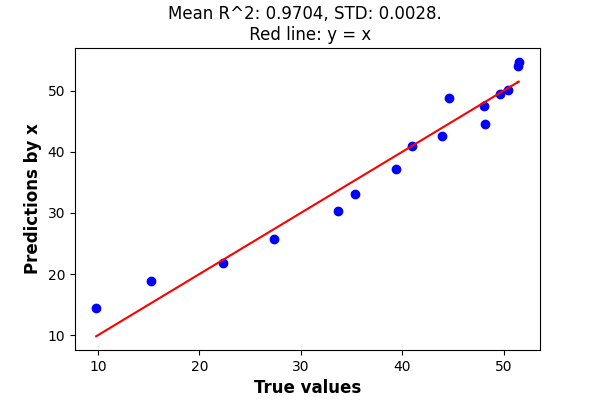

Supplement: Supplementary file 3 — Supporting Information [file ANIE-64-e202413395-s003.zip › Supporting Info - Machine readable data part 2/Figure 10 - esterification and mutual information/Mutual Information and Regression outputs/Cross validation charts/CV_LOO_x.png]

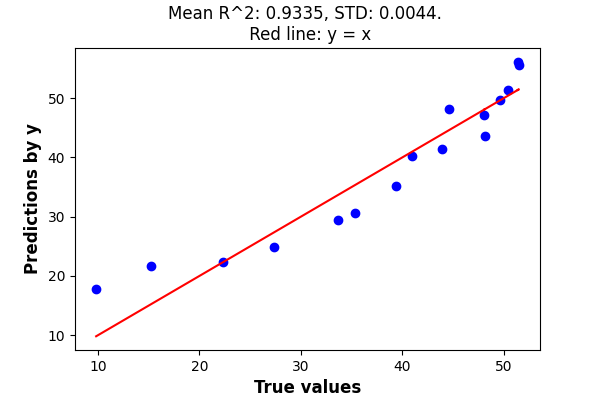

Supplement: Supplementary file 3 — Supporting Information [file ANIE-64-e202413395-s003.zip › Supporting Info - Machine readable data part 2/Figure 10 - esterification and mutual information/Mutual Information and Regression outputs/Cross validation charts/CV_LOO_y.png]

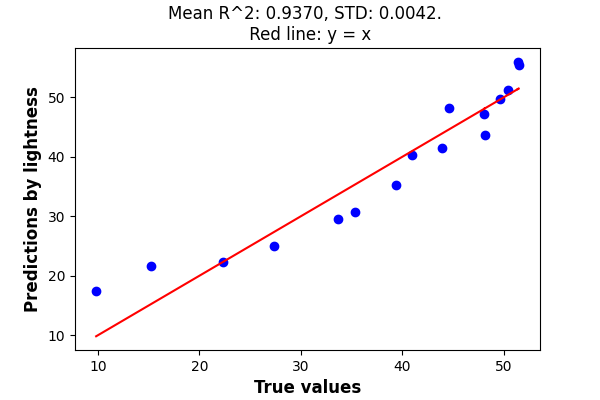

Supplement: Supplementary file 3 — Supporting Information [file ANIE-64-e202413395-s003.zip › Supporting Info - Machine readable data part 2/Figure 10 - esterification and mutual information/Mutual Information and Regression outputs/Cross validation charts/CV_LOO_lightness.png]

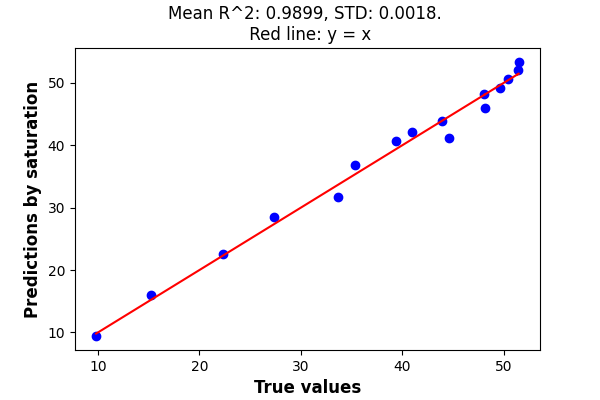

Supplement: Supplementary file 3 — Supporting Information [file ANIE-64-e202413395-s003.zip › Supporting Info - Machine readable data part 2/Figure 10 - esterification and mutual information/Mutual Information and Regression outputs/Cross validation charts/CV_LOO_saturation.png]

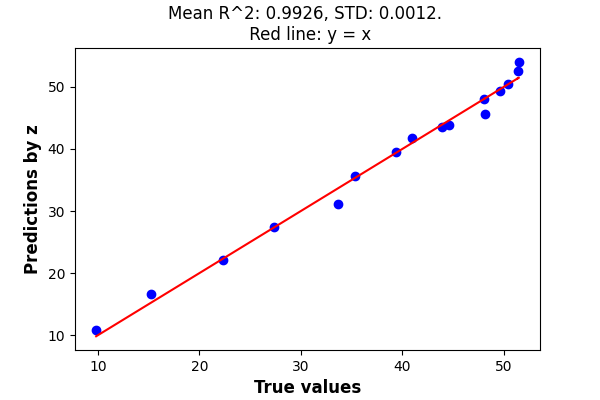

Supplement: Supplementary file 3 — Supporting Information [file ANIE-64-e202413395-s003.zip › Supporting Info - Machine readable data part 2/Figure 10 - esterification and mutual information/Mutual Information and Regression outputs/Cross validation charts/CV_LOO_z.png]

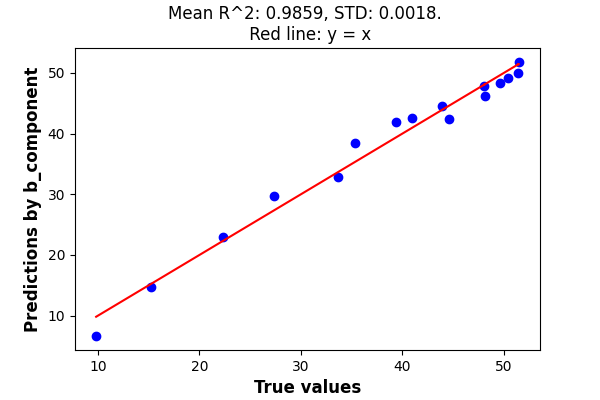

Supplement: Supplementary file 3 — Supporting Information [file ANIE-64-e202413395-s003.zip › Supporting Info - Machine readable data part 2/Figure 10 - esterification and mutual information/Mutual Information and Regression outputs/Cross validation charts/CV_LOO_b_component.png]

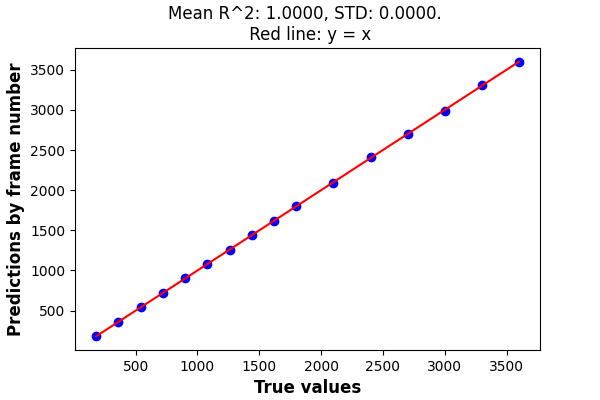

Supplement: Supplementary file 3 — Supporting Information [file ANIE-64-e202413395-s003.zip › Supporting Info - Machine readable data part 2/Figure 10 - esterification and mutual information/Mutual Information and Regression outputs/Cross validation charts/CV_LOO_frame number.png]

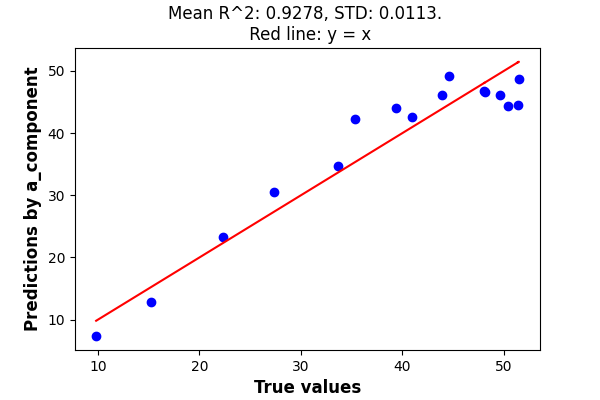

Supplement: Supplementary file 3 — Supporting Information [file ANIE-64-e202413395-s003.zip › Supporting Info - Machine readable data part 2/Figure 10 - esterification and mutual information/Mutual Information and Regression outputs/Cross validation charts/CV_LOO_a_component.png]

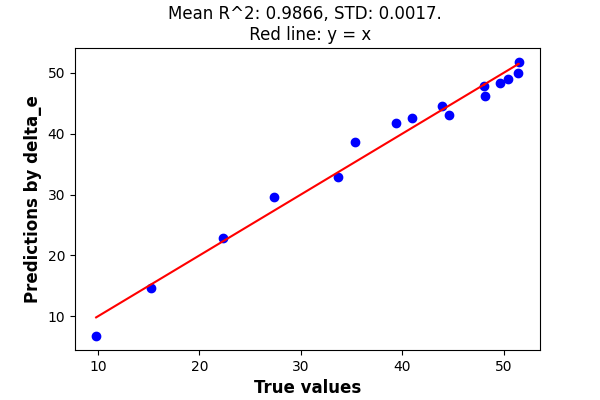

Supplement: Supplementary file 3 — Supporting Information [file ANIE-64-e202413395-s003.zip › Supporting Info - Machine readable data part 2/Figure 10 - esterification and mutual information/Mutual Information and Regression outputs/Cross validation charts/CV_LOO_delta_e.png]

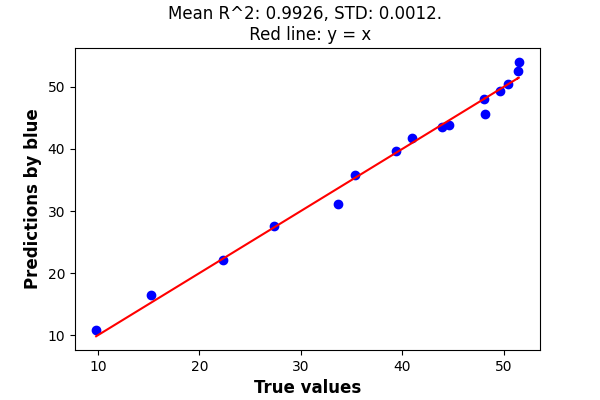

Supplement: Supplementary file 3 — Supporting Information [file ANIE-64-e202413395-s003.zip › Supporting Info - Machine readable data part 2/Figure 10 - esterification and mutual information/Mutual Information and Regression outputs/Cross validation charts/CV_LOO_blue.png]

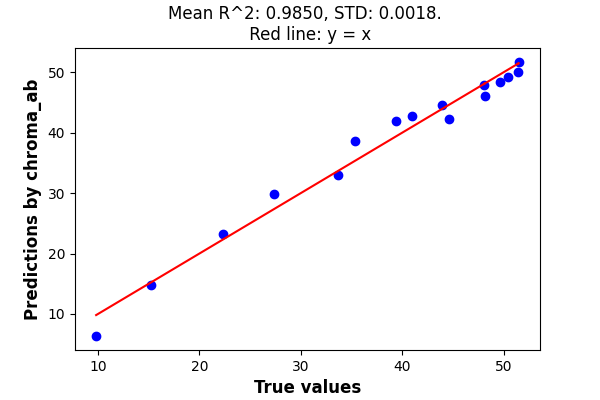

Supplement: Supplementary file 3 — Supporting Information [file ANIE-64-e202413395-s003.zip › Supporting Info - Machine readable data part 2/Figure 10 - esterification and mutual information/Mutual Information and Regression outputs/Cross validation charts/CV_LOO_chroma_ab.png]

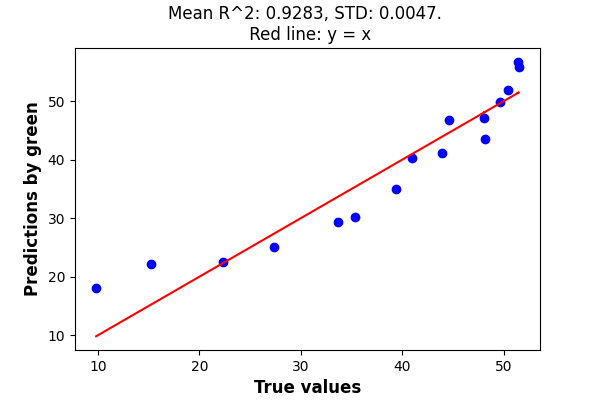

Supplement: Supplementary file 3 — Supporting Information [file ANIE-64-e202413395-s003.zip › Supporting Info - Machine readable data part 2/Figure 10 - esterification and mutual information/Mutual Information and Regression outputs/Cross validation charts/CV_LOO_green.png]

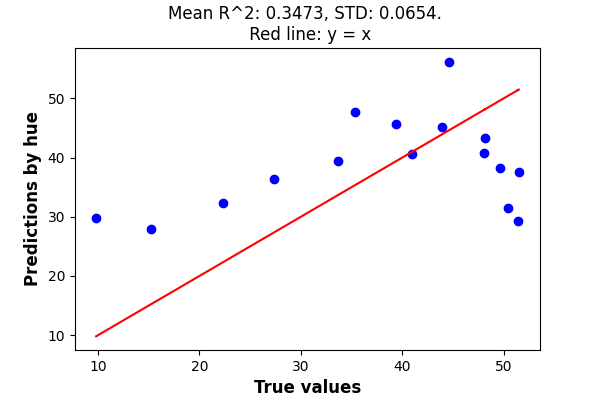

Supplement: Supplementary file 3 — Supporting Information [file ANIE-64-e202413395-s003.zip › Supporting Info - Machine readable data part 2/Figure 10 - esterification and mutual information/Mutual Information and Regression outputs/Cross validation charts/CV_LOO_hue.png]

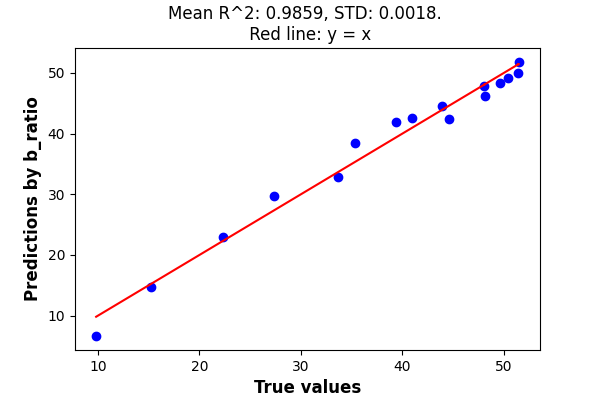

Supplement: Supplementary file 3 — Supporting Information [file ANIE-64-e202413395-s003.zip › Supporting Info - Machine readable data part 2/Figure 10 - esterification and mutual information/Mutual Information and Regression outputs/Cross validation charts/CV_LOO_b_ratio.png]

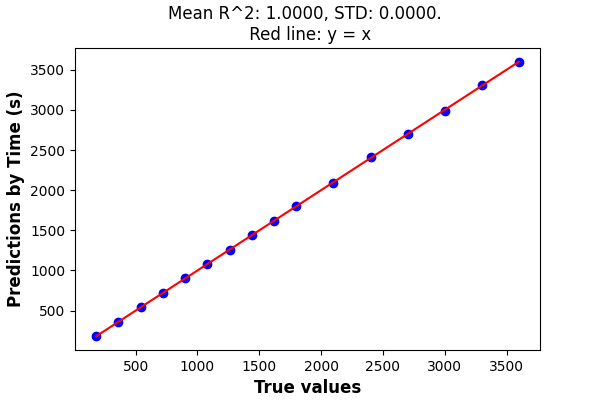

Supplement: Supplementary file 3 — Supporting Information [file ANIE-64-e202413395-s003.zip › Supporting Info - Machine readable data part 2/Figure 10 - esterification and mutual information/Mutual Information and Regression outputs/Cross validation charts/CV_LOO_Time (s).png]

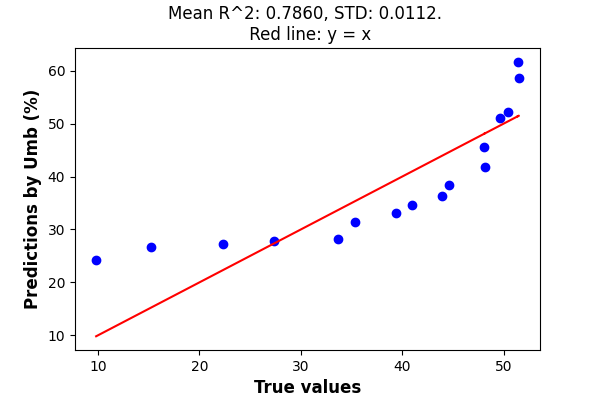

Supplement: Supplementary file 3 — Supporting Information [file ANIE-64-e202413395-s003.zip › Supporting Info - Machine readable data part 2/Figure 10 - esterification and mutual information/Mutual Information and Regression outputs/Cross validation charts/CV_LOO_Umb (%).png]

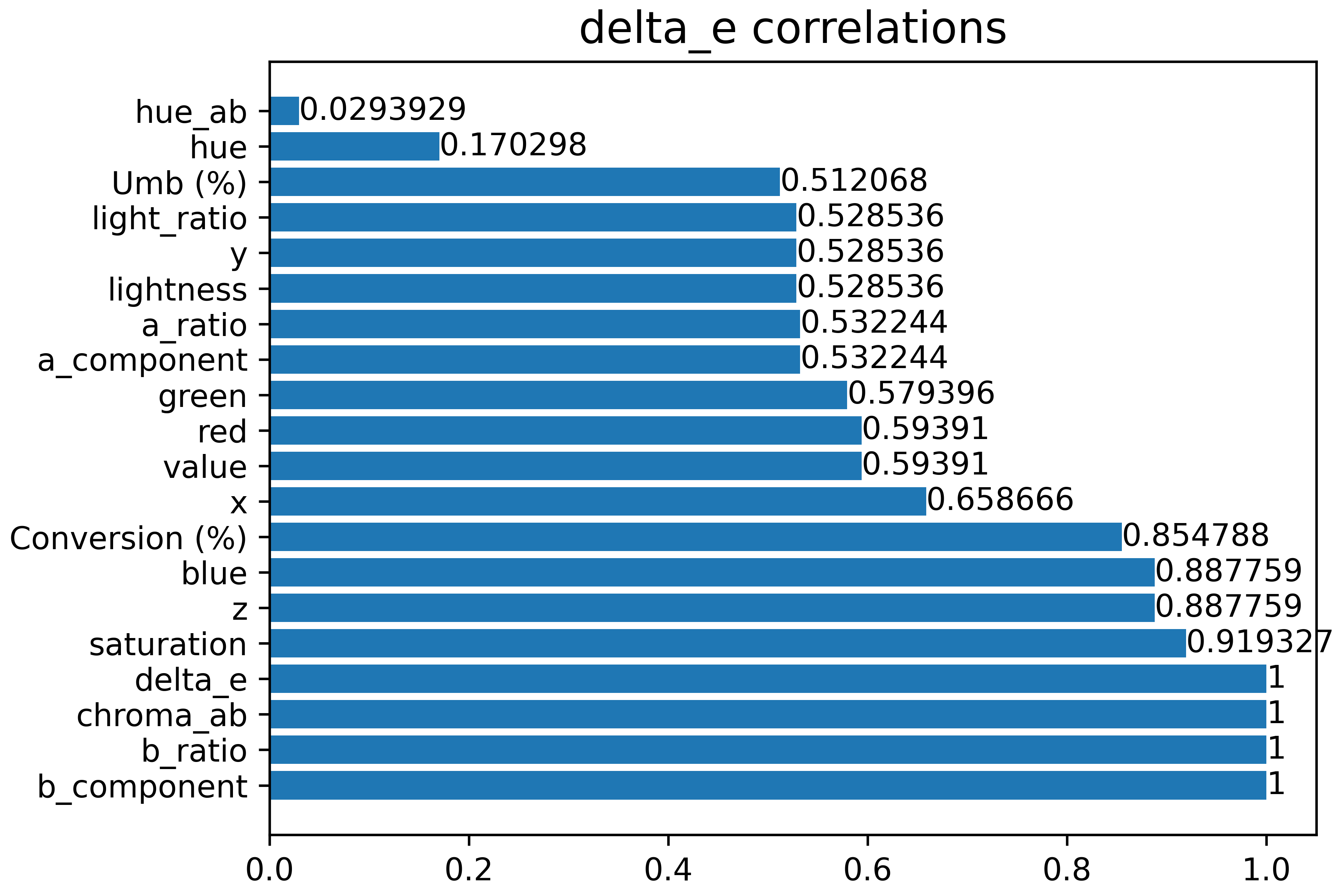

Supplement: Supplementary file 3 — Supporting Information [file ANIE-64-e202413395-s003.zip › Supporting Info - Machine readable data part 2/Figure 10 - esterification and mutual information/Mutual Information and Regression outputs/Mutual information charts/Correlations for delta_e.png]

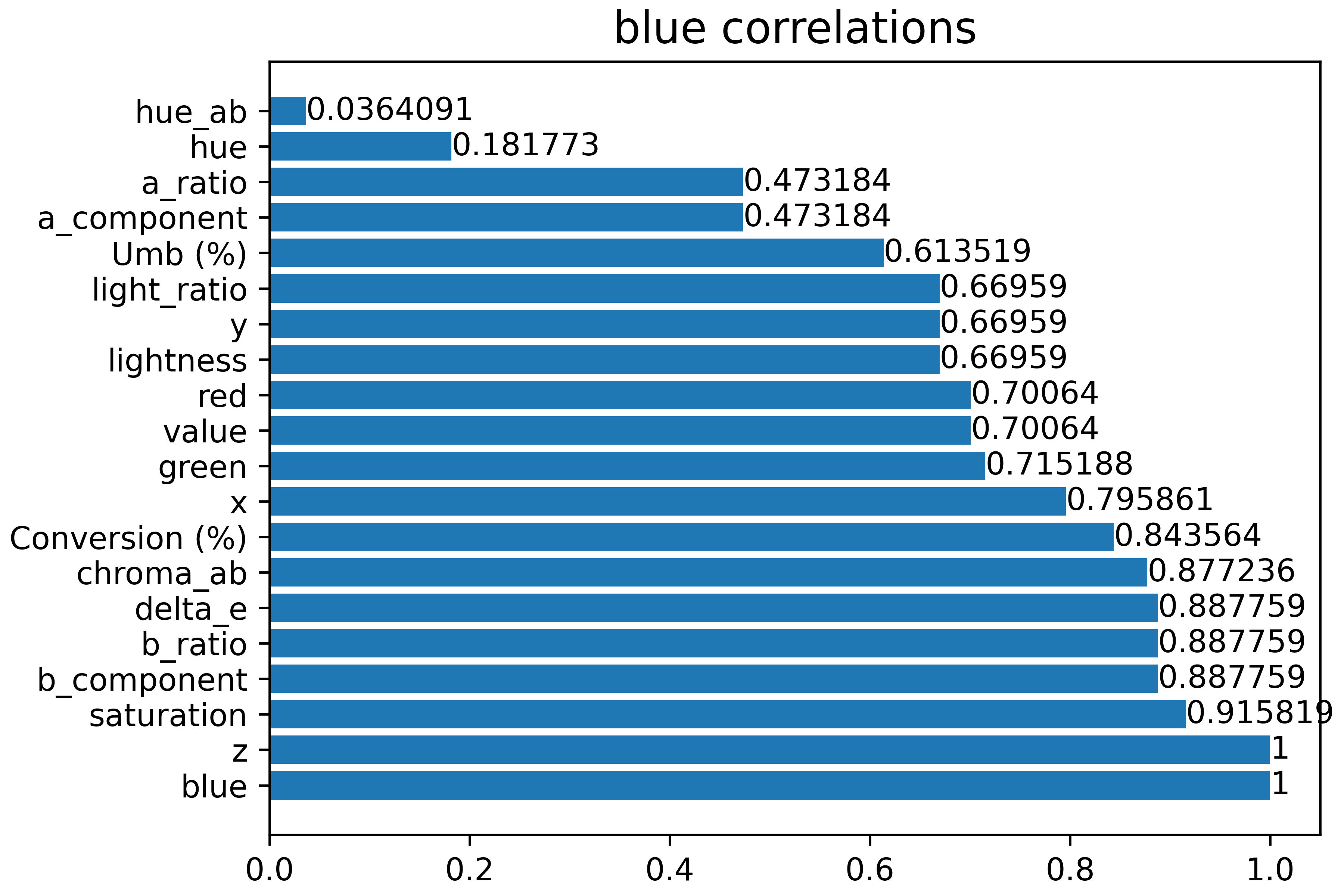

Supplement: Supplementary file 3 — Supporting Information [file ANIE-64-e202413395-s003.zip › Supporting Info - Machine readable data part 2/Figure 10 - esterification and mutual information/Mutual Information and Regression outputs/Mutual information charts/Correlations for blue.png]

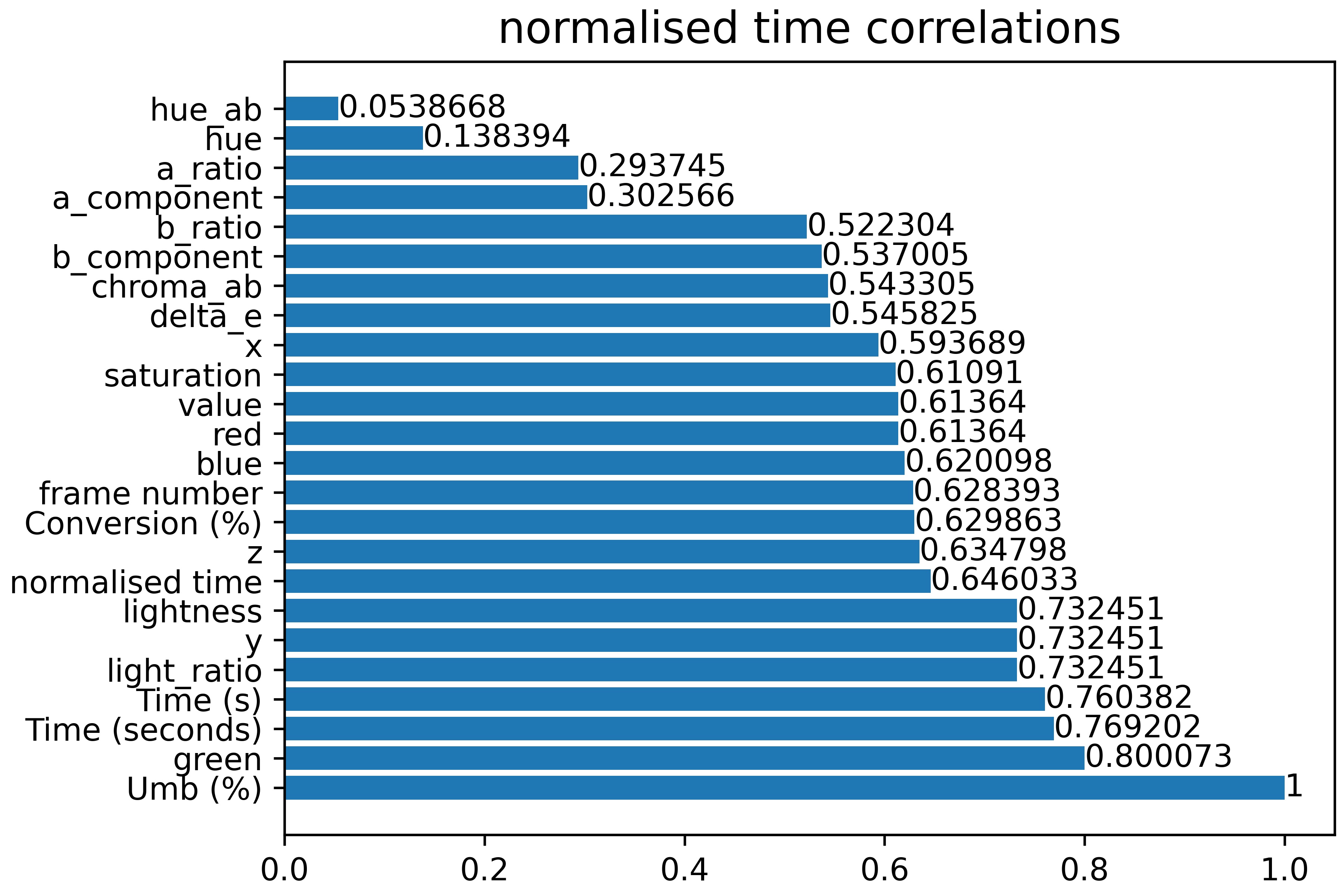

Supplement: Supplementary file 3 — Supporting Information [file ANIE-64-e202413395-s003.zip › Supporting Info - Machine readable data part 2/Figure 10 - esterification and mutual information/Mutual Information and Regression outputs/Mutual information charts/Correlations for normalised time.png]

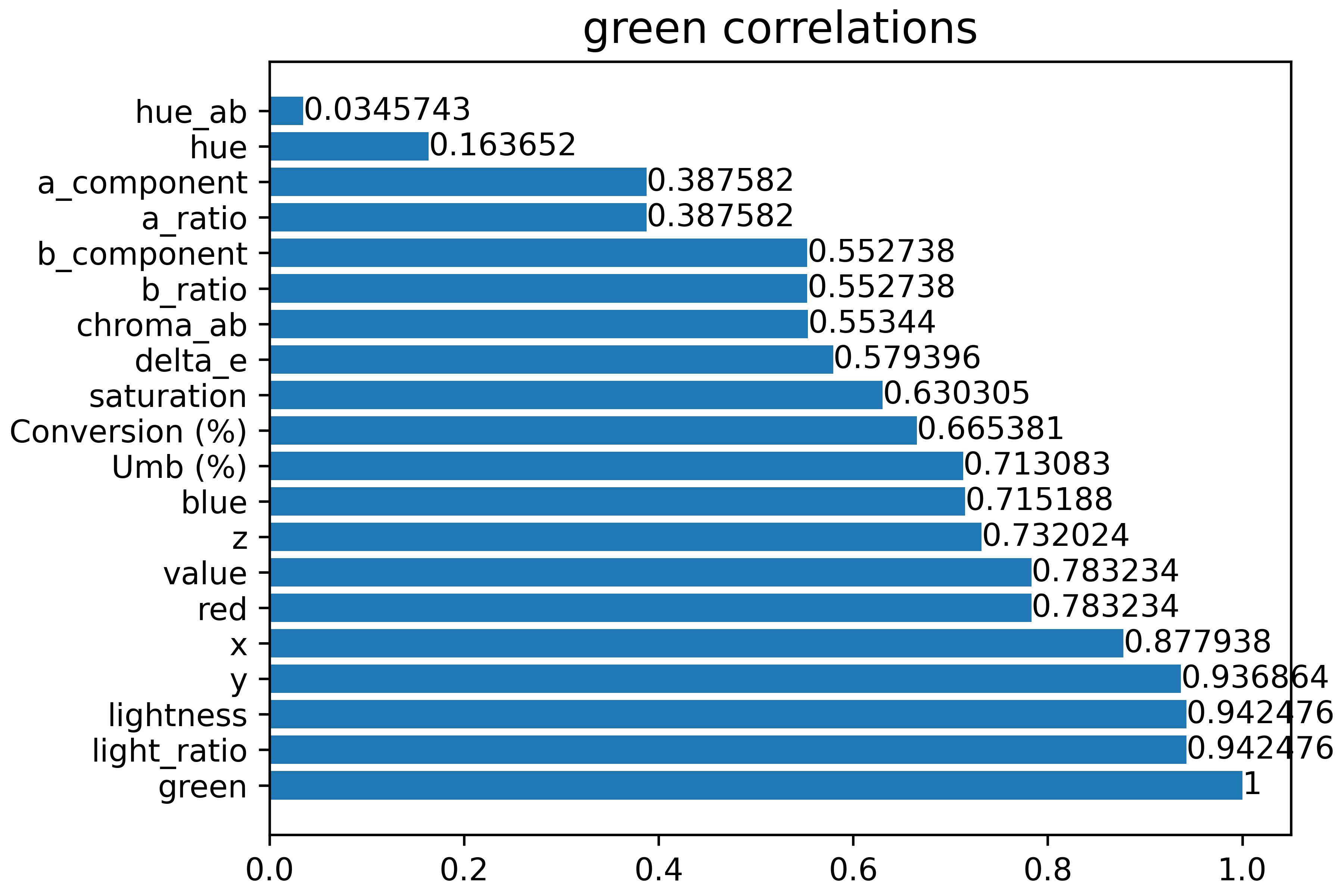

Supplement: Supplementary file 3 — Supporting Information [file ANIE-64-e202413395-s003.zip › Supporting Info - Machine readable data part 2/Figure 10 - esterification and mutual information/Mutual Information and Regression outputs/Mutual information charts/Correlations for green.png]

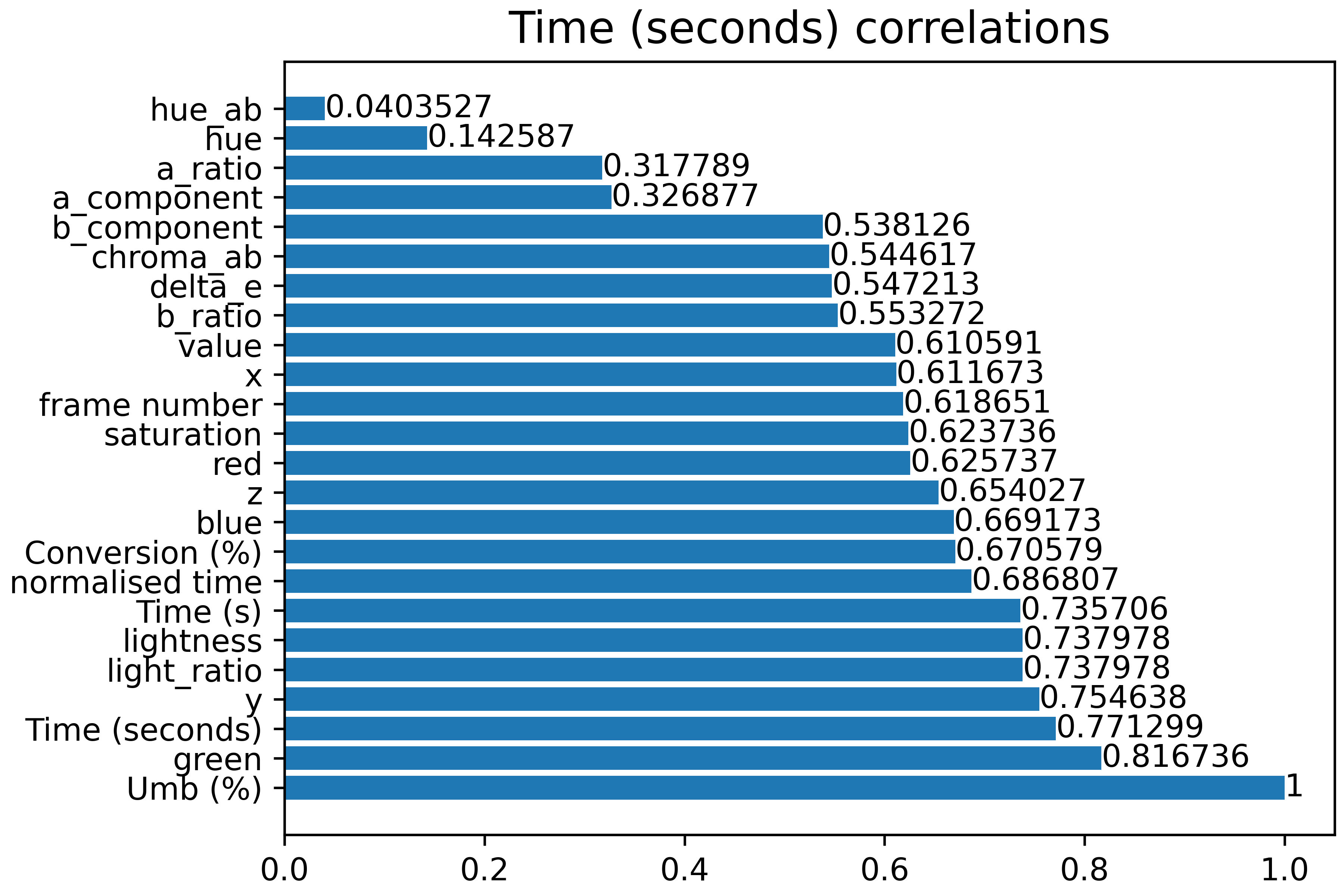

Supplement: Supplementary file 3 — Supporting Information [file ANIE-64-e202413395-s003.zip › Supporting Info - Machine readable data part 2/Figure 10 - esterification and mutual information/Mutual Information and Regression outputs/Mutual information charts/Correlations for Time (seconds).png]

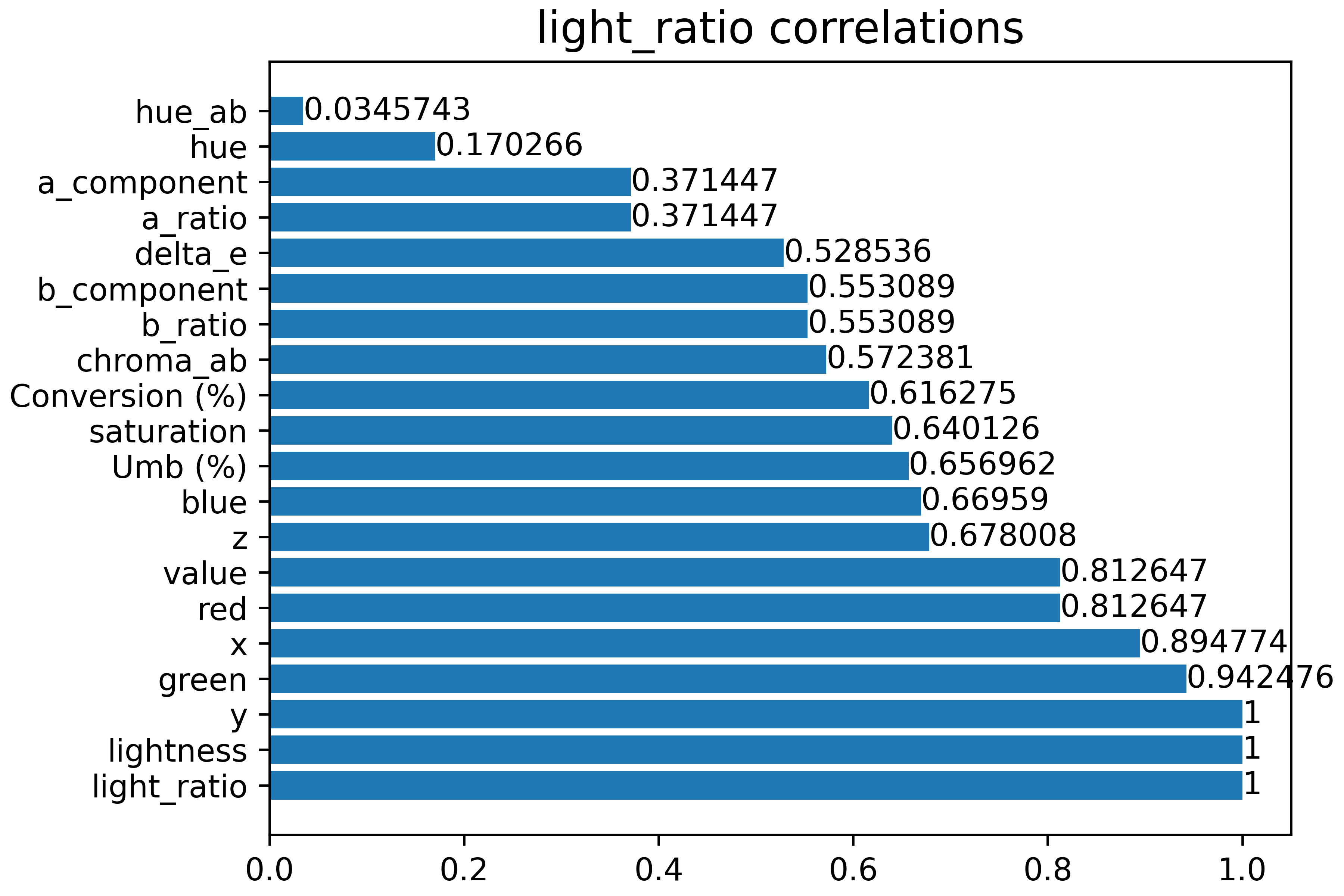

Supplement: Supplementary file 3 — Supporting Information [file ANIE-64-e202413395-s003.zip › Supporting Info - Machine readable data part 2/Figure 10 - esterification and mutual information/Mutual Information and Regression outputs/Mutual information charts/Correlations for light_ratio.png]

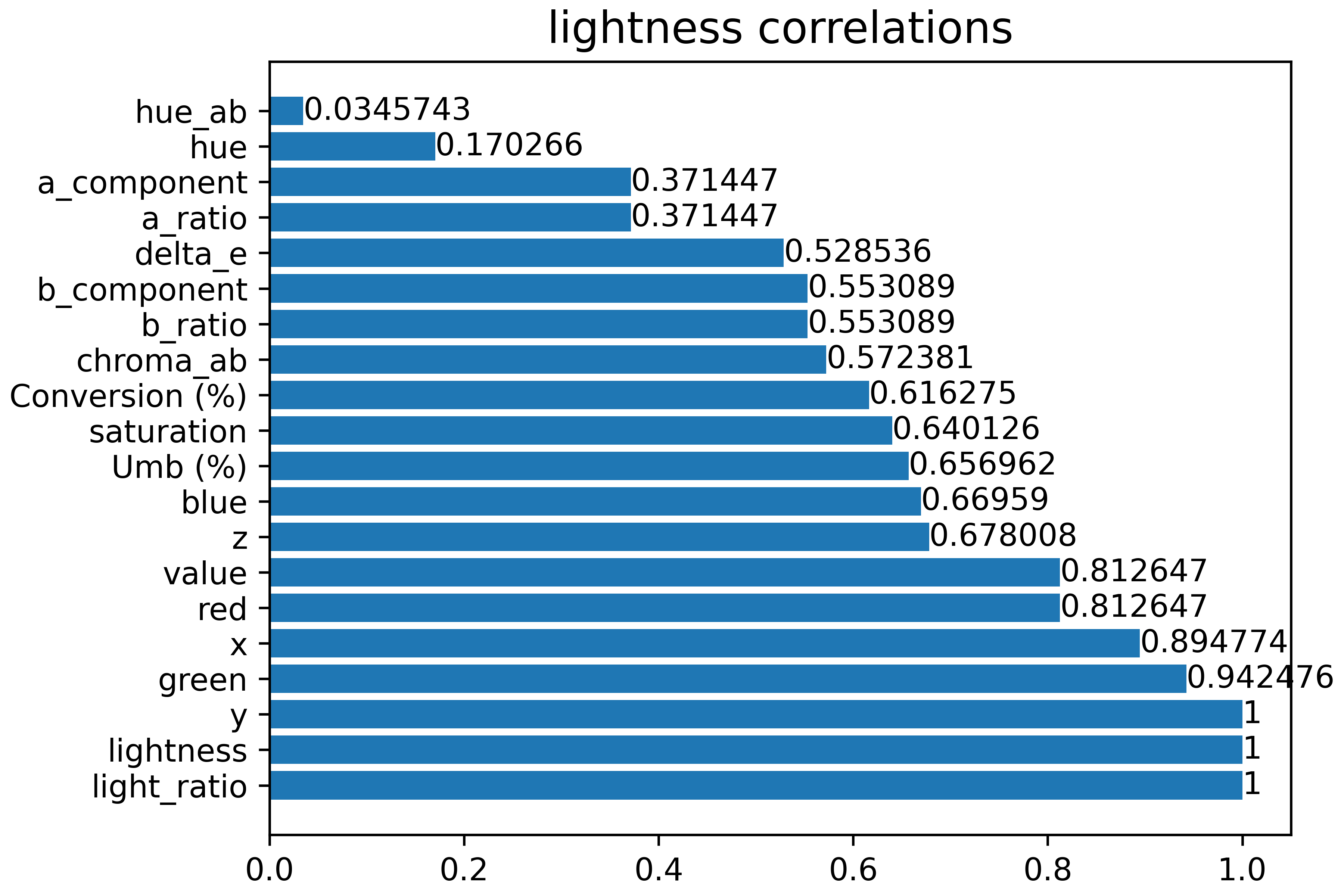

Supplement: Supplementary file 3 — Supporting Information [file ANIE-64-e202413395-s003.zip › Supporting Info - Machine readable data part 2/Figure 10 - esterification and mutual information/Mutual Information and Regression outputs/Mutual information charts/Correlations for lightness.png]

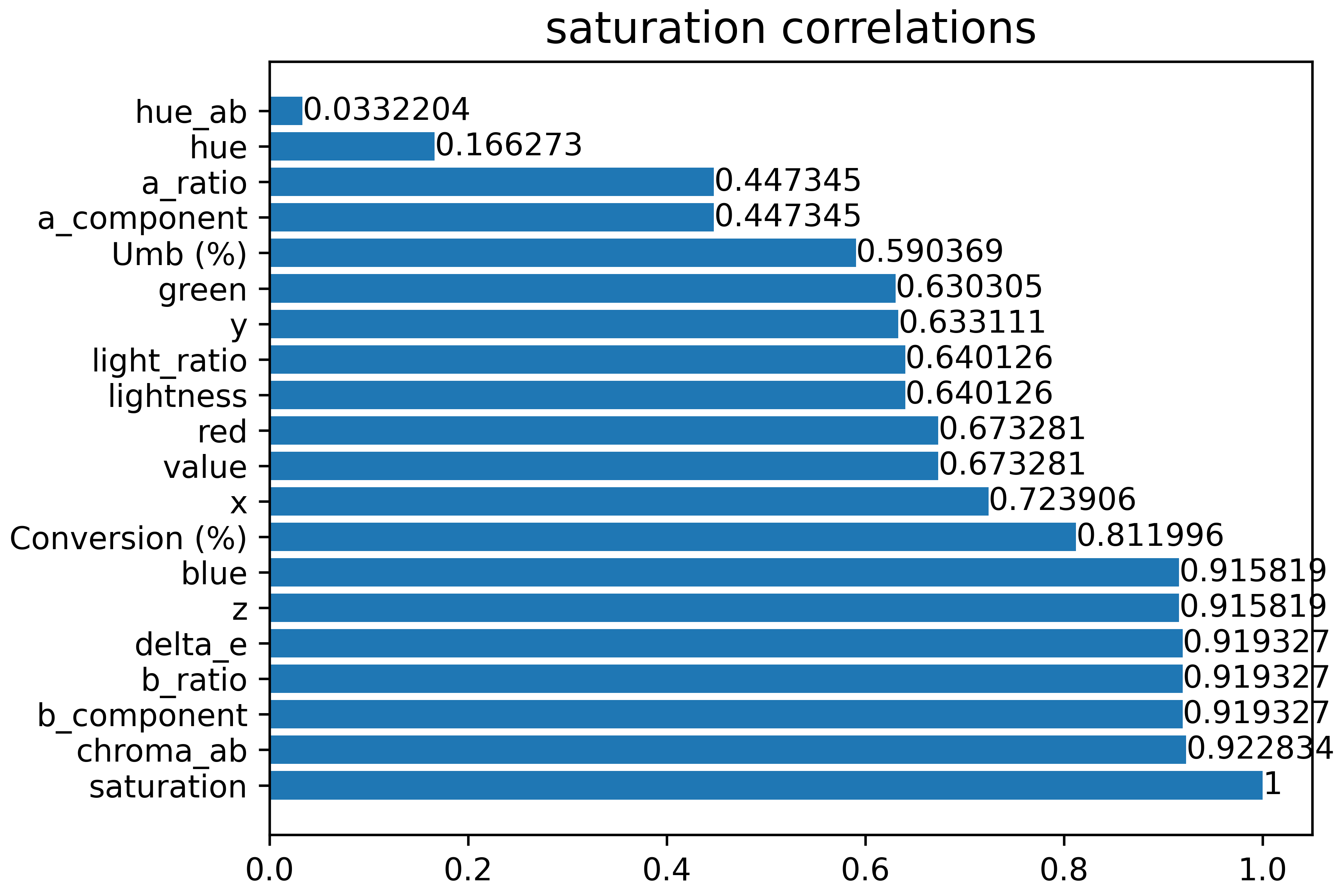

Supplement: Supplementary file 3 — Supporting Information [file ANIE-64-e202413395-s003.zip › Supporting Info - Machine readable data part 2/Figure 10 - esterification and mutual information/Mutual Information and Regression outputs/Mutual information charts/Correlations for saturation.png]

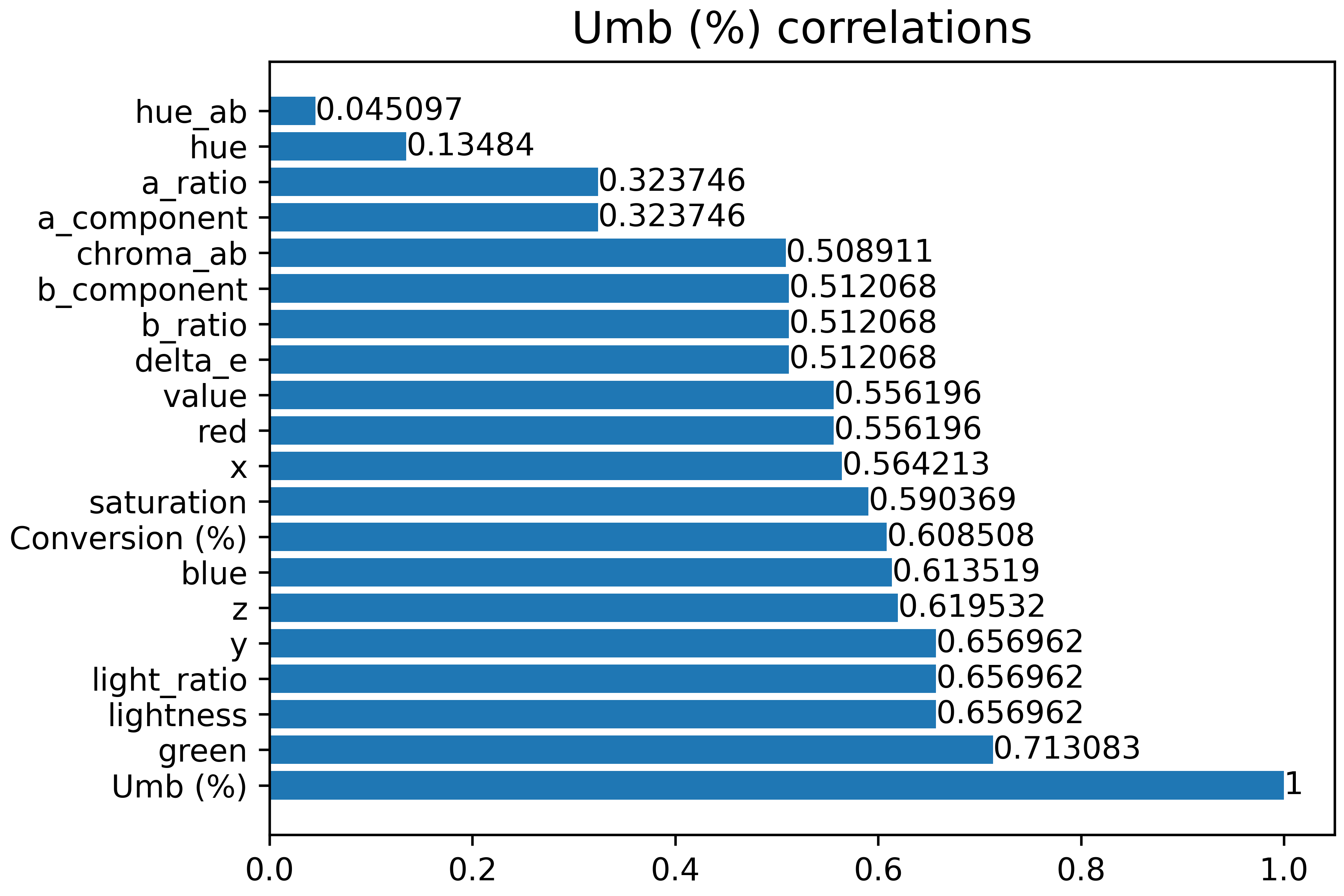

Supplement: Supplementary file 3 — Supporting Information [file ANIE-64-e202413395-s003.zip › Supporting Info - Machine readable data part 2/Figure 10 - esterification and mutual information/Mutual Information and Regression outputs/Mutual information charts/Correlations for Umb (%).png]

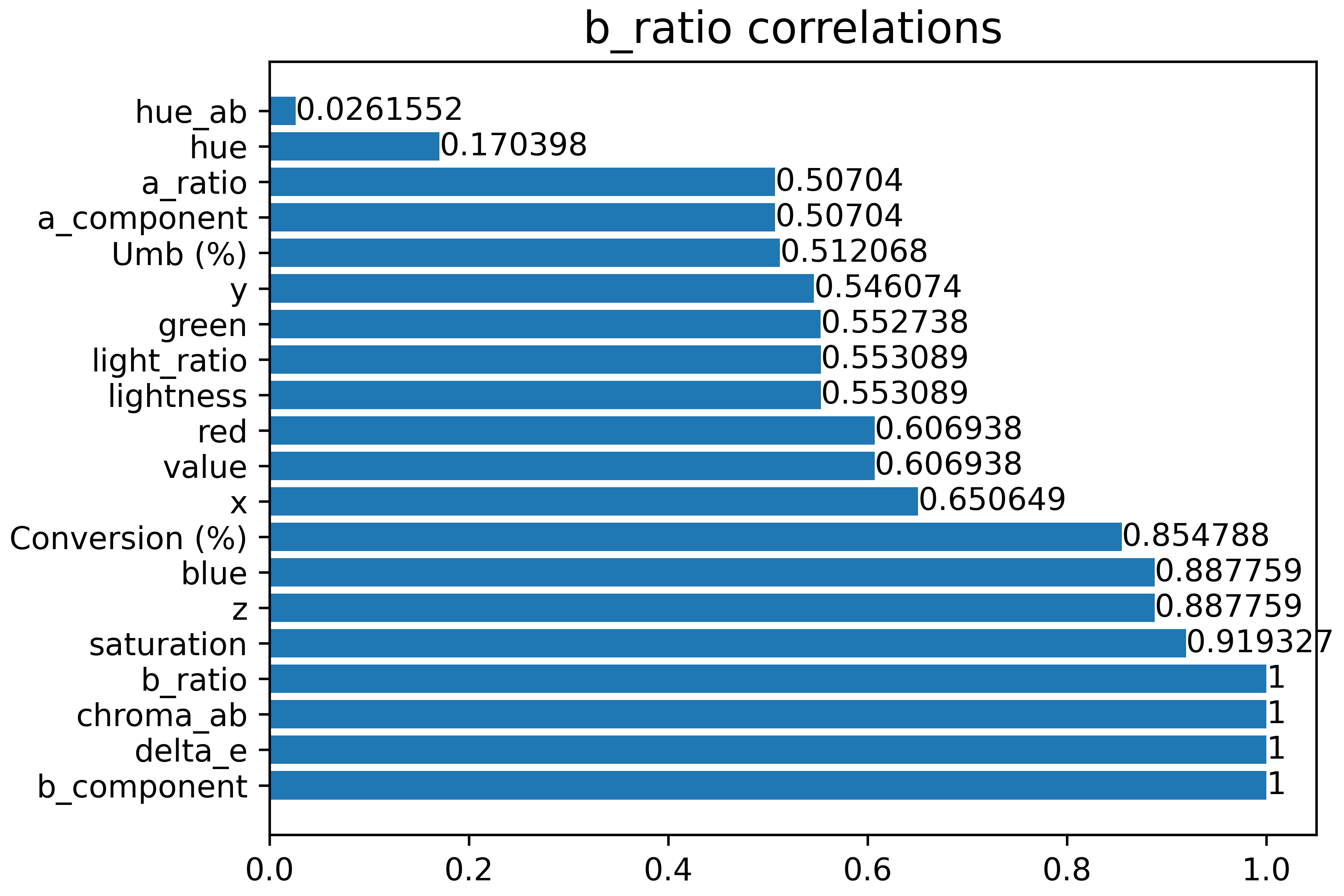

Supplement: Supplementary file 3 — Supporting Information [file ANIE-64-e202413395-s003.zip › Supporting Info - Machine readable data part 2/Figure 10 - esterification and mutual information/Mutual Information and Regression outputs/Mutual information charts/Correlations for b_ratio.png]

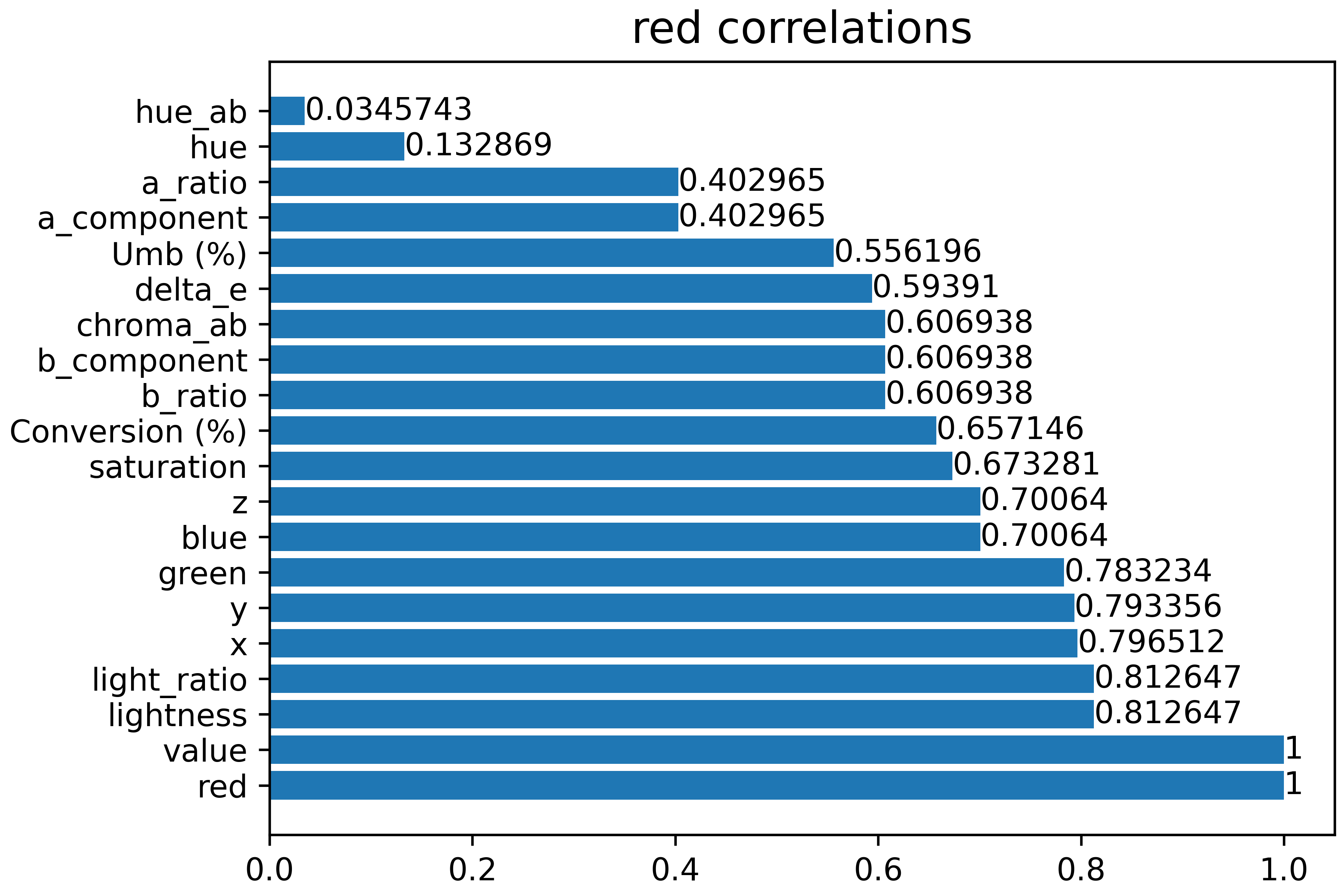

Supplement: Supplementary file 3 — Supporting Information [file ANIE-64-e202413395-s003.zip › Supporting Info - Machine readable data part 2/Figure 10 - esterification and mutual information/Mutual Information and Regression outputs/Mutual information charts/Correlations for red.png]

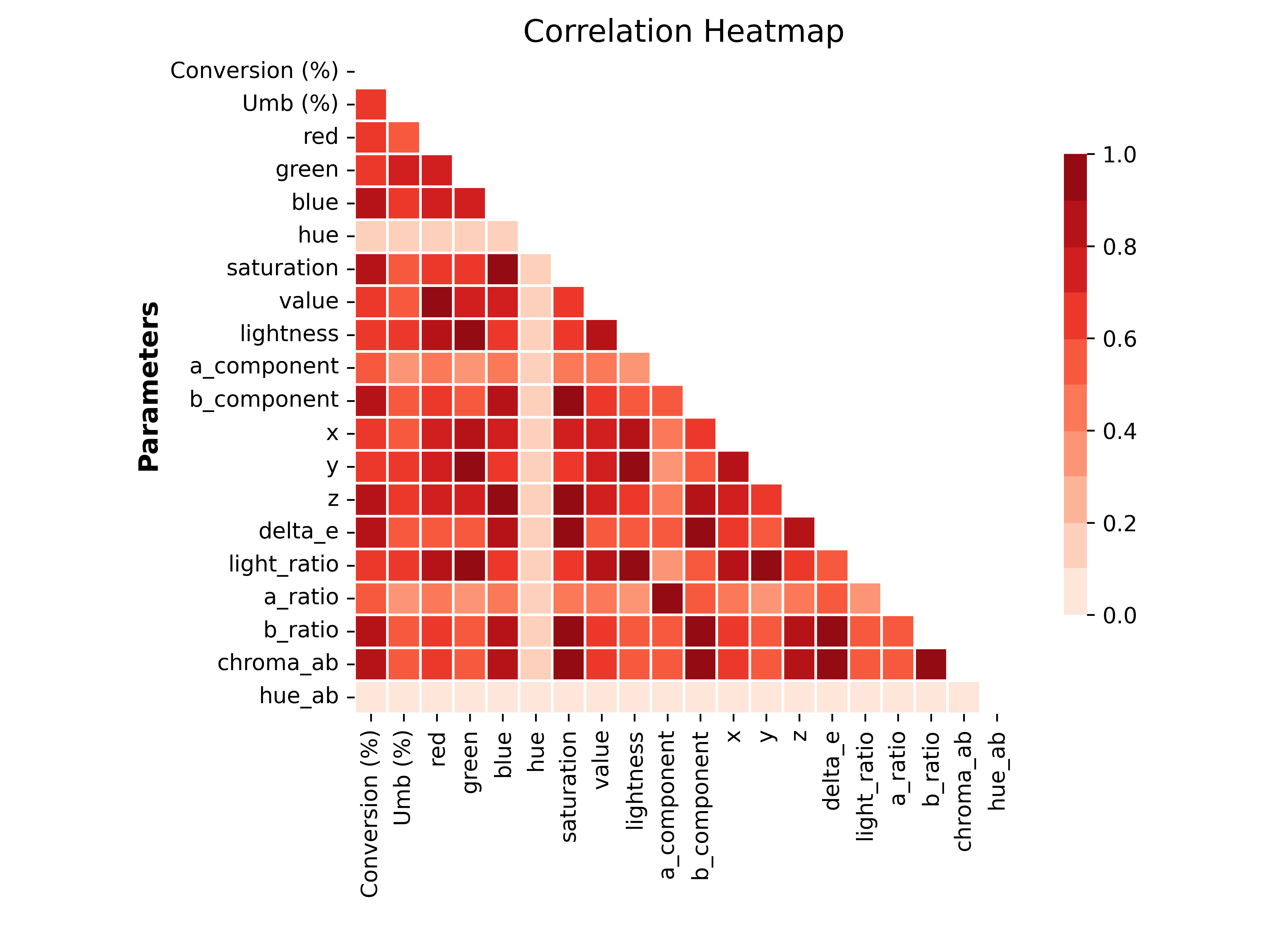

Supplement: Supplementary file 3 — Supporting Information [file ANIE-64-e202413395-s003.zip › Supporting Info - Machine readable data part 2/Figure 10 - esterification and mutual information/Mutual Information and Regression outputs/Mutual information charts/Correlation Heatmap.png]

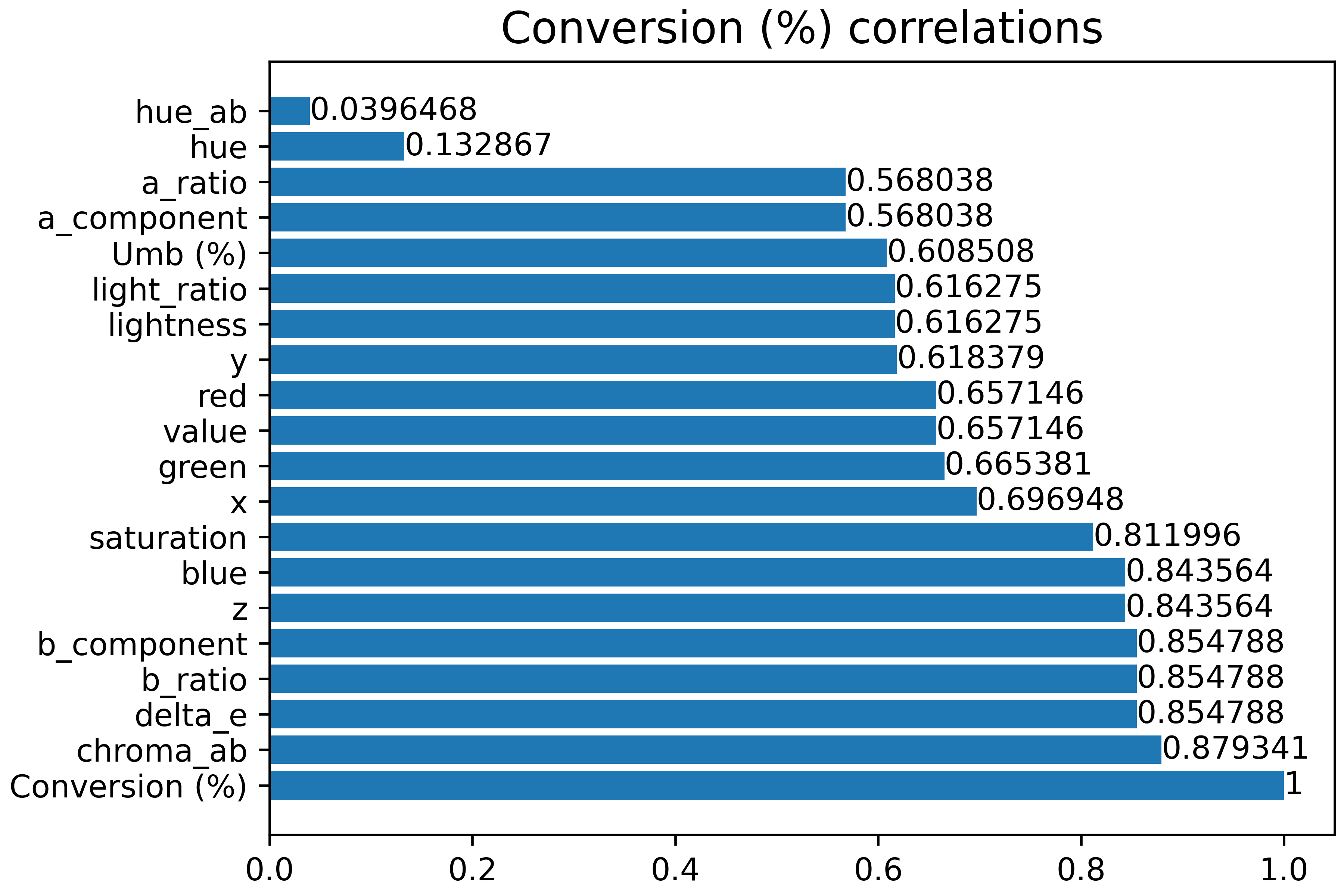

Supplement: Supplementary file 3 — Supporting Information [file ANIE-64-e202413395-s003.zip › Supporting Info - Machine readable data part 2/Figure 10 - esterification and mutual information/Mutual Information and Regression outputs/Mutual information charts/Correlations for Conversion (%).png]

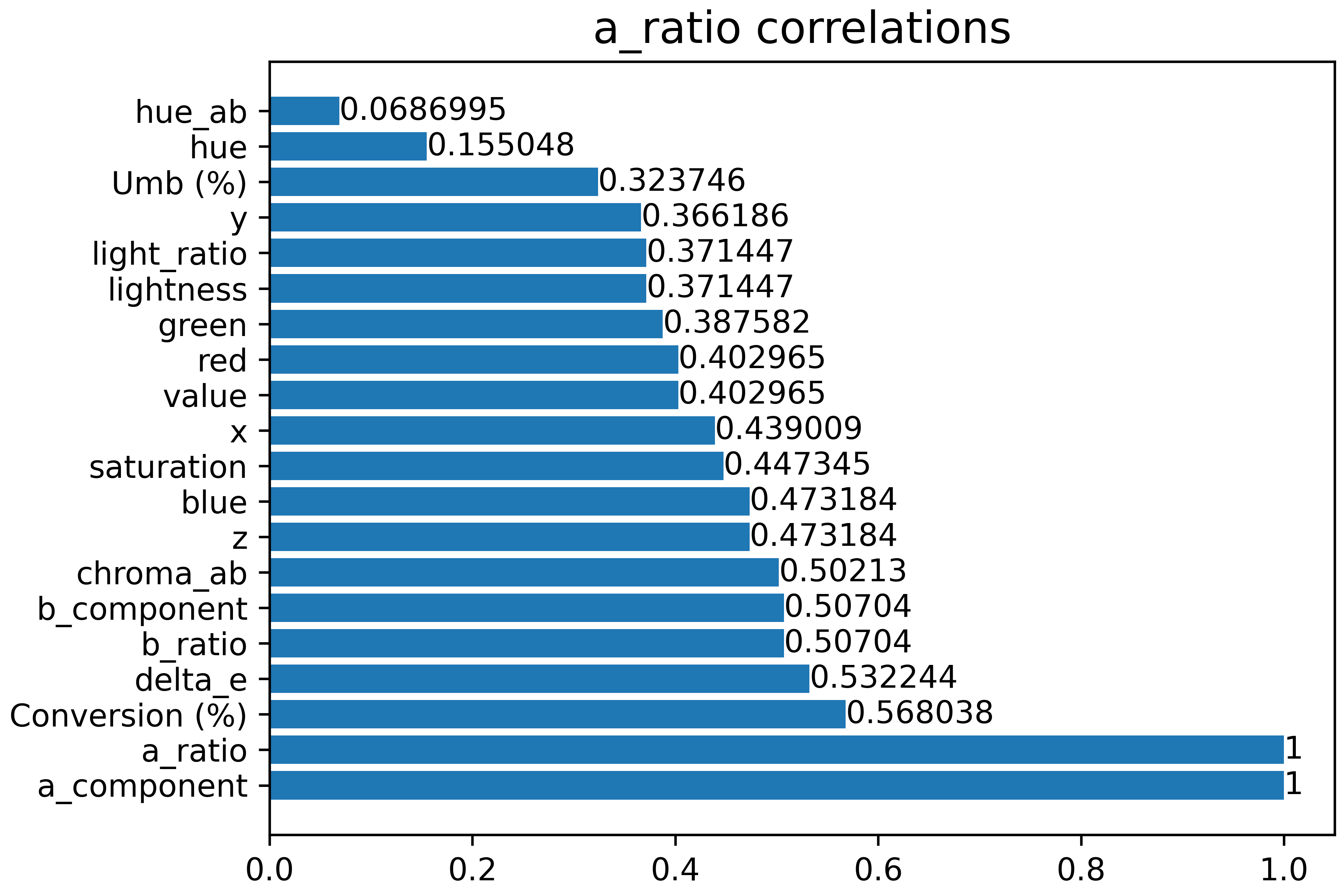

Supplement: Supplementary file 3 — Supporting Information [file ANIE-64-e202413395-s003.zip › Supporting Info - Machine readable data part 2/Figure 10 - esterification and mutual information/Mutual Information and Regression outputs/Mutual information charts/Correlations for a_ratio.png]

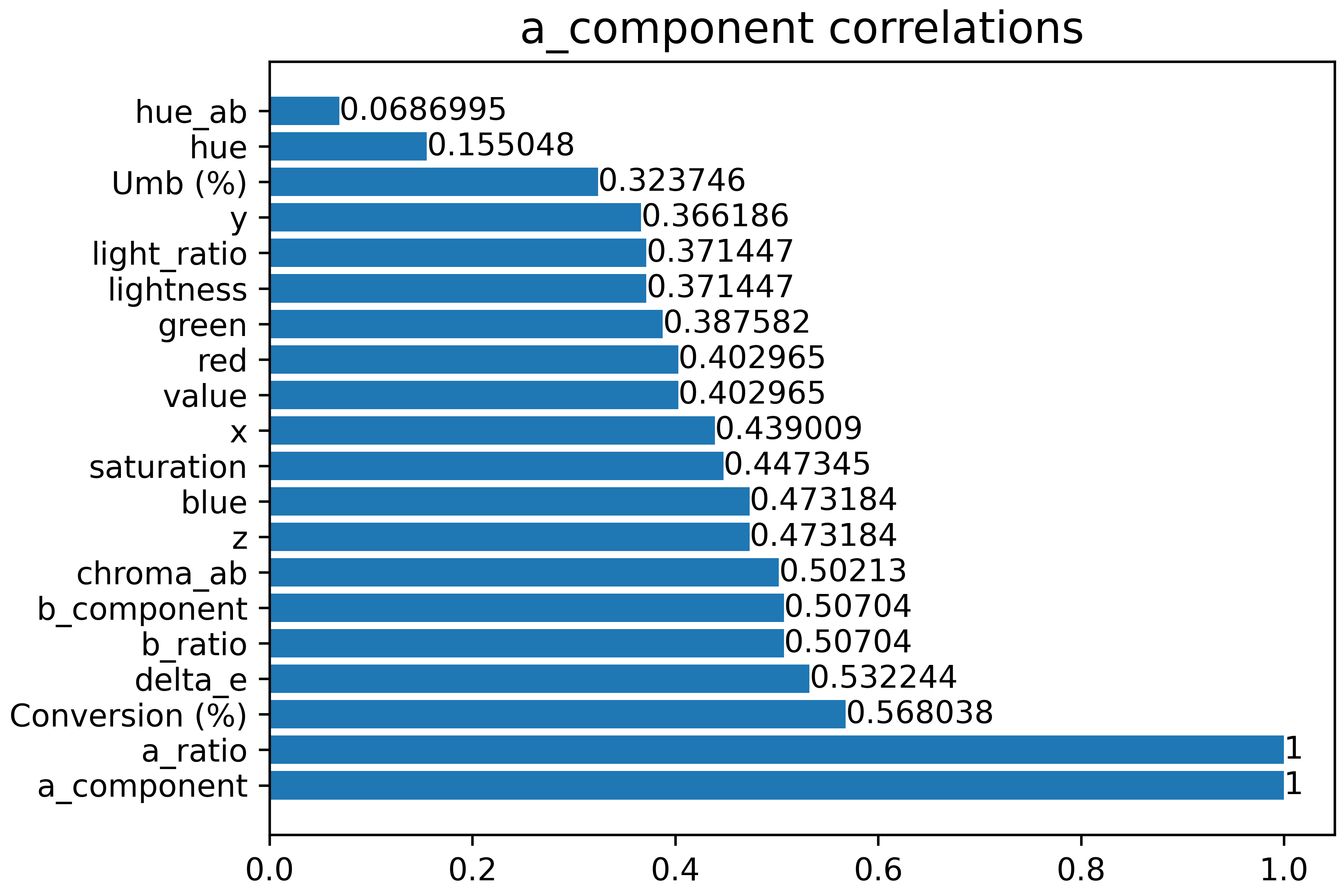

Supplement: Supplementary file 3 — Supporting Information [file ANIE-64-e202413395-s003.zip › Supporting Info - Machine readable data part 2/Figure 10 - esterification and mutual information/Mutual Information and Regression outputs/Mutual information charts/Correlations for a_component.png]

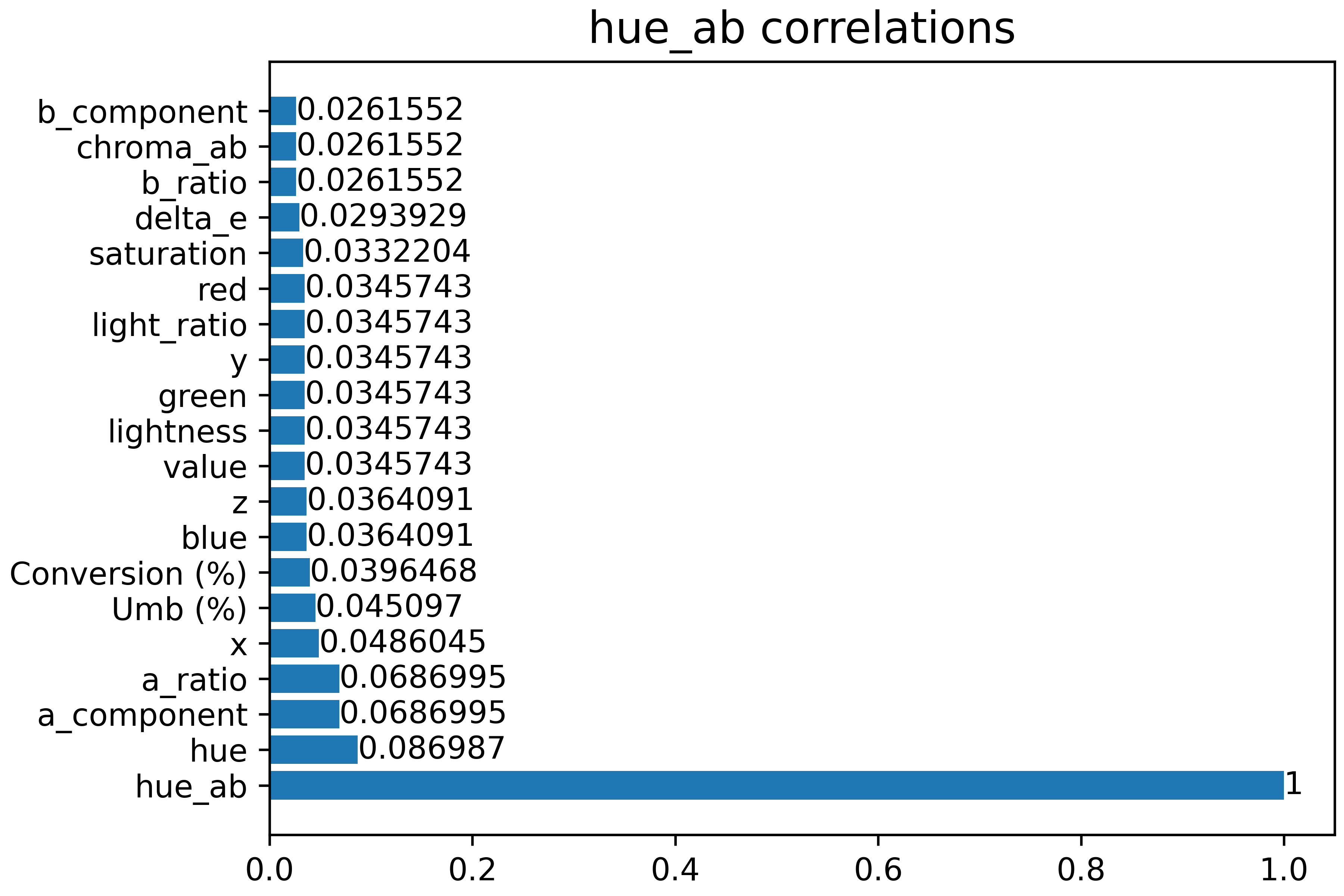

Supplement: Supplementary file 3 — Supporting Information [file ANIE-64-e202413395-s003.zip › Supporting Info - Machine readable data part 2/Figure 10 - esterification and mutual information/Mutual Information and Regression outputs/Mutual information charts/Correlations for hue_ab.png]

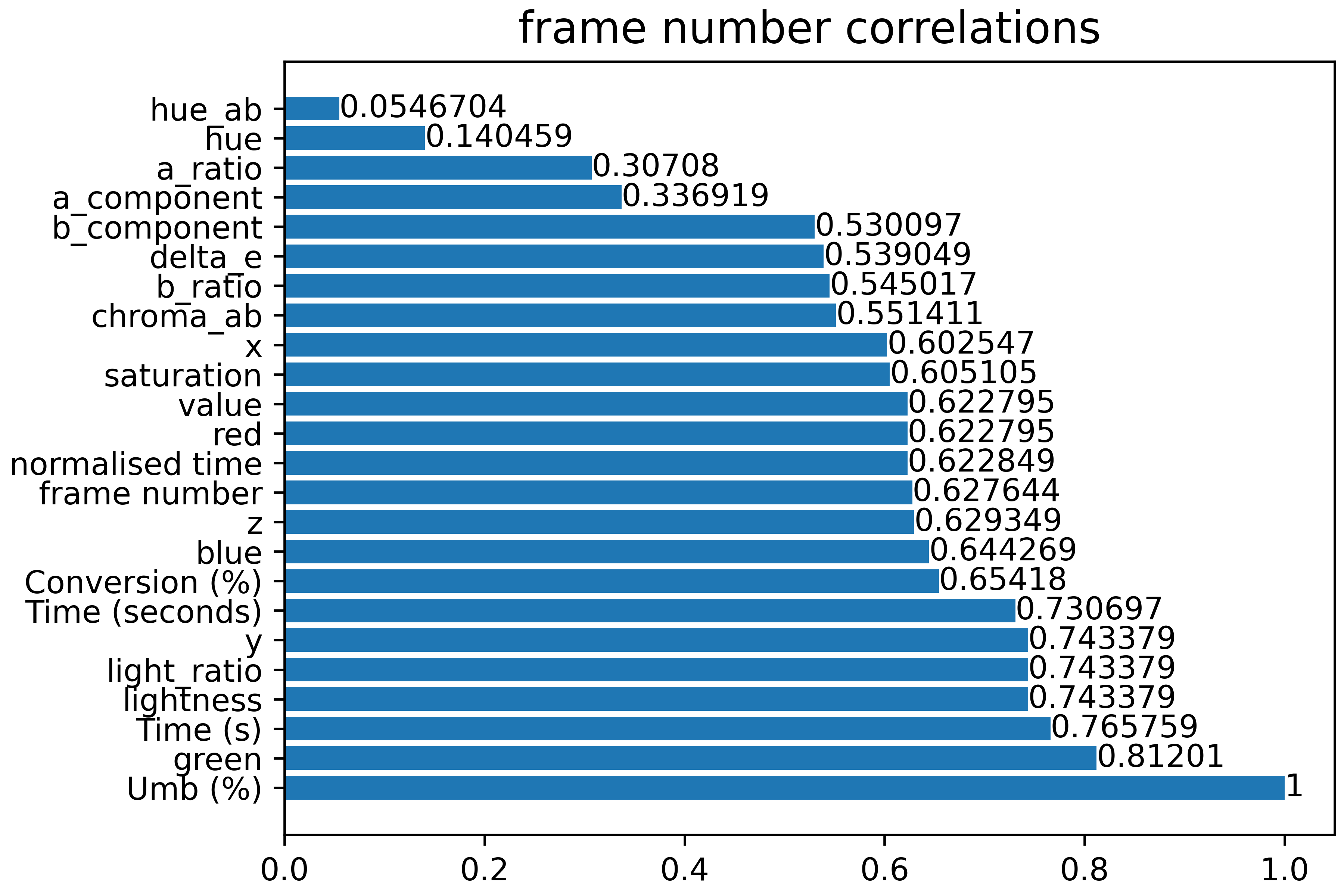

Supplement: Supplementary file 3 — Supporting Information [file ANIE-64-e202413395-s003.zip › Supporting Info - Machine readable data part 2/Figure 10 - esterification and mutual information/Mutual Information and Regression outputs/Mutual information charts/Correlations for frame number.png]

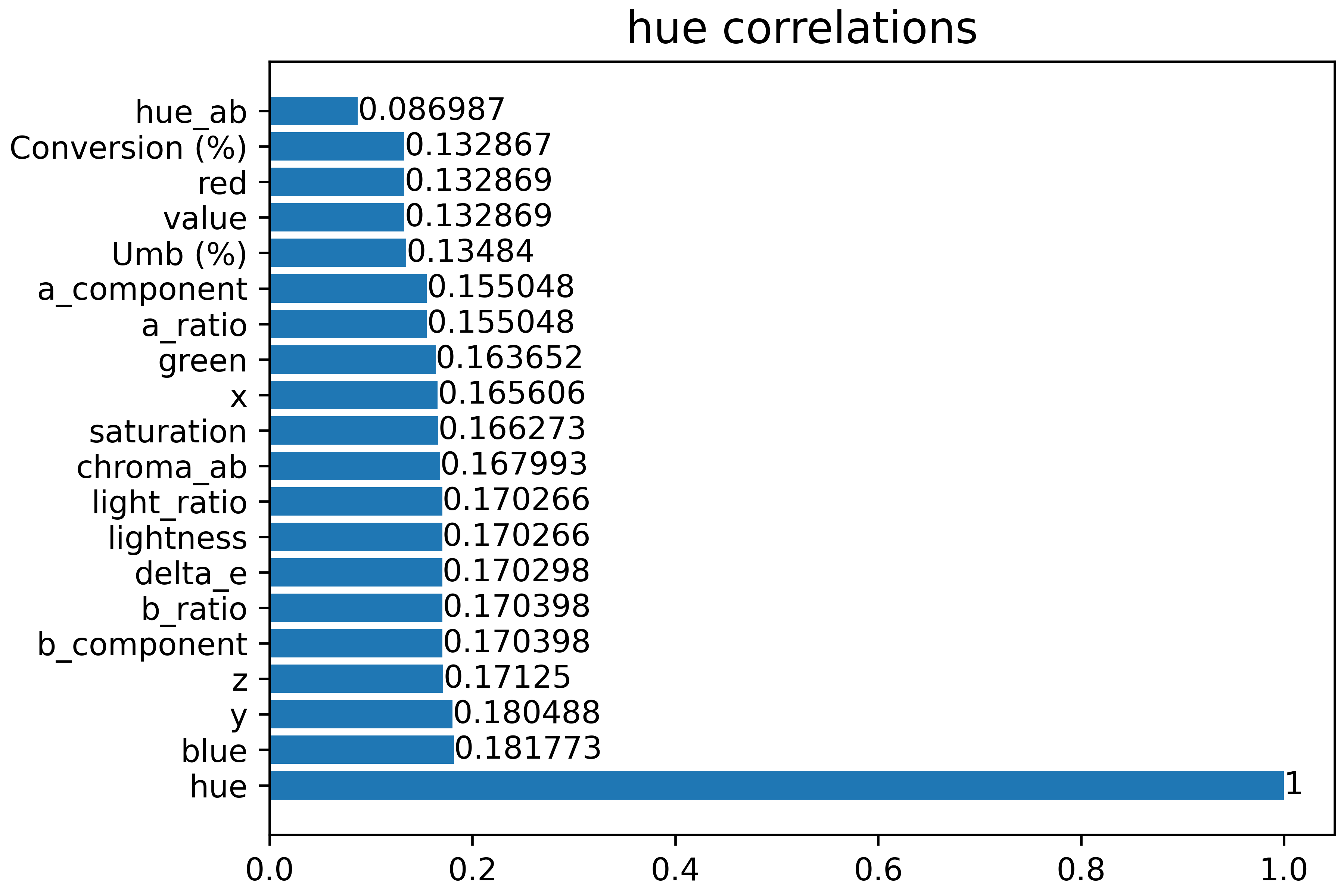

Supplement: Supplementary file 3 — Supporting Information [file ANIE-64-e202413395-s003.zip › Supporting Info - Machine readable data part 2/Figure 10 - esterification and mutual information/Mutual Information and Regression outputs/Mutual information charts/Correlations for hue.png]

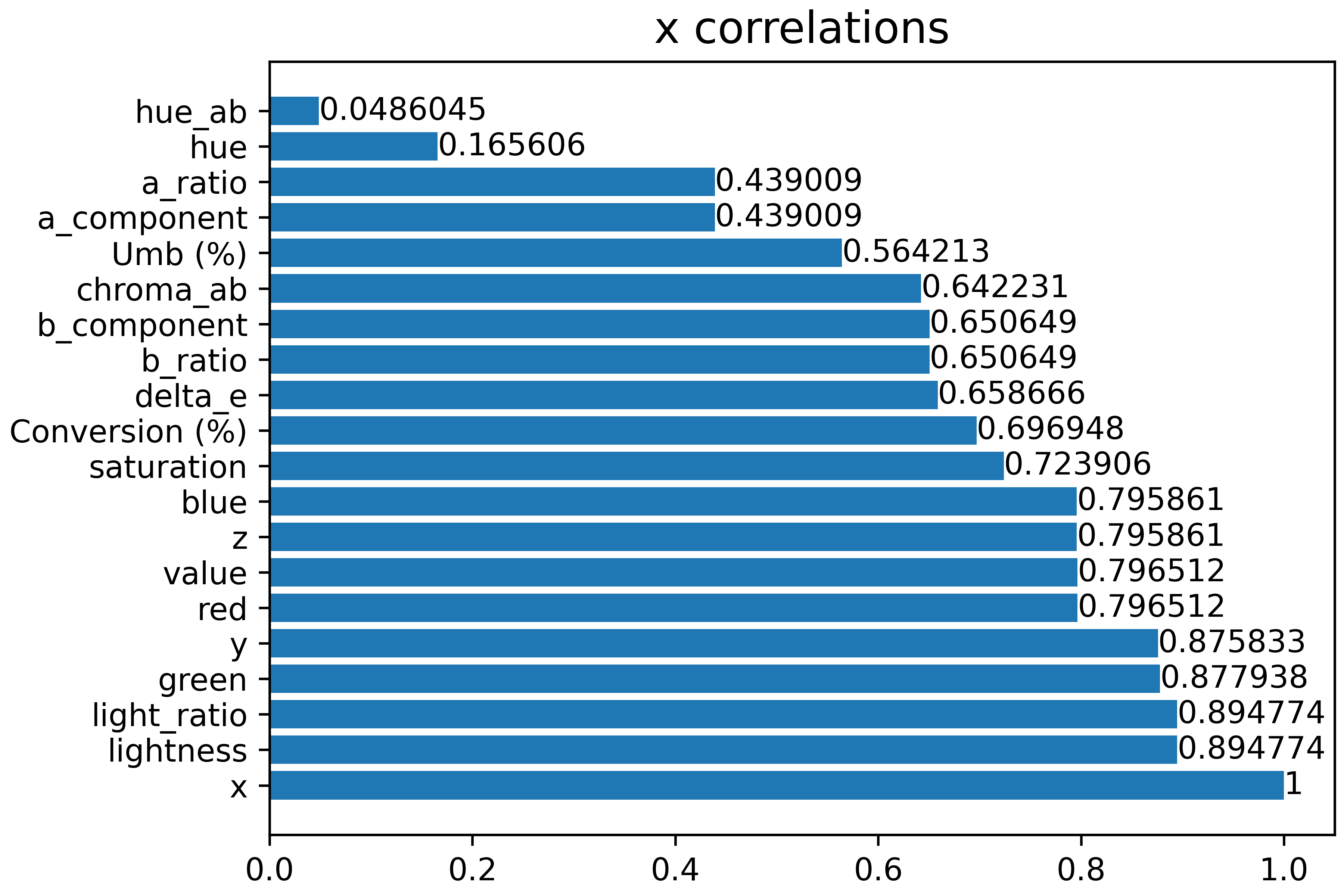

Supplement: Supplementary file 3 — Supporting Information [file ANIE-64-e202413395-s003.zip › Supporting Info - Machine readable data part 2/Figure 10 - esterification and mutual information/Mutual Information and Regression outputs/Mutual information charts/Correlations for x.png]

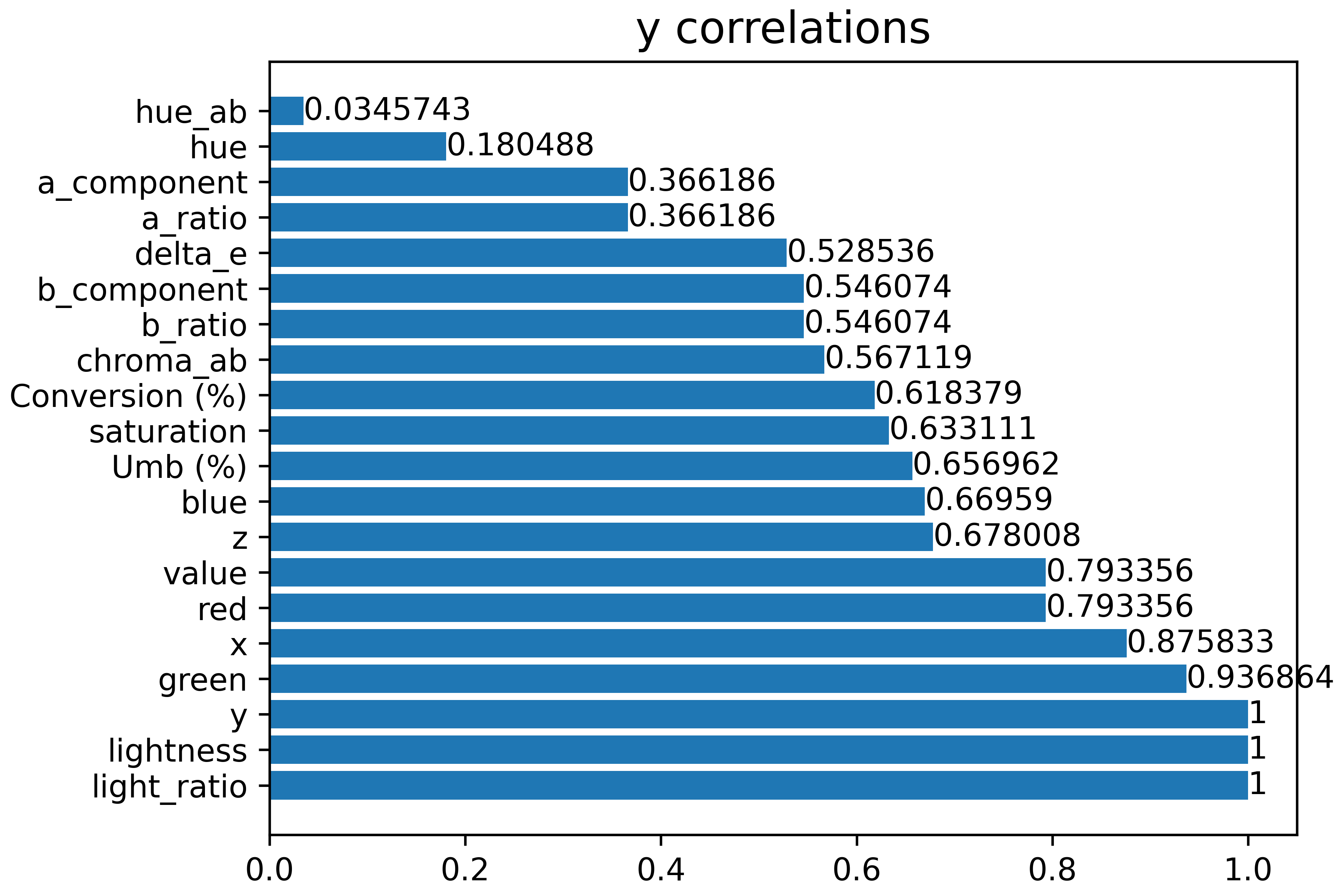

Supplement: Supplementary file 3 — Supporting Information [file ANIE-64-e202413395-s003.zip › Supporting Info - Machine readable data part 2/Figure 10 - esterification and mutual information/Mutual Information and Regression outputs/Mutual information charts/Correlations for y.png]

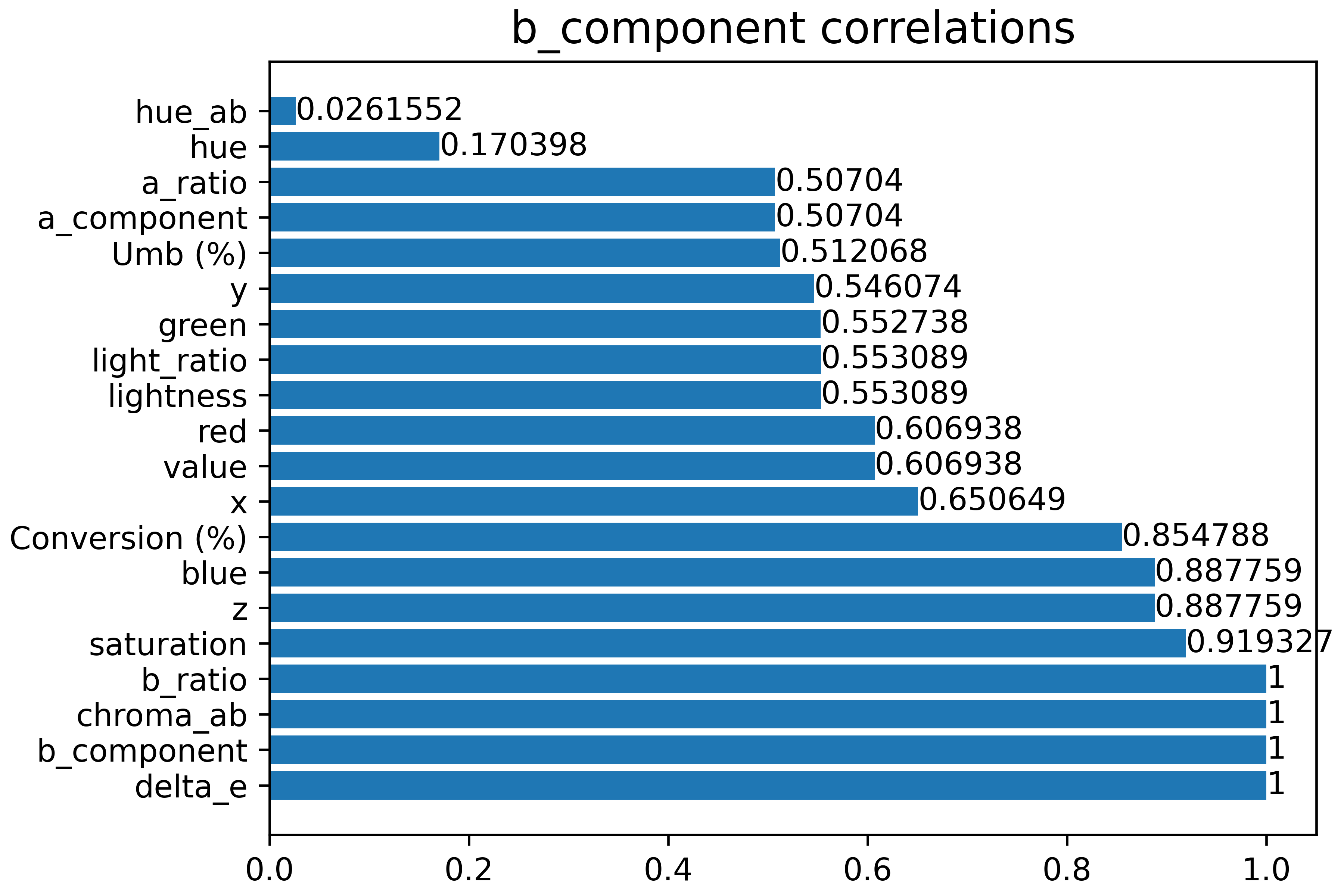

Supplement: Supplementary file 3 — Supporting Information [file ANIE-64-e202413395-s003.zip › Supporting Info - Machine readable data part 2/Figure 10 - esterification and mutual information/Mutual Information and Regression outputs/Mutual information charts/Correlations for b_component.png]

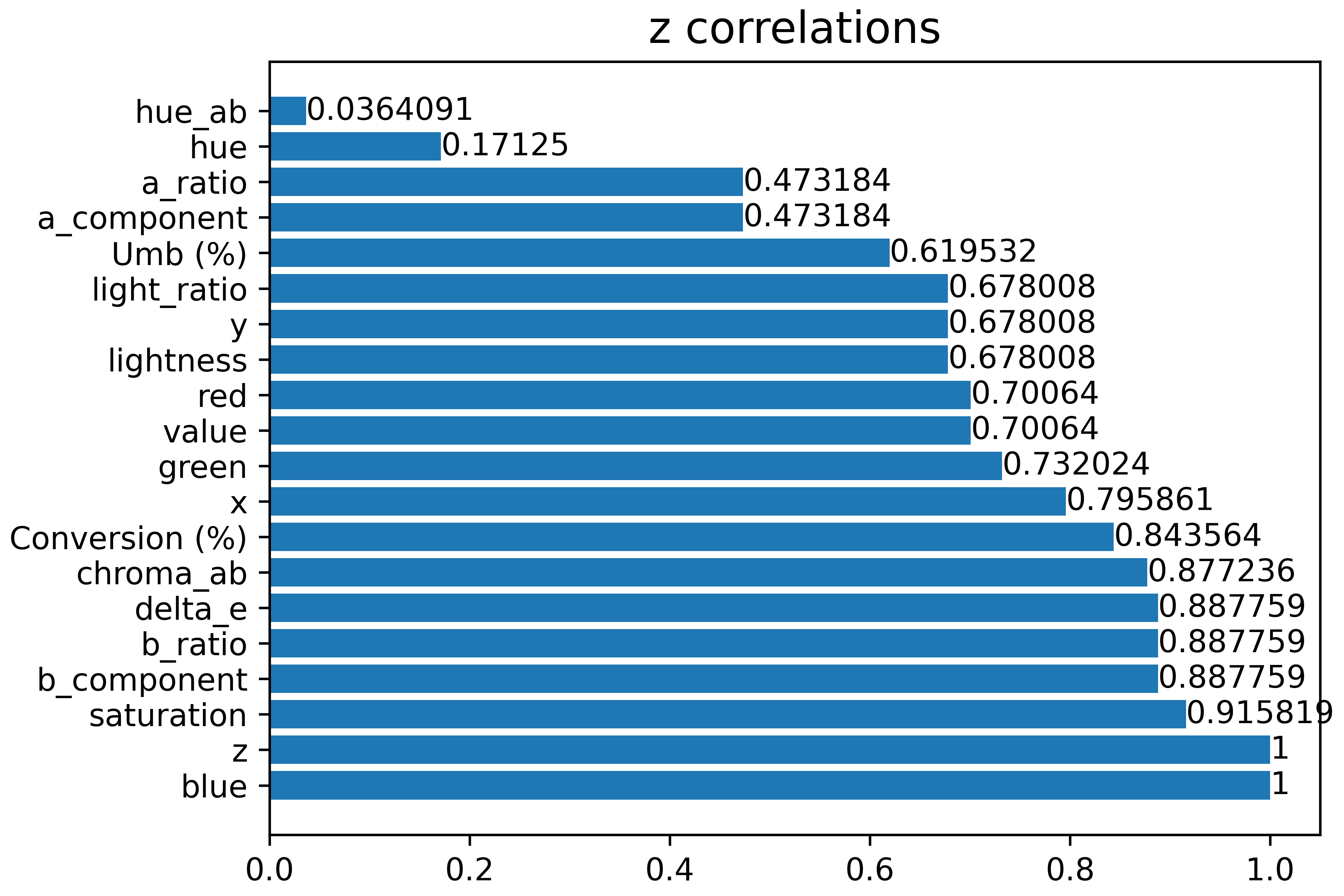

Supplement: Supplementary file 3 — Supporting Information [file ANIE-64-e202413395-s003.zip › Supporting Info - Machine readable data part 2/Figure 10 - esterification and mutual information/Mutual Information and Regression outputs/Mutual information charts/Correlations for z.png]

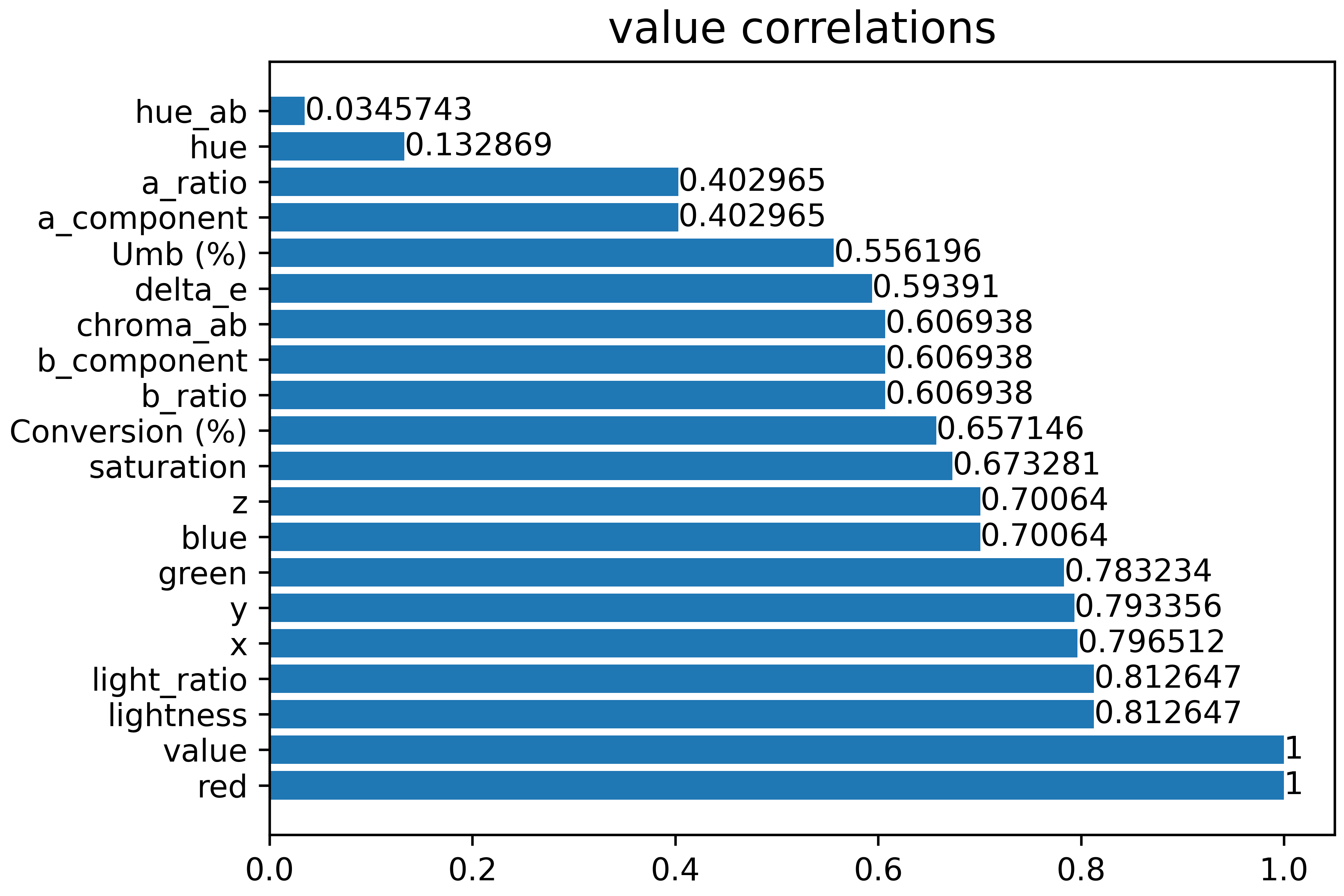

Supplement: Supplementary file 3 — Supporting Information [file ANIE-64-e202413395-s003.zip › Supporting Info - Machine readable data part 2/Figure 10 - esterification and mutual information/Mutual Information and Regression outputs/Mutual information charts/Correlations for value.png]

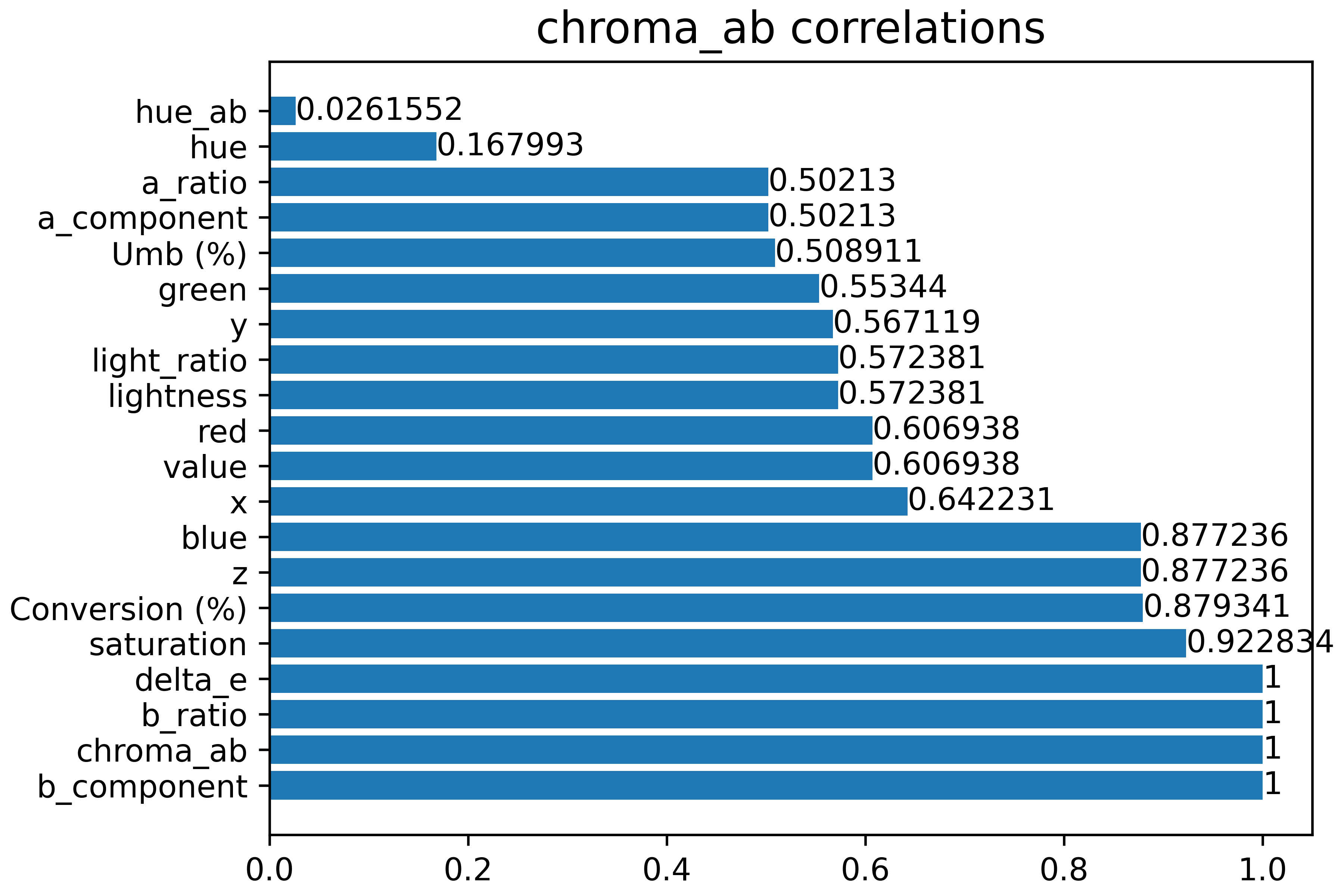

Supplement: Supplementary file 3 — Supporting Information [file ANIE-64-e202413395-s003.zip › Supporting Info - Machine readable data part 2/Figure 10 - esterification and mutual information/Mutual Information and Regression outputs/Mutual information charts/Correlations for chroma_ab.png]

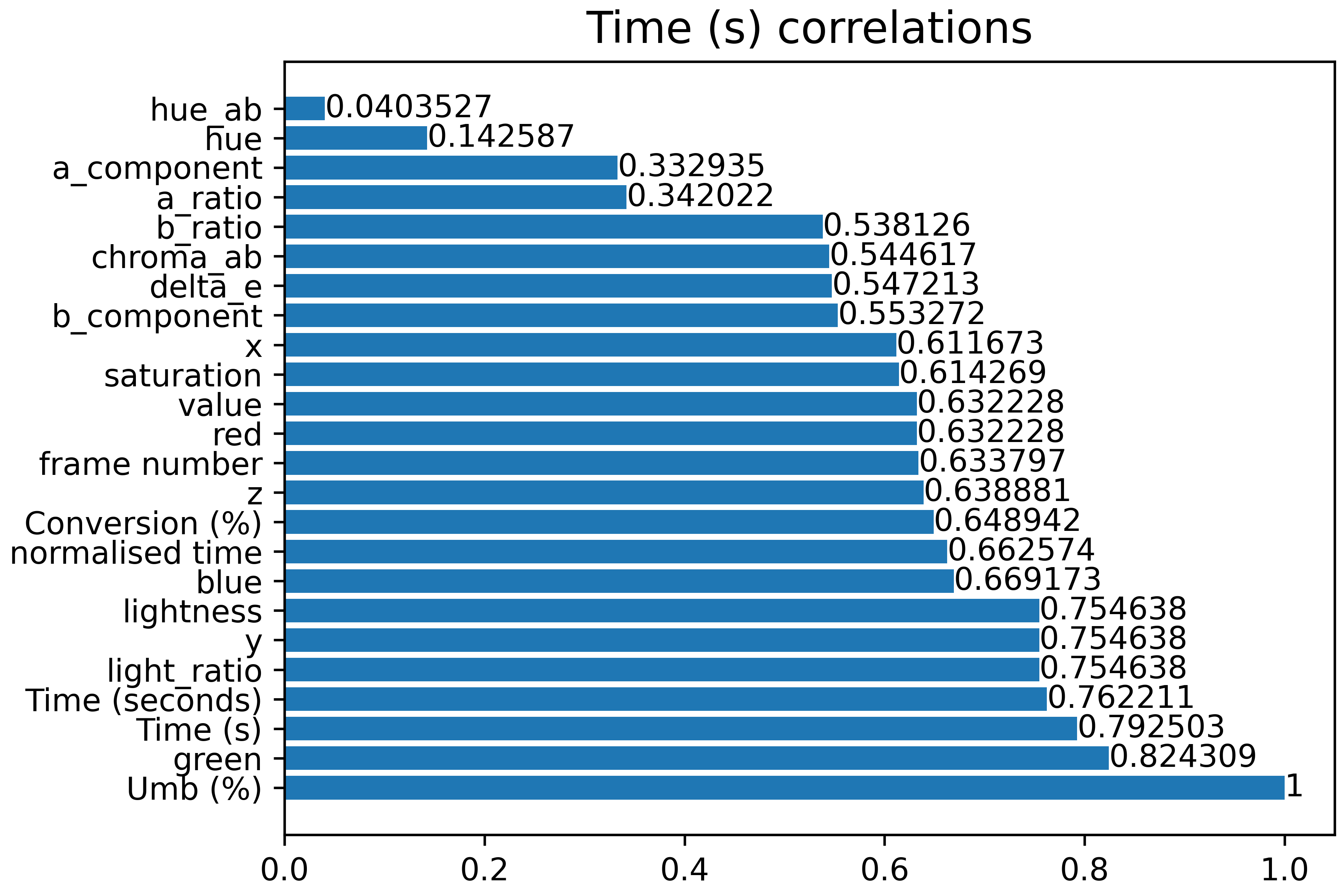

Supplement: Supplementary file 3 — Supporting Information [file ANIE-64-e202413395-s003.zip › Supporting Info - Machine readable data part 2/Figure 10 - esterification and mutual information/Mutual Information and Regression outputs/Mutual information charts/Correlations for Time (s).png]

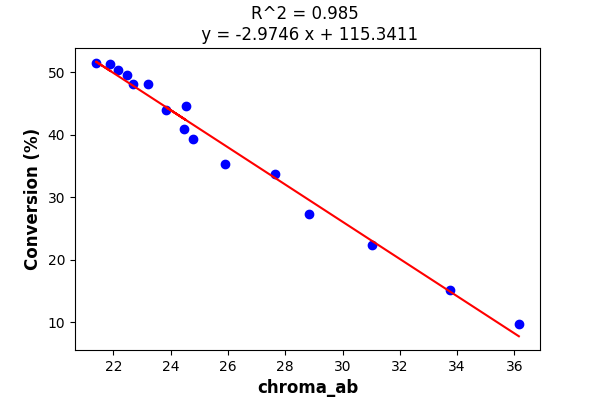

Supplement: Supplementary file 3 — Supporting Information [file ANIE-64-e202413395-s003.zip › Supporting Info - Machine readable data part 2/Figure 10 - esterification and mutual information/Mutual Information and Regression outputs/Regression charts/Regression_chroma_ab.png]

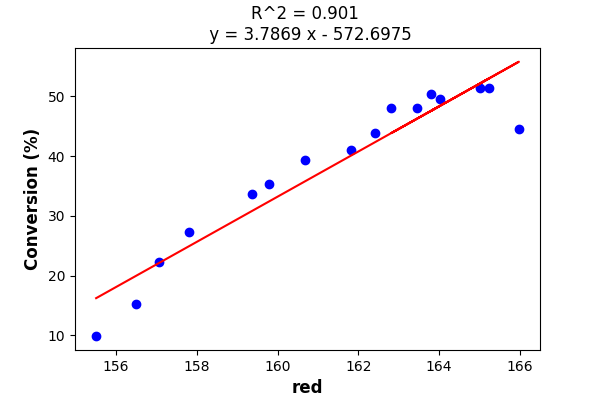

Supplement: Supplementary file 3 — Supporting Information [file ANIE-64-e202413395-s003.zip › Supporting Info - Machine readable data part 2/Figure 10 - esterification and mutual information/Mutual Information and Regression outputs/Regression charts/Regression_red.png]

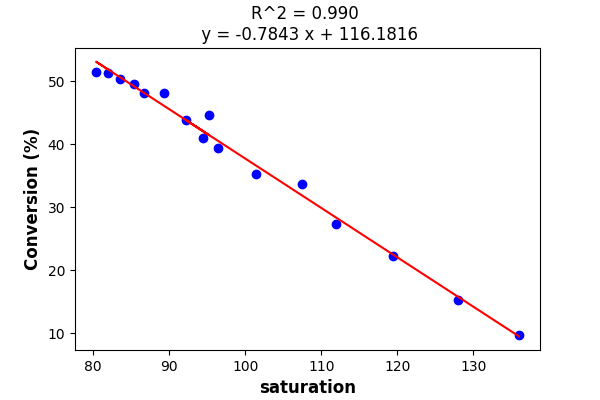

Supplement: Supplementary file 3 — Supporting Information [file ANIE-64-e202413395-s003.zip › Supporting Info - Machine readable data part 2/Figure 10 - esterification and mutual information/Mutual Information and Regression outputs/Regression charts/Regression_saturation.png]

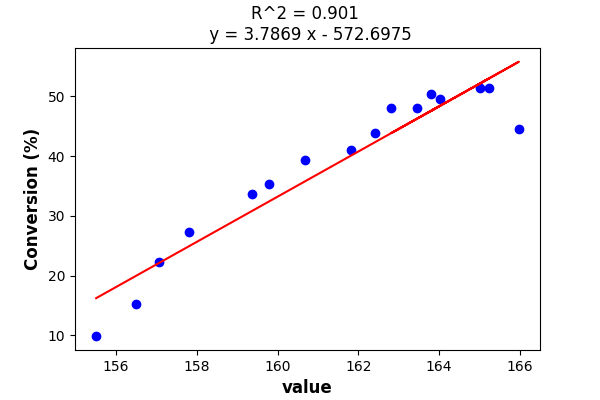

Supplement: Supplementary file 3 — Supporting Information [file ANIE-64-e202413395-s003.zip › Supporting Info - Machine readable data part 2/Figure 10 - esterification and mutual information/Mutual Information and Regression outputs/Regression charts/Regression_value.png]

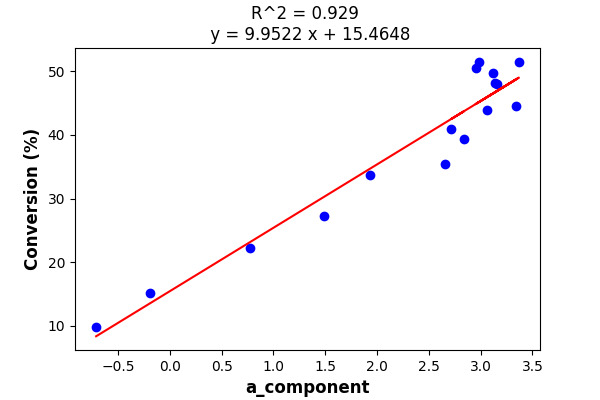

Supplement: Supplementary file 3 — Supporting Information [file ANIE-64-e202413395-s003.zip › Supporting Info - Machine readable data part 2/Figure 10 - esterification and mutual information/Mutual Information and Regression outputs/Regression charts/Regression_a_component.png]

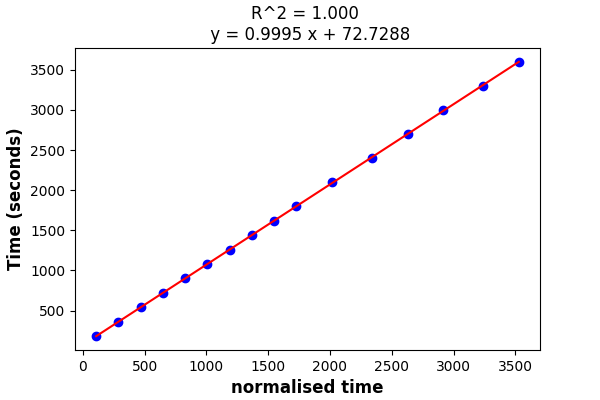

Supplement: Supplementary file 3 — Supporting Information [file ANIE-64-e202413395-s003.zip › Supporting Info - Machine readable data part 2/Figure 10 - esterification and mutual information/Mutual Information and Regression outputs/Regression charts/Regression_normalised time.png]

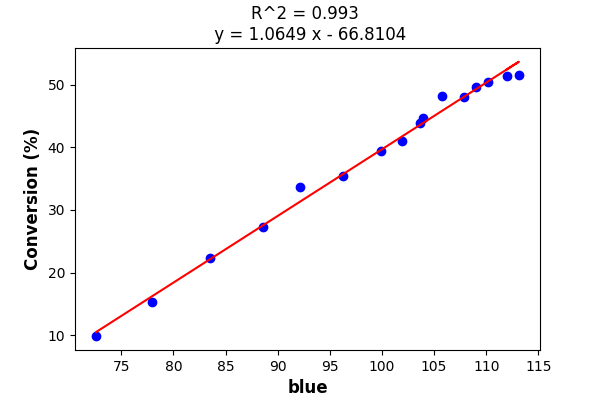

Supplement: Supplementary file 3 — Supporting Information [file ANIE-64-e202413395-s003.zip › Supporting Info - Machine readable data part 2/Figure 10 - esterification and mutual information/Mutual Information and Regression outputs/Regression charts/Regression_blue.png]

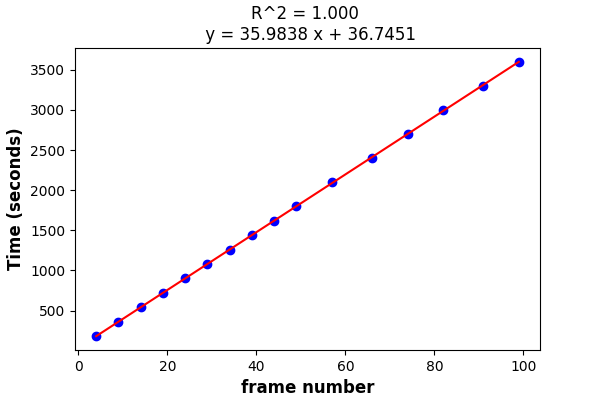

Supplement: Supplementary file 3 — Supporting Information [file ANIE-64-e202413395-s003.zip › Supporting Info - Machine readable data part 2/Figure 10 - esterification and mutual information/Mutual Information and Regression outputs/Regression charts/Regression_frame number.png]

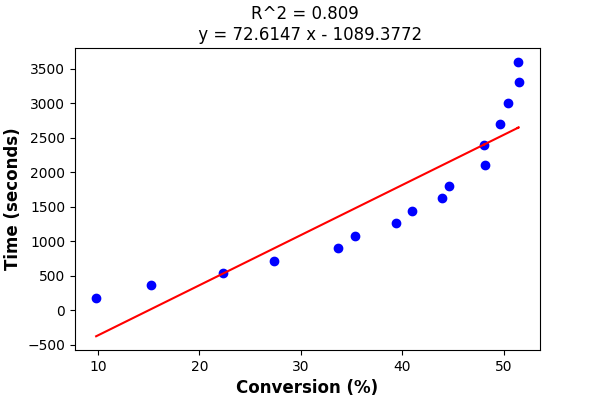

Supplement: Supplementary file 3 — Supporting Information [file ANIE-64-e202413395-s003.zip › Supporting Info - Machine readable data part 2/Figure 10 - esterification and mutual information/Mutual Information and Regression outputs/Regression charts/Regression_Conversion (%).png]

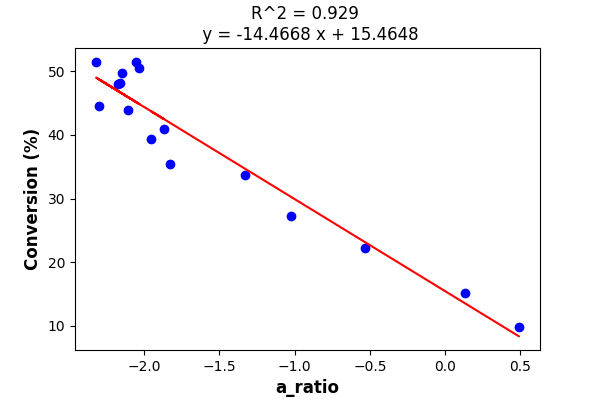

Supplement: Supplementary file 3 — Supporting Information [file ANIE-64-e202413395-s003.zip › Supporting Info - Machine readable data part 2/Figure 10 - esterification and mutual information/Mutual Information and Regression outputs/Regression charts/Regression_a_ratio.png]

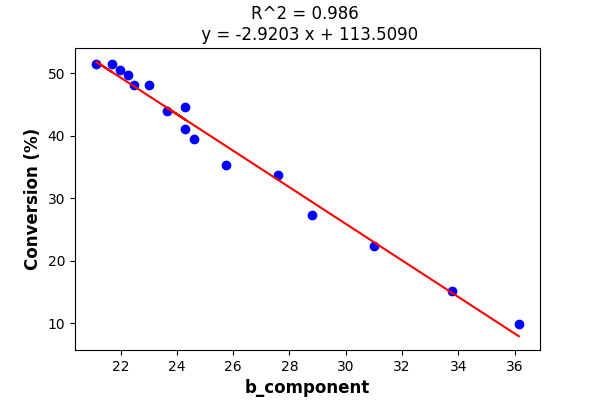

Supplement: Supplementary file 3 — Supporting Information [file ANIE-64-e202413395-s003.zip › Supporting Info - Machine readable data part 2/Figure 10 - esterification and mutual information/Mutual Information and Regression outputs/Regression charts/Regression_b_component.png]

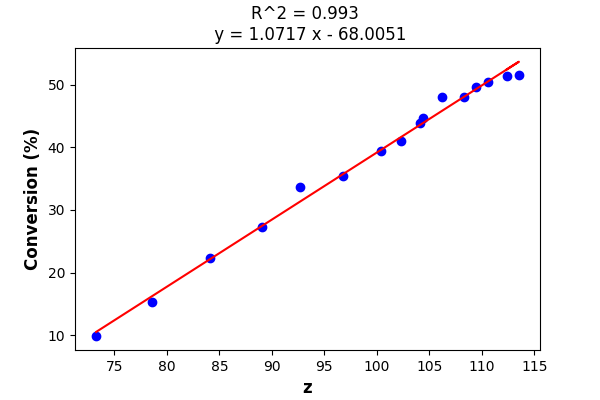

Supplement: Supplementary file 3 — Supporting Information [file ANIE-64-e202413395-s003.zip › Supporting Info - Machine readable data part 2/Figure 10 - esterification and mutual information/Mutual Information and Regression outputs/Regression charts/Regression_z.png]

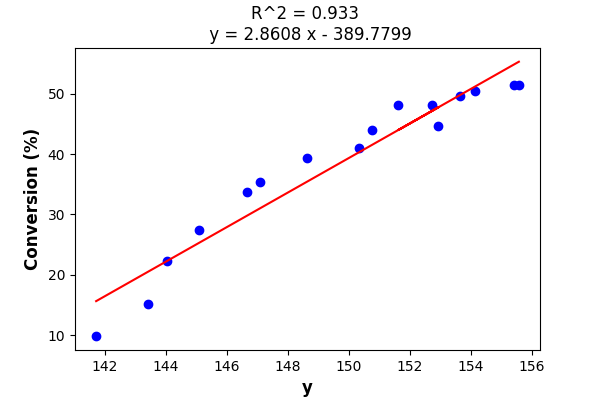

Supplement: Supplementary file 3 — Supporting Information [file ANIE-64-e202413395-s003.zip › Supporting Info - Machine readable data part 2/Figure 10 - esterification and mutual information/Mutual Information and Regression outputs/Regression charts/Regression_y.png]

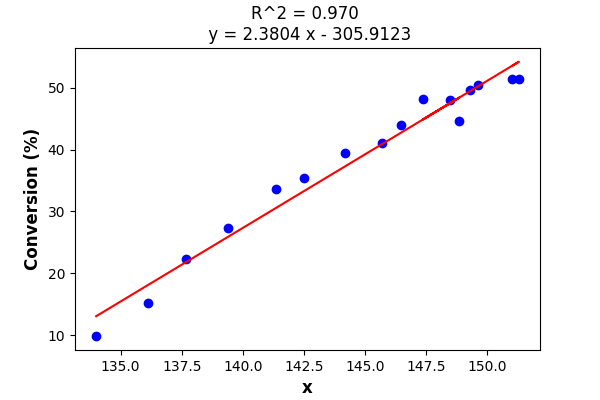

Supplement: Supplementary file 3 — Supporting Information [file ANIE-64-e202413395-s003.zip › Supporting Info - Machine readable data part 2/Figure 10 - esterification and mutual information/Mutual Information and Regression outputs/Regression charts/Regression_x.png]

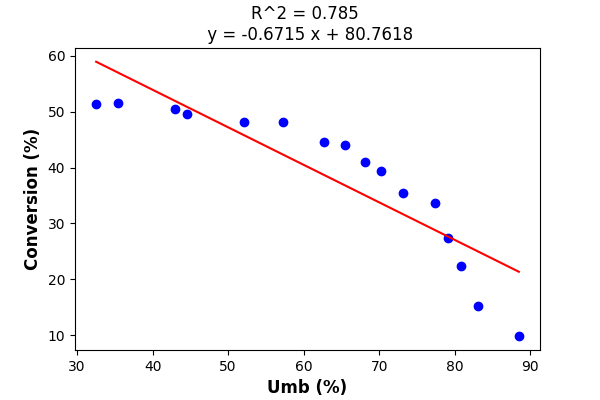

Supplement: Supplementary file 3 — Supporting Information [file ANIE-64-e202413395-s003.zip › Supporting Info - Machine readable data part 2/Figure 10 - esterification and mutual information/Mutual Information and Regression outputs/Regression charts/Regression_Umb (%).png]

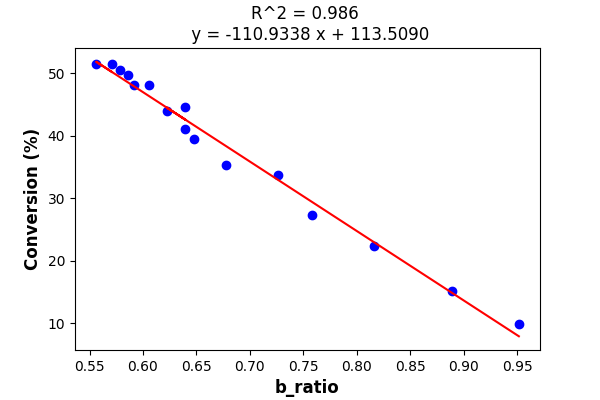

Supplement: Supplementary file 3 — Supporting Information [file ANIE-64-e202413395-s003.zip › Supporting Info - Machine readable data part 2/Figure 10 - esterification and mutual information/Mutual Information and Regression outputs/Regression charts/Regression_b_ratio.png]

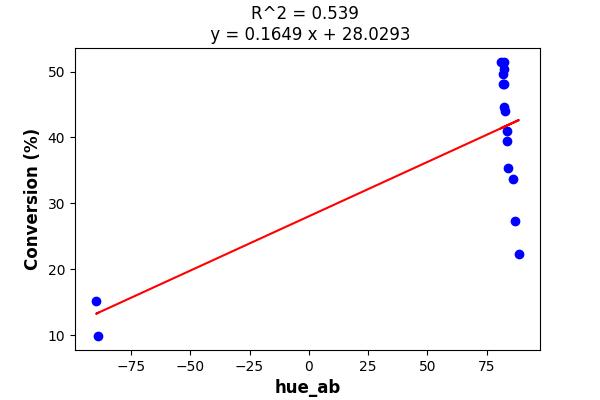

Supplement: Supplementary file 3 — Supporting Information [file ANIE-64-e202413395-s003.zip › Supporting Info - Machine readable data part 2/Figure 10 - esterification and mutual information/Mutual Information and Regression outputs/Regression charts/Regression_hue_ab.png]

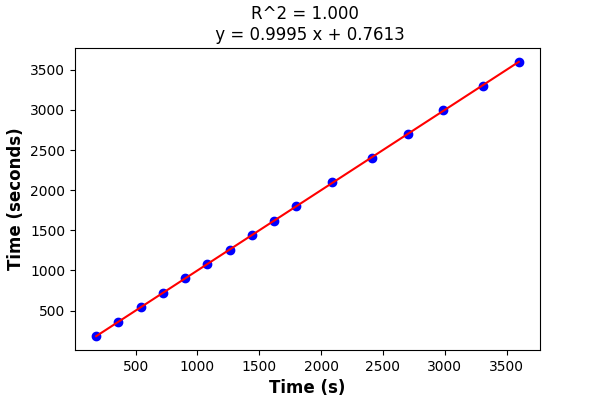

Supplement: Supplementary file 3 — Supporting Information [file ANIE-64-e202413395-s003.zip › Supporting Info - Machine readable data part 2/Figure 10 - esterification and mutual information/Mutual Information and Regression outputs/Regression charts/Regression_Time (s).png]
