# Supplementary figures and images for: Parallel and High Throughput Reaction Monitoring with Computer Vision (part 1 of 3)
Source: Angew Chem Int Ed Engl. 2024 Oct 31;64(1):e202413395. doi: 10.1002/anie.202413395 (PMC11701362; doi:10.1002/anie.202413395)

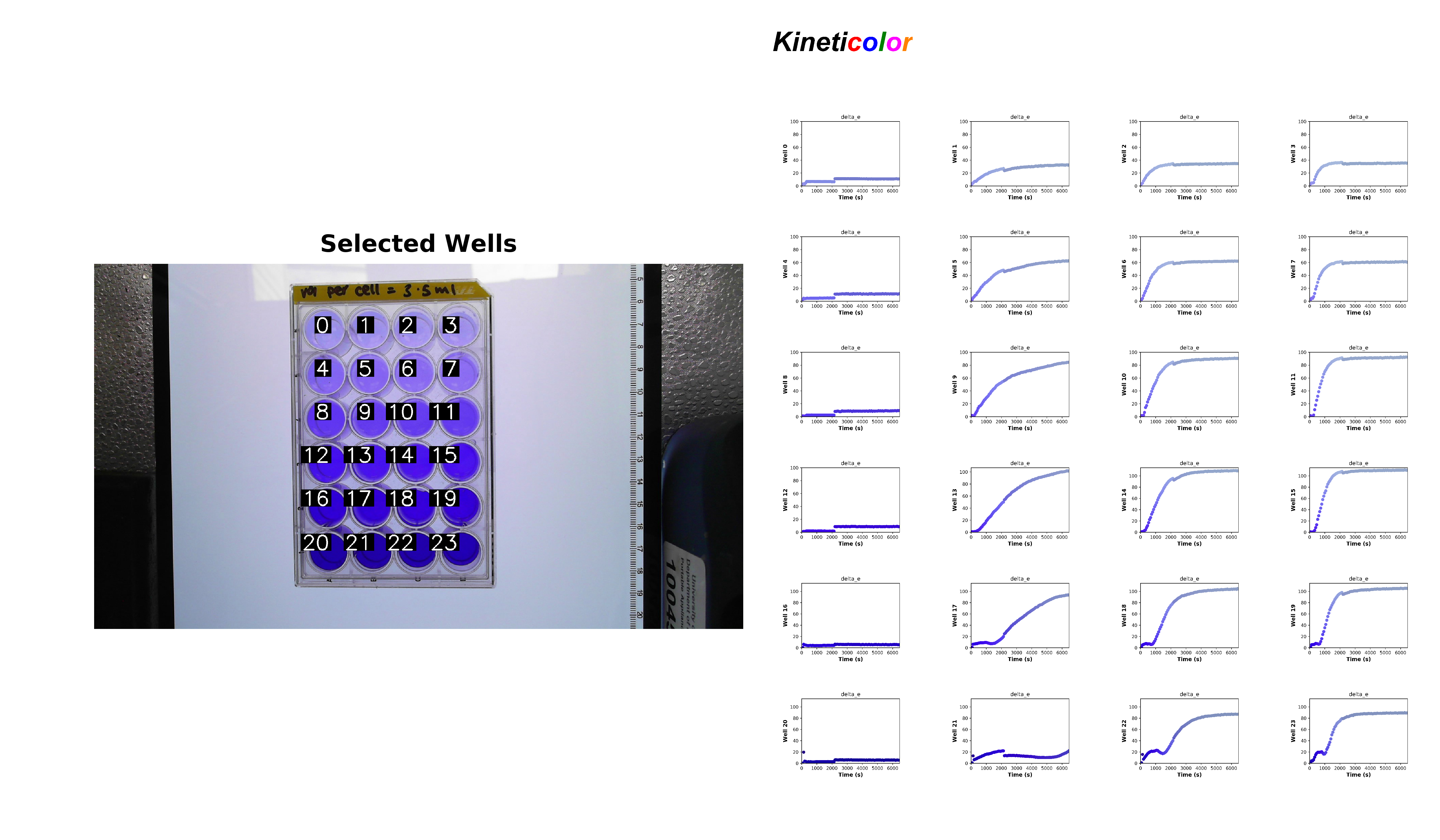

Supplement: Supplementary file 2 — Supporting Information [file ANIE-64-e202413395-s002.zip › Supporting Info - Machine readable data part 1/Table 1- crystal violet HTE analysis/Wellplate_TILE_delta_e.PNG]

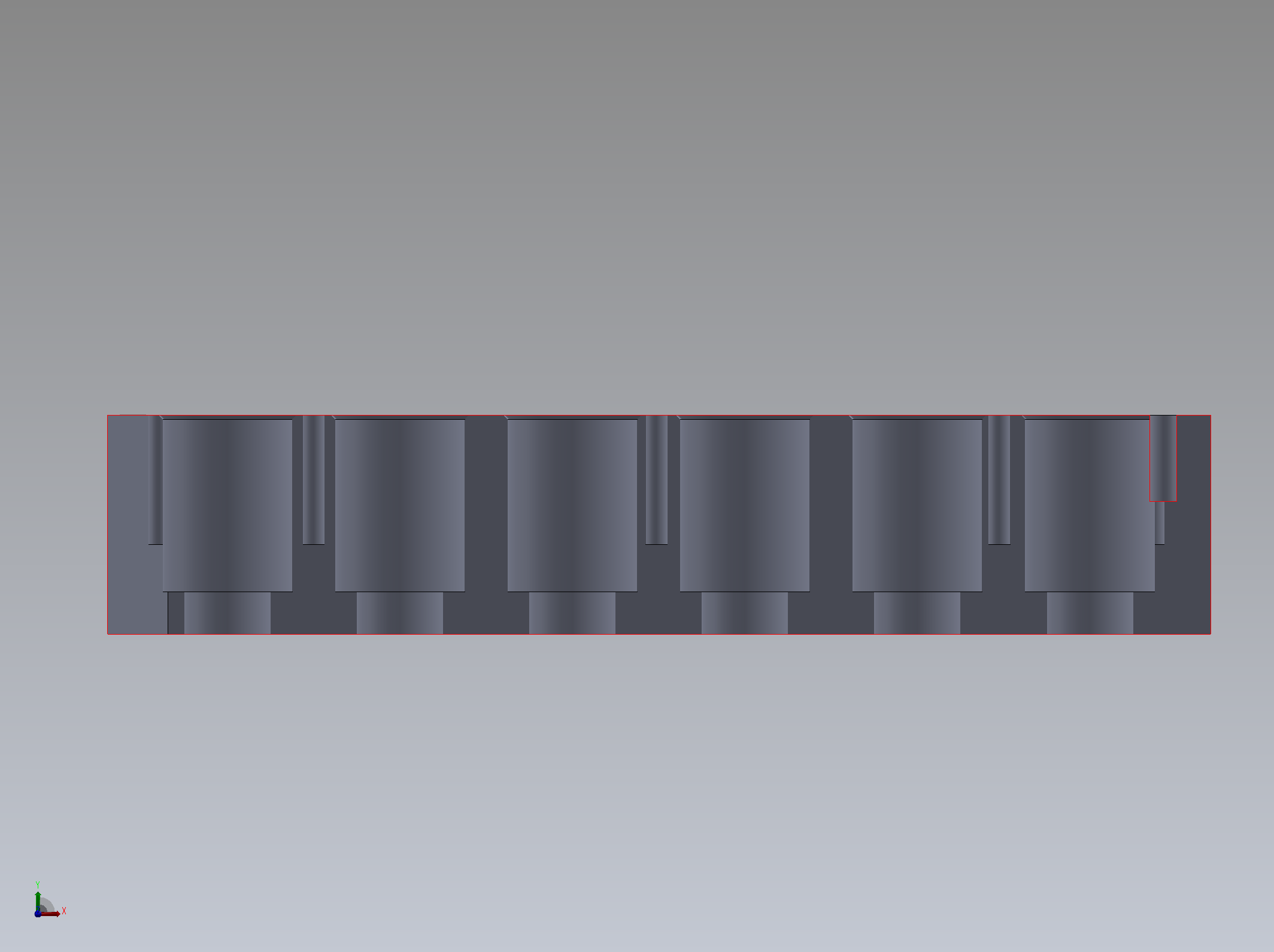

Supplement: Supplementary file 2 — Supporting Information [file ANIE-64-e202413395-s002.zip › Supporting Info - Machine readable data part 1/Figure 6 - CAD files for bespoke well plate/Henry 24015 CH_cross section.png]

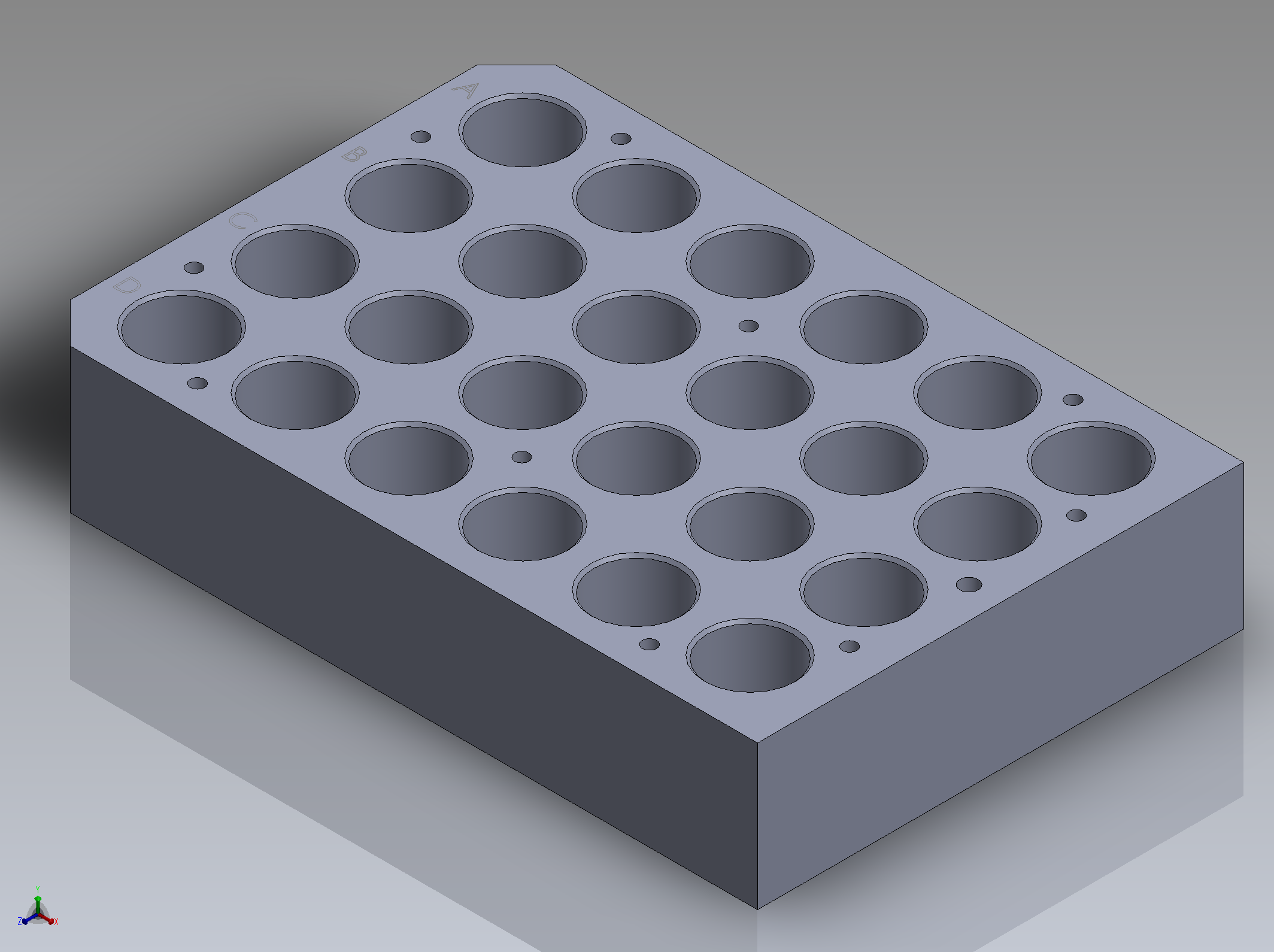

Supplement: Supplementary file 2 — Supporting Information [file ANIE-64-e202413395-s002.zip › Supporting Info - Machine readable data part 1/Figure 6 - CAD files for bespoke well plate/Henry 24015 CH_iso.png]

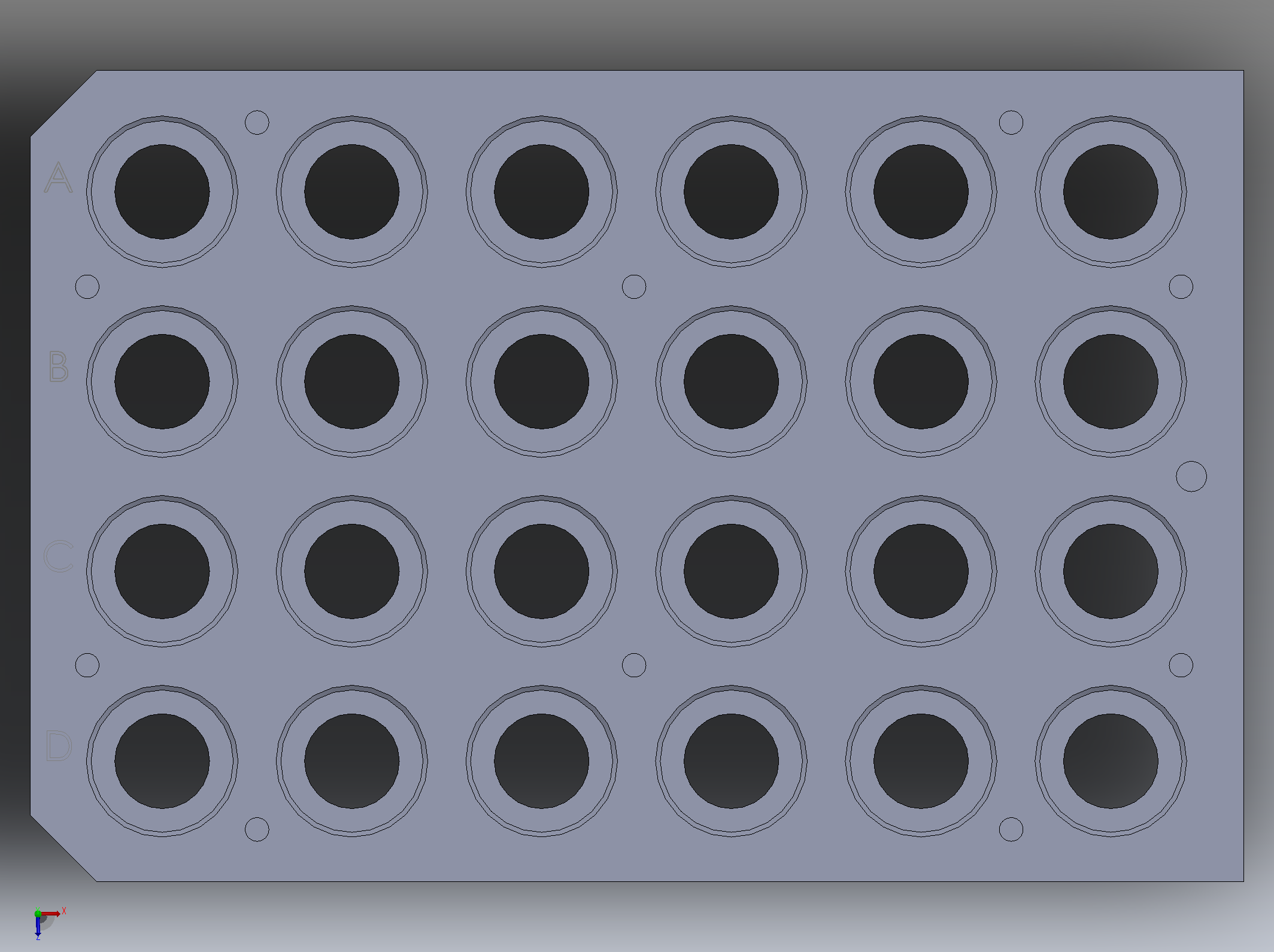

Supplement: Supplementary file 2 — Supporting Information [file ANIE-64-e202413395-s002.zip › Supporting Info - Machine readable data part 1/Figure 6 - CAD files for bespoke well plate/Henry 24015 CH.png]

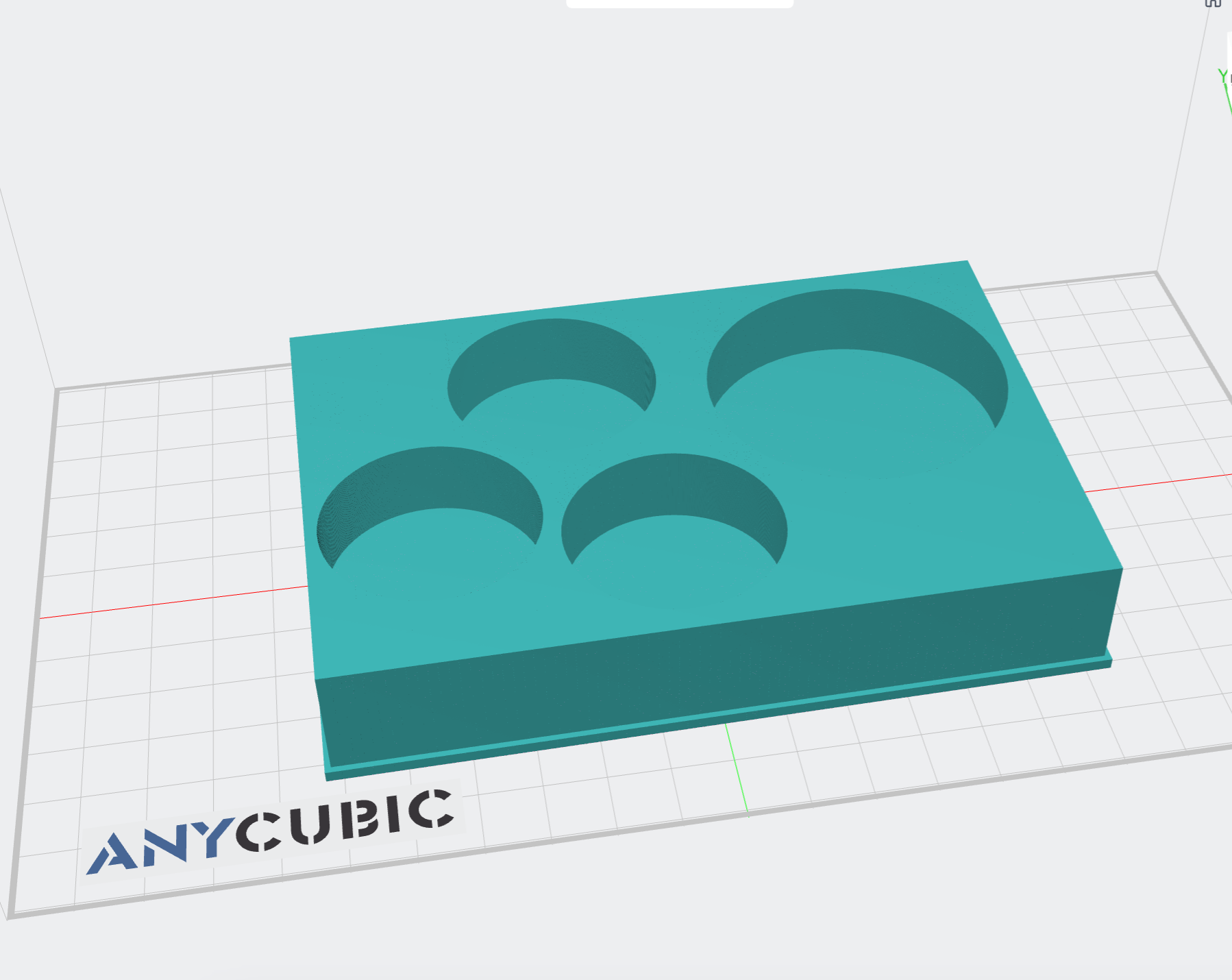

Supplement: Supplementary file 2 — Supporting Information [file ANIE-64-e202413395-s002.zip › Supporting Info - Machine readable data part 1/3D printouts/custom beaker holder.png]

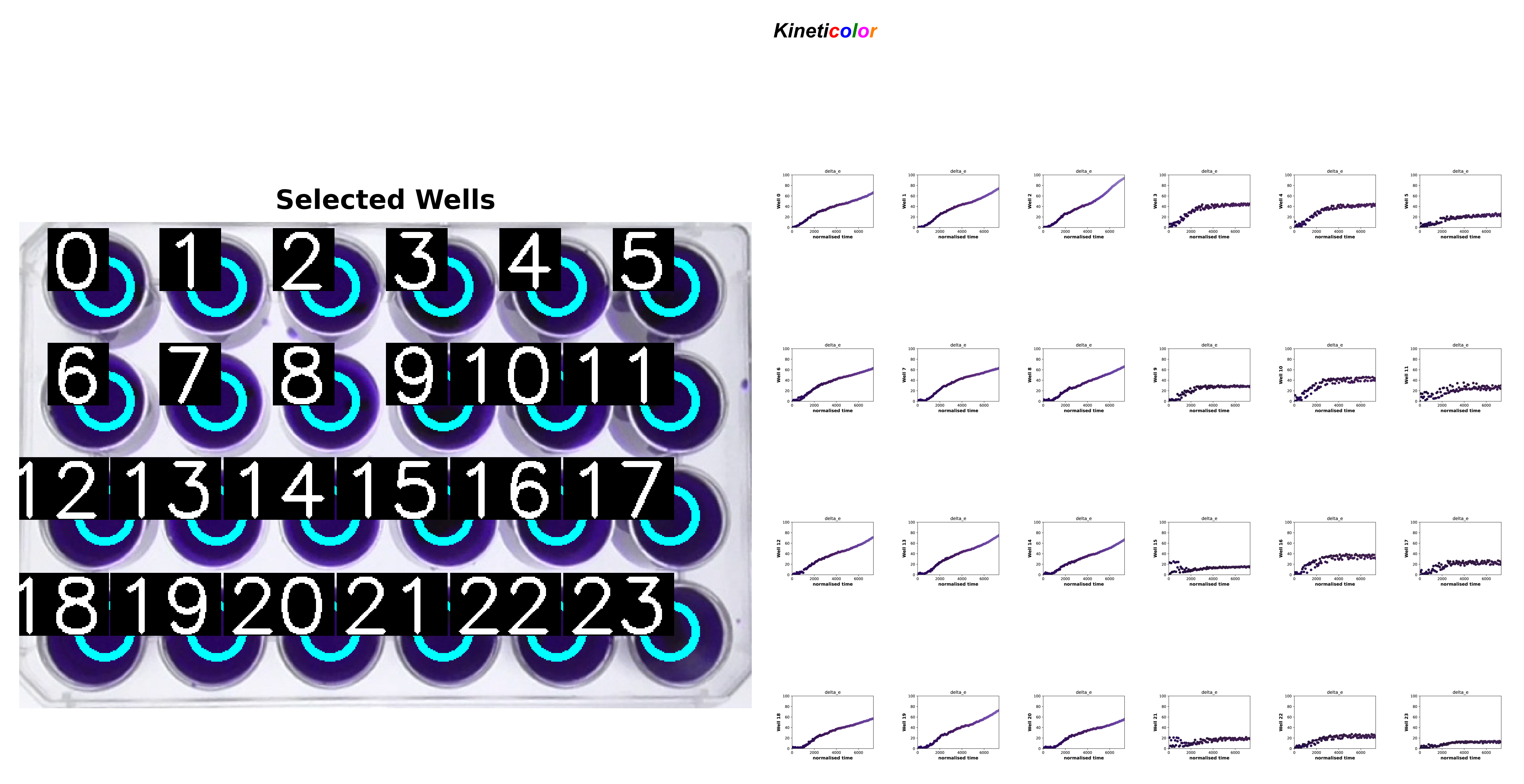

Supplement: Supplementary file 2 — Supporting Information [file ANIE-64-e202413395-s002.zip › Supporting Info - Machine readable data part 1/Figure 9 - crystal violet mixing analysis/Kineticolor outputs/delta_e over normalised time Summary CV Mixing.PNG]

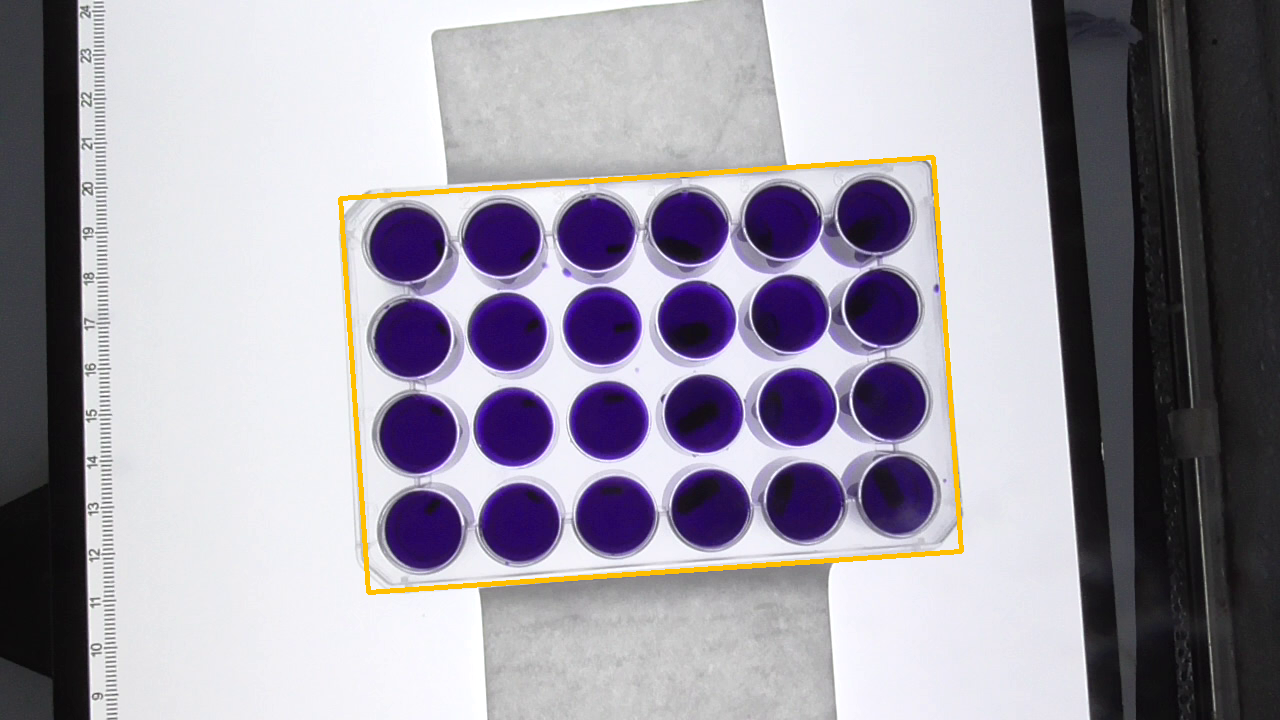

Supplement: Supplementary file 2 — Supporting Information [file ANIE-64-e202413395-s002.zip › Supporting Info - Machine readable data part 1/Figure 9 - crystal violet mixing analysis/Kineticolor outputs/ROI.png]

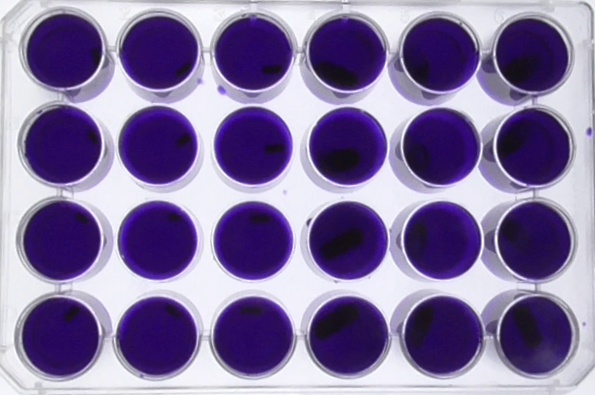

Supplement: Supplementary file 2 — Supporting Information [file ANIE-64-e202413395-s002.zip › Supporting Info - Machine readable data part 1/Figure 9 - crystal violet mixing analysis/Kineticolor outputs/first_frame.jpg]

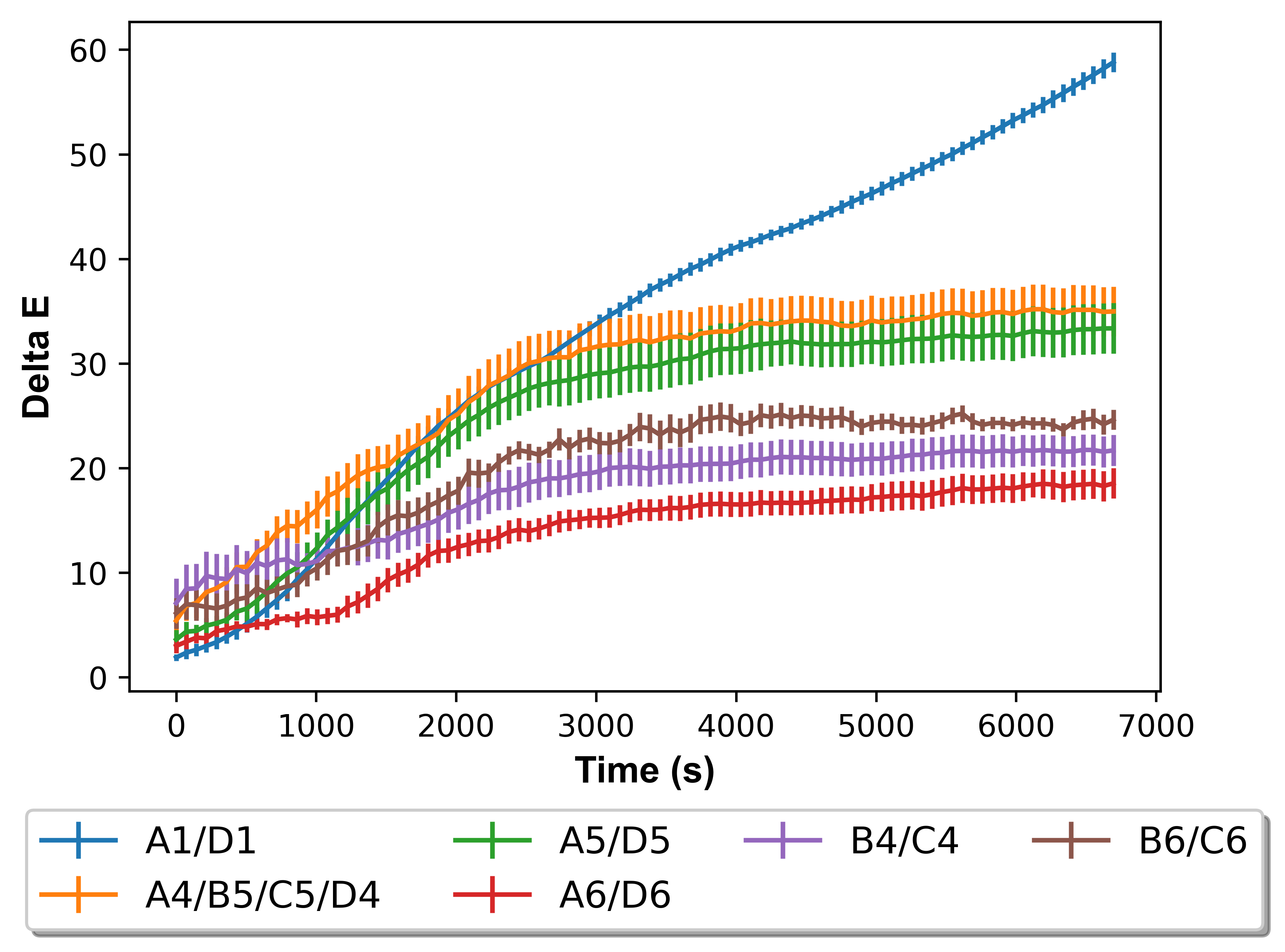

Supplement: Supplementary file 2 — Supporting Information [file ANIE-64-e202413395-s002.zip › Supporting Info - Machine readable data part 1/Figure 9 - crystal violet mixing analysis/Kineticolor outputs/stirrer-24plot.png]

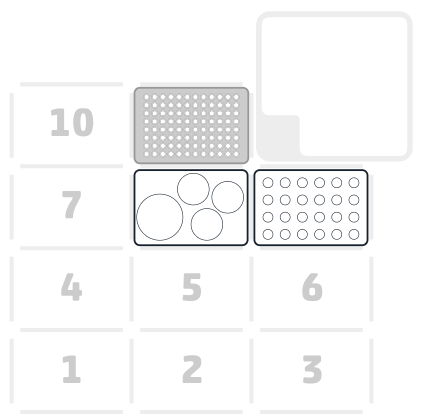

Supplement: Supplementary file 2 — Supporting Information [file ANIE-64-e202413395-s002.zip › Supporting Info - Machine readable data part 1/Figure 9 - crystal violet mixing analysis/OT-2 Liquid Handling Robot Files/Uniform Crystal Violet protocol layout.png]

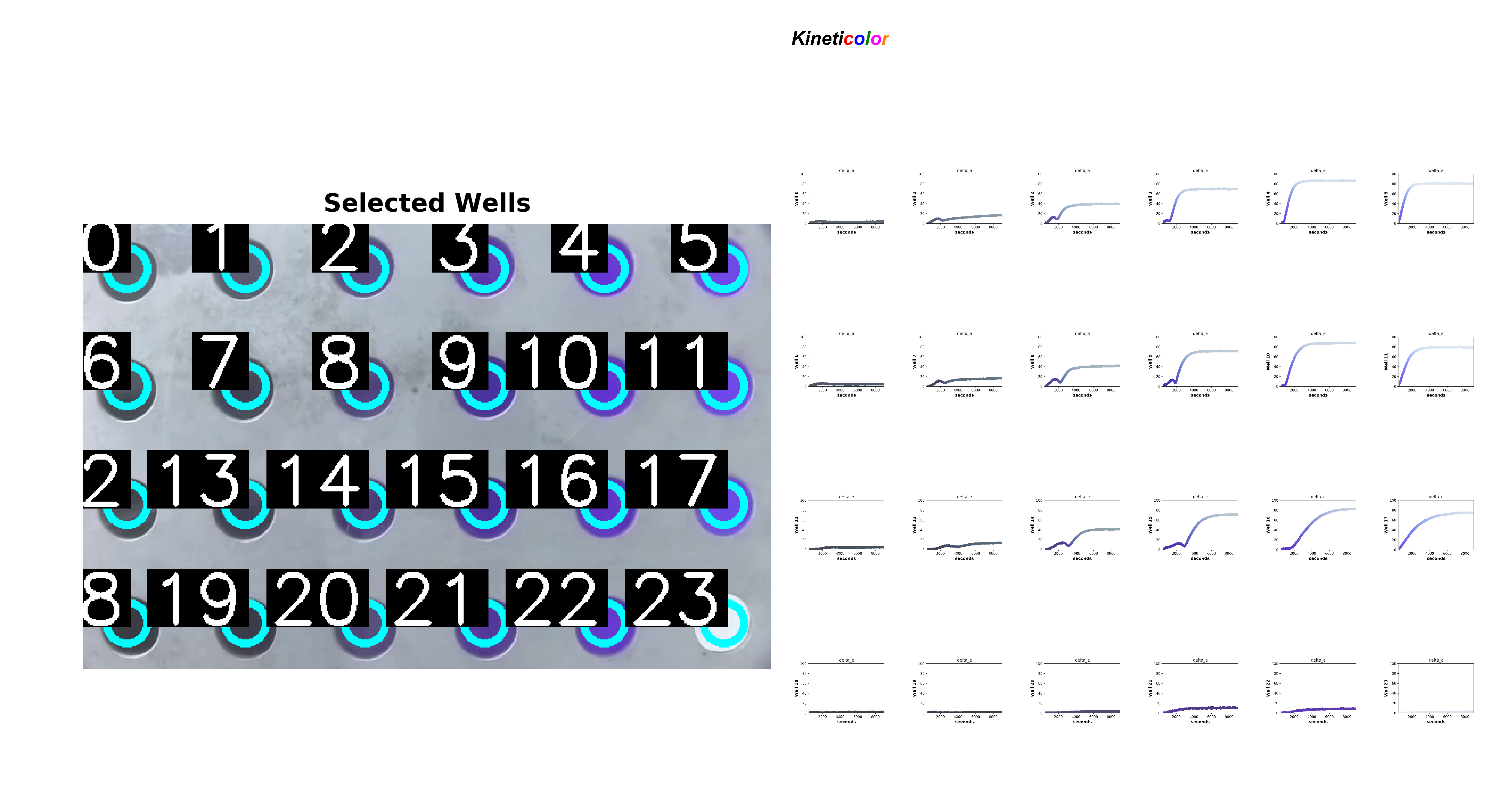

Supplement: Supplementary file 2 — Supporting Information [file ANIE-64-e202413395-s002.zip › Supporting Info - Machine readable data part 1/Figure 7 - investigating camera lenses for well plate filming/Zoom lens - good quality/Wellplate_TILE_delta_e over seconds.PNG]

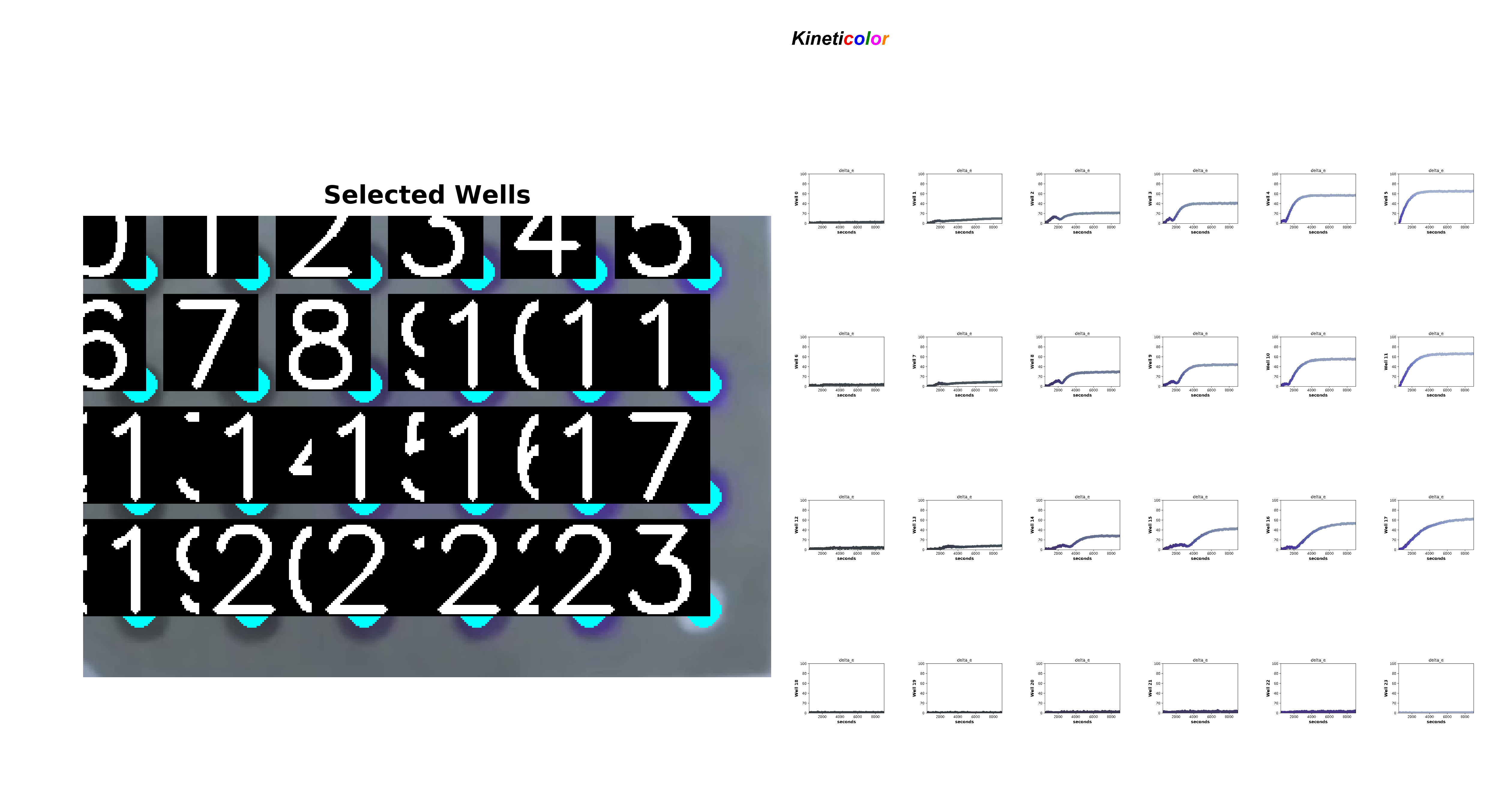

Supplement: Supplementary file 2 — Supporting Information [file ANIE-64-e202413395-s002.zip › Supporting Info - Machine readable data part 1/Figure 7 - investigating camera lenses for well plate filming/CCTV lens - poor quality/Wellplate_TILE_delta_e over seconds.PNG]

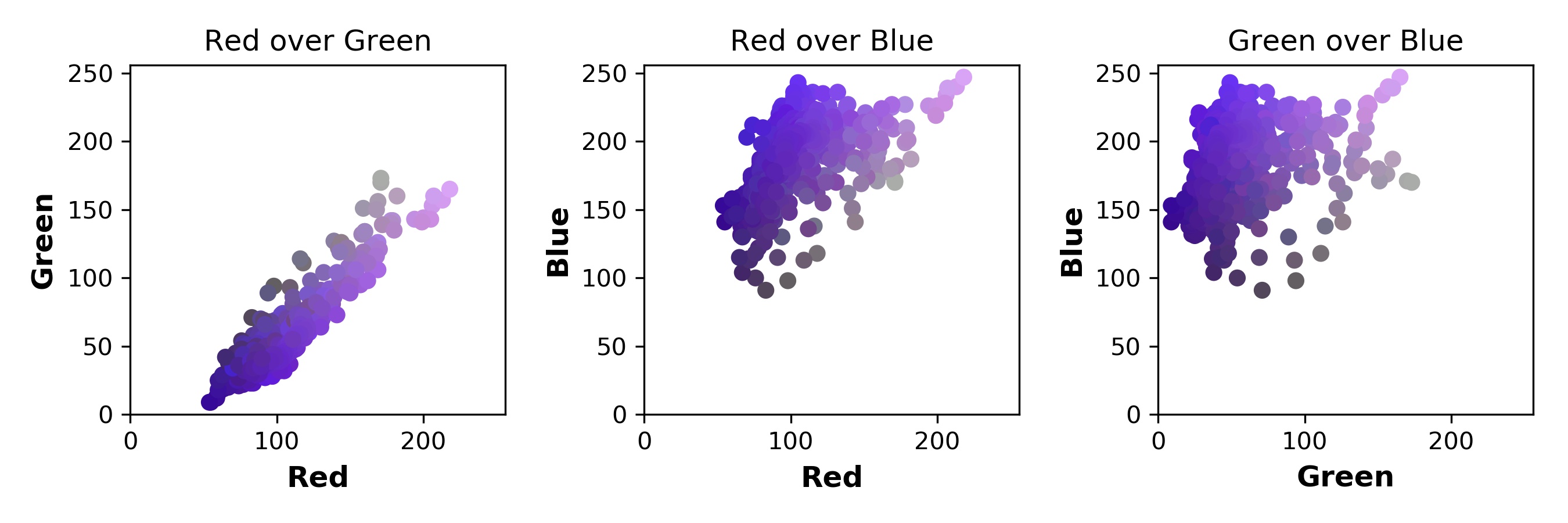

Supplement: Supplementary file 2 — Supporting Information [file ANIE-64-e202413395-s002.zip › Supporting Info - Machine readable data part 1/Figure 4 - glare analysis/24_below_SIanal__6/rgb.png]

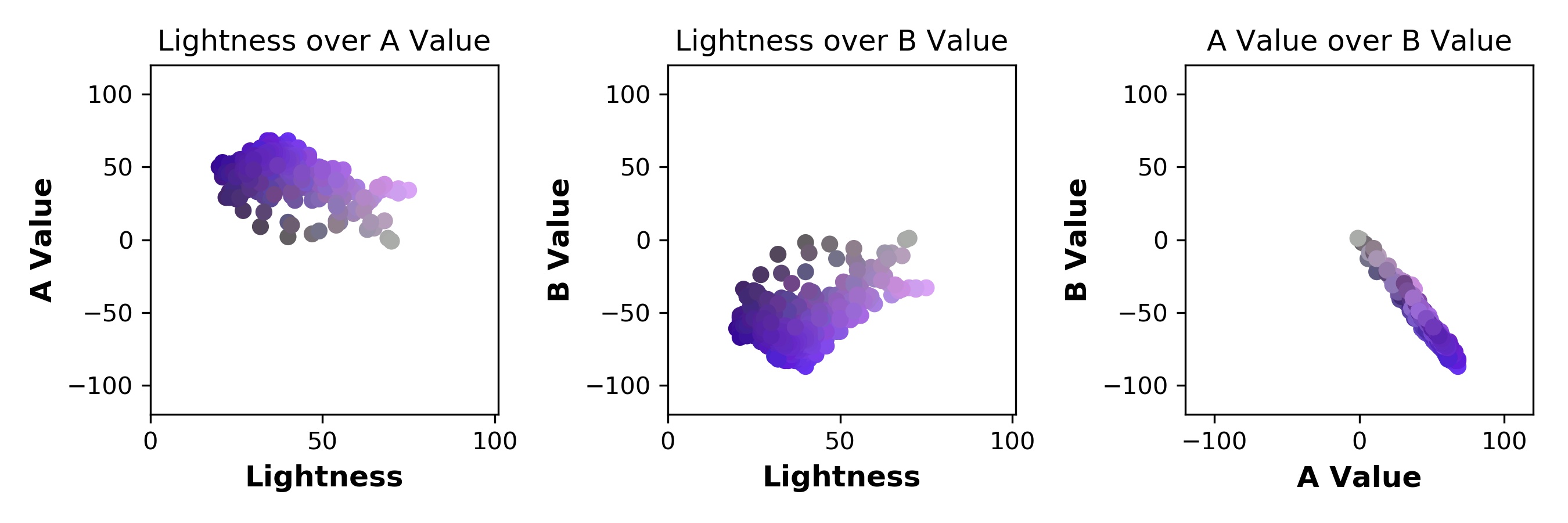

Supplement: Supplementary file 2 — Supporting Information [file ANIE-64-e202413395-s002.zip › Supporting Info - Machine readable data part 1/Figure 4 - glare analysis/24_below_SIanal__6/lab.png]

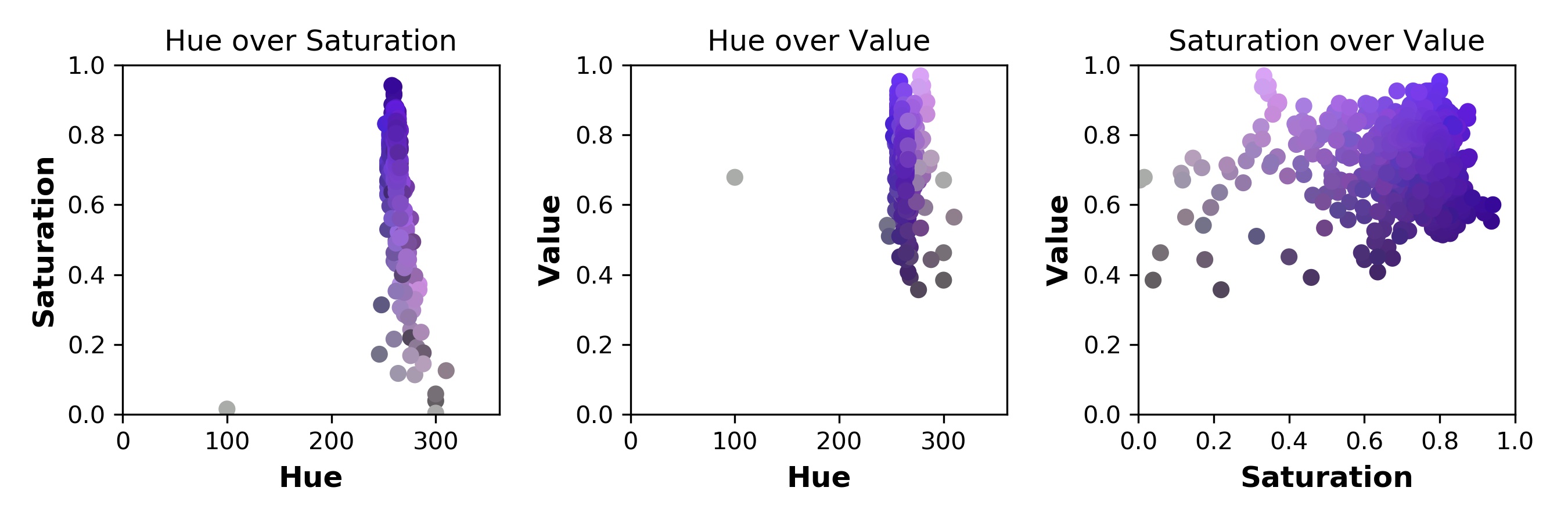

Supplement: Supplementary file 2 — Supporting Information [file ANIE-64-e202413395-s002.zip › Supporting Info - Machine readable data part 1/Figure 4 - glare analysis/24_below_SIanal__6/hsv.png]

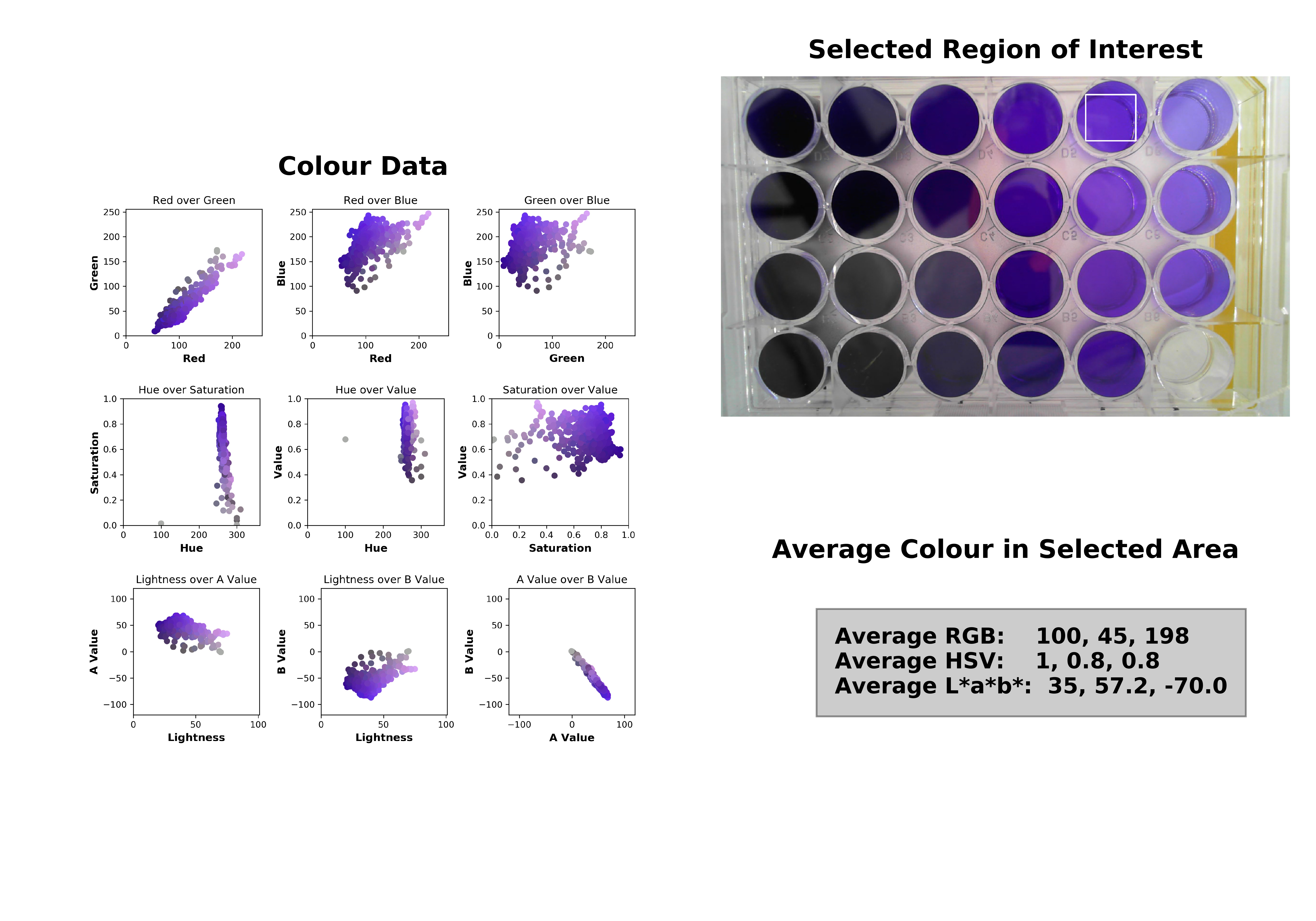

Supplement: Supplementary file 2 — Supporting Information [file ANIE-64-e202413395-s002.zip › Supporting Info - Machine readable data part 1/Figure 4 - glare analysis/24_below_SIanal__6/TILE_WITH_ROI.PNG]

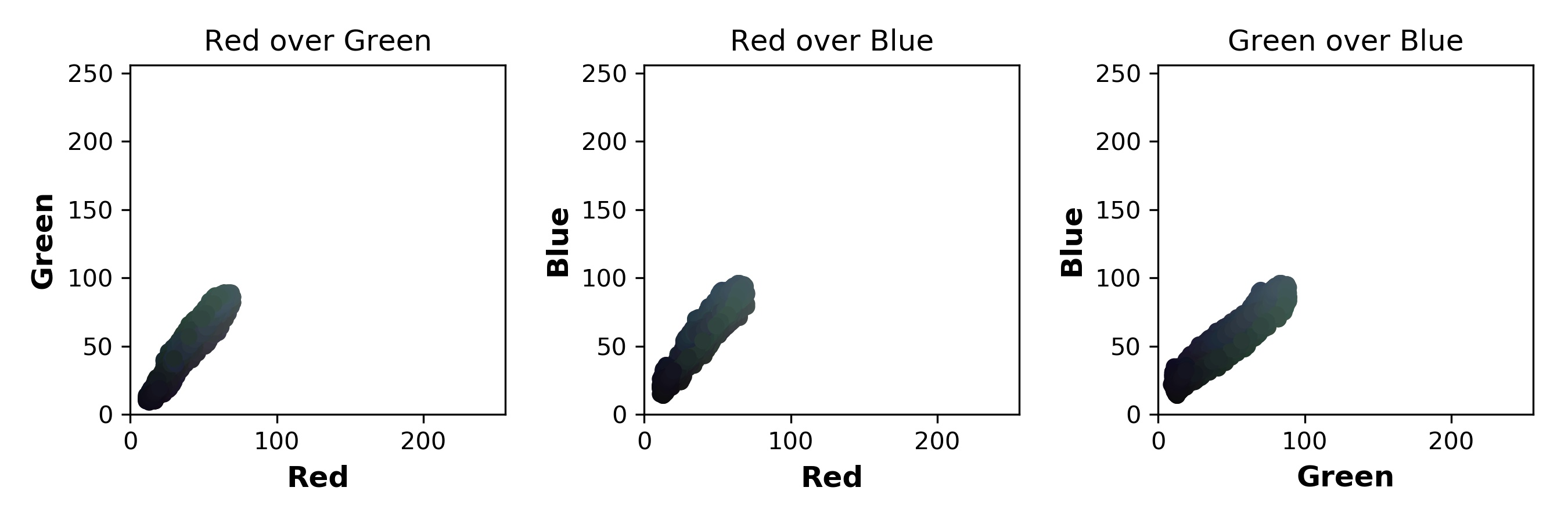

Supplement: Supplementary file 2 — Supporting Information [file ANIE-64-e202413395-s002.zip › Supporting Info - Machine readable data part 1/Figure 4 - glare analysis/24_above_SIanal/rgb.png]

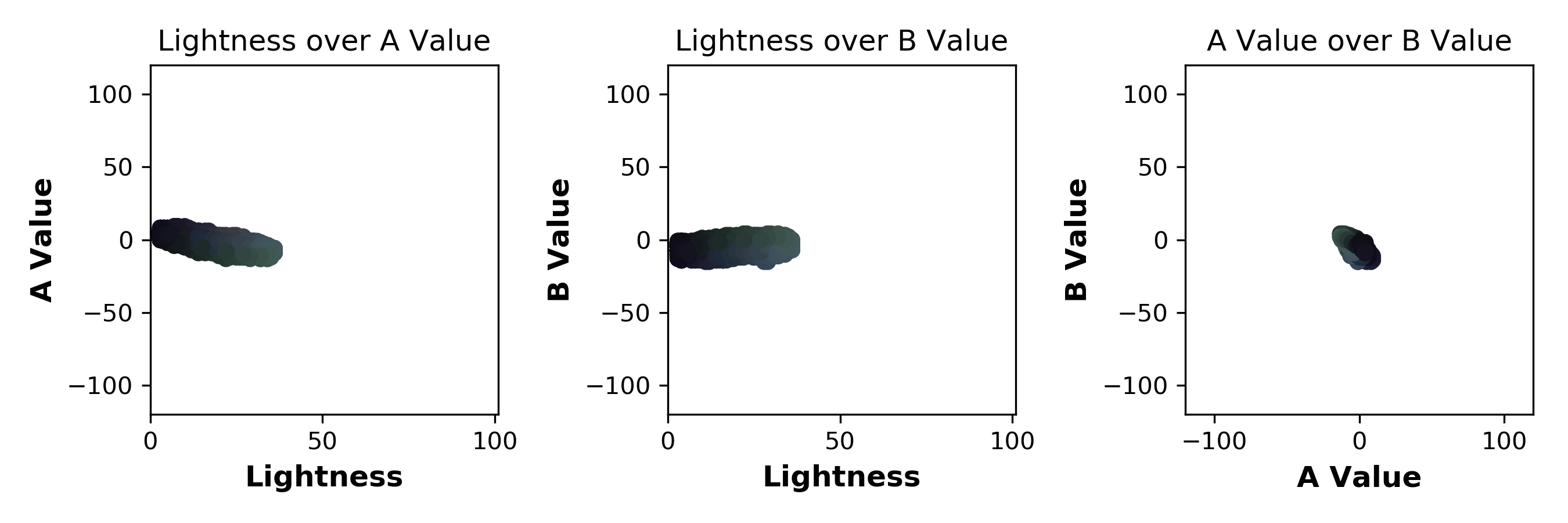

Supplement: Supplementary file 2 — Supporting Information [file ANIE-64-e202413395-s002.zip › Supporting Info - Machine readable data part 1/Figure 4 - glare analysis/24_above_SIanal/lab.png]

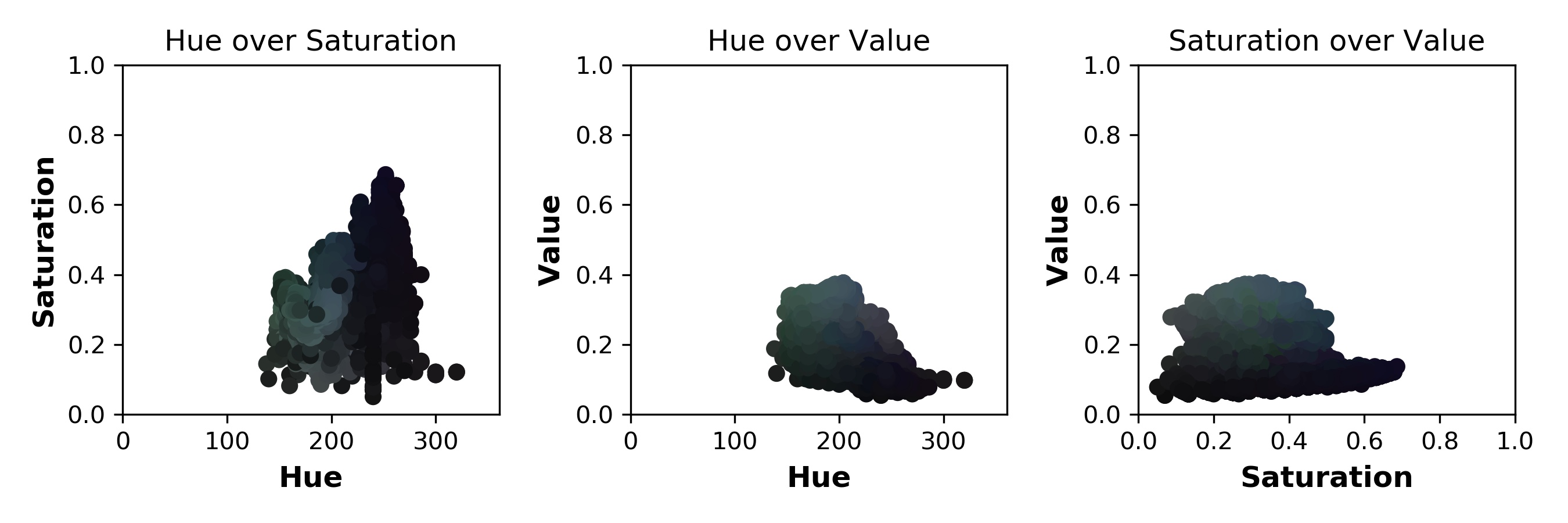

Supplement: Supplementary file 2 — Supporting Information [file ANIE-64-e202413395-s002.zip › Supporting Info - Machine readable data part 1/Figure 4 - glare analysis/24_above_SIanal/hsv.png]

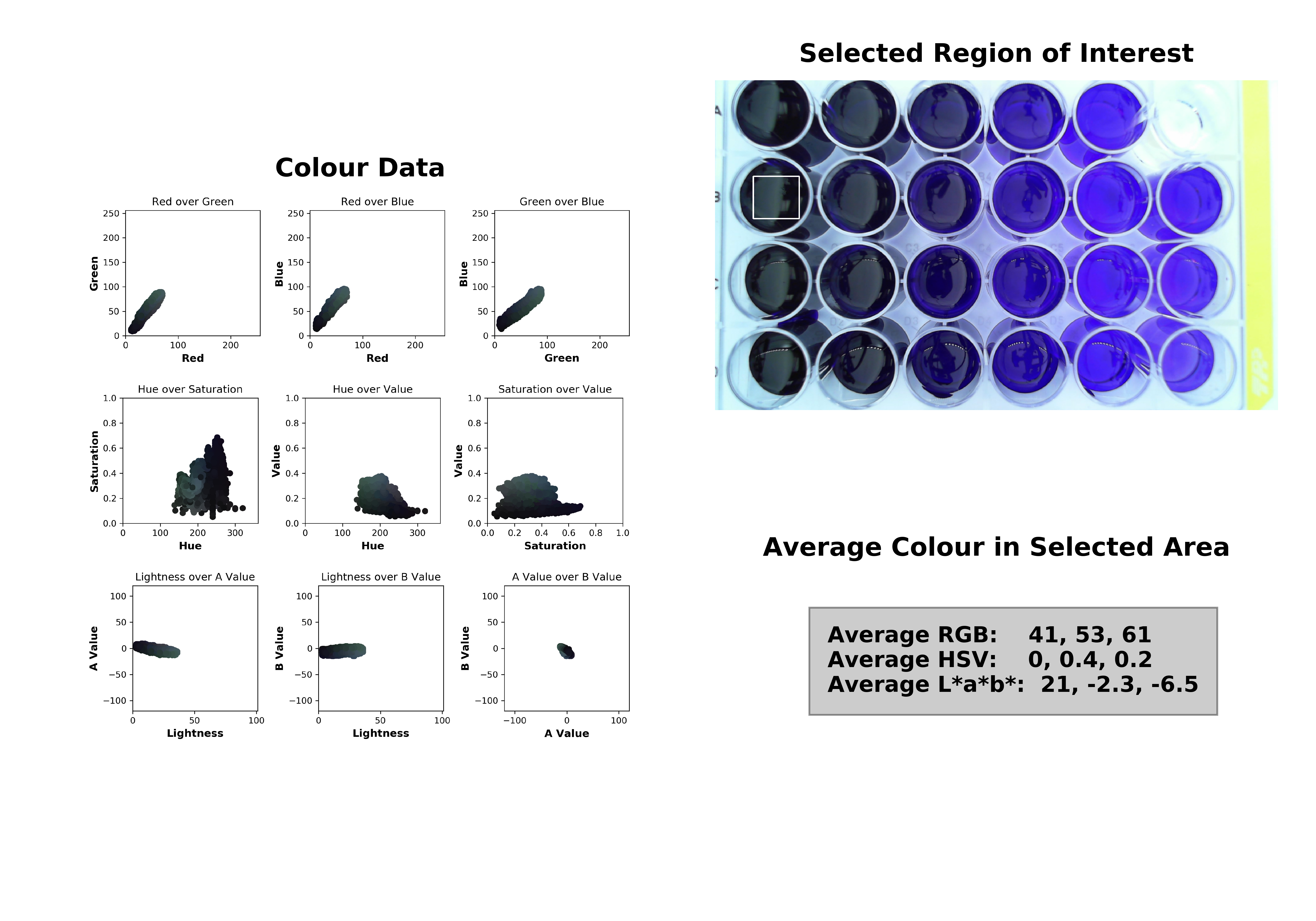

Supplement: Supplementary file 2 — Supporting Information [file ANIE-64-e202413395-s002.zip › Supporting Info - Machine readable data part 1/Figure 4 - glare analysis/24_above_SIanal/TILE_WITH_ROI.PNG]

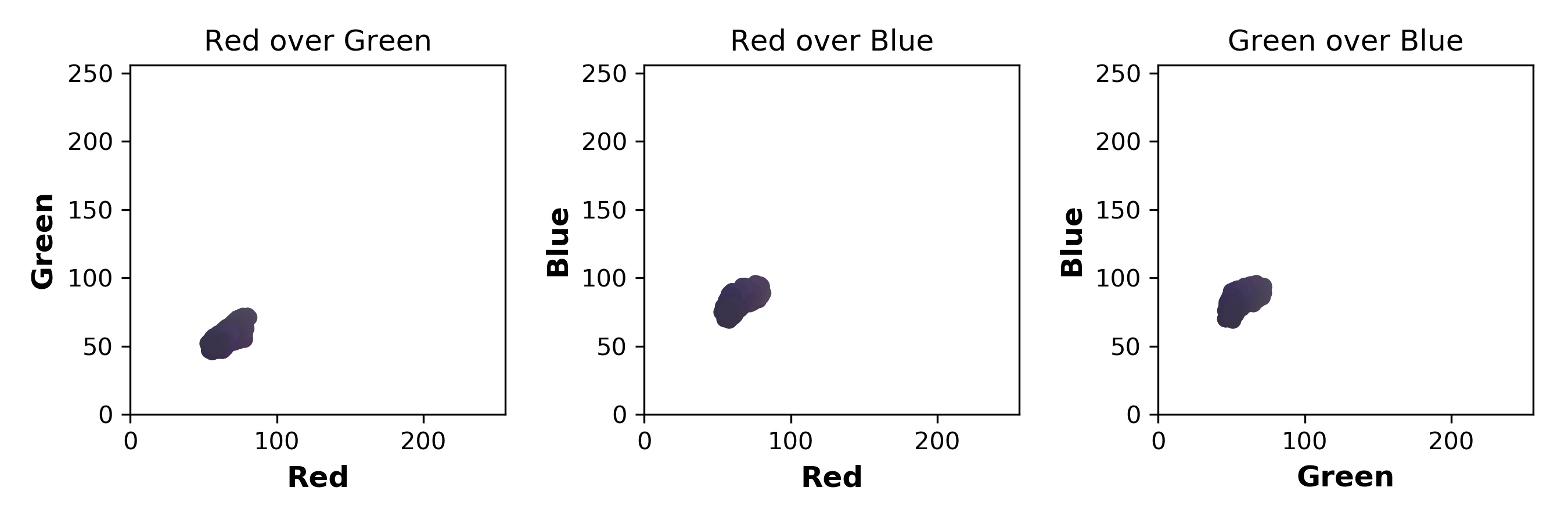

Supplement: Supplementary file 2 — Supporting Information [file ANIE-64-e202413395-s002.zip › Supporting Info - Machine readable data part 1/Figure 4 - glare analysis/24_below_SIanal__1/rgb.png]

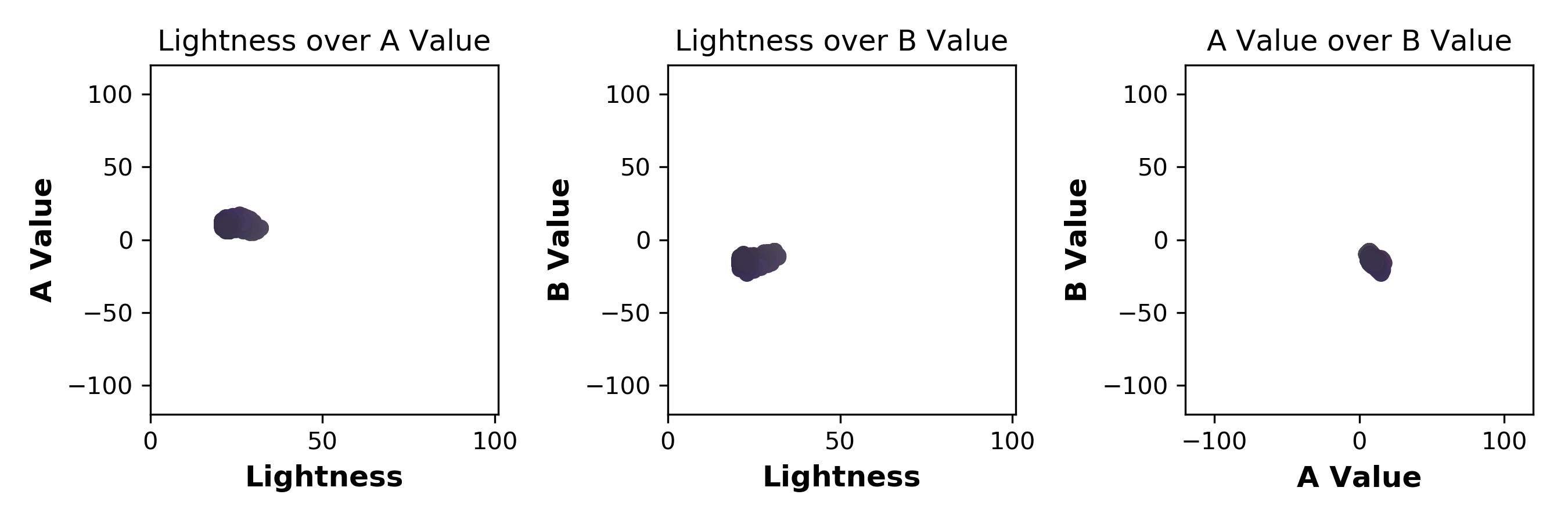

Supplement: Supplementary file 2 — Supporting Information [file ANIE-64-e202413395-s002.zip › Supporting Info - Machine readable data part 1/Figure 4 - glare analysis/24_below_SIanal__1/lab.png]

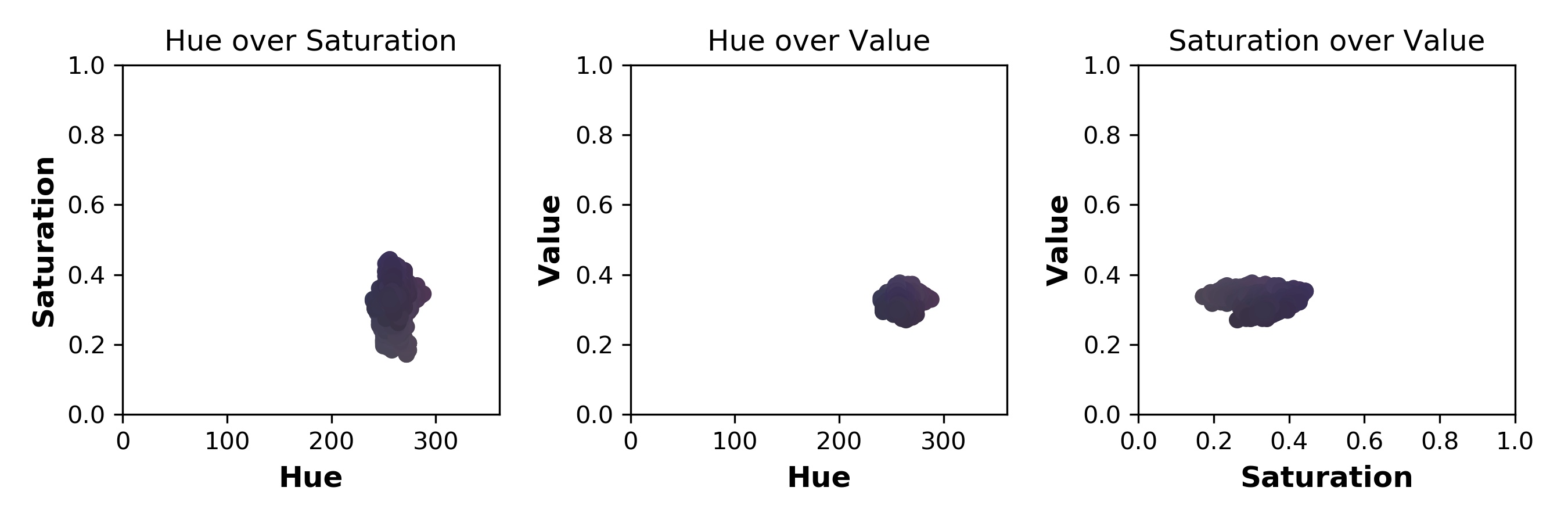

Supplement: Supplementary file 2 — Supporting Information [file ANIE-64-e202413395-s002.zip › Supporting Info - Machine readable data part 1/Figure 4 - glare analysis/24_below_SIanal__1/hsv.png]

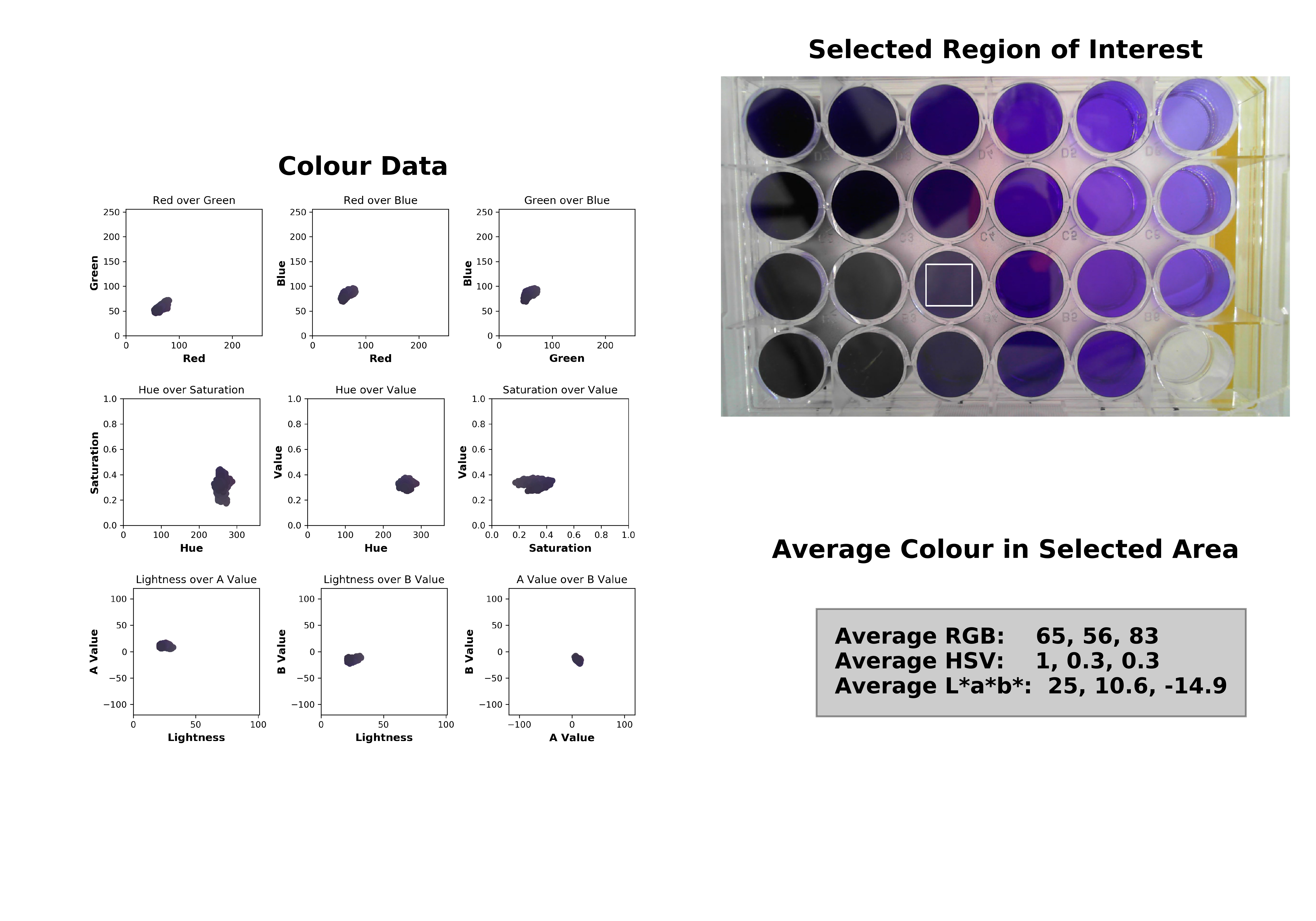

Supplement: Supplementary file 2 — Supporting Information [file ANIE-64-e202413395-s002.zip › Supporting Info - Machine readable data part 1/Figure 4 - glare analysis/24_below_SIanal__1/TILE_WITH_ROI.PNG]

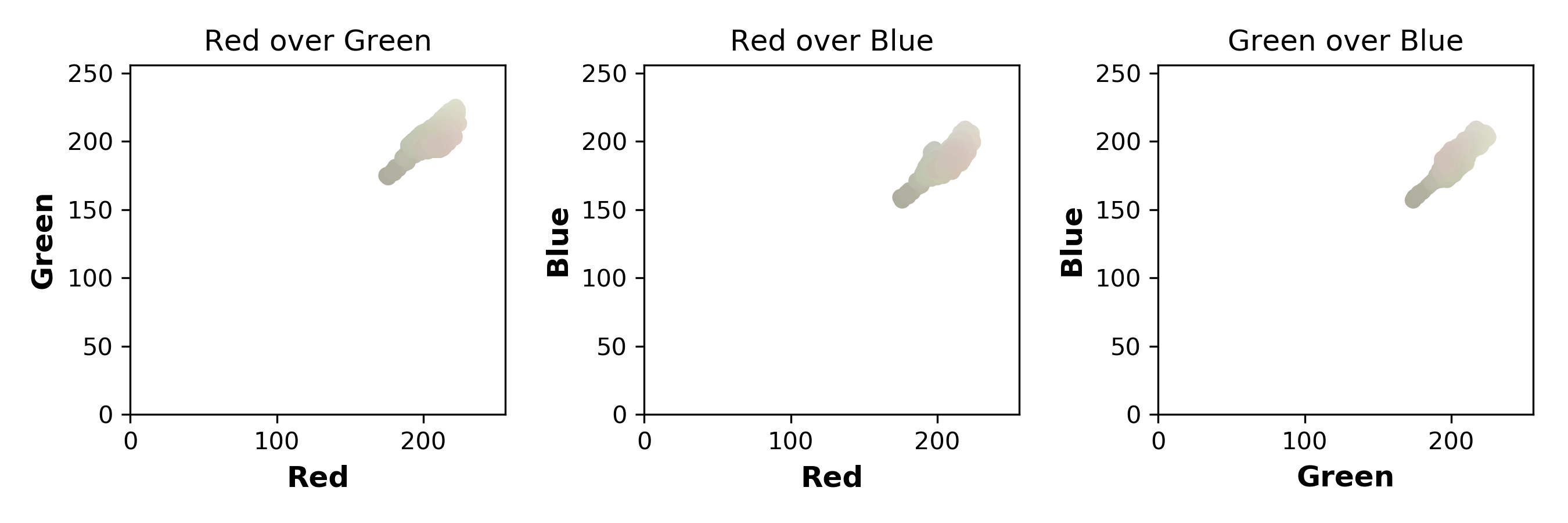

Supplement: Supplementary file 2 — Supporting Information [file ANIE-64-e202413395-s002.zip › Supporting Info - Machine readable data part 1/Figure 4 - glare analysis/6_below_SIanal__1/rgb.png]

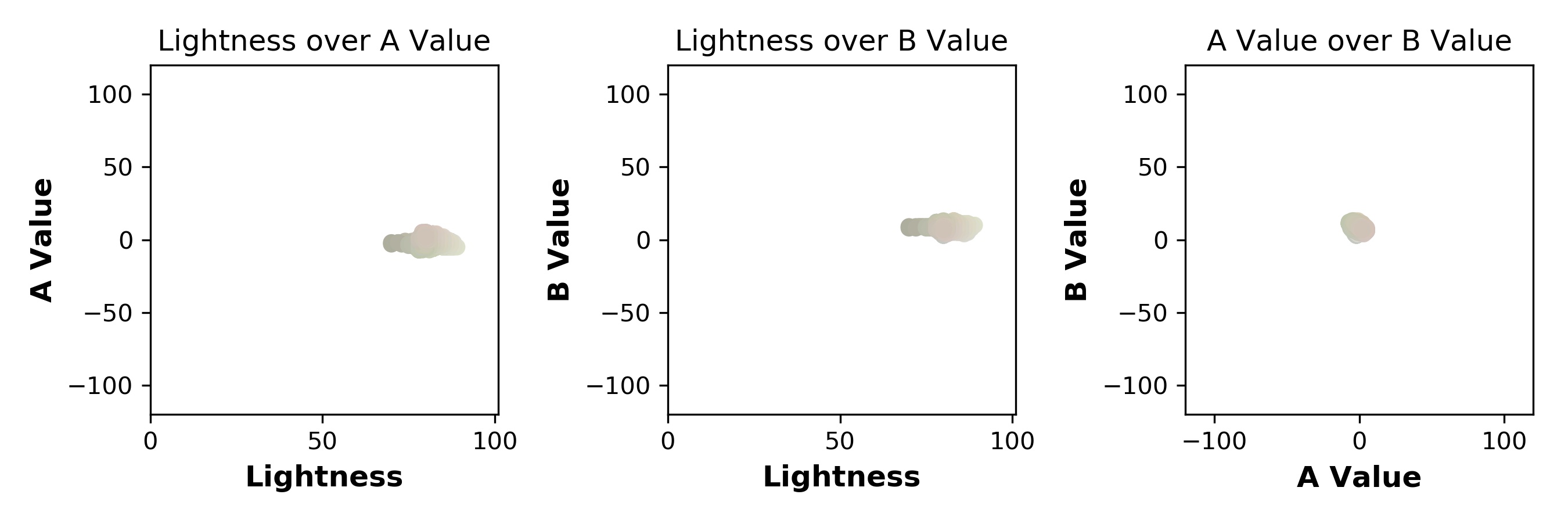

Supplement: Supplementary file 2 — Supporting Information [file ANIE-64-e202413395-s002.zip › Supporting Info - Machine readable data part 1/Figure 4 - glare analysis/6_below_SIanal__1/lab.png]

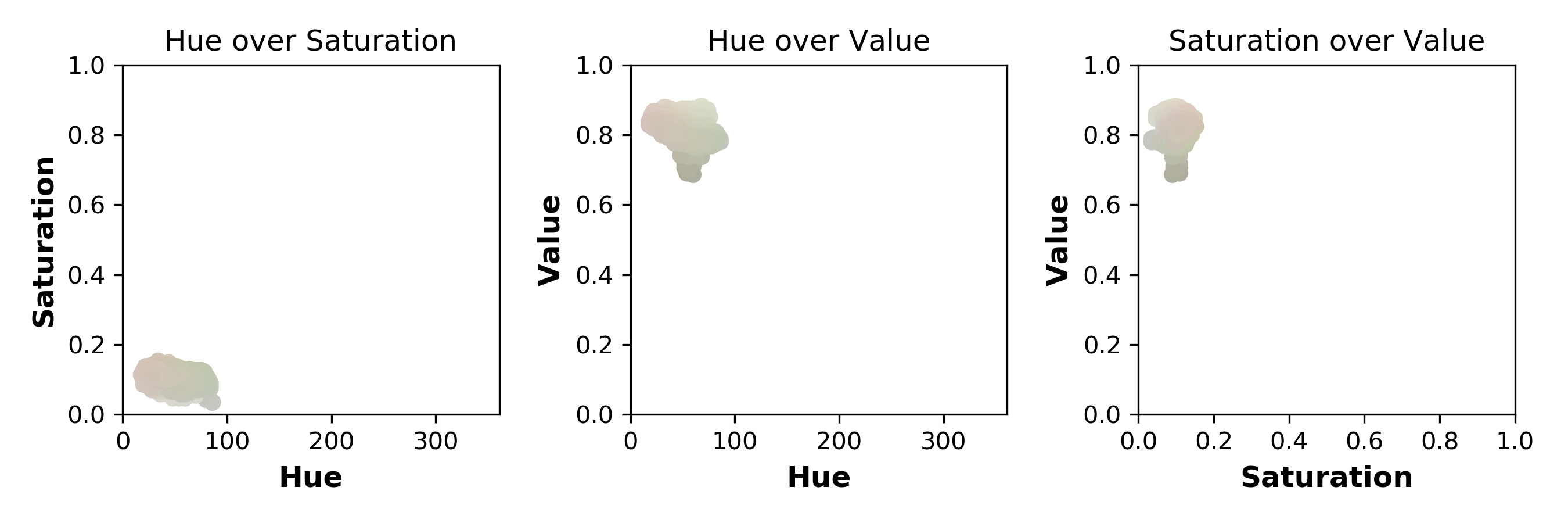

Supplement: Supplementary file 2 — Supporting Information [file ANIE-64-e202413395-s002.zip › Supporting Info - Machine readable data part 1/Figure 4 - glare analysis/6_below_SIanal__1/hsv.png]

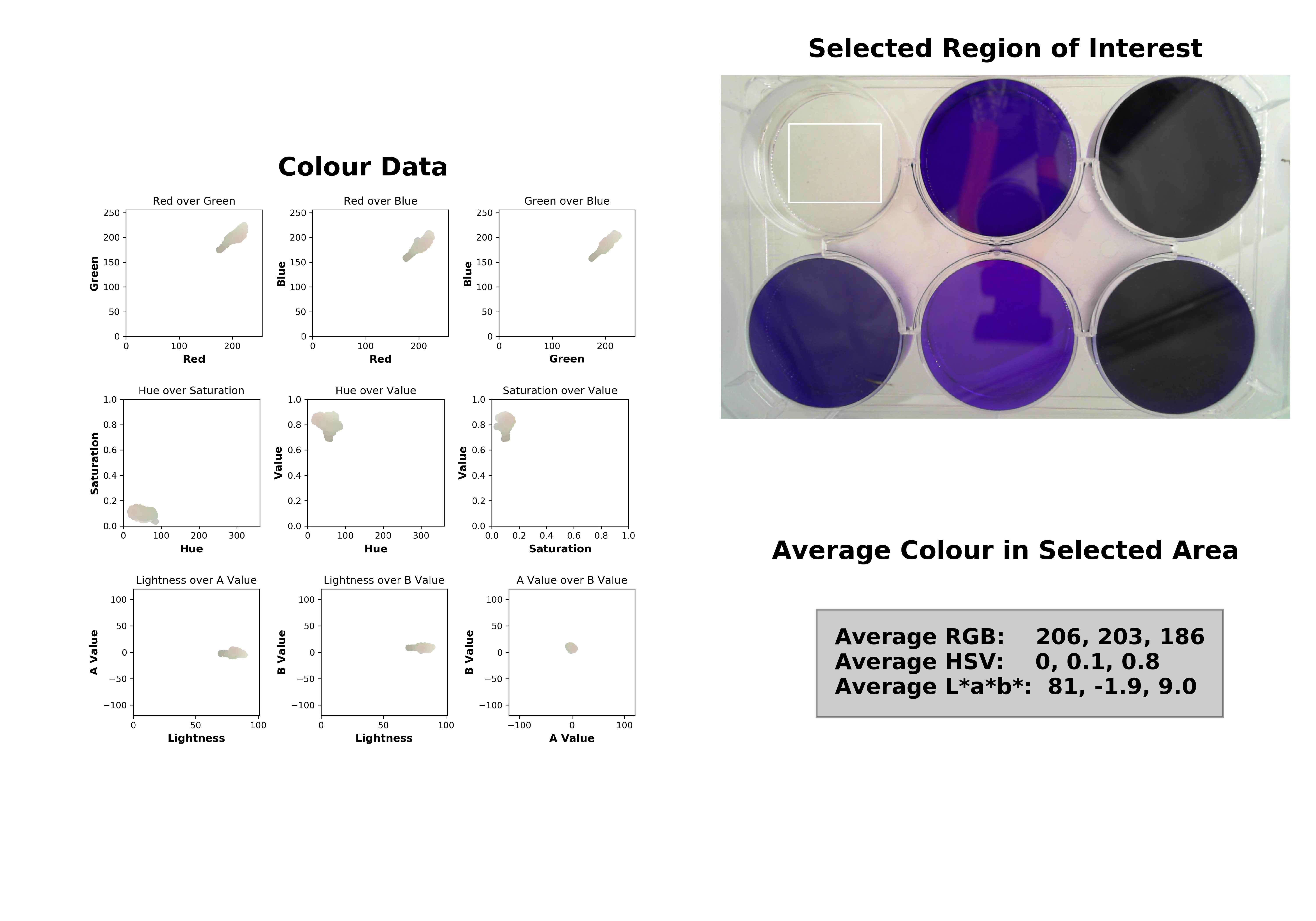

Supplement: Supplementary file 2 — Supporting Information [file ANIE-64-e202413395-s002.zip › Supporting Info - Machine readable data part 1/Figure 4 - glare analysis/6_below_SIanal__1/TILE_WITH_ROI.PNG]

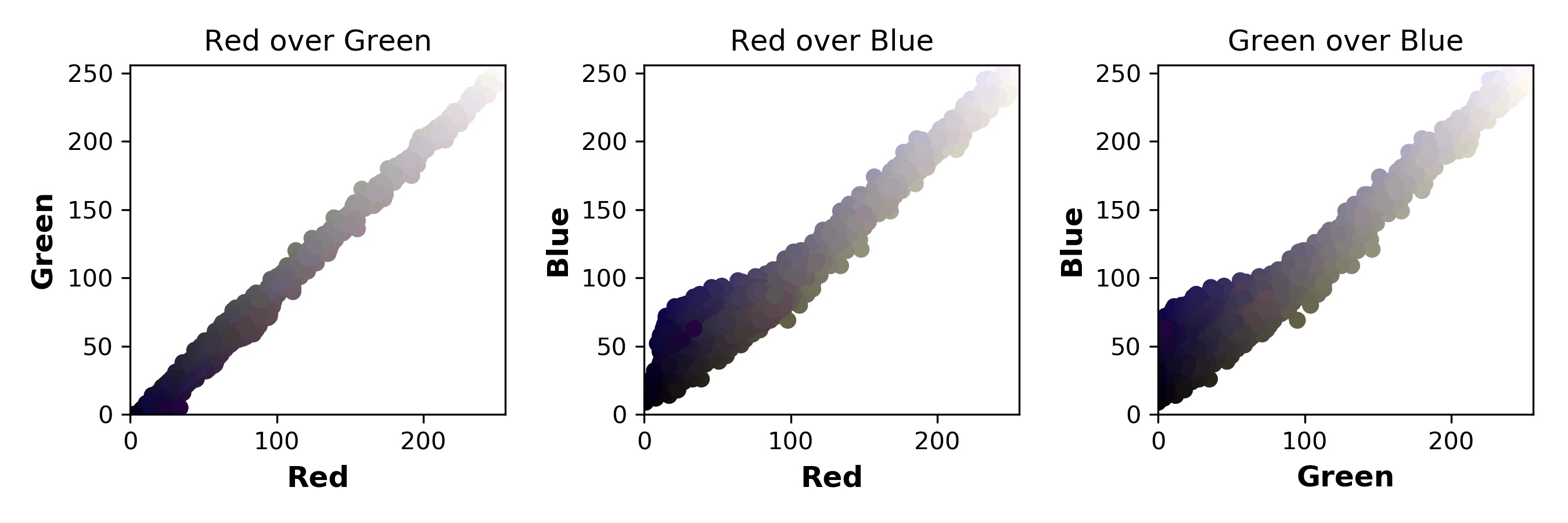

Supplement: Supplementary file 2 — Supporting Information [file ANIE-64-e202413395-s002.zip › Supporting Info - Machine readable data part 1/Figure 4 - glare analysis/6_above_SIanal__1/rgb.png]

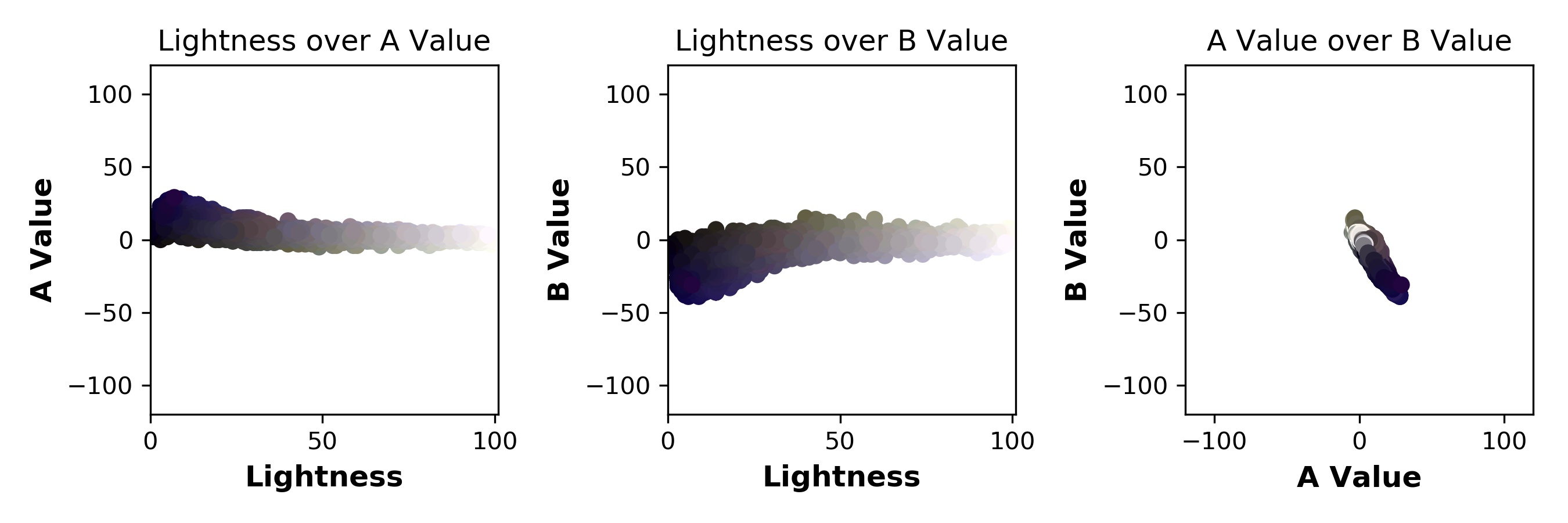

Supplement: Supplementary file 2 — Supporting Information [file ANIE-64-e202413395-s002.zip › Supporting Info - Machine readable data part 1/Figure 4 - glare analysis/6_above_SIanal__1/lab.png]

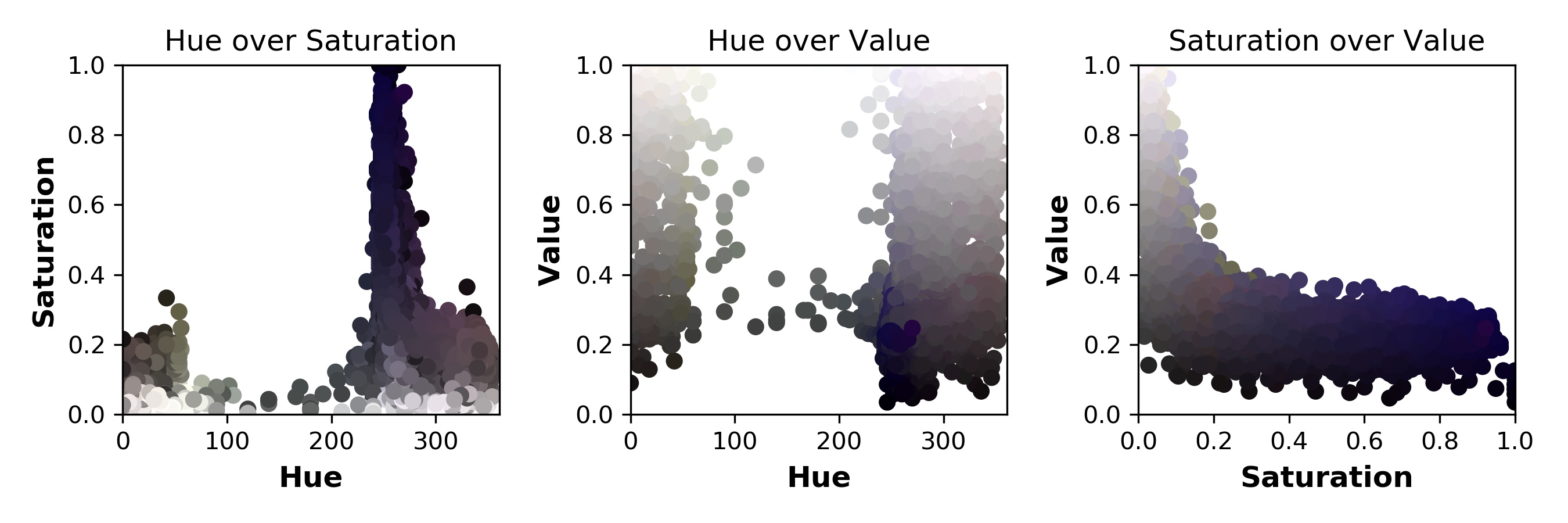

Supplement: Supplementary file 2 — Supporting Information [file ANIE-64-e202413395-s002.zip › Supporting Info - Machine readable data part 1/Figure 4 - glare analysis/6_above_SIanal__1/hsv.png]

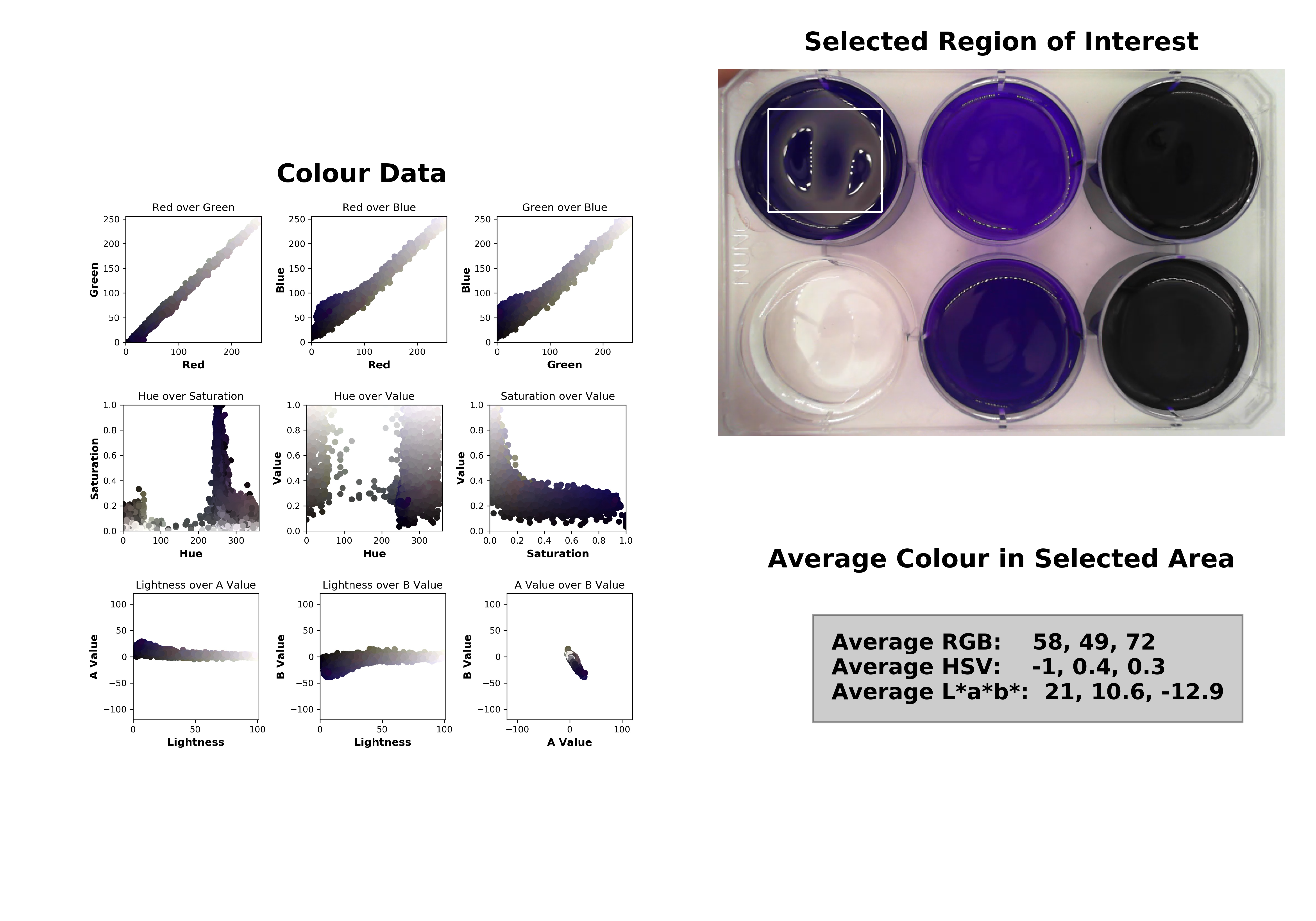

Supplement: Supplementary file 2 — Supporting Information [file ANIE-64-e202413395-s002.zip › Supporting Info - Machine readable data part 1/Figure 4 - glare analysis/6_above_SIanal__1/TILE_WITH_ROI.PNG]

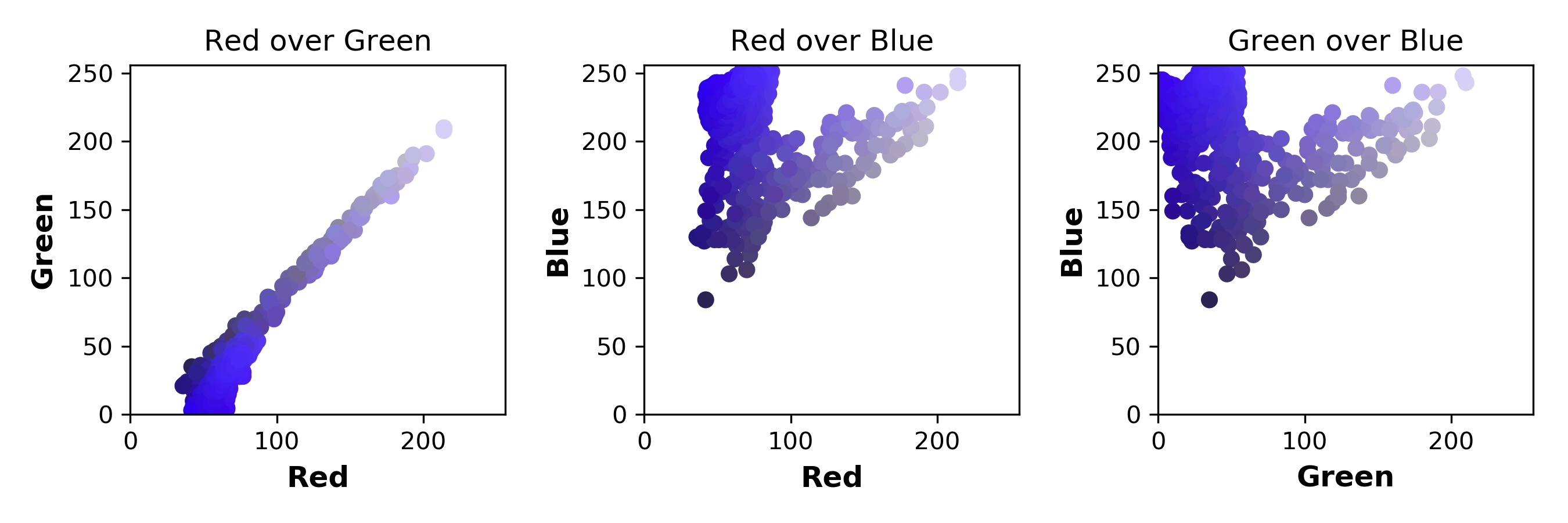

Supplement: Supplementary file 2 — Supporting Information [file ANIE-64-e202413395-s002.zip › Supporting Info - Machine readable data part 1/Figure 4 - glare analysis/24_above_SIanal__6/rgb.png]

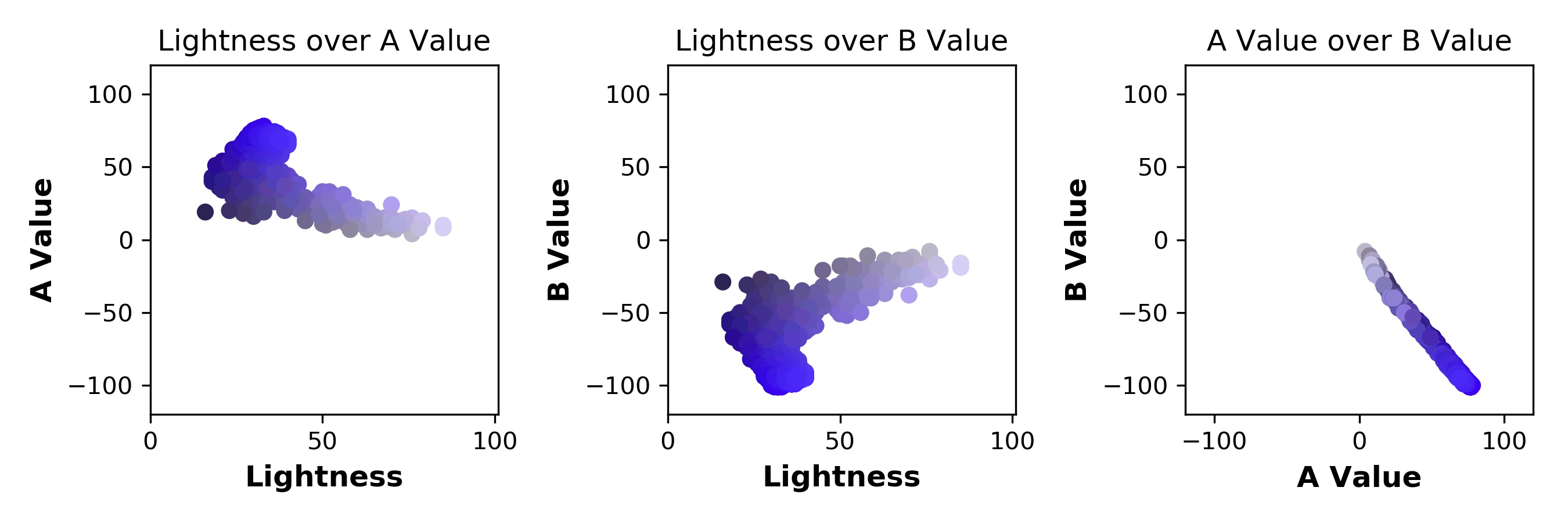

Supplement: Supplementary file 2 — Supporting Information [file ANIE-64-e202413395-s002.zip › Supporting Info - Machine readable data part 1/Figure 4 - glare analysis/24_above_SIanal__6/lab.png]

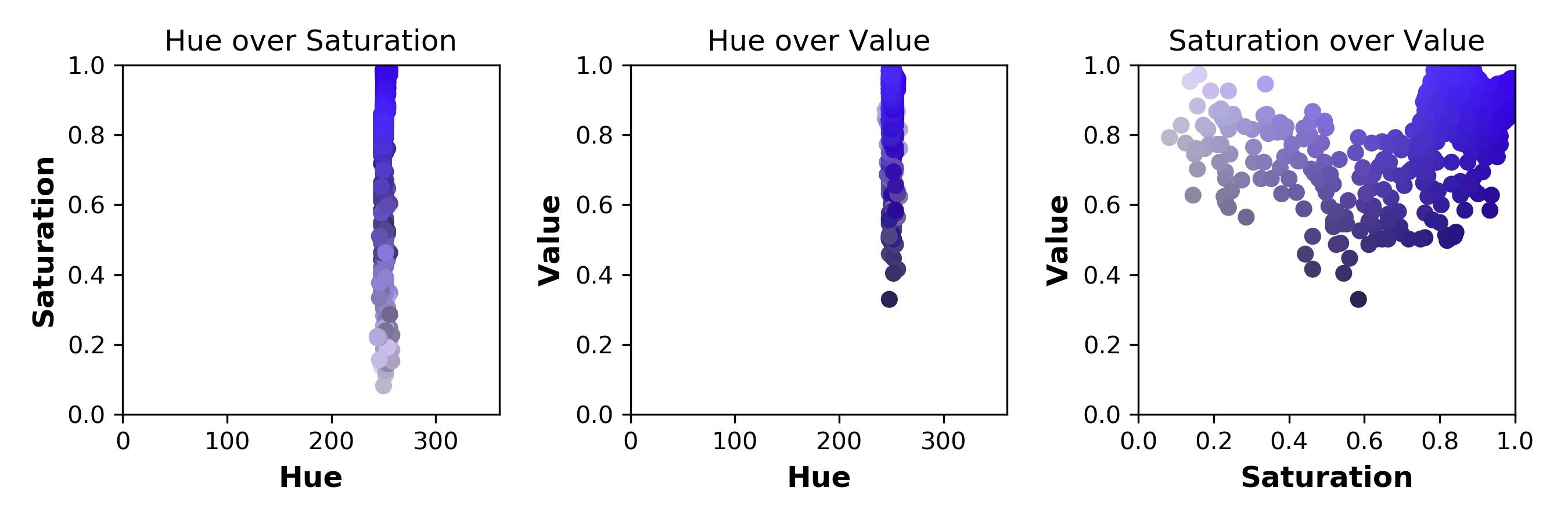

Supplement: Supplementary file 2 — Supporting Information [file ANIE-64-e202413395-s002.zip › Supporting Info - Machine readable data part 1/Figure 4 - glare analysis/24_above_SIanal__6/hsv.png]

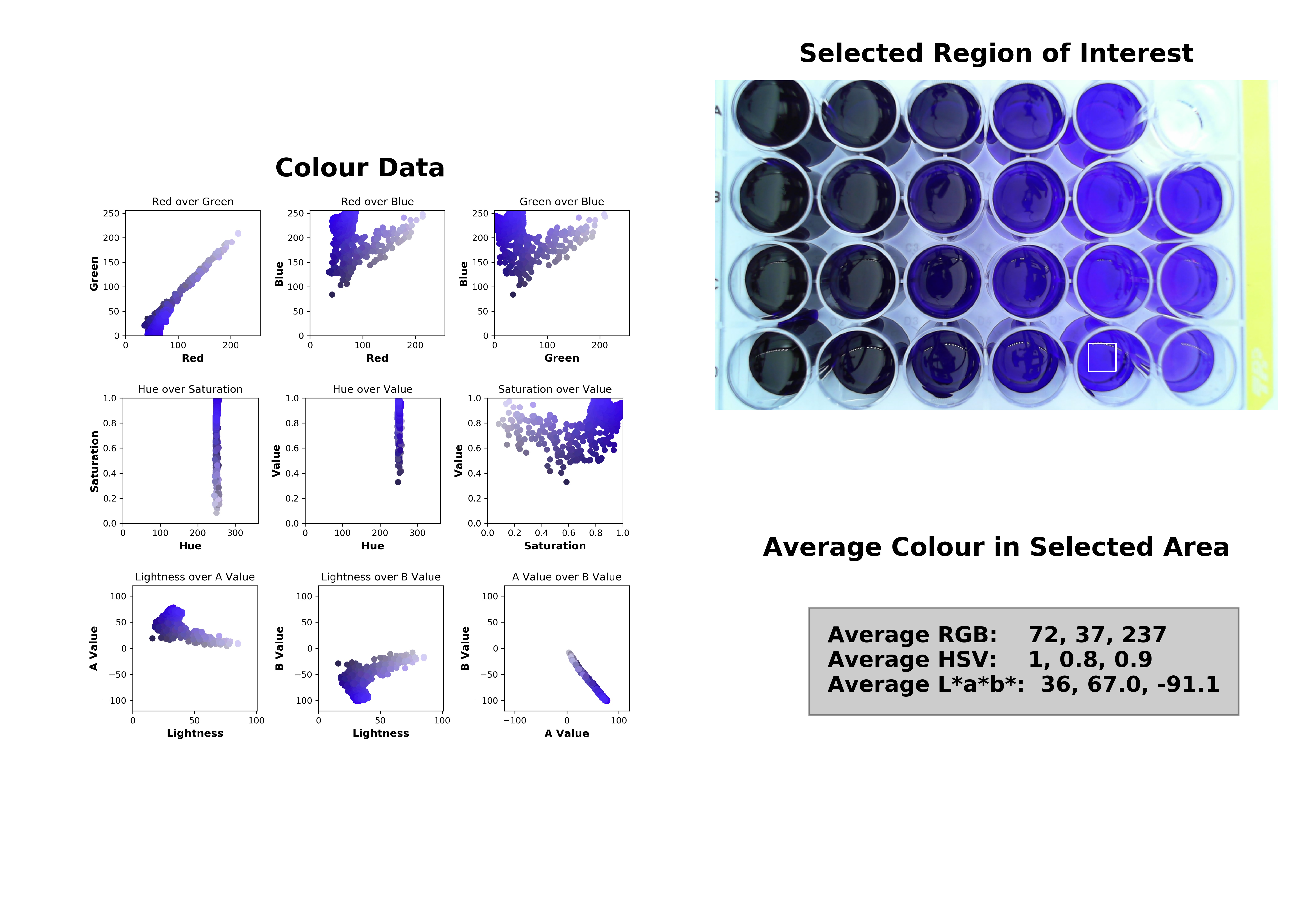

Supplement: Supplementary file 2 — Supporting Information [file ANIE-64-e202413395-s002.zip › Supporting Info - Machine readable data part 1/Figure 4 - glare analysis/24_above_SIanal__6/TILE_WITH_ROI.PNG]

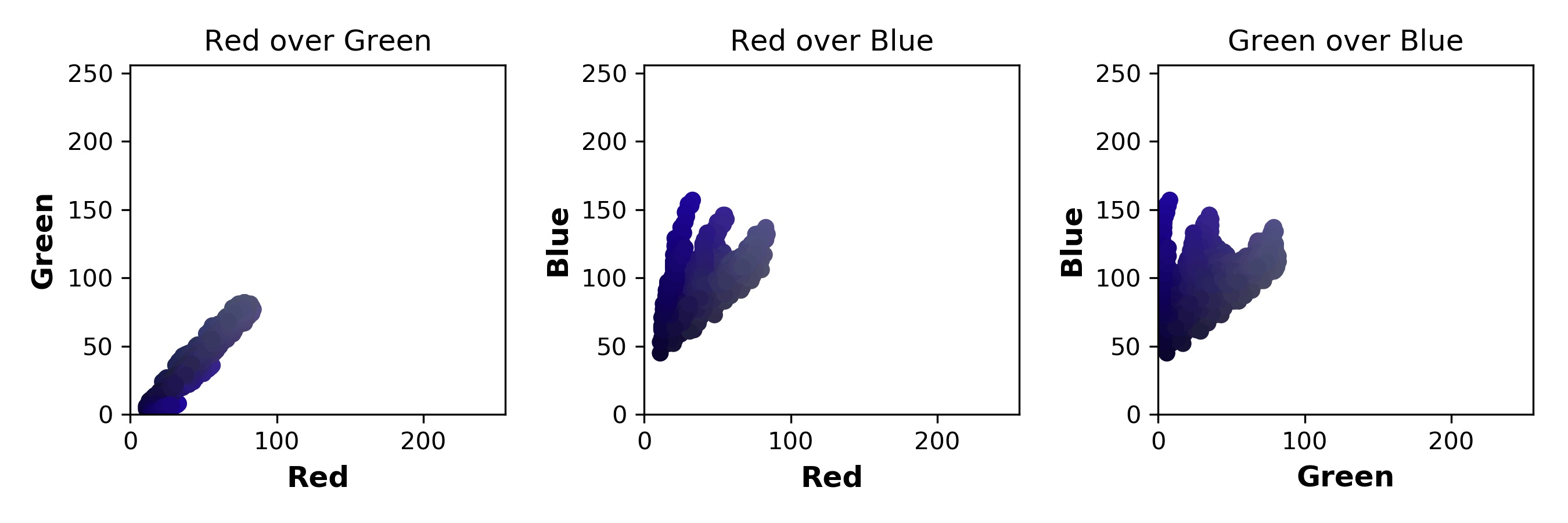

Supplement: Supplementary file 2 — Supporting Information [file ANIE-64-e202413395-s002.zip › Supporting Info - Machine readable data part 1/Figure 4 - glare analysis/24_above_SIanal__1/rgb.png]

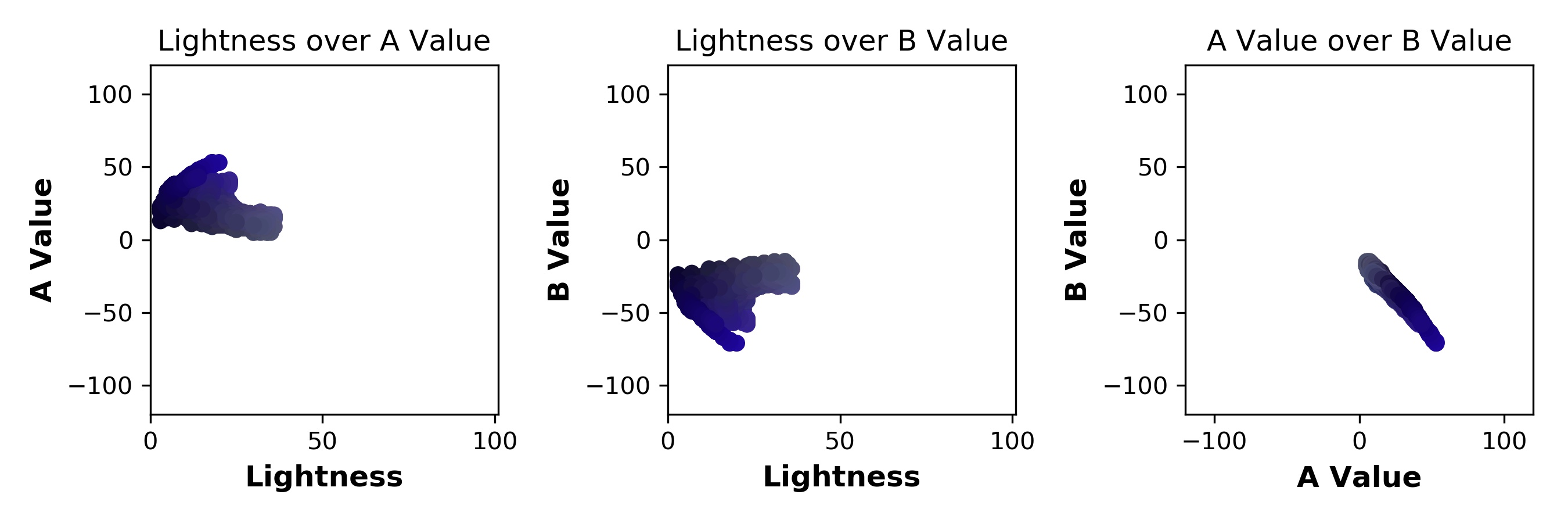

Supplement: Supplementary file 2 — Supporting Information [file ANIE-64-e202413395-s002.zip › Supporting Info - Machine readable data part 1/Figure 4 - glare analysis/24_above_SIanal__1/lab.png]

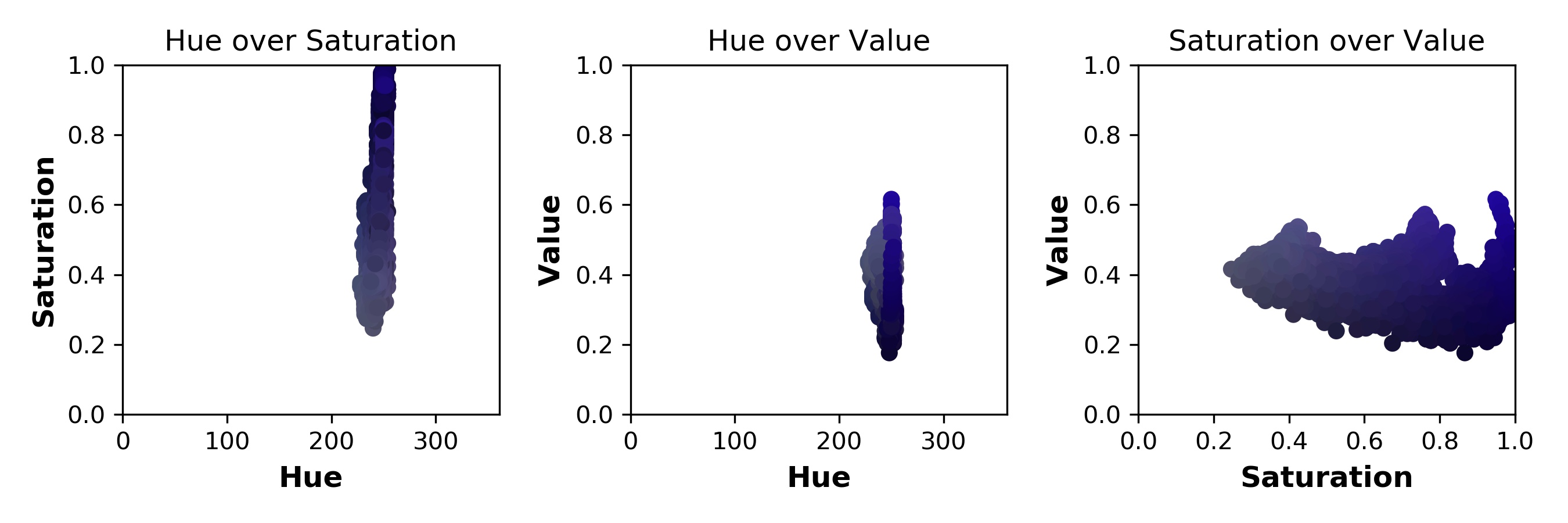

Supplement: Supplementary file 2 — Supporting Information [file ANIE-64-e202413395-s002.zip › Supporting Info - Machine readable data part 1/Figure 4 - glare analysis/24_above_SIanal__1/hsv.png]

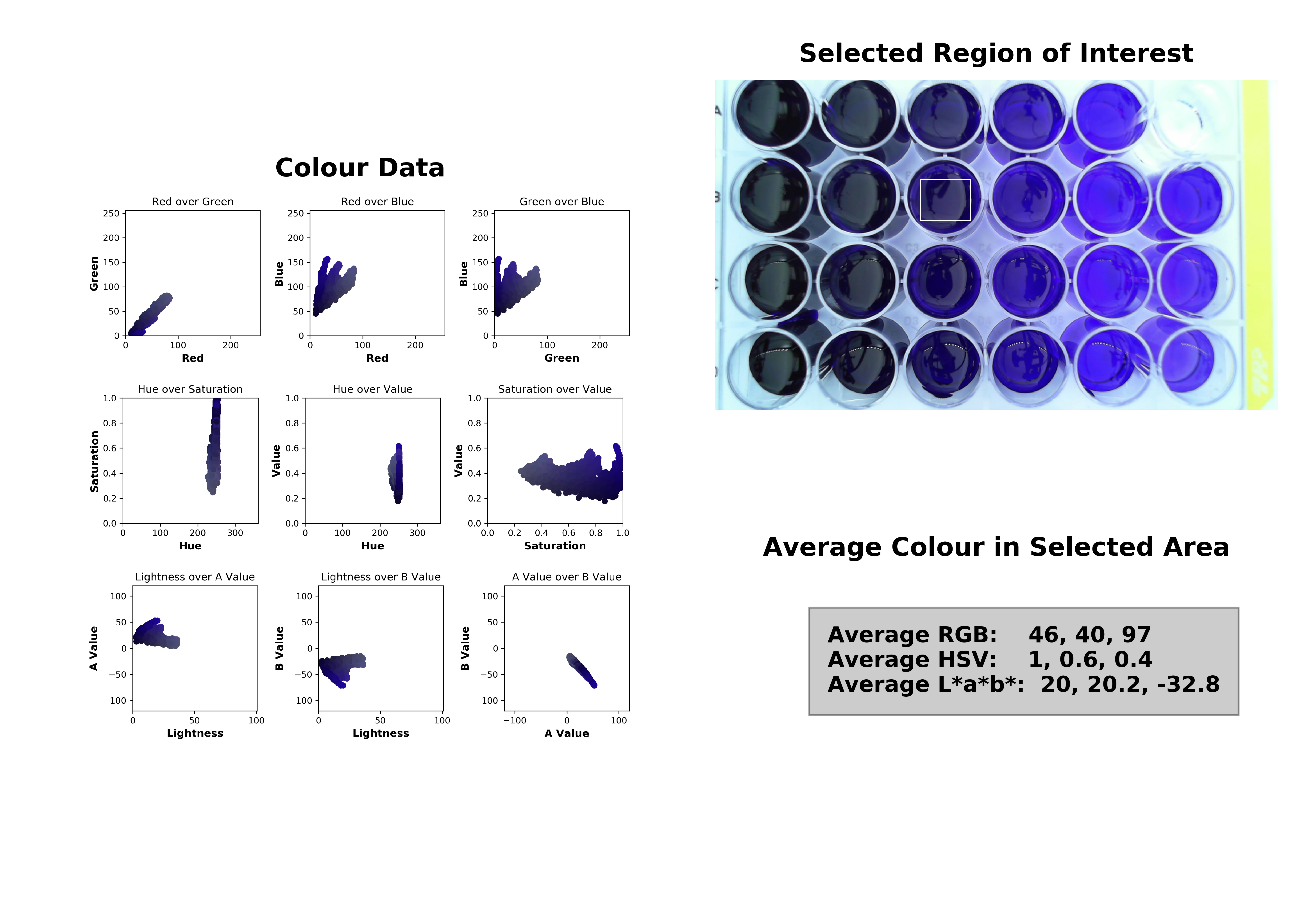

Supplement: Supplementary file 2 — Supporting Information [file ANIE-64-e202413395-s002.zip › Supporting Info - Machine readable data part 1/Figure 4 - glare analysis/24_above_SIanal__1/TILE_WITH_ROI.PNG]

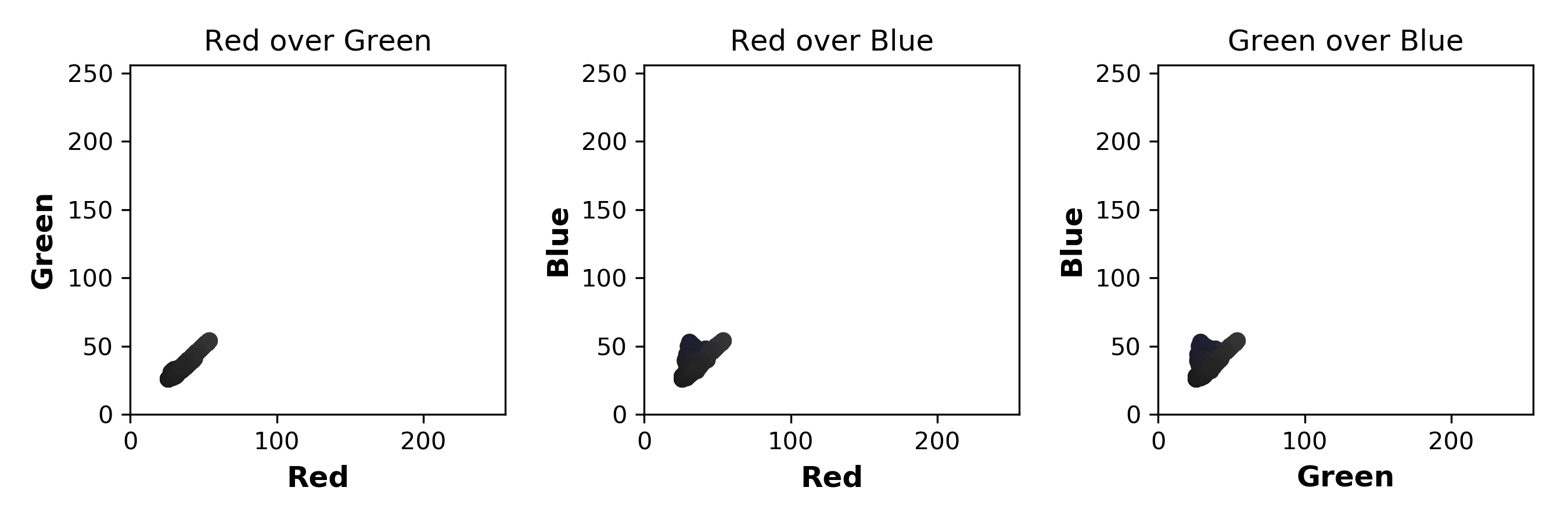

Supplement: Supplementary file 2 — Supporting Information [file ANIE-64-e202413395-s002.zip › Supporting Info - Machine readable data part 1/Figure 4 - glare analysis/24_below_SIanal/rgb.png]

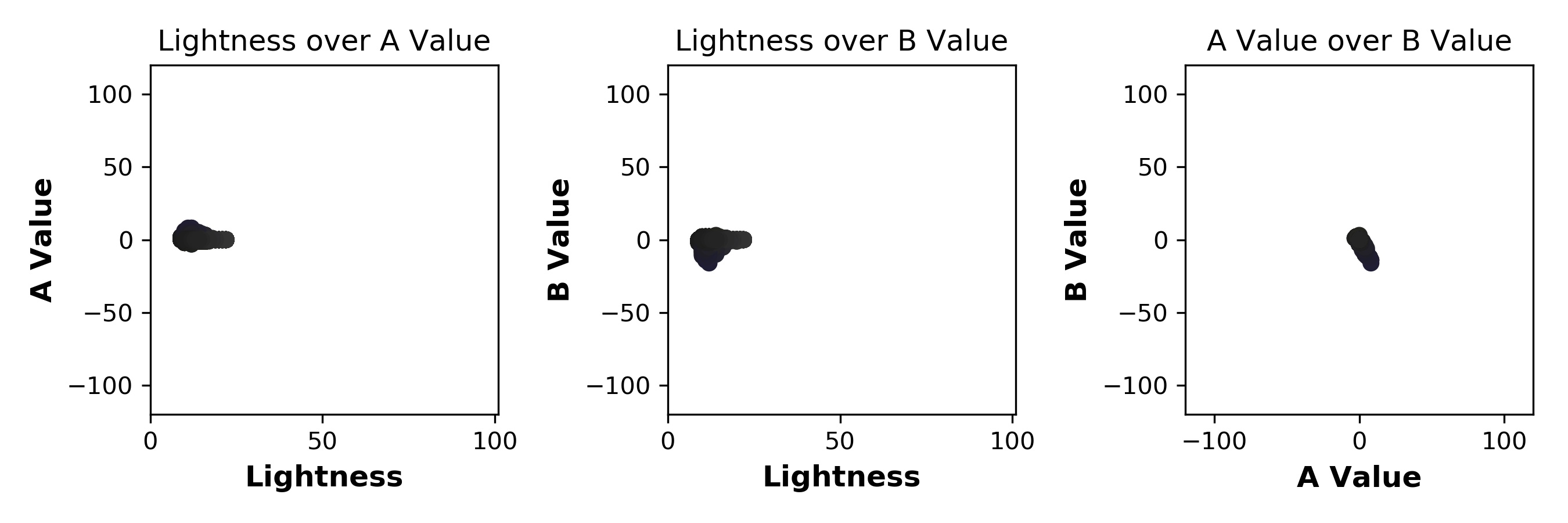

Supplement: Supplementary file 2 — Supporting Information [file ANIE-64-e202413395-s002.zip › Supporting Info - Machine readable data part 1/Figure 4 - glare analysis/24_below_SIanal/lab.png]

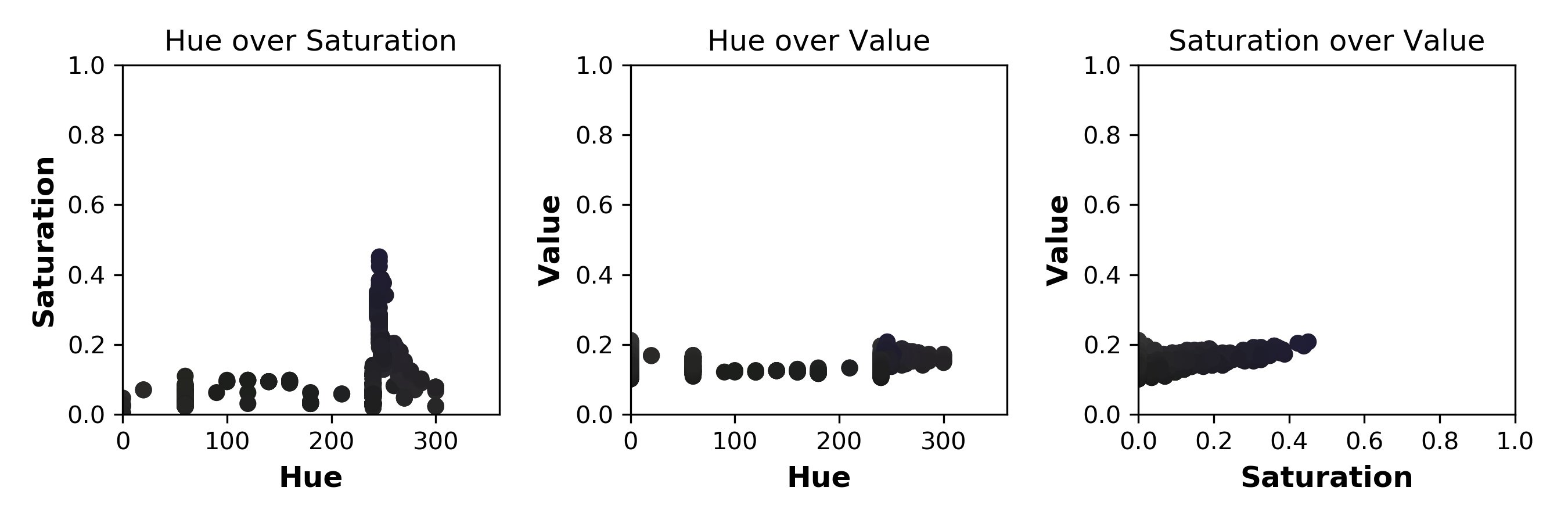

Supplement: Supplementary file 2 — Supporting Information [file ANIE-64-e202413395-s002.zip › Supporting Info - Machine readable data part 1/Figure 4 - glare analysis/24_below_SIanal/hsv.png]

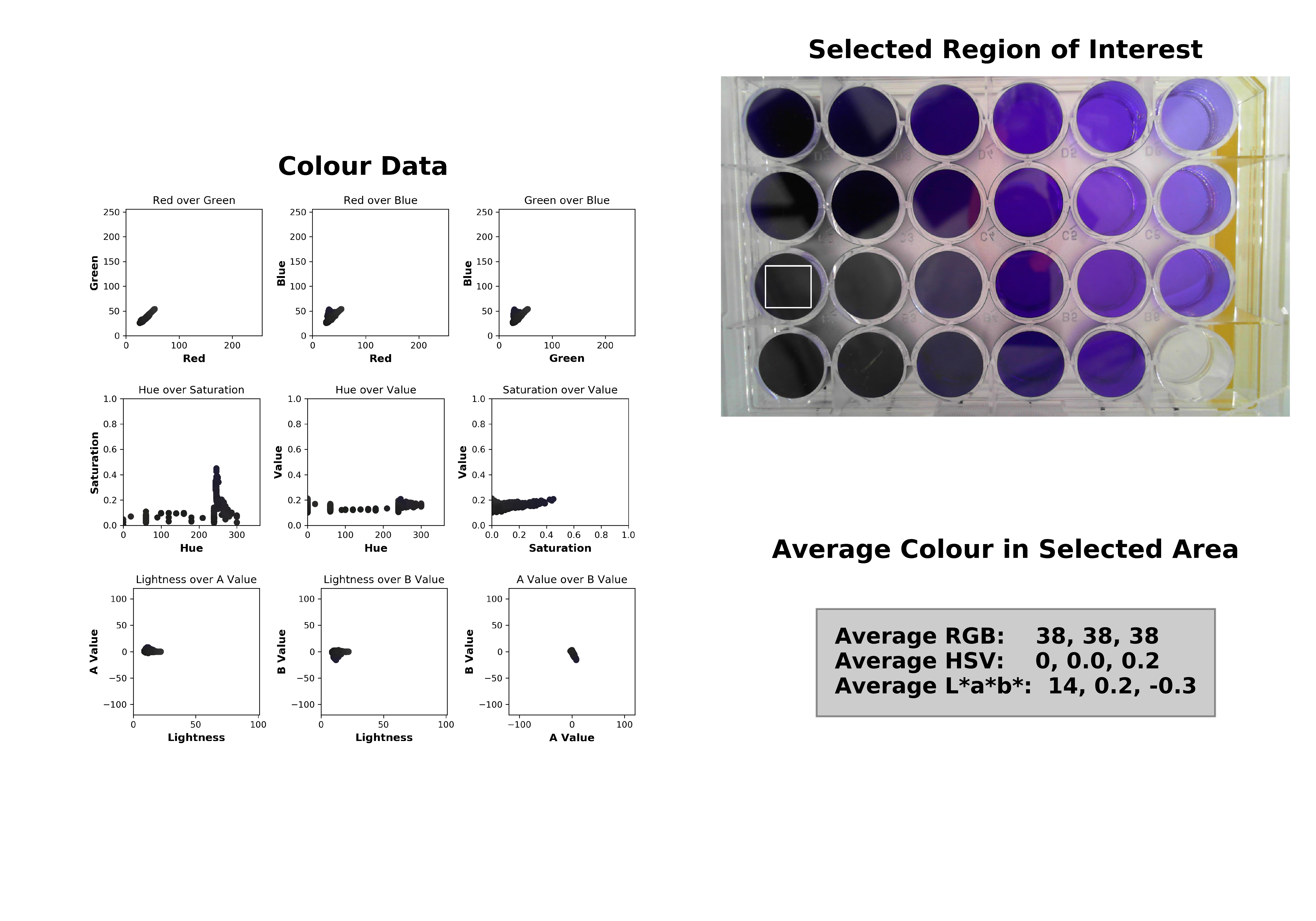

Supplement: Supplementary file 2 — Supporting Information [file ANIE-64-e202413395-s002.zip › Supporting Info - Machine readable data part 1/Figure 4 - glare analysis/24_below_SIanal/TILE_WITH_ROI.PNG]

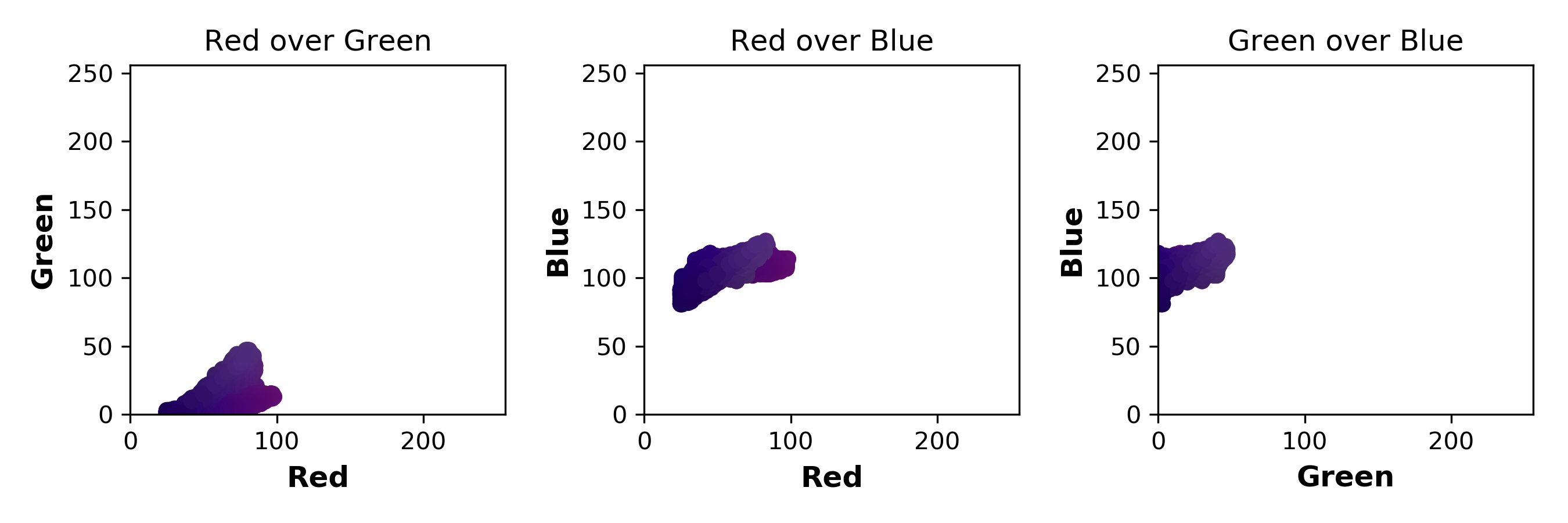

Supplement: Supplementary file 2 — Supporting Information [file ANIE-64-e202413395-s002.zip › Supporting Info - Machine readable data part 1/Figure 4 - glare analysis/24_below_SIanal__2/rgb.png]

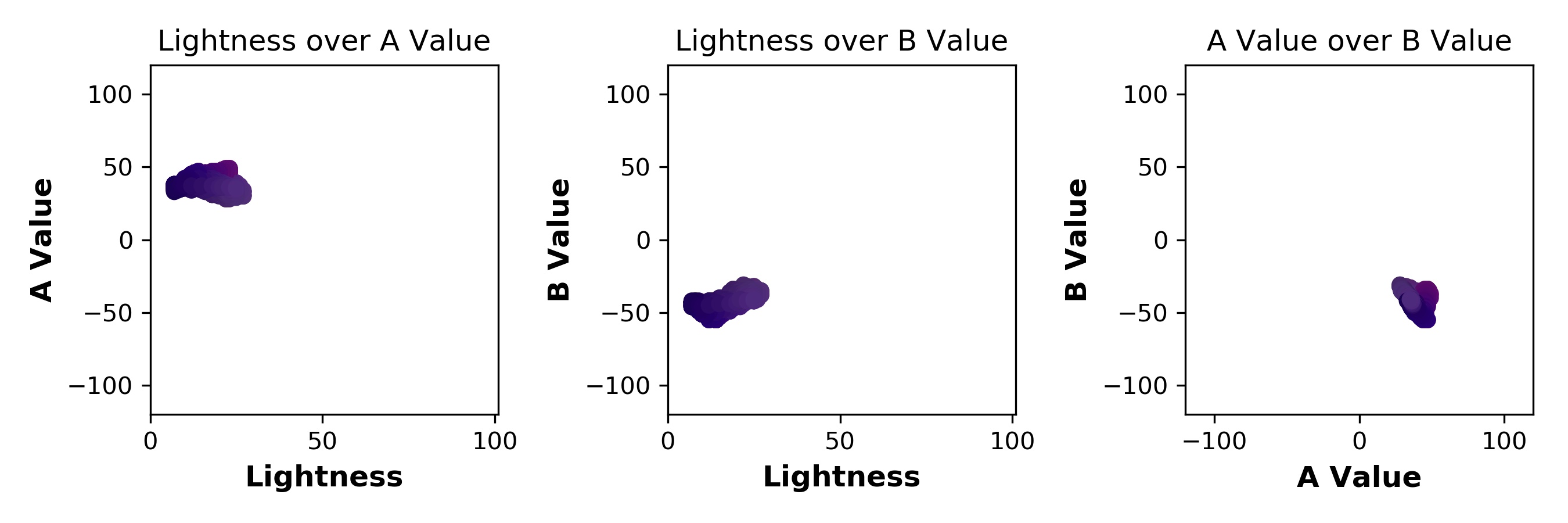

Supplement: Supplementary file 2 — Supporting Information [file ANIE-64-e202413395-s002.zip › Supporting Info - Machine readable data part 1/Figure 4 - glare analysis/24_below_SIanal__2/lab.png]

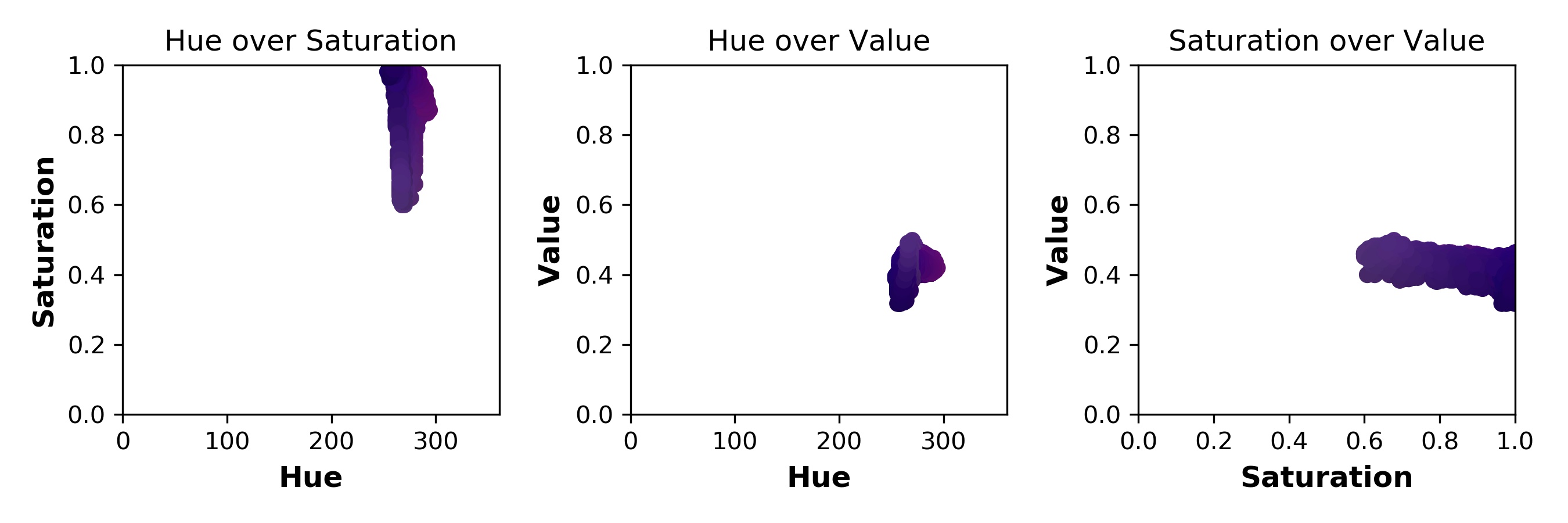

Supplement: Supplementary file 2 — Supporting Information [file ANIE-64-e202413395-s002.zip › Supporting Info - Machine readable data part 1/Figure 4 - glare analysis/24_below_SIanal__2/hsv.png]

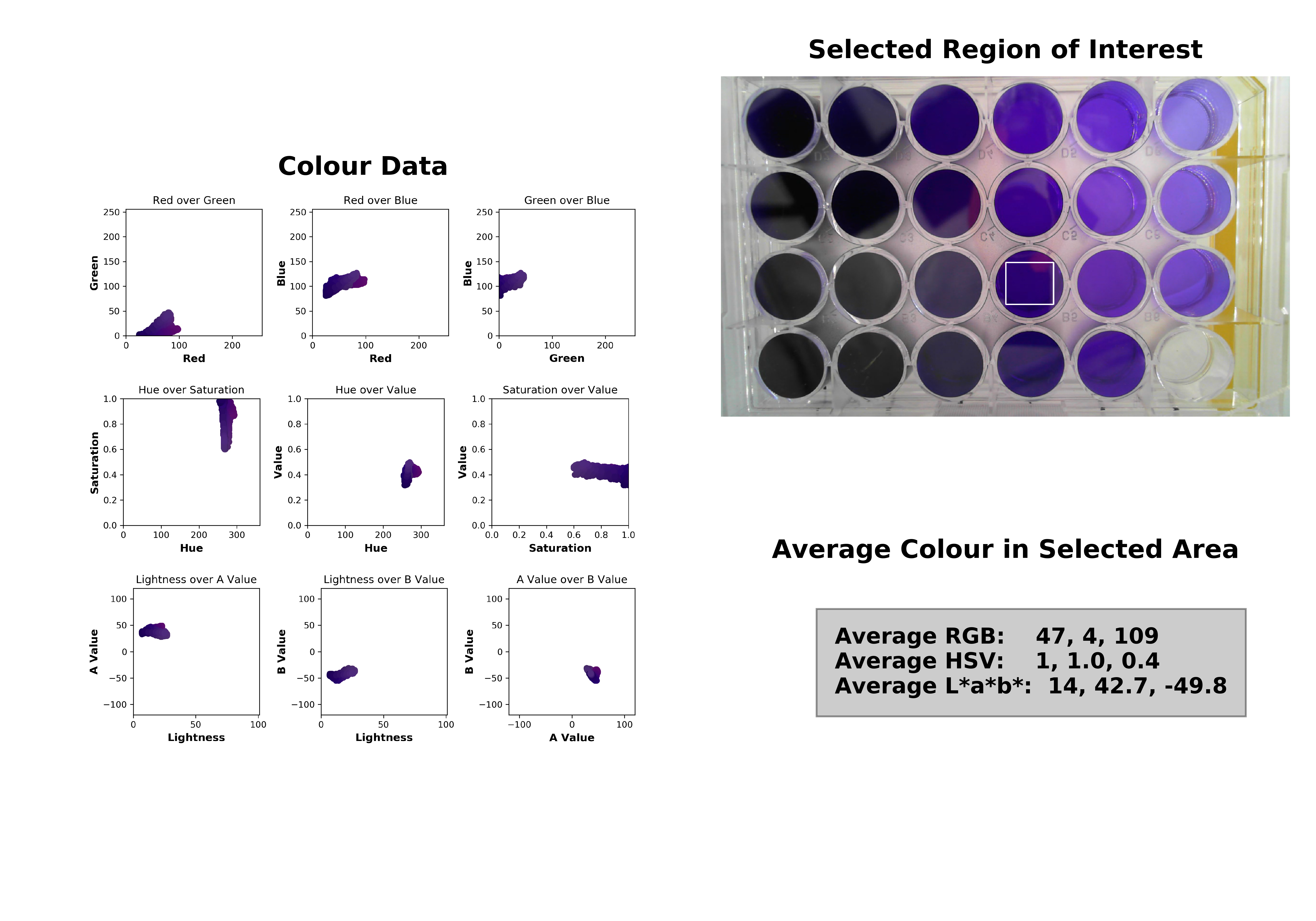

Supplement: Supplementary file 2 — Supporting Information [file ANIE-64-e202413395-s002.zip › Supporting Info - Machine readable data part 1/Figure 4 - glare analysis/24_below_SIanal__2/TILE_WITH_ROI.PNG]

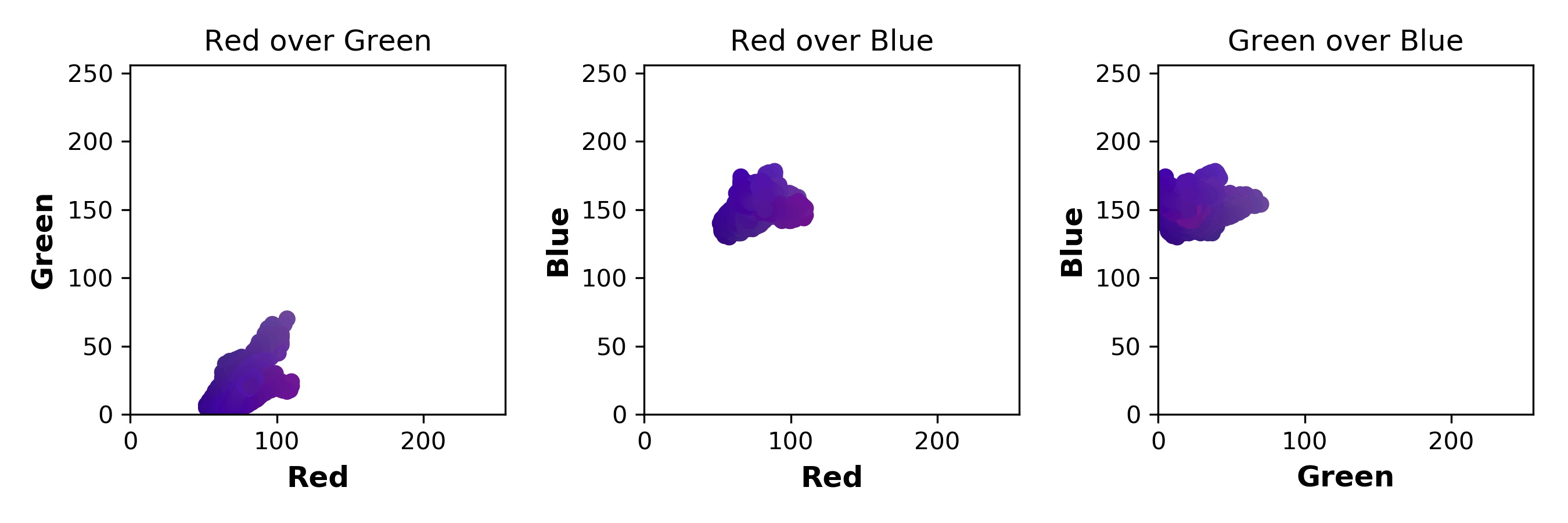

Supplement: Supplementary file 2 — Supporting Information [file ANIE-64-e202413395-s002.zip › Supporting Info - Machine readable data part 1/Figure 4 - glare analysis/24_below_SIanal__5/rgb.png]

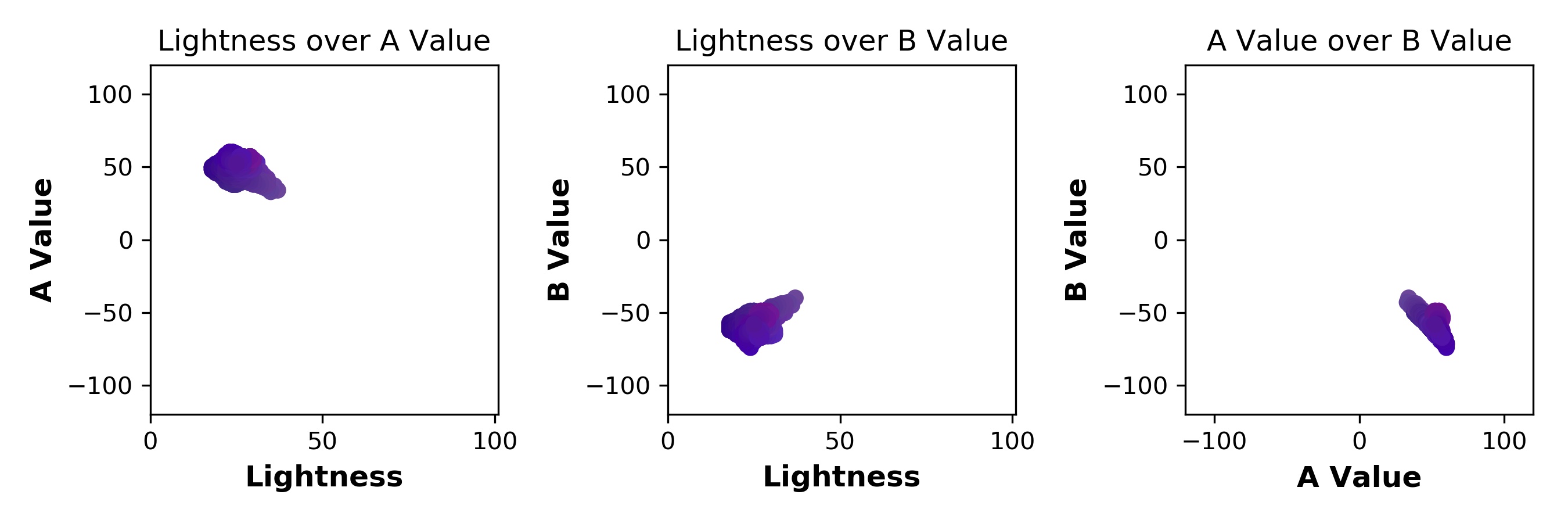

Supplement: Supplementary file 2 — Supporting Information [file ANIE-64-e202413395-s002.zip › Supporting Info - Machine readable data part 1/Figure 4 - glare analysis/24_below_SIanal__5/lab.png]

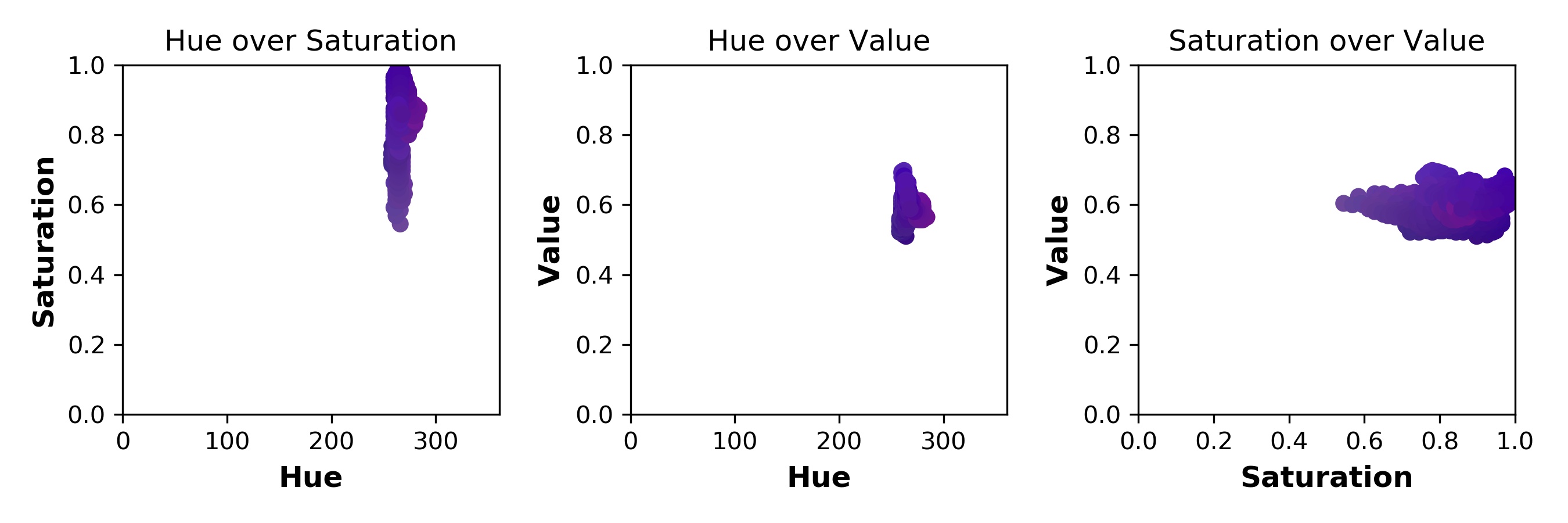

Supplement: Supplementary file 2 — Supporting Information [file ANIE-64-e202413395-s002.zip › Supporting Info - Machine readable data part 1/Figure 4 - glare analysis/24_below_SIanal__5/hsv.png]

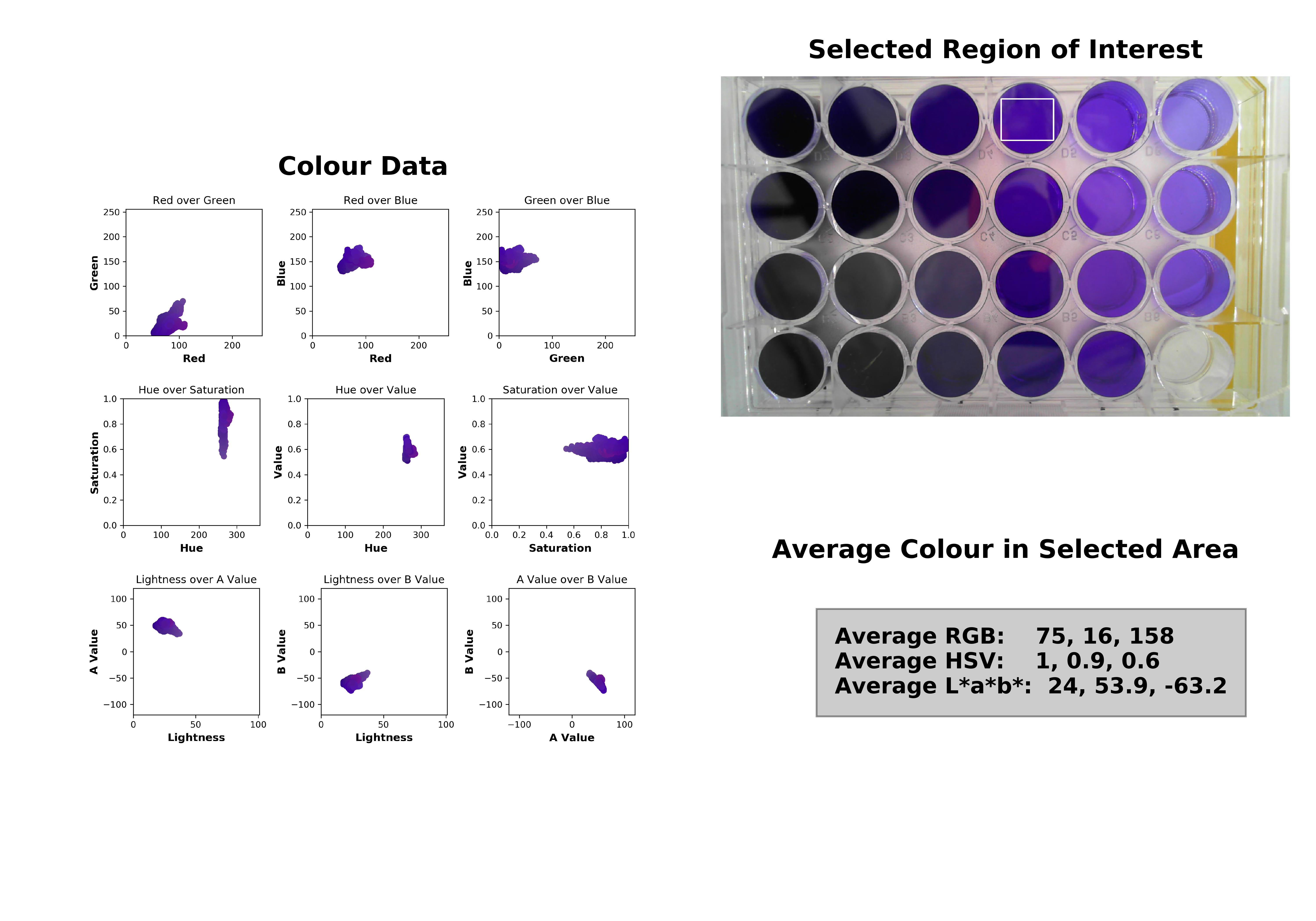

Supplement: Supplementary file 2 — Supporting Information [file ANIE-64-e202413395-s002.zip › Supporting Info - Machine readable data part 1/Figure 4 - glare analysis/24_below_SIanal__5/TILE_WITH_ROI.PNG]

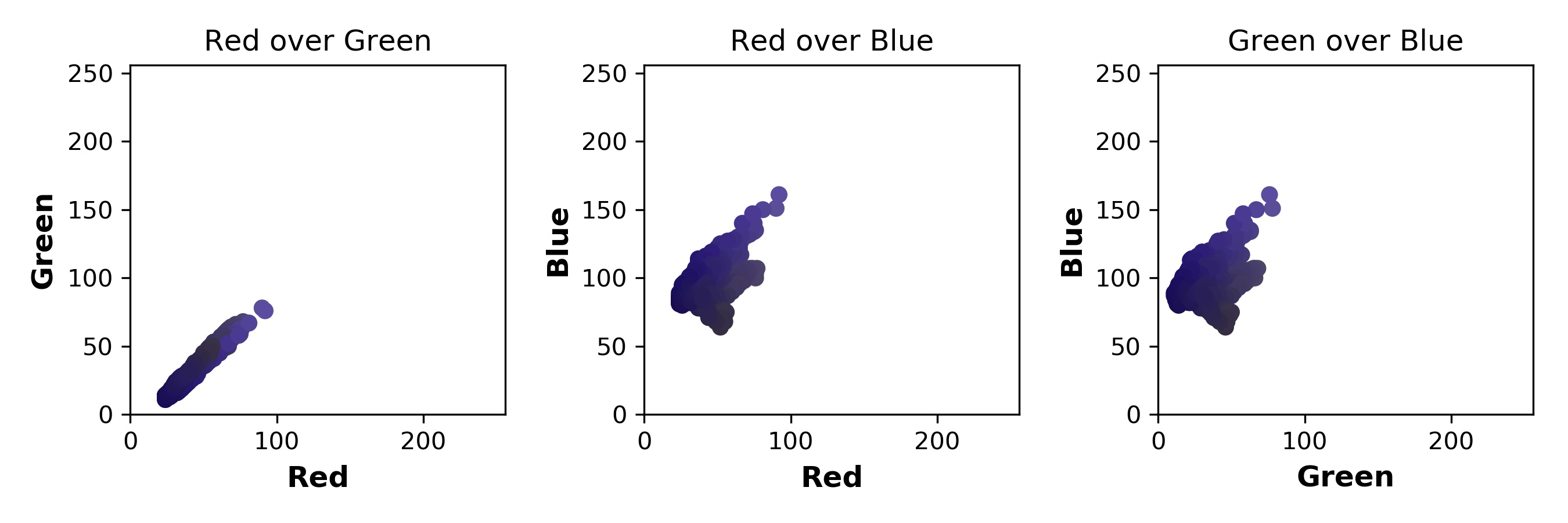

Supplement: Supplementary file 2 — Supporting Information [file ANIE-64-e202413395-s002.zip › Supporting Info - Machine readable data part 1/Figure 4 - glare analysis/6_below_SIanal/rgb.png]

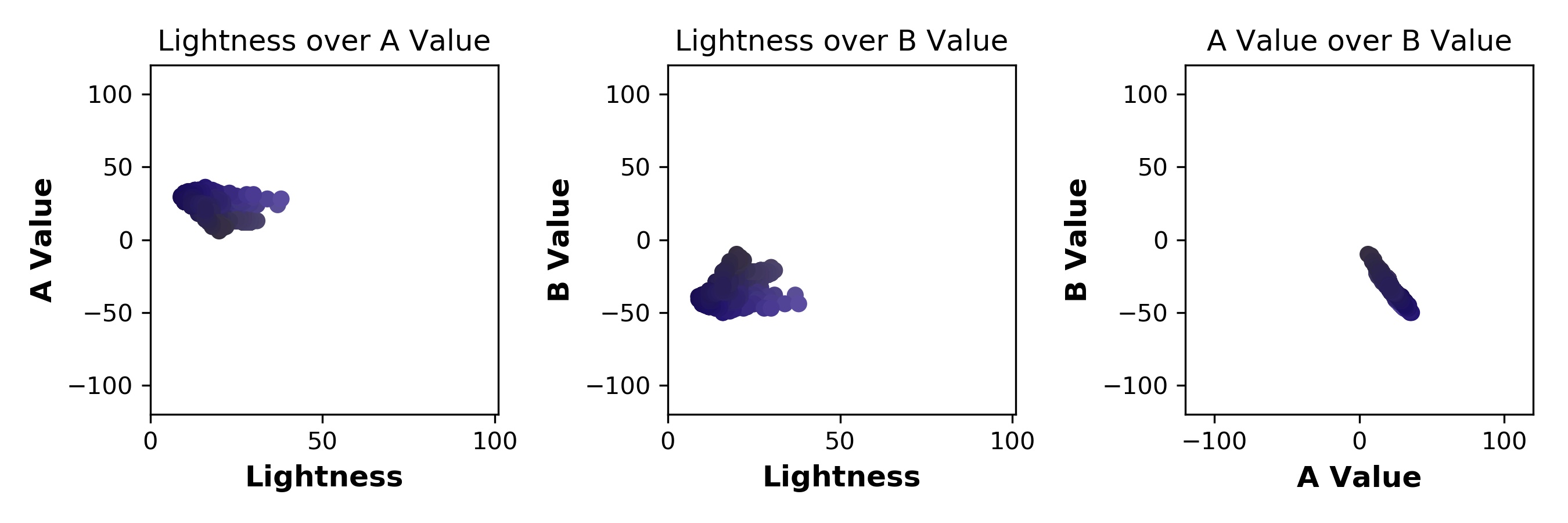

Supplement: Supplementary file 2 — Supporting Information [file ANIE-64-e202413395-s002.zip › Supporting Info - Machine readable data part 1/Figure 4 - glare analysis/6_below_SIanal/lab.png]

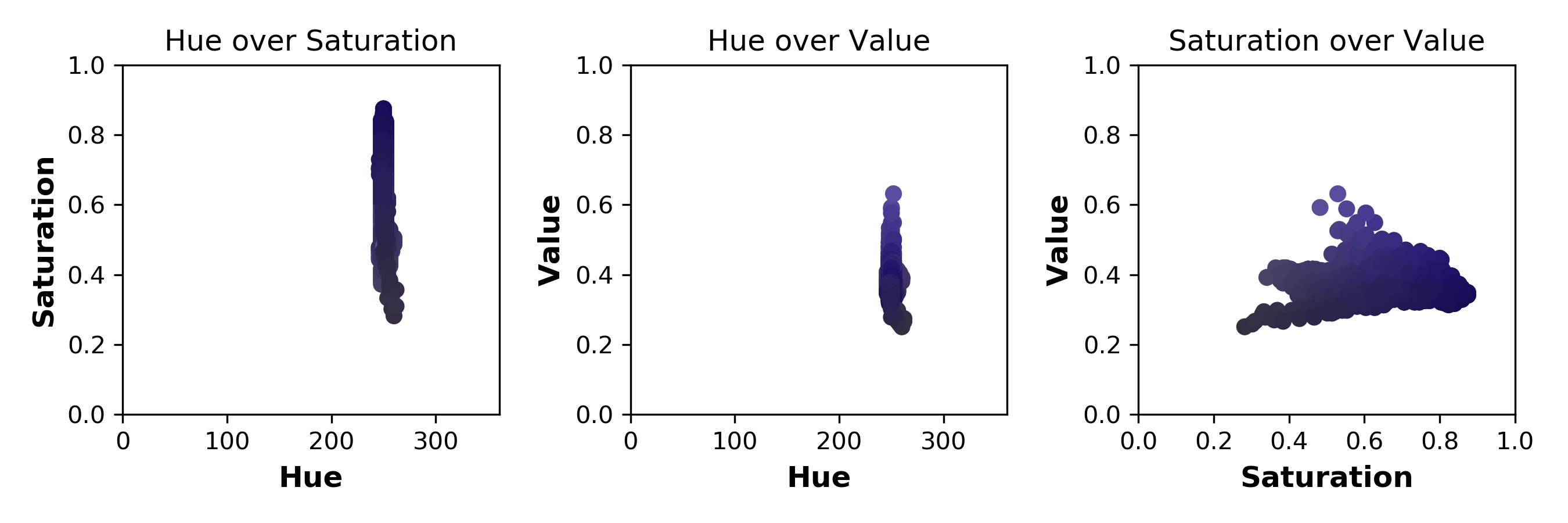

Supplement: Supplementary file 2 — Supporting Information [file ANIE-64-e202413395-s002.zip › Supporting Info - Machine readable data part 1/Figure 4 - glare analysis/6_below_SIanal/hsv.png]

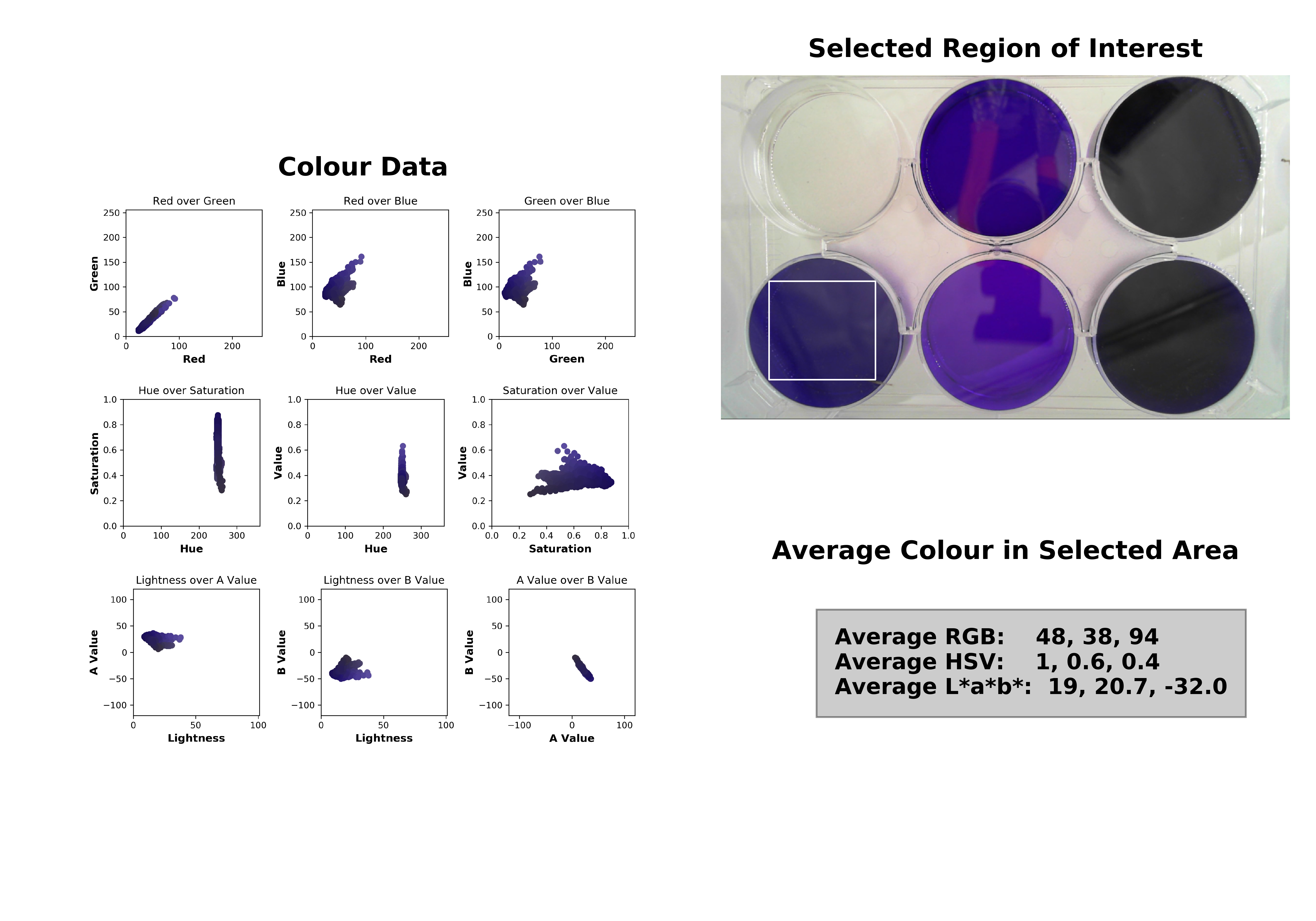

Supplement: Supplementary file 2 — Supporting Information [file ANIE-64-e202413395-s002.zip › Supporting Info - Machine readable data part 1/Figure 4 - glare analysis/6_below_SIanal/TILE_WITH_ROI.PNG]

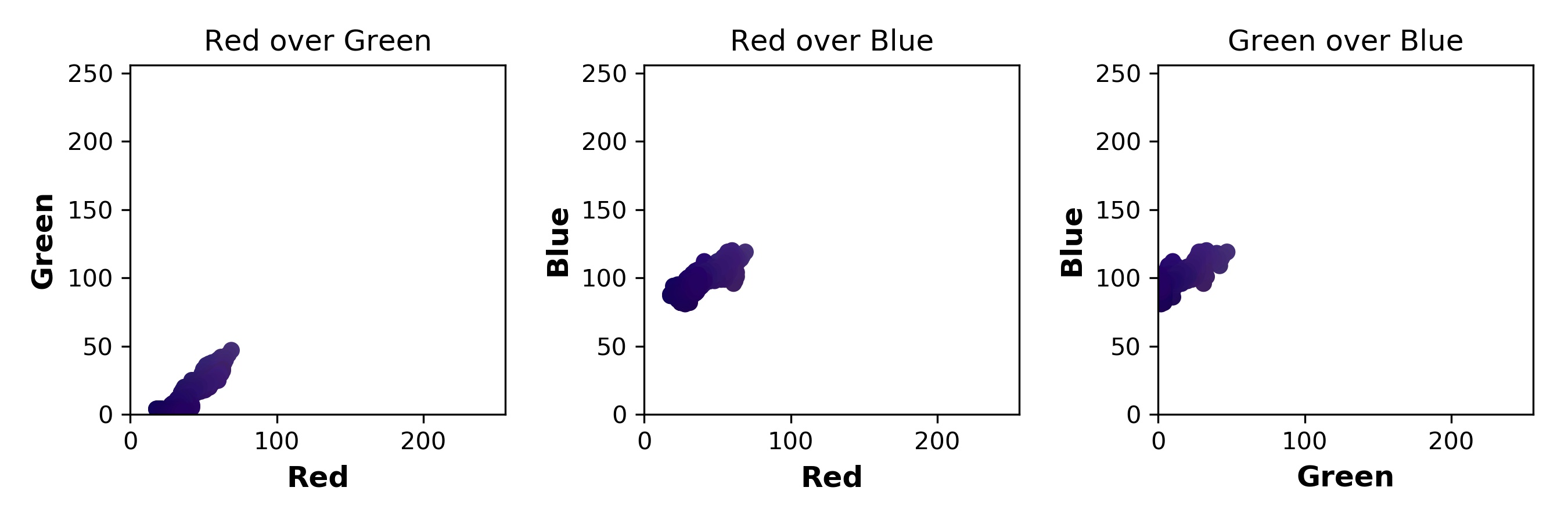

Supplement: Supplementary file 2 — Supporting Information [file ANIE-64-e202413395-s002.zip › Supporting Info - Machine readable data part 1/Figure 4 - glare analysis/24_below_SIanal__4/rgb.png]

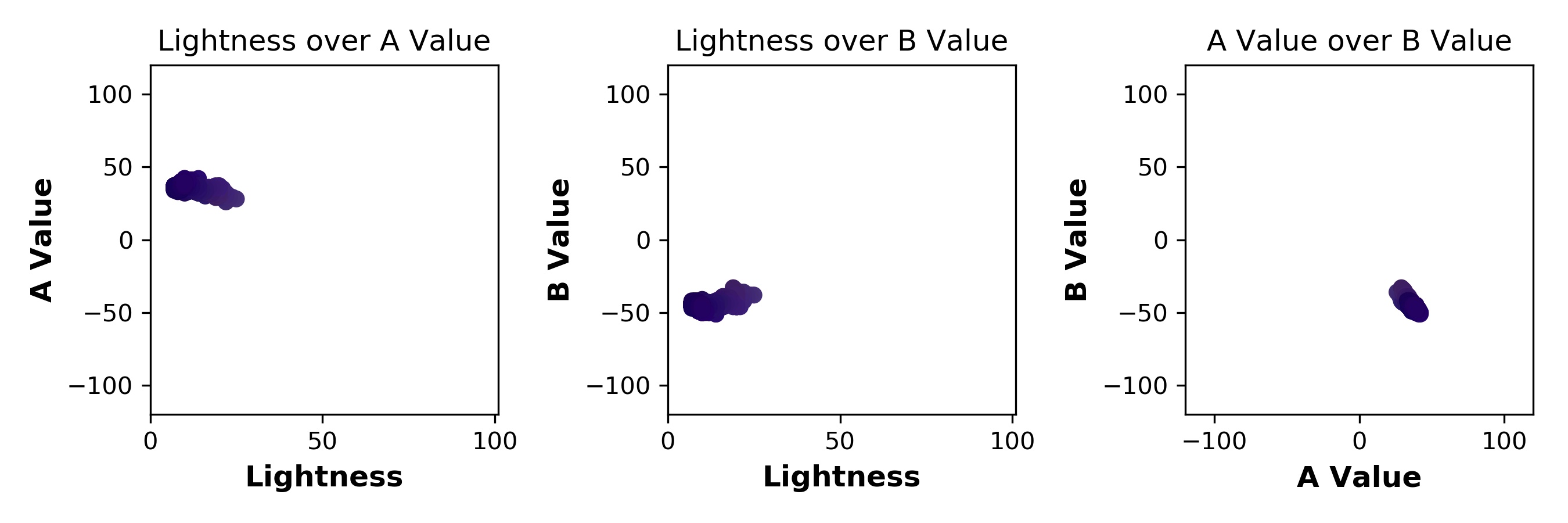

Supplement: Supplementary file 2 — Supporting Information [file ANIE-64-e202413395-s002.zip › Supporting Info - Machine readable data part 1/Figure 4 - glare analysis/24_below_SIanal__4/lab.png]

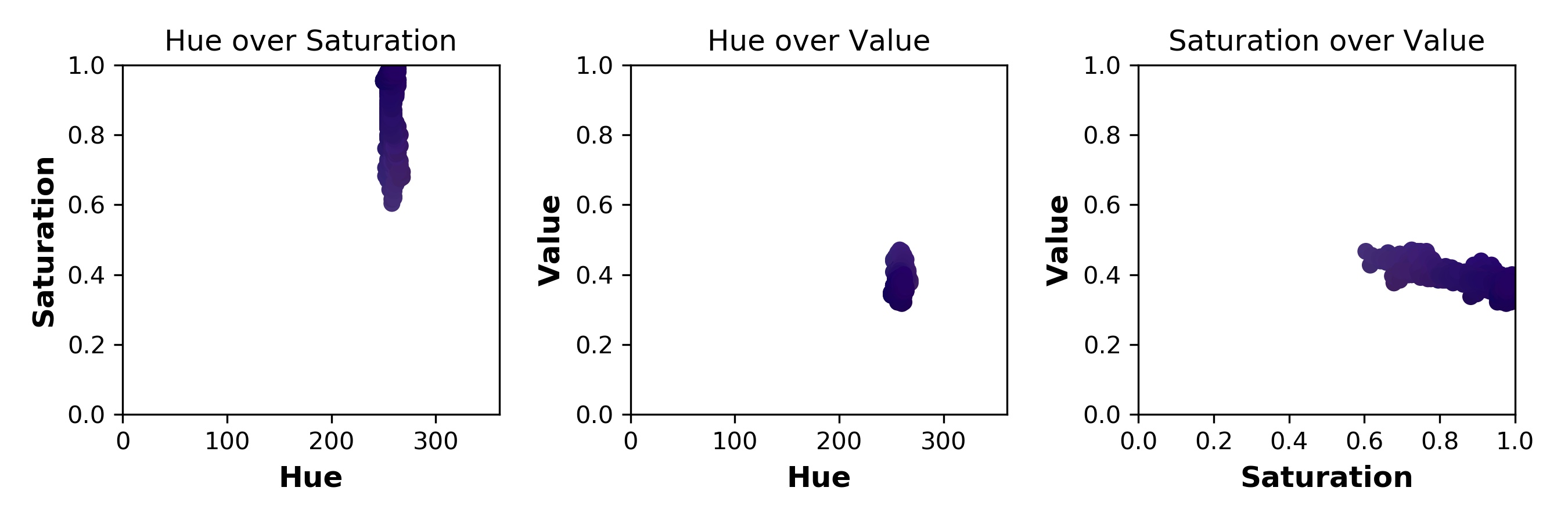

Supplement: Supplementary file 2 — Supporting Information [file ANIE-64-e202413395-s002.zip › Supporting Info - Machine readable data part 1/Figure 4 - glare analysis/24_below_SIanal__4/hsv.png]

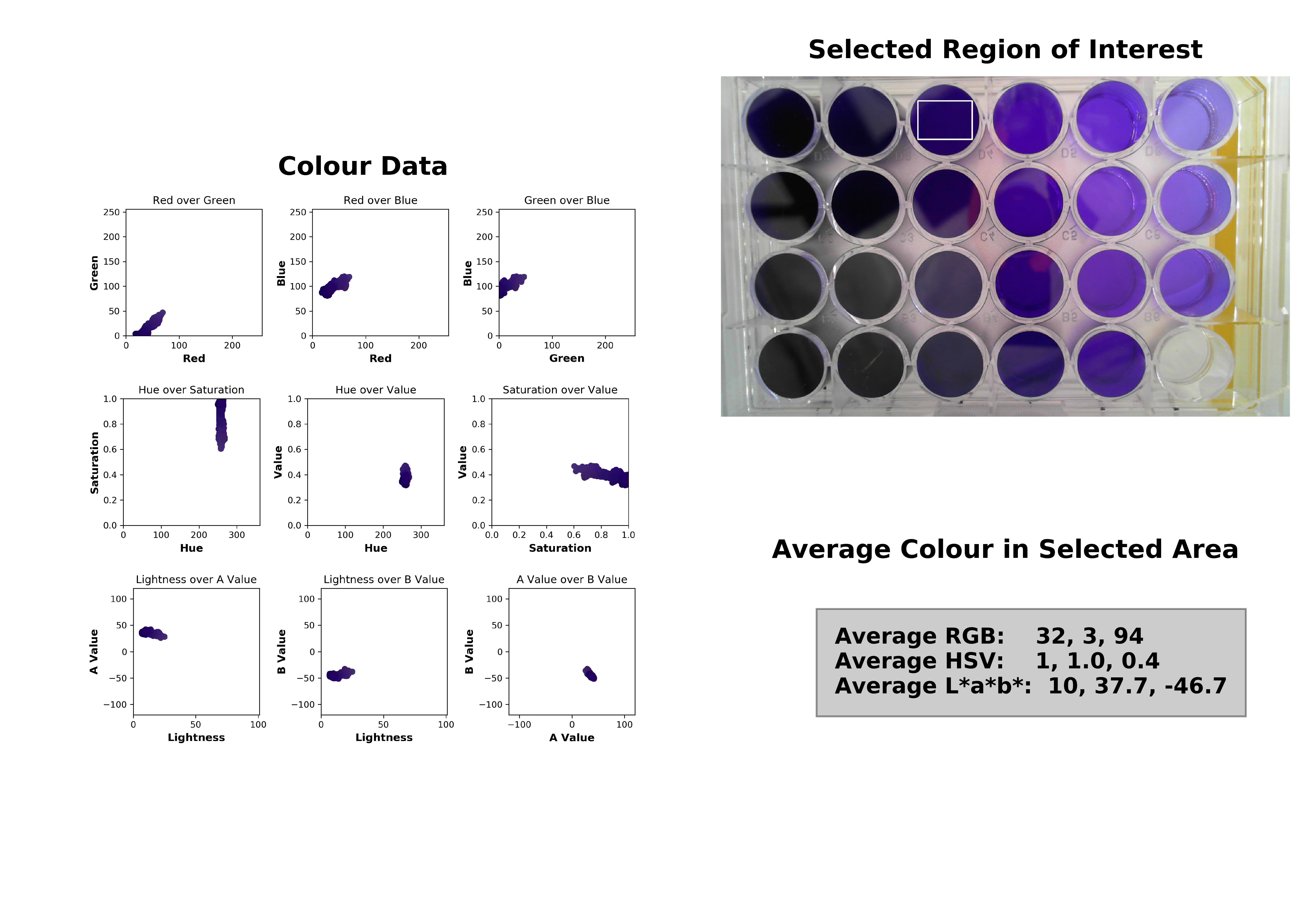

Supplement: Supplementary file 2 — Supporting Information [file ANIE-64-e202413395-s002.zip › Supporting Info - Machine readable data part 1/Figure 4 - glare analysis/24_below_SIanal__4/TILE_WITH_ROI.PNG]

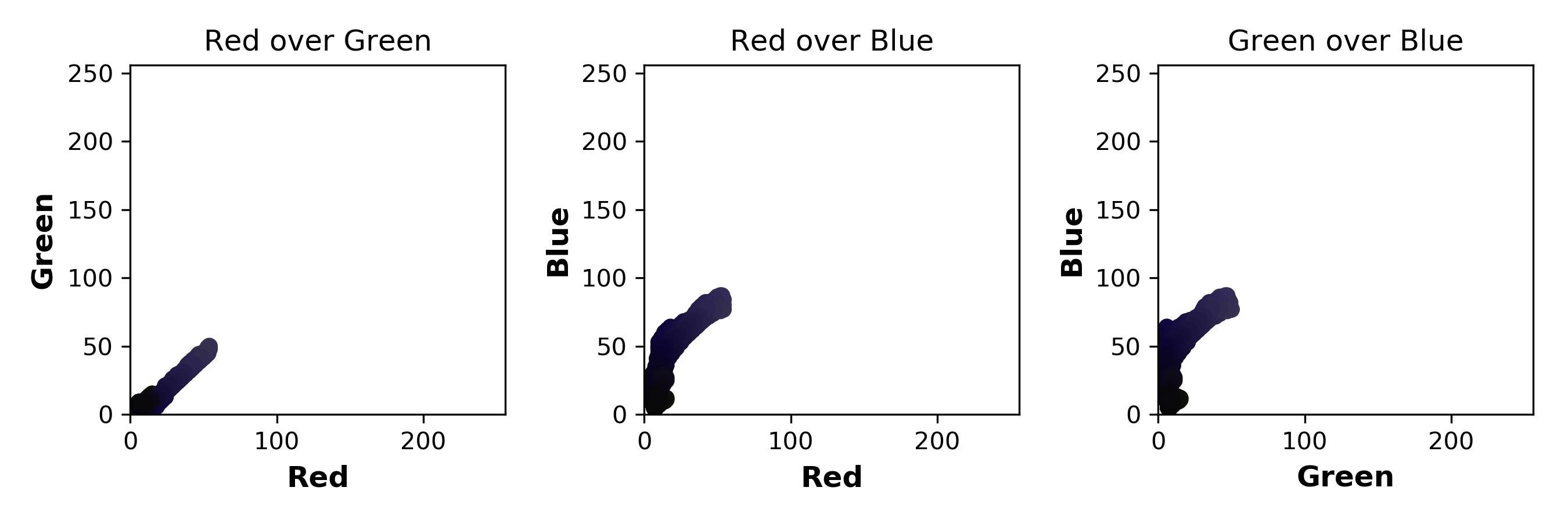

Supplement: Supplementary file 2 — Supporting Information [file ANIE-64-e202413395-s002.zip › Supporting Info - Machine readable data part 1/Figure 4 - glare analysis/24_below_SIanal__3/rgb.png]

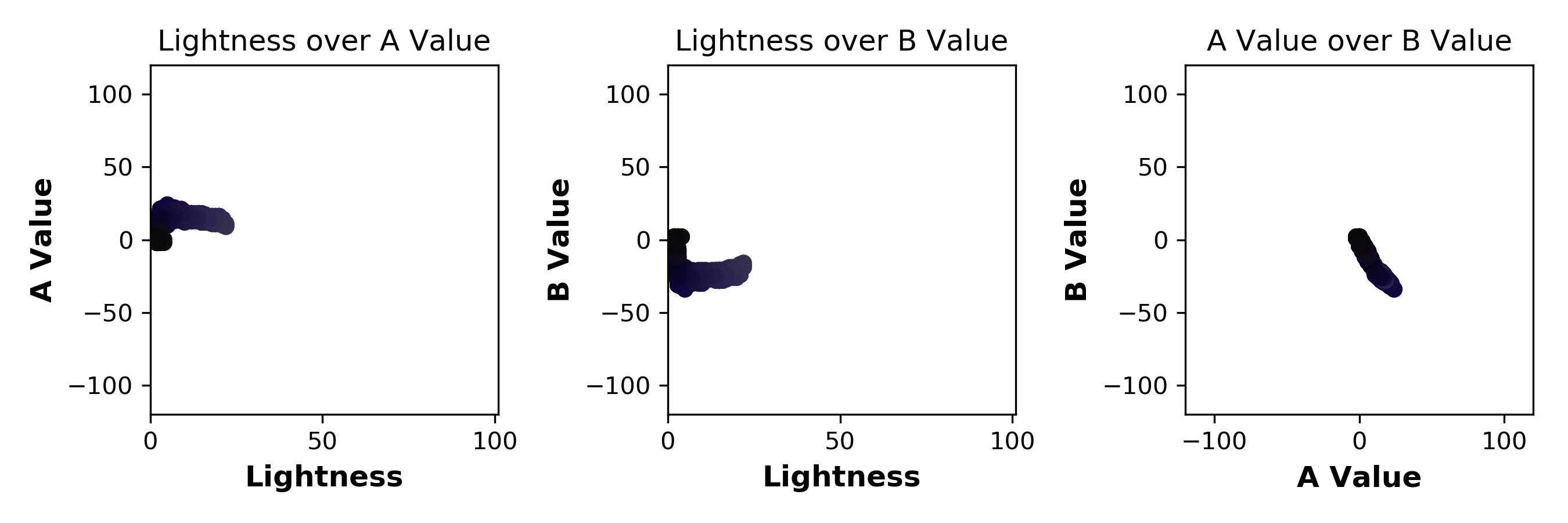

Supplement: Supplementary file 2 — Supporting Information [file ANIE-64-e202413395-s002.zip › Supporting Info - Machine readable data part 1/Figure 4 - glare analysis/24_below_SIanal__3/lab.png]

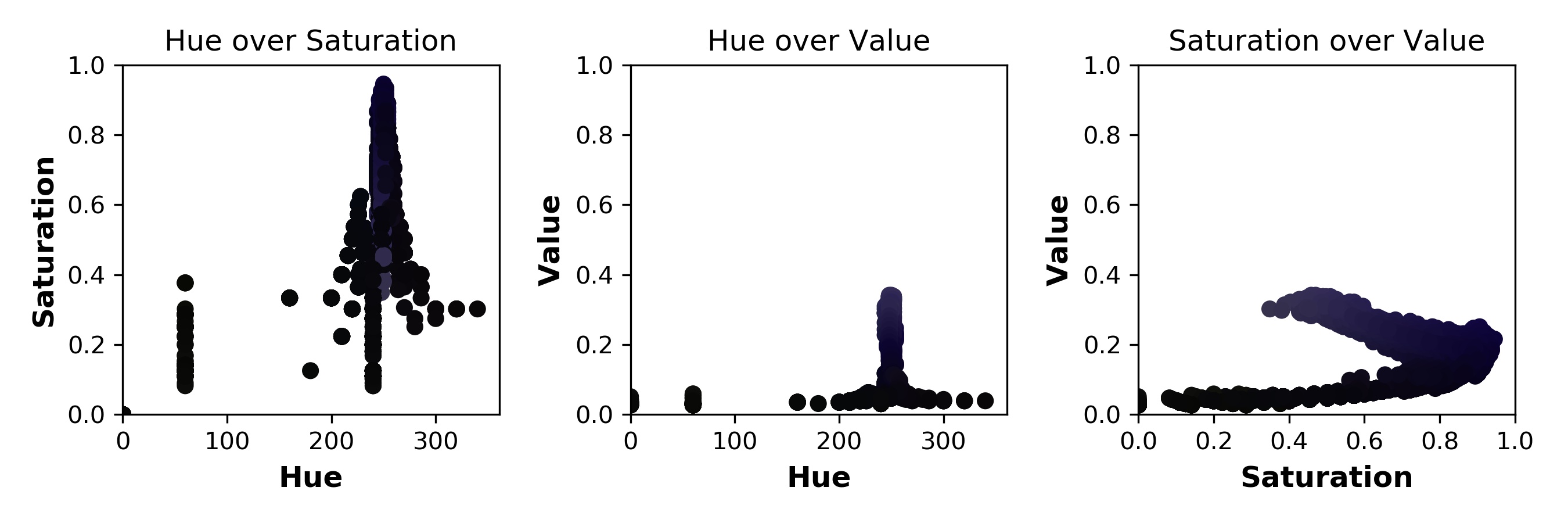

Supplement: Supplementary file 2 — Supporting Information [file ANIE-64-e202413395-s002.zip › Supporting Info - Machine readable data part 1/Figure 4 - glare analysis/24_below_SIanal__3/hsv.png]

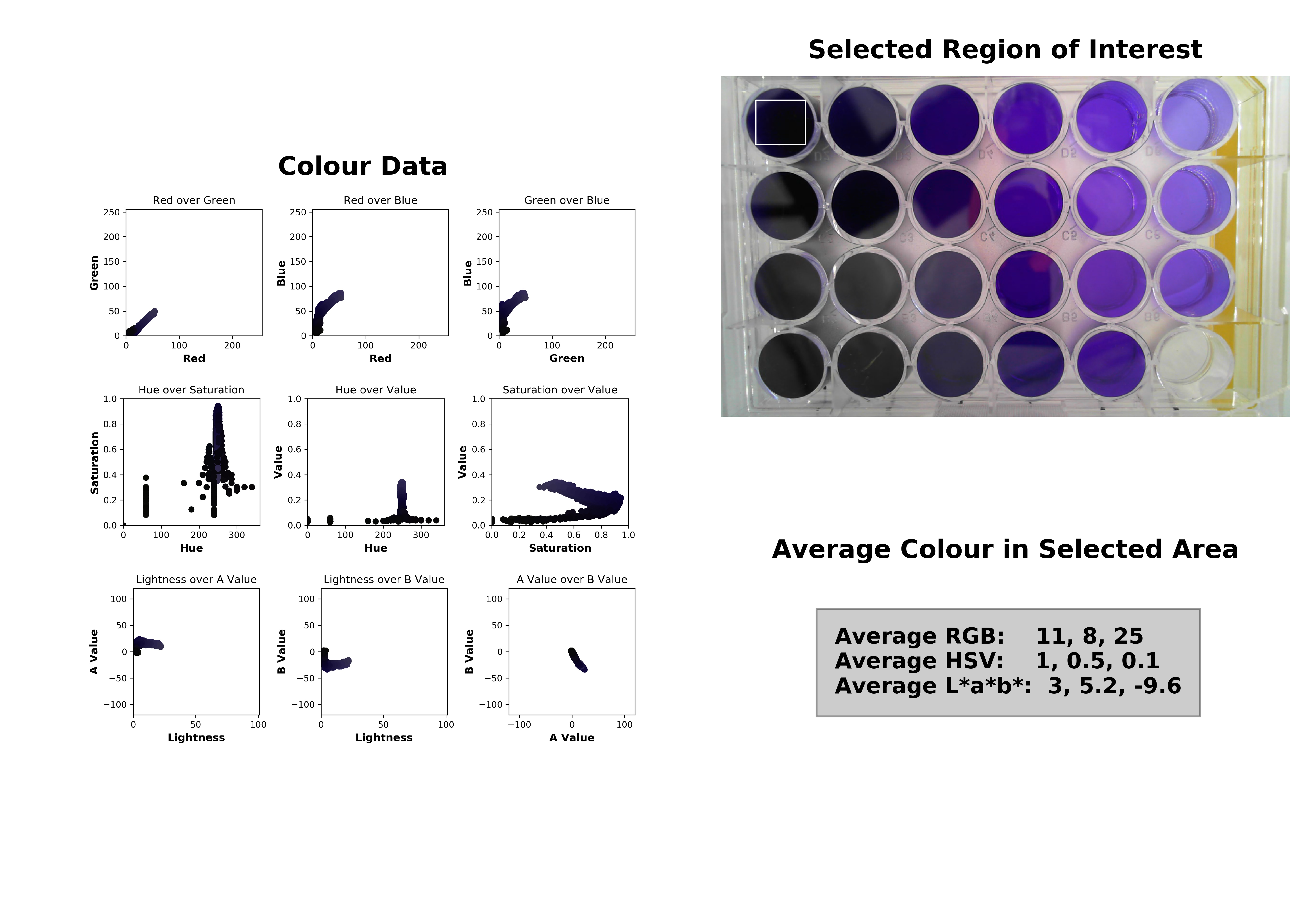

Supplement: Supplementary file 2 — Supporting Information [file ANIE-64-e202413395-s002.zip › Supporting Info - Machine readable data part 1/Figure 4 - glare analysis/24_below_SIanal__3/TILE_WITH_ROI.PNG]

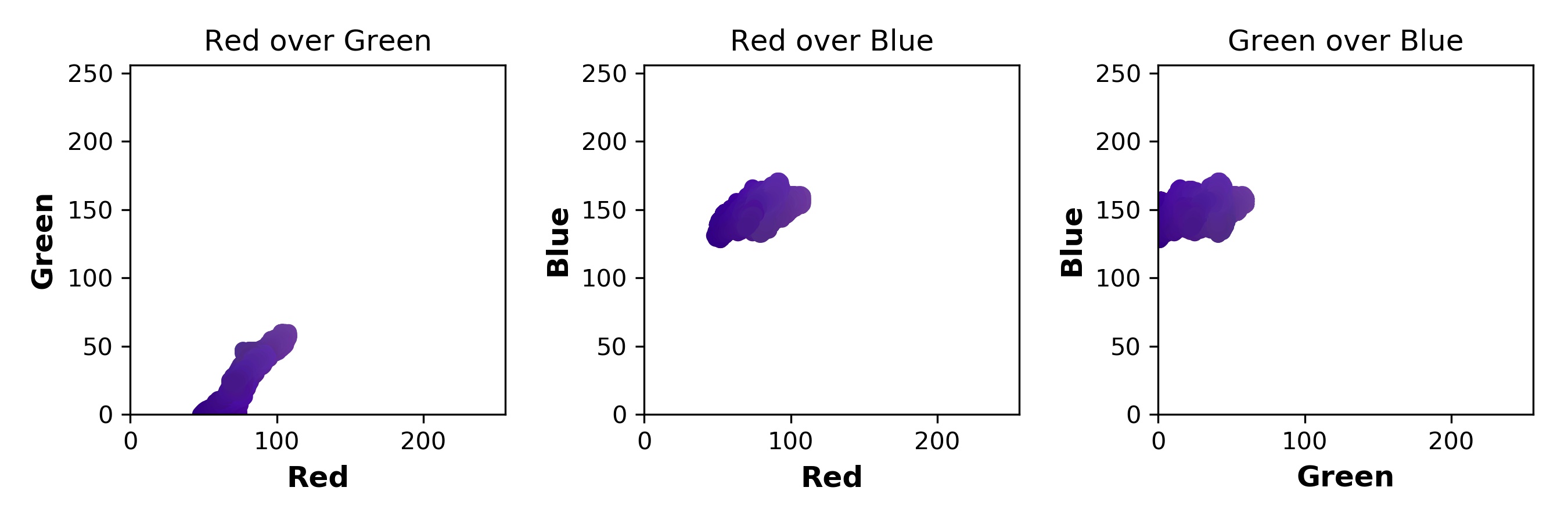

Supplement: Supplementary file 2 — Supporting Information [file ANIE-64-e202413395-s002.zip › Supporting Info - Machine readable data part 1/Figure 4 - glare analysis/6_below_SIanal__2/rgb.png]

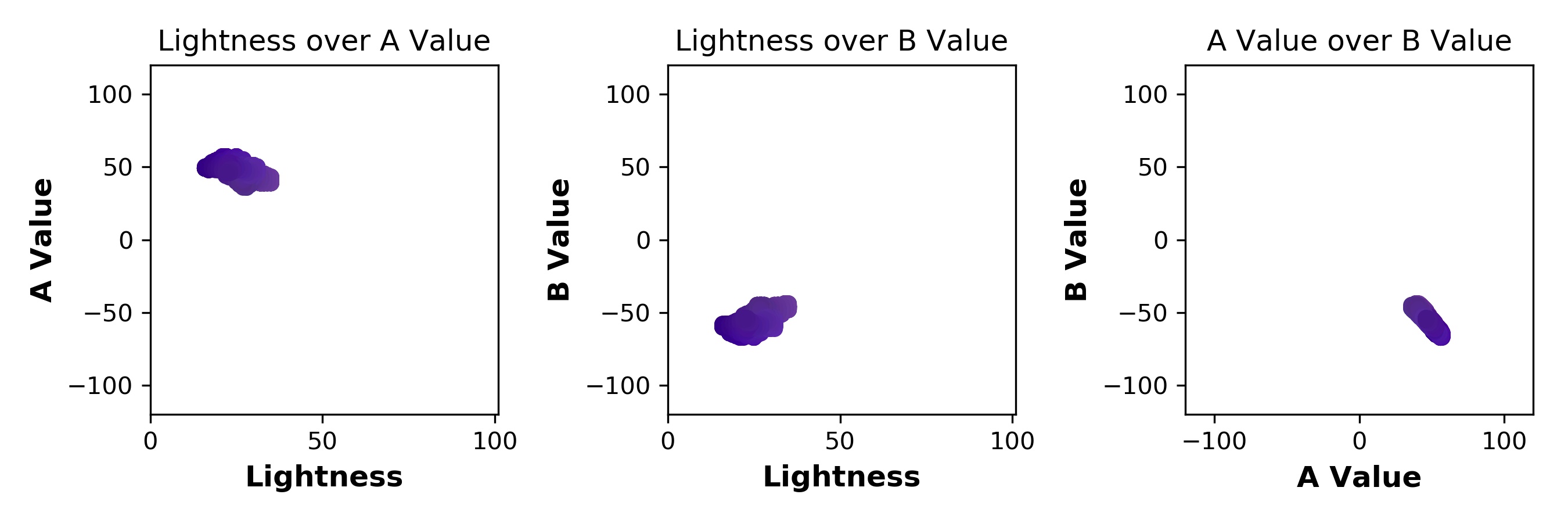

Supplement: Supplementary file 2 — Supporting Information [file ANIE-64-e202413395-s002.zip › Supporting Info - Machine readable data part 1/Figure 4 - glare analysis/6_below_SIanal__2/lab.png]

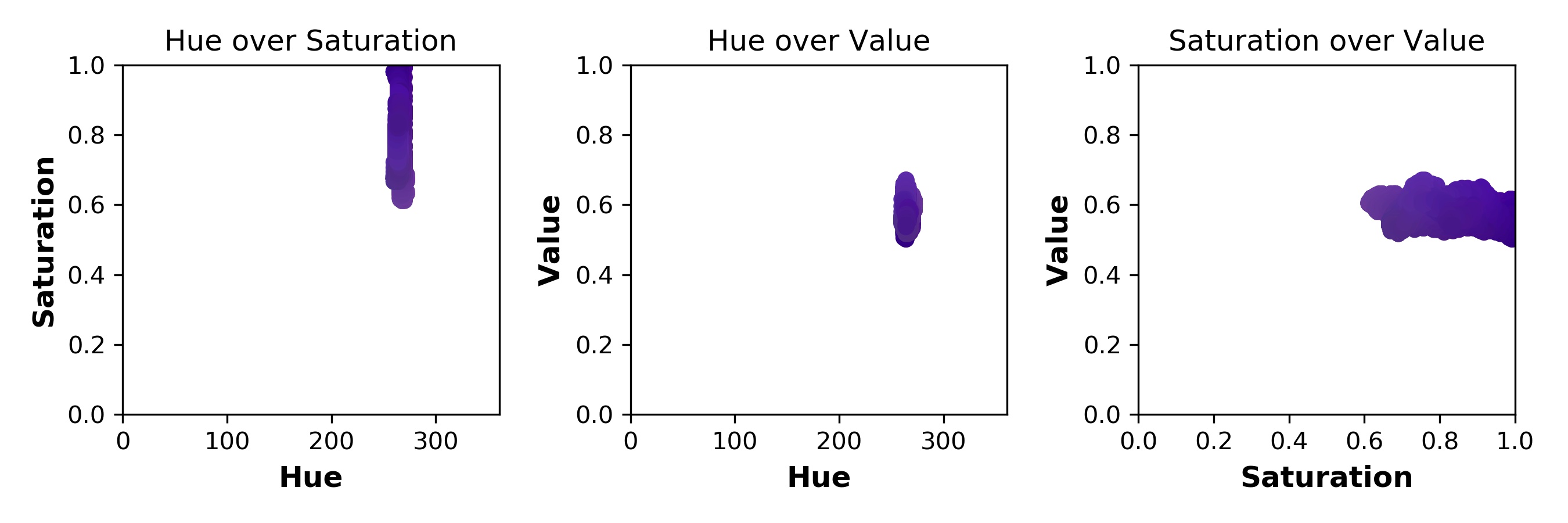

Supplement: Supplementary file 2 — Supporting Information [file ANIE-64-e202413395-s002.zip › Supporting Info - Machine readable data part 1/Figure 4 - glare analysis/6_below_SIanal__2/hsv.png]

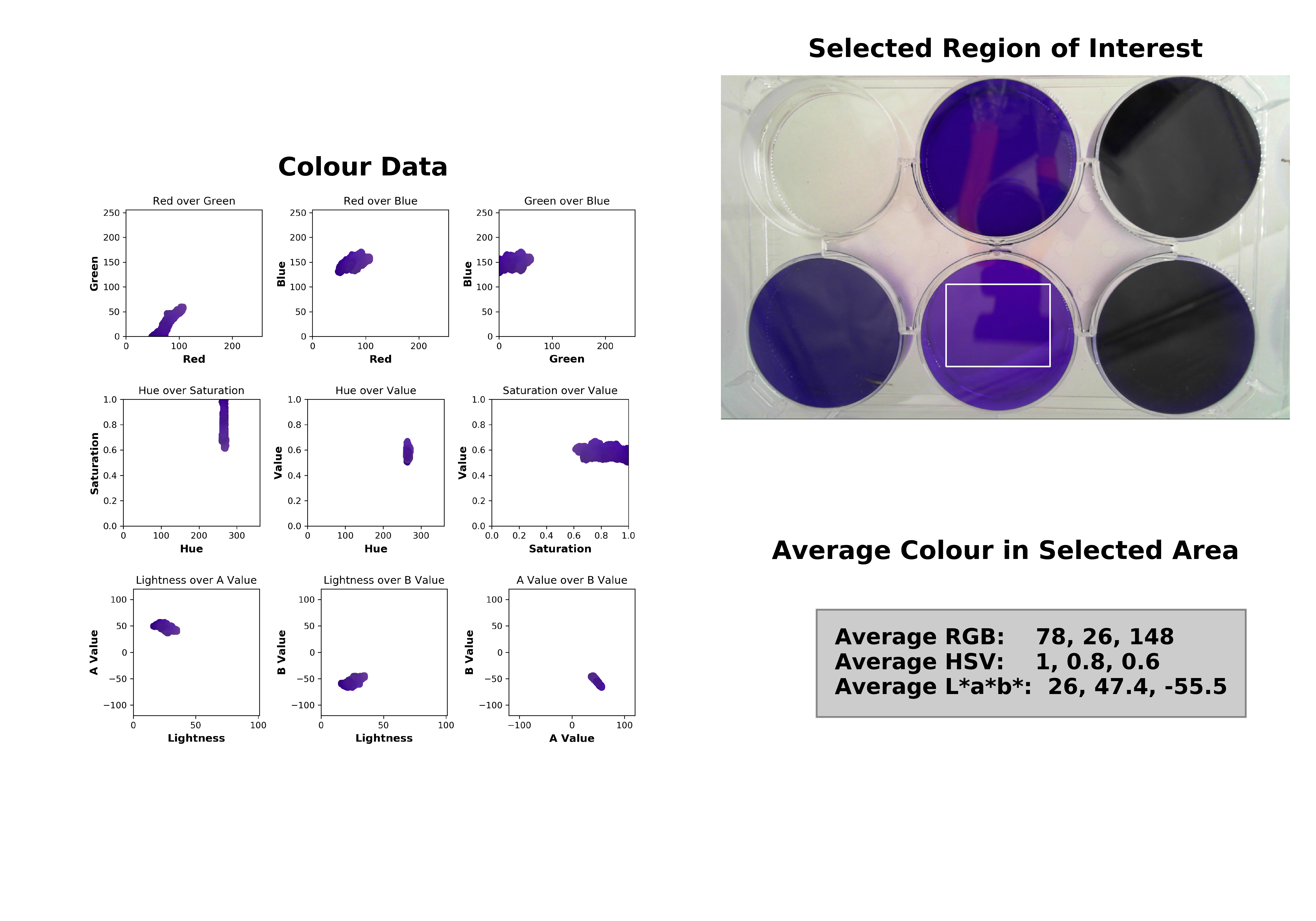

Supplement: Supplementary file 2 — Supporting Information [file ANIE-64-e202413395-s002.zip › Supporting Info - Machine readable data part 1/Figure 4 - glare analysis/6_below_SIanal__2/TILE_WITH_ROI.PNG]

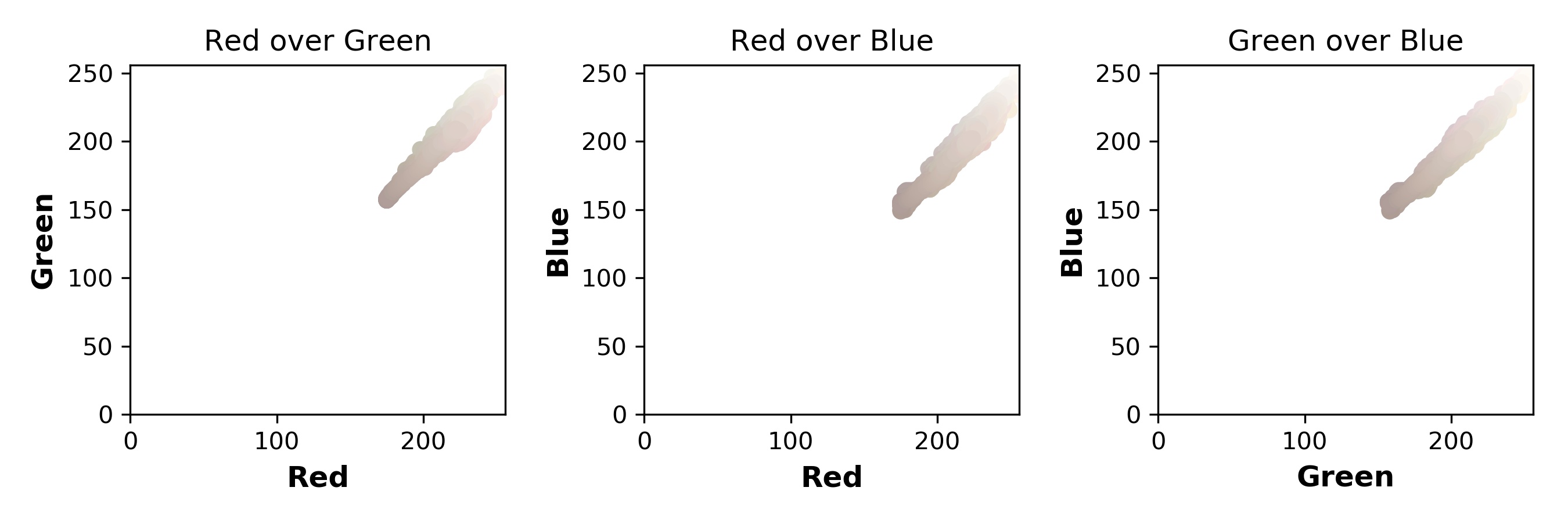

Supplement: Supplementary file 2 — Supporting Information [file ANIE-64-e202413395-s002.zip › Supporting Info - Machine readable data part 1/Figure 4 - glare analysis/6_above_SIanal__2/rgb.png]

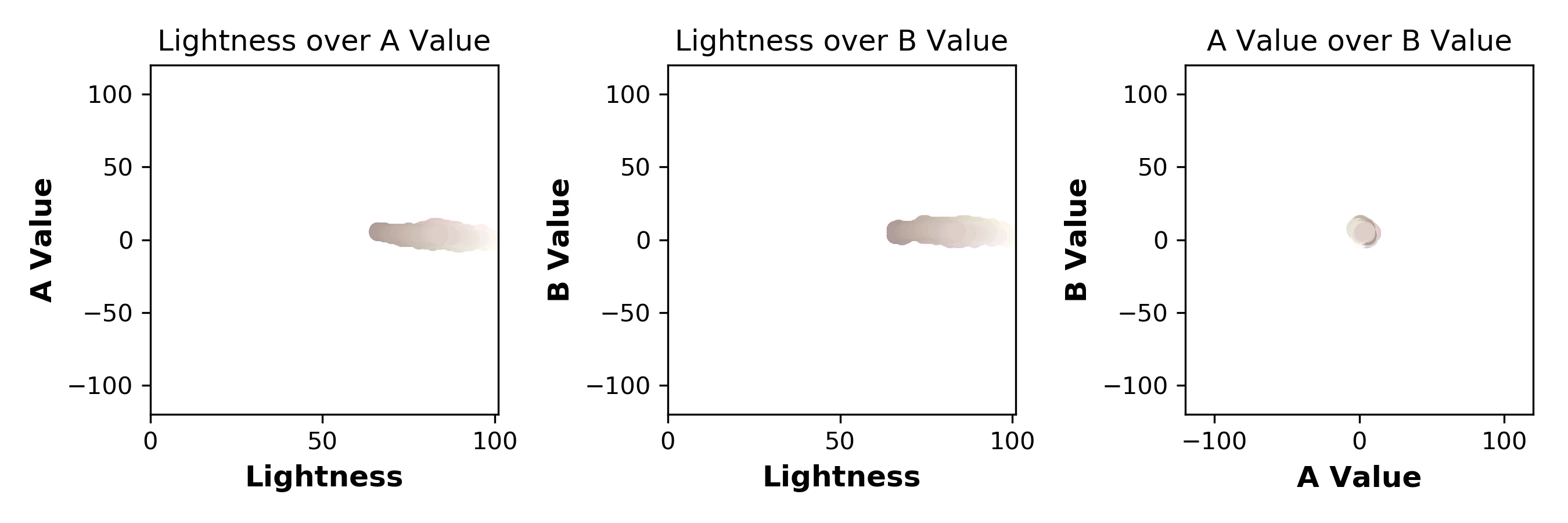

Supplement: Supplementary file 2 — Supporting Information [file ANIE-64-e202413395-s002.zip › Supporting Info - Machine readable data part 1/Figure 4 - glare analysis/6_above_SIanal__2/lab.png]

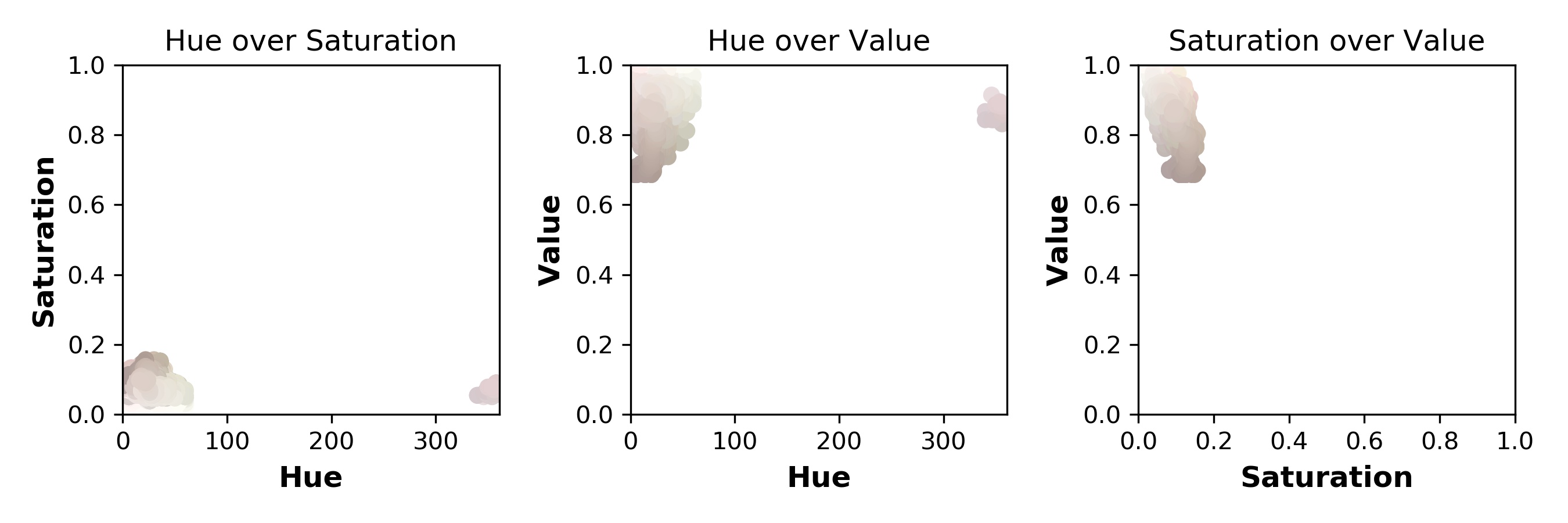

Supplement: Supplementary file 2 — Supporting Information [file ANIE-64-e202413395-s002.zip › Supporting Info - Machine readable data part 1/Figure 4 - glare analysis/6_above_SIanal__2/hsv.png]

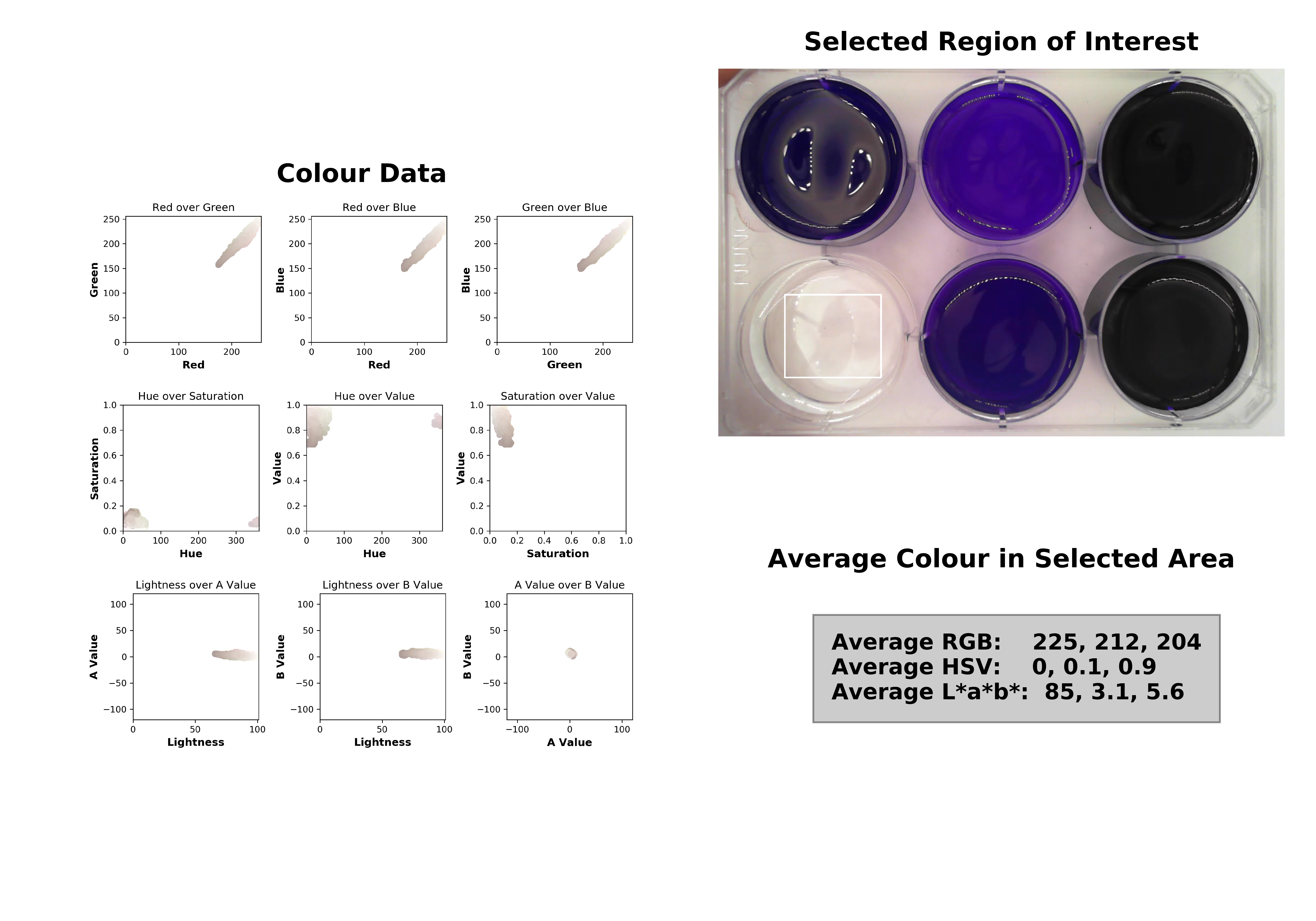

Supplement: Supplementary file 2 — Supporting Information [file ANIE-64-e202413395-s002.zip › Supporting Info - Machine readable data part 1/Figure 4 - glare analysis/6_above_SIanal__2/TILE_WITH_ROI.PNG]

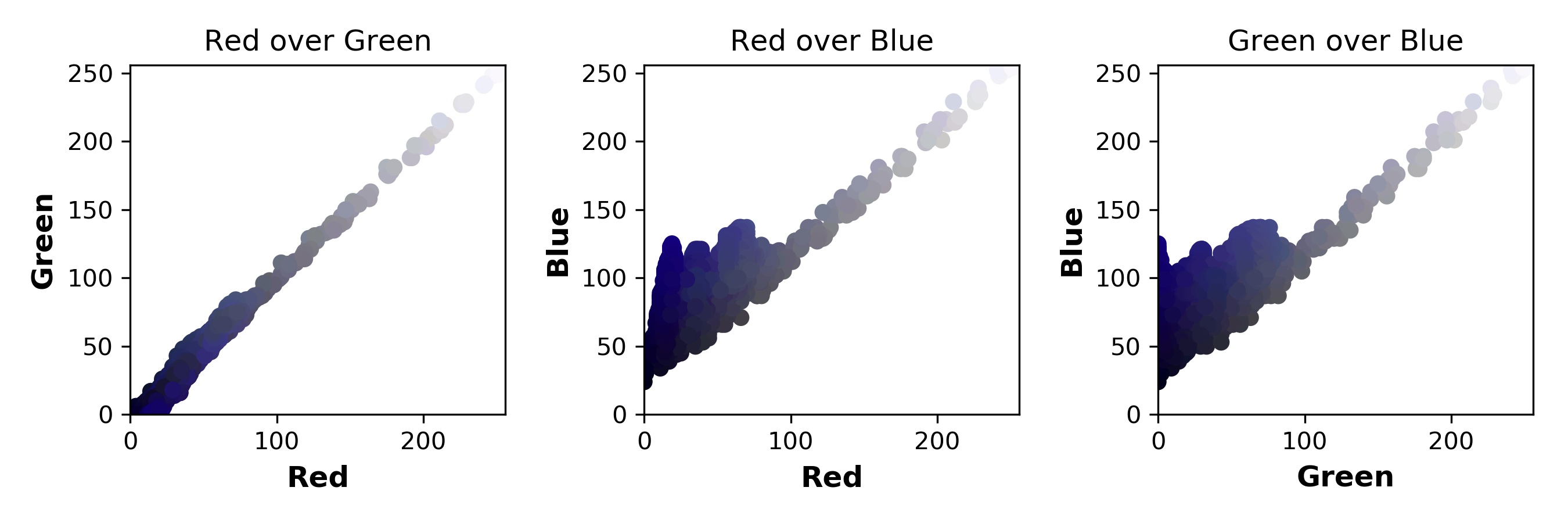

Supplement: Supplementary file 2 — Supporting Information [file ANIE-64-e202413395-s002.zip › Supporting Info - Machine readable data part 1/Figure 4 - glare analysis/24_above_SIanal__4/rgb.png]

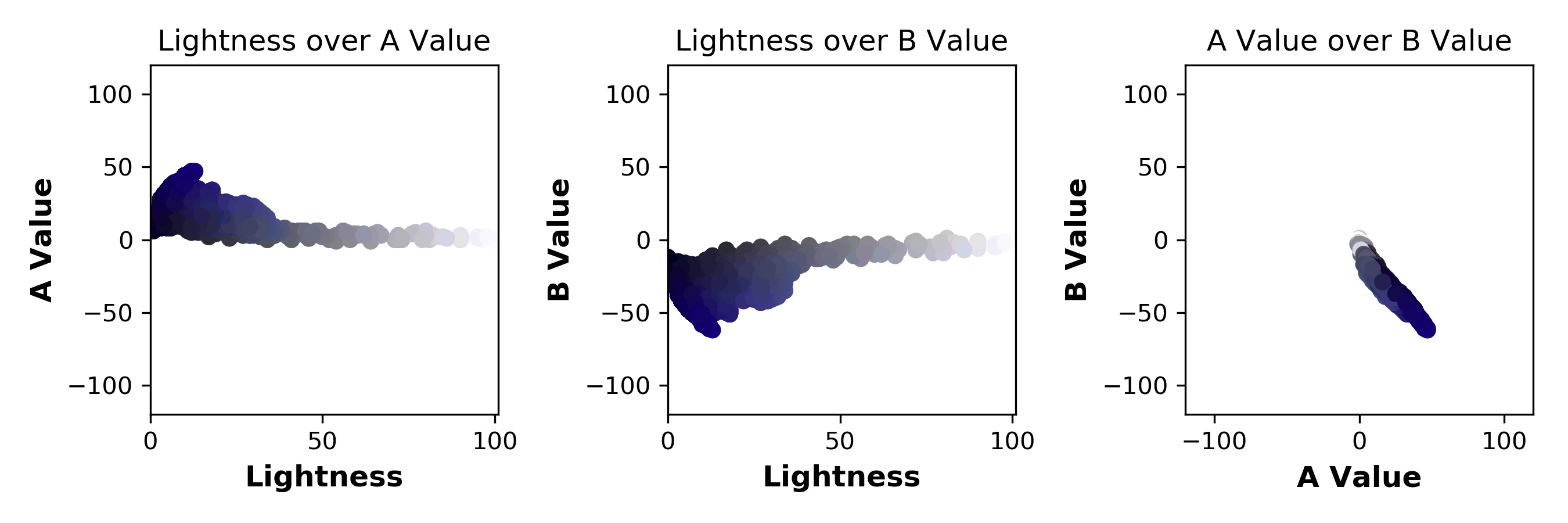

Supplement: Supplementary file 2 — Supporting Information [file ANIE-64-e202413395-s002.zip › Supporting Info - Machine readable data part 1/Figure 4 - glare analysis/24_above_SIanal__4/lab.png]

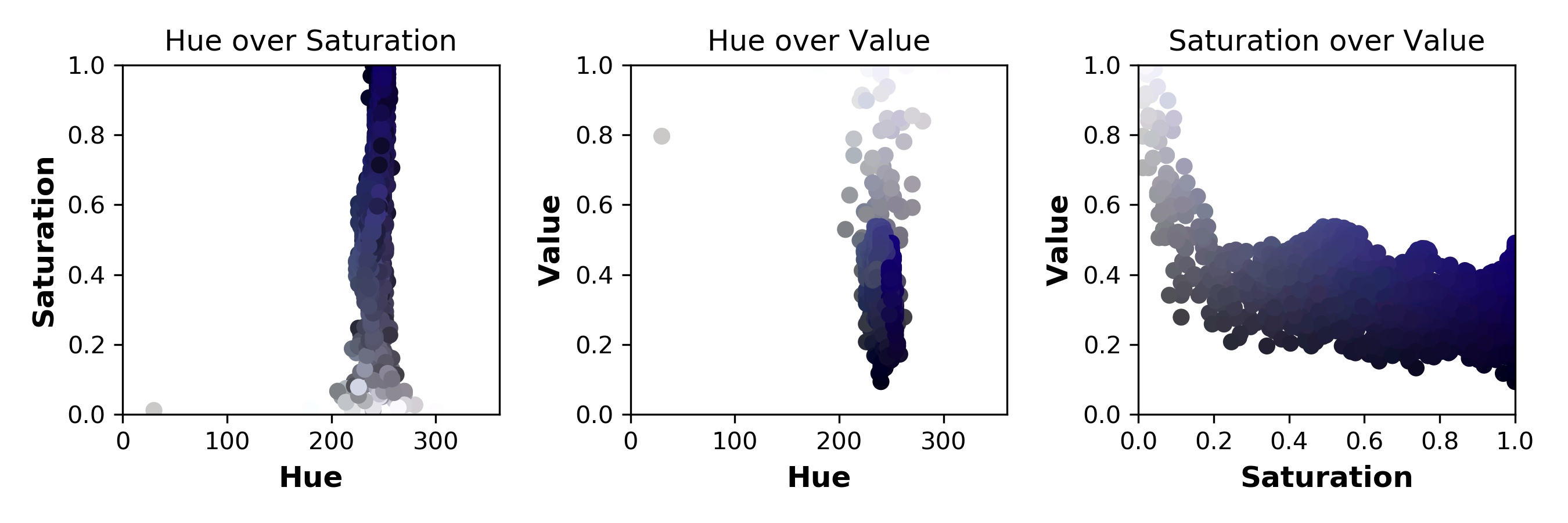

Supplement: Supplementary file 2 — Supporting Information [file ANIE-64-e202413395-s002.zip › Supporting Info - Machine readable data part 1/Figure 4 - glare analysis/24_above_SIanal__4/hsv.png]

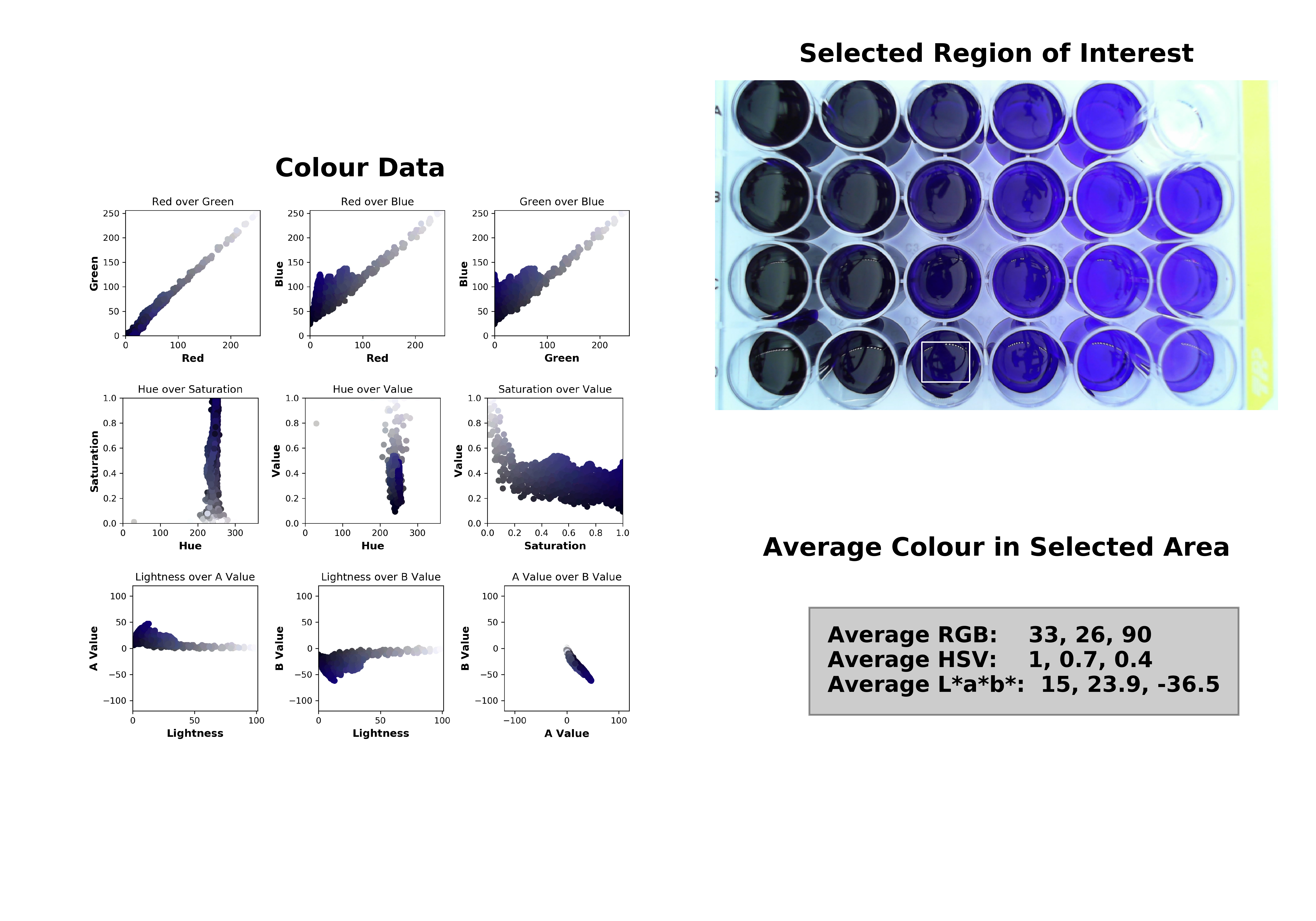

Supplement: Supplementary file 2 — Supporting Information [file ANIE-64-e202413395-s002.zip › Supporting Info - Machine readable data part 1/Figure 4 - glare analysis/24_above_SIanal__4/TILE_WITH_ROI.PNG]

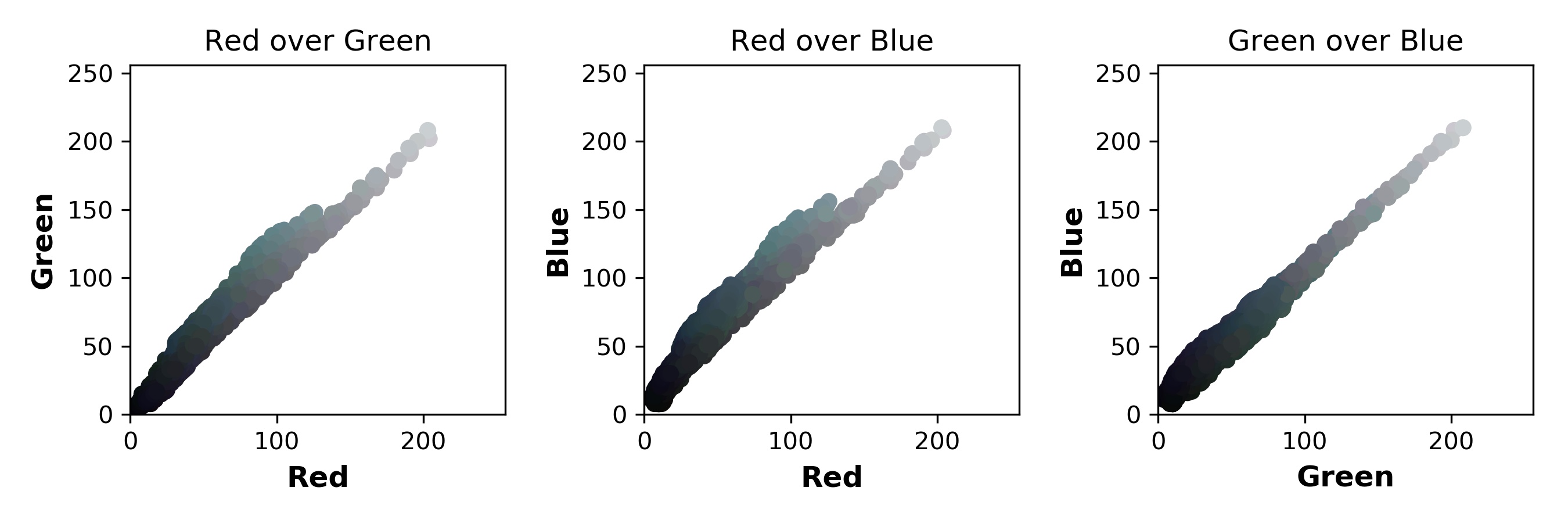

Supplement: Supplementary file 2 — Supporting Information [file ANIE-64-e202413395-s002.zip › Supporting Info - Machine readable data part 1/Figure 4 - glare analysis/24_above_SIanal__3/rgb.png]

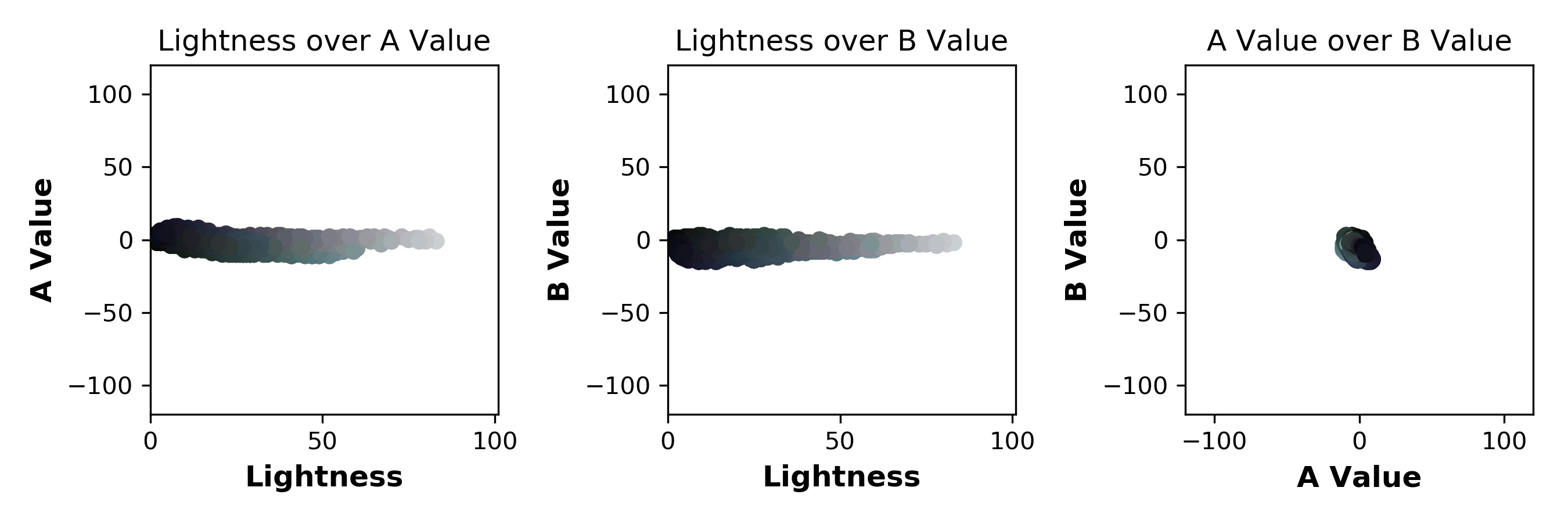

Supplement: Supplementary file 2 — Supporting Information [file ANIE-64-e202413395-s002.zip › Supporting Info - Machine readable data part 1/Figure 4 - glare analysis/24_above_SIanal__3/lab.png]

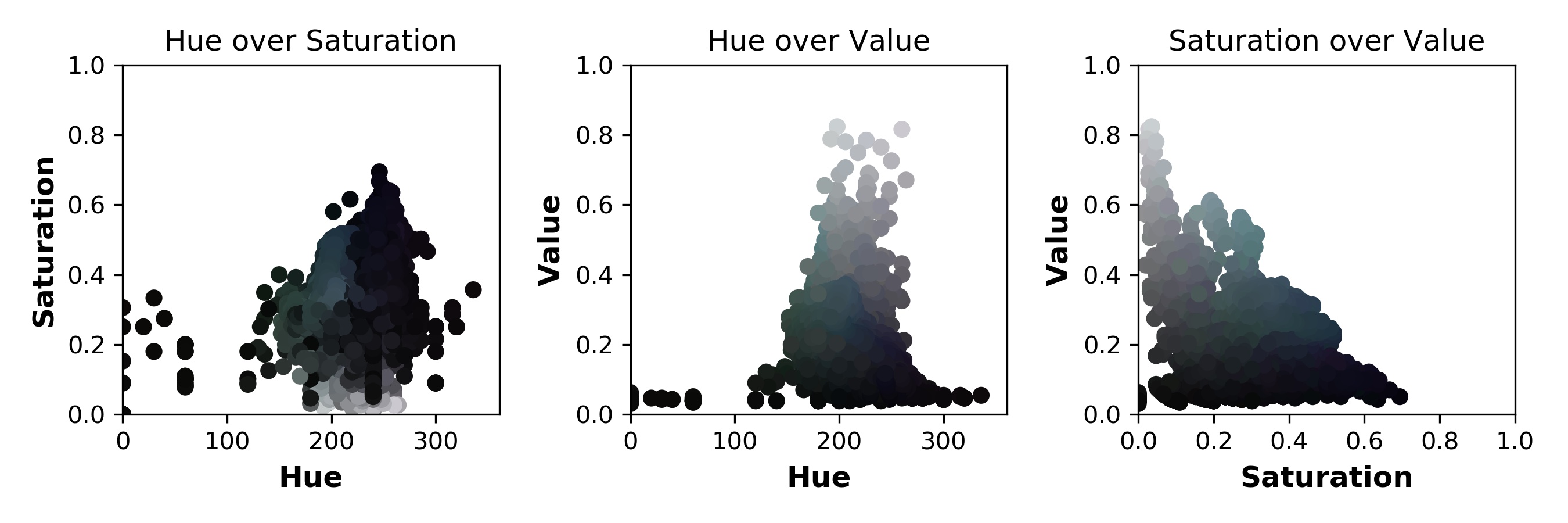

Supplement: Supplementary file 2 — Supporting Information [file ANIE-64-e202413395-s002.zip › Supporting Info - Machine readable data part 1/Figure 4 - glare analysis/24_above_SIanal__3/hsv.png]

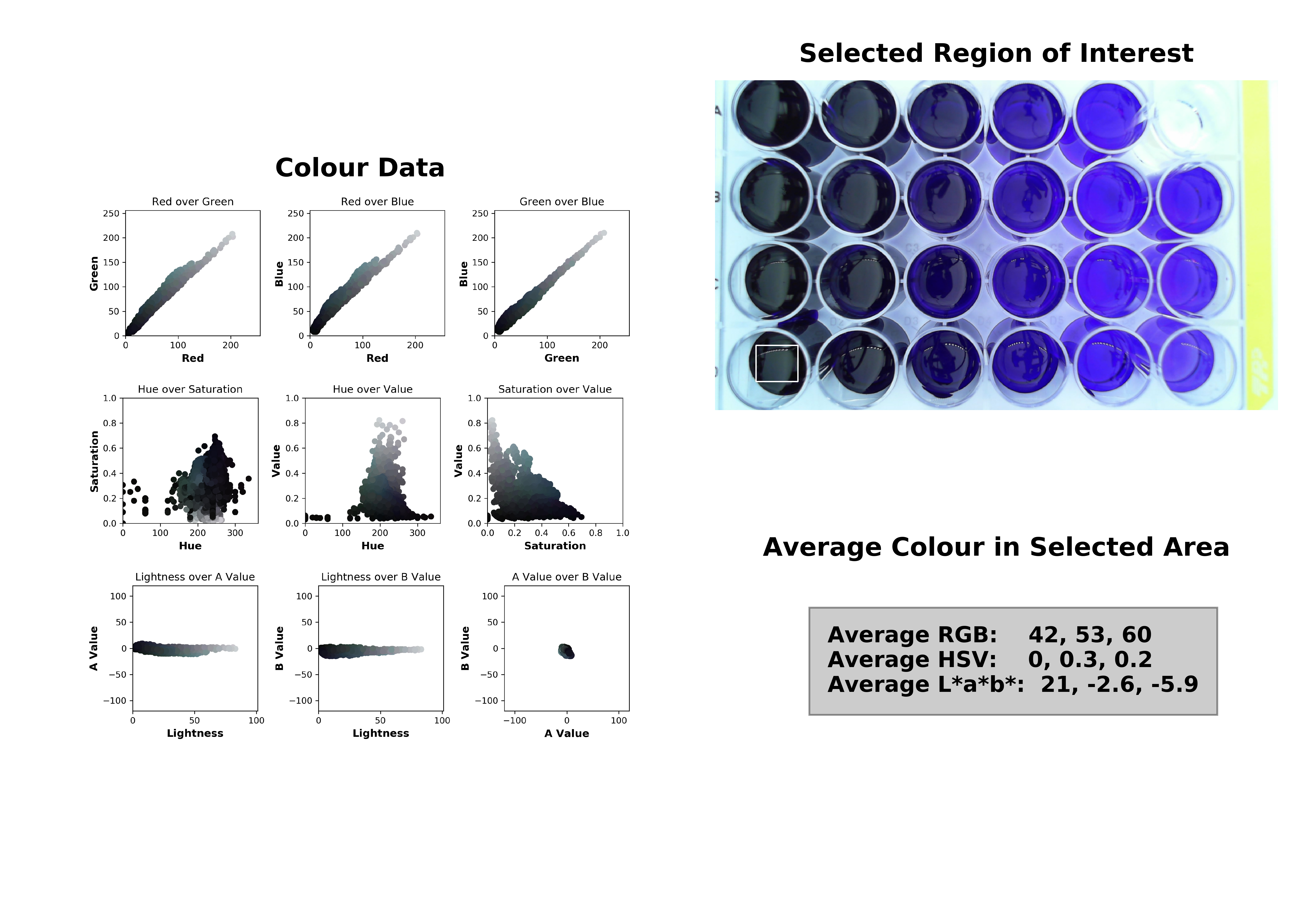

Supplement: Supplementary file 2 — Supporting Information [file ANIE-64-e202413395-s002.zip › Supporting Info - Machine readable data part 1/Figure 4 - glare analysis/24_above_SIanal__3/TILE_WITH_ROI.PNG]

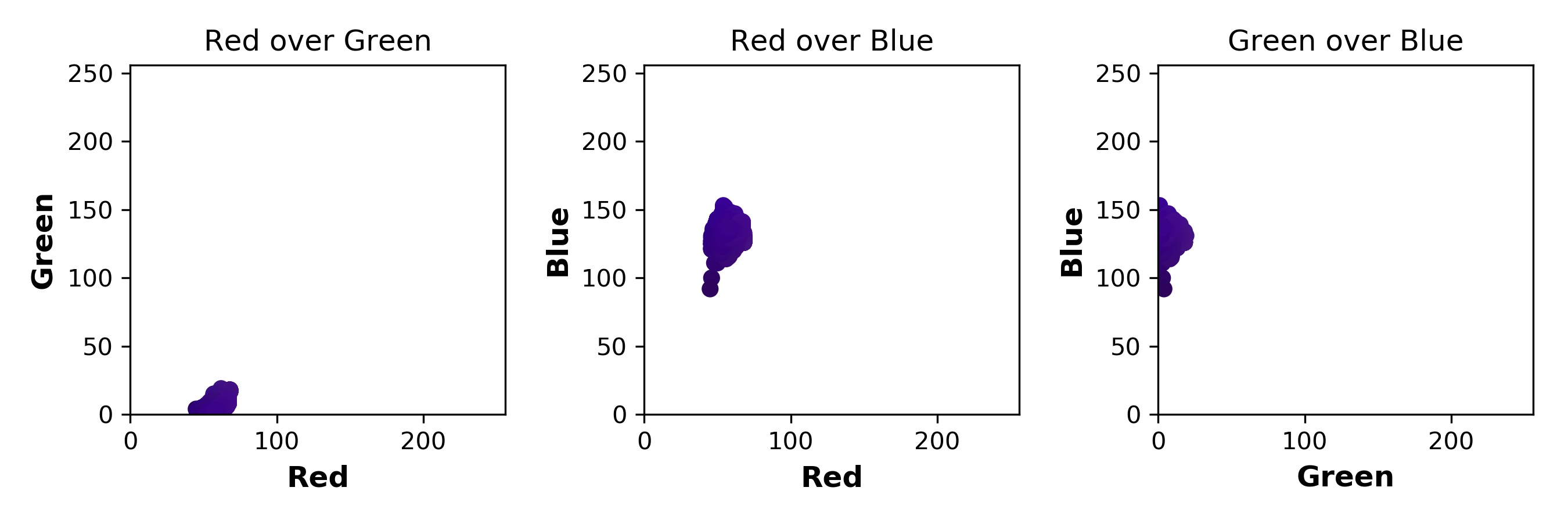

Supplement: Supplementary file 2 — Supporting Information [file ANIE-64-e202413395-s002.zip › Supporting Info - Machine readable data part 1/Figure 4 - glare analysis/6_above_SIanal__3/rgb.png]

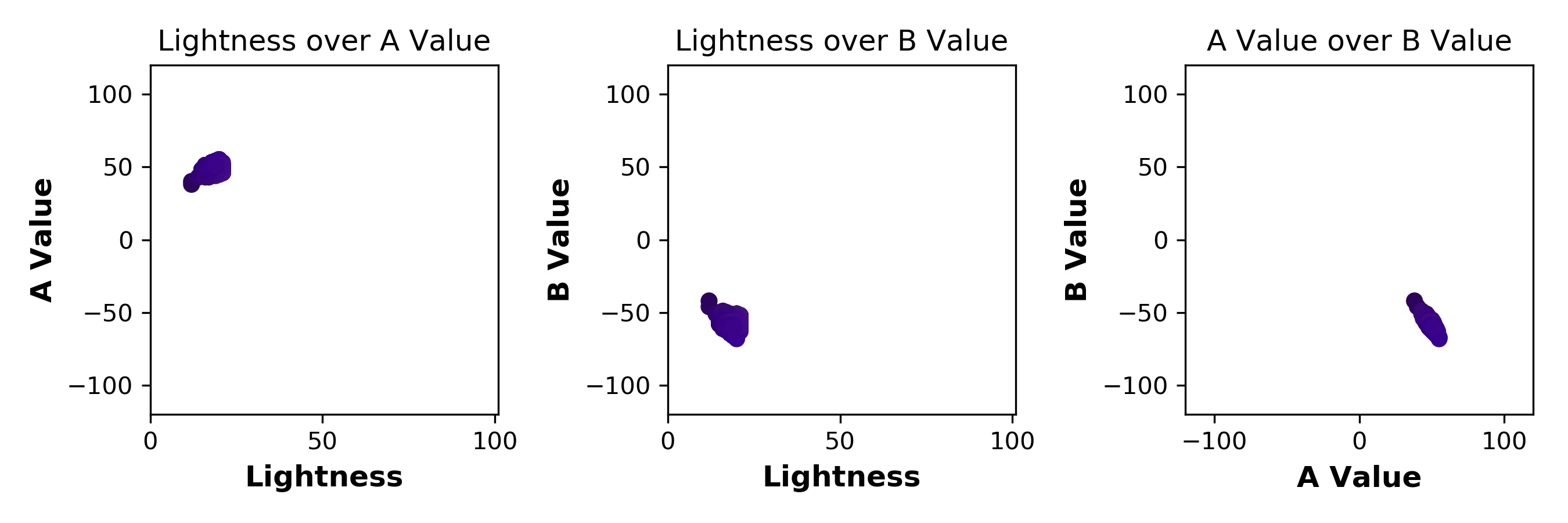

Supplement: Supplementary file 2 — Supporting Information [file ANIE-64-e202413395-s002.zip › Supporting Info - Machine readable data part 1/Figure 4 - glare analysis/6_above_SIanal__3/lab.png]

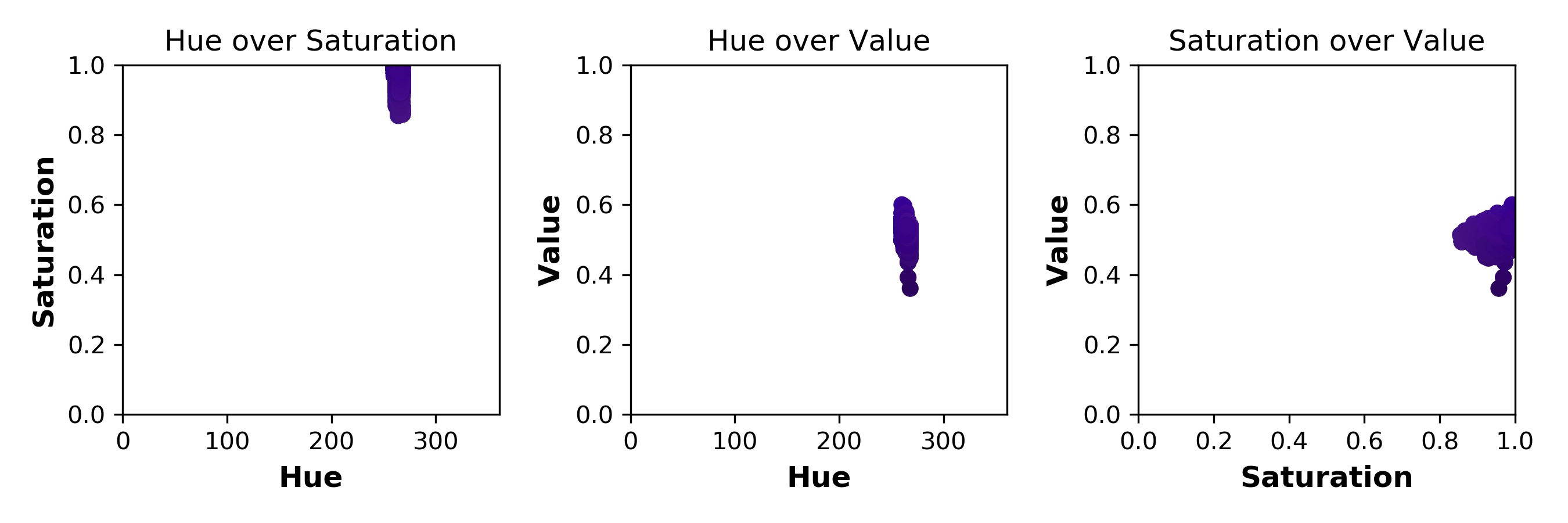

Supplement: Supplementary file 2 — Supporting Information [file ANIE-64-e202413395-s002.zip › Supporting Info - Machine readable data part 1/Figure 4 - glare analysis/6_above_SIanal__3/hsv.png]

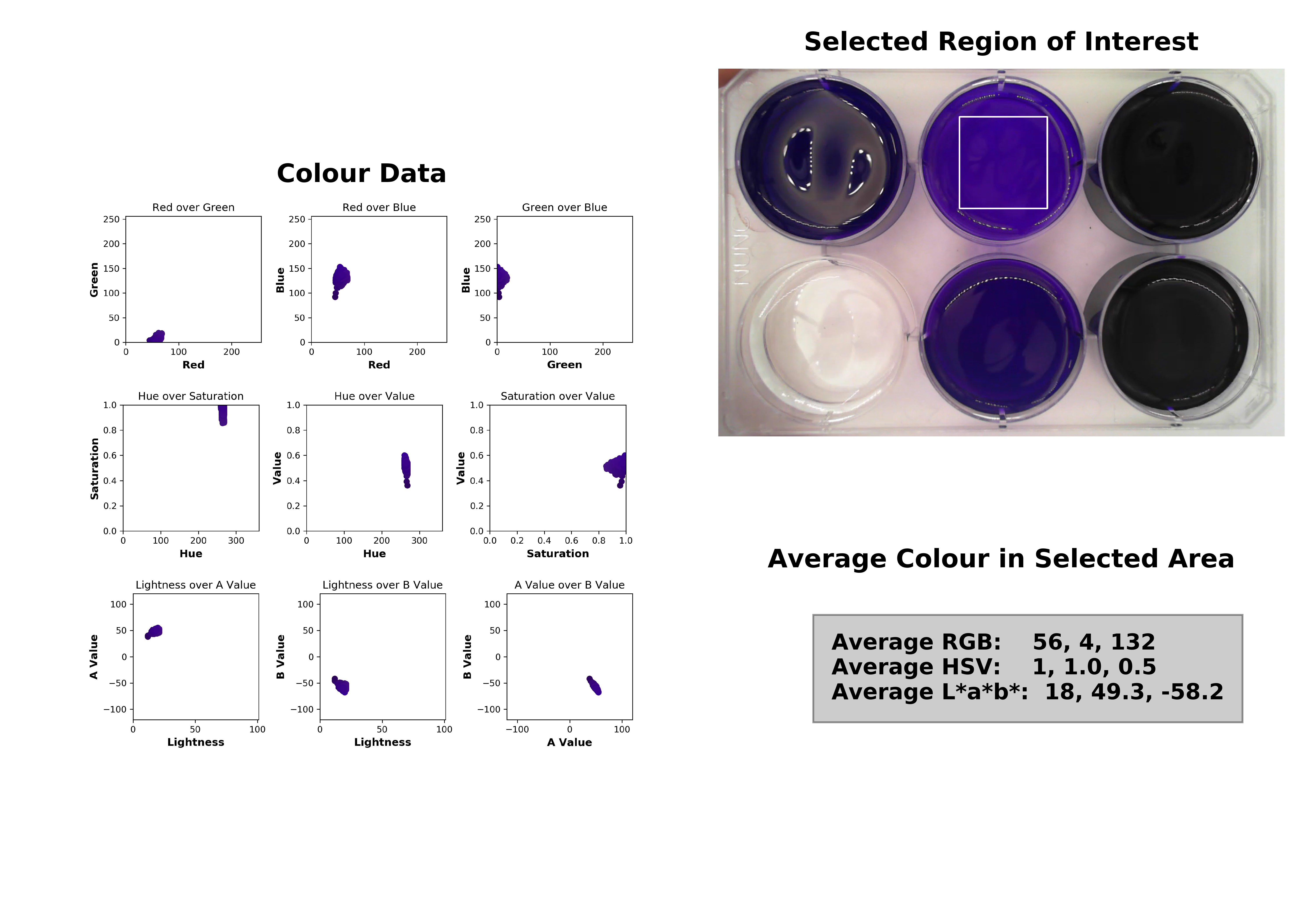

Supplement: Supplementary file 2 — Supporting Information [file ANIE-64-e202413395-s002.zip › Supporting Info - Machine readable data part 1/Figure 4 - glare analysis/6_above_SIanal__3/TILE_WITH_ROI.PNG]

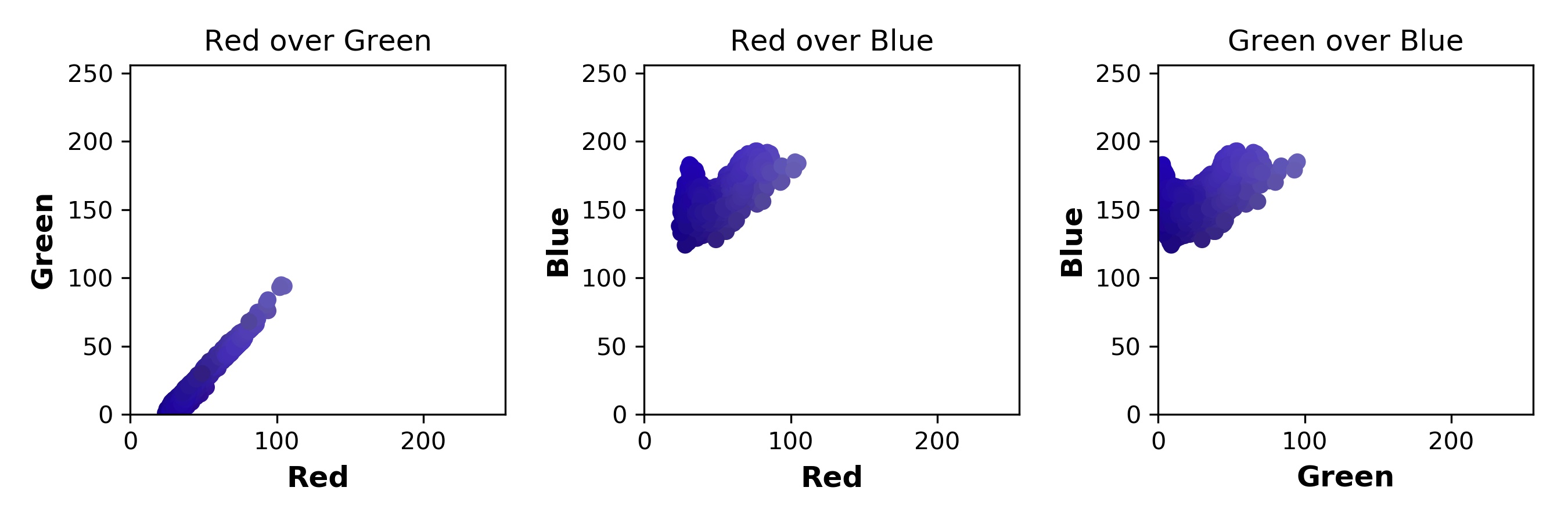

Supplement: Supplementary file 2 — Supporting Information [file ANIE-64-e202413395-s002.zip › Supporting Info - Machine readable data part 1/Figure 4 - glare analysis/24_above_SIanal__2/rgb.png]

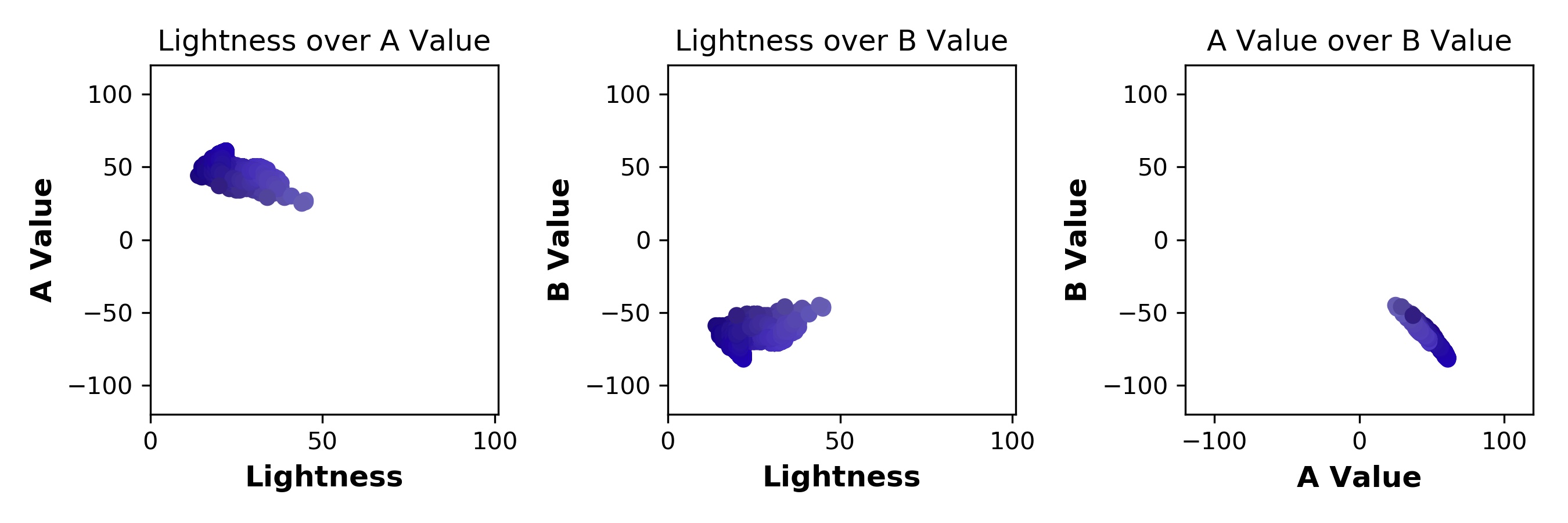

Supplement: Supplementary file 2 — Supporting Information [file ANIE-64-e202413395-s002.zip › Supporting Info - Machine readable data part 1/Figure 4 - glare analysis/24_above_SIanal__2/lab.png]

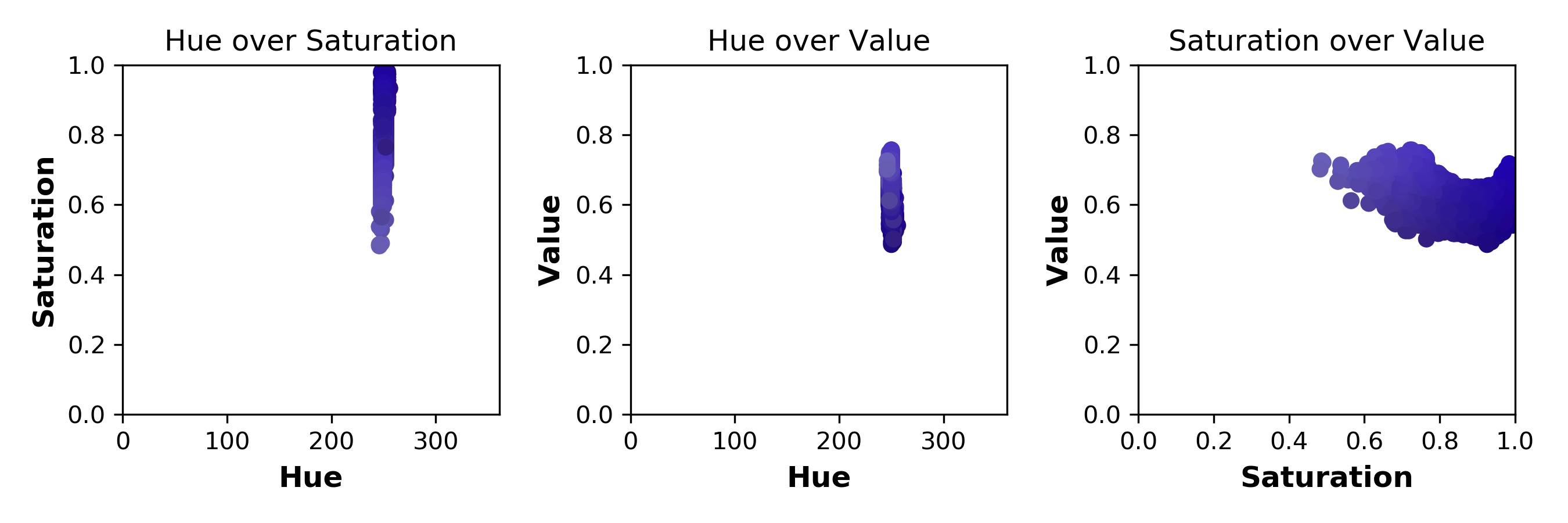

Supplement: Supplementary file 2 — Supporting Information [file ANIE-64-e202413395-s002.zip › Supporting Info - Machine readable data part 1/Figure 4 - glare analysis/24_above_SIanal__2/hsv.png]

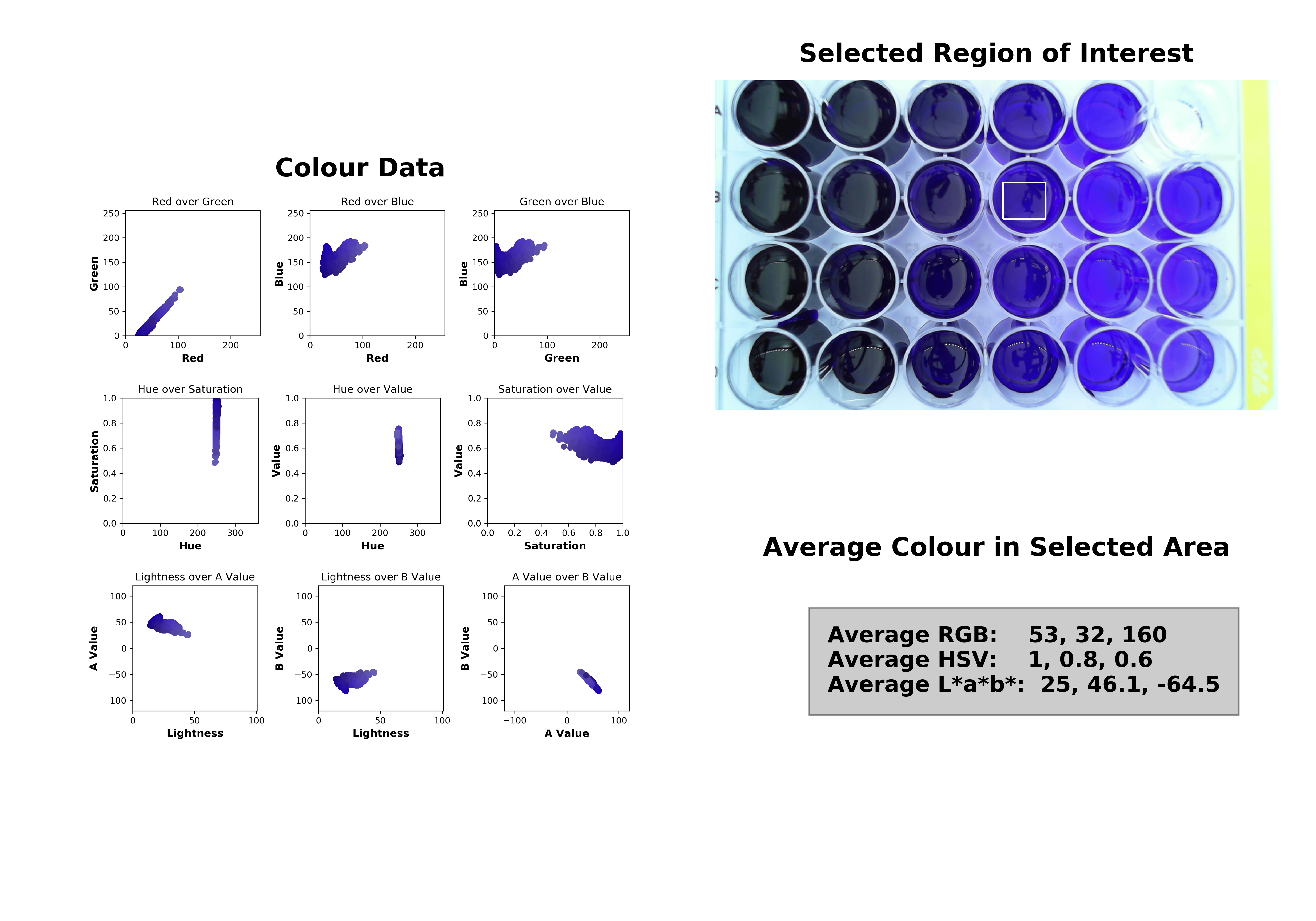

Supplement: Supplementary file 2 — Supporting Information [file ANIE-64-e202413395-s002.zip › Supporting Info - Machine readable data part 1/Figure 4 - glare analysis/24_above_SIanal__2/TILE_WITH_ROI.PNG]

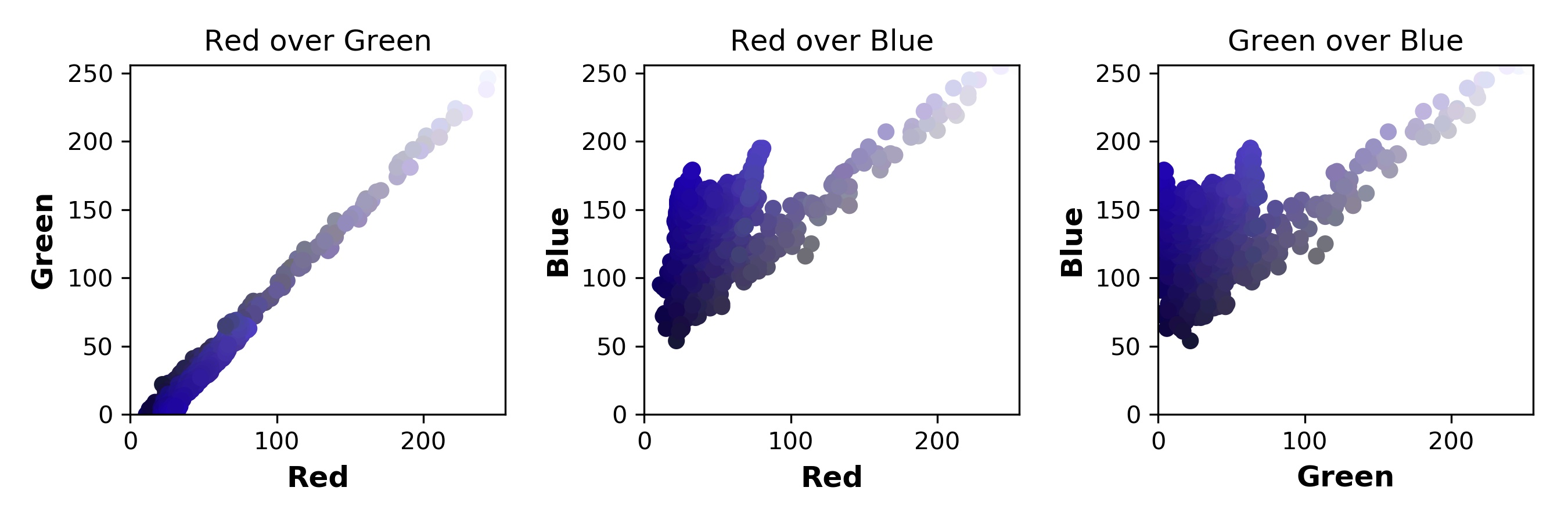

Supplement: Supplementary file 2 — Supporting Information [file ANIE-64-e202413395-s002.zip › Supporting Info - Machine readable data part 1/Figure 4 - glare analysis/24_above_SIanal__5/rgb.png]

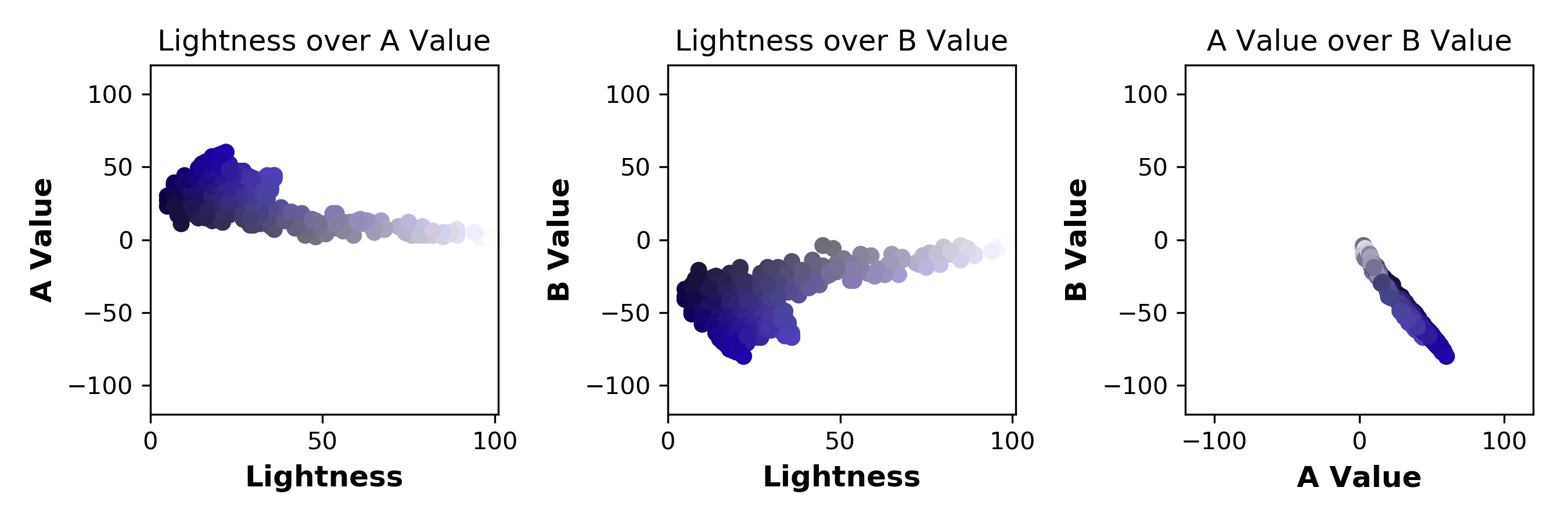

Supplement: Supplementary file 2 — Supporting Information [file ANIE-64-e202413395-s002.zip › Supporting Info - Machine readable data part 1/Figure 4 - glare analysis/24_above_SIanal__5/lab.png]

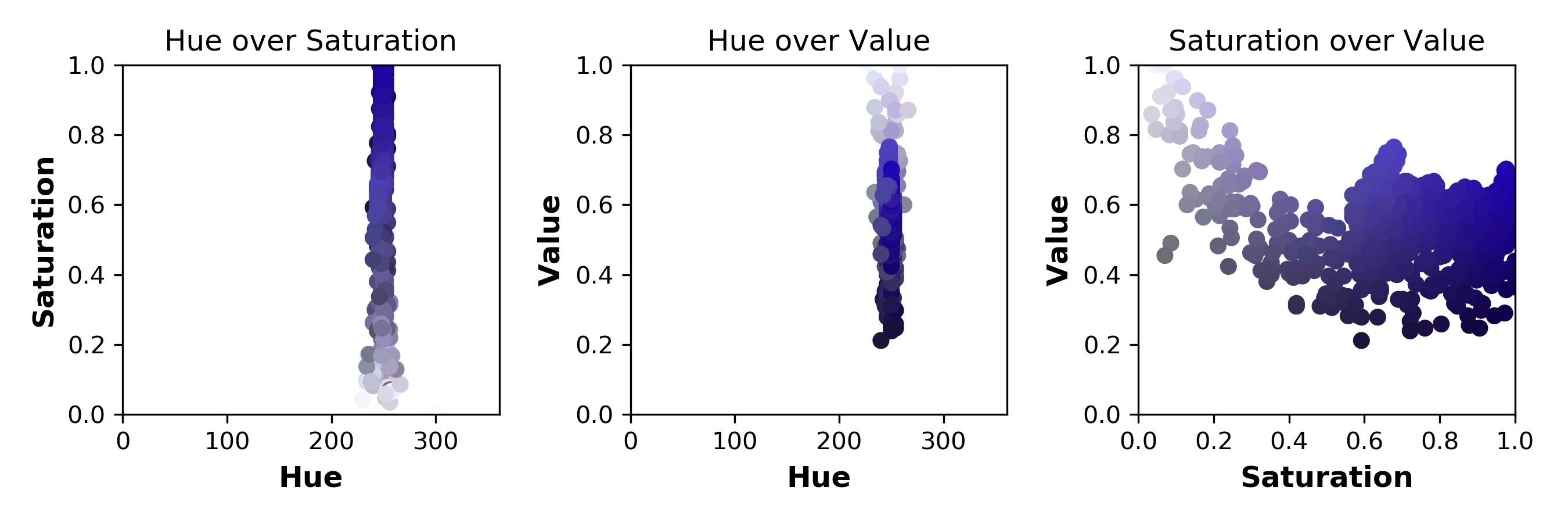

Supplement: Supplementary file 2 — Supporting Information [file ANIE-64-e202413395-s002.zip › Supporting Info - Machine readable data part 1/Figure 4 - glare analysis/24_above_SIanal__5/hsv.png]

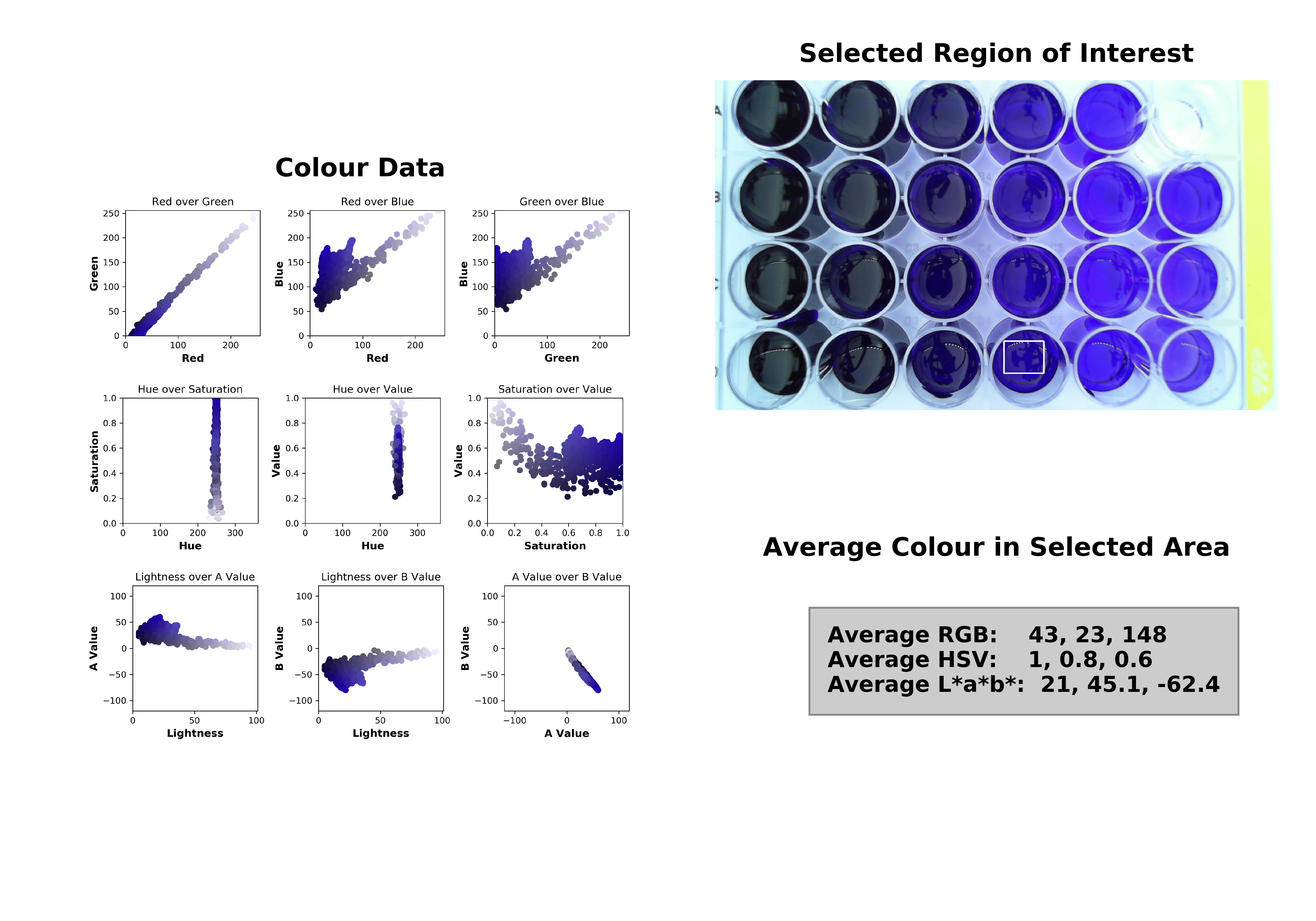

Supplement: Supplementary file 2 — Supporting Information [file ANIE-64-e202413395-s002.zip › Supporting Info - Machine readable data part 1/Figure 4 - glare analysis/24_above_SIanal__5/TILE_WITH_ROI.PNG]

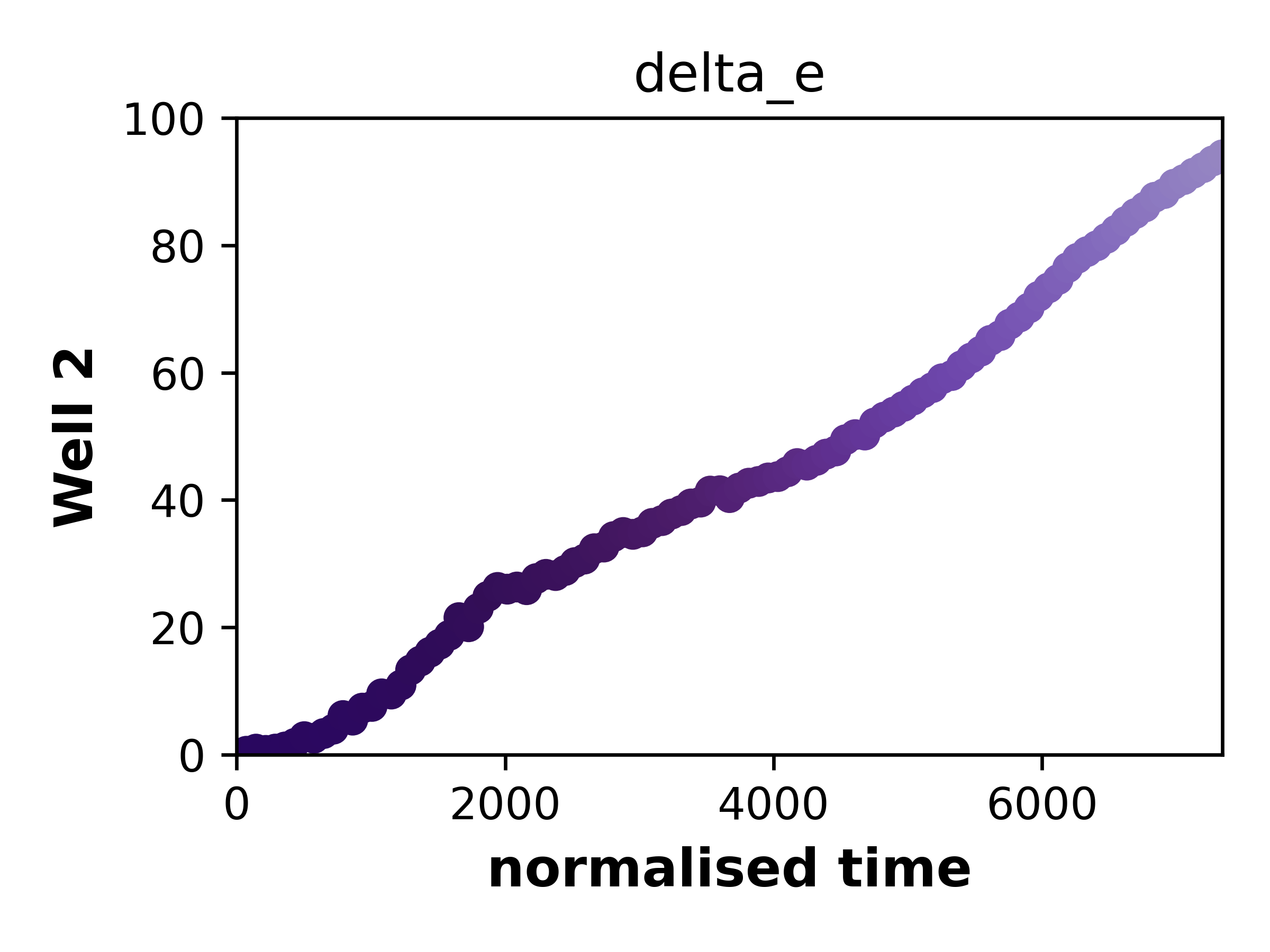

Supplement: Supplementary file 2 — Supporting Information [file ANIE-64-e202413395-s002.zip › Supporting Info - Machine readable data part 1/Figure 9 - crystal violet mixing analysis/Kineticolor outputs/temp/delta_e over normalised time - Well 2.png]

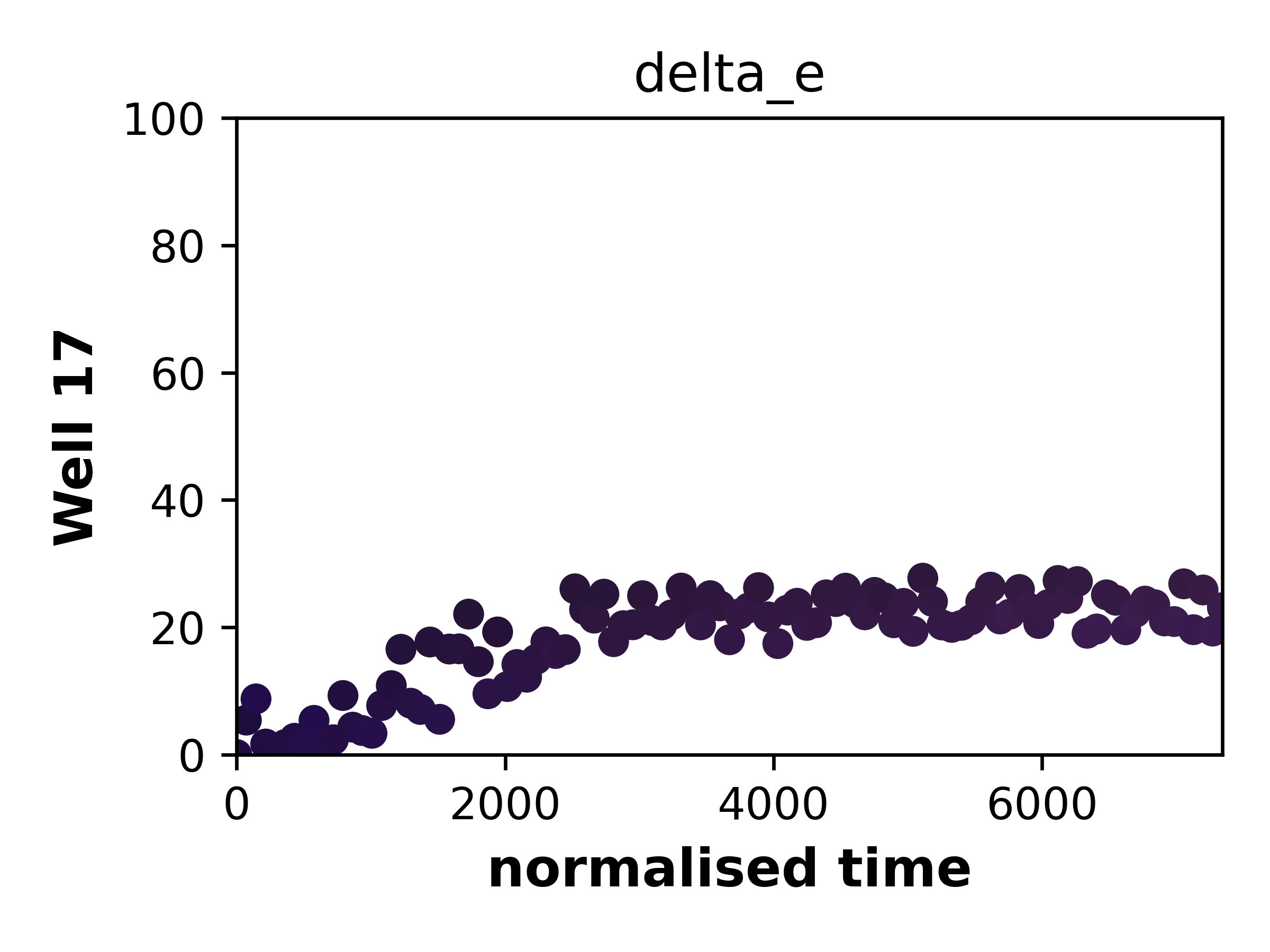

Supplement: Supplementary file 2 — Supporting Information [file ANIE-64-e202413395-s002.zip › Supporting Info - Machine readable data part 1/Figure 9 - crystal violet mixing analysis/Kineticolor outputs/temp/delta_e over normalised time - Well 17.png]

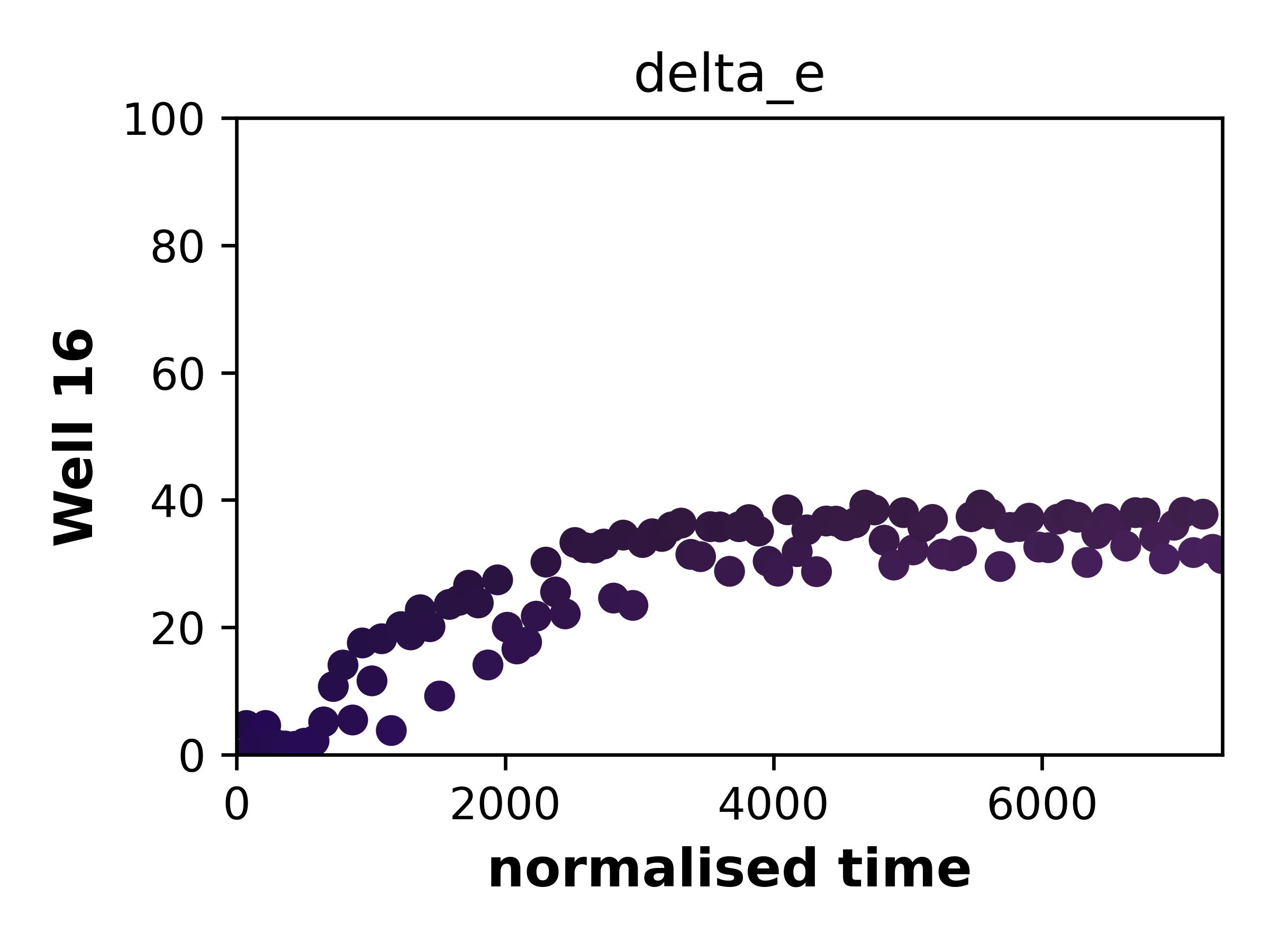

Supplement: Supplementary file 2 — Supporting Information [file ANIE-64-e202413395-s002.zip › Supporting Info - Machine readable data part 1/Figure 9 - crystal violet mixing analysis/Kineticolor outputs/temp/delta_e over normalised time - Well 16.png]

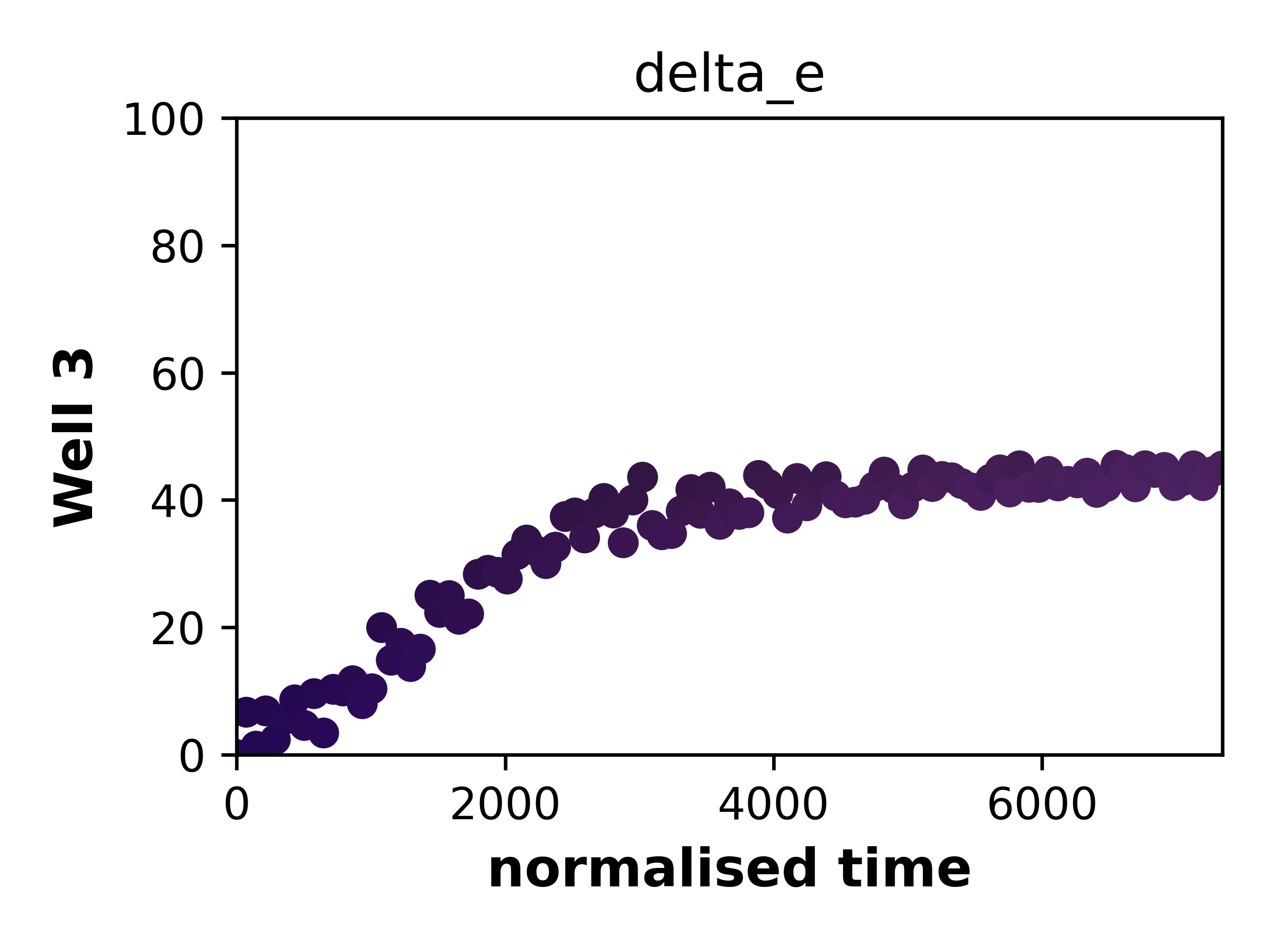

Supplement: Supplementary file 2 — Supporting Information [file ANIE-64-e202413395-s002.zip › Supporting Info - Machine readable data part 1/Figure 9 - crystal violet mixing analysis/Kineticolor outputs/temp/delta_e over normalised time - Well 3.png]

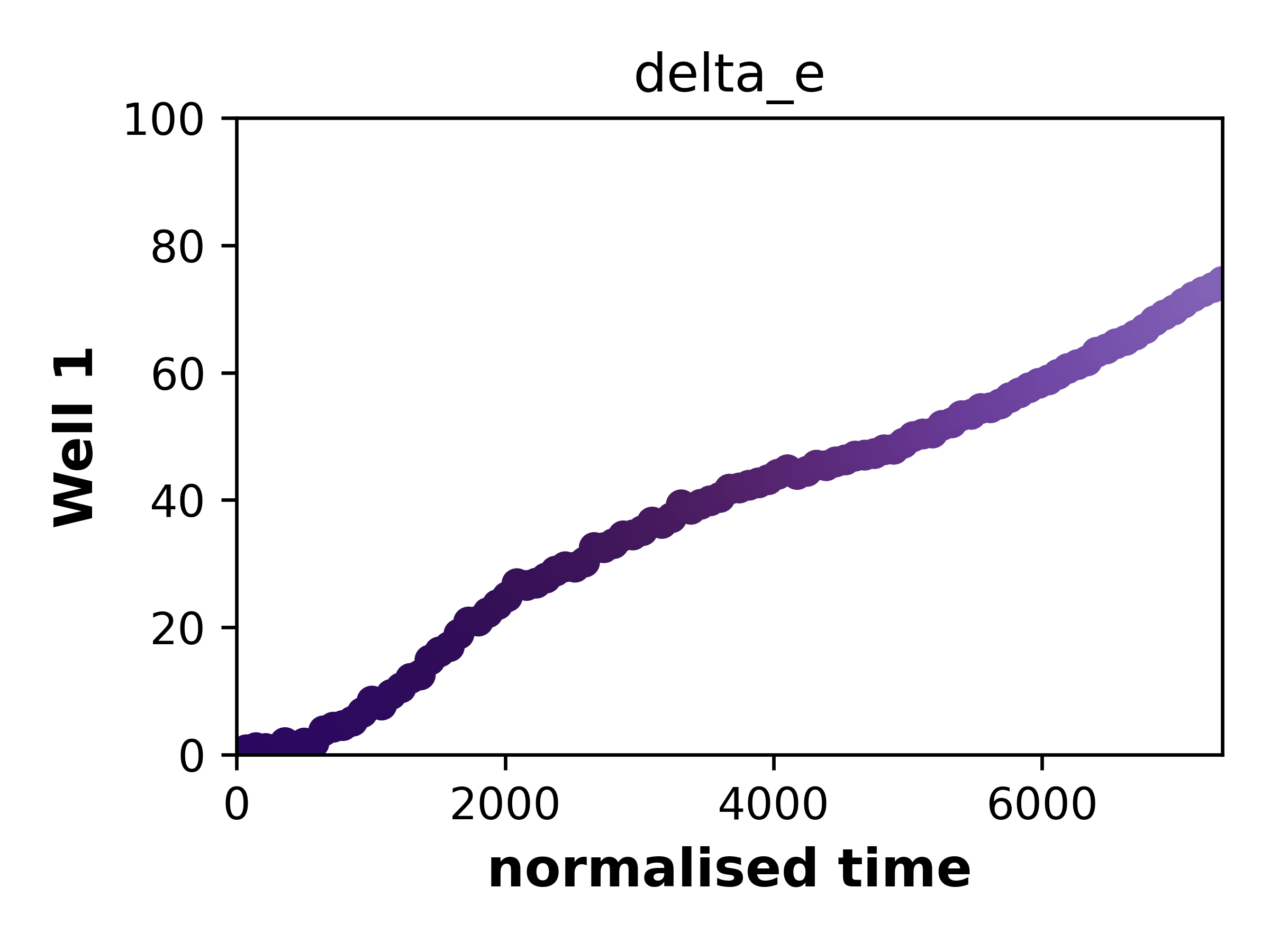

Supplement: Supplementary file 2 — Supporting Information [file ANIE-64-e202413395-s002.zip › Supporting Info - Machine readable data part 1/Figure 9 - crystal violet mixing analysis/Kineticolor outputs/temp/delta_e over normalised time - Well 1.png]

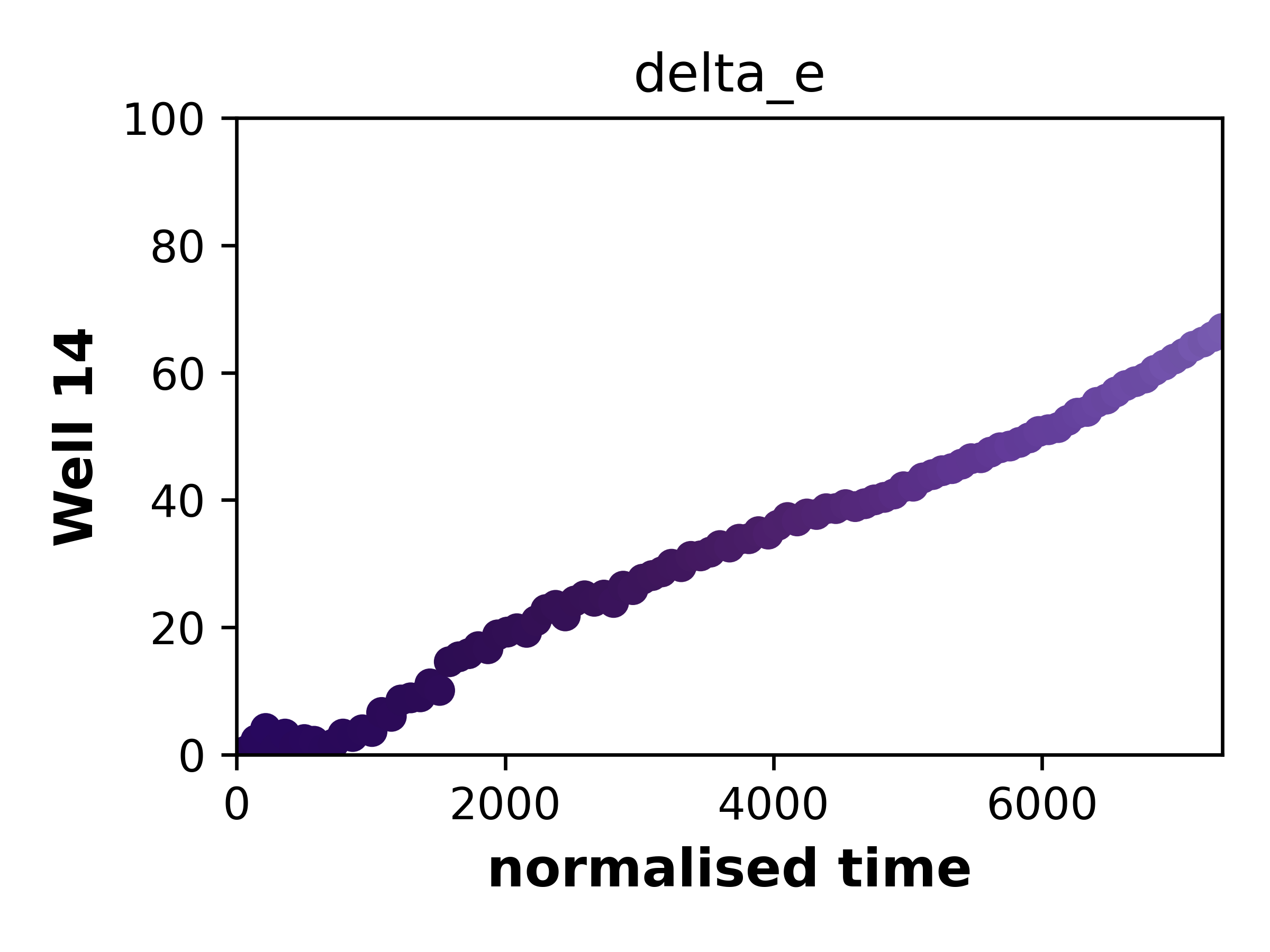

Supplement: Supplementary file 2 — Supporting Information [file ANIE-64-e202413395-s002.zip › Supporting Info - Machine readable data part 1/Figure 9 - crystal violet mixing analysis/Kineticolor outputs/temp/delta_e over normalised time - Well 14.png]

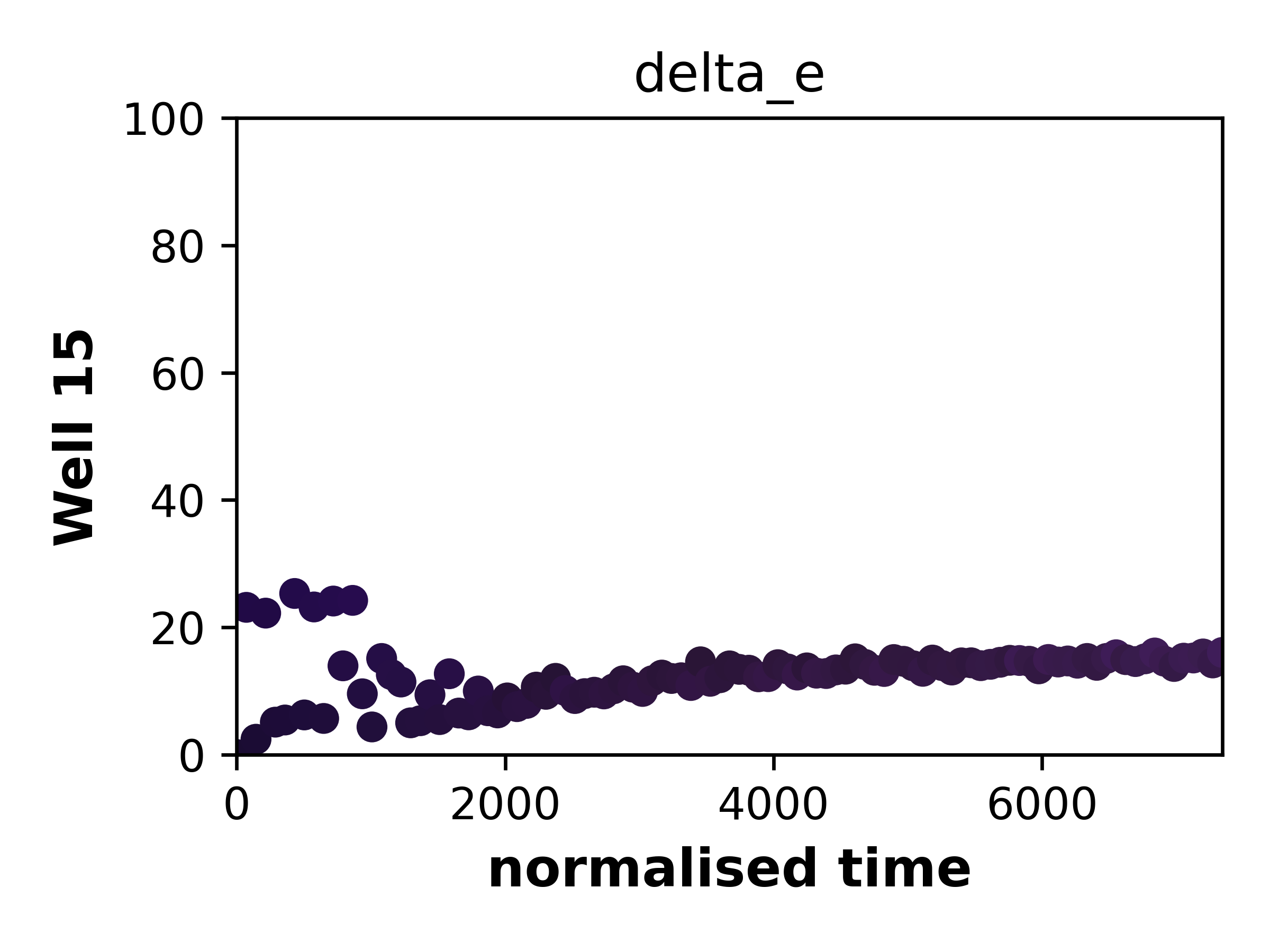

Supplement: Supplementary file 2 — Supporting Information [file ANIE-64-e202413395-s002.zip › Supporting Info - Machine readable data part 1/Figure 9 - crystal violet mixing analysis/Kineticolor outputs/temp/delta_e over normalised time - Well 15.png]

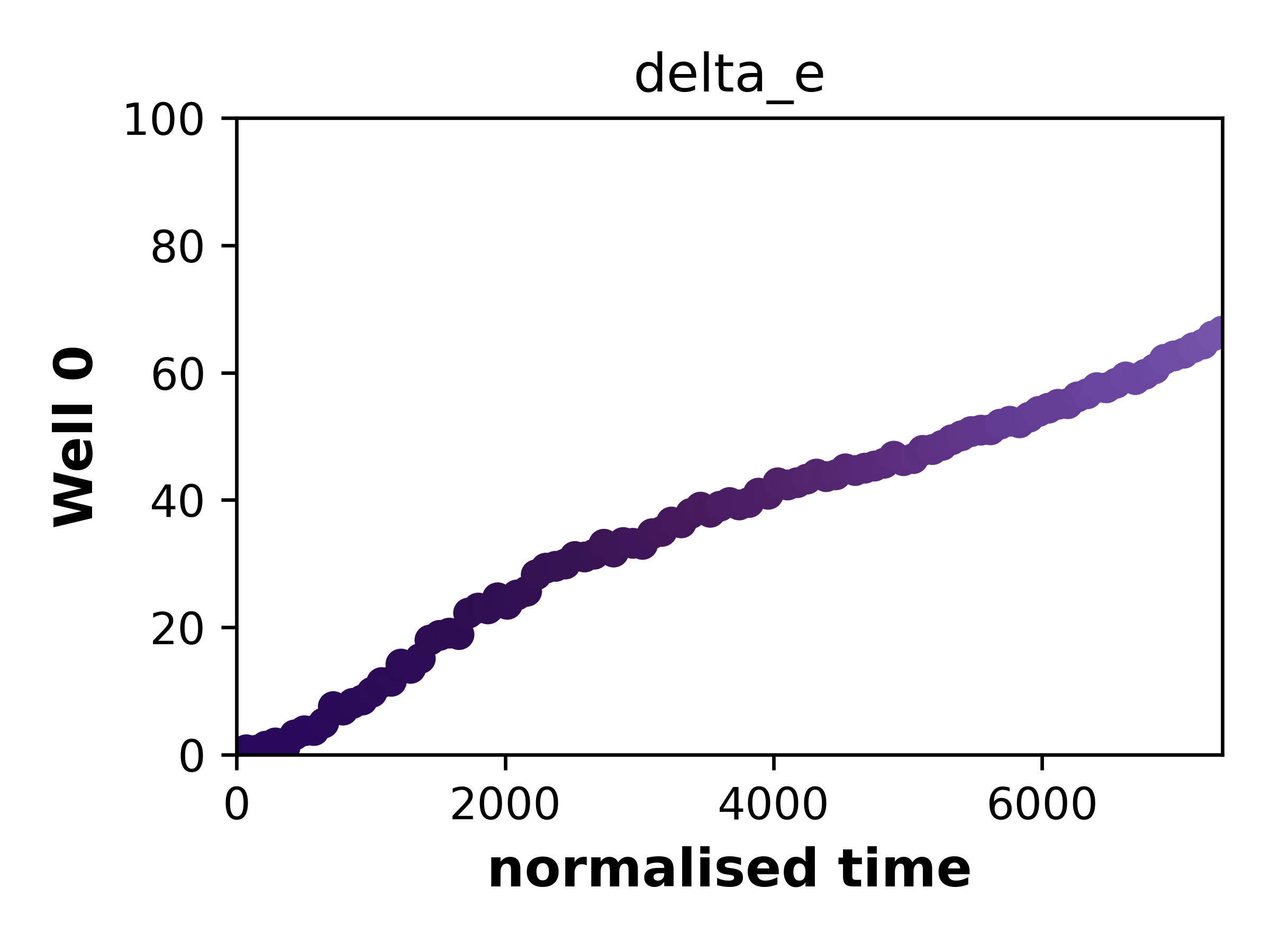

Supplement: Supplementary file 2 — Supporting Information [file ANIE-64-e202413395-s002.zip › Supporting Info - Machine readable data part 1/Figure 9 - crystal violet mixing analysis/Kineticolor outputs/temp/delta_e over normalised time - Well 0.png]
