# Supplementary material for: Parallel and High Throughput Reaction Monitoring with Computer Vision
Source: Angew Chem Int Ed Engl. 2024 Oct 31;64(1):e202413395. doi: 10.1002/anie.202413395 (PMC11701362; doi:10.1002/anie.202413395)
Supplement: Supplementary file 3 — Supporting Information [file ANIE-64-e202413395-s003.zip › Supporting Info - Machine readable data part 2/Table 2 - esterification in HTE/HPLC data.pdf]

**Sample name:**	Blank		
**Data file:**	2024-07-03 12-20-19+01-00-01.dx	**Operator:**	SYSTEM
**Instrument:**	1220 Infinity II HPLC	**Injection date:**	2024-07-03 12:21:17+01:00
**Inj. volume:**	5.000 µL	**Location:**	41
**Acq. method:**	Barry's standard method_low flow_higher A.amx	**Type:**	Sample
**Processing method:**	HB Standard method.pmx		
**Manually modified:**	None		

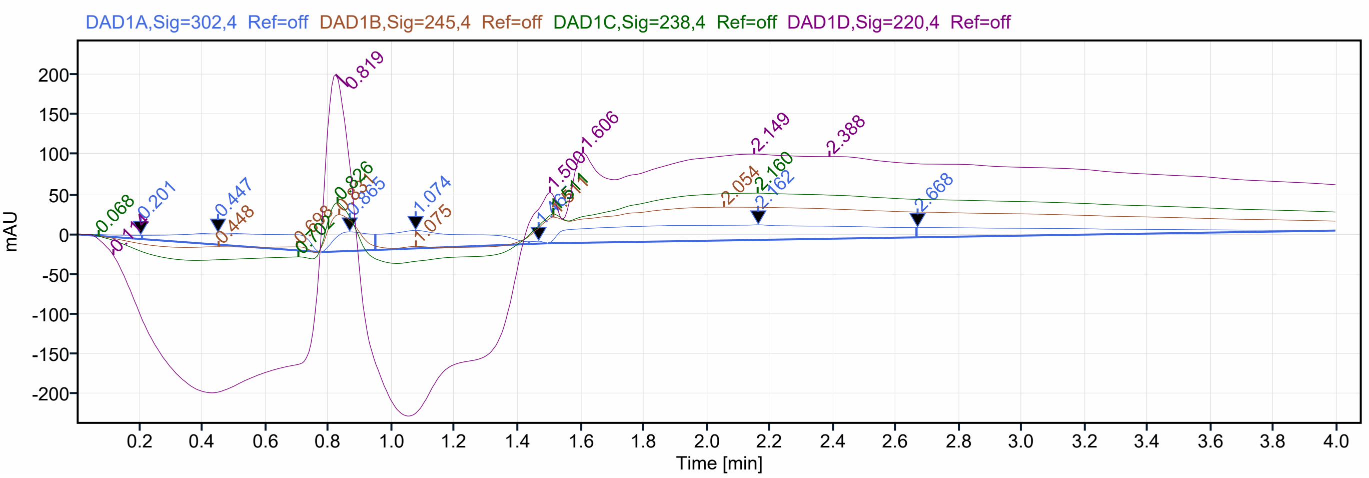

Sample Name	Name	RT (mins)	Area	Concentration (mg/L)
Blank	DMAP			
Blank	ISTD (Acetophenone)			
Blank	Pivalic Anhydride			
Blank	Product Ester			
Blank	Umbelliferone			

Sample name: Vial 1

Data file: 2024-07-03 12-25-20+01-00-02.dx

Operator: SYSTEM

Instrument: 1220 Infinity II HPLC

Injection date: 2024-07-03 12:26:13+01:00

Inj. volume: 5.000 µL

Location: 1

Acq. method: Barry's standard method_low flow_higher A.amx

Type: Sample

Processing method: HB Standard method.pmx

Manually modified: None

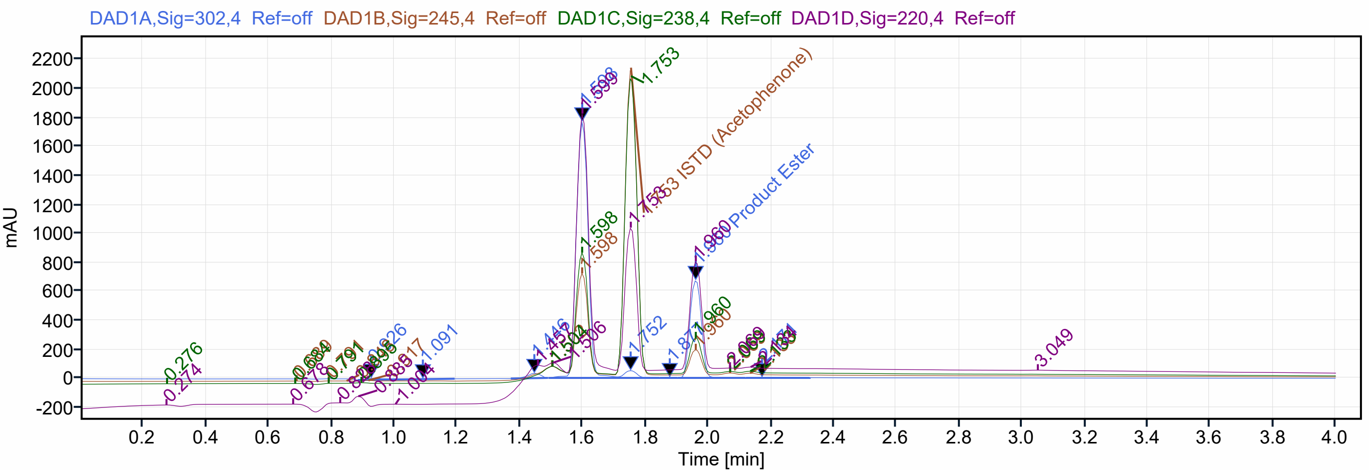

Sample Name	Name	RT (mins)	Area	Concentration (mg/L)
Vial 1	Umbelliferone			
Vial 1	Pivalic Anhydride			
Vial 1	DMAP			
Vial 1	ISTD (Acetophenone)	1.753	5416.5199	
Vial 1	Product Ester	1.960	1725.5113	

Injection Report - By Sample

Kineticolor

**Sample name:**	Vial 2
**Data file:**	2024-07-03 12-30-17+01-00-03.dx
**Operator:**	SYSTEM
**Instrument:**	1220 Infinity II HPLC
**Injection date:**	2024-07-03 12:31:09+01:00
**Inj. volume:**	5.000 µL
**Location:**	2
**Acq. method:**	Barry's standard method_low flow_higher A.amx
**Type:**	Sample
**Processing method:**	HB Standard method.pmx
**Manually modified:**	None

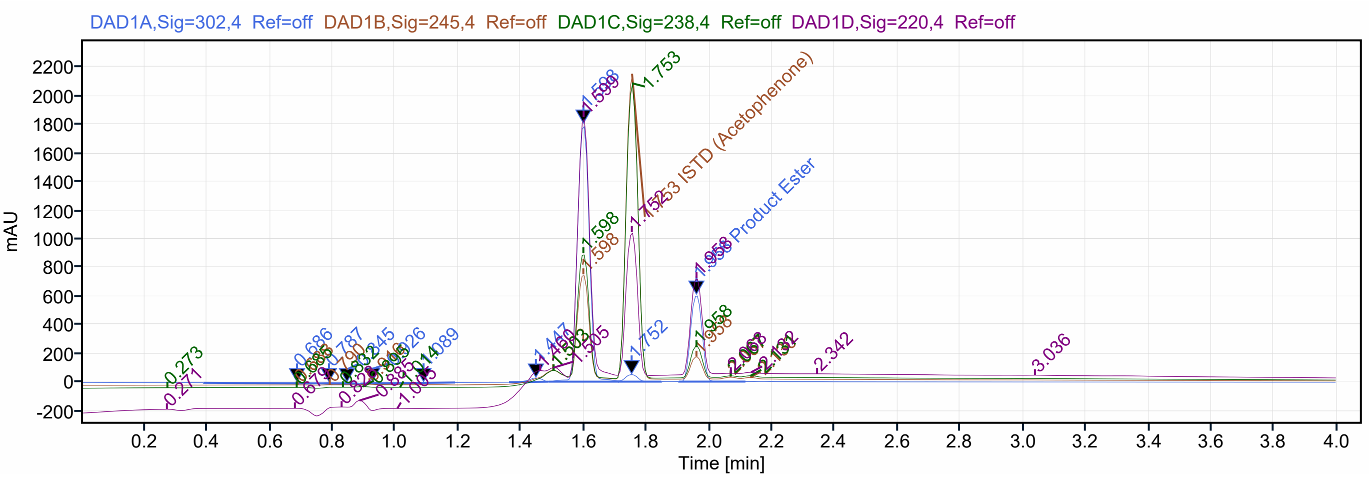

Sample Name	Name	RT (mins)	Area	Concentration (mg/L)
Vial 2	Umbelliferone			
Vial 2	Pivalic Anhydride			
Vial 2	DMAP			
Vial 2	ISTD (Acetophenone)	1.753	5445.5842	
Vial 2	Product Ester	1.958	1526.8603	

Injection Report - By Sample

Kineticolor

Sample name: Vial 3

Data file: 2024-07-03 12-35-13+01-00-04.dx

Operator: SYSTEM

Instrument: 1220 Infinity II HPLC

Injection date: 2024-07-03 12:36:08+01:00

Inj. volume: 5.000 µL

Location: 3

Acq. method: Barry's standard method_low flow_higher A.amx

Type: Sample

Processing method: HB Standard method.pmx

Manually modified: None

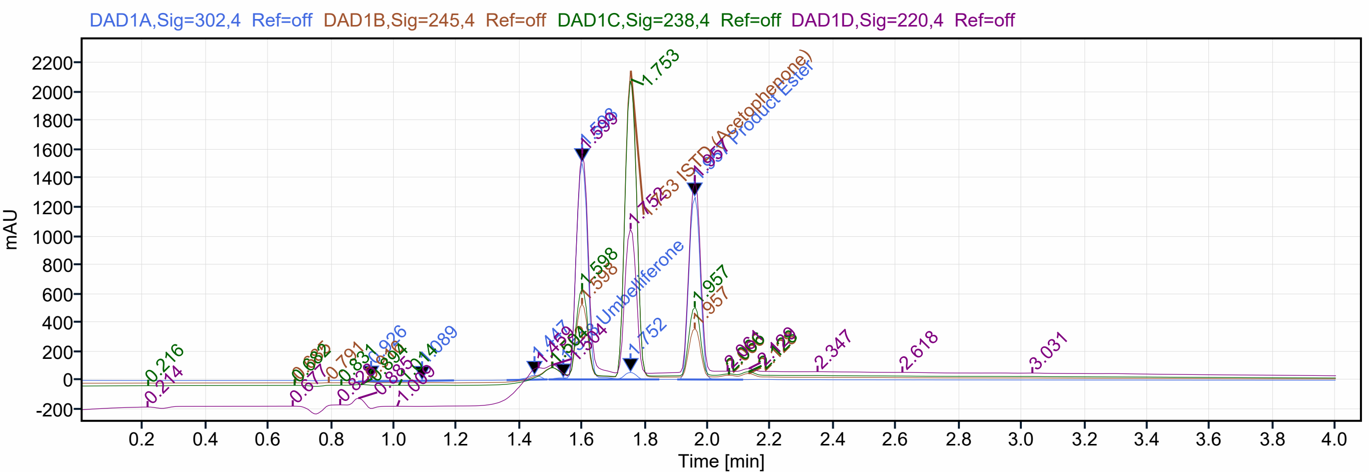

Sample Name	Name	RT (mins)	Area	Concentration (mg/L)
Vial 3	Pivalic Anhydride			
Vial 3	DMAP			
Vial 3	Umbelliferone	1.538	20.9418	
Vial 3	ISTD (Acetophenone)	1.753	5430.7351	
Vial 3	Product Ester	1.957	3197.1756	

Injection Report - By Sample

Kineticolor

Sample name: Vial 4

Data file: 2024-07-03 12-40-13+01-00-05.dx

Operator: SYSTEM

Instrument: 1220 Infinity II HPLC

Injection date: 2024-07-03 12:41:06+01:00

Inj. volume: 5.000 µL

Location: 4

Acq. method: Barry's standard method_low flow_higher A.amx

Type: Sample

Processing method: HB Standard method.pmx

Manually modified: None

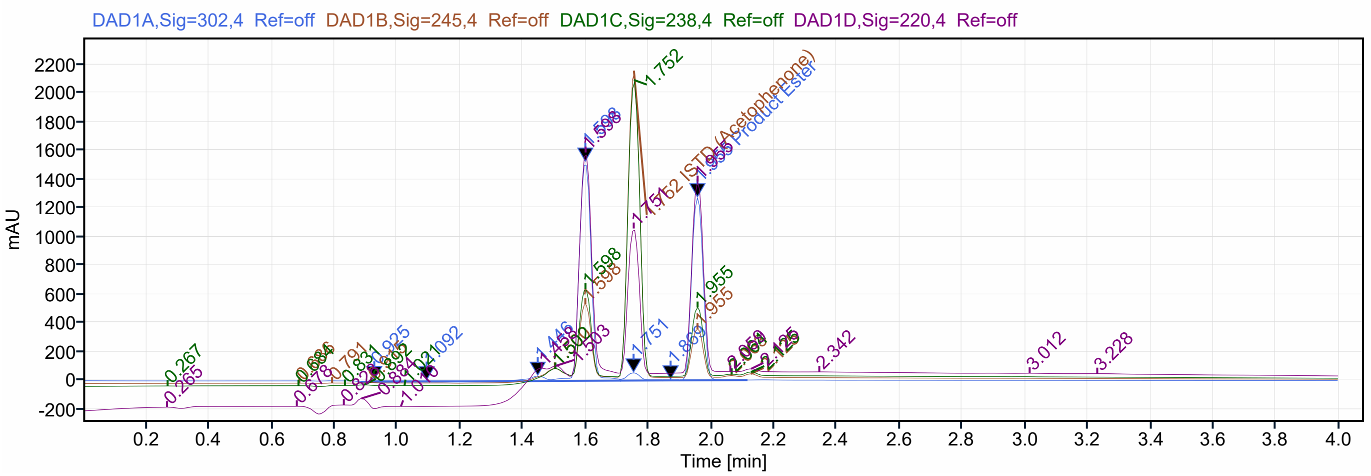

Sample Name	Name	RT (mins)	Area	Concentration (mg/L)
Vial 4	Umbelliferone			
Vial 4	Pivalic Anhydride			
Vial 4	DMAP			
Vial 4	ISTD (Acetophenone)	1.752	5447.8199	
Vial 4	Product Ester	1.955	3218.6657	

**Sample name:**	Vial 5		
**Data file:**	2024-07-03 12-45-09+01-00-06.dx	**Operator:**	SYSTEM
**Instrument:**	1220 Infinity II HPLC	**Injection date:**	2024-07-03 12:46:02+01:00
**Inj. volume:**	5.000 µL	**Location:**	5
**Acq. method:**	Barry's standard method_low flow_higher A.amx	**Type:**	Sample
**Processing method:**	HB Standard method.pmx		
**Manually modified:**	None		

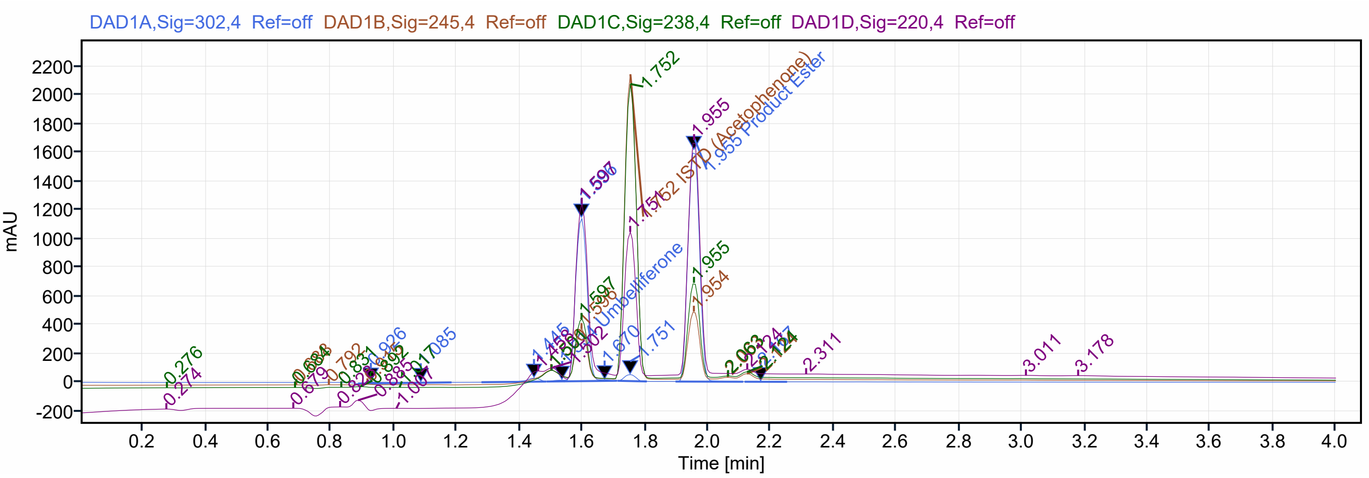

Sample Name	Name	RT (mins)	Area	Concentration (mg/L)
Vial 5	Pivalic Anhydride			
Vial 5	DMAP			
Vial 5	Umbelliferone	1.534	19.4986	
Vial 5	ISTD (Acetophenone)	1.752	5420.0326	
Vial 5	Product Ester	1.955	4085.0789	

Injection Report - By Sample

Kineticolor

**Sample name:**	Vial 6		
**Data file:**	2024-07-03 12-50-06+01-00-07.dx	**Operator:**	SYSTEM
**Instrument:**	1220 Infinity II HPLC	**Injection date:**	2024-07-03 12:50:58+01:00
**Inj. volume:**	5.000 µL	**Location:**	6
**Acq. method:**	Barry's standard method_low flow_higher A.amx	**Type:**	Sample
**Processing method:**	HB Standard method.pmx		
**Manually modified:**	None		

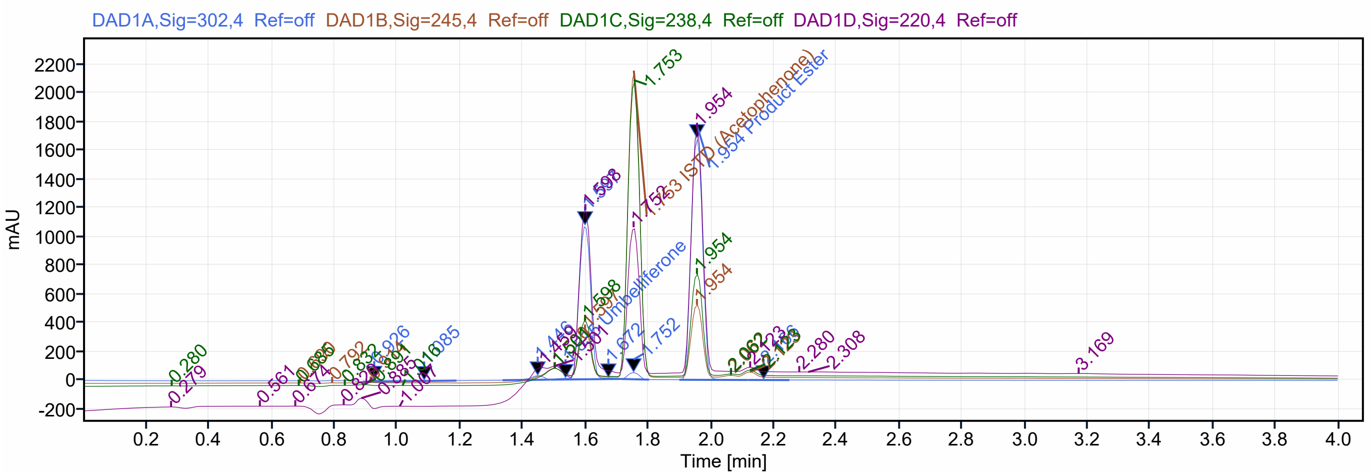

Sample Name	Name	RT (mins)	Area	Concentration (mg/L)
Vial 6	Pivalic Anhydride			
Vial 6	DMAP			
Vial 6	Umbelliferone	1.535	17.8267	
Vial 6	ISTD (Acetophenone)	1.753	5436.5533	
Vial 6	Product Ester	1.954	4247.0624	

**Sample name:**	Vial 7		
**Data file:**	2024-07-03 12-55-01+01-00-08.dx	**Operator:**	SYSTEM
**Instrument:**	1220 Infinity II HPLC	**Injection date:**	2024-07-03 12:55:58+01:00
**Inj. volume:**	5.000 µL	**Location:**	7
**Acq. method:**	Barry's standard method_low flow_higher A.amx	**Type:**	Sample
**Processing method:**	HB Standard method.pmx		
**Manually modified:**	None		

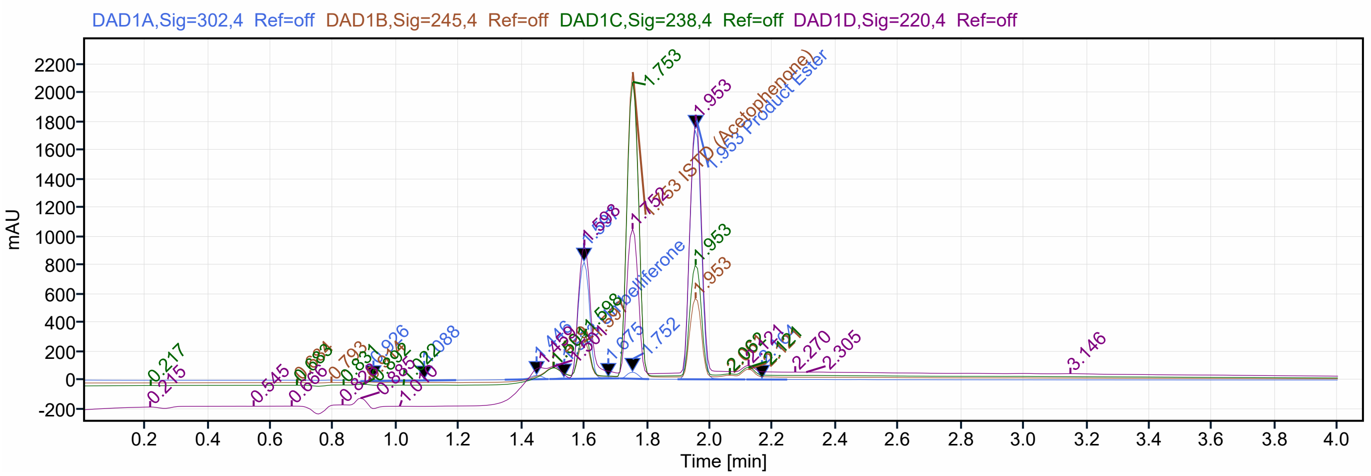

Sample Name	Name	RT (mins)	Area	Concentration (mg/L)
Vial 7	Pivalic Anhydride			
Vial 7	DMAP			
Vial 7	Umbelliferone	1.533	16.5017	
Vial 7	ISTD (Acetophenone)	1.753	5424.4573	
Vial 7	Product Ester	1.953	4435.3981	

Injection Report - By Sample

Kineticolor

Sample name: Vial 8

Data file: 2024-07-03 13-00-02+01-00-09.dx

Operator: SYSTEM

Instrument: 1220 Infinity II HPLC

Injection date: 2024-07-03 13:00:55+01:00

Inj. volume: 5.000 µL

Location: 8

Acq. method: Barry's standard method_low flow_higher A.amx

Type: Sample

Processing method: HB Standard method.pmx

Manually modified: None

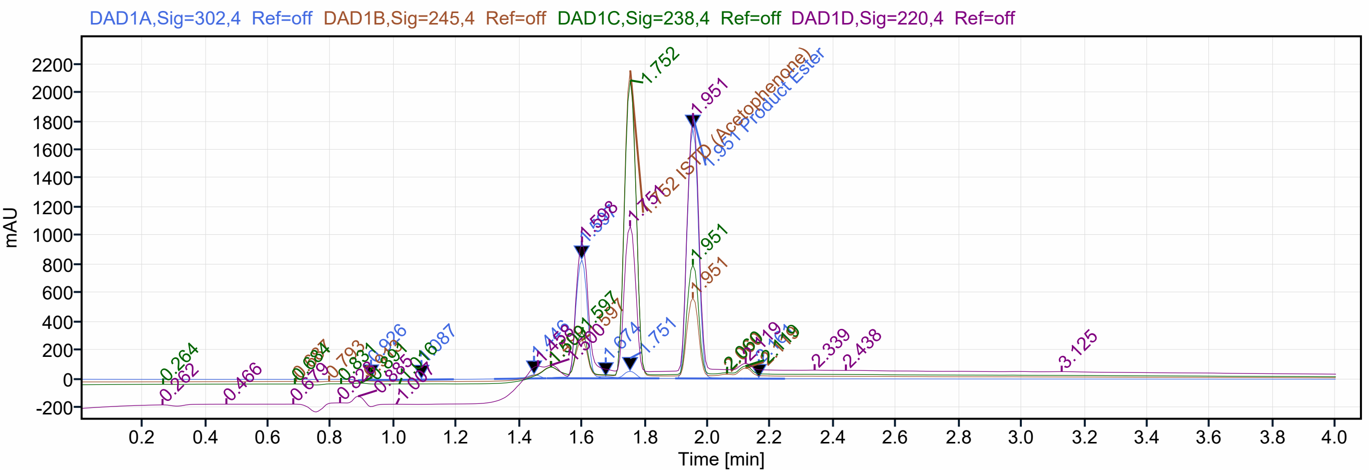

Sample Name	Name	RT (mins)	Area	Concentration (mg/L)
Vial 8	Umbelliferone			
Vial 8	Pivalic Anhydride			
Vial 8	DMAP			
Vial 8	ISTD (Acetophenone)	1.752	5429.9644	
Vial 8	Product Ester	1.951	4419.8946	

**Sample name:**	Blank		
**Data file:**	2024-07-03 13-05-00+01-00-10.dx	**Operator:**	SYSTEM
**Instrument:**	1220 Infinity II HPLC	**Injection date:**	2024-07-03 13:05:53+01:00
**Inj. volume:**	5.000 µL	**Location:**	41
**Acq. method:**	Barry's standard method_low flow_higher A.amx	**Type:**	Sample
**Processing method:**	HB Standard method.pmx		
**Manually modified:**	None		

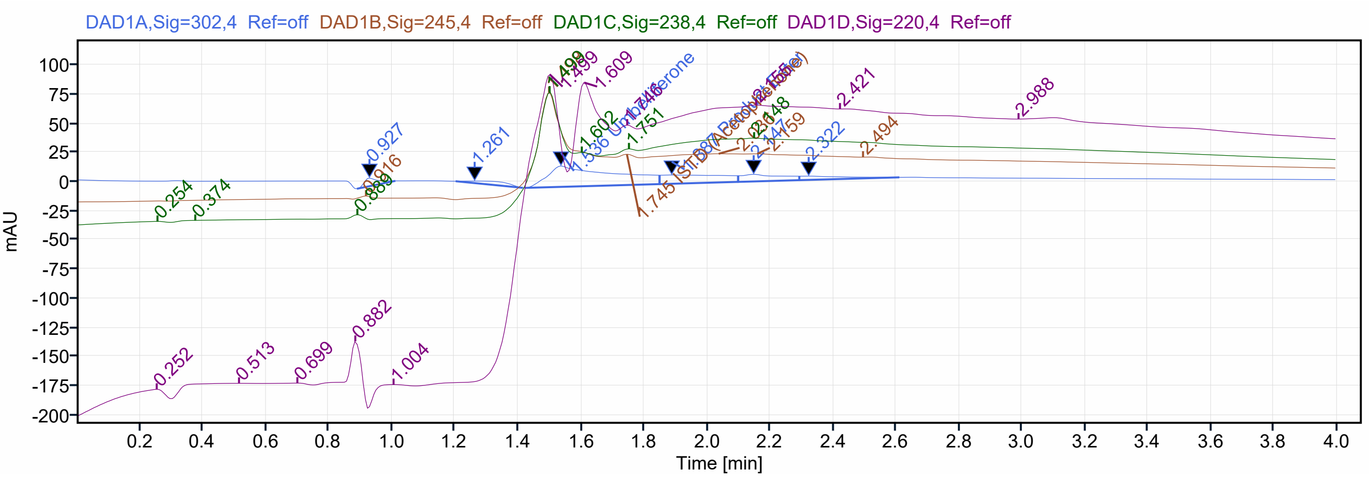

Sample Name	Name	RT (mins)	Area	Concentration (mg/L)
Blank	Pivalic Anhydride			
Blank	DMAP			
Blank	Umbelliferone	1.536	262.1306	
Blank	ISTD (Acetophenone)	1.745	161.5140	
Blank	Product Ester	1.887	98.7666	

Injection Report - By Sample

Kineticolor

**Sample name:**	Vial 9		
**Data file:**	2024-07-03 13-09-57+01-00-11.dx	**Operator:**	SYSTEM
**Instrument:**	1220 Infinity II HPLC	**Injection date:**	2024-07-03 13:10:52+01:00
**Inj. volume:**	5.000 µL	**Location:**	9
**Acq. method:**	Barry's standard method_low flow_higher A.amx	**Type:**	Sample
**Processing method:**	HB Standard method.pmx		
**Manually modified:**	None		

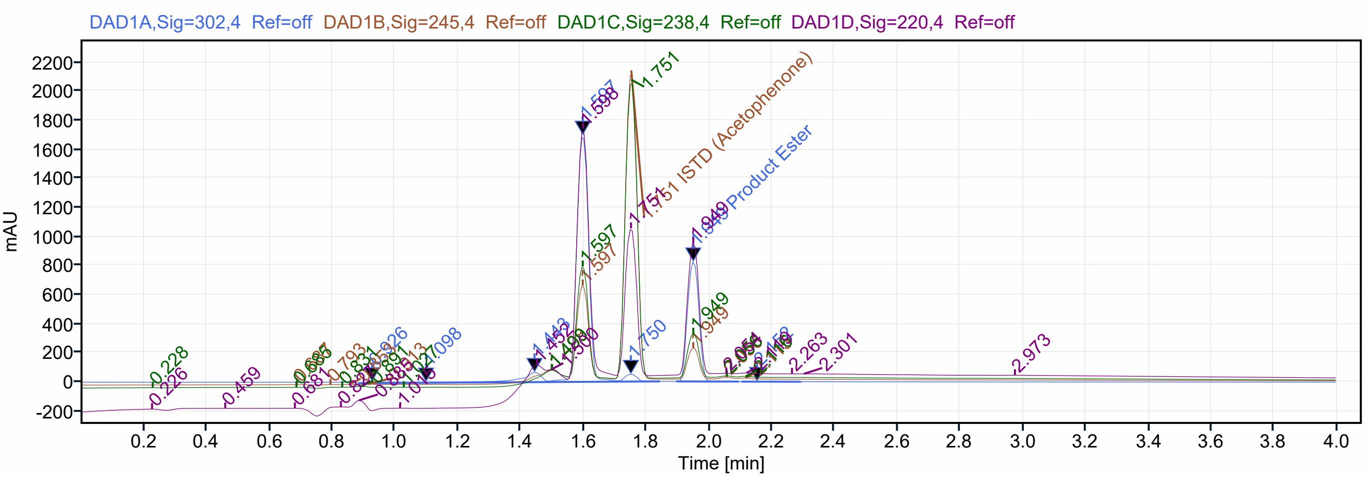

Sample Name	Name	RT (mins)	Area	Concentration (mg/L)
Vial 9	Umbelliferone			
Vial 9	Pivalic Anhydride			
Vial 9	DMAP			
Vial 9	ISTD (Acetophenone)	1.751	5409.6594	
Vial 9	Product Ester	1.949	2069.4781	

Injection Report - By Sample

Kineticolor

Sample name: Vial 10

Data file: 2024-07-03 13-14-57+01-00-12.dx

Operator: SYSTEM

Instrument: 1220 Infinity II HPLC

Injection date: 2024-07-03 13:15:54+01:00

Inj. volume: 5.000 µL

Location: 10

Acq. method: Barry's standard method_low flow_higher A.amx

Type: Sample

Processing method: HB Standard method.pmx

Manually modified: None

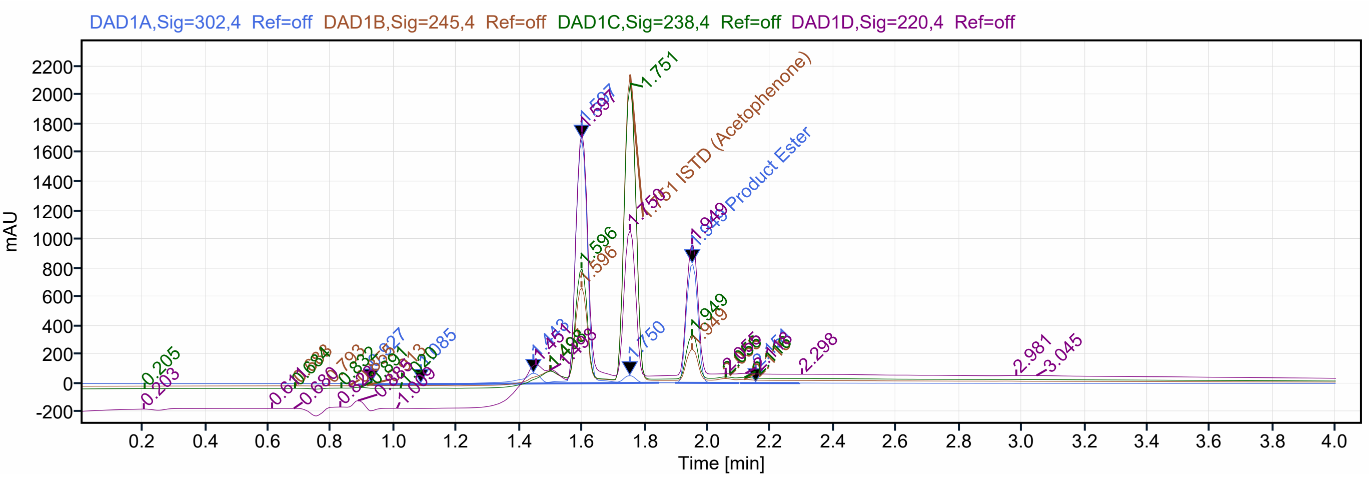

Sample Name	Name	RT (mins)	Area	Concentration (mg/L)
Vial 10	Umbelliferone			
Vial 10	Pivalic Anhydride			
Vial 10	DMAP			
Vial 10	ISTD (Acetophenone)	1.751	5393.3303	
Vial 10	Product Ester	1.949	2073.0260	

**Sample name:**	Vial 11
**Data file:**	2024-07-03 13-19-57+01-00-13.dx
**Operator:**	SYSTEM
**Instrument:**	1220 Infinity II HPLC
**Injection date:**	2024-07-03 13:20:53+01:00
**Inj. volume:**	5.000 µL
**Location:**	11
**Acq. method:**	Barry's standard method_low flow_higher A.amx
**Type:**	Sample
**Processing method:**	HB Standard method.pmx
**Manually modified:**	None

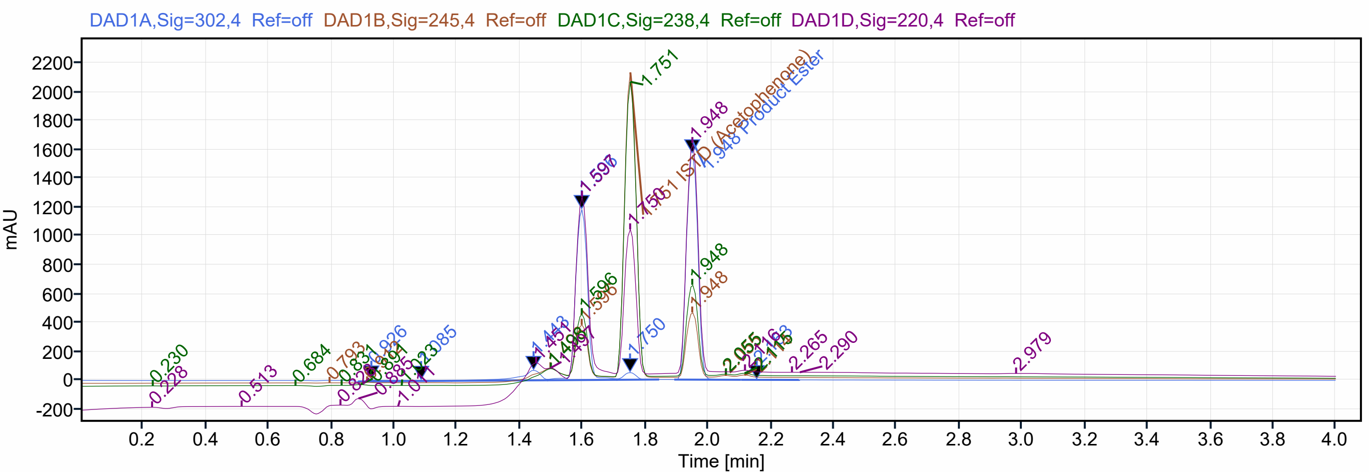

Sample Name	Name	RT (mins)	Area	Concentration (mg/L)
Vial 11	Umbelliferone			
Vial 11	Pivalic Anhydride			
Vial 11	DMAP			
Vial 11	ISTD (Acetophenone)	1.751	5375.1700	
Vial 11	Product Ester	1.948	3951.2280	

Injection Report - By Sample

Kineticolor

**Sample name:**	Vial 12		
**Data file:**	2024-07-03 13-24-57+01-00-14.dx	**Operator:**	SYSTEM
**Instrument:**	1220 Infinity II HPLC	**Injection date:**	2024-07-03 13:25:50+01:00
**Inj. volume:**	5.000 µL	**Location:**	12
**Acq. method:**	Barry's standard method_low flow_higher A.amx	**Type:**	Sample
**Processing method:**	HB Standard method.pmx		
**Manually modified:**	None		

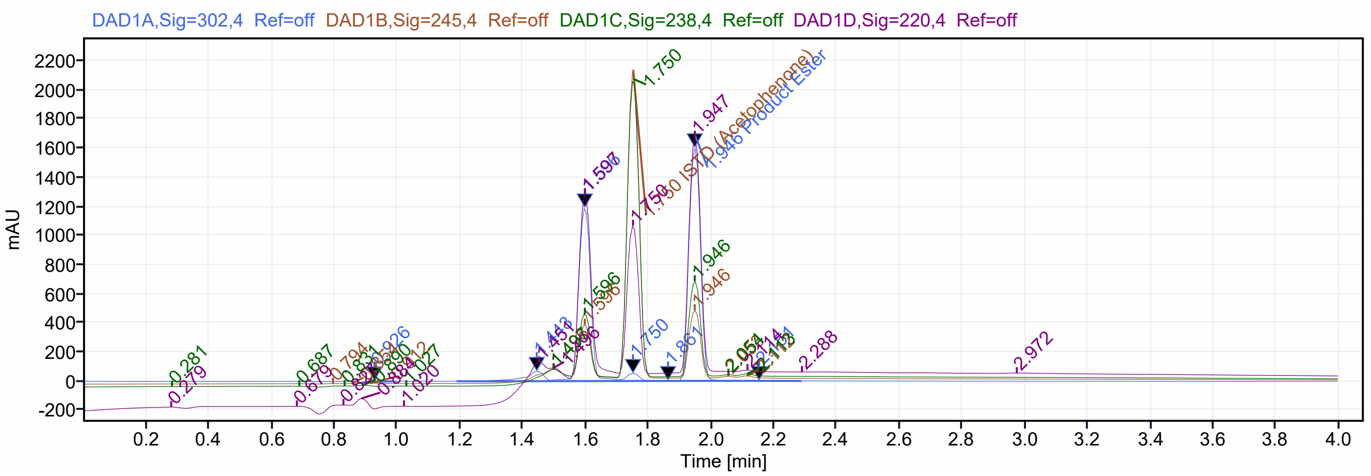

Sample Name	Name	RT (mins)	Area	Concentration (mg/L)
Vial 12	Umbelliferone			
Vial 12	Pivalic Anhydride			
Vial 12	DMAP			
Vial 12	ISTD (Acetophenone)	1.750	5386.9989	
Vial 12	Product Ester	1.946	4050.5163	

Injection Report - By Sample

Kineticolor

Sample name: Vial 13

Data file: 2024-07-03 13-29-54+01-00-15.dx

Operator: SYSTEM

Instrument: 1220 Infinity II HPLC

Injection date: 2024-07-03 13:30:45+01:00

Inj. volume: 5.000 µL

Location: 13

Acq. method: Barry's standard method_low flow_higher A.amx

Type: Sample

Processing method: HB Standard method.pmx

Manually modified: None

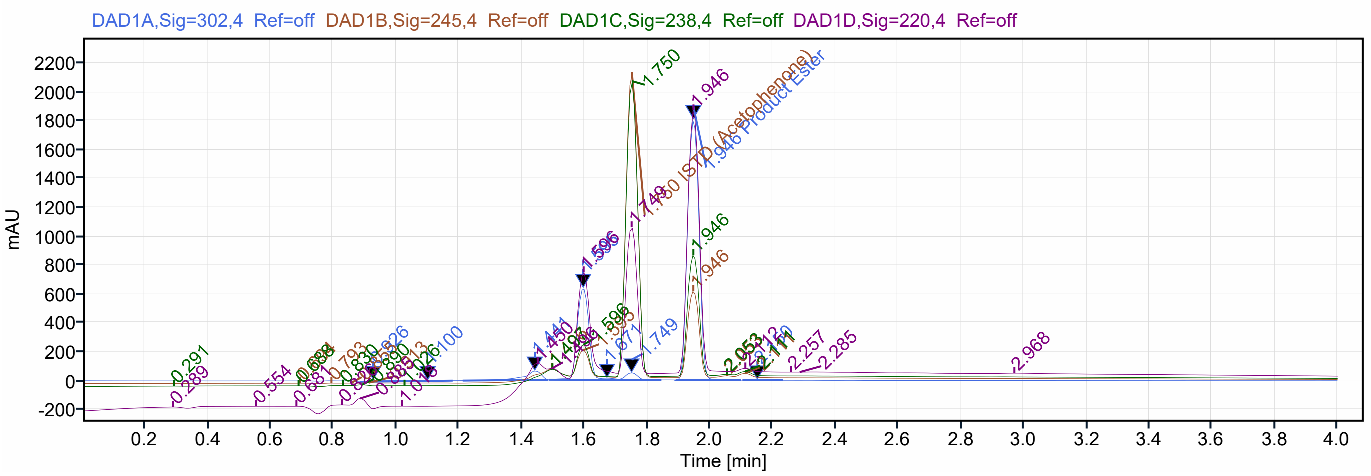

Sample Name	Name	RT (mins)	Area	Concentration (mg/L)
Vial 13	Umbelliferone			
Vial 13	Pivalic Anhydride			
Vial 13	DMAP			
Vial 13	ISTD (Acetophenone)	1.750	5379.5444	
Vial 13	Product Ester	1.946	4588.2126	

Injection Report - By Sample

Kineticolor

Sample name: Vial 14

Data file: 2024-07-03 13-34-49+01-00-16.dx

Operator: SYSTEM

Instrument: 1220 Infinity II HPLC

Injection date: 2024-07-03 13:35:42+01:00

Inj. volume: 5.000 µL

Location: 14

Acq. method: Barry's standard method_low flow_higher A.amx

Type: Sample

Processing method: HB Standard method.pmx

Manually modified: None

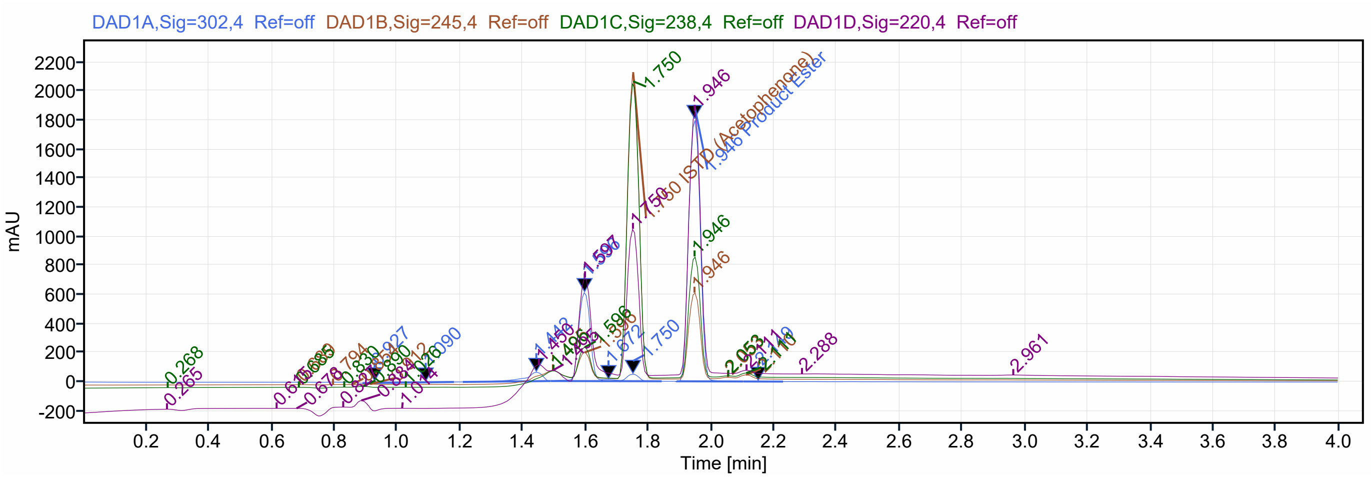

Sample Name	Name	RT (mins)	Area	Concentration (mg/L)
Vial 14	Umbelliferone			
Vial 14	Pivalic Anhydride			
Vial 14	DMAP			
Vial 14	ISTD (Acetophenone)	1.750	5361.3558	
Vial 14	Product Ester	1.946	4570.2064	

Injection Report - By Sample

Kineticolor

**Sample name:**	Vial 15		
**Data file:**	2024-07-03 13-39-46+01-00-17.dx	**Operator:**	SYSTEM
**Instrument:**	1220 Infinity II HPLC	**Injection date:**	2024-07-03 13:40:39+01:00
**Inj. volume:**	5.000 µL	**Location:**	15
**Acq. method:**	Barry's standard method_low flow_higher A.amx	**Type:**	Sample
**Processing method:**	HB Standard method.pmx		
**Manually modified:**	None		

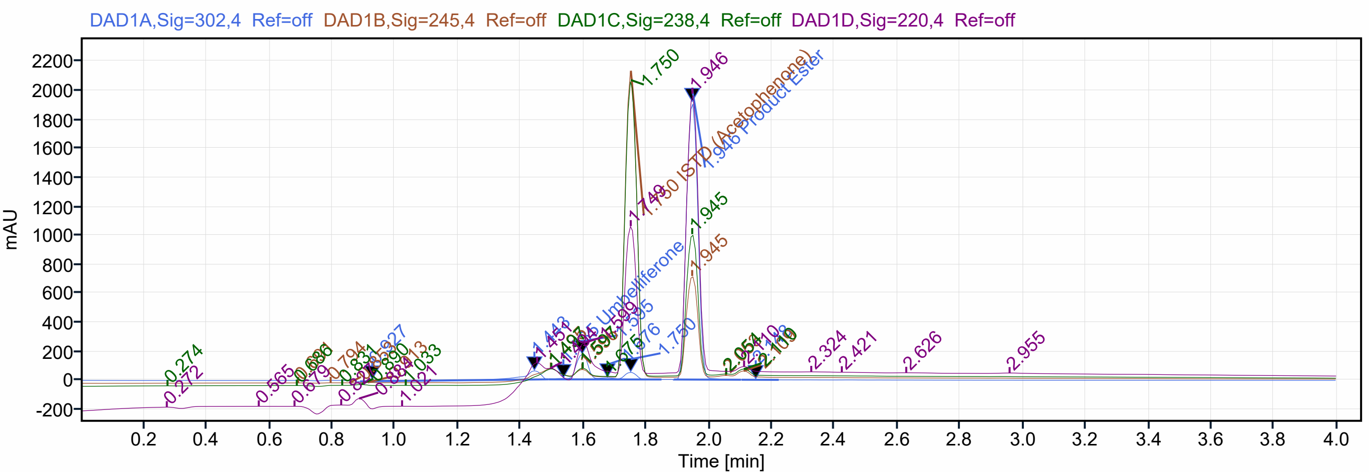

Sample Name	Name	RT (mins)	Area	Concentration (mg/L)
Vial 15	Pivalic Anhydride			
Vial 15	DMAP			
Vial 15	Umbelliferone	1.535	15.3227	
Vial 15	ISTD (Acetophenone)	1.750	5381.5545	
Vial 15	Product Ester	1.946	4888.0920	

Injection Report - By Sample

Kineticolor

**Sample name:**	Vial 16		
**Data file:**	2024-07-03 13-44-43+01-00-18.dx	**Operator:**	SYSTEM
**Instrument:**	1220 Infinity II HPLC	**Injection date:**	2024-07-03 13:45:39+01:00
**Inj. volume:**	5.000 µL	**Location:**	16
**Acq. method:**	Barry's standard method_low flow_higher A.amx	**Type:**	Sample
**Processing method:**	HB Standard method.pmx		
**Manually modified:**	None		

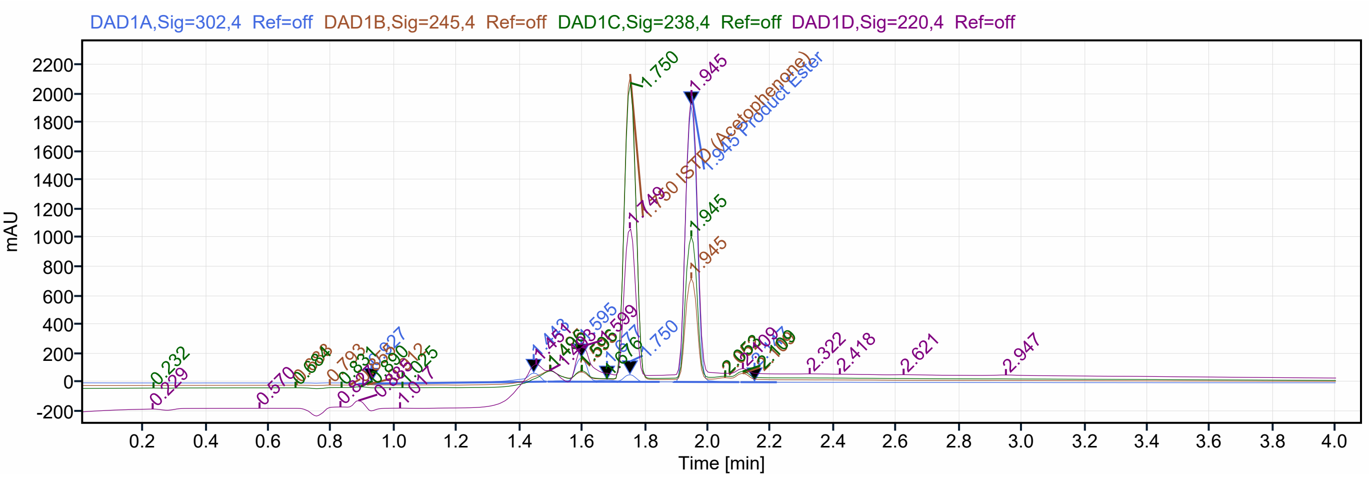

Sample Name	Name	RT (mins)	Area	Concentration (mg/L)
Vial 16	Umbelliferone			
Vial 16	Pivalic Anhydride			
Vial 16	DMAP			
Vial 16	ISTD (Acetophenone)	1.750	5367.1993	
Vial 16	Product Ester	1.945	4875.9956	

Sample name: Blank

Data file: 2024-07-03 13-49-42+01-00-19.dx

Operator: SYSTEM

Instrument: 1220 Infinity II HPLC

Injection date: 2024-07-03 13:50:36+01:00

Inj. volume: 5.000 µL

Location: 42

Acq. method: Barry's standard method_low flow_higher A.amx

Type: Sample

Processing method: HB Standard method.pmx

Manually modified: None

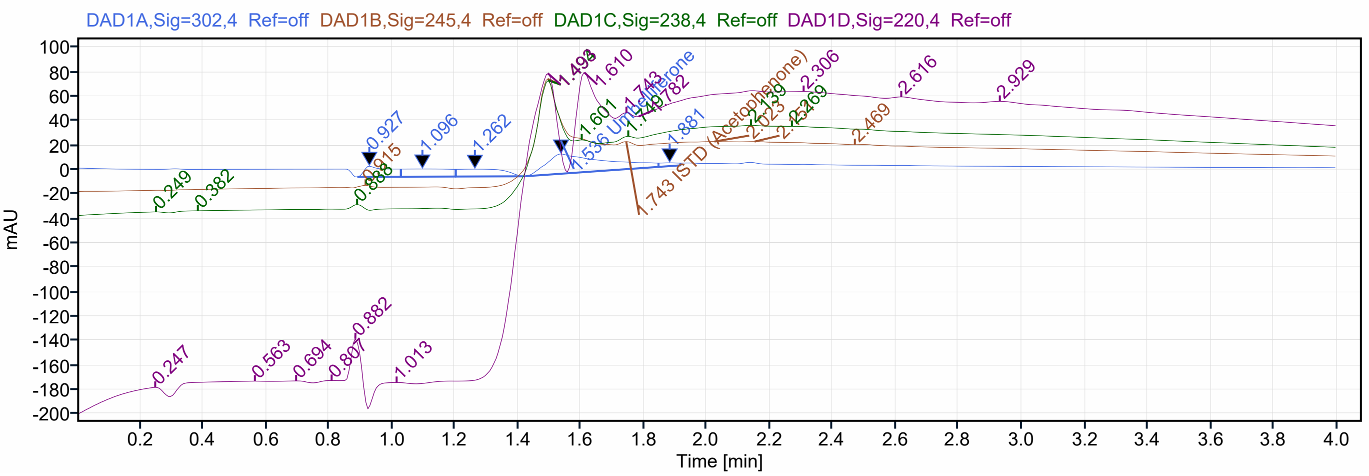

Sample Name	Name	RT (mins)	Area	Concentration (mg/L)
Blank	Product Ester			
Blank	Pivalic Anhydride			
Blank	DMAP			
Blank	Umbelliferone	1.536	206.7356	
Blank	ISTD (Acetophenone)	1.743	157.4472	

Sample name: Vial 17

Data file: 2024-07-03 13-54-40+01-00-20.dx

Operator: SYSTEM

Instrument: 1220 Infinity II HPLC

Injection date: 2024-07-03 13:55:34+01:00

Inj. volume: 5.000 µL

Location: 17

Acq. method: Barry's standard method_low flow_higher A.amx

Type: Sample

Processing method: HB Standard method.pmx

Manually modified: None

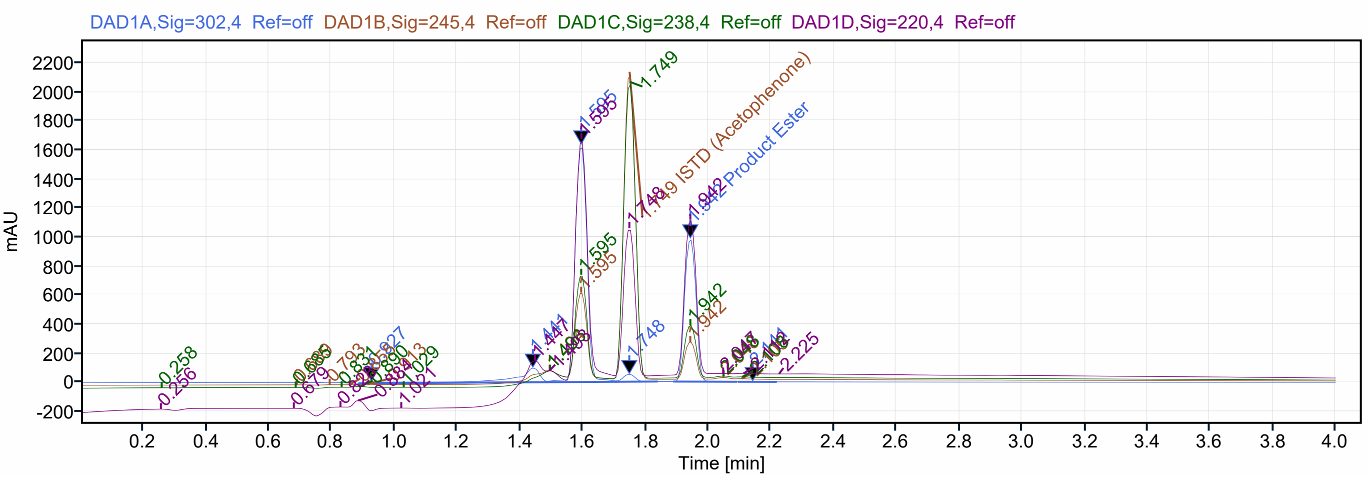

Sample Name	Name	RT (mins)	Area	Concentration (mg/L)
Vial 17	Umbelliferone			
Vial 17	Pivalic Anhydride			
Vial 17	DMAP			
Vial 17	ISTD (Acetophenone)	1.749	5376.4223	
Vial 17	Product Ester	1.942	2463.6647	

Injection Report - By Sample

Kineticolor

**Sample name:**	Vial 18		
**Data file:**	2024-07-03 13-59-38+01-00-21.dx	**Operator:**	SYSTEM
**Instrument:**	1220 Infinity II HPLC	**Injection date:**	2024-07-03 14:00:31+01:00
**Inj. volume:**	5.000 µL	**Location:**	18
**Acq. method:**	Barry's standard method_low flow_higher A.amx	**Type:**	Sample
**Processing method:**	HB Standard method.pmx		
**Manually modified:**	None		

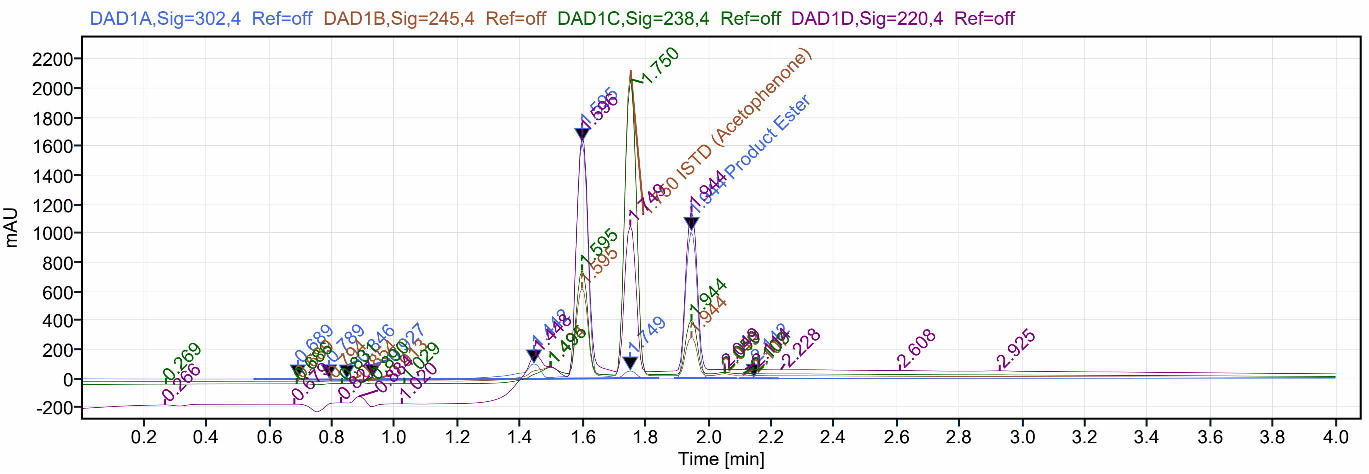

Sample Name	Name	RT (mins)	Area	Concentration (mg/L)
Vial 18	Umbelliferone			
Vial 18	Pivalic Anhydride			
Vial 18	DMAP			
Vial 18	ISTD (Acetophenone)	1.750	5347.5493	
Vial 18	Product Ester	1.944	2536.1326	

Injection Report - By Sample

Kineticolor

**Sample name:**	Vial 19		
**Data file:**	2024-07-03 14-04-35+01-00-22.dx	**Operator:**	SYSTEM
**Instrument:**	1220 Infinity II HPLC	**Injection date:**	2024-07-03 14:05:29+01:00
**Inj. volume:**	5.000 µL	**Location:**	19
**Acq. method:**	Barry's standard method_low flow_higher A.amx	**Type:**	Sample
**Processing method:**	HB Standard method.pmx		
**Manually modified:**	None		

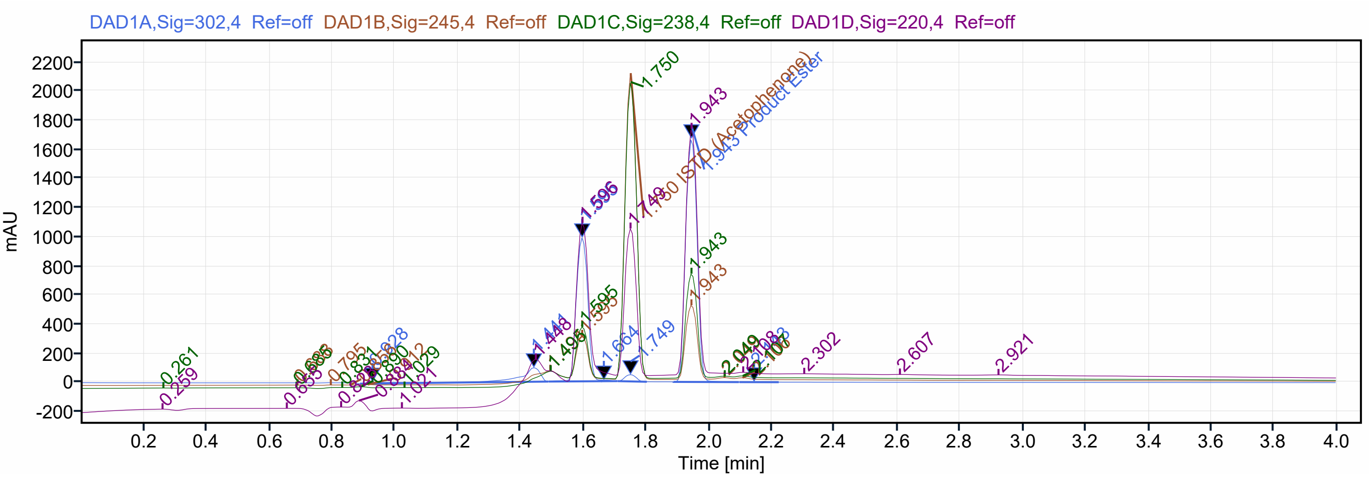

Sample Name	Name	RT (mins)	Area	Concentration (mg/L)
Vial 19	Umbelliferone			
Vial 19	Pivalic Anhydride			
Vial 19	DMAP			
Vial 19	ISTD (Acetophenone)	1.750	5336.5881	
Vial 19	Product Ester	1.943	4216.8989	

Sample name: Vial 20

Data file: 2024-07-03 14-09-33+01-00-23.dx

Operator: SYSTEM

Instrument: 1220 Infinity II HPLC

Injection date: 2024-07-03 14:10:30+01:00

Inj. volume: 5.000 µL

Location: 20

Acq. method: Barry's standard method_low flow_higher A.amx

Type: Sample

Processing method: HB Standard method.pmx

Manually modified: None

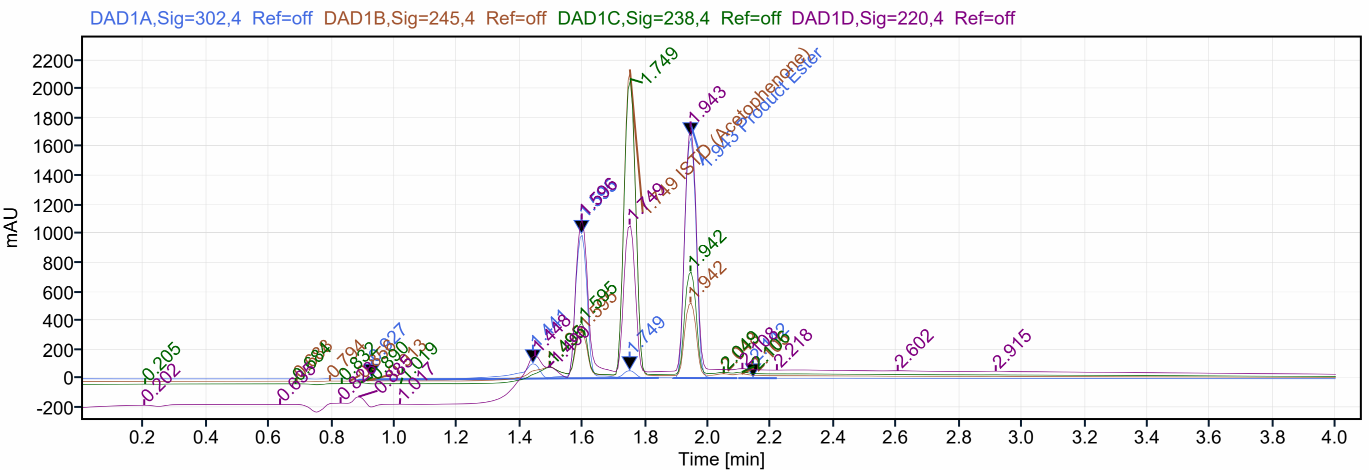

Sample Name	Name	RT (mins)	Area	Concentration (mg/L)
Vial 20	Umbelliferone			
Vial 20	Pivalic Anhydride			
Vial 20	DMAP			
Vial 20	ISTD (Acetophenone)	1.749	5361.2158	
Vial 20	Product Ester	1.943	4200.9050	

**Sample name:**	Vial 21		
**Data file:**	2024-07-03 14-14-33+01-00-24.dx	**Operator:**	SYSTEM
**Instrument:**	1220 Infinity II HPLC	**Injection date:**	2024-07-03 14:15:29+01:00
**Inj. volume:**	5.000 µL	**Location:**	21
**Acq. method:**	Barry's standard method_low flow_higher A.amx	**Type:**	Sample
**Processing method:**	HB Standard method.pmx		
**Manually modified:**	None		

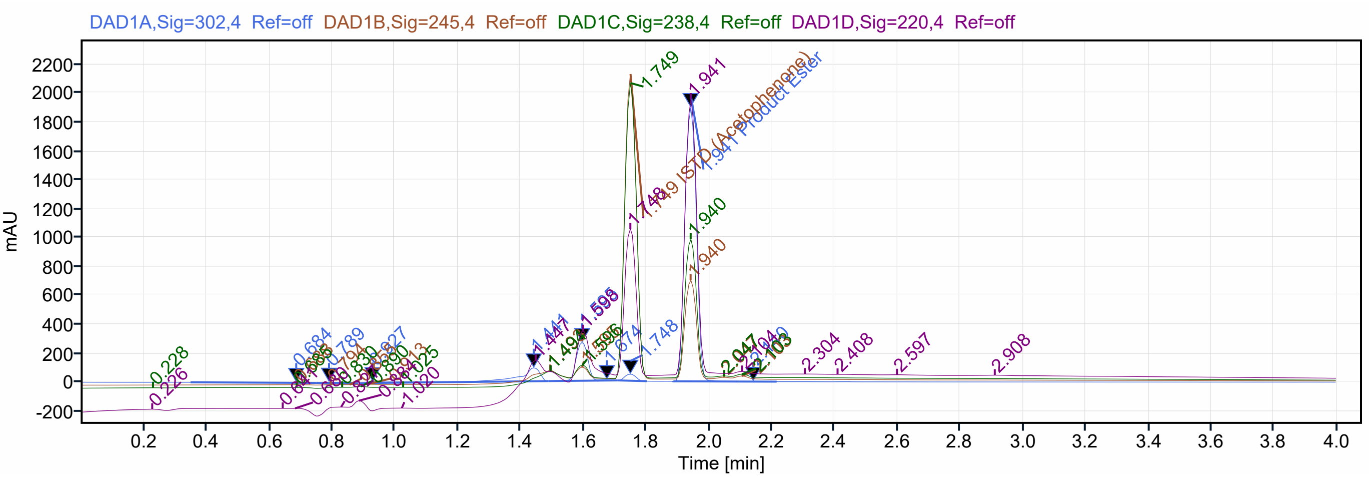

Sample Name	Name	RT (mins)	Area	Concentration (mg/L)
Vial 21	Umbelliferone			
Vial 21	Pivalic Anhydride			
Vial 21	DMAP			
Vial 21	ISTD (Acetophenone)	1.749	5357.7534	
Vial 21	Product Ester	1.941	4828.9888	

Injection Report - By Sample

Kineticolor

**Sample name:**	Vial 22		
**Data file:**	2024-07-03 14-19-33+01-00-25.dx	**Operator:**	SYSTEM
**Instrument:**	1220 Infinity II HPLC	**Injection date:**	2024-07-03 14:20:26+01:00
**Inj. volume:**	5.000 µL	**Location:**	22
**Acq. method:**	Barry's standard method_low flow_higher A.amx	**Type:**	Sample
**Processing method:**	HB Standard method.pmx		
**Manually modified:**	None		

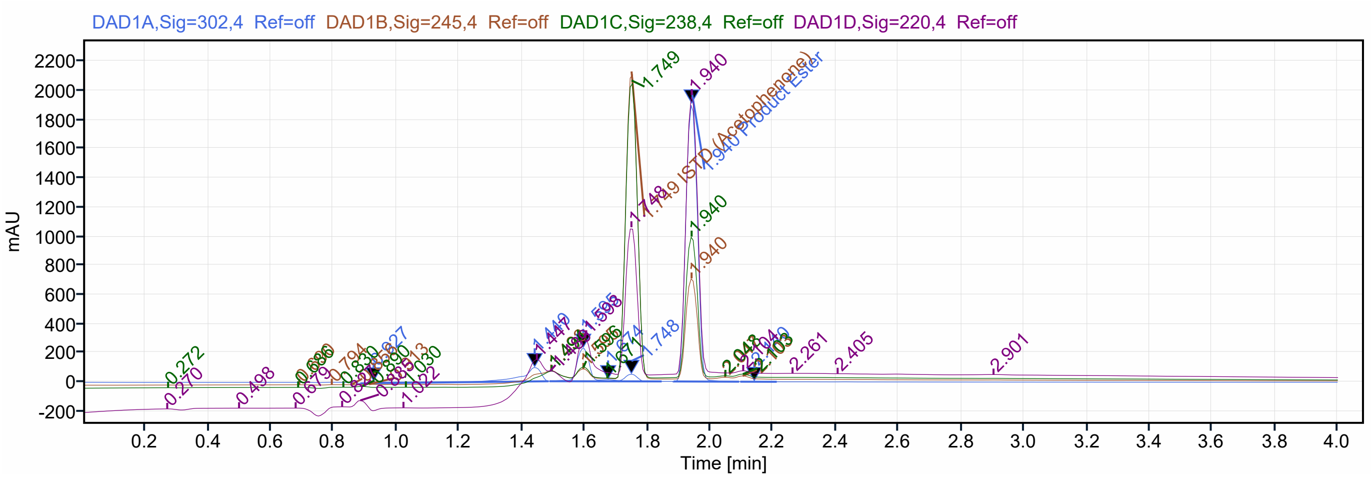

Sample Name	Name	RT (mins)	Area	Concentration (mg/L)
Vial 22	Umbelliferone			
Vial 22	Pivalic Anhydride			
Vial 22	DMAP			
Vial 22	ISTD (Acetophenone)	1.749	5344.3228	
Vial 22	Product Ester	1.940	4847.8936	

Injection Report - By Sample

Kineticolor

**Sample name:**	Vial 23		
**Data file:**	2024-07-03 14-24-30+01-00-26.dx	**Operator:**	SYSTEM
**Instrument:**	1220 Infinity II HPLC	**Injection date:**	2024-07-03 14:25:22+01:00
**Inj. volume:**	5.000 µL	**Location:**	23
**Acq. method:**	Barry's standard method_low flow_higher A.amx	**Type:**	Sample
**Processing method:**	HB Standard method.pmx		
**Manually modified:**	None		

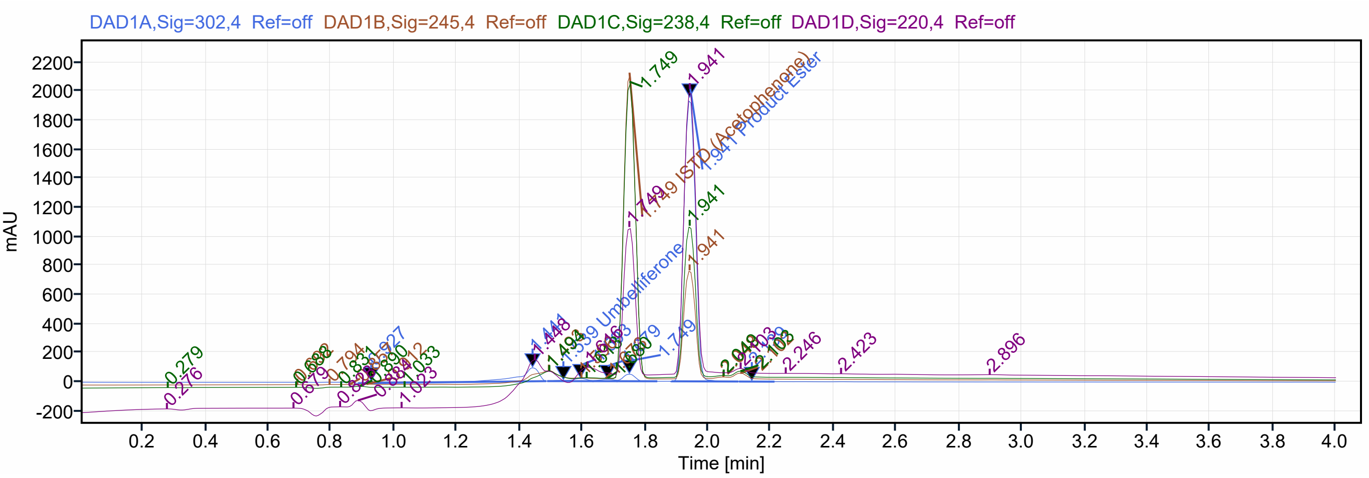

Sample Name	Name	RT (mins)	Area	Concentration (mg/L)
Vial 23	Pivalic Anhydride			
Vial 23	DMAP			
Vial 23	Umbelliferone	1.539	13.3851	
Vial 23	ISTD (Acetophenone)	1.749	5338.8670	
Vial 23	Product Ester	1.941	4981.0308	

Injection Report - By Sample

Kineticolor

**Sample name:**	Vial 24		
**Data file:**	2024-07-03 14-29-27+01-00-27.dx	**Operator:**	SYSTEM
**Instrument:**	1220 Infinity II HPLC	**Injection date:**	2024-07-03 14:30:21+01:00
**Inj. volume:**	5.000 µL	**Location:**	24
**Acq. method:**	Barry's standard method_low flow_higher A.amx	**Type:**	Sample
**Processing method:**	HB Standard method.pmx		
**Manually modified:**	None		

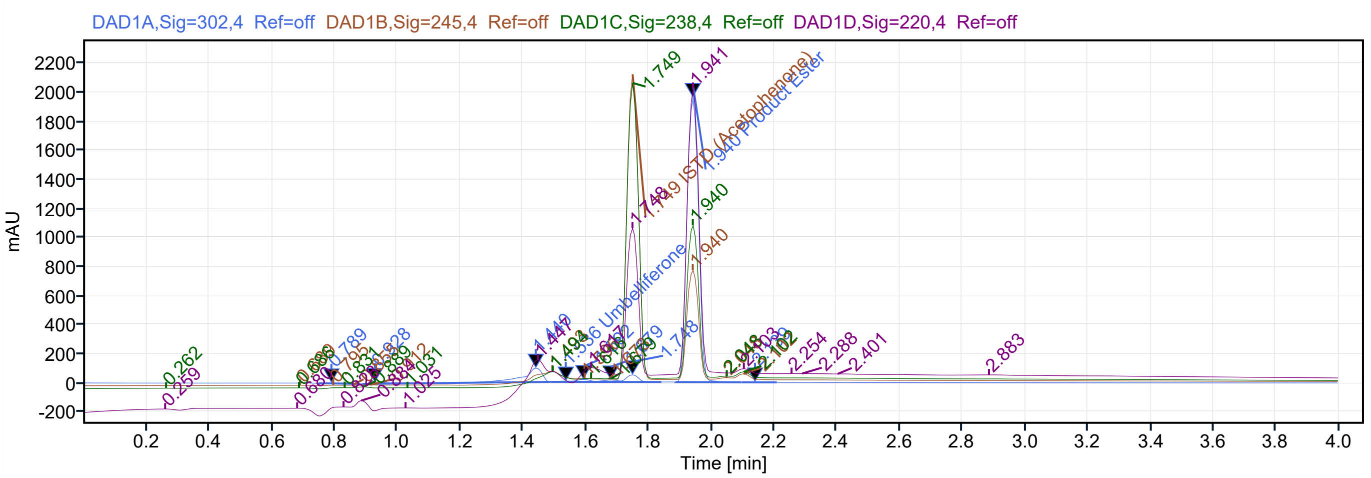

Sample Name	Name	RT (mins)	Area	Concentration (mg/L)
Vial 24	Pivalic Anhydride			
Vial 24	DMAP			
Vial 24	Umbelliferone	1.536	15.1549	
Vial 24	ISTD (Acetophenone)	1.749	5336.5247	
Vial 24	Product Ester	1.940	5013.3175	

Injection Report - By Sample

Kineticolor

Sample name: Blank

Data file: 2024-07-03 14-34-24+01-00-28.dx

Operator: SYSTEM

Instrument: 1220 Infinity II HPLC

Injection date: 2024-07-03 14:35:19+01:00

Inj. volume: 5.000 µL

Location: 42

Acq. method: Barry's standard method_low flow_higher A.amx

Type: Sample

Processing method: HB Standard method.pmx

Manually modified: None

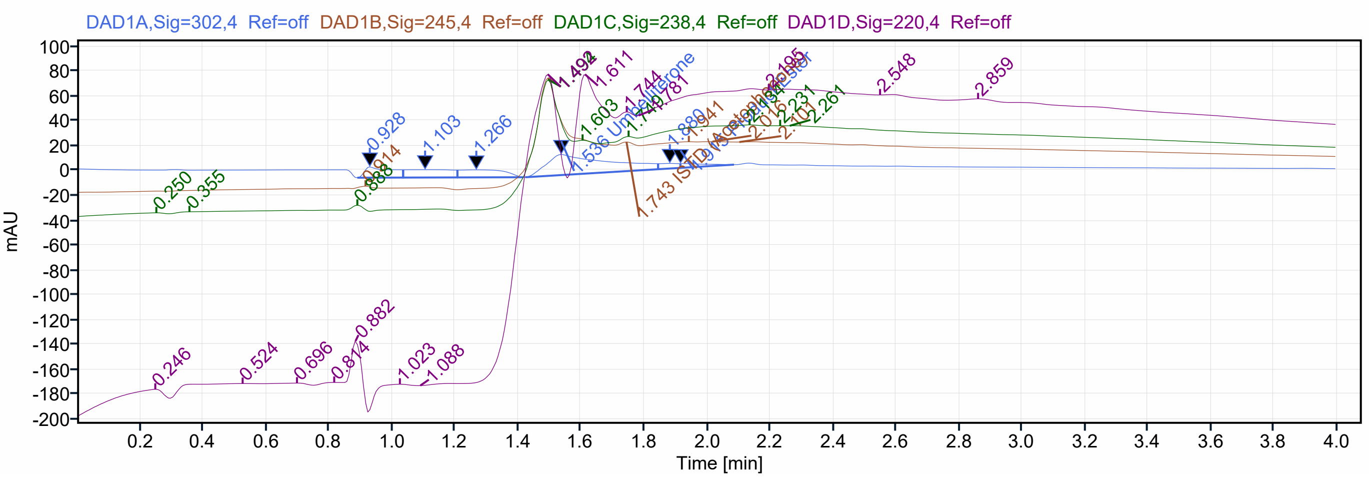

Sample Name	Name	RT (mins)	Area	Concentration (mg/L)
Blank	Pivalic Anhydride			
Blank	DMAP			
Blank	Umbelliferone	1.536	222.1513	
Blank	ISTD (Acetophenone)	1.743	109.2307	
Blank	Product Ester	1.915	17.4645	
