# Supplementary material for: Parallel and High Throughput Reaction Monitoring with Computer Vision
Source: Angew Chem Int Ed Engl. 2024 Oct 31;64(1):e202413395. doi: 10.1002/anie.202413395 (PMC11701362; doi:10.1002/anie.202413395)
Supplement: Supplementary file 3 — Supporting Information [file ANIE-64-e202413395-s003.zip › Supporting Info - Machine readable data part 2/Table 2 - esterification in HTE/HPLC Method.pdf]

Acquisition Method Report

Agilent

Acquisition Method: **Barry's standard method_low flow_higher A.amx**
Path: **C:\CDSProjects\1220 Infinity II\Results\HB\blank new.sirslt**

1 Method Information

**Last Saved As:**	C:\CDSProjects\1220 Infinity II\Methods\Barry's standard method_low flow_higher A.amx	**Modified:**	2024-07-05 15:53:49+01:00	**Modifier:**	SYSTEM
**Created:**	2024-03-28 09:35:46+00:00	**Creator:**	SYSTEM	**Description:**	
**Version:**	2024-0705-1453-49352	**Method Status:**	Generic		

2 DAD Method

**Peakwidth:**	> 0.1 min (2 s response time) (2.5 Hz)	**Slit:**	4 nm	**UV Lamp Required:**	Yes
**Vis Lamp Required:**	No	**Module Display Name:**	DAD	**Module Type:**	G4294B
**Order:**	1				

2.1 Analog Output 1

**Analog 1 Zero Offset:**	5 %	**Analog 1 Attenuation:**	1000 mAU

2.2 Analog Output 2

**Analog 2 Zero Offset:**	5 %	**Analog 2 Attenuation:**	1000 mAU

2.3 Signals

Signal table

Acquire	Signal	Wavelength	Bandwidth	Use Ref.
Yes	Signal A	302 nm	4 nm	No
Yes	Signal B	245 nm	4 nm	No
Yes	Signal C	238 nm	4 nm	No
Yes	Signal D	220 nm	4 nm	No
No	Signal E			
No	Signal F			
No	Signal G			
No	Signal H			

2.4 Prepare Mode

**Margin for negative Absorbance:**	100 mAU

2.5 Autobalance

**Autobalance Prerun:**	Yes	**Autobalance Postrun:**	No

2.6 Spectrum

**Spectrum Store:**	None

2.7 Stoptime

**Stoptime Mode:**	As Pump/Injector

2.8 Posttime

**Posttime Mode:**	Off

3 Column Oven Method

**Module Display Name:**	Column Oven	**Module Type:**	G4294B	**Order:**	1

3.1 Temperature Control

**Temperature Control Mode:**	Not Controlled

3.1.1 Temperature Not Ready Limit

**Temperature Not Ready Limit On:**	Yes	**Temperature Not Ready Limit Value:**	0.8 °C

3.2 Stoptime

**Stoptime Mode:**	As Pump/Injector

3.3 Posttime

**Posttime Mode:**	Off

4 Grad. Pump Method

**Flow:**	1.800 mL/min	**Low Pressure Limit:**	0.00 bar	**High Pressure Limit:**	600.00 bar
**Maximum Flow Gradient:**	100.000 mL/min²	**Primary Channel:**	Automatic	**Module Display Name:**	Grad. Pump
**Module Type:**	G4294B	**Order:**	1		

Solvent Composition

Empty columns are hidden.

Channel	Used	Percent
A	Yes	5.0 %
B	Yes	95.0 %

Timetable

Time	A	B	Flow	Pressure
Start. Cond. min	5.0 %	95.0 %	1.800 mL/min	600.00 bar
0.50 min	85.0 %	15.0 %	1.800 mL/min	600.00 bar
3.00 min	75.0 %	25.0 %	1.800 mL/min	600.00 bar
3.50 min	5.0 %	95.0 %	1.800 mL/min	600.00 bar
4.00 min	5.0 %	95.0 %	1.800 mL/min	600.00 bar

4.1 Stroke

Automatic Stroke Calculation: Yes

4.2 Compress

Compressibility Mode: Compressibility Value Set | **Compressibility:** 100 10e-6/bar

4.3 Stoptime

Stoptime Mode: Time set | **Stoptime:** 4.00 min

4.4 Posttime

Posttime Mode: Off

5 Sampler Method

Module Display Name: Sampler | **Module Type:** G4294B | **Order:** 1

5.1 Auxiliary

Draw Speed: 200 µL/min | **Eject Speed:** 200 µL/min | **Draw Position Offset:** 0.0 mm

5.2 Injection

Injection Mode: Standard injection | **Injection Volume:** 5.00 µL

5.3 High throughput

5.3.1 Overlapped Injection

Enable Overlapped Injection: No

5.4 Stoptime

Stoptime Mode: As Pump/No Limit

5.5 Posttime

Posttime Mode: Off

6 Method Properties

Instrument Technique: Liquid Chromatography

7 Schema version

Schema version: 2.3

Method Audit Trail

#	Username<br>Reviewed	Date (yyyy-MM-dd)<br>Version	Category<br>Hostname	Description<br>Reason
1	SYSTEM	2024-03-28 09:35:46+00:00	Method	Saved method 'C:\CDSProjects\1220 Infinity II\Methods\Barry's standard method_short_bchange.amx' version 2024-0320-1153-19432 as 'C:\CDSProjects\1220 Infinity II\Methods\Barry's standard method_low flow_higher A.amx'.
		2024-0328-0935-46677	chem-625-marcr.ds.strath.ac.uk	
2	SYSTEM	2024-03-28 09:36:02+00:00	Grad. Pump	Changed Flow from 2.000 mL/min to 1.800 mL/min
		2024-0328-0936-03077	chem-625-marcr.ds.strath.ac.uk	
3	SYSTEM	2024-03-28 09:36:02+00:00	Grad. Pump	Changed timetable entry from "Change Solvent Composition" at time 0.5 with parameter Solvent composition A: 75.0 % B:25.0 % to "Change Solvent Composition" at time 0.5 with parameter Solvent composition A: 85.0 % B:15.0 %
		2024-0328-0936-03077	chem-625-marcr.ds.strath.ac.uk	

#	Username<br>Reviewed	Date (yyyy-MM-dd)<br>Version	Category<br>Hostname	Description<br>Reason
4	SYSTEM	2024-03-28 10:12:24+00:00	Grad. Pump	Changed timetable entry from "Change Flow" at time 0.5 with parameter Flow: 2 mL/min to "Change Flow" at time 0.5 with parameter Flow: 1.8 mL/min
		2024-0328-1012-24858	chem-625-marcr.ds.strath.ac.uk	
5	SYSTEM	2024-03-28 10:12:24+00:00	Grad. Pump	Changed timetable entry from "Change Flow" at time 3 with parameter Flow: 2 mL/min to "Change Flow" at time 3 with parameter Flow: 1.8 mL/min
		2024-0328-1012-24858	chem-625-marcr.ds.strath.ac.uk	
6	SYSTEM	2024-03-28 10:12:24+00:00	Grad. Pump	Changed timetable entry from "Change Flow" at time 3.5 with parameter Flow: 2 mL/min to "Change Flow" at time 3.5 with parameter Flow: 1.8 mL/min
		2024-0328-1012-24858	chem-625-marcr.ds.strath.ac.uk	
7	SYSTEM	2024-03-28 10:12:24+00:00	Grad. Pump	Changed timetable entry from "Change Flow" at time 4 with parameter Flow: 2 mL/min to "Change Flow" at time 4 with parameter Flow: 1.8 mL/min
		2024-0328-1012-24858	chem-625-marcr.ds.strath.ac.uk	
8	SYSTEM	2024-07-05 15:53:49+01:00	Properties	Changed description from 'HPLC method for amide coupling reactions<br><br>Separating Fmoc-Ala-OH and 9,9-dimethylfluorene (ISTD)<br><br>' to '<br>'.
		2024-0705-1453-49352	chem-625-marcr.ds.strath.ac.uk	
