# Supplementary material for: Parallel and High Throughput Reaction Monitoring with Computer Vision
Source: Angew Chem Int Ed Engl. 2024 Oct 31;64(1):e202413395. doi: 10.1002/anie.202413395 (PMC11701362; doi:10.1002/anie.202413395)

"NMR 3.6.2 A" 1 1 "C:\Users\Barry\Documents\e-Lab Book\HB 3 Well Plate\HB 3.6 Umbelliferone Esterifi

Person wlb21183
HB 3.6.2a
@proton CDCl3 {C:\NMRdata} MR 15

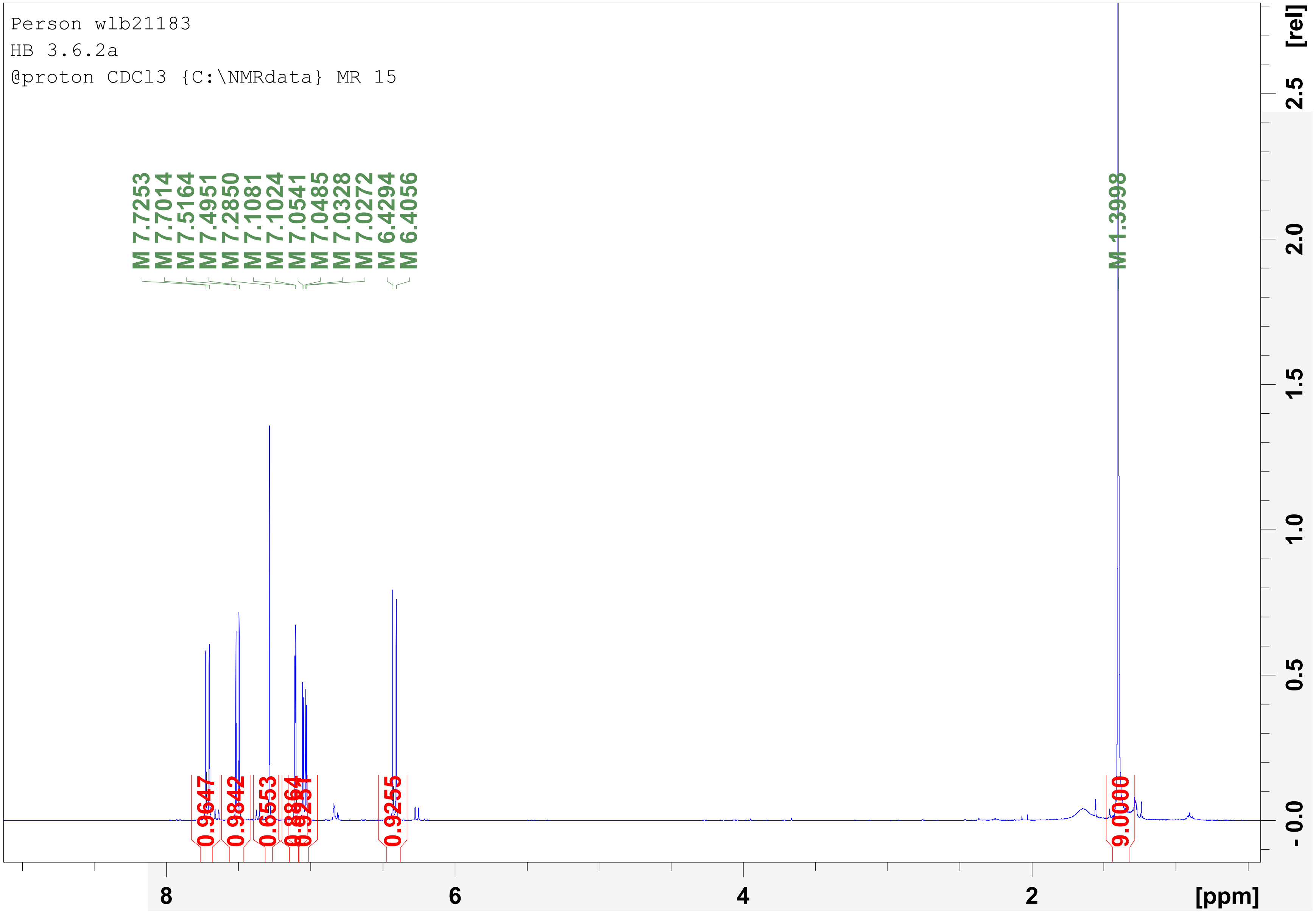

Supplement: Supplementary file 3 — Supporting Information [file ANIE-64-e202413395-s003.zip › Supporting Info - Machine readable data part 2/Figure 10 - esterification and mutual information/NMR_/Product NMR.pdf]
