# Supplementary material for: Parallel and High Throughput Reaction Monitoring with Computer Vision
Source: Angew Chem Int Ed Engl. 2024 Oct 31;64(1):e202413395. doi: 10.1002/anie.202413395 (PMC11701362; doi:10.1002/anie.202413395)
Supplement: Supplementary file 3 — Supporting Information [file ANIE-64-e202413395-s003.zip › Supporting Info - Machine readable data part 2/Figure 10 - esterification and mutual information/NMR_/Umbelliferone NMR.pdf]

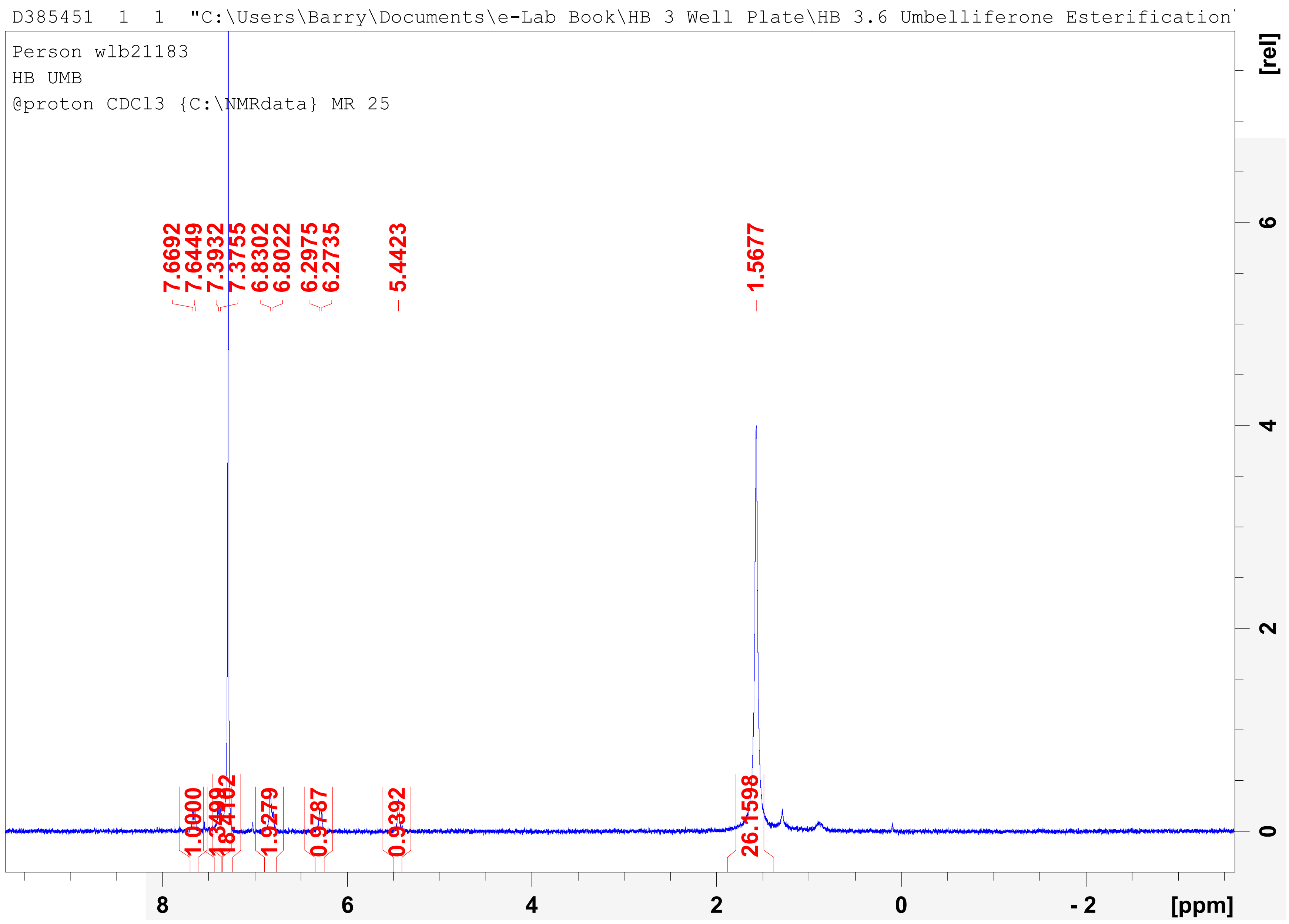
D385451 1 1 "C:\Users\Barry\Documents\e-Lab Book\HB 3 Well Plate\HB 3.6 Umbelliferone Esterification`
Person wlb21183
HB UMB
@proton CDCl3 {C:\NMRdata} MR 25
7.6692
7.6449
7.3932
7.3755
6.8302
6.8022
6.2975
6.2735
5.4423
1.5677
1.0000
1.3499
18.4102
1.9279
0.9787
0.9392
26.1598
[rel]
[ppm]
