# Supplementary material for: Parallel and High Throughput Reaction Monitoring with Computer Vision
Source: Angew Chem Int Ed Engl. 2024 Oct 31;64(1):e202413395. doi: 10.1002/anie.202413395 (PMC11701362; doi:10.1002/anie.202413395)
Supplement: Supplementary file 3 — Supporting Information [file ANIE-64-e202413395-s003.zip › Supporting Info - Machine readable data part 2/Figure 10 - esterification and mutual information/NMR_/Pivalic Anhydride NMR.pdf]

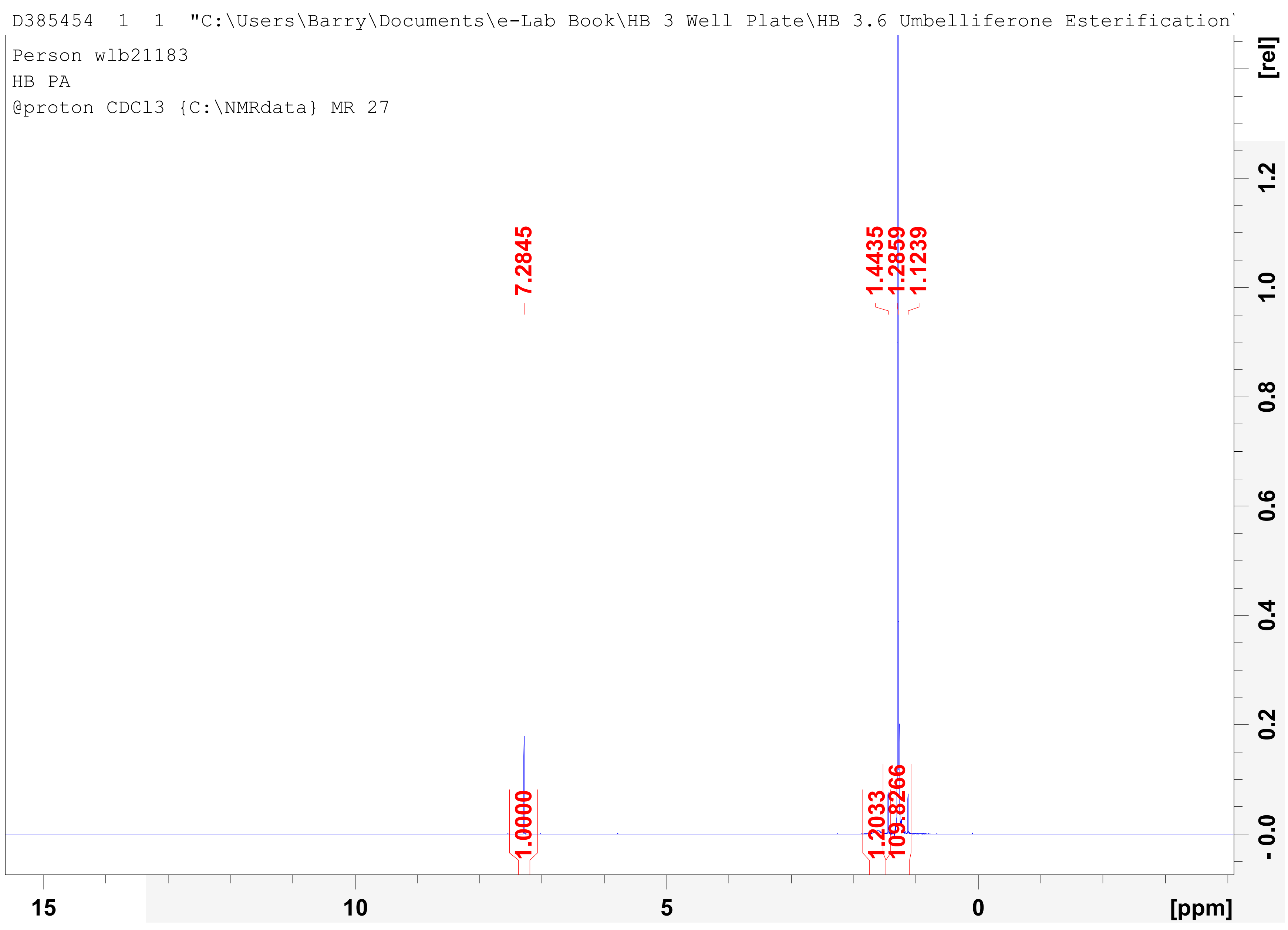
D385454 1 1 "C:\Users\Barry\Documents\e-Lab Book\HB 3 Well Plate\HB 3.6 Umbelliferone Esterification`
Person wlb21183
HB PA
@proton CDCl3 {C:\NMRdata} MR 27
7.2845
1.4435
1.2859
1.1239
1.0000
1.2033
109.8266
[rel]
1.2
1.0
0.8
0.6
0.4
0.2
-0.0
15
10
5
0
[ppm]
