# Supplementary material for: Parallel and High Throughput Reaction Monitoring with Computer Vision
Source: Angew Chem Int Ed Engl. 2024 Oct 31;64(1):e202413395. doi: 10.1002/anie.202413395 (PMC11701362; doi:10.1002/anie.202413395)
Supplement: Supplementary file 3 — Supporting Information [file ANIE-64-e202413395-s003.zip › Supporting Info - Machine readable data part 2/Figure 10 - esterification and mutual information/HPLC_/Ester kinetics 2.pdf]

Injection Report - By Sample

Kineticolor

Sample name: Blank

Data file: 2024-06-20 13-34-20+01-00-01.dx

Operator: SYSTEM

Instrument: 1220 Infinity II HPLC

Injection date: 2024-06-20 13:35:14+01:00

Inj. volume: 5.000 µL

Location: 31

Acq. method: Barry's standard method_low flow_higher A.amx

Type: Sample

Processing method: HB Standard method.pmx

Manually modified: None

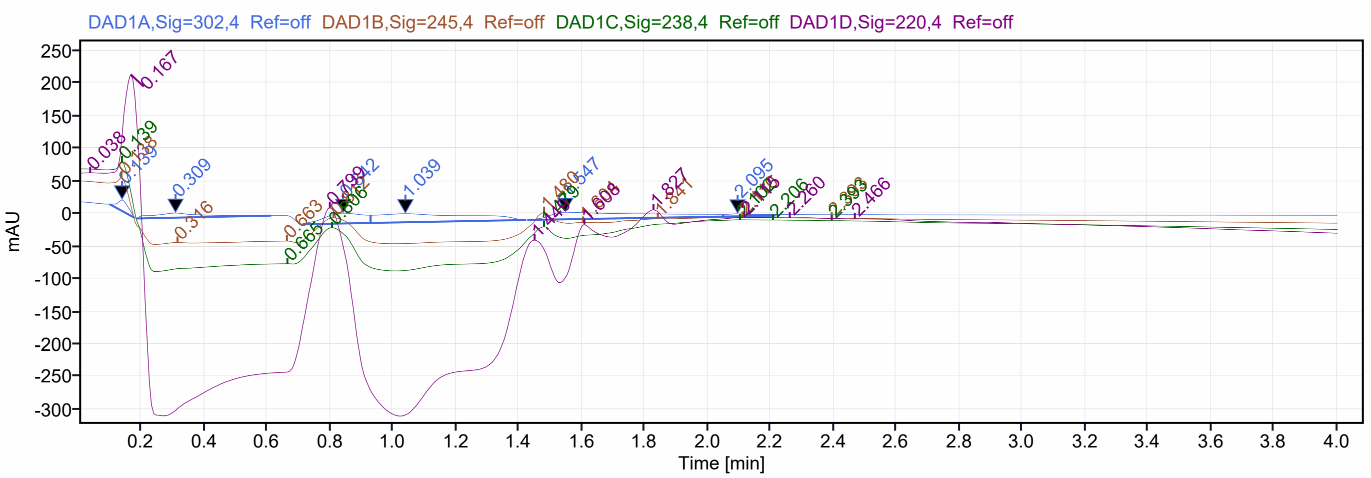

Sample Name	Name	RT (mins)	Area	Concentration (mg/L)
Blank	DMAP			
Blank	ISTD (Acetophenone)			
Blank	Pivalic Anhydride			
Blank	Product Ester			
Blank	Umbelliferone			

Injection Report - By Sample

Kineticolor

**Sample name:**	3 minutes		
**Data file:**	2024-06-20 13-39-18+01-00-02.dx	**Operator:**	SYSTEM
**Instrument:**	1220 Infinity II HPLC	**Injection date:**	2024-06-20 13:40:12+01:00
**Inj. volume:**	5.000 µL	**Location:**	1
**Acq. method:**	Barry's standard method_low flow_higher A.amx	**Type:**	Sample
**Processing method:**	HB Standard method.pmx		
**Manually modified:**	None		

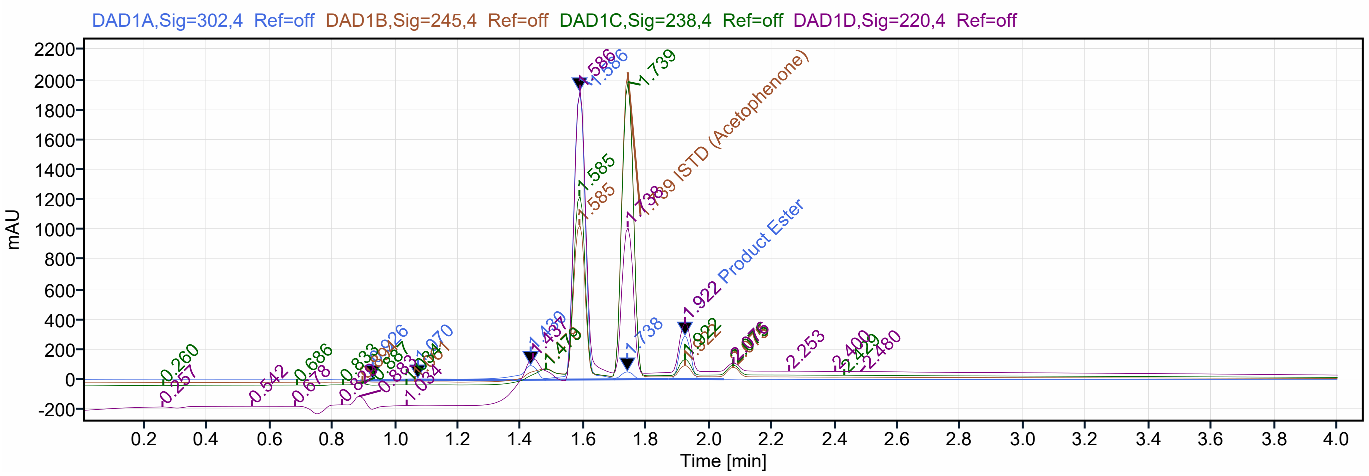

Sample Name	Name	RT (mins)	Area	Concentration (mg/L)
3 minutes	Umbelliferone			
3 minutes	Pivalic Anhydride			
3 minutes	DMAP			
3 minutes	ISTD (Acetophenone)	1.739	5152.7556	
3 minutes	Product Ester	1.922	714.0738	

Injection Report - By Sample

Kineticolor

**Sample name:**	6 minutes
**Data file:**	2024-06-20 13-44-15+01-00-03.dx
**Operator:**	SYSTEM
**Instrument:**	1220 Infinity II HPLC
**Injection date:**	2024-06-20 13:45:10+01:00
**Inj. volume:**	5.000 µL
**Location:**	2
**Acq. method:**	Barry's standard method_low flow_higher A.amx
**Type:**	Sample
**Processing method:**	HB Standard method.pmx
**Manually modified:**	None

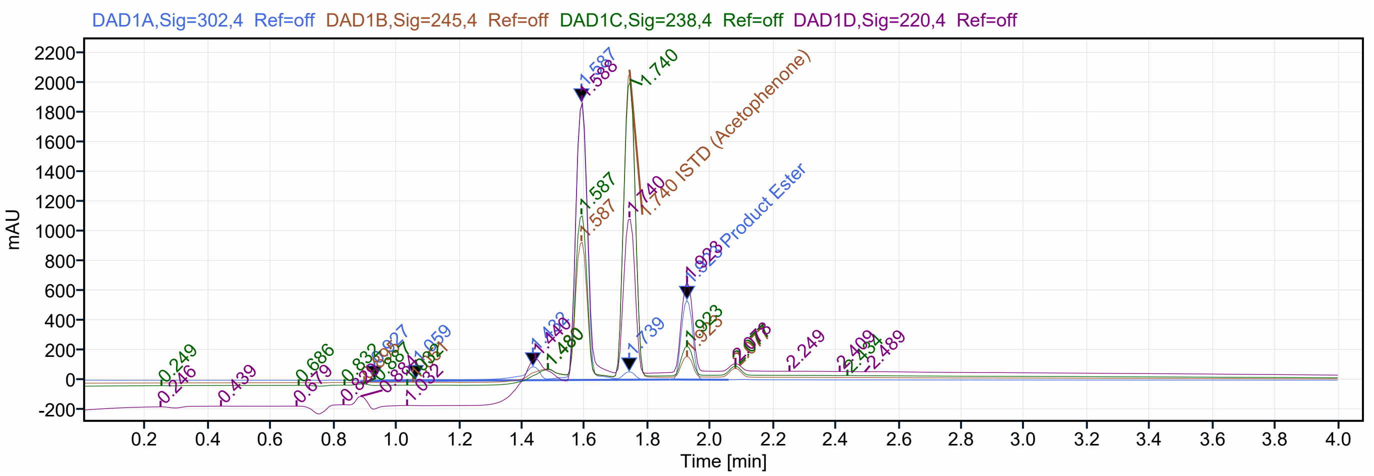

Sample Name	Name	RT (mins)	Area	Concentration (mg/L)
6 minutes	Umbelliferone			
6 minutes	Pivalic Anhydride			
6 minutes	DMAP			
6 minutes	ISTD (Acetophenone)	1.740	5248.6096	
6 minutes	Product Ester	1.923	1325.2575	

Injection Report - By Sample

Kineticolor

Sample name: 9 minutes

Data file: 2024-06-20 13-49-14+01-00-04.dx

Operator: SYSTEM

Instrument: 1220 Infinity II HPLC

Injection date: 2024-06-20 13:50:07+01:00

Inj. volume: 5.000 µL

Location: 3

Acq. method: Barry's standard method_low flow_higher A.amx

Type: Sample

Processing method: HB Standard method.pmx

Manually modified: None

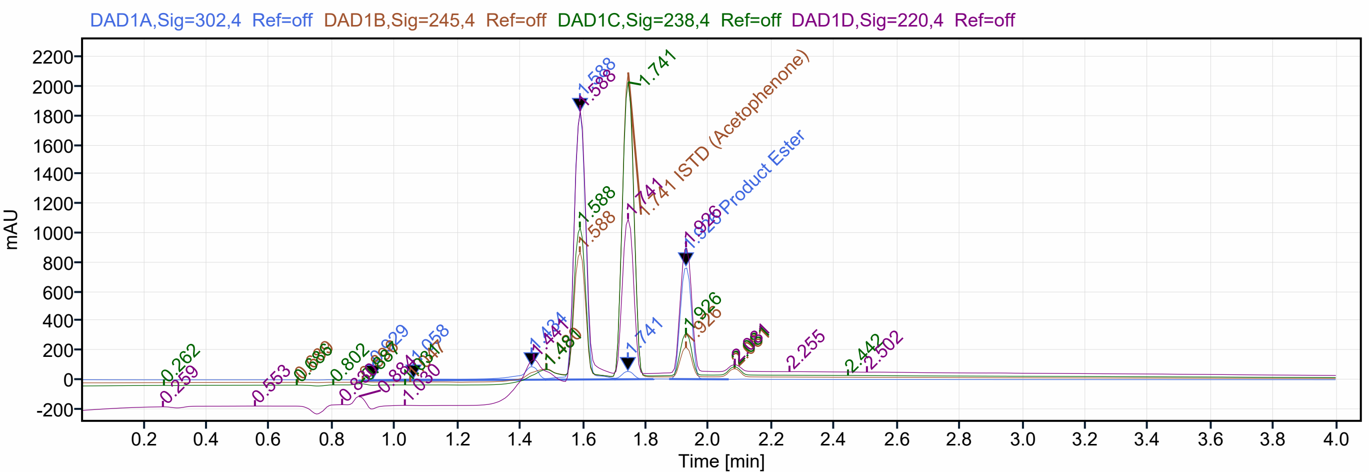

Sample Name	Name	RT (mins)	Area	Concentration (mg/L)
9 minutes	Umbelliferone			
9 minutes	Pivalic Anhydride			
9 minutes	DMAP			
9 minutes	ISTD (Acetophenone)	1.741	5273.6702	
9 minutes	Product Ester	1.926	1904.4437	

**Sample name:**	12 minutes		
**Data file:**	2024-06-20 13-54-11+01-00-05.dx	**Operator:**	SYSTEM
**Instrument:**	1220 Infinity II HPLC	**Injection date:**	2024-06-20 13:55:05+01:00
**Inj. volume:**	5.000 µL	**Location:**	4
**Acq. method:**	Barry's standard method_low flow_higher A.amx	**Type:**	Sample
**Processing method:**	HB Standard method.pmx		
**Manually modified:**	None		

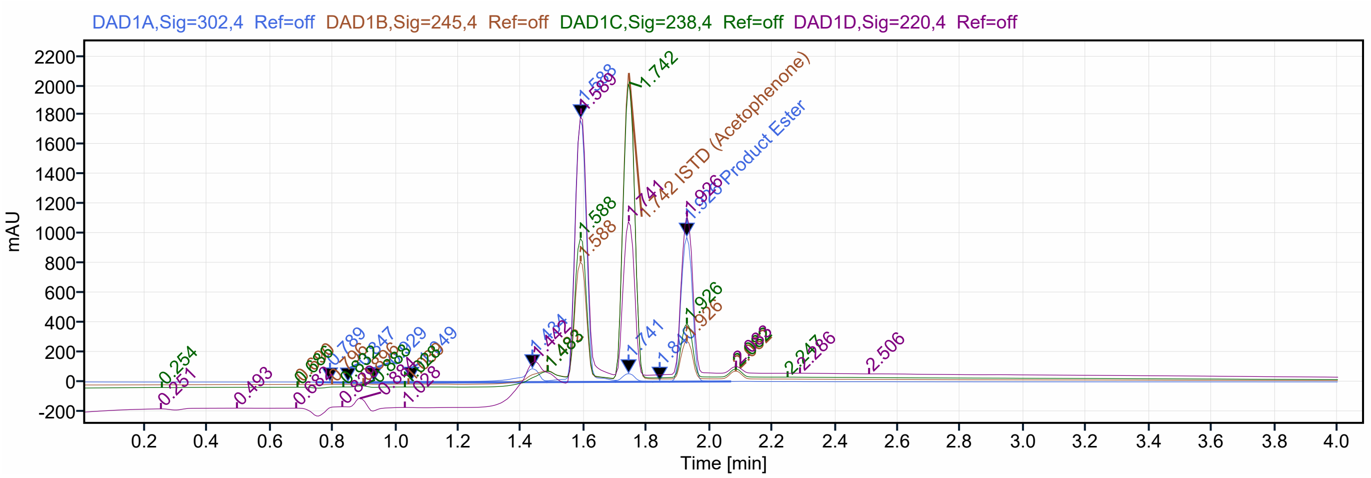

Sample Name	Name	RT (mins)	Area	Concentration (mg/L)
12 minutes	Umbelliferone			
12 minutes	Pivalic Anhydride			
12 minutes	DMAP			
12 minutes	ISTD (Acetophenone)	1.742	5249.1061	
12 minutes	Product Ester	1.926	2443.0522	

Injection Report - By Sample

Kineticolor

Sample name: 15 minutes

Data file: 2024-06-20 13-59-09+01-00-06.dx

Operator: SYSTEM

Instrument: 1220 Infinity II HPLC

Injection date: 2024-06-20 14:00:03+01:00

Inj. volume: 5.000 µL

Location: 5

Acq. method: Barry's standard method_low flow_higher A.amx

Type: Sample

Processing method: HB Standard method.pmx

Manually modified: None

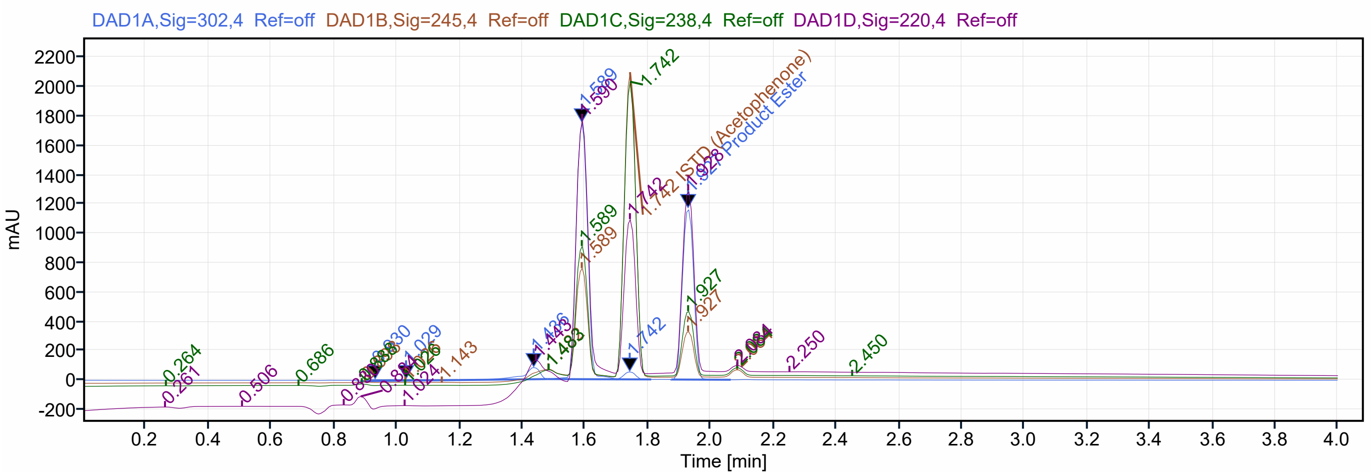

Sample Name	Name	RT (mins)	Area	Concentration (mg/L)
15 minutes	Umbelliferone			
15 minutes	Pivalic Anhydride			
15 minutes	DMAP			
15 minutes	ISTD (Acetophenone)	1.742	5290.1407	
15 minutes	Product Ester	1.927	2906.9047	

Sample name: 18 minutes

Data file: 2024-06-20 14-04-07+01-00-07.dx

Operator: SYSTEM

Instrument: 1220 Infinity II HPLC

Injection date: 2024-06-20 14:05:00+01:00

Inj. volume: 5.000 µL

Location: 6

Acq. method: Barry's standard method_low flow_higher A.amx

Type: Sample

Processing method: HB Standard method.pmx

Manually modified: None

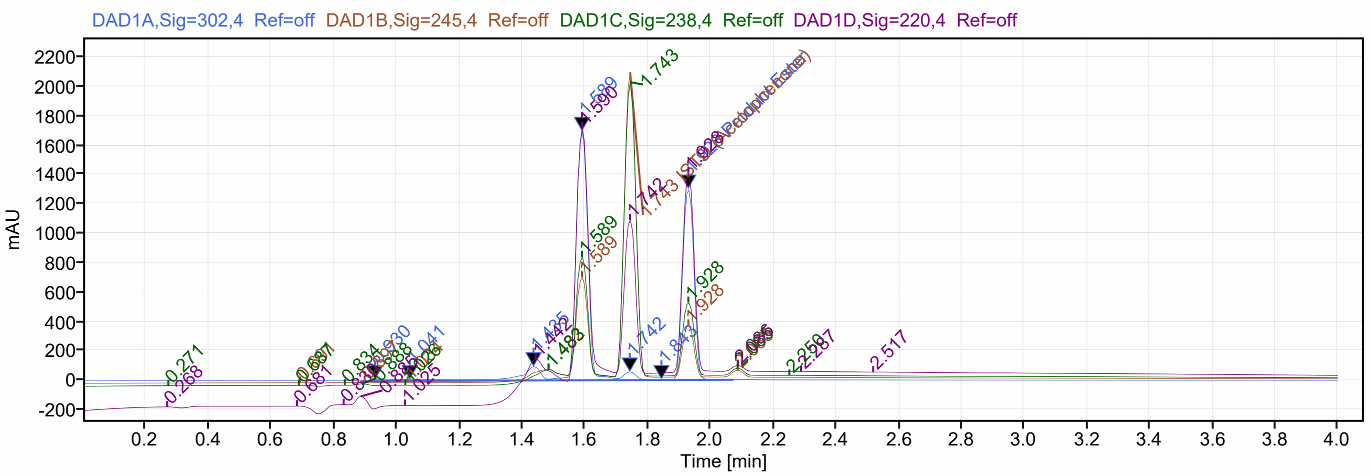

Sample Name	Name	RT (mins)	Area	Concentration (mg/L)
18 minutes	Umbelliferone			
18 minutes	Pivalic Anhydride			
18 minutes	DMAP			
18 minutes	ISTD (Acetophenone)	1.743	5271.7278	
18 minutes	Product Ester	1.928	3252.1277	

Injection Report - By Sample

Kineticolor

**Sample name:**	21 minutes		
**Data file:**	2024-06-20 14-09-03+01-00-08.dx	**Operator:**	SYSTEM
**Instrument:**	1220 Infinity II HPLC	**Injection date:**	2024-06-20 14:09:58+01:00
**Inj. volume:**	5.000 µL	**Location:**	7
**Acq. method:**	Barry's standard method_low flow_higher A.amx	**Type:**	Sample
**Processing method:**	HB Standard method.pmx		
**Manually modified:**	None		

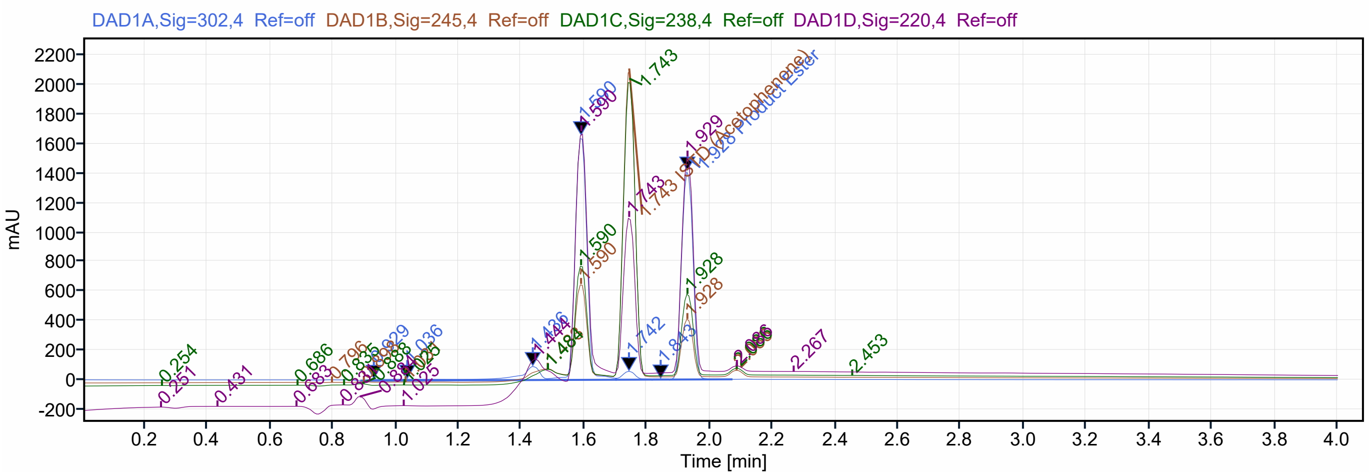

Sample Name	Name	RT (mins)	Area	Concentration (mg/L)
21 minutes	Umbelliferone			
21 minutes	Pivalic Anhydride			
21 minutes	DMAP			
21 minutes	ISTD (Acetophenone)	1.743	5319.4373	
21 minutes	Product Ester	1.928	3543.6739	

Sample name: 24 minutes

Data file: 2024-06-20 14-14-02+01-00-09.dx

Operator: SYSTEM

Instrument: 1220 Infinity II HPLC

Injection date: 2024-06-20 14:14:56+01:00

Inj. volume: 5.000 µL

Location: 8

Acq. method: Barry's standard method_low flow_higher A.amx

Type: Sample

Processing method: HB Standard method.pmx

Manually modified: None

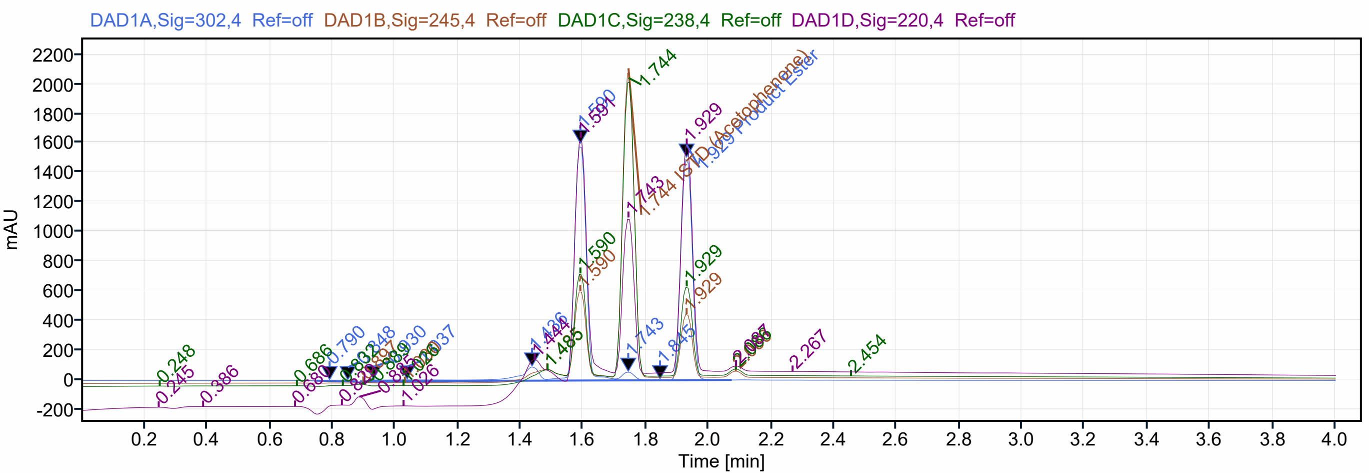

Sample Name	Name	RT (mins)	Area	Concentration (mg/L)
24 minutes	Umbelliferone			
24 minutes	Pivalic Anhydride			
24 minutes	DMAP			
24 minutes	ISTD (Acetophenone)	1.744	5297.6385	
24 minutes	Product Ester	1.929	3757.9848	

Kineticolor

**Sample name:**	Blank
**Data file:**	2024-06-20 14-19-00+01-00-10.dx
**Operator:**	SYSTEM
**Instrument:**	1220 Infinity II HPLC
**Injection date:**	2024-06-20 14:19:56+01:00
**Inj. volume:**	5.000 µL
**Location:**	31
**Acq. method:**	Barry's standard method_low flow_higher A.amx
**Type:**	Sample
**Processing method:**	HB Standard method.pmx
**Manually modified:**	None

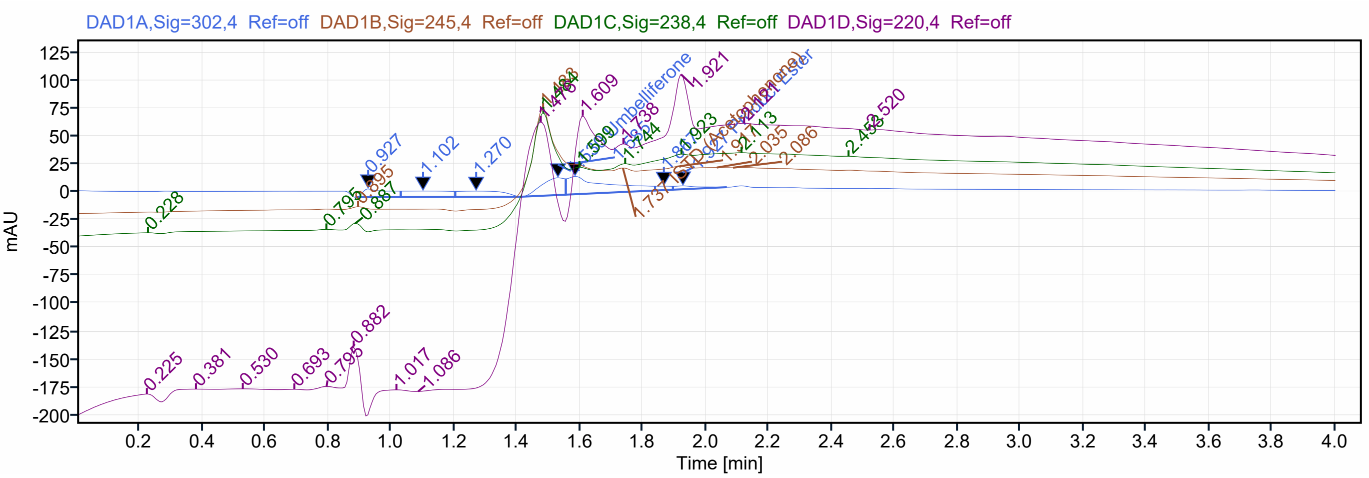

Sample Name	Name	RT (mins)	Area	Concentration (mg/L)
Blank	Pivalic Anhydride			
Blank	DMAP			
Blank	Umbelliferone	1.530	78.1308	
Blank	ISTD (Acetophenone)	1.737	108.8160	
Blank	Product Ester	1.927	16.5345	

Injection Report - By Sample

Kineticolor

Sample name: 27 minutes

Data file: 2024-06-20 14-24-00+01-00-11.dx

Operator: SYSTEM

Instrument: 1220 Infinity II HPLC

Injection date: 2024-06-20 14:24:57+01:00

Inj. volume: 5.000 µL

Location: 9

Acq. method: Barry's standard method_low flow_higher A.amx

Type: Sample

Processing method: HB Standard method.pmx

Manually modified: None

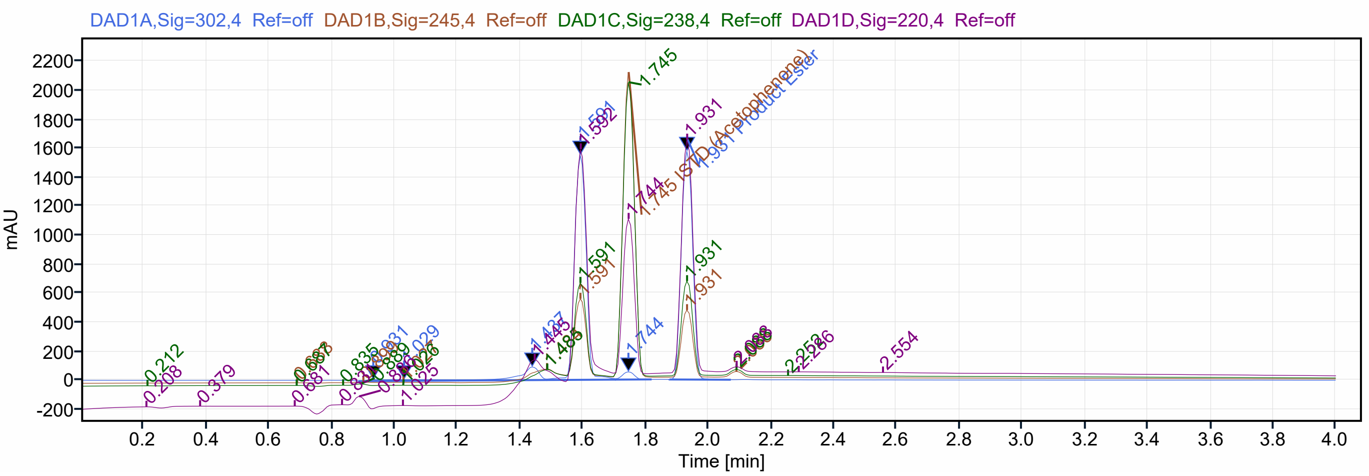

Sample Name	Name	RT (mins)	Area	Concentration (mg/L)
27 minutes	Umbelliferone			
27 minutes	Pivalic Anhydride			
27 minutes	DMAP			
27 minutes	ISTD (Acetophenone)	1.745	5362.3969	
27 minutes	Product Ester	1.931	3947.0626	

Injection Report - By Sample

Kineticolor

Sample name: 30 minutes

Data file: 2024-06-20 14-29-01+01-00-12.dx

Operator: SYSTEM

Instrument: 1220 Infinity II HPLC

Injection date: 2024-06-20 14:30:03+01:00

Inj. volume: 5.000 µL

Location: 10

Acq. method: Barry's standard method_low flow_higher A.amx

Type: Sample

Processing method: HB Standard method.pmx

Manually modified: None

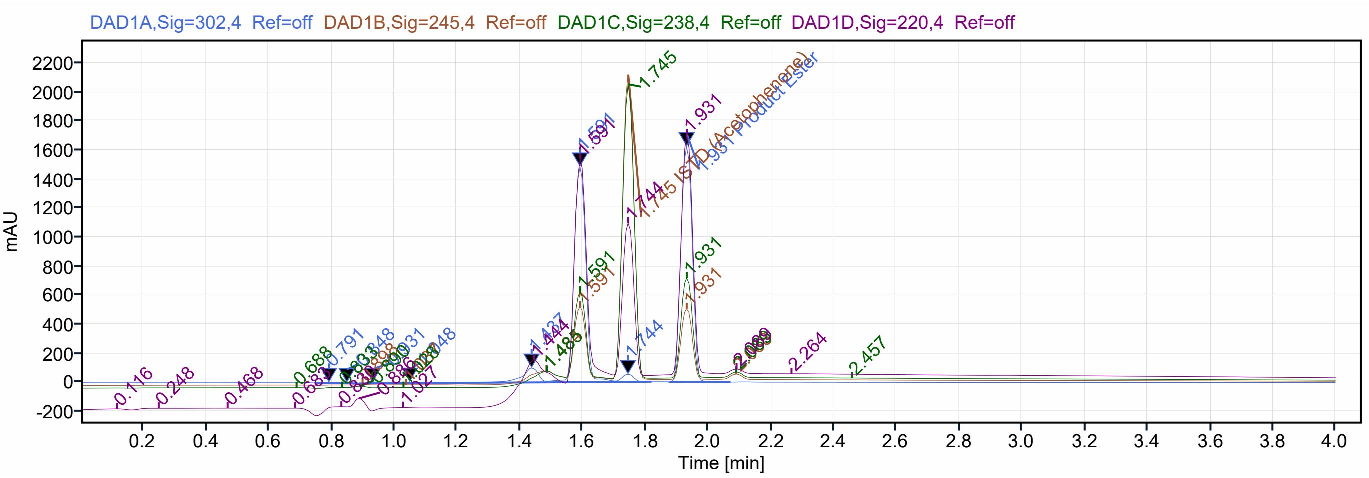

Sample Name	Name	RT (mins)	Area	Concentration (mg/L)
30 minutes	Umbelliferone			
30 minutes	Pivalic Anhydride			
30 minutes	DMAP			
30 minutes	ISTD (Acetophenone)	1.745	5347.6197	
30 minutes	Product Ester	1.931	4065.1656	

Injection Report - By Sample

Kineticolor

**Sample name:**	35 minutes
**Data file:**	2024-06-20 14-34-07+01-00-13.dx
**Instrument:**	1220 Infinity II HPLC
**Inj. volume:**	5.000 µL
**Acq. method:**	Barry's standard method_low flow_higher A.amx
**Processing method:**	HB Standard method.pmx
**Manually modified:**	None

**Operator:**	SYSTEM
**Injection date:**	2024-06-20 14:35:02+01:00
**Location:**	11
**Type:**	Sample

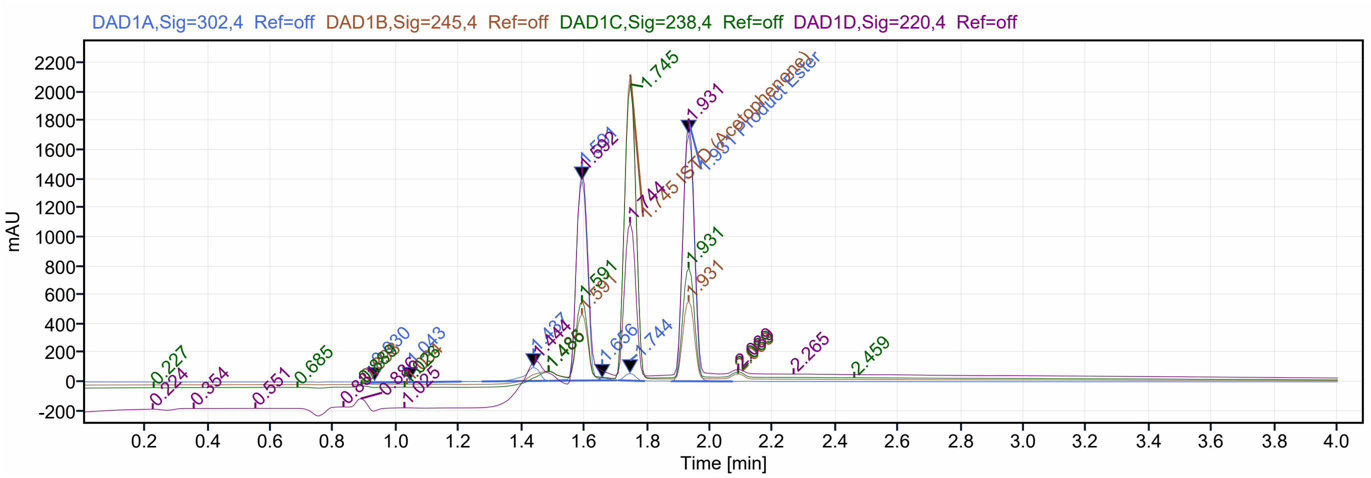

Sample Name	Name	RT (mins)	Area	Concentration (mg/L)
35 minutes	Umbelliferone			
35 minutes	Pivalic Anhydride			
35 minutes	DMAP			
35 minutes	ISTD (Acetophenone)	1.745	5352.6296	
35 minutes	Product Ester	1.931	4286.3188	

Injection Report - By Sample

Kineticolor

Sample name: 40 minutes

Data file: 2024-06-20 14-39-06+01-00-14.dx

Operator: SYSTEM

Instrument: 1220 Infinity II HPLC

Injection date: 2024-06-20 14:39:59+01:00

Inj. volume: 5.000 µL

Location: 12

Acq. method: Barry's standard method_low flow_higher A.amx

Type: Sample

Processing method: HB Standard method.pmx

Manually modified: None

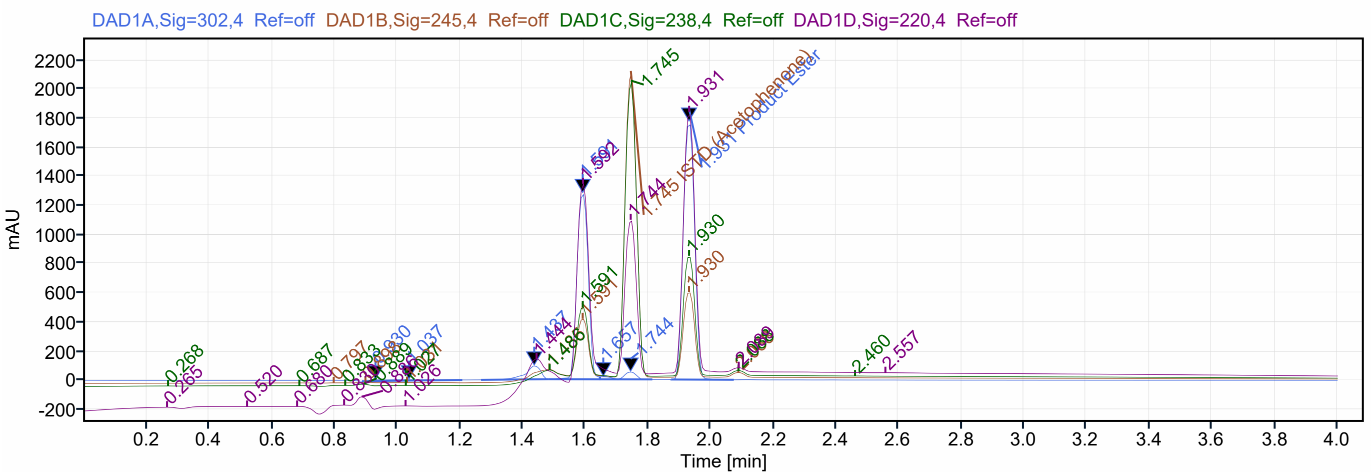

Sample Name	Name	RT (mins)	Area	Concentration (mg/L)
40 minutes	Umbelliferone			
40 minutes	Pivalic Anhydride			
40 minutes	DMAP			
40 minutes	ISTD (Acetophenone)	1.745	5362.7817	
40 minutes	Product Ester	1.931	4459.8039	

Injection Report - By Sample

Kineticolor

**Sample name:**	45 minutes		
**Data file:**	2024-06-20 14-44-04+01-00-15.dx	**Operator:**	SYSTEM
**Instrument:**	1220 Infinity II HPLC	**Injection date:**	2024-06-20 14:44:57+01:00
**Inj. volume:**	5.000 µL	**Location:**	13
**Acq. method:**	Barry's standard method_low flow_higher A.amx	**Type:**	Sample
**Processing method:**	HB Standard method.pmx		
**Manually modified:**	None		

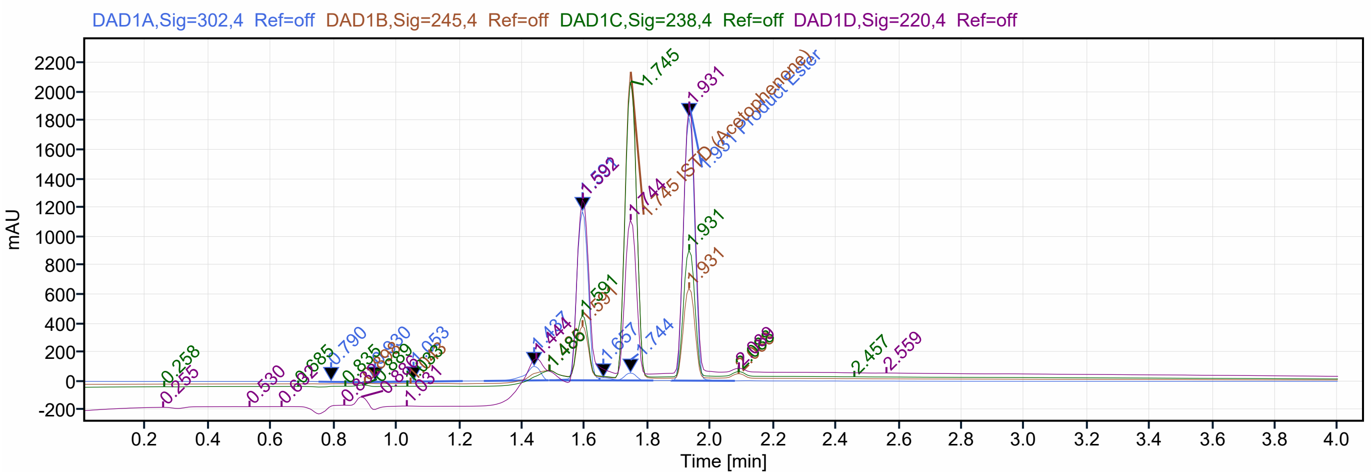

Sample Name	Name	RT (mins)	Area	Concentration (mg/L)
45 minutes	Umbelliferone			
45 minutes	Pivalic Anhydride			
45 minutes	DMAP			
45 minutes	ISTD (Acetophenone)	1.745	5400.5640	
45 minutes	Product Ester	1.931	4591.9478	

Injection Report - By Sample

Kineticolor

Sample name: 50 minutes

Data file: 2024-06-20 14-49-01+01-00-16.dx

Operator: SYSTEM

Instrument: 1220 Infinity II HPLC

Injection date: 2024-06-20 14:49:55+01:00

Inj. volume: 5.000 µL

Location: 14

Acq. method: Barry's standard method_low flow_higher A.amx

Type: Sample

Processing method: HB Standard method.pmx

Manually modified: None

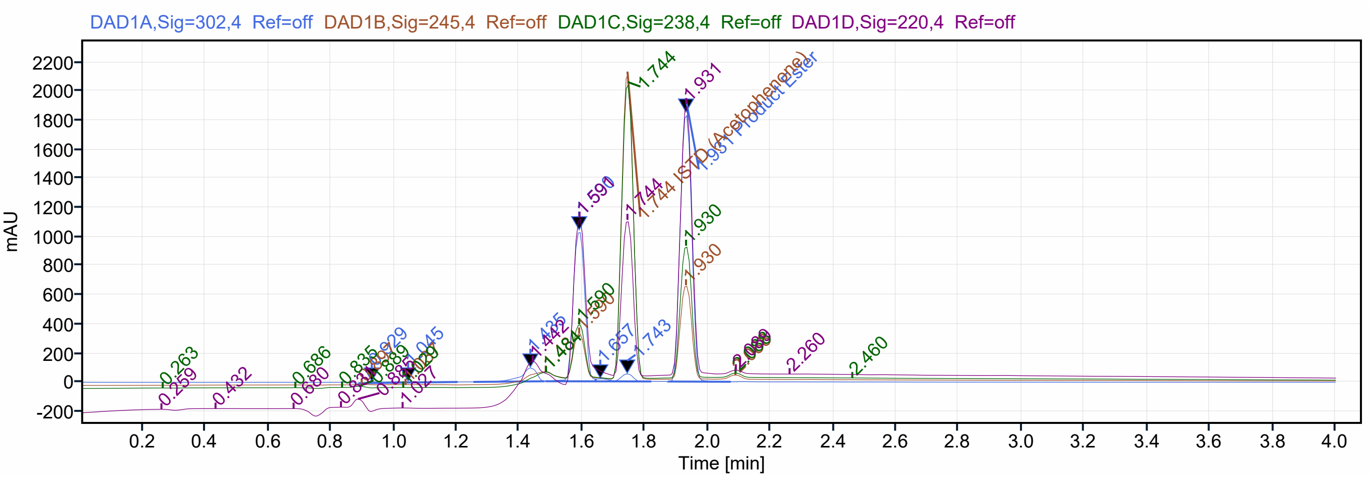

Sample Name	Name	RT (mins)	Area	Concentration (mg/L)
50 minutes	Umbelliferone			
50 minutes	Pivalic Anhydride			
50 minutes	DMAP			
50 minutes	ISTD (Acetophenone)	1.744	5391.3118	
50 minutes	Product Ester	1.931	4663.1628	

Injection Report - By Sample

Kineticolor

Sample name: 55 minutes

Data file: 2024-06-20 14-53-59+01-00-17.dx

Operator: SYSTEM

Instrument: 1220 Infinity II HPLC

Injection date: 2024-06-20 14:54:52+01:00

Inj. volume: 5.000 µL

Location: 15

Acq. method: Barry's standard method_low flow_higher A.amx

Type: Sample

Processing method: HB Standard method.pmx

Manually modified: None

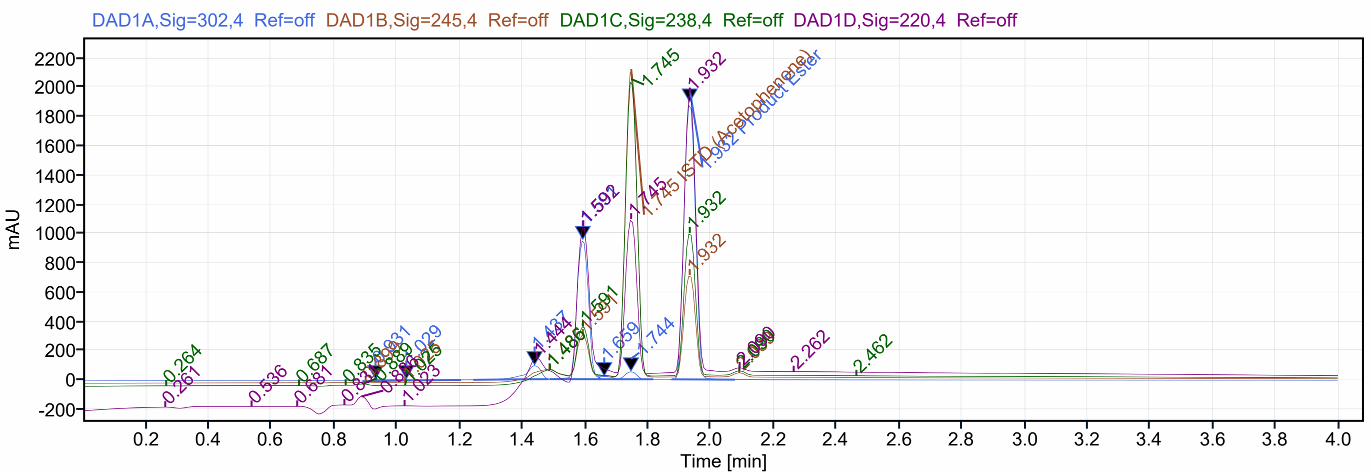

Sample Name	Name	RT (mins)	Area	Concentration (mg/L)
55 minutes	Umbelliferone			
55 minutes	Pivalic Anhydride			
55 minutes	DMAP			
55 minutes	ISTD (Acetophenone)	1.745	5359.9442	
55 minutes	Product Ester	1.932	4785.6305	

Injection Report - By Sample

Kineticolor

Sample name: 60 minutes

Data file: 2024-06-20 14-58-56+01-00-18.dx

Operator: SYSTEM

Instrument: 1220 Infinity II HPLC

Injection date: 2024-06-20 14:59:52+01:00

Inj. volume: 5.000 µL

Location: 16

Acq. method: Barry's standard method_low flow_higher A.amx

Type: Sample

Processing method: HB Standard method.pmx

Manually modified: None

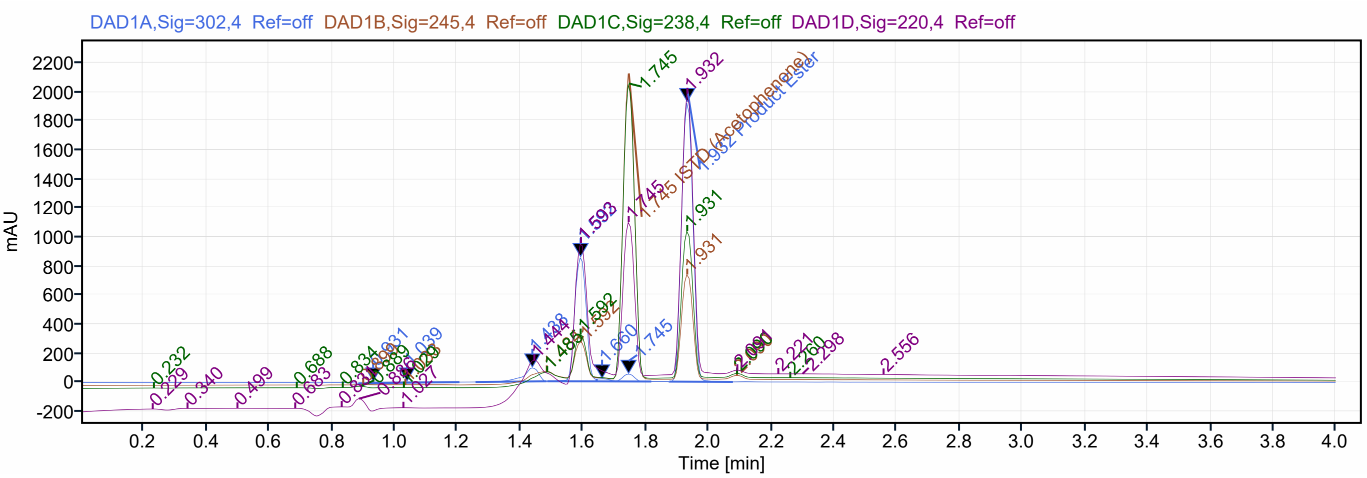

Sample Name	Name	RT (mins)	Area	Concentration (mg/L)
60 minutes	Umbelliferone			
60 minutes	Pivalic Anhydride			
60 minutes	DMAP			
60 minutes	ISTD (Acetophenone)	1.745	5366.8565	
60 minutes	Product Ester	1.932	4874.1952	

Injection Report - By Sample

Kineticolor

**Sample name:**	Blank		
**Data file:**	2024-06-20 15-03-56+01-00-19.dx	**Operator:**	SYSTEM
**Instrument:**	1220 Infinity II HPLC	**Injection date:**	2024-06-20 15:04:50+01:00
**Inj. volume:**	5.000 µL	**Location:**	31
**Acq. method:**	Barry's standard method_low flow_higher A.amx	**Type:**	Sample
**Processing method:**	HB Standard method.pmx		
**Manually modified:**	None		

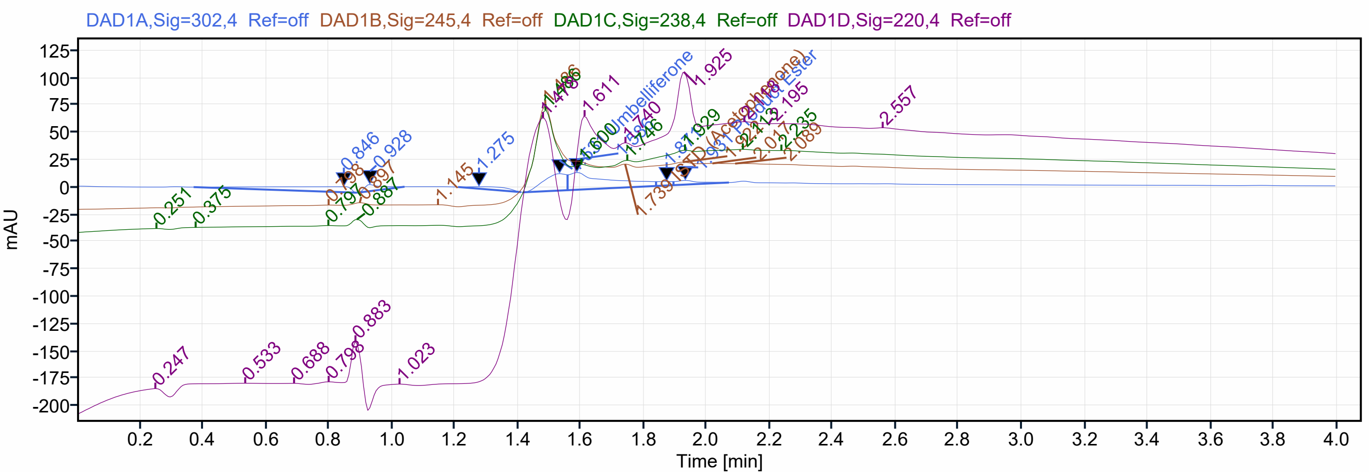

Sample Name	Name	RT (mins)	Area	Concentration (mg/L)
Blank	Pivalic Anhydride			
Blank	DMAP			
Blank	Umbelliferone	1.531	76.8722	
Blank	ISTD (Acetophenone)	1.739	110.5823	
Blank	Product Ester	1.931	19.8669	
