# Supplementary material for: Parallel and High Throughput Reaction Monitoring with Computer Vision
Source: Angew Chem Int Ed Engl. 2024 Oct 31;64(1):e202413395. doi: 10.1002/anie.202413395 (PMC11701362; doi:10.1002/anie.202413395)
Supplement: Supplementary file 3 — Supporting Information [file ANIE-64-e202413395-s003.zip › Supporting Info - Machine readable data part 2/Figure 10 - esterification and mutual information/HPLC_/Ester kinetics 3.pdf]

Injection Report - By Sample

Kineticolor

**Sample name:**	Blank		
**Data file:**	2024-06-20 15-24-30+01-00-01.dx	**Operator:**	SYSTEM
**Instrument:**	1220 Infinity II HPLC	**Injection date:**	2024-06-20 15:25:56+01:00
**Inj. volume:**	5.000 µL	**Location:**	31
**Acq. method:**	Barry's standard method_low flow_higher A.amx	**Type:**	Sample
**Processing method:**	HB Standard method.pmx		
**Manually modified:**	None		

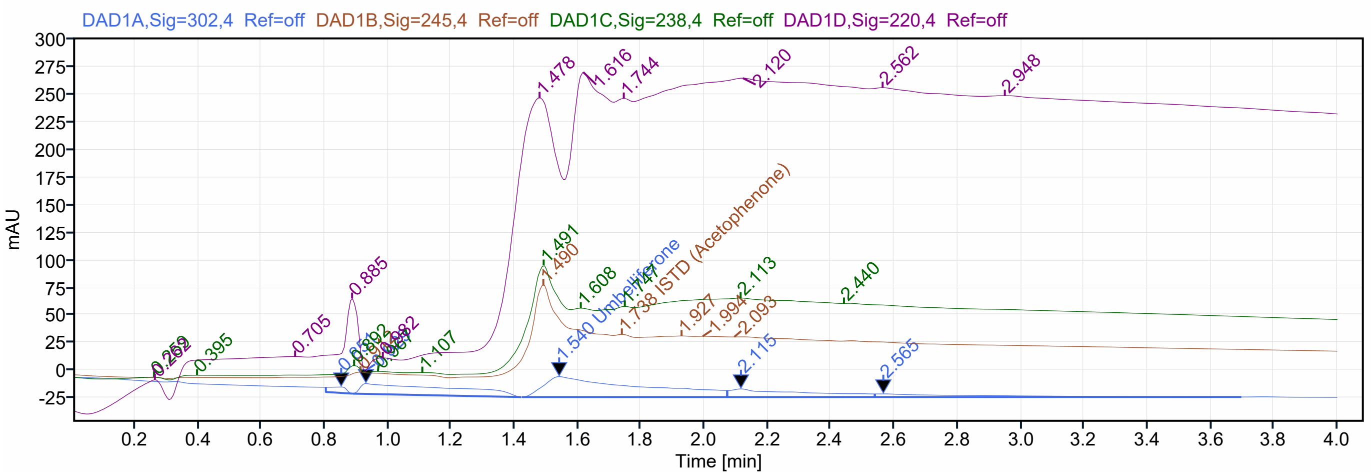

Sample Name	Name	RT (mins)	Area	Concentration (mg/L)
Blank	Product Ester			
Blank	Pivalic Anhydride			
Blank	DMAP			
Blank	Umbelliferone	1.540	388.1960	
Blank	ISTD (Acetophenone)	1.738	111.2018	

**Sample name:**	3 minutes
**Data file:**	2024-06-20 15-30-00+01-00-02.dx
**Operator:**	SYSTEM
**Instrument:**	1220 Infinity II HPLC
**Injection date:**	2024-06-20 15:30:53+01:00
**Inj. volume:**	5.000 µL
**Location:**	1
**Acq. method:**	Barry's standard method_low flow_higher A.amx
**Type:**	Sample
**Processing method:**	HB Standard method.pmx
**Manually modified:**	None

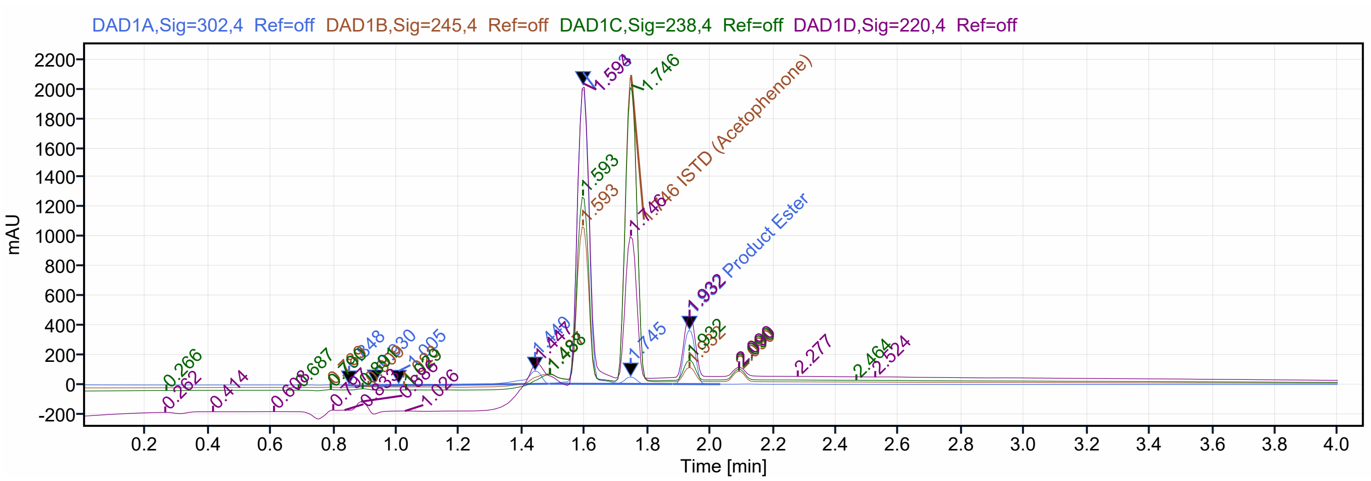

Sample Name	Name	RT (mins)	Area	Concentration (mg/L)
3 minutes	Umbelliferone			
3 minutes	Pivalic Anhydride			
3 minutes	DMAP			
3 minutes	ISTD (Acetophenone)	1.746	5267.4141	
3 minutes	Product Ester	1.932	907.7428	

Sample name: 6 minutes

Data file: 2024-06-20 15-34-57+01-00-03.dx

Operator: SYSTEM

Instrument: 1220 Infinity II HPLC

Injection date: 2024-06-20 15:35:51+01:00

Inj. volume: 5.000 µL

Location: 2

Acq. method: Barry's standard method_low flow_higher A.amx

Type: Sample

Processing method: HB Standard method.pmx

Manually modified: None

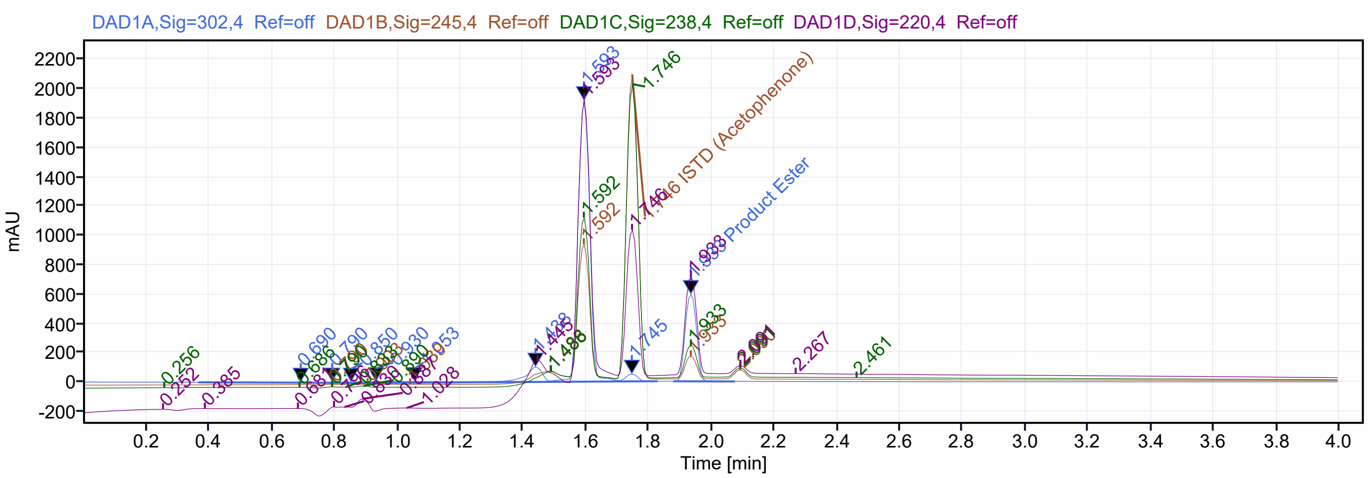

Sample Name	Name	RT (mins)	Area	Concentration (mg/L)
6 minutes	Umbelliferone			
6 minutes	Pivalic Anhydride			
6 minutes	DMAP			
6 minutes	ISTD (Acetophenone)	1.746	5284.2894	
6 minutes	Product Ester	1.933	1477.8582	

Injection Report - By Sample **Kineticolor**

Sample name: 9 minutes

Data file: 2024-06-20 15-39-55+01-00-04.dx

Operator: SYSTEM

Instrument: 1220 Infinity II HPLC

Injection date: 2024-06-20 15:40:49+01:00

Inj. volume: 5.000 µL

Location: 3

Acq. method: Barry's standard method_low flow_higher A.amx

Type: Sample

Processing method: HB Standard method.pmx

Manually modified: None

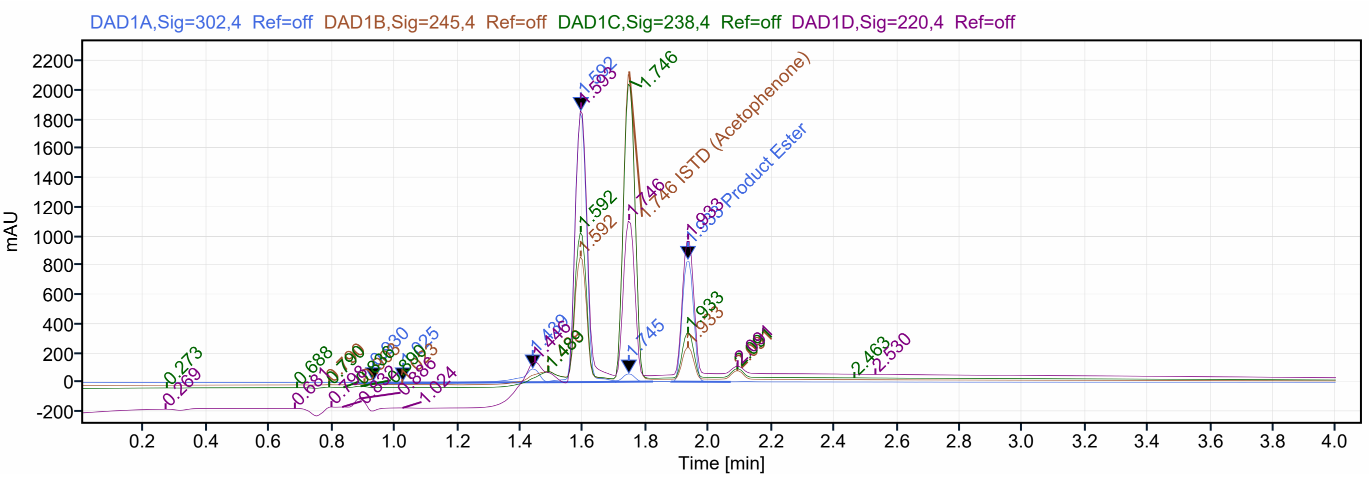

Sample Name	Name	RT (mins)	Area	Concentration (mg/L)
9 minutes	Umbelliferone			
9 minutes	Pivalic Anhydride			
9 minutes	DMAP			
9 minutes	ISTD (Acetophenone)	1.746	5378.4899	
9 minutes	Product Ester	1.933	2074.9555	

Sample name: 12 minutes

Data file: 2024-06-20 15-44-52+01-00-05.dx

Operator: SYSTEM

Instrument: 1220 Infinity II HPLC

Injection date: 2024-06-20 15:45:46+01:00

Inj. volume: 5.000 µL

Location: 4

Acq. method: Barry's standard method_low flow_higher A.amx

Type: Sample

Processing method: HB Standard method.pmx

Manually modified: None

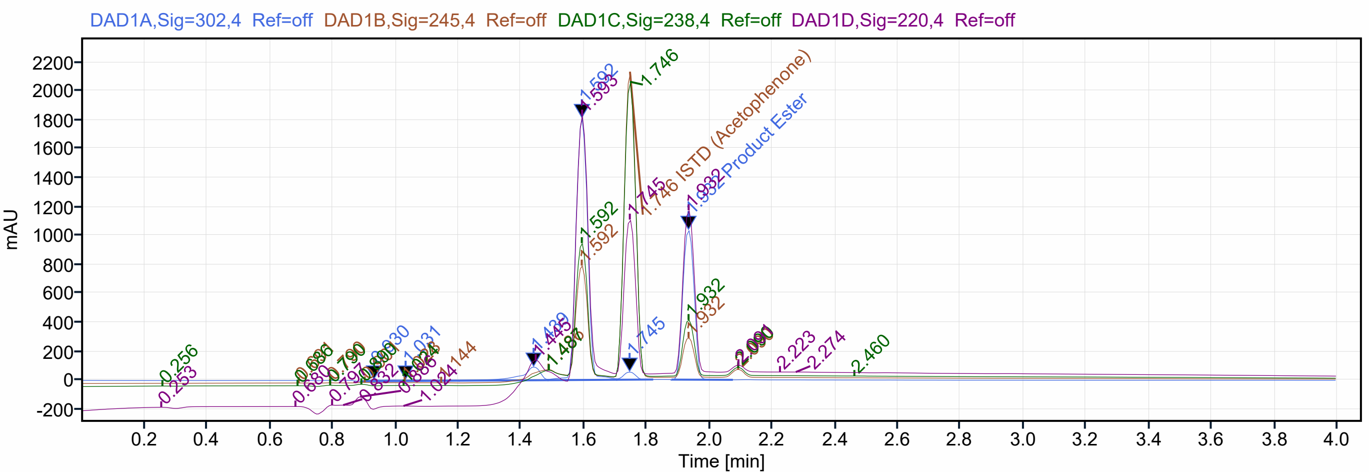

Sample Name	Name	RT (mins)	Area	Concentration (mg/L)
12 minutes	Umbelliferone			
12 minutes	Pivalic Anhydride			
12 minutes	DMAP			
12 minutes	ISTD (Acetophenone)	1.746	5387.5647	
12 minutes	Product Ester	1.932	2579.0278	

**Sample name:**	15 minutes		
**Data file:**	2024-06-20 15-49-50+01-00-06.dx	**Operator:**	SYSTEM
**Instrument:**	1220 Infinity II HPLC	**Injection date:**	2024-06-20 15:50:43+01:00
**Inj. volume:**	5.000 µL	**Location:**	5
**Acq. method:**	Barry's standard method_low flow_higher A.amx	**Type:**	Sample
**Processing method:**	HB Standard method.pmx		
**Manually modified:**	None		

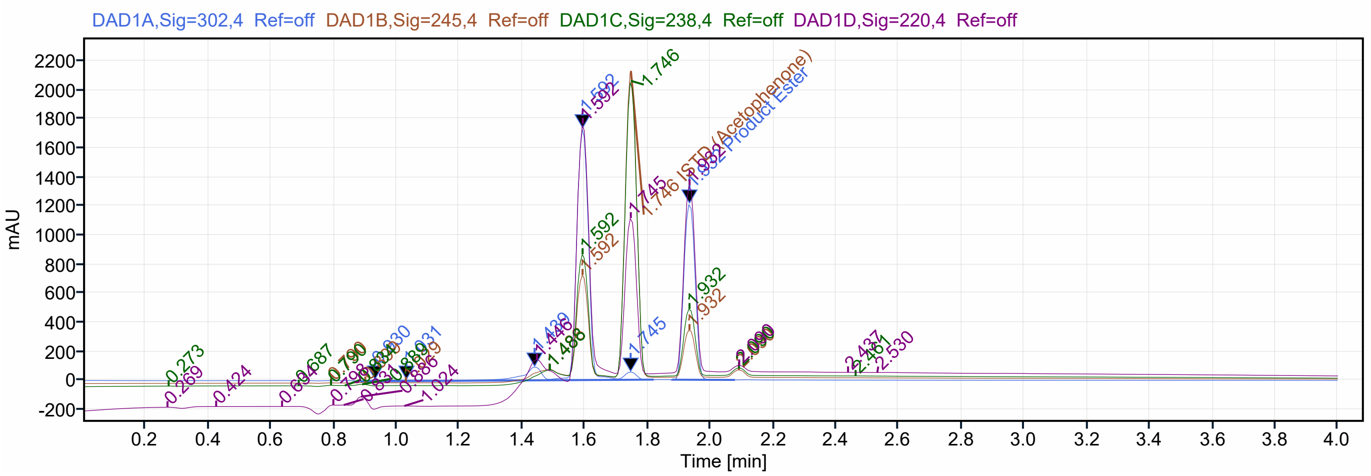

Sample Name	Name	RT (mins)	Area	Concentration (mg/L)
15 minutes	Umbelliferone			
15 minutes	Pivalic Anhydride			
15 minutes	DMAP			
15 minutes	ISTD (Acetophenone)	1.746	5372.6472	
15 minutes	Product Ester	1.932	3022.2689	

Sample name: 18 minutes

Data file: 2024-06-20 15-54-47+01-00-07.dx

Operator: SYSTEM

Instrument: 1220 Infinity II HPLC

Injection date: 2024-06-20 15:55:41+01:00

Inj. volume: 5.000 µL

Location: 6

Acq. method: Barry's standard method_low flow_higher A.amx

Type: Sample

Processing method: HB Standard method.pmx

Manually modified: None

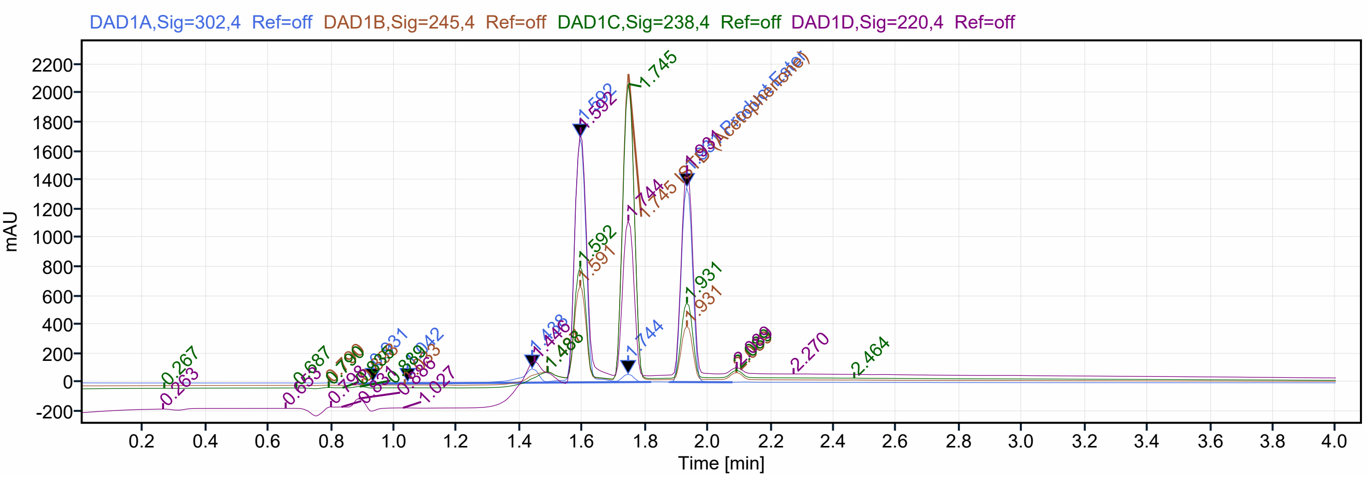

Sample Name	Name	RT (mins)	Area	Concentration (mg/L)
18 minutes	Umbelliferone			
18 minutes	Pivalic Anhydride			
18 minutes	DMAP			
18 minutes	ISTD (Acetophenone)	1.745	5385.6564	
18 minutes	Product Ester	1.931	3364.8540	

Injection Report - By Sample

Kineticolor

**Sample name:**	21 minutes		
**Data file:**	2024-06-20 15-59-44+01-00-08.dx	**Operator:**	SYSTEM
**Instrument:**	1220 Infinity II HPLC	**Injection date:**	2024-06-20 16:00:39+01:00
**Inj. volume:**	5.000 µL	**Location:**	7
**Acq. method:**	Barry's standard method_low flow_higher A.amx	**Type:**	Sample
**Processing method:**	HB Standard method.pmx		
**Manually modified:**	None		

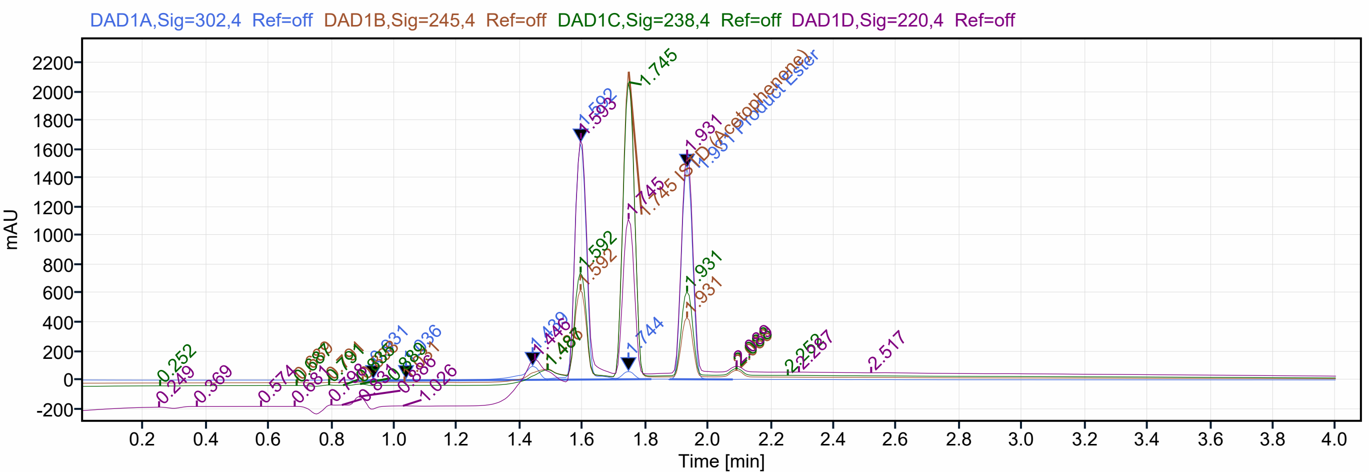

Sample Name	Name	RT (mins)	Area	Concentration (mg/L)
21 minutes	Umbelliferone			
21 minutes	Pivalic Anhydride			
21 minutes	DMAP			
21 minutes	ISTD (Acetophenone)	1.745	5407.6907	
21 minutes	Product Ester	1.931	3671.6495	

Injection Report - By Sample

**Sample name:**	24 minutes		
**Data file:**	2024-06-20 16-04-42+01-00-09.dx	**Operator:**	SYSTEM
**Instrument:**	1220 Infinity II HPLC	**Injection date:**	2024-06-20 16:05:37+01:00
**Inj. volume:**	5.000 µL	**Location:**	8
**Acq. method:**	Barry's standard method_low flow_higher A.amx	**Type:**	Sample
**Processing method:**	HB Standard method.pmx		
**Manually modified:**	None		

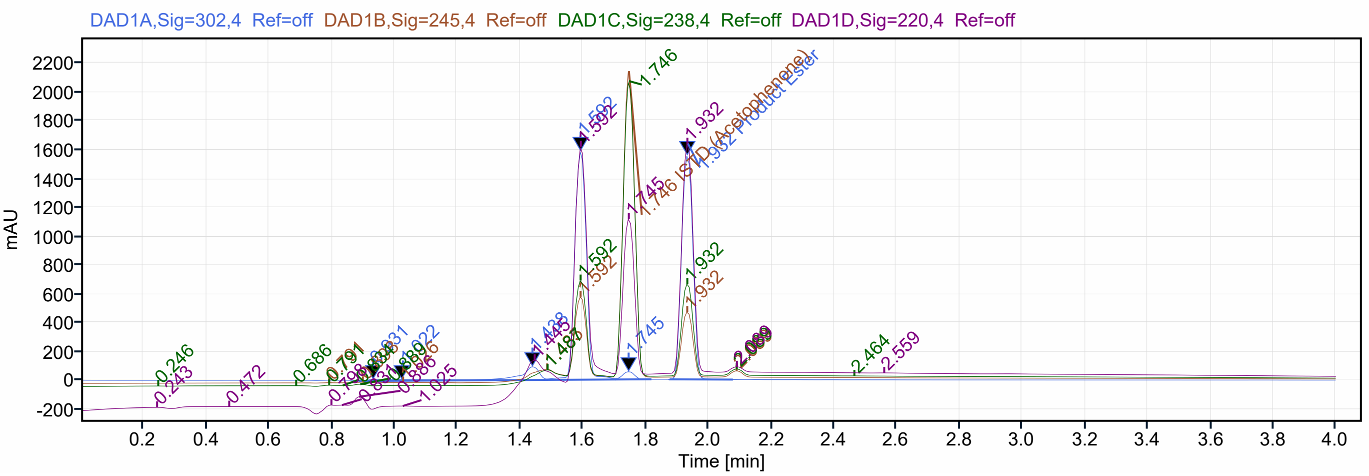

Sample Name	Name	RT (mins)	Area	Concentration (mg/L)
24 minutes	Umbelliferone			
24 minutes	Pivalic Anhydride			
24 minutes	DMAP			
24 minutes	ISTD (Acetophenone)	1.746	5426.4265	
24 minutes	Product Ester	1.932	3901.2833	

Injection Report - By Sample

Kineticolor

**Sample name:**	Blank
**Data file:**	2024-06-20 16-09-40+01-00-10.dx
**Operator:**	SYSTEM
**Instrument:**	1220 Infinity II HPLC
**Injection date:**	2024-06-20 16:10:35+01:00
**Inj. volume:**	5.000 µL
**Location:**	31
**Acq. method:**	Barry's standard method_low flow_higher A.amx
**Type:**	Sample
**Processing method:**	HB Standard method.pmx
**Manually modified:**	None

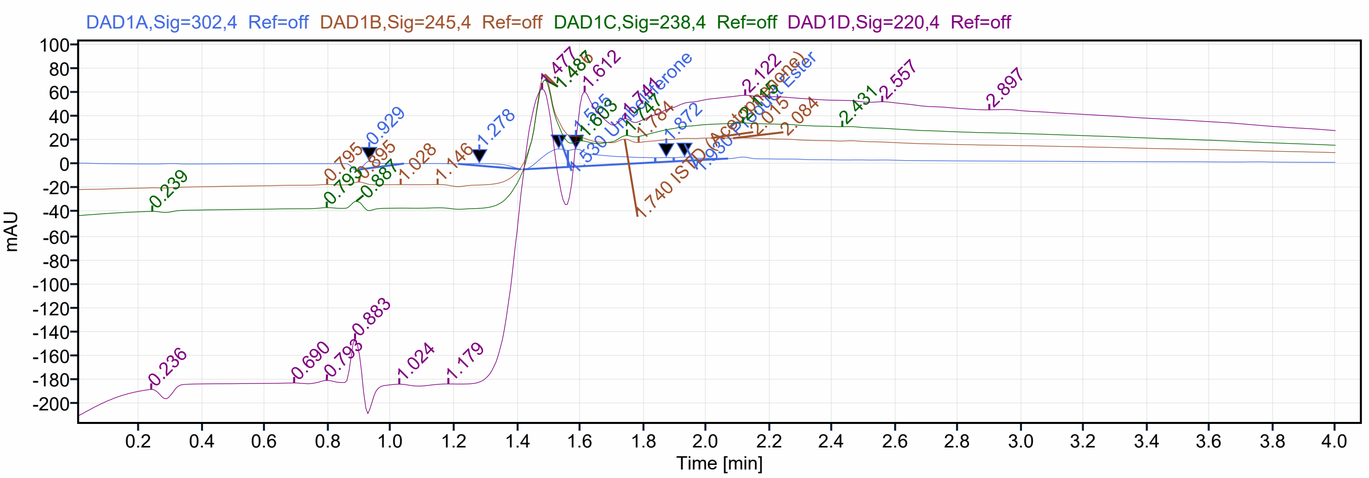

Sample Name	Name	RT (mins)	Area	Concentration (mg/L)
Blank	Pivalic Anhydride			
Blank	DMAP			
Blank	Umbelliferone	1.530	79.7113	
Blank	ISTD (Acetophenone)	1.740	111.5809	
Blank	Product Ester	1.930	17.4317	

Injection Report - By Sample

Kineticolor

**Sample name:**	27 minutes		
**Data file:**	2024-06-20 16-14-39+01-00-11.dx	**Operator:**	SYSTEM
**Instrument:**	1220 Infinity II HPLC	**Injection date:**	2024-06-20 16:15:36+01:00
**Inj. volume:**	5.000 µL	**Location:**	9
**Acq. method:**	Barry's standard method_low flow_higher A.amx	**Type:**	Sample
**Processing method:**	HB Standard method.pmx		
**Manually modified:**	None		

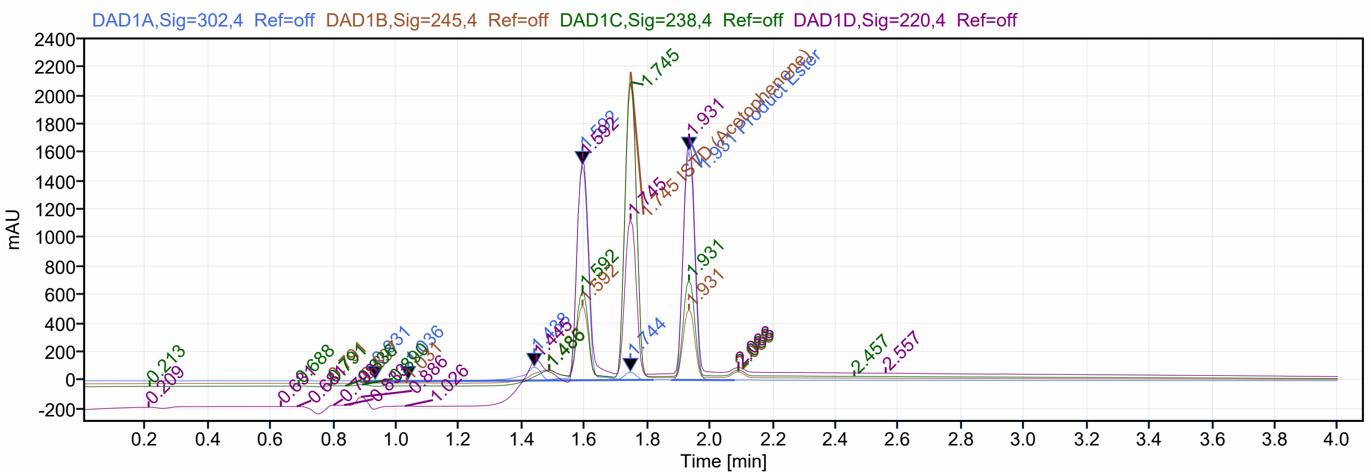

Sample Name	Name	RT (mins)	Area	Concentration (mg/L)
27 minutes	Umbelliferone			
27 minutes	Pivalic Anhydride			
27 minutes	DMAP			
27 minutes	ISTD (Acetophenone)	1.745	5473.8151	
27 minutes	Product Ester	1.931	4027.2613	

Injection Report - By Sample

Kineticolor

**Sample name:**	30 minutes		
**Data file:**	2024-06-20 16-19-39+01-00-12.dx	**Operator:**	SYSTEM
**Instrument:**	1220 Infinity II HPLC	**Injection date:**	2024-06-20 16:20:41+01:00
**Inj. volume:**	5.000 µL	**Location:**	10
**Acq. method:**	Barry's standard method_low flow_higher A.amx	**Type:**	Sample
**Processing method:**	HB Standard method.pmx		
**Manually modified:**	None		

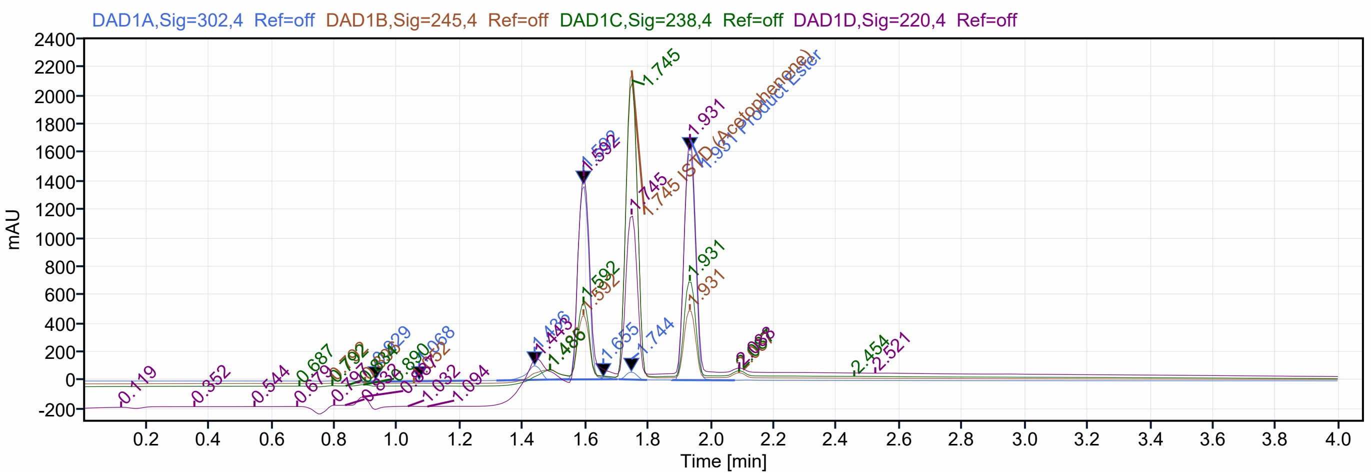

Sample Name	Name	RT (mins)	Area	Concentration (mg/L)
30 minutes	Umbelliferone			
30 minutes	Pivalic Anhydride			
30 minutes	DMAP			
30 minutes	ISTD (Acetophenone)	1.745	5508.8606	
30 minutes	Product Ester	1.931	4020.7533	

Sample name: 35 minutes

Data file: 2024-06-20 16-24-44+01-00-13.dx

Operator: SYSTEM

Instrument: 1220 Infinity II HPLC

Injection date: 2024-06-20 16:25:41+01:00

Inj. volume: 5.000 µL

Location: 11

Acq. method: Barry's standard method_low flow_higher A.amx

Type: Sample

Processing method: HB Standard method.pmx

Manually modified: None

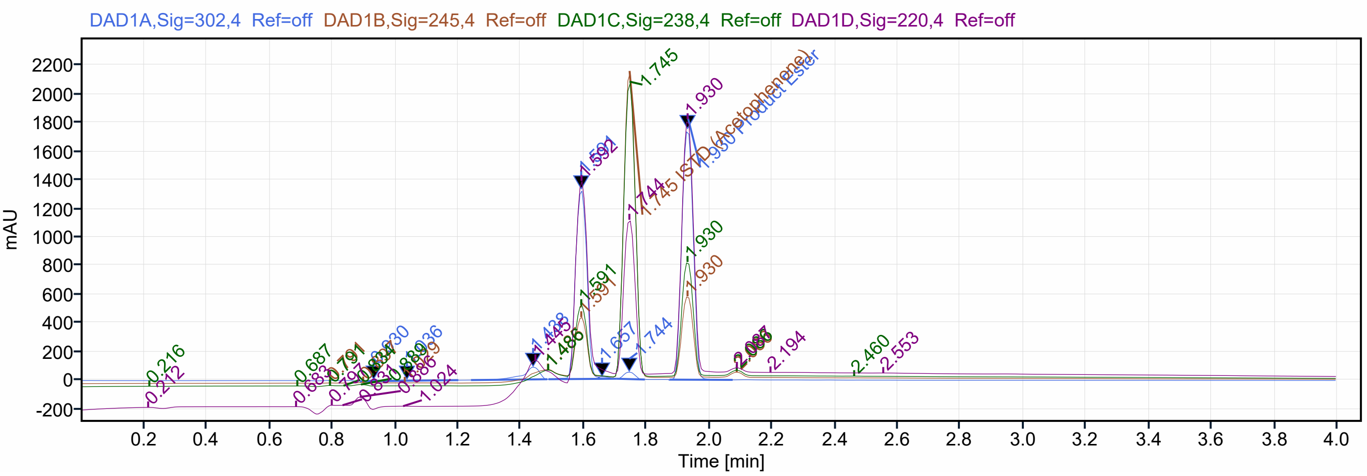

Sample Name	Name	RT (mins)	Area	Concentration (mg/L)
35 minutes	Umbelliferone			
35 minutes	Pivalic Anhydride			
35 minutes	DMAP			
35 minutes	ISTD (Acetophenone)	1.745	5458.6870	
35 minutes	Product Ester	1.930	4402.4937	

**Sample name:**	40 minutes		
**Data file:**	2024-06-20 16-29-45+01-00-14.dx	**Operator:**	SYSTEM
**Instrument:**	1220 Infinity II HPLC	**Injection date:**	2024-06-20 16:30:39+01:00
**Inj. volume:**	5.000 µL	**Location:**	12
**Acq. method:**	Barry's standard method_low flow_higher A.amx	**Type:**	Sample
**Processing method:**	HB Standard method.pmx		
**Manually modified:**	None		

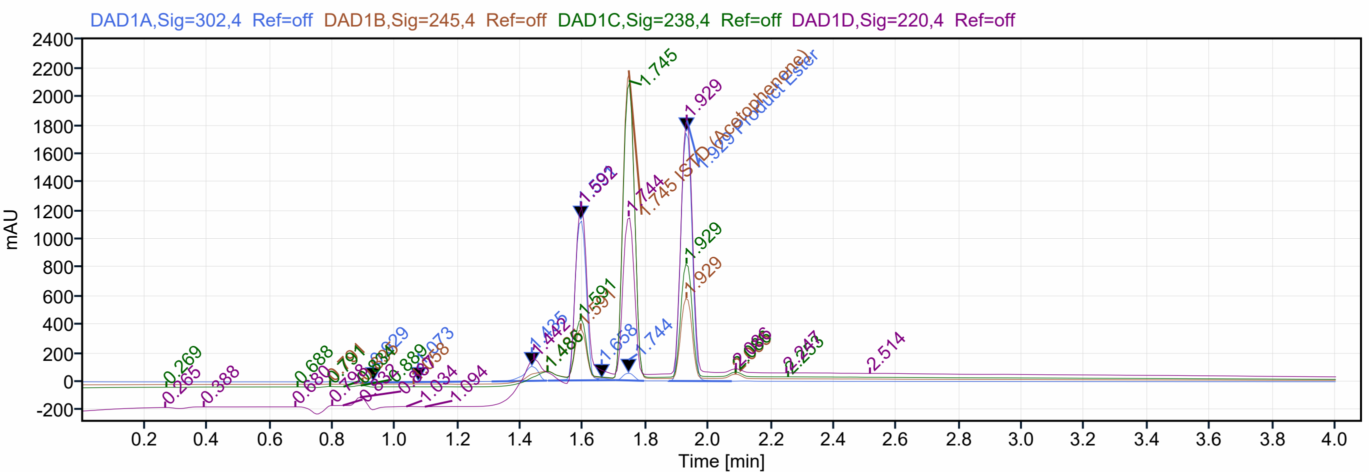

Sample Name	Name	RT (mins)	Area	Concentration (mg/L)
40 minutes	Umbelliferone			
40 minutes	Pivalic Anhydride			
40 minutes	DMAP			
40 minutes	ISTD (Acetophenone)	1.745	5529.5701	
40 minutes	Product Ester	1.929	4406.0442	

Sample name: 45 minutes

Data file: 2024-06-20 16-34-43+01-00-15.dx

Operator: SYSTEM

Instrument: 1220 Infinity II HPLC

Injection date: 2024-06-20 16:35:37+01:00

Inj. volume: 5.000 µL

Location: 13

Acq. method: Barry's standard method_low flow_higher A.amx

Type: Sample

Processing method: HB Standard method.pmx

Manually modified: None

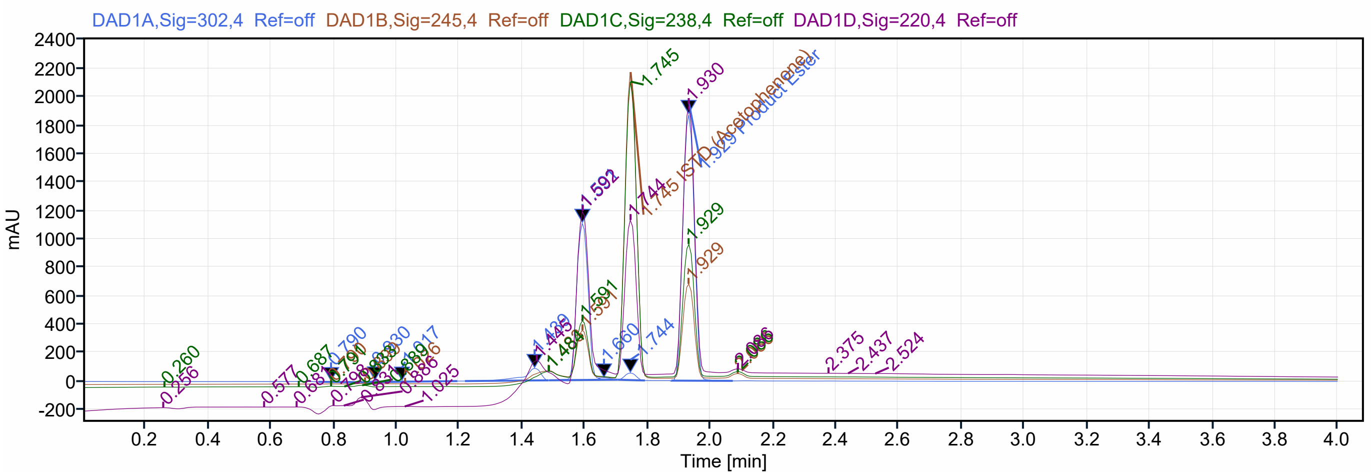

Sample Name	Name	RT (mins)	Area	Concentration (mg/L)
45 minutes	Umbelliferone			
45 minutes	Pivalic Anhydride			
45 minutes	DMAP			
45 minutes	ISTD (Acetophenone)	1.745	5493.1163	
45 minutes	Product Ester	1.929	4733.9502	

Injection Report - By Sample

Kineticolor

Sample name: 50 minutes

Data file: 2024-06-20 16-39-40+01-00-16.dx

Operator: SYSTEM

Instrument: 1220 Infinity II HPLC

Injection date: 2024-06-20 16:40:34+01:00

Inj. volume: 5.000 µL

Location: 14

Acq. method: Barry's standard method_low flow_higher A.amx

Type: Sample

Processing method: HB Standard method.pmx

Manually modified: None

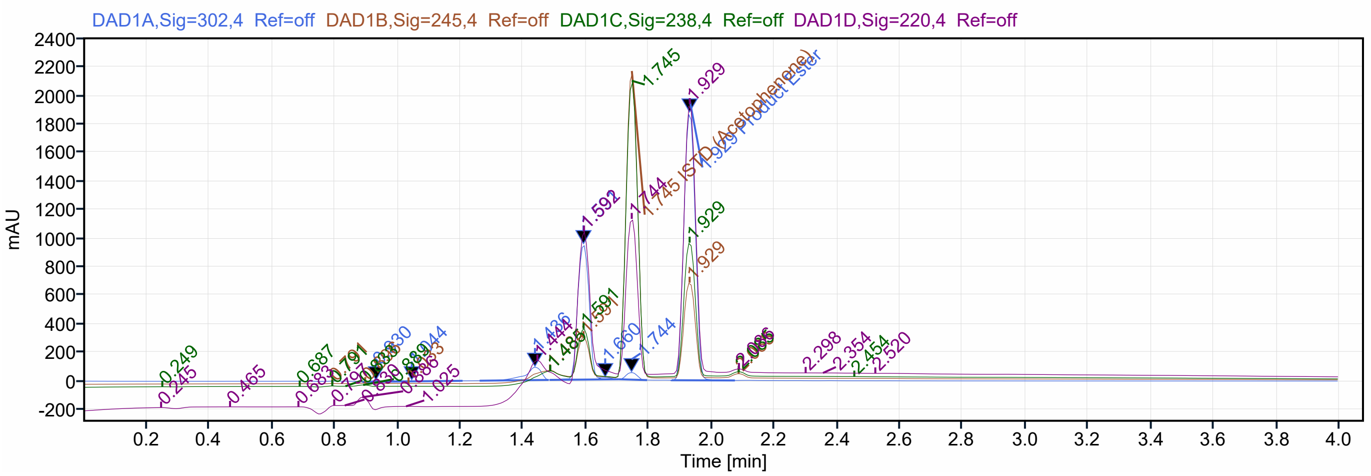

Sample Name	Name	RT (mins)	Area	Concentration (mg/L)
50 minutes	Umbelliferone			
50 minutes	Pivalic Anhydride			
50 minutes	DMAP			
50 minutes	ISTD (Acetophenone)	1.745	5495.7101	
50 minutes	Product Ester	1.929	4745.2845	

Injection Report - By Sample

Kineticolor

**Sample name:**	55 minutes		
**Data file:**	2024-06-20 16-44-38+01-00-17.dx	**Operator:**	SYSTEM
**Instrument:**	1220 Infinity II HPLC	**Injection date:**	2024-06-20 16:45:32+01:00
**Inj. volume:**	5.000 µL	**Location:**	15
**Acq. method:**	Barry's standard method_low flow_higher A.amx	**Type:**	Sample
**Processing method:**	HB Standard method.pmx		
**Manually modified:**	None		

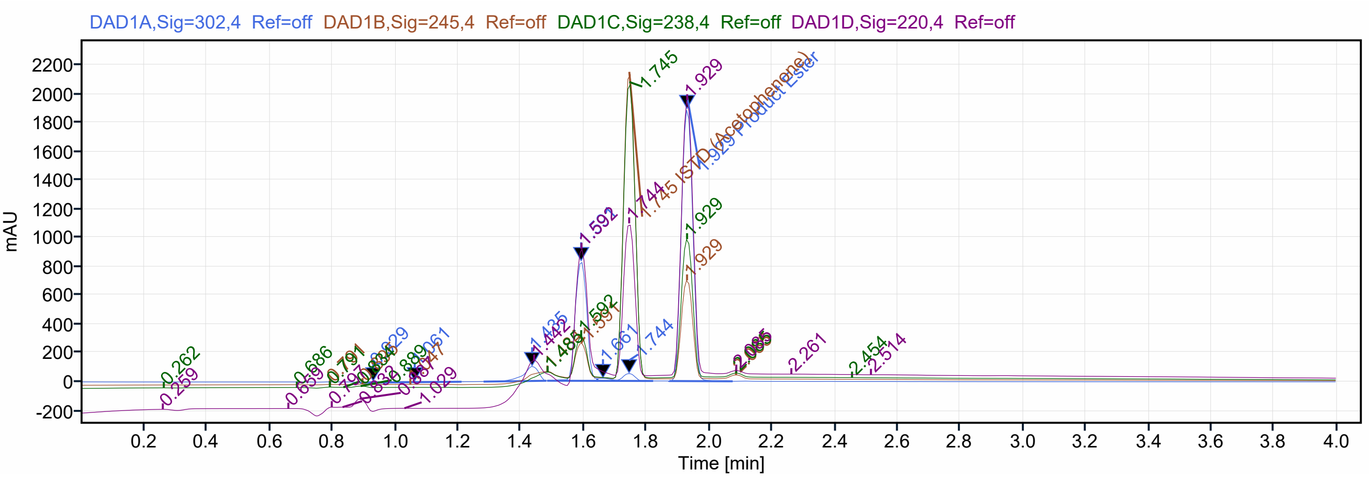

Sample Name	Name	RT (mins)	Area	Concentration (mg/L)
55 minutes	Umbelliferone			
55 minutes	Pivalic Anhydride			
55 minutes	DMAP			
55 minutes	ISTD (Acetophenone)	1.745	5435.1609	
55 minutes	Product Ester	1.929	4773.8017	

Injection Report - By Sample

Kineticolor

Sample name: 60 minutes

Data file: 2024-06-20 16-49-35+01-00-18.dx

Operator: SYSTEM

Instrument: 1220 Infinity II HPLC

Injection date: 2024-06-20 16:50:29+01:00

Inj. volume: 5.000 µL

Location: 16

Acq. method: Barry's standard method_low flow_higher A.amx

Type: Sample

Processing method: HB Standard method.pmx

Manually modified: None

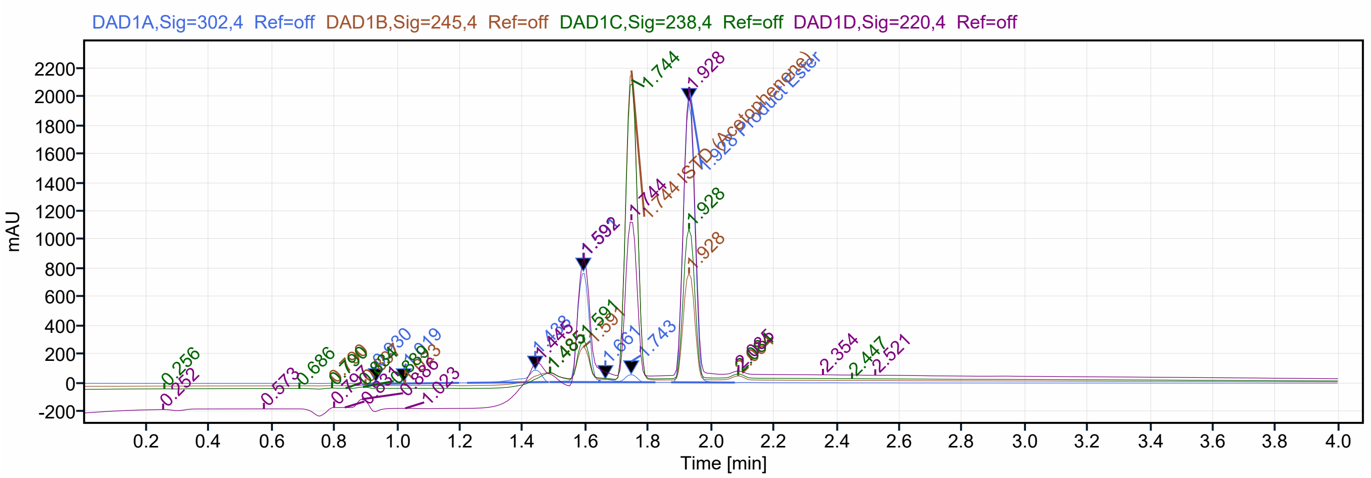

Sample Name	Name	RT (mins)	Area	Concentration (mg/L)
60 minutes	Umbelliferone			
60 minutes	Pivalic Anhydride			
60 minutes	DMAP			
60 minutes	ISTD (Acetophenone)	1.744	5530.4663	
60 minutes	Product Ester	1.928	4968.2676	

**Sample name:**	Blank		
**Data file:**	2024-06-20 16-54-33+01-00-19.dx	**Operator:**	SYSTEM
**Instrument:**	1220 Infinity II HPLC	**Injection date:**	2024-06-20 16:55:27+01:00
**Inj. volume:**	5.000 µL	**Location:**	31
**Acq. method:**	Barry's standard method_low flow_higher A.amx	**Type:**	Sample
**Processing method:**	HB Standard method.pmx		
**Manually modified:**	None		

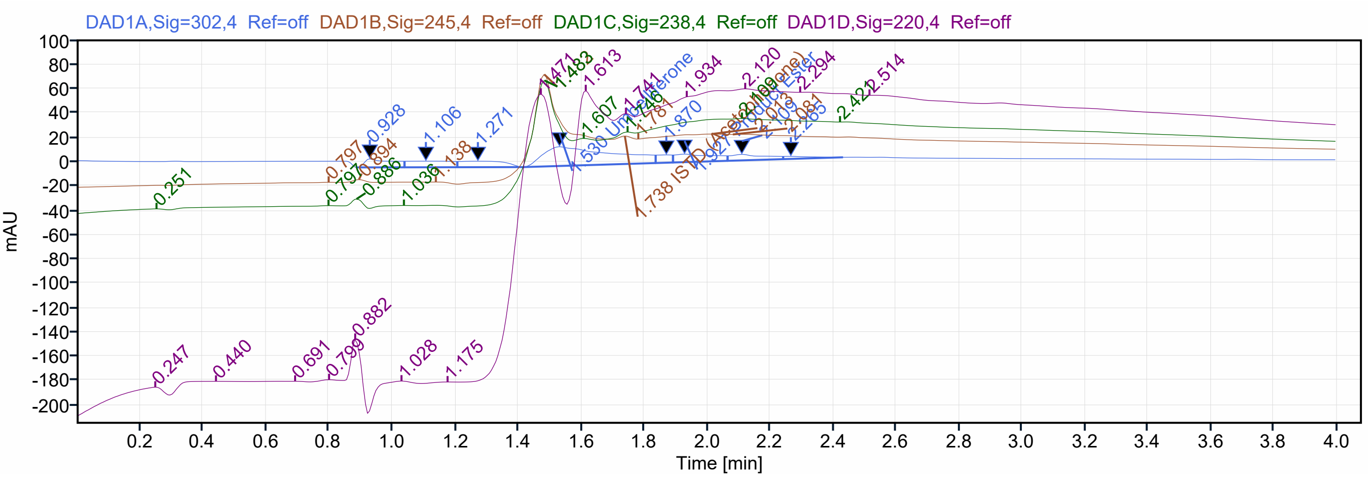

Sample Name	Name	RT (mins)	Area	Concentration (mg/L)
Blank	Pivalic Anhydride			
Blank	DMAP			
Blank	Umbelliferone	1.530	241.5600	
Blank	ISTD (Acetophenone)	1.738	110.0730	
Blank	Product Ester	1.927	55.4177	
