# Supplementary material for: Parallel and High Throughput Reaction Monitoring with Computer Vision
Source: Angew Chem Int Ed Engl. 2024 Oct 31;64(1):e202413395. doi: 10.1002/anie.202413395 (PMC11701362; doi:10.1002/anie.202413395)
Supplement: Supplementary file 3 — Supporting Information [file ANIE-64-e202413395-s003.zip › Supporting Info - Machine readable data part 2/Figure 10 - esterification and mutual information/HPLC_/Ester kinetics 1.pdf]

# *Injection Report - By Sample*

**Kineticolor**

| | | | |
|---|---|---|---|
| **Sample name:** | Blank | | |
| **Data file:** | 2024-06-05 13-14-40+01-00-01.dx | **Operator:** | SYSTEM |
| **Instrument:** | 1220 Infinity II HPLC | **Injection date:** | 2024-06-05 13:16:08+01:00 |
| **Inj. volume:** | 5.000 µL | **Location:** | 41 |
| **Acq. method:** | Barry's standard method_low flow_higher A.amx | **Type:** | Sample |
| **Processing method:** | *HB Standard method.pmx | | |
| **Manually modified:** | None | | |

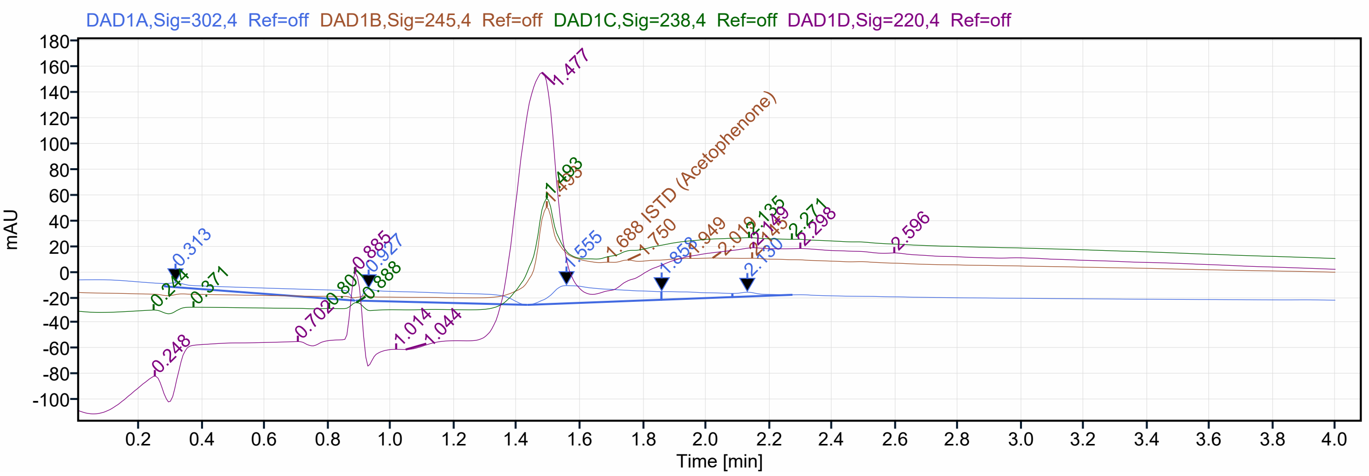


| Sample Name | Name | RT (mins) | Area | Concentration (mg/L) |
|---|---|---|---|---|
| Blank | Umbelliferone | | | |
| Blank | DMAP | | | |
| Blank | Pivalic Anhydride | | | |
| Blank | Product Ester | | | |
| Blank | ISTD (Acetophenone) | 1.688 | 14.8108 | |

**Sample name:** 3 minutes

**Data file:** 2024-06-05 13-20-11+01-00-02.dx

**Operator:** SYSTEM

**Instrument:** 1220 Infinity II HPLC

**Injection date:** 2024-06-05 13:21:04+01:00

**Inj. volume:** 5.000 µL

**Location:** 1

**Acq. method:** Barry's standard method_low flow_higher A.amx

**Type:** Sample

**Processing method:** *HB Standard method.pmx

**Manually modified:** Manual CompoundID

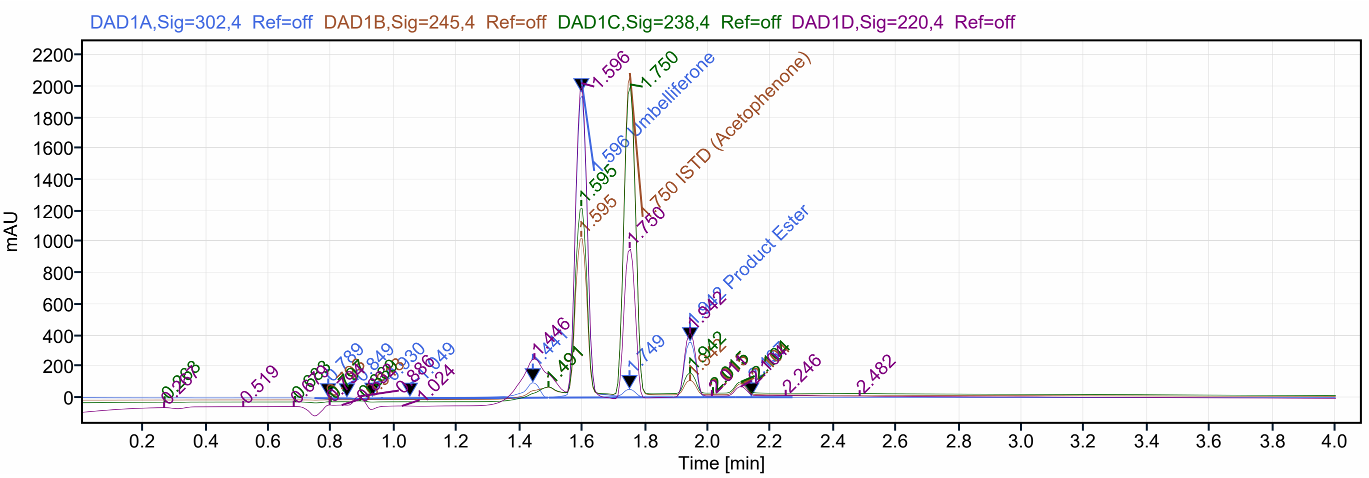


| Sample Name | Name | RT (mins) | Area | Concentration (mg/L) |
|---|---|---|---|---|
| 3 minutes | Pivalic Anhydride | | | |
| 3 minutes | DMAP | | | |
| 3 minutes | Umbelliferone | 1.596 | 5050.2775 | |
| 3 minutes | ISTD (Acetophenone) | 1.750 | 5252.9808 | |
| 3 minutes | Product Ester | 1.942 | 900.2172 | |

# *Injection Report - By Sample*

*Kineticolor*

**Sample name:** 6 minutes

**Data file:** 2024-06-05 13-25-09+01-00-03.dx

**Operator:** SYSTEM

**Instrument:** 1220 Infinity II HPLC

**Injection date:** 2024-06-05 13:26:02+01:00

**Inj. volume:** 5.000 µL

**Location:** 2

**Acq. method:** Barry's standard method_low flow_higher A.amx

**Type:** Sample

**Processing method:** *HB Standard method.pmx

**Manually modified:** None

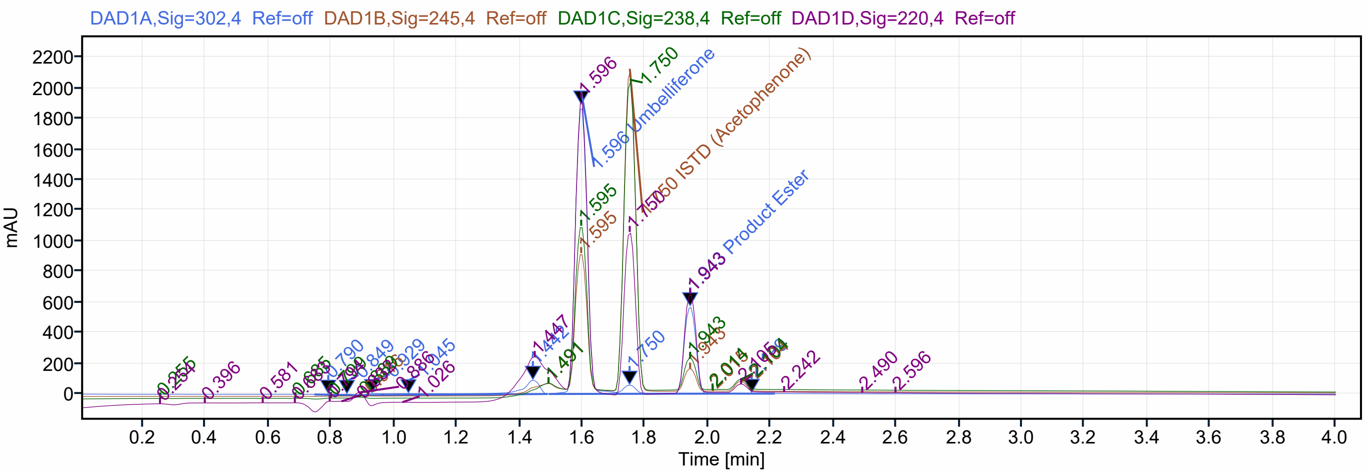


| Sample Name | Name | RT (mins) | Area | Concentration (mg/L) |
|---|---|---|---|---|
| 6 minutes | Pivalic Anhydride | | | |
| 6 minutes | DMAP | | | |
| 6 minutes | Umbelliferone | 1.596 | 4845.0618 | |
| 6 minutes | ISTD (Acetophenone) | 1.750 | 5367.7260 | |
| 6 minutes | Product Ester | 1.943 | 1427.5802 | |

# Injection Report - By Sample

Kineticolor

**Sample name:** 9 minutes

**Data file:** 2024-06-05 13-30-06+01-00-04.dx

**Operator:** SYSTEM

**Instrument:** 1220 Infinity II HPLC

**Injection date:** 2024-06-05 13:31:00+01:00

**Inj. volume:** 5.000 µL

**Location:** 3

**Acq. method:** Barry's standard method_low flow_higher A.amx

**Type:** Sample

**Processing method:** *HB Standard method.pmx

**Manually modified:** None

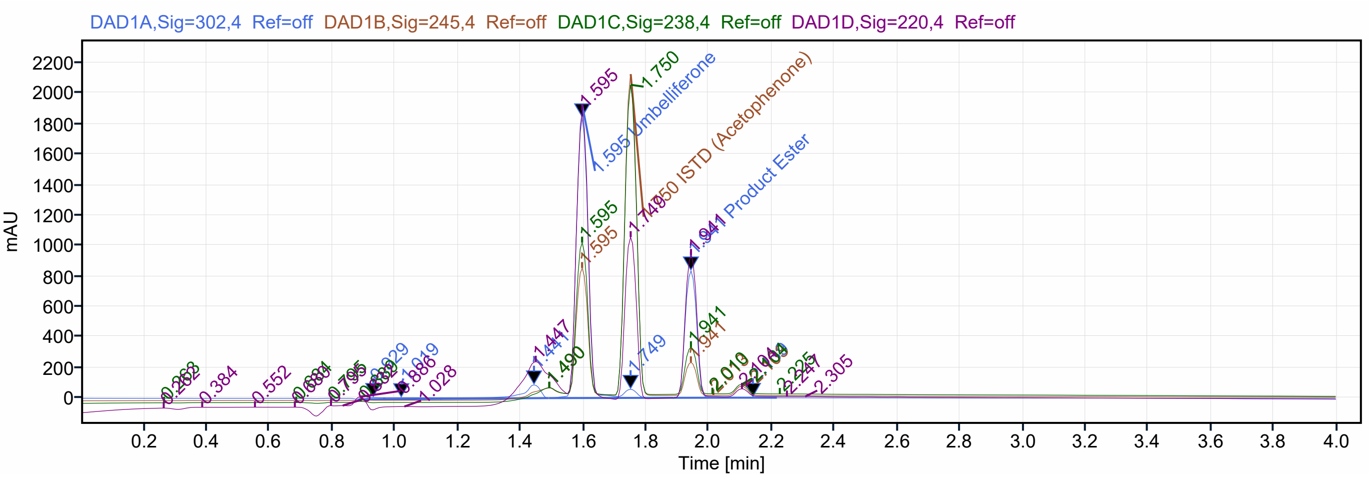


| Sample Name | Name | RT (mins) | Area | Concentration (mg/L) |
|---|---|---|---|---|
| 9 minutes | Pivalic Anhydride | | | |
| 9 minutes | DMAP | | | |
| 9 minutes | Umbelliferone | 1.595 | 4715.2534 | |
| 9 minutes | ISTD (Acetophenone) | 1.750 | 5373.4322 | |
| 9 minutes | Product Ester | 1.941 | 2091.9160 | |

**Sample name:** 12 minutes

**Data file:** 2024-06-05 13-35-04+01-00-05.dx

**Operator:** SYSTEM

**Instrument:** 1220 Infinity II HPLC

**Injection date:** 2024-06-05 13:35:58+01:00

**Inj. volume:** 5.000 µL

**Location:** 4

**Acq. method:** Barry's standard method_low flow_higher A.amx

**Type:** Sample

**Processing method:** *HB Standard method.pmx

**Manually modified:** None

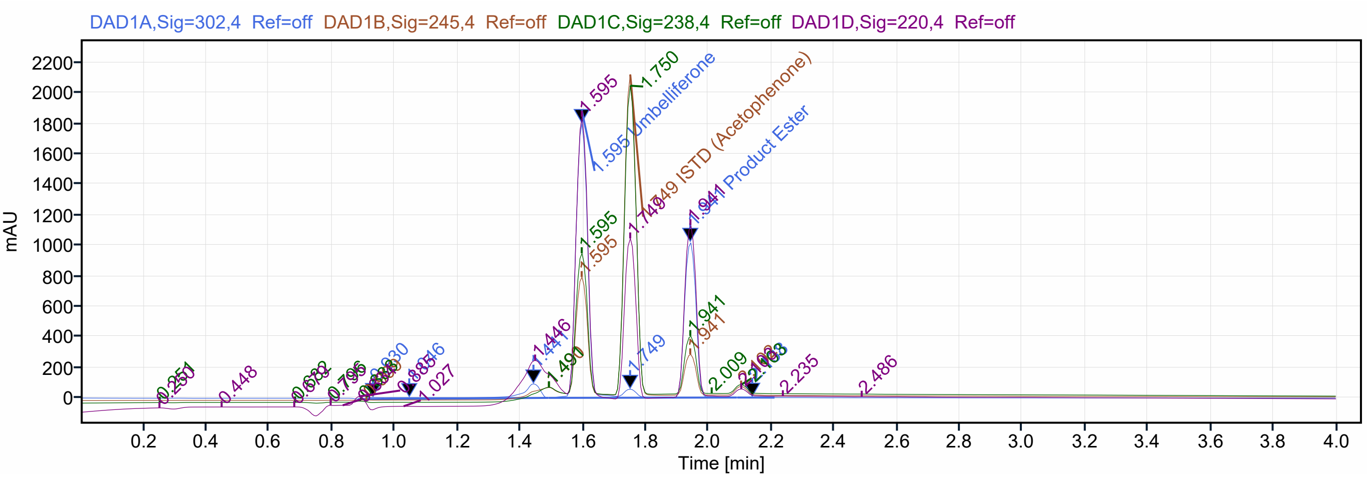


| Sample Name | Name | RT (mins) | Area | Concentration (mg/L) |
|---|---|---|---|---|
| 12 minutes | Pivalic Anhydride | | | |
| 12 minutes | DMAP | | | |
| 12 minutes | Umbelliferone | 1.595 | 4600.5823 | |
| 12 minutes | ISTD (Acetophenone) | 1.749 | 5356.1163 | |
| 12 minutes | Product Ester | 1.941 | 2555.7759 | |

| | |
|---|---|
| **Sample name:** | 15 minutes |
| **Data file:** | 2024-06-05 13-40-03+01-00-06.dx |
| **Operator:** | SYSTEM |
| **Instrument:** | 1220 Infinity II HPLC |
| **Injection date:** | 2024-06-05 13:40:56+01:00 |
| **Inj. volume:** | 5.000 µL |
| **Location:** | 5 |
| **Acq. method:** | Barry's standard method_low flow_higher A.amx |
| **Type:** | Sample |
| **Processing method:** | *HB Standard method.pmx |
| **Manually modified:** | None |

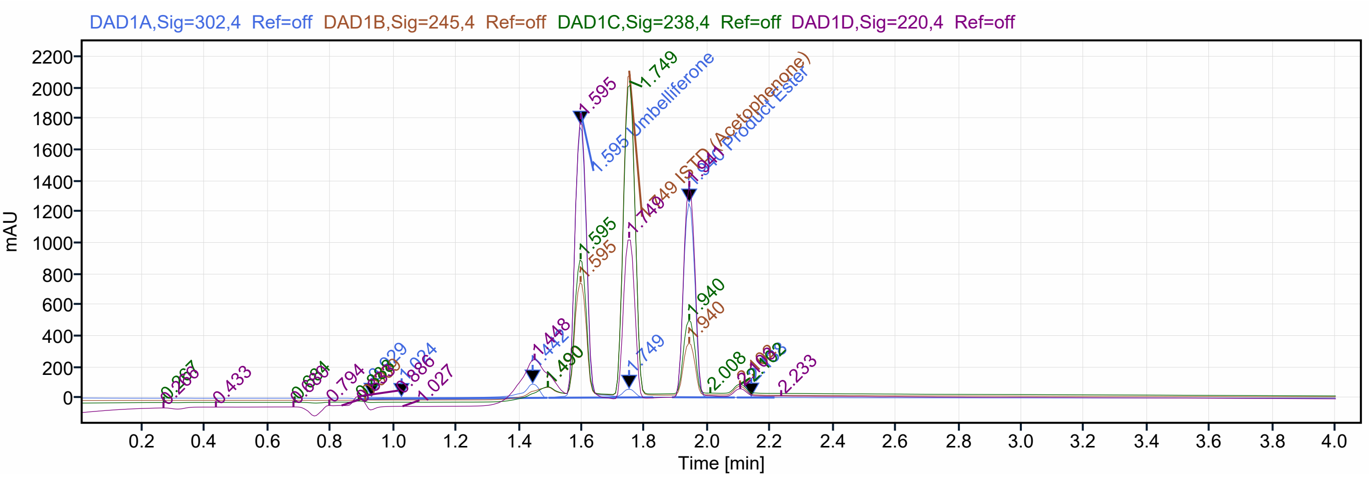


| Sample Name | Name | RT (mins) | Area | Concentration (mg/L) |
|---|---|---|---|---|
| 15 minutes | Pivalic Anhydride | | | |
| 15 minutes | DMAP | | | |
| 15 minutes | Umbelliferone | 1.595 | 4480.8733 | |
| 15 minutes | ISTD (Acetophenone) | 1.749 | 5326.7334 | |
| 15 minutes | Product Ester | 1.940 | 3133.9023 | |

# *Injection Report - By Sample*

**Kineticolor**

| | | | |
|---|---|---|---|
| **Sample name:** | 18 minutes | | |
| **Data file:** | 2024-06-05 13-45-00+01-00-07.dx | **Operator:** | SYSTEM |
| **Instrument:** | 1220 Infinity II HPLC | **Injection date:** | 2024-06-05 13:45:54+01:00 |
| **Inj. volume:** | 5.000 µL | **Location:** | 6 |
| **Acq. method:** | Barry's standard method_low flow_higher A.amx | **Type:** | Sample |
| **Processing method:** | *HB Standard method.pmx | | |
| **Manually modified:** | None | | |

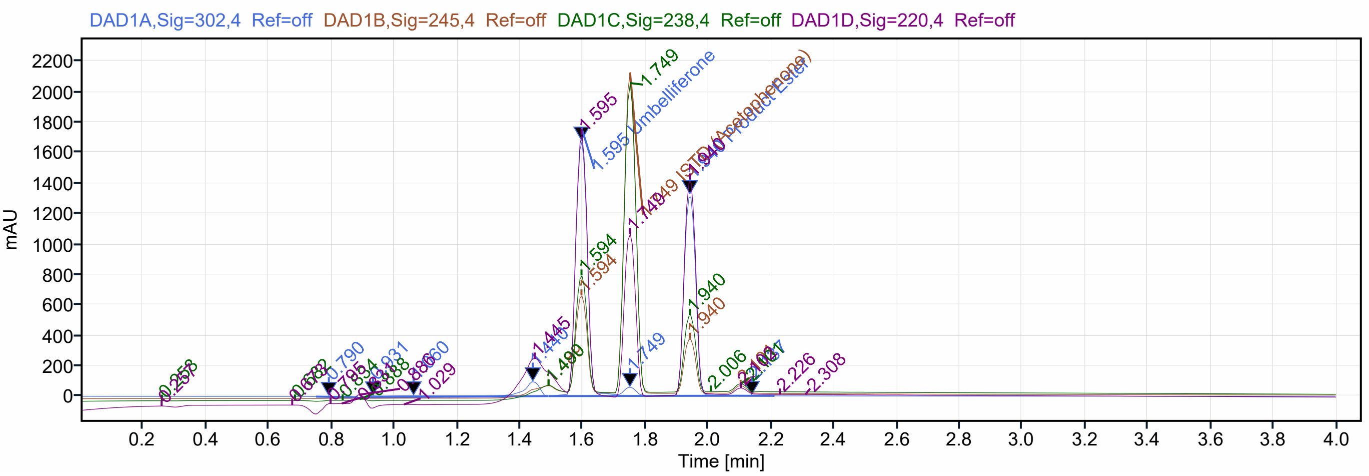


| Sample Name | Name | RT (mins) | Area | Concentration (mg/L) |
|---|---|---|---|---|
| 18 minutes | Pivalic Anhydride | | | |
| 18 minutes | DMAP | | | |
| 18 minutes | Umbelliferone | 1.595 | 4272.2703 | |
| 18 minutes | ISTD (Acetophenone) | 1.749 | 5376.6725 | |
| 18 minutes | Product Ester | 1.940 | 3318.4702 | |

**Sample name:** 21 minutes

**Data file:** 2024-06-05 13-49-57+01-00-08.dx

**Operator:** SYSTEM

**Instrument:** 1220 Infinity II HPLC

**Injection date:** 2024-06-05 13:50:51+01:00

**Inj. volume:** 5.000 µL

**Location:** 7

**Acq. method:** Barry's standard method_low flow_higher A.amx

**Type:** Sample

**Processing method:** *HB Standard method.pmx

**Manually modified:** None

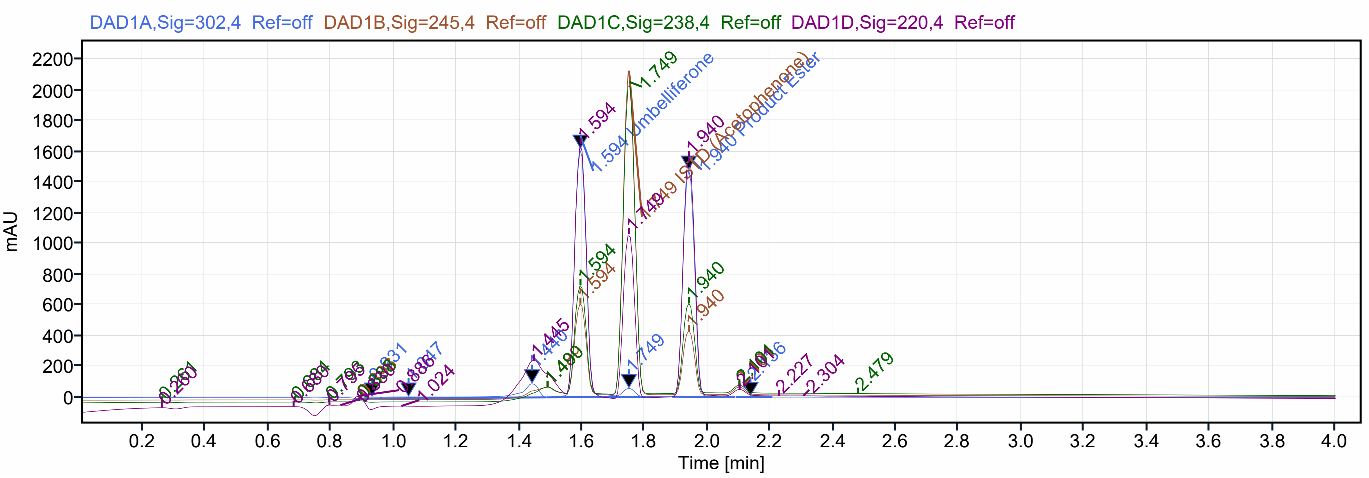


| Sample Name | Name | RT (mins) | Area | Concentration (mg/L) |
|---|---|---|---|---|
| 21 minutes | Pivalic Anhydride | | | |
| 21 minutes | DMAP | | | |
| 21 minutes | Umbelliferone | 1.594 | 4103.0652 | |
| 21 minutes | ISTD (Acetophenone) | 1.749 | 5371.5854 | |
| 21 minutes | Product Ester | 1.940 | 3693.2132 | |

# *Injection Report - By Sample*

Kineticolor

**Sample name:** 24 minutes

**Data file:** 2024-06-05 13-54-55+01-00-09.dx

**Operator:** SYSTEM

**Instrument:** 1220 Infinity II HPLC

**Injection date:** 2024-06-05 13:55:50+01:00

**Inj. volume:** 5.000 µL

**Location:** 8

**Acq. method:** Barry's standard method_low flow_higher A.amx

**Type:** Sample

**Processing method:** *HB Standard method.pmx

**Manually modified:** None

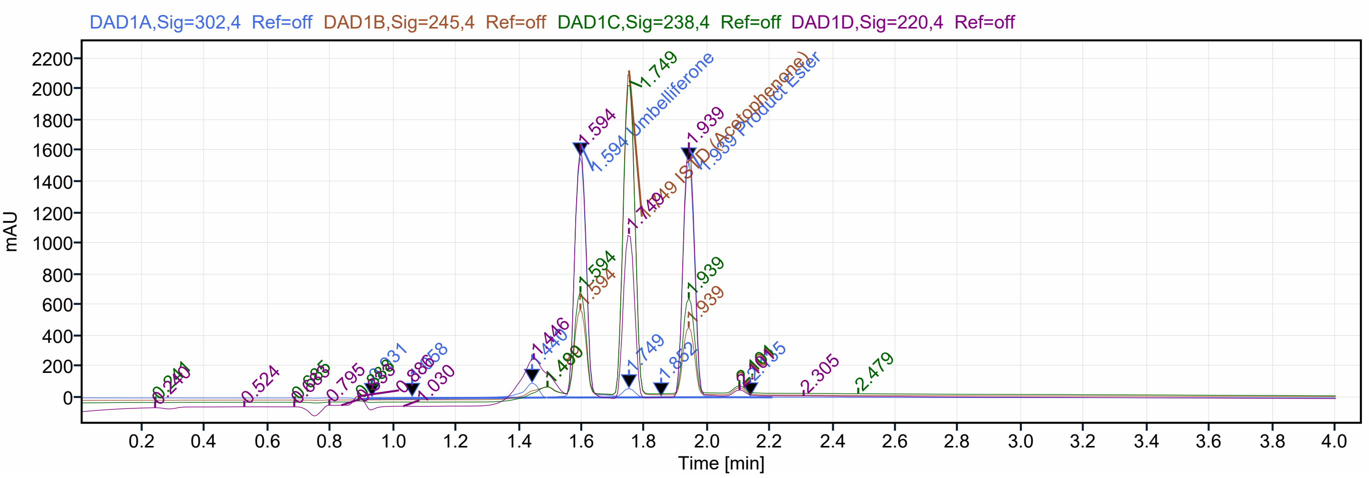


| Sample Name | Name | RT (mins) | Area | Concentration (mg/L) |
|---|---|---|---|---|
| 24 minutes | Pivalic Anhydride | | | |
| 24 minutes | DMAP | | | |
| 24 minutes | Umbelliferone | 1.594 | 3967.2291 | |
| 24 minutes | ISTD (Acetophenone) | 1.749 | 5357.8741 | |
| 24 minutes | Product Ester | 1.939 | 3833.9113 | |

# *Injection Report - By Sample*

**Kineticolor**

| | | | |
|---|---|---|---|
| **Sample name:** | Blank | | |
| **Data file:** | 2024-06-05 13-59-53+01-00-10.dx | **Operator:** | SYSTEM |
| **Instrument:** | 1220 Infinity II HPLC | **Injection date:** | 2024-06-05 14:00:48+01:00 |
| **Inj. volume:** | 5.000 µL | **Location:** | 41 |
| **Acq. method:** | Barry's standard method_low flow_higher A.amx | **Type:** | Sample |
| **Processing method:** | *HB Standard method.pmx | | |
| **Manually modified:** | None | | |

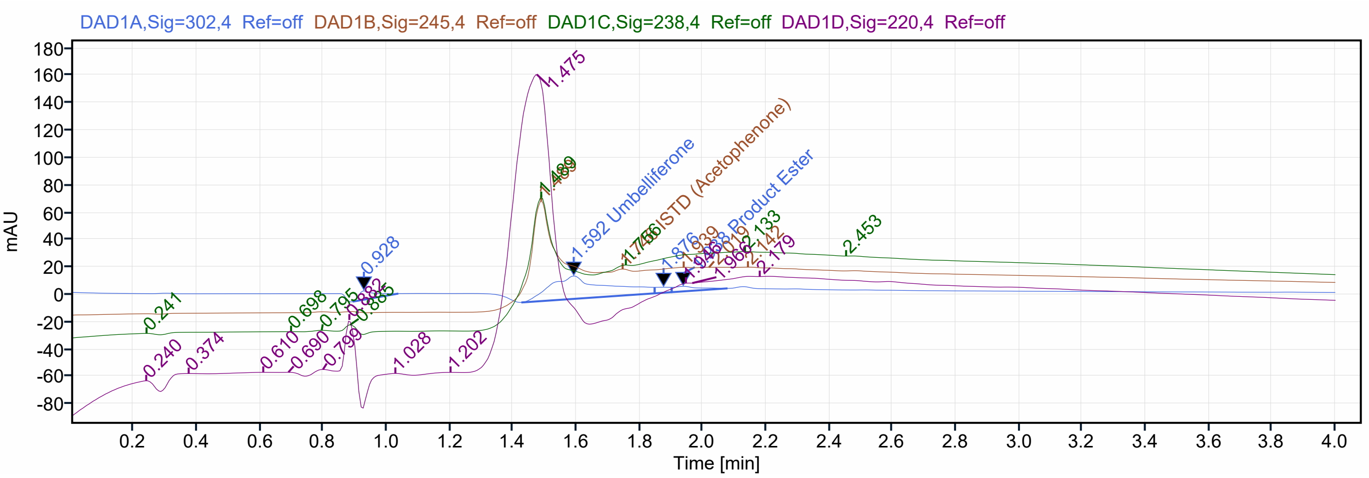


| Sample Name | Name | RT (mins) | Area | Concentration (mg/L) |
|---|---|---|---|---|
| Blank | Pivalic Anhydride | | | |
| Blank | DMAP | | | |
| Blank | Umbelliferone | 1.592 | 209.3481 | |
| Blank | ISTD (Acetophenone) | 1.746 | 139.8835 | |
| Blank | Product Ester | 1.938 | 20.4894 | |

# *Injection Report - By Sample*

Kineticolor

**Sample name:** 27 minutes

**Data file:** 2024-06-05 14-04-53+01-00-11.dx

**Operator:** SYSTEM

**Instrument:** 1220 Infinity II HPLC

**Injection date:** 2024-06-05 14:05:50+01:00

**Inj. volume:** 5.000 µL

**Location:** 9

**Acq. method:** Barry's standard method_low flow_higher A.amx

**Type:** Sample

**Processing method:** *HB Standard method.pmx

**Manually modified:** None

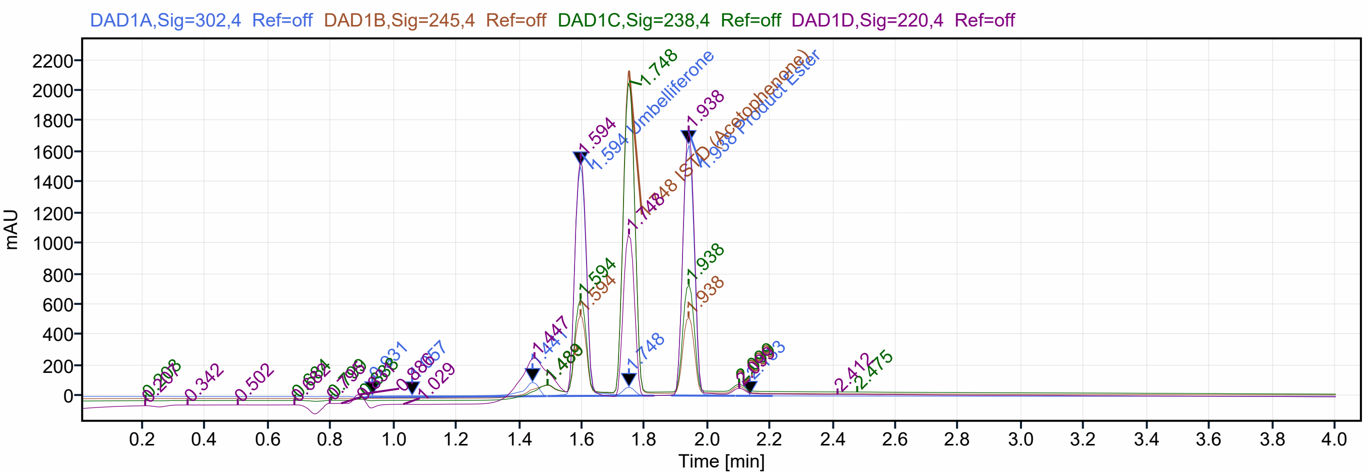


| Sample Name | Name | RT (mins) | Area | Concentration (mg/L) |
|---|---|---|---|---|
| 27 minutes | Pivalic Anhydride | | | |
| 27 minutes | DMAP | | | |
| 27 minutes | Umbelliferone | 1.594 | 3823.0681 | |
| 27 minutes | ISTD (Acetophenone) | 1.748 | 5378.6890 | |
| 27 minutes | Product Ester | 1.938 | 4125.0589 | |

# *Injection Report - By Sample*

**Kineticolor**

| | | | |
|---|---|---|---|
| **Sample name:** | 30 minutes | | |
| **Data file:** | 2024-06-05 14-09-53+01-00-12.dx | **Operator:** | SYSTEM |
| **Instrument:** | 1220 Infinity II HPLC | **Injection date:** | 2024-06-05 14:10:54+01:00 |
| **Inj. volume:** | 5.000 µL | **Location:** | 10 |
| **Acq. method:** | Barry's standard method_low flow_higher A.amx | **Type:** | Sample |
| **Processing method:** | *HB Standard method.pmx | | |
| **Manually modified:** | None | | |

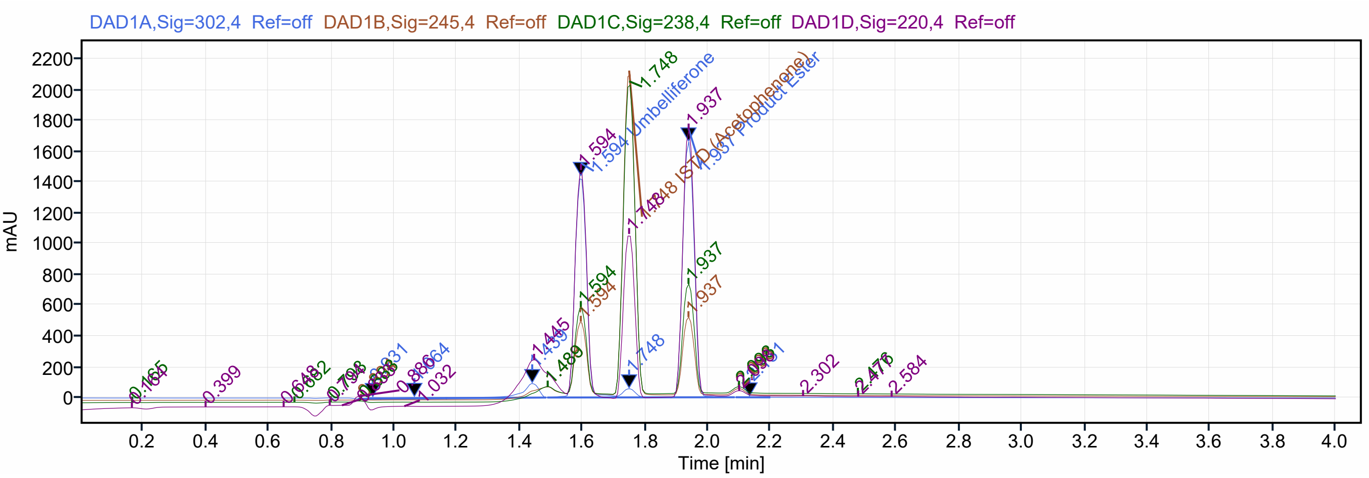


| Sample Name | Name | RT (mins) | Area | Concentration (mg/L) |
|---|---|---:|---:|---:|
| 30 minutes | Pivalic Anhydride | | | |
| 30 minutes | DMAP | | | |
| 30 minutes | Umbelliferone | 1.594 | 3654.1121 | |
| 30 minutes | ISTD (Acetophenone) | 1.748 | 5368.9907 | |
| 30 minutes | Product Ester | 1.937 | 4180.3006 | |

# *Injection Report - By Sample*

**Kineticolor**

| | | | |
|---|---|---|---|
| **Sample name:** | 35 minutes | | |
| **Data file:** | 2024-06-05 14-14-57+01-00-13.dx | **Operator:** | SYSTEM |
| **Instrument:** | 1220 Infinity II HPLC | **Injection date:** | 2024-06-05 14:15:53+01:00 |
| **Inj. volume:** | 5.000 µL | **Location:** | 11 |
| **Acq. method:** | Barry's standard method_low flow_higher A.amx | **Type:** | Sample |
| **Processing method:** | *HB Standard method.pmx | | |
| **Manually modified:** | None | | |

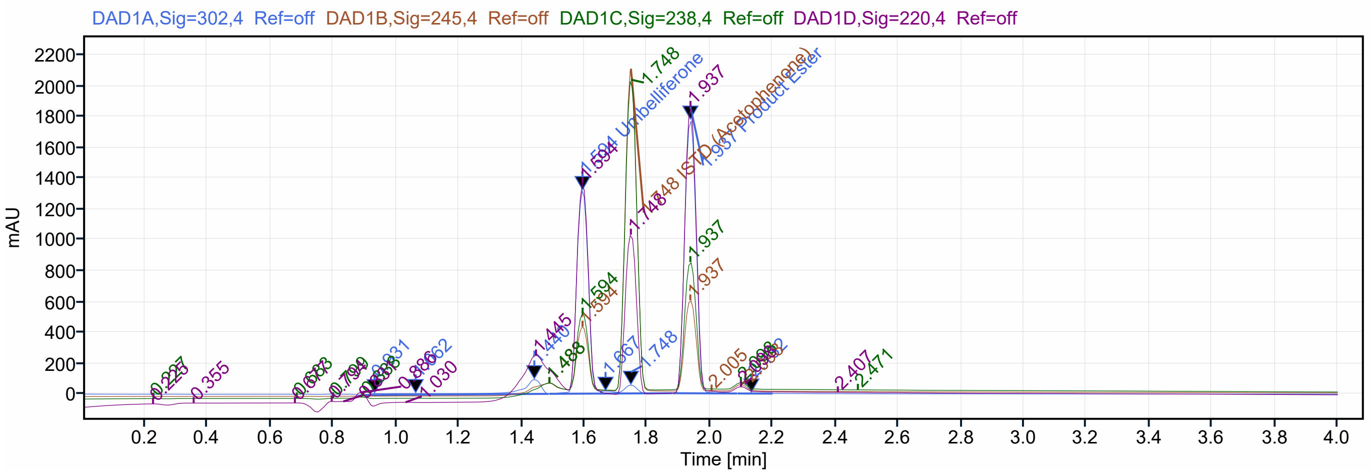


| Sample Name | Name | RT (mins) | Area | Concentration (mg/L) |
|---|---|---|---|---|
| 35 minutes | Pivalic Anhydride | | | |
| 35 minutes | DMAP | | | |
| 35 minutes | Umbelliferone | 1.594 | 3311.5145 | |
| 35 minutes | ISTD (Acetophenone) | 1.748 | 5322.4140 | |
| 35 minutes | Product Ester | 1.937 | 4468.9020 | |

# *Injection Report - By Sample*

**Kineticolor**

| | | | |
|---|---|---|---|
| **Sample name:** | 40 minutes | | |
| **Data file:** | 2024-06-05 14-19-57+01-00-14.dx | **Operator:** | SYSTEM |
| **Instrument:** | 1220 Infinity II HPLC | **Injection date:** | 2024-06-05 14:20:51+01:00 |
| **Inj. volume:** | 5.000 µL | **Location:** | 12 |
| **Acq. method:** | Barry's standard method_low flow_higher A.amx | **Type:** | Sample |
| **Processing method:** | *HB Standard method.pmx | | |
| **Manually modified:** | None | | |

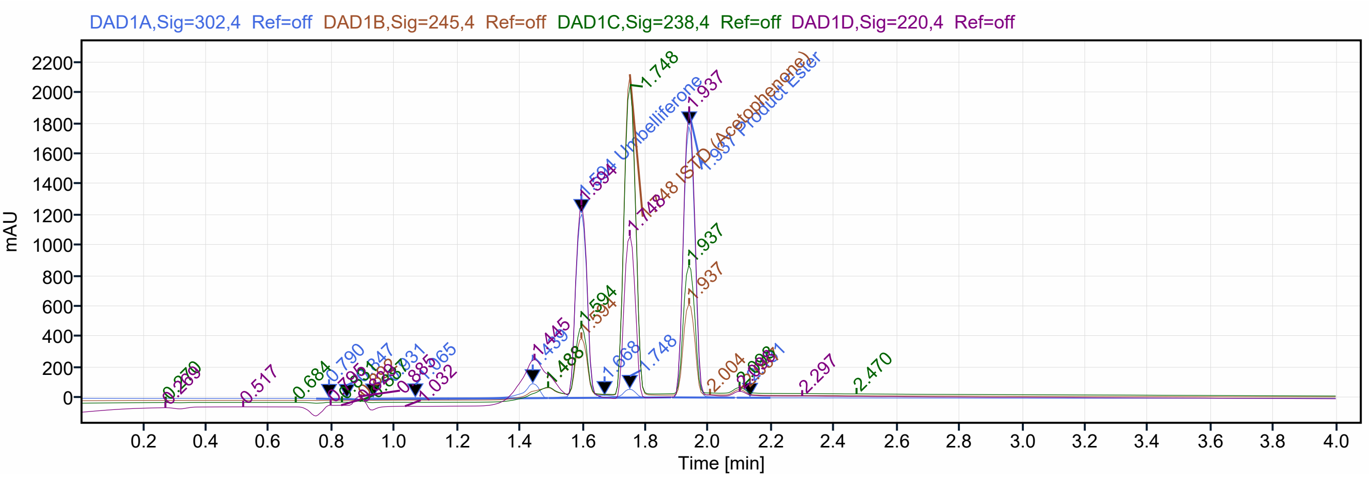


| Sample Name | Name | RT (mins) | Area | Concentration (mg/L) |
|---|---|---|---|---|
| 40 minutes | Pivalic Anhydride | | | |
| 40 minutes | DMAP | | | |
| 40 minutes | Umbelliferone | 1.594 | 3029.6212 | |
| 40 minutes | ISTD (Acetophenone) | 1.748 | 5351.5456 | |
| 40 minutes | Product Ester | 1.937 | 4489.9948 | |

# *Injection Report - By Sample*

Kineticolor

**Sample name:** 45 minutes

**Data file:** 2024-06-05 14-24-55+01-00-15.dx

**Operator:** SYSTEM

**Instrument:** 1220 Infinity II HPLC

**Injection date:** 2024-06-05 14:25:49+01:00

**Inj. volume:** 5.000 µL

**Location:** 13

**Acq. method:** Barry's standard method_low flow_higher A.amx

**Type:** Sample

**Processing method:** *HB Standard method.pmx

**Manually modified:** None

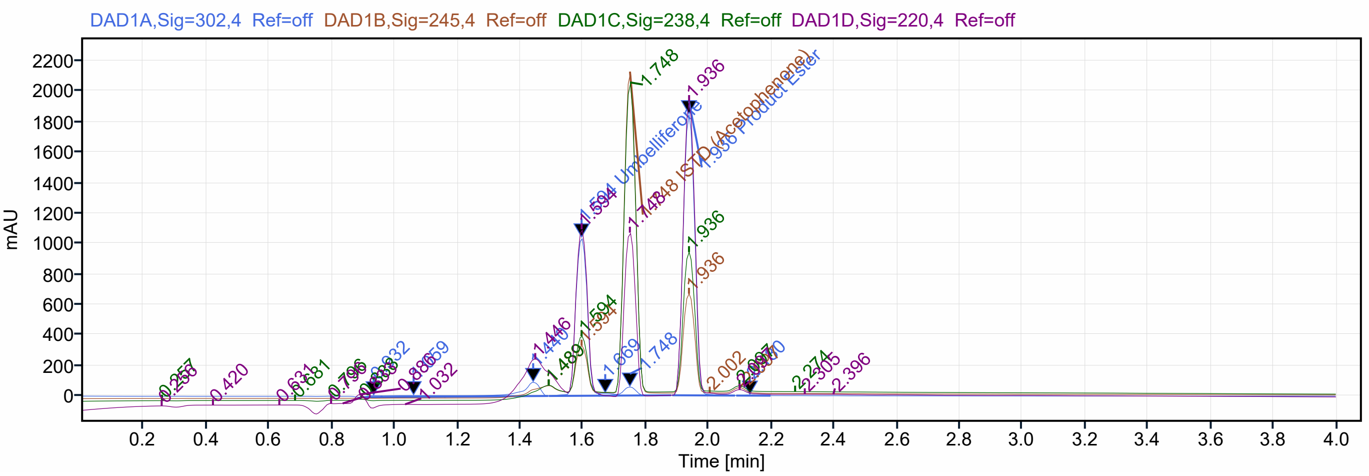


| Sample Name | Name | RT (mins) | Area | Concentration (mg/L) |
|---|---|---|---|---|
| 45 minutes | Pivalic Anhydride | | | |
| 45 minutes | DMAP | | | |
| 45 minutes | Umbelliferone | 1.594 | 2604.6119 | |
| 45 minutes | ISTD (Acetophenone) | 1.748 | 5375.1416 | |
| 45 minutes | Product Ester | 1.936 | 4657.7776 | |

# *Injection Report - By Sample*

**Kineticolor**

| | | | |
|---|---|---|---|
| **Sample name:** | 50 minutes | | |
| **Data file:** | 2024-06-05 14-29-53+01-00-16.dx | **Operator:** | SYSTEM |
| **Instrument:** | 1220 Infinity II HPLC | **Injection date:** | 2024-06-05 14:30:48+01:00 |
| **Inj. volume:** | 5.000 µL | **Location:** | 14 |
| **Acq. method:** | Barry's standard method_low flow_higher A.amx | **Type:** | Sample |
| **Processing method:** | *HB Standard method.pmx | | |
| **Manually modified:** | None | | |

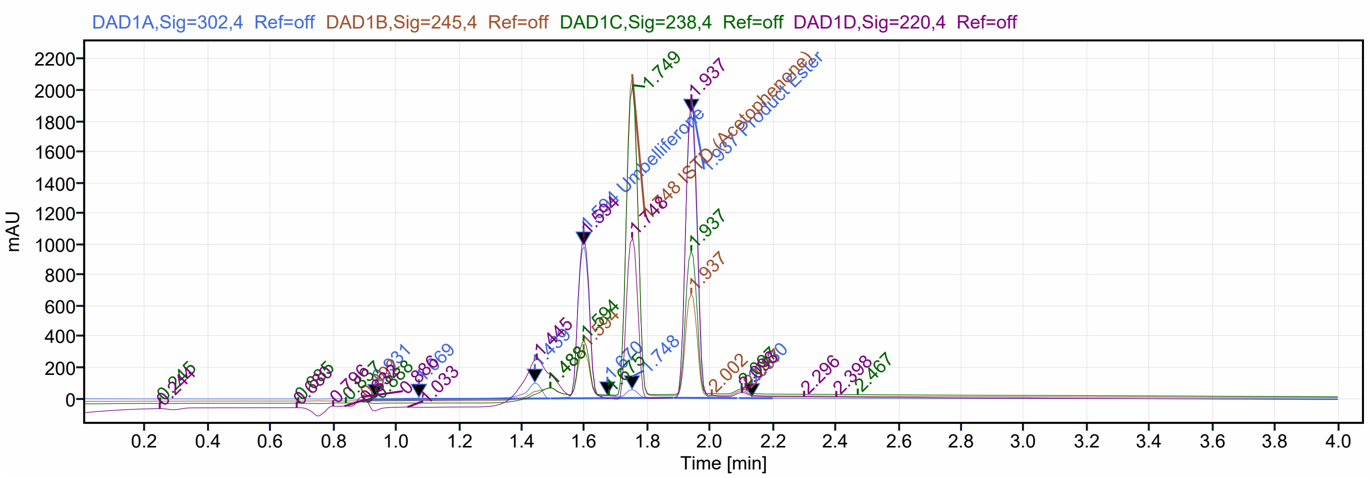


| Sample Name | Name | RT (mins) | Area | Concentration (mg/L) |
|---|---|---|---|---|
| 50 minutes | Pivalic Anhydride | | | |
| 50 minutes | DMAP | | | |
| 50 minutes | Umbelliferone | 1.594 | 2478.2530 | |
| 50 minutes | ISTD (Acetophenone) | 1.748 | 5305.6270 | |
| 50 minutes | Product Ester | 1.937 | 4669.9370 | |

# *Injection Report - By Sample*

**Kineticolor**

**Sample name:** 55 minutes

**Data file:** 2024-06-05 14-34-52+01-00-17.dx

**Operator:** SYSTEM

**Instrument:** 1220 Infinity II HPLC

**Injection date:** 2024-06-05 14:35:45+01:00

**Inj. volume:** 5.000 µL

**Location:** 15

**Acq. method:** Barry's standard method_low flow_higher A.amx

**Type:** Sample

**Processing method:** *HB Standard method.pmx

**Manually modified:** None

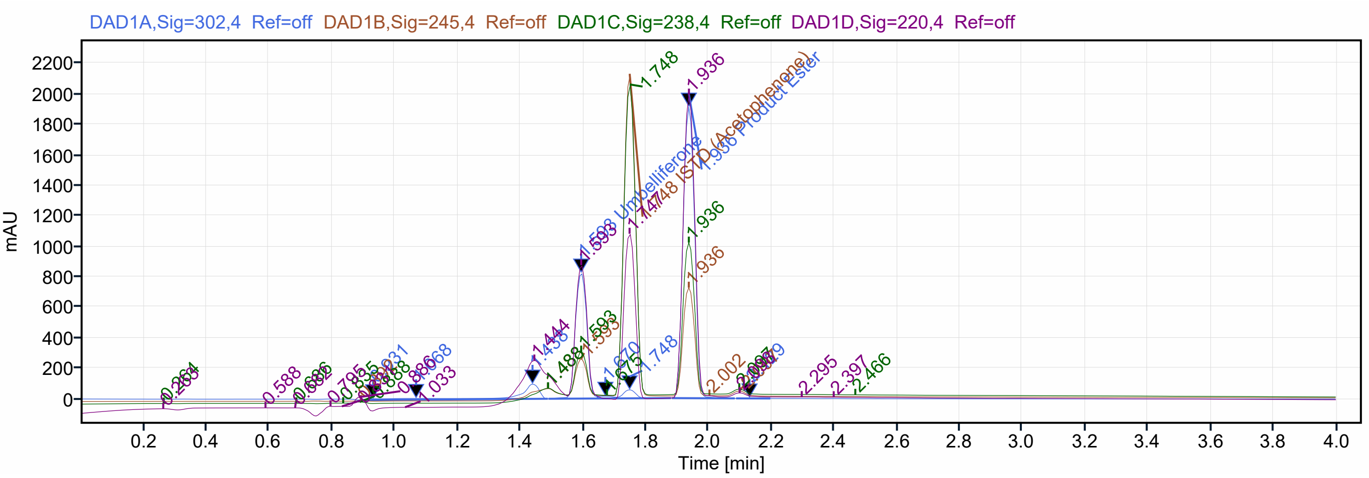


| Sample Name | Name | RT (mins) | Area | Concentration (mg/L) |
|---|---|---|---|---|
| 55 minutes | Pivalic Anhydride | | | |
| 55 minutes | DMAP | | | |
| 55 minutes | Umbelliferone | 1.593 | 2070.3827 | |
| 55 minutes | ISTD (Acetophenone) | 1.748 | 5380.5124 | |
| 55 minutes | Product Ester | 1.936 | 4835.4680 | |

# *Injection Report - By Sample*

**Kineticolor**

| | | | |
|---|---|---|---|
| **Sample name:** | 60 minutes | | |
| **Data file:** | 2024-06-05 14-39-49+01-00-18.dx | **Operator:** | SYSTEM |
| **Instrument:** | 1220 Infinity II HPLC | **Injection date:** | 2024-06-05 14:40:44+01:00 |
| **Inj. volume:** | 5.000 µL | **Location:** | 16 |
| **Acq. method:** | Barry's standard method_low flow_higher A.amx | **Type:** | Sample |
| **Processing method:** | *HB Standard method.pmx | | |
| **Manually modified:** | None | | |

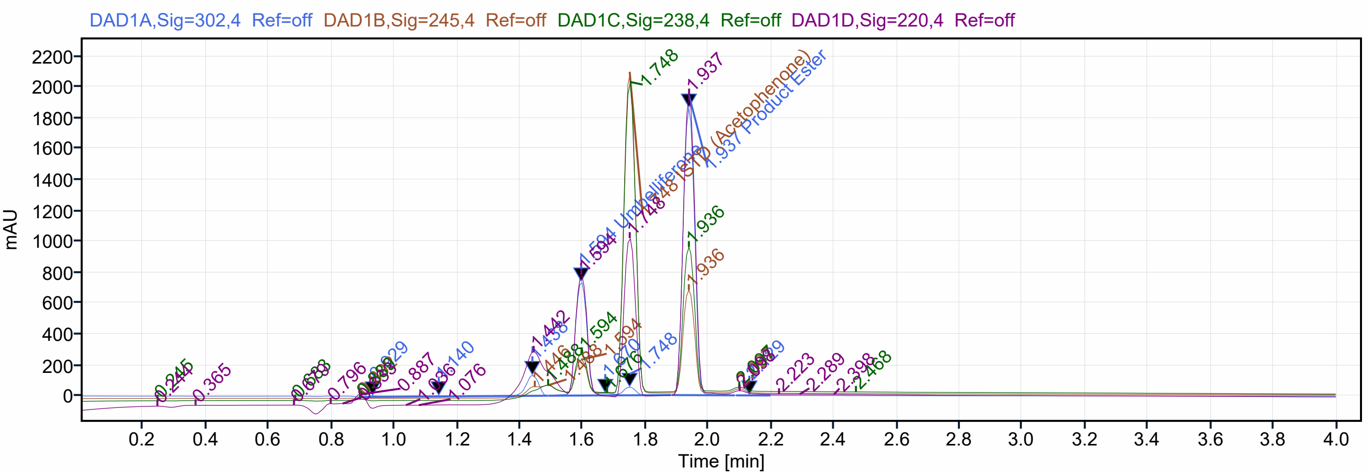


| Sample Name | Name | RT (mins) | Area | Concentration (mg/L) |
|---|---|---|---|---|
| 60 minutes | Pivalic Anhydride | | | |
| 60 minutes | DMAP | | | |
| 60 minutes | Umbelliferone | 1.594 | 1852.8964 | |
| 60 minutes | ISTD (Acetophenone) | 1.748 | 5241.9683 | |
| 60 minutes | Product Ester | 1.937 | 4702.2950 | |

# *Injection Report - By Sample*

**Kineticolor**

| | | | |
|---|---|---|---|
| **Sample name:** | Blank | | |
| **Data file:** | 2024-06-05 14-44-48+01-00-19.dx | **Operator:** | SYSTEM |
| **Instrument:** | 1220 Infinity II HPLC | **Injection date:** | 2024-06-05 14:45:42+01:00 |
| **Inj. volume:** | 5.000 µL | **Location:** | 41 |
| **Acq. method:** | Barry's standard method_low flow_higher A.amx | **Type:** | Sample |
| **Processing method:** | *HB Standard method.pmx | | |
| **Manually modified:** | None | | |

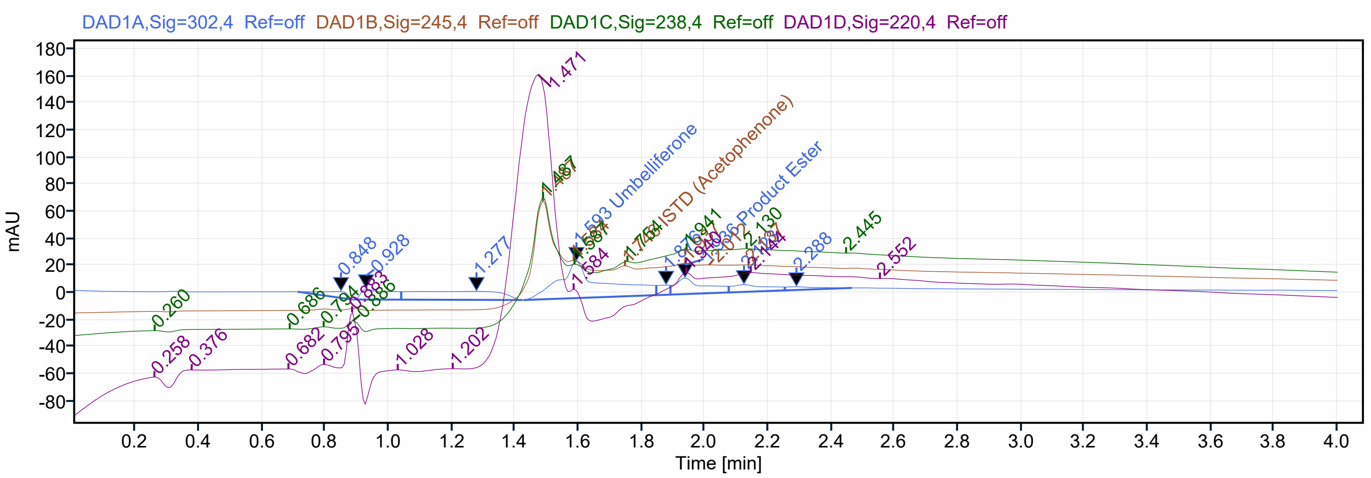


| Sample Name | Name | RT (mins) | Area | Concentration (mg/L) |
|---|---|---|---|---|
| Blank | Pivalic Anhydride | | | |
| Blank | DMAP | | | |
| Blank | Umbelliferone | 1.593 | 265.3624 | |
| Blank | ISTD (Acetophenone) | 1.746 | 32.7750 | |
| Blank | Product Ester | 1.936 | 76.9025 | |
