# Supplementary figures and images for: Parallel and High Throughput Reaction Monitoring with Computer Vision (part 3 of 3)
Source: Angew Chem Int Ed Engl. 2024 Oct 31;64(1):e202413395. doi: 10.1002/anie.202413395 (PMC11701362; doi:10.1002/anie.202413395)

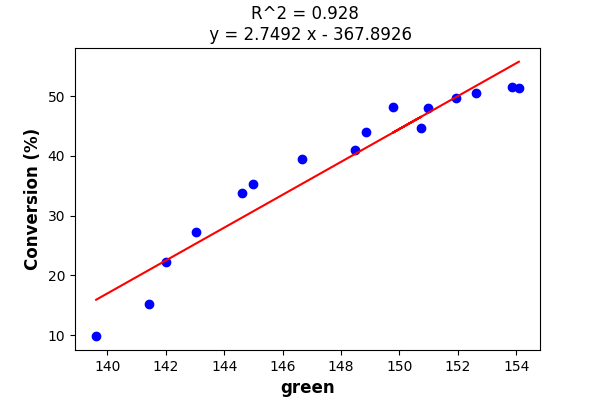

Supplement: Supplementary file 3 — Supporting Information [file ANIE-64-e202413395-s003.zip › Supporting Info - Machine readable data part 2/Figure 10 - esterification and mutual information/Mutual Information and Regression outputs/Regression charts/Regression_green.png]

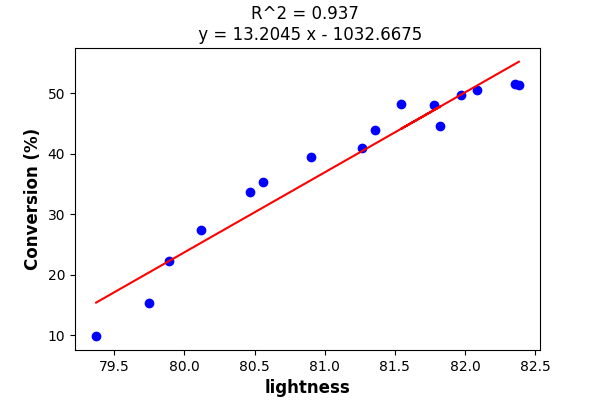

Supplement: Supplementary file 3 — Supporting Information [file ANIE-64-e202413395-s003.zip › Supporting Info - Machine readable data part 2/Figure 10 - esterification and mutual information/Mutual Information and Regression outputs/Regression charts/Regression_lightness.png]

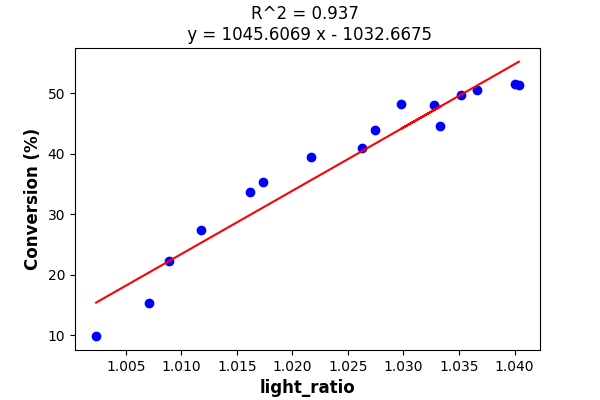

Supplement: Supplementary file 3 — Supporting Information [file ANIE-64-e202413395-s003.zip › Supporting Info - Machine readable data part 2/Figure 10 - esterification and mutual information/Mutual Information and Regression outputs/Regression charts/Regression_light_ratio.png]

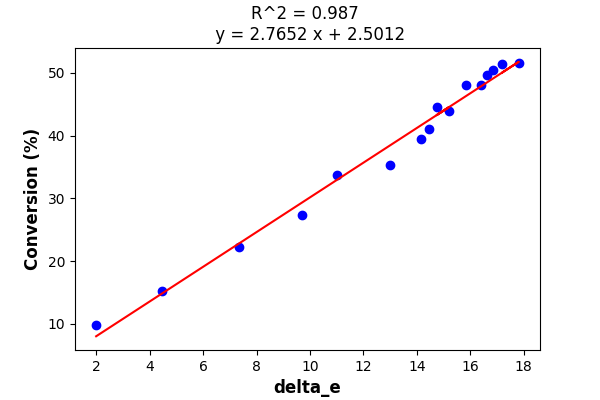

Supplement: Supplementary file 3 — Supporting Information [file ANIE-64-e202413395-s003.zip › Supporting Info - Machine readable data part 2/Figure 10 - esterification and mutual information/Mutual Information and Regression outputs/Regression charts/Regression_delta_e.png]

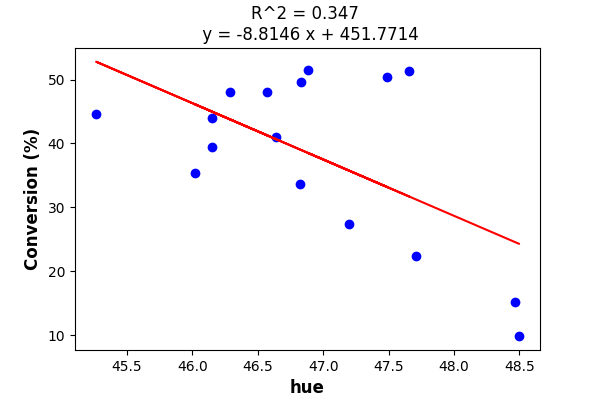

Supplement: Supplementary file 3 — Supporting Information [file ANIE-64-e202413395-s003.zip › Supporting Info - Machine readable data part 2/Figure 10 - esterification and mutual information/Mutual Information and Regression outputs/Regression charts/Regression_hue.png]
