# Supplementary material for: Parallel and High Throughput Reaction Monitoring with Computer Vision
Source: Angew Chem Int Ed Engl. 2024 Oct 31;64(1):e202413395. doi: 10.1002/anie.202413395 (PMC11701362; doi:10.1002/anie.202413395)
Supplement: Supplementary file 3 — Supporting Information [file ANIE-64-e202413395-s003.zip › Supporting Info - Machine readable data part 2/Figure 10 - esterification and mutual information/NMR_/RE-processed/Umbelliferone NMR aromatic.pdf]

Person wlb21183
HB UMB
@proton CDCl3 {C:\NMRdata} MR 25

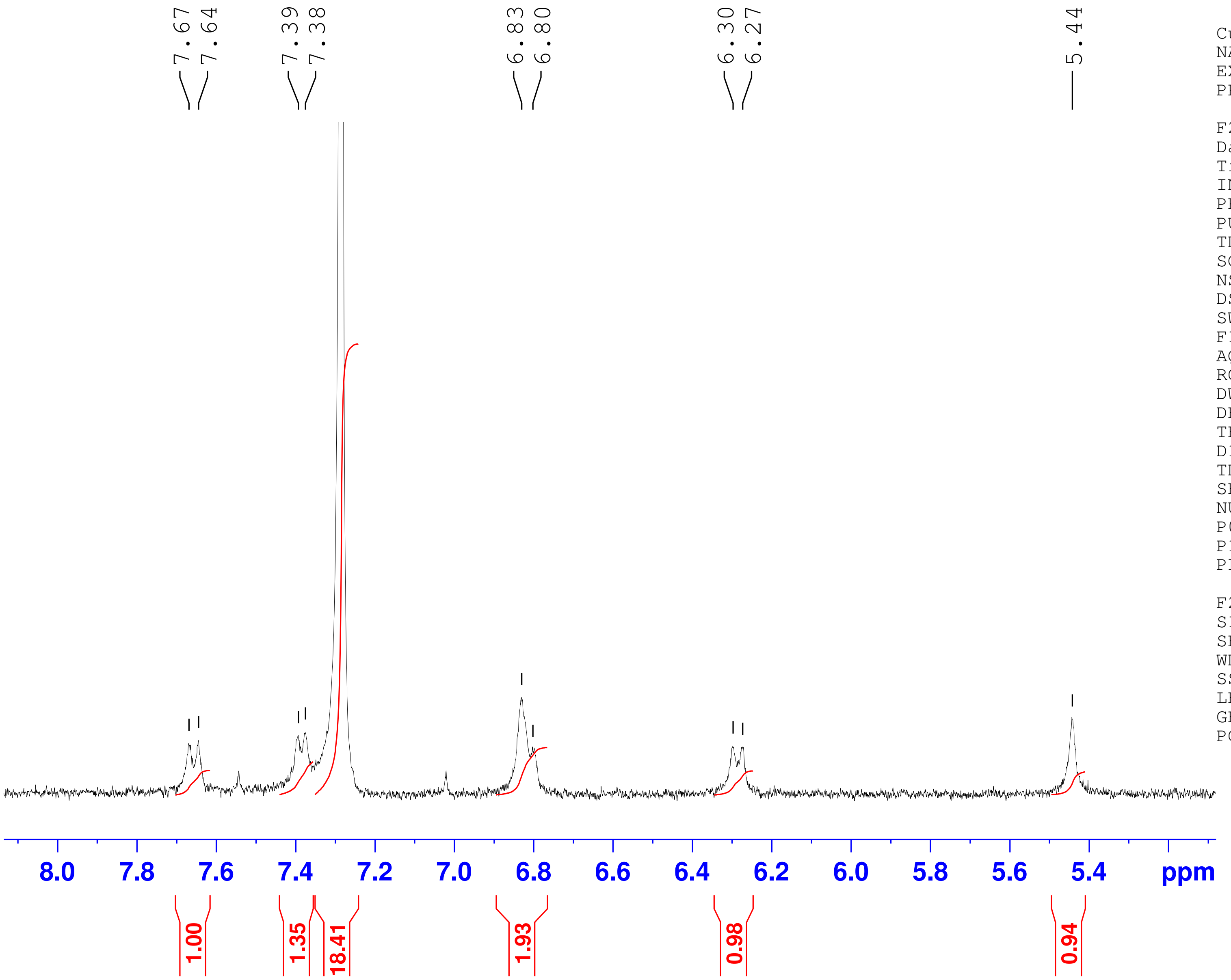

Current Data Parameters

NAME	D385451
EXPNO	1
PROCNO	1

F2 - Acquisition Parameters

Date_	20240222
Time	18.07 h
INSTRUM	spect
PROBHD	Z122623_0021 (
PULPROG	zg30
TD	32564
SOLVENT	CDCl3
NS	4
DS	2
SWH	8223.685 Hz
FIDRES	0.505078 Hz
AQ	1.9798912 sec
RG	128
DW	60.800 usec
DE	10.00 usec
TE	300.0 K
D1	2.00000000 sec
TD0	1
SFO1	400.1324710 MHz
NUC1	1H
P0	4.00 usec
P1	12.00 usec
PLW1	7.19999981 W

F2 - Processing parameters

SI	32768
SF	400.1300000 MHz
WDW	EM
SSB	0
LB	0.30 Hz
GB	0
PC	1.00
