# Supplementary material for: Parallel and High Throughput Reaction Monitoring with Computer Vision
Source: Angew Chem Int Ed Engl. 2024 Oct 31;64(1):e202413395. doi: 10.1002/anie.202413395 (PMC11701362; doi:10.1002/anie.202413395)
Supplement: Supplementary file 3 — Supporting Information [file ANIE-64-e202413395-s003.zip › Supporting Info - Machine readable data part 2/Figure 10 - esterification and mutual information/NMR_/RE-processed/Umbelliferone NMR full.pdf]

Person wlb21183
HB UMB
@proton CDCl3 {C:\NMRdata} MR 25

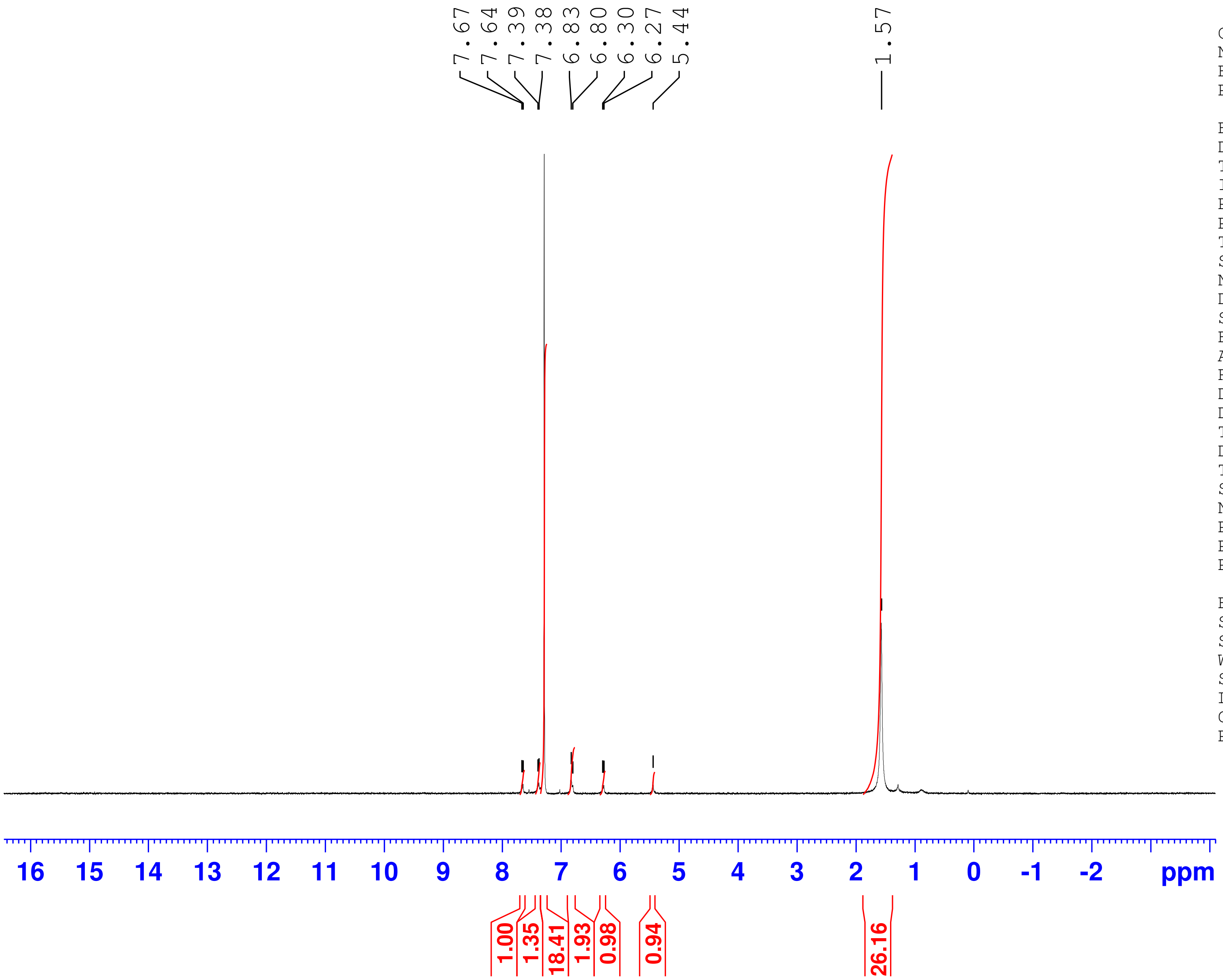

Current Data Parameters
NAME D385451
EXPNO 1
PROCNO 1

F2 - Acquisition Parameters
Date_ 20240222
Time 18.07 h
INSTRUM spect
PROBHD Z122623_0021 (
PULPROG zg30
TD 32564
SOLVENT CDCl3
NS 4
DS 2
SWH 8223.685 Hz
FIDRES 0.505078 Hz
AQ 1.9798912 sec
RG 128
DW 60.800 usec
DE 10.00 usec
TE 300.0 K
D1 2.00000000 sec
TD0 1
SFO1 400.1324710 MHz
NUC1 1H
P0 4.00 usec
P1 12.00 usec
PLW1 7.19999981 W

F2 - Processing parameters
SI 32768
SF 400.1300000 MHz
WDW EM
SSB 0
LB 0.30 Hz
GB 0
PC 1.00
