# Supplementary material for: Parallel and High Throughput Reaction Monitoring with Computer Vision
Source: Angew Chem Int Ed Engl. 2024 Oct 31;64(1):e202413395. doi: 10.1002/anie.202413395 (PMC11701362; doi:10.1002/anie.202413395)
Supplement: Supplementary file 3 — Supporting Information [file ANIE-64-e202413395-s003.zip › Supporting Info - Machine readable data part 2/Figure 10 - esterification and mutual information/NMR_/RE-processed/product closeup.pdf]

Person wlb21183
HB 3.6.2a
@proton CDCl3 {C:\NMRdata} MR 15

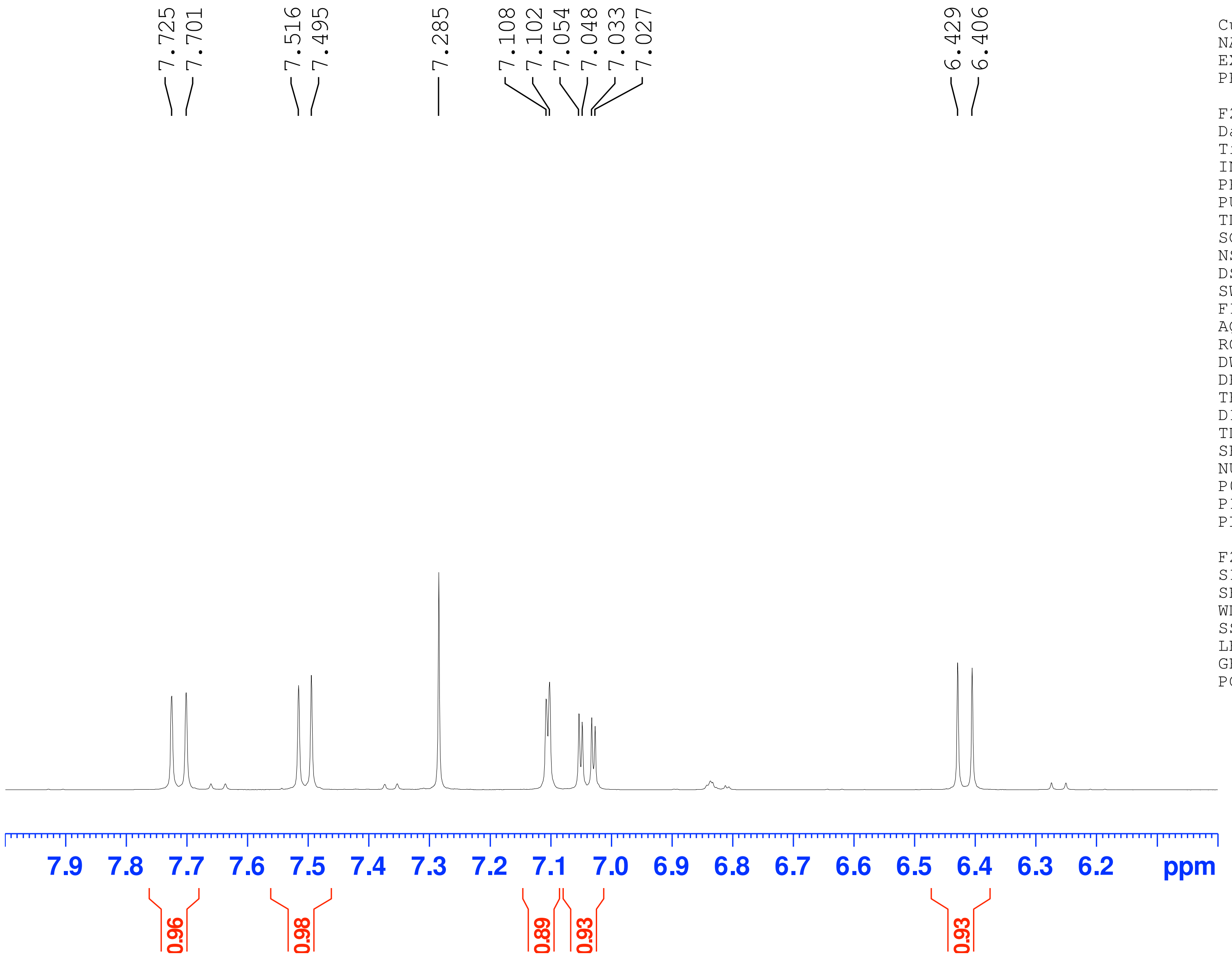

BRUKER

Current Data Parameters
NAME NMR 3.6.2 A Processec
EXPNO 1
PROCNO 1

F2 - Acquisition Parameters

Date_	20240109	
Time	15.52	h
INSTRUM	spect	
PROBHD	Z122623_0021 (	
PULPROG	zg30	
TD	32564	
SOLVENT	CDCl3	
NS	4	
DS	2	
SWH	8223.685	Hz
FIDRES	0.505078	Hz
AQ	1.9798912	sec
RG	128	
DW	60.800	usec
DE	10.00	usec
TE	300.0	K
D1	2.00000000	sec
TD0	1	
SFO1	400.1324710	MHz
NUC1	1H	
P0	4.00	usec
P1	12.00	usec
PLW1	7.19999981	W

F2 - Processing parameters

SI	32768	
SF	400.1300000	MHz
WDW	EM	
SSB	0	
LB	0.30	Hz
GB	0	
PC	1.00	
